# Clinical application of whole-genome sequencing of solid tumors for precision oncology

**DOI:** 10.1038/s12276-024-01288-x

**Published:** 2024-08-13

**Authors:** Ryul Kim, Seokhwi Kim, Brian Baek-Lok Oh, Woo Sik Yu, Chang Woo Kim, Hoon Hur, Sang-Yong Son, Min Jae Yang, Dae Sung Cho, Taeyang Ha, Subin Heo, Jeon Yeob Jang, Jae Sung Yun, Kyu-Sung Kwack, Jai Keun Kim, Jimi Huh, Sun Gyo Lim, Sang-Uk Han, Hyun Woo Lee, Ji Eun Park, Chul-Ho Kim, Jin Roh, Young Wha Koh, Dakeun Lee, Jang-Hee Kim, Gil Ho Lee, Choong-Kyun Noh, Yun Jung Jung, Ji Won Park, Seungsoo Sheen, Mi Sun Ahn, Yong Won Choi, Tae-Hwan Kim, Seok Yun Kang, Jin-Hyuk Choi, Soo Yeon Baek, Kee Myung Lee, Sun Il Kim, Sung Hyun Noh, Se-Hyuk Kim, Hyemin Hwang, Eunjung Joo, Shinjung Lee, Jong-Yeon Shin, Ji-Young Yun, Junggil Park, Kijong Yi, Youngoh Kwon, Won-Chul Lee, Hansol Park, Joonoh Lim, Boram Yi, Jaemo Koo, June-Young Koh, Sangmoon Lee, Yuna Lee, Bo-Rahm Lee, Erin Connolly-Strong, Young Seok Ju, Minsuk Kwon

**Affiliations:** 1 Inocras, San Diego, CA USA; 2 Department of Pathology, Ajou University School of Medicine, Suwon, Republic of Korea; 3 Department of Thoracic and Cardiovascular Surgery, Ajou University School of Medicine, Suwon, Republic of Korea; 4 Department of Surgery, Ajou University School of Medicine, Suwon, Republic of Korea; 5 Department of Gastroenterology, Ajou University School of Medicine, Suwon, Republic of Korea; 6 Department of Urology, Ajou University School of Medicine, Suwon, Republic of Korea; 7 Department of Radiology, Ajou University School of Medicine, Suwon, Republic of Korea; 8 Department of Otolaryngology, Ajou University School of Medicine, Suwon, Republic of Korea; 9 Department of Hematology-Oncology, Ajou University School of Medicine, Gyeonggi-do, Republic of Korea; 10 Department of Pulmonary and Critical Care Medicine, Ajou University School of Medicine, Suwon, Republic of Korea; 11 Department of Neurosurgery, Ajou University School of Medicine, Gyeonggi-do, Republic of Korea; 12 Division of Hematology-Oncology, Department of Medicine, Samsung Medical Center, Sungkyunkwan University School of Medicine, Seoul, Republic of Korea; 13 Present Address: Department of Radiology and Research Institute of Radiology, Asan Medical Center, University of Ulsan College of Medicine, Seoul, Republic of Korea

**Keywords:** Cancer genomics, Next-generation sequencing

## Abstract

Genomic alterations in tumors play a pivotal role in determining their clinical trajectory and responsiveness to treatment. Targeted panel sequencing (TPS) has served as a key clinical tool over the past decade, but advancements in sequencing costs and bioinformatics have now made whole-genome sequencing (WGS) a feasible single-assay approach for almost all cancer genomes in clinical settings. This paper reports on the findings of a prospective, single-center study exploring the real-world clinical utility of WGS (tumor and matched normal tissues) and has two primary objectives: (1) assessing actionability for therapeutic options and (2) providing clarity for clinical questions. Of the 120 patients with various solid cancers who were enrolled, 95 (79%) successfully received genomic reports within a median of 11 working days from sampling to reporting. Analysis of these 95 WGS reports revealed that 72% (68/95) yielded clinically relevant insights, with 69% (55/79) pertaining to therapeutic actionability and 81% (13/16) pertaining to clinical clarity. These benefits include the selection of informed therapeutics and/or active clinical trials based on the identification of driver mutations, tumor mutational burden (TMB) and mutational signatures, pathogenic germline variants that warrant genetic counseling, and information helpful for inferring cancer origin. Our findings highlight the potential of WGS as a comprehensive tool in precision oncology and suggests that it should be integrated into routine clinical practice to provide a complete image of the genomic landscape to enable tailored cancer management.

## Comprehensive genomic profiling: transforming precision oncology in clinical practice

Personalized medicine customizes cancer treatment to each patient, using molecular profiling of tumors to find specific genetic changes that can guide treatment. Despite progress, the practical use of whole-genome sequencing in clinical settings is still not fully explored. This study examines the use of WGS for cancer patients, aiming to make it a regular part of care. The study involved 120 participants with various solid tumors, using the CancerVision^TM^ for sequencing. Researchers conclude that WGS is a valuable tool in precision oncology, offering insights that can significantly impact treatment strategies. The study marks progress in integration of genomic medicine into clinical practice, showcasing the feasibility and benefits of WGS in a real-world hospital setting. Future research may further establish WGS as a standard part of cancer care, potentially changing how we approach treatment for different tumor types. This summary was initially drafted using artificial intelligence, then revised and fact-checked by the author.

## Introduction

Personalized medicine aims to customize cancer treatment strategies for each individual, ensuring interventions that yield the best patient outcomes. Central to this approach is molecular profiling of tumors. Molecular profiling has been established as an essential tool in clinical practice for identifying targetable alterations^1^. Projects such as The Cancer Genome Atlas and International Cancer Genome Consortium have unveiled both prevalent and rare genetic anomalies^2,3^, with many presenting actionable clinical implications. These genetic deviations not only suggest viable treatment paths but also provide insights into the severity of the disease, assisting physicians in customizing treatment plans^4^. For instance, the presence of *ERBB2* (HER2) amplification in breast cancers is associated with more aggressive tumor characteristics and a poorer prognosis^5^. However, the introduction of HER2 inhibitors, such as trastuzumab, has markedly enhanced patient outcomes in these scenarios^6^. This exemplifies the critical role of identifying specific molecular alterations, such as HER2 status in breast cancer, as a cornerstone for informed clinical decision-making in oncology. To this end, the Food and Drug Administration recognizes many conventional molecular diagnostic techniques, including immunohistochemistry, in situ hybridization, and conventional DNA sequencing^7^.

More recently, next-generation sequencing (NGS) techniques have emerged as more efficient and comprehensive molecular diagnostic methods because they can detect various genetic alterations simultaneously from single tests^8^. Indeed, NGS-based comprehensive genomic profiling (CGP) is rapidly transitioning into a standard, go-to technique in clinical practice, highlighted by its incorporation into the European Society for Medical Oncology guidelines^9^. In this context, the NGS technique is recommended as an alternative to conventional testing methods for the detection of specific actionable mutations in advanced non-squamous non-small cell lung, prostate, ovarian, cholangiocarcinoma, and colon cancer^9^. Furthermore, NGS is recommended for evaluating the tumor mutational burden (TMB), a molecular marker for the response to pembrolizumab (immune checkpoint inhibitor) in a few cancer types (cervical, specific neuroendocrine, salivary, thyroid, and vulvar cancers)^9^.

Until recently, targeted panel sequencing (TPS) platforms have been the predominant methods used for CGP in clinical settings. TPS technology conventionally focuses on particular exons and introns of 50–500 known cancer genes (typically representing 0.01%–0.1% of the genome). Although TPS technology offers advantages, such as minimal sequencing throughput (approximately 1–10 Gb) and simple bioinformatic processes, it has several limitations. Notably, TPS exhibits reduced detection sensitivity for off-target driver mutations, complex genomic rearrangements, and mutational signatures^10,11^. This narrow coverage, paired with delays in updating the panel design, might overlook emerging data and new actionable markers not on the panel. Because TPS does not typically assess matched normal tissues, the differentiation of somatically acquired mutations from germline polymorphisms is often challenging^12^.

These considerations underscore the potential utility of whole-genome sequencing (WGS) in the clinic. WGS provides comprehensive information on somatic point mutations, copy number alterations (CNAs), and structural variations (SVs), and this information is essential for precision oncology and individualized treatment plans^13^. As technology improves and becomes more affordable, incorporating WGS in standard clinical care is vital for fully realizing the potential of genomic medicine in oncology.

A fundamental question regarding WGS concerns its practical applicability in real-world clinical scenarios. Some believe that the sheer amount of data from WGS might compromise its effectiveness in clinical settings. This research assessed the practical use of a commercially accessible WGS bioinformatics pipeline for cancer patients at Ajou University Hospital in Suwon, South Korea.

## Materials and methods

### Study design and participants

The research protocol was approved by the Institutional Review Board (IRB) of Ajou University Hospital (Suwon, Korea; IRB Code: AJOUIRB-SMP-2022-278) and was performed with adherence to the principles of the Declaration of Helsinki and the stipulations of Good Clinical Practice Guidelines. This prospective, single-center-based genomics trial in the clinical setting (Clinical Research Information Service registration: #KCT0008118) included adult participants with diverse solid cancer types and stages and was conducted at Ajou University Hospital (Suwon, South Korea) from September 2022 to April 2023. Informed consent was obtained from all participants prior to their inclusion. The primary objective of the study was to assess the clinical utility of implementing WGS in a real-world hospital setting. The inclusion criterion was adult patients (age ≥19 years) with histopathologically verified solid cancers who were eligible for biopsy or surgical excision as part of their routine care regimen. Patients were enrolled if medical oncologists wanted to apply WGS to provide (1) actionability for therapeutic options and/or (2) clarity for clinical questions. For the trial, a total of 120 CancerVision^TM^ assays (Inocras, San Diego CA, USA) were obtained from Inocras (San Diego CA, USA). Patients were excluded if they had insufficient tissue samples or declined genetic testing.

### Sequencing and detection of genomic variances

Clinical-grade WGS was performed using the WGS component of the CancerVision^TM^ assay as previously reported^12^. CancerVision^TM^ guarantees a 3-week turn-around time (TAT) through a controlled and standardized pipeline, regardless of the institution from which it is commissioned. Briefly, WGS was performed on tumor samples obtained via surgery or biopsy as part of routine clinical care and stored as fresh frozen tissue. Biopsy sample cores were retrieved first for routine pathology, and at least one additional core was obtained for cancer WGS. If the acquisition of sufficient fresh cancer tissue was not possible, formalin-fixed paraffin-embedded (FFPE) tissues for pathology reviews were alternatively obtained to extract cancer DNA. For the matched normal samples, peripheral blood was used. DNA extraction and library preparation were performed at Inocras, Inc., in a Clinical Laboratory Improvement Amendments (CLIA)-certified laboratory. For DNA extraction, we used the AllPrep DNA/RNA Mini Kit (Qiagen, Hilden Germany) and AllPrep DNA/RNA FFPE Kit (Qiagen, Hilden Germany) for fresh and FFPE samples, respectively. For library preparation, we used TruSeq DNA PCR-Free (Illumina, San Diego, CA) and TruSeq DNA Nano (Illumina, San Diego, CA) library preparation kits for fresh and FFPE samples, respectively. Sequencing was performed on the Illumina NovaSeq 6000 platform (Illumina, San Diego, CA) with an average depth of coverage of 40x for tumors and 20x for blood.

### Genomic analysis and interpretation

Comprehensive genomic analysis and interpretation were conducted using the CancerVision™ platform (Inocras, San Diego, CA, USA). Briefly, BCL files created by the sequencer were converted to fastq files using bcl2fastq software. After obtaining the adapter sequences, the reads in the fastq files were mapped to the human reference genome GRCh38 using a machine learning-augmented burrows-wheeler aligner maximal exact matches algorithm^14^. This was followed by preprocessing, which included marking duplicates and creating analysis-ready compressed reference-oriented alignment map (CRAM) files. CRAM files for tumors and matched normal tissues were analyzed using Mutect2^15^ and Strelka2^16^ to detect somatic single nucleotide variants and small indels, creating variant call format files. To construct a high-confidence somatic variant dataset, we included variants with specific attributes for further analysis: 2 or more variant read counts in tumor samples, most read counts in a normal sample, 15 or more mapping quality score, 5% or lower variant allele frequency, and 1% or lower allele frequency in a panel of normal samples. To identify driver mutations, we applied specific criteria tailored to oncogenes and tumor suppressor genes (TSGs). For oncogenes, variants were considered if they exhibited oncogene-related alterations, such as missense mutations or TERT promoter mutations, and were either frequently reported in the Catalog of Somatic Mutations In Cancer (COSMIC)^17^ or associated with actionable drugs. In the case of TSGs, variants were included if they demonstrated TSG-related alterations, such as frameshift insertions. Additionally, less severe alterations, such as missense mutations, were deemed significant if they were predicted to be deleterious by the SIFT of PolyPhen or if they were reported to be pathogenic in ClinVar^18^. These stringent criteria allowed for the identification of clinically relevant driver mutations.

Tumor characteristics, such as the tumor cell fraction (TCF), tumor cell ploidy, and segmented CNA profiles, were estimated using Sequenza^19^, whereas Delly was employed to identify somatic genomic rearrangements^20^. CNAs with a gene copy number equal to or exceeding the ploidy + 5 threshold were considered to indicate oncogenic amplification. However, if targeted drugs were available, the cutoff was decreased to a ploidy of + 1. On the other hand, a TSG biallelic deletion was identified based on two criteria: a gene copy number less than or equal to 0.5 or a gene copy number less than 1 with a segment total copy number of 0. The SVs selected for further analysis met specific criteria. These included a requirement for the variant read count in tumor samples to be two or more, whereas the variant read count in normal samples needed to be one or less. Additionally, SVs were subjected to a minimum mapping quality threshold of 15, and those with greater than 5% allele frequency in a panel of normal individuals were excluded from the analysis. These stringent criteria ensured the selection of high-quality SVs for subsequent investigation.

The refined somatic variants were annotated using the Ensembl Variant Effect Predictor^21^. Their therapeutic relevance was ascertained through external databases from COSMIC^17^ and OncoKB^22^. Germline polymorphisms were also annotated using the Ensemble Variant Effect Predictor to determine their pathogenicity^21^. All variants, both germline and somatic, were subjected to rigorous manual review and curation within Inocras’s proprietary genome browser, yielding a patient report form for clinical practitioners. (Supplementary Fig. 1)

#### Correction of FFPE artifacts

To remove sequencing artifacts abundant in FFPE tumor specimens^23^, we utilized specialized point mutation and CNA filters, harnessing machine learning methodologies. Briefly, we fashioned models based on features derived from the WGS data. Each sample was trained on FFPE samples and equipped with designated variant labels. For the CNA assessments, we utilized a bespoke Inocras patented FFPE copy number variation (**CNV**) rectification algorithm.

#### Mutational signature analysis

Using the COSMIC database’s cataloged signatures, we assessed the prevalence of mutational signatures for single-base substitutions (SBSs), indels, and SVs in every sample by employing the nonnegative least squares approach based on previously reported algorithms^24^. The selection of signature sets was tailored to each sample based on the cancer type^25^. For instances where the default signature combination did not sufficiently explain samples (cosine similarity <90%), a manual evaluation was undertaken to adjust the signature set by either addition or removal.

#### Analysis of Homologous Recombination Deficiency (HRD) and Microsatellite Instability (MSI)

To assess HRD, we developed our proprietary algorithm by combining HRD-associated features, such as mutational signatures of point mutations and copy number changes^26^. These included single-base substitution signatures (SBS3 and SBS8; reference signatures are available at https://cancer.sanger.ac.uk/signatures/), an indel signature (ID6), genomic rearrangement signatures (RS3 and RS5), deletions accompanied by microhomology, and CNVs. Custom scripts provided scores of the multidimensional features using coefficients derived from published algorithms to compute the final HRD probability scores. Scores equal to or exceeding 0.7 were deemed HRD positive. For a quantitative evaluation of somatic microsatellite alterations, we considered both the MSIsensor score^27^ and the proportion of MSI-related mutational signatures (SBS6, SBS15, SBS20, SBS21, SBS26, SBS30, and SBS44)^25^.

#### Computing resources

We utilized Amazon Web Services to execute the bioinformatic pipeline. A total of 20 r6i.8xlarge instances were utilized, each equipped with 32 virtual CPUs (vCPUs) and 256GB of RAM, facilitating the efficient processing of large datasets and complex computations. Concurrent processing of each sample from a flow cell (30 samples) was conducted through parallel processing, leveraging all vCPUs of each instance. Although the analysis duration varied based on factors such as data size, algorithm complexity, and hardware performance, on average, it took approximately 13 h per sample to complete the analysis.

## Results

### Patients and characteristics

**Fig. 1:**
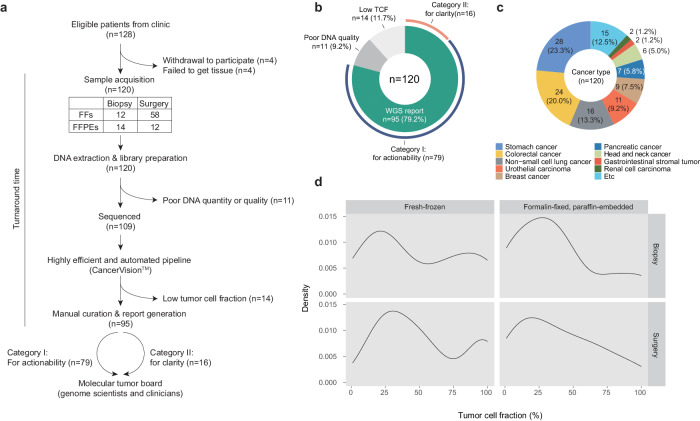
Study design and metrics of the sequencing cohort. **a** Flow diagram of the study protocol. **b** Technical success rate of the CancerVision^TM^ application in the clinical setting. **c** Tumor types enrolled in this study. **d** The distribution of the tumor cell fraction of cancer tissues estimated using WGS according to specimen type.

The study initially enrolled 128 solid tumor patients between September 2022 and April 2023; the patients were referred by medical oncologists for whom two main points needed to be addressed: (1) assessing the actionability of therapeutic options (referred to as the actionability question; Category I) and (2) providing clarity for clinical questions (referred to as the clarity question; Category II). In the present study, 8 patients were excluded, including 4 due to the absence of tumor specimens and 4 due to voluntary withdrawal (Fig. 1a). The full demographics of the 120 patients ultimately enrolled in this study are shown in Supplementary Table 1 (*n* = 120).

Among the 120 patients ultimately enrolled, 11 were not sequenced due to suboptimal DNA quality/volume. Of the 109 patients whose genomes were sequenced, 14 had noninterpretable genome sequences attributed to a low tumor cell fraction (TCF < 15%; Fig. 1a). Consequently, interpretable WGS reports were obtained for 95 patients, reflecting a 79% technical success rate (95/120) in real-world WGS (Fig. 1b).

The objective of this study was to observe the clinical application of WGS in a real-world setting, leading to a heterogeneous composition of our study cohort. Briefly, the entire cohort (*n* = 120) included 23 tumor types (Fig. 1c), consisting of 71 male (59.2%) and 49 female patients (40.8%) with a median age of 60 years (range 19–85 years). At the time of sample acquisition, 99 (82.5%) patients were treatment naïve, 7 (5.8%) had relapsed, and 14 (11.7%) were receiving active treatment. Tumor specimens were obtained from fresh-frozen tissues obtained from surgery (*n* = 58; 48.3%), fresh-frozen tissues obtained from biopsy (*n* = 36; 30.0%), FFPE tissues obtained from surgery (*n* = 12; 10.0%), and FFPE tissues obtained from biopsy (*n* = 14; 11.7%) (Fig. 1a).

For the 109 patients whose genomes were sequenced, the median TCF estimated from the genome sequences was 43.6% on average (range 0.0–100.0%). Contrary to conventional wisdom, TCF was not substantially different between surgical and biopsy specimens (*P* = 0.359 from analysis of variance; Fig. 1d), suggesting widespread applicability of WGS regardless of the sample collection methods.

### Turnaround time in the clinical setting

**Fig. 2:**
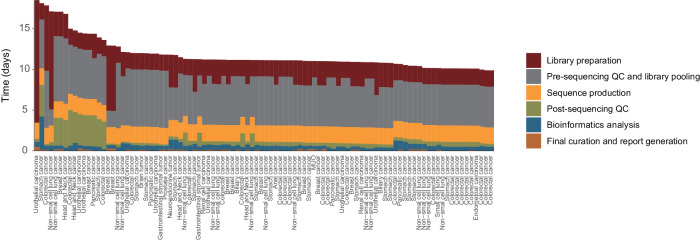
Turnaround time of WGS-based cancer genome profiling in the clinical setting. TAT was measured as working days from sample collection to the acquisition of the WGS report. The processes of the WGS assay were subdivided into six components: (1) library preparation, (2) presequencing quality assessment and library pooling, (3) sequence production, (4) postsequencing process, (5) bioinformatics analysis, and (6) final curation/report generation.

For the 95 samples processed, the median TAT from sample acquisition to reporting was 11 working days (range 9 to 18 days) (Fig. 2). The breakdown of the duration included laboratory experiments (median 2 days, range 2–15 days), presequencing quality control and library pooling (median 5 days, range 1–8 days), sequence production (2 days), postsequencing time (median 1 day, range 1–4 days), bioinformatics analysis (dry pipeline from raw fastq files to filtered mutation calls) for a median of 12.6 h (range 4.7–98 h), and report generation, including final curation from medical genome scientists (~1.5 h). In four cases, the library preparation step was substantially delayed due to the unexpected breakdown of laboratory equipment. Overall, 87.4% of the patients (*n* = 83) received their WGS reports within a two-week period. Considering TATs of 11–18 days for TPS in real-world settings^28,29^, the TATs for WGS in our study were found to be generally satisfactory.

### Mutational detection

**Fig. 3:**
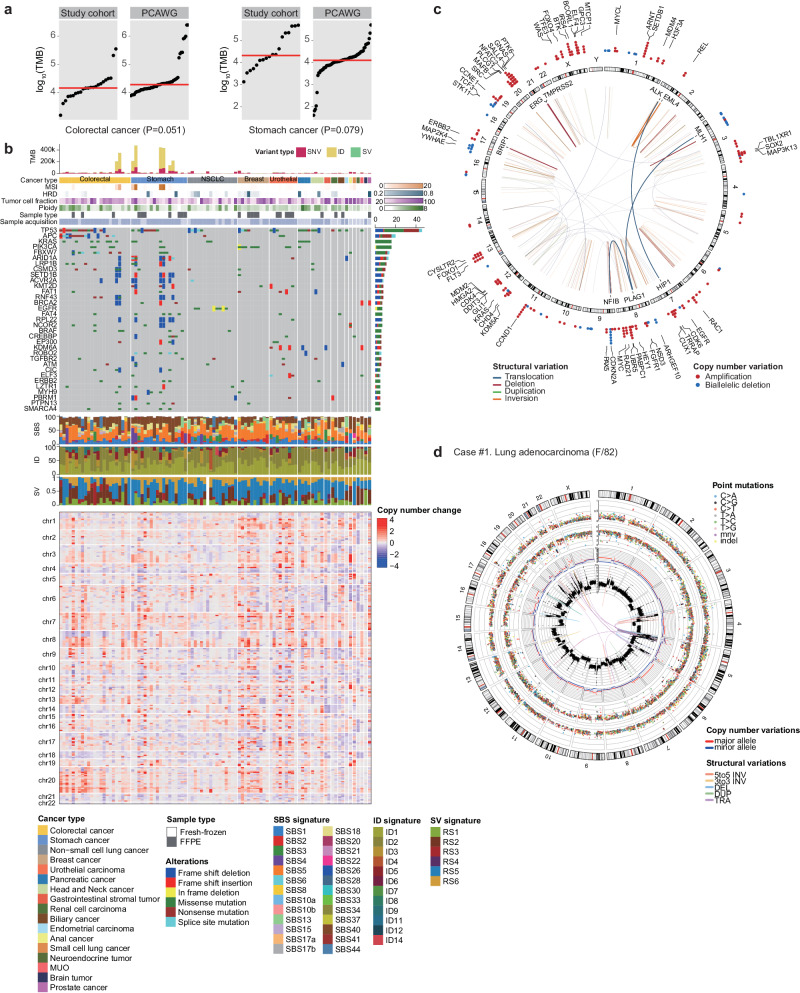
Landscapes of somatically acquired mutations in cancer genomes. **a** Comparison of tumor mutational burden (TMB) between Pan-Cancer Analysis of Whole-Genomes (PCAWG) and this study. Two studies showed similar TMBs in two cancer types. Every dot represents a sample, and the red horizontal lines are the median TMBs in the respective cancer type. **b** Landscape of somatically acquired mutations in 95 solid tumors in this study. Top to bottom: tumor mutational burden (TMB), cancer type, microsatellite instability (MSI) score, homologous recombination deficiency (HRD) score, tumor cell fraction estimated from WGS, genome ploidy, sample type (fresh-frozen or FFPE), sample acquisition method (biopsy or surgery), point mutations in frequently mutated cancer genes, mutational signatures (SBS, ID, and SV), and copy number alterations. **c** Cancer genes frequently altered by SVs (arcs inside the circle) and copy number changes (dots outside the circle). **d** A representative Circos plot summarizing all the somatic mutations (from outer to inner circles; chromosomes, point mutations with variant allele fraction (VAF), point mutations with intermutational distance, genome-wide loss-of-heterozygosity (LOH) pattern, genome-wide copy number changes, and SVs).

Our WGS analysis of 95 tumor/normal matched patient samples revealed a total of 3.54 million somatic mutations, including 1,436,165 SBSs, 2,089,735 indels, and 11,614 SVs. The average local sequencing read depth for these variants was 41.38x (range 23.57–92.01) for tumors and 18.57x (range 11.53–29.54) for matched normal tissues (Supplementary Fig. 2). Overall, the mutational burdens of the SBSs were close to the previously known burdens (Fig. 3a), suggesting that our sequencing analyses were conducted properly. Mutations altering known cancer genes, such as *EGFR* L858R, *KRAS* G12V, and *BRAF* V600E, along with their mutational signatures and genome-wide CNAs, are summarized in Fig. 3b. Collectively, 288 CNA and 261 SV events altered known cancer genes (Fig. 3c). For each sample, a combined mutational landscape was obtained (Fig. 3d), which was cross validated to evaluate the accuracy of the calls. Notably, this combined information is typically challenging to access in other assays. In our cancer cohort, 32 (33.7%) and 75 (62.5%) of the tumors carried whole-genome duplications (ploidy > 3.5) and complex rearrangements (including chromothripsis, chromoplexy and breakage-fusion-bridge cycles), respectively, which were associated with poorer drug responses and survival^30,31^.

In addition to these somatic mutations, we identified 110 rare (<0.5% of the population) pathogenic or likely pathogenic germline variants in cancer predisposition genes (Supplementary Table 2), including the following the American College of Medical Genetics and Genomics secondary genes^32^: *BRCA2* (*n* = 2), *BRCA1* (*n* = 1), *APC* (*n* = 1), *LDLR* (*n* = 1), and *PALB2* (*n* = 2).

To compare WGS with TPS, we employed two approaches. First, we conducted a sister trial involving a retrospective analysis aiming to demonstrate the analytical validity of WGS compared to TPS^12^. The goal of this trial is to illustrate that WGS is capable of capturing the majority of driver mutations detected by TPS. Second, we meticulously examined our findings with a particular focus on mutations that may not be detectable through standard TPS analysis. (Supplementary Fig. 3) Among the eighteen patients in our cohort who underwent TPS, WGS revealed a total of 283 somatic driver mutations, comprising 160 SNVs/indels, 65 CNVs, and 58 SVs. However, only 57 of these mutations (20.1%), including 50 SNVs/indels and 7 CNVs but no SVs, were identified by TPS. Collectively, these findings suggest that WGS offers greater sensitivity in detecting somatic driver mutations, particularly CNVs and/or SVs.

### Actionability of approved therapeutics (I-1)

**Fig. 4:**
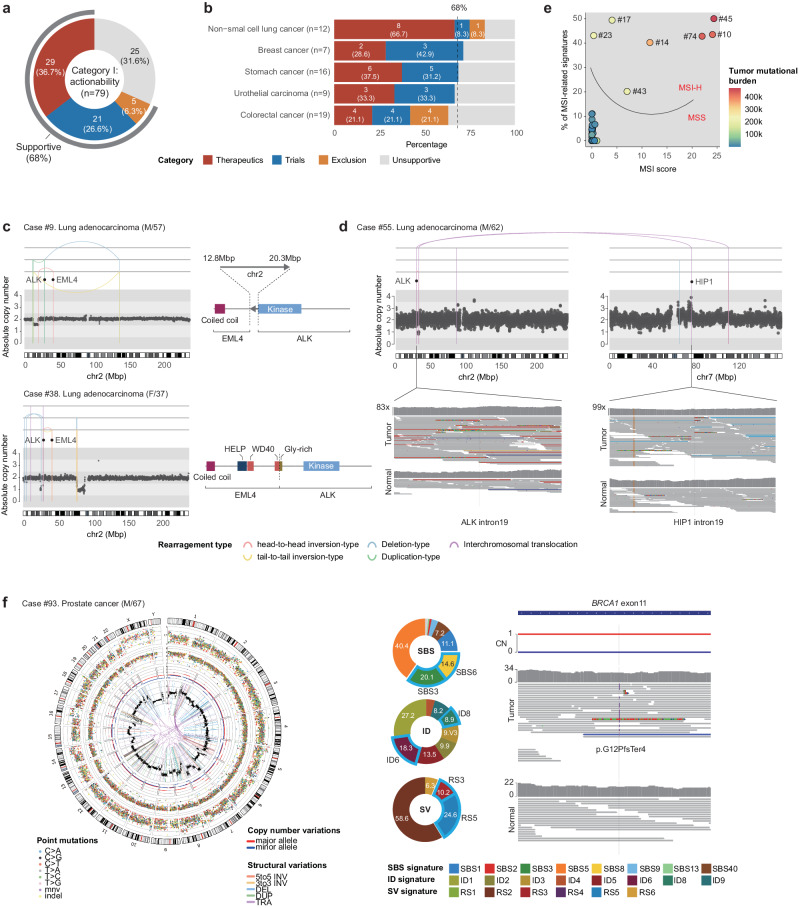
Clinical utility of WGS in the assessment of therapeutic options. **a** Overall, WGS was supportive for therapeutic options in ~70% of the patients. WGS provided information for therapeutics (I-1), clinical trials (I-2), or the exclusion of options (I-3). **b** Clinical utility for assessing therapeutic options across cancer types. **c** Two lung adenocarcinoma patients harboring an oncogenic *EML4*-*ALK* fusion gene via balanced chromothripsis mechanisms. **d** A lung adenocarcinoma patient harboring a rare *HIP1*-*ALK* fusion gene. **e** Seven patients with GI tract cancers showing MSI-H features, a mutational pattern-based target for pembrolizumab treatment. Two independent variables, i.e., the MSI score and MSI-associated mutational signatures, clearly distinguish MSI-H cancer samples. **f** A prostate cancer patient with HRD, a mutational pattern-based target for PARP inhibitors. A Circos plot showing HRD features, proportions of HRD-associated signatures for SBSs, indels, and SVs, and mutations that induced HRD in cancer (somatically acquired BRCA1 frameshift insertion and loss-of-heterozygosity events).

To provide detailed information, we summarized the clinical context and utility of WGS of all 95 patients in Supplementary Table 3. Of the 95 patients in the cohort, 79 were enrolled primarily for assessing therapeutic actionability. Among these, for 55 patients (70%), the findings from WGS supported the clinical decision-making process (Figs. 3b, 4a).

The actionable results from WGS were divided into three distinct subgroups: (1) informing the selection of targeted therapeutics (Category I-1; *n* = 28; 35.4%), (2) facilitating screening for mutation-specific clinical trials (Category I-2; *n* = 22; 27.8%), and (3) aiding in the elimination of ineffective treatment options (Category I-3; *n* = 5; 6.3%) (Fig. 4a). The actionability was variable according to the tumor type. Specifically, non-small cell lung cancer patients were more likely to have Category I-1 actionability (Fig. 4b; *P* = 0.360 according to the exact binomial test), although this difference did not reach statistical significance, likely due to the low number of samples enrolled. In contrast, colorectal cancer patients were less likely to have Category I-1 actionability than other patients (Fig. 4b; *P* = 0.630 using the exact binomial test).

In Category I-1 (*n* = 28; Supplementary Table 1), 15 patients harbored actionable point mutations, such as *EGFR* L858R in lung adenocarcinomas (actionable with erlotinib and others)^33^, *BRAF* V600E in colon cancers (actionable with encorafenib)^34^, *PIK3CA* H1047R/N345K in hormone positive breast cancers (actionable with alpelisib)^35^, and *FGFR3* G370C/S249C/Y737C in urothelial cancers (actionable with erdafitinib)^36^. Often, relatively infrequent but actionable point drivers, such as *PIK3CA* N345K, have been identified in hormone-positive breast cancer (actionable with alpelisib).

Frequently, we identified actionable targets beyond simple point mutations. Three lung adenocarcinomas carry *ALK* fusion genes produced by SVs (all actionable with alectinib, brigatinib or lorlatinib)^37–39^. In two of these patients (patients #9 and #38), balanced chromothripsis, a type of complex rearrangement, resulted in the formation of the *EML4*-*ALK* fusion gene (Fig. 4c)^40^. In the remaining patient (patient #55), translocations between chromosomes 2 and 7 shaped an atypical ALK fusion gene (*HIP1*-*ALK*; Fig. 4d)^41^. Additionally, seven gastrointestinal cancer patients (six stomach cancer patients and one colon cancer patient) exhibited microsatellite instability-high (**MSI-H**) features, indicating that pembrolizumab could be a potential primary therapeutic drug^42^. WGS offered dual insights into MSI-H tumors (both the mutation burden at microsatellite loci and MSI-related mutational signatures), allowing for a clear differentiation of MSI-H tumors from MSS tumors (Fig. 4e).

In Category I-1, two cases displayed actionable features more sensitively detected by WGS than with conventional methods. First, a prostate cancer patient (patient #93) presented a clear HRD phenotype (Fig. 4f; actionable with olaparib, niraparib, or talazoparib)^43–45^. This was characterized by the combination of diverse somatic mutation profiles, including a substantial mutational burden of significant single-base substitutions (SBS3; *n* = 1,452; 20.1% of all 7232 SBSs), indels (ID6; *n* = 142; 18.3% of all 827 indels), and genomic rearrangements (RS3 and RS5; *n* = 107; 34.8% of all 323 SVs). Despite the HRD phenotype, WGS analysis revealed the sporadic nature of the cancer, with two somatically acquired mutations: a frameshift insertion and a combined loss of heterozygosity in the *BRCA*2 gene (Fig. 4f).

### Actionability in clinical trials (I-2)

**Fig. 5:**
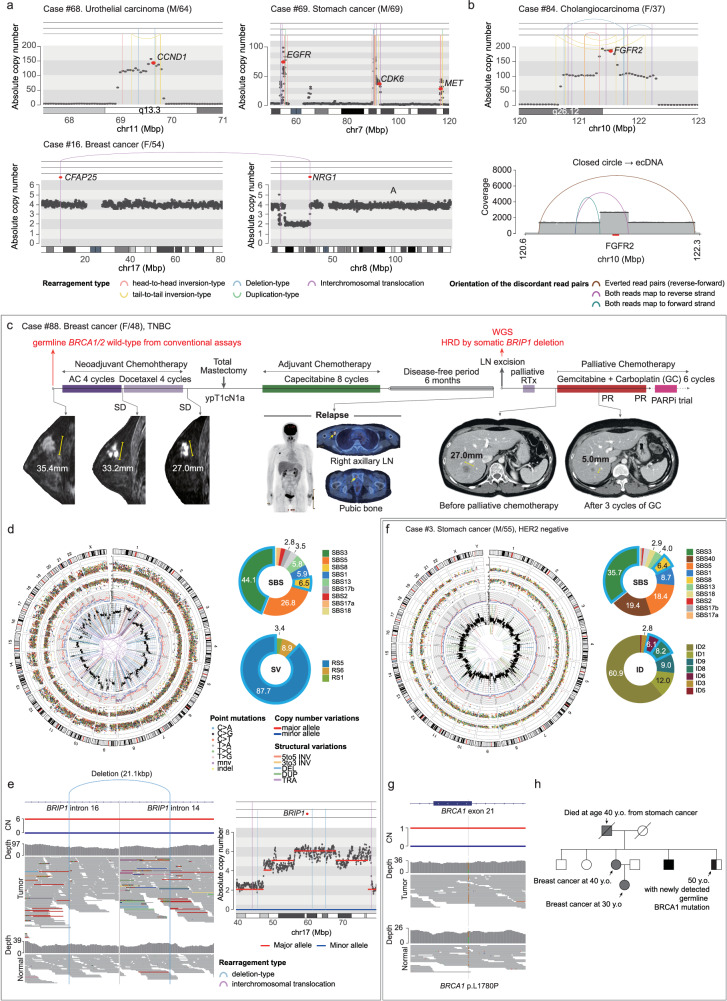
Clinical utility of WGS in the assessment of therapeutic options: available clinical trials (Category I-2). **a** Three cancer cases showing oncogene amplification (*CCND1*, *EGFR*) or *NRG1* rearrangement, representing genomic targets for clinical trials. **b** A patient with cholangiocarcinoma harboring ecDNA-mediated *FGFR2* hyperamplification, a genomic target for a clinical trial. **c** The clinical course of a triple-negative breast cancer patient who was identified as a possible candidate for a clinical trial using WGS. Surgically removed tissue from the right axillary lymph node (LN) was subjected to WGS. WGS identified the strong HRD feature and its underlying genetic cause (complete inactivation of somatically acquired *BRIP1*), which are targets for clinical trials. **d** Circos plot demonstrating the characteristic HRD features of the patient. Outer to inner: ideogram, point mutations (single base substitutions [SBSs] and short indels) and their variant allele frequencies, distances between adjacent point mutations, major (red line) and minor (blue line) allelic copy number (CN), total segmented CN (black dot) and structural variations (SVs). SBS and SV mutational signatures associated with HRD are shown in pie graphs. **e** Integrative genome viewer (IGV) snapshot of a somatic SV disrupting the BRCA1 interacting helicase 1 (*BRIP1*) gene, namely a 21.1-Kbp deletion between *BRIP1* intron 14 and *BRIP1* intron 6. **f** A patient with stomach cancer who was identified as a possible candidate for a clinical trial using WGS. WGS identified the strong HRD feature and its underlying genetic cause (a germline BRCA1 pathogenic variant combined with loss of heterozygosity of the locus in the cancer), which are targets for a clinical trial. A Circos plot showing the HRD features mentioned above and two pie graphs showing HRD-associated mutational signatures. **g** Pathogenic mutations underlying HRD in the patient: we identified germline *BRCA1* mutations with loss of heterozygosity in the tumor sample. **h** Pedigree of the patients. WGS revealed a strong cancer predisposition in the patient’s family. The pathogenic BRCA1 p.L1780P variant was also identified in the younger brother.

As briefly mentioned above, a substantial portion of the cases had the potential for enrollment in clinical trials based on WGS findings (*n* = 22 out of 79; 27.8%; Category I-2; Figs. 3b, 4a; Supplementary Table 3**;** information on possible trials is shown in Supplementary Table 1). Among these, 12 patients had point mutations in key target genes, such as *KRAS* (*n* = 4), *PIK3CA* (*n* = 3), *HRAS* (*n* = 2), and other genes (one each for *BRAF*, *PTEN*, *ERBB2* and *AKT*). Additionally, six patients were potential candidates for clinical trials because of their focal amplification of *CCND1* (*n* = 3) and *MET* (*n* = 1) or the presence of a fusion gene involving *NRG1* (Fig. 5a).

A cholangiocarcinoma patient (patient #84) exhibited focal, extrachromosomal DNA (**ecDNA**)-driven *FGFR2* hyperamplification^46^, involving approximately 185 copies (Fig. 5b). However, FGFR2 fusions or rearrangements are potential targets for treatment with the FGFR-targeting tyrosine kinase inhibitor pemigatinib, and FGFR2 amplifications are known to be less responsive to pemigatinib^47^. An ongoing clinical trial (NCT04526106) is evaluating the efficacy of RLY-4008, a highly selective FGFR2 inhibitor, in patients with solid tumors with FGFR2 amplification. (Supplementary Table 3).

In our cohort, HRD phenotype findings from WGS correlated with potential clinical trial opportunities for three patients. One patient, a 48-year-old female with triple-negative breast cancer, was particularly illustrative of the insight gained from WGS (patient #88; Fig. 5c). Her young age at diagnosis initiated conventional *BRCA1/2* testing, which yielded a negative result. Given the information, she underwent neoadjuvant chemotherapy (Adriamycin, cyclophosphamide, and docetaxel) with a limited response (stable disease from the Response Evaluation Criteria in Solid Tumors v1.1 criteria). She experienced local recurrence in the right axillary lymph nodes and distant metastases in the pubic bone 6 months after mastectomy and adjuvant chemotherapy (8 cycles of capecitabine). Unexpectedly, WGS analysis of a specimen from the axillary lymph nodes revealed a strong HRD signature (HRD score = 0.924; Fig. 5d). Consistent with previous *BRCA1/2* testing, pathogenic germline mutations in the *BRCA1* and *BRCA2* genes were not detected with WGS. However, the *BRIP1* (BRCA1-interacting helicase 1) gene was completely inactivated by two somatically acquired mutations (a 21.1-Kbp deletion between introns 14 and 16 and a combined loss of heterozygosity of the *BRIP1* gene; Fig. 5e). This finding likely contributed to the observed HRD phenotype in her cancer^48^. Informed by the WGS data, the patient was prescribed a platinum-based treatment regimen because of the connection between homologous recombination pathway deficiencies and therapeutic efficacy^49^. After receiving gemcitabine plus carboplatin for three cycles, she exhibited a partial response, which continued through six cycles (Fig. 5c). Presently, her suitability for a talazoparib (PARPi) maintenance therapy as part of a clinical trial (NCT04755868) is being considered, a decision based on WGS findings.

The remaining two patients (patients #3 and #76) had stomach cancers, the HRD phenotypes of which were recognized by the strong HRD signatures in their somatic mutation profiles (0.718 and 0.840, respectively; patient #3 is represented in Fig. 5f). These strong signatures suggested a familial origin for these cancers. Subsequent analysis for germline variants revealed causative mutations: a germline pathogenic variant in *BRCA1* p.L1780P for patient #3, and in *BRCA2* p.C717* for patient #76. Additionally, the secondary hits at these loci were observed in their matched cancer tissues (a large deletion for patient #3; a somatically acquired stop-gain base substitution for patient #76), confirming the causative role of the inherited mutations in the development of the tumors (Fig. 5g). These findings indicated the potential for enrollment in a PARP inhibitor clinical trial for any solid tumor with deleterious mutations in HRD-associated genes (NCT04171700). Importantly, the WGS results were the first to reveal the familial cancer history of these patients. Notably, WGS allowed the patients to first recognize their familial cancer history (Fig. 5h) and prompted their family members to undergo genetic testing. We further detected a pathogenic germline mutation in the younger brother of patient #3.

### Actionability by exclusion of nonbeneficial therapeutic strategies (I-3)

In five patients, WGS assisted in clinical decision-making by predicting potentially ineffective therapeutic options (Category I-3). These included four colorectal cancer patients (two with *KRAS* G12V, one with *KRAS* Q61L, one with *NRAS* G61K; patients #18, #28, #79 and #21; Supplementary Table 1) and one non-small cell lung cancer patient with no targetable mutations (patient #29). Although RAS oncogenic mutations were the earliest drivers discovered in human cancers, they have not been druggable for ~40 years^50^. Recently, a few *KRAS* G12C-selective inhibitors, such as sotorasib and adagrasib, were approved for the treatment of solid tumors^51,52^. However, *KRAS* mutations other than G12C are still intractable for other targeted agents. These genomic mutations have led clinicians to avoid the use of potential targeted therapies such as cetuximab in colorectal cancers^53^ and erlotinib/crizotinib in lung adenocarcinoma^54^.

### Clarity for clinical questions (II)

**Fig. 6:**
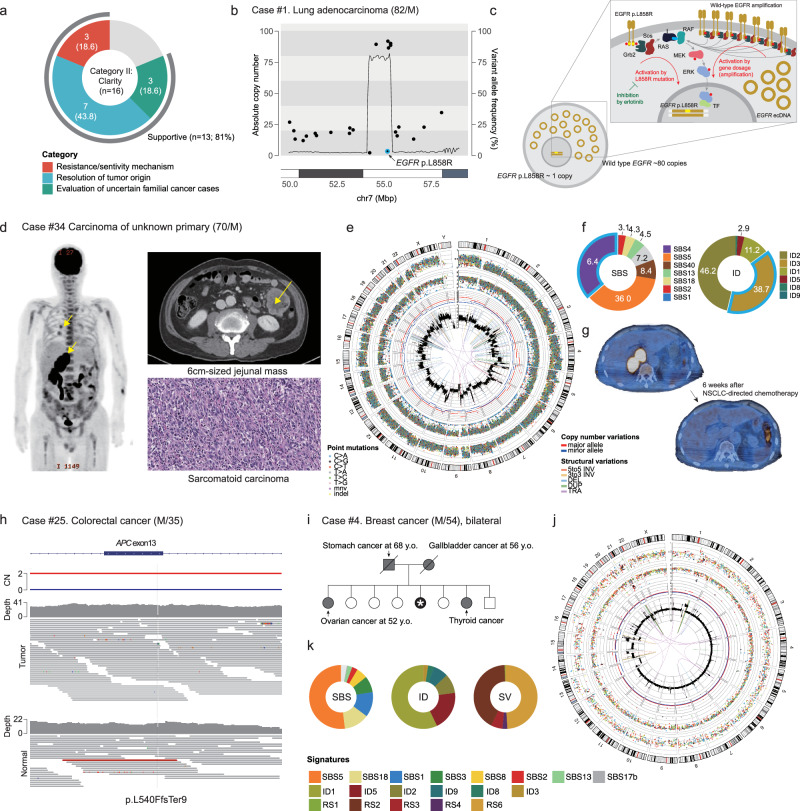
Clinical utility of WGS for providing clarity to clinical questions (Category II). **a** WGS was supportive in ~81% of the cases conducted for clinical clarity. WGS provided information on the resistance/sensitivity mechanism (II-1), tumor origin (II-2), and evaluation of familial cancer patients (II-3). **b** A lung adenocarcinoma patient evaluated by WGS to determine the mechanism of resistance to erlotinib treatment. Hyperamplification of the *EGFR* wild-type allele was found. **c** In cancer, the MAPK pathway is activated by two mechanisms: (1) EGFR L858R and (2) EGFR-wt amplification. The latter cannot be inhibited by erlotinib. **d** 2-Deoxy-2-[fluorine-18]fluoro-D-glucose (18F-FDG) positron-emission tomography-computed tomography (PET-CT) image at the initial presentation of a patient with cancer at an unknown primary site showing a 6.4-cm jejunal mass. A hypermetabolic mass was located in the right adrenal gland and identified as a potential peritoneal carcinomatosis. Additionally, we noted multiple small nodules in both lungs. A computed tomography scan and hematoxylin and eosin image of the right adrenal gland mass are also shown. **e** Circos plot showing genomic findings from WGS. **f** Mutational signature analysis of SBSs and IDs. High proportions of SBS4 and ID3 mutational signatures (attributed to tobacco smoking) were noted. **g** Treatment response to non-small cell lung cancer (NSCLC)-directed chemotherapy: nivolumab plus ipilimumab in combination with paclitaxel plus carboplatin. **h** A patient with colorectal cancer with a germline APC truncating mutation, suggesting that the cancer was a familial case (familial adenomatous polyposis). **i** A patient with bilateral breast cancer suspected to have familial cancer given her clinical features and familial history. **j**, **k** According to the cancer genome profiling, HRD-associated features were absent, suggesting that bilateral breast cancer was likely sporadic. In line with these observations, no pathogenic germline mutations were found.

Of the 95 patients in the cohort, 16 were enrolled, not primarily for understanding therapeutic actionability but for obtaining insights into the questions that are associated with clinical decision-making (Fig. 1a; Supplementary Table 1). These 16 patients were further categorized into three groups: (1) identification of drug resistance and/or responsive mechanisms (*n* = 6; Category II-1), (2) resolution of tumor origin (*n* = 7; Category II-2), and (3) evaluation of uncertain familial cancer cases (*n* = 3; Category II-3). WGS successfully addressed these clinical questions in 81% (13/16) of the patients in the clarity category (Fig. 6a).

The six patients in the II-1 subcategory included patients who were resistant (*n* = 5) or hyperresponsive (*n* = 1) to targeted therapeutics (Supplementary Tables 1, 3). Posttreatment cancer tissues were analyzed using WGS to understand the molecular basis of these unusual treatment sensitivities, and conclusive insights were obtained in half of the patients (3/6). For example, in an *EGFR* L858R lung adenocarcinoma patient (patient #1), erlotinib was not effective. WGS of the posttreatment tissue revealed that the ecDNA-driven hyperamplification of EGFR reached more than 80 copies, which likely amplified the wild-type *EGFR* allele (Fig. 6b), suggesting dual independent activating mechanisms of the MAPK pathway in this tumor (Fig. 6c). This finding directly indicated that erlotinib inhibition, which exclusively targets the *EGFR* L858R mutation, was not sufficient.

For the other seven patients, a pathological review was insufficient to reveal the origin of the tumors; therefore, the selection of optimal treatments was challenging (Category II-2). Intriguingly, WGS was helpful for all the patients (7 out of 7; 100%). For example, patient #34 had a 6.4-cm jejunal mass, which was initially diagnosed as sarcomatoid carcinoma of unknown origin based on pathologic evaluation (Fig. 6d). Positron emission tomography revealed three metabolically active masses, including two lung nodules and one large adrenal mass. The application of WGS to jejunal tumor revealed a heavy mutational load attributable to tobacco smoking (i.e., over 43,509 somatic mutations; 36.4% of base substitutions occurred in the SBS4 mutational signature; 38.7% of indels occurred in the ID3 mutational signature; Fig. 6e, f). These data strongly suggest direct exposure of the cancer cell lineage to tobacco smoke^55^; thus, the most likely primary origin is the lung rather than the adrenal gland or jejunum per se. Given this information, the patient was diagnosed with non-small cell lung cancer with sarcomatoid differentiation that metastasized to other organs and was subsequently treated with the Checkmate-9LA regimen, which included nivolumab, ipilimumab, paclitaxel, and carboplatin^56^. The patient demonstrated remarkable and sustained responses (Fig. 6g).

Three patients benefited from WGS to evaluate their familial features (Category II-3). A colorectal cancer patient (patient #25) had first-degree relatives who had a history of colorectal cancer (father) and total colectomy (brother). WGS revealed a germline frameshift indel in the *APC* TSG that was combined with loss of heterozygosity in cancer cells, leading to the diagnosis of autosomal dominant familial adenomatous polyposis in the patient (Fig. 6h)^57^. Interestingly, WGS yielded the opposite results in a similar scenario. One patient with breast cancer (patient #4) had bilateral breast masses that metastasized to the lung and pleura. Her sister died from ovarian cancer at a young age (57 years old), and her parents had a history of stomach cancer and cholangiocarcinoma (Fig. 6i). Clinically, the patient was strongly suspected to have familial breast cancer due to *BRCA1* or *BRCA2* pathogenic variants. However, no germline pathogenic variants were detected using WGS. In addition, the mutational patterns suggested that her breast cancer was a typical luminal type (Fig. 6j), and HRD-associated mutational signatures were completely absent (HRD = 0; Fig. 6k), confirming that the cancer was sporadic.

## Discussion

In this prospective hospital‐based cohort study, we evaluated the clinical utility of WGS in precision oncology for solid tumors. A challenge in the clinical application of WGS is achieving standardized processing and the medically relevant interpretation of extensive sequencing data within the constrained timeframes necessary for clinical decision-making^58^. This study utilized the commercially available CancerVision^TM^, a CLIA- and CAP-certified clinical assay. This assay incorporates a suite of automated, comprehensive bioinformatics pipelines that explore various dimensions of cancer genomes, including somatic point mutations, CNAs, SVs, and germline pathogenic alterations, and provides analyses of mutational burden and signatures. This approach allowed clinicians to implement a versatile, single-test solution in the clinical environment.

Cancer is a genomic disease shaped by the intricate interplay of numerous genomic alterations rather than a single cancer gene mutation. Consequently, the molecular characteristics of each cancer are different, manifesting through diverse indicators such as a combination of key driver mutations, mutational signatures (such as HRD and MSI), CNA profiles, and TMB. Assessing these characteristics collectively can aid in pinpointing the distinct molecular vulnerabilities of each cancer patient.

As demonstrated in this work, the assay spans a wide range of clinical questions, primarily informing on two main areas, i.e., seeking actionable genomic biomarkers (“actionability”) and providing clarity for clinical questions (“clarity”). With this comprehensive assay, patients can obtain a holistic view of their cancer. This includes identifying potential targeted treatment options, alignment with ongoing clinical trials, understanding of germline predispositions to cancer, and molecular insights that can be relevant for the potential of therapeutic resistance and pinpointing the origin of cancer. WGS has broader applications than what were demonstrated in this study, including evaluating precancerous lesions such as polyps and monitoring residual disease using tumor genomic data^59,60^.

The definition of “clinical actionability” varies across studies and is often confined to direct therapeutic implications. For instance, the REALM study (enriched with lung cancer cases) showed ~40% clinical actionability of the TPS in on-label drug choices^61^. Defining actionability in terms of therapeutic impact can be biased, as different cancer types have varying numbers of available treatments. For example, cancers such as lung adenocarcinomas with more treatment options might show greater perceived utility in genomic tests. Despite the heterogeneity of our real-world cohort, our study echoes previous observations, reporting an actionability rate of 37% based on the same criteria. However, such a constrained view of actionability may not capture the full spectrum of individual tumor complexities. In our endeavor to provide a broader perspective on actionability, we evaluated the benefits of WGS for its comprehensive analysis of both somatic and germline alterations. In this cohort, we observed that WGS of tumor/normal samples provided clinically valuable insights in ~70% of patients (67/95). It is plausible that including additional aspects of utility, which are not covered in this study, could further enhance this actionability rate.

The cases were primarily determined by medical oncologists at a specific hospital, and the sample size was not extensive. Thus, our cohort may exhibit biases and cannot be readily generalized to all cancer patients. Nonetheless, the clinical application of WGS remains at a nascent stage. Preliminary positive outcomes from smaller scale studies are imperative for the initiation of larger clinical trials. Despite the relatively modest scale of our study, it is one of the largest WGS-associated clinical trials conducted to date. The insights gained from this study could provide valuable groundwork for future research and larger scale trials. Furthermore, we cautiously propose that the observed bias in our study may not invariably yield positive results (i.e., more actionable findings compared to the background) due to the potential enrichment of complex and refractory cases within our cohort, which are less amenable to resolution using conventional techniques.

An indirect but important benefit of the clinical application of WGS is the opportunities it creates for population-scale research from the clinical side. Globally, approximately 20 million new cancer cases are reported annually. Over the last decade, academia-driven cancer genome studies have explored at most 100,000 cancer whole genomes worldwide, most of which lack a detailed medical history. These efforts have primarily identified frequent driver mutations from early-stage surgical tumor specimens, but the clinical impact of many of these mutations remains unclear. The highly individualized nature of cancer, arising from diverse and heterogeneous genomic mutations, suggests that many less frequent driver mutations may go undetected^62^. In particular, mutations associated with later stages of cancer—such as recurrence, metastasis, and drug resistance—are likely more heterogeneous and rarer than those involved in initial tumorigenesis. To effectively capture and clinically annotate these mutations, systematic and comprehensive datasets are crucial. These datasets should encompass genome-wide profiling using WGS and complete clinical histories, covering a wide patient population (clinic-driven research). Genomic data from different hospitals need to be integrated without platform differences. This will be possible when WGS is widely adopted for routine clinical procedures.

Until recently, there have been significant barriers to integrating comprehensive tumor genome analysis into routine practice, including the high cost of sequencing and the complexity of managing large datasets. As a result, concessions were often made. Genetic analyses were largely limited to exonic regions, with whole exome sequencing covering only approximately 1–2% of the entire genome. In some cases, the focus was narrowed even further to TPS of the exonic regions of pertinent genes, representing a scant 0.3% of the genome. However, economic studies indicate that using TPS testing can yield cost savings for both Centers for Medicare and Medicaid Services (CMS) and commercial payers compared with standard limited gene testing^63–65^. The TPS approach can necessitate multiple tests, for example, for somatic, germline, and HRD analyses. This requirement can lead to additional costs being incurred. The WGS approach has emerged as a more financially prudent option for in-depth genomic profiling as the era of the $100 genome is near^66^. By deriving insights into germline, somatic, genomic instability, and mutation signatures from a single test, WGS not only ensures cost savings but also reduces diagnostic burden.

In conclusion, the CancerVision WGS test employed in this study demonstrated its ease of use and clinical utility for solid tumor patients.

## Supplementary information

Supplementary information
