# Supplementary material for: Clinical application of whole-genome sequencing of solid tumors for precision oncology
Source: Exp Mol Med. 2024 Aug 13;56(8):1856–68. doi: 10.1038/s12276-024-01288-x (PMC11371929; doi:10.1038/s12276-024-01288-x)
Supplement: Supplementary file 1 — Supplementary information [file 12276_2024_1288_MOESM1_ESM.pdf]

Supplementary Information

Supplementary Fig. 1. A standardized patient report of CancerVision™.

Supplementary Fig. 2. Local sequencing read-depth of tumor and normal samples.

Supplementary Fig. 3. Comparison between whole genome sequencing (**WGS**) and targeted panel sequencing (**TPS**) in eight patients who underwent both tests in terms of single nucleotide variants and indels (**SNV/Indels**), copy number variations (**CNVs**), and structural variations (**SVs**).

Supplementary Table 1. Clinicopathological characteristics and Categories for Clinical Utility of WGS

Supplementary Table 2. List of pathogenic or likely-pathogenic germline variants

Supplementary Table 3. Library of clinical history and genomic portrait of all patients

Supplementary Fig. 1. A standardized patient report of CancerVisionTM.

CANCERVISION

Patient ID		Original Name			
Age at Registration		Specimen Type	Other	Tumor Collection Time	2022-09-19 00:00:00
Sex at Birth		Ordering Provider		Normal Collection Time	
Accession #		Ordering Client		Registration Date	2022-09-28 19:00:00
Diagnosis	[8140/3] Adenocarcinoma, NOS				

Report summary

Tumor mutation count 6,966 Driver copy number alterations 3 Driver structural variations 6

1. Key Biomarker Findings

**The prescribing information for the FDA-approved therapeutic option may not include the associated key biomarkers and it should be noted this information does not pertain to pediatric indications.*

Key biomarkers	Origin	FDA-approved drugs for patient's cancer	FDA-approved drugs for another cancer
TP53 E343*	Somatic	None	Acalabrutinib + Obinutuzumab, Ibrutinib, Duvelisib, etc.
BRCA1 NP_009225.1:p.Leu1780Pro (germline)	Germline	None	Olaparib + Talazoparib

2. Germline Mutation Summary

**Please refer to next pages for more detailed descriptions.*

One pathogenic and no likely pathogenic germline variant detected.

3. Genomic Instability

**Please refer to next pages for additional descriptions and reference ranges.*

	Tumor mutation burden	Microsatellite status	Homologous recombination deficiency
Result	Low	Stable	Deficient
Score	2.43 mut/Mb	2.54	0.93

4. Key signatures

Results are signatures with >10% contributions in SBS mutational signature analysis other than clock-like, unknown signatures. Please refer to next pages for more detailed descriptions.

Exogenous signature: ☐ UV light exposure ☐ Tobacco smoking signature ☐ Other
*e.g. Aflatoxin exposure

Endogenous etiology signature: ☑ HRD signature ☐ Defective DNA mismatch repair ☐ APOBEC signature ☑ Other
*e.g. polymerase eta somatic hypermutation

Actionable Findings

Genomic alteration	Treatment	Disease	Evidence level
Not found			

Level of Evidence Scoring: The level calls aligns with the AMP/ASCO/CAP consensus, ranging from A (FDA-approved biomarkers) to D (emerging diagnostic and therapeutic relevance)[1]

Somatic Driver Alterations

Single Nucleotide Variations and Small Indels

Mode	Gene symbol	Variant	Consequence	Variant allele frequency	Loss of heterozygosity
Tumor suppressor gene	TP53	E343*	Stop gained	0.138	Present
Tumor suppressor gene	MYH9	E1131Gfs*31	Frameshift variant	0.06	Absent

Copy Number Alterations

Mode	Gene symbol	Consequence	Copy number
Oncogene	QKI	Amplification	9
Oncogene	AFDN	Amplification	18
Oncogene	KRAS	Amplification	8

Structural Variations

Mode	Gene symbol	Consequence	Type	Loss of heterozygosity
Tumor suppressor gene	MAP3K13	Disruption	.	Absent
Tumor suppressor gene	FOXO3	Disruption	.	Absent
Tumor suppressor gene	POT1,NF1	Disruption	.	Absent
Tumor suppressor gene	NDRG1	Disruption	.	Absent
Tumor suppressor gene	PTEN	Disruption	.	Absent
Tumor suppressor gene	RUNX1	Disruption	.	Absent

Germline Cancer-related Variants

Pathogenicity	Mode	Gene	Variant type	Variant	Genotype	Loss of heterozygosity
Pathogenic	_	BRCA1	PM (SNV)	NP_009225.1:p.Leu1780Pro	Het	Present

Tumor Mutational Burden

(Score: 2.43 mut/Mb)

SNVs		Indels	
Count	5,705	Count	1,261
Density	1.99 mut/Mb	Density	0.44 mut/Mb

**The Tumor Mutational Burden (TMB) score represents the number of mutations per Mb across the whole genome of the tumor. A tumor is considered to have a high TMB if the score is > 10mut/mb.*

Mutational Signature

Single Base Substitutions

Signature	Estimated count	Contribution (%)	Proposed etiology
SBS3	2560.6	45.4	Defective homologous recombination DNA damage repair
SBS5	749.36	13.3	Unknown (clock-like signature)
SBS40	745.14	13.2	Unknown

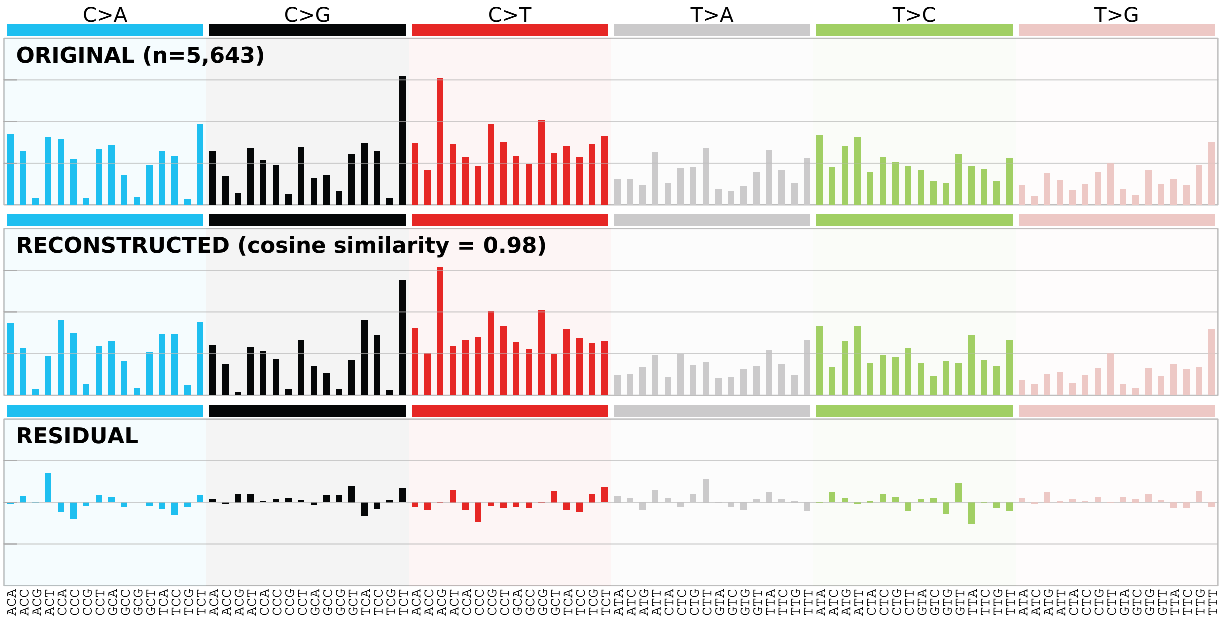

Mutational signature analysis categorizes the found somatic substitutions into 96 mutational contexts, creates a histogram, and then finds the known signature combination that best describes the relevant histogram. Combination of 6 possible types of substitutions and 4 types of nucleotides, that can be present in front of or following the mutational location, produces 96 possible cases (6x4x4).

Indels

Signature	Estimated count	Contribution (%)	Proposed etiology
ID2	496.92	61.6	Slippage during DNA replication of the replicated DNA strand

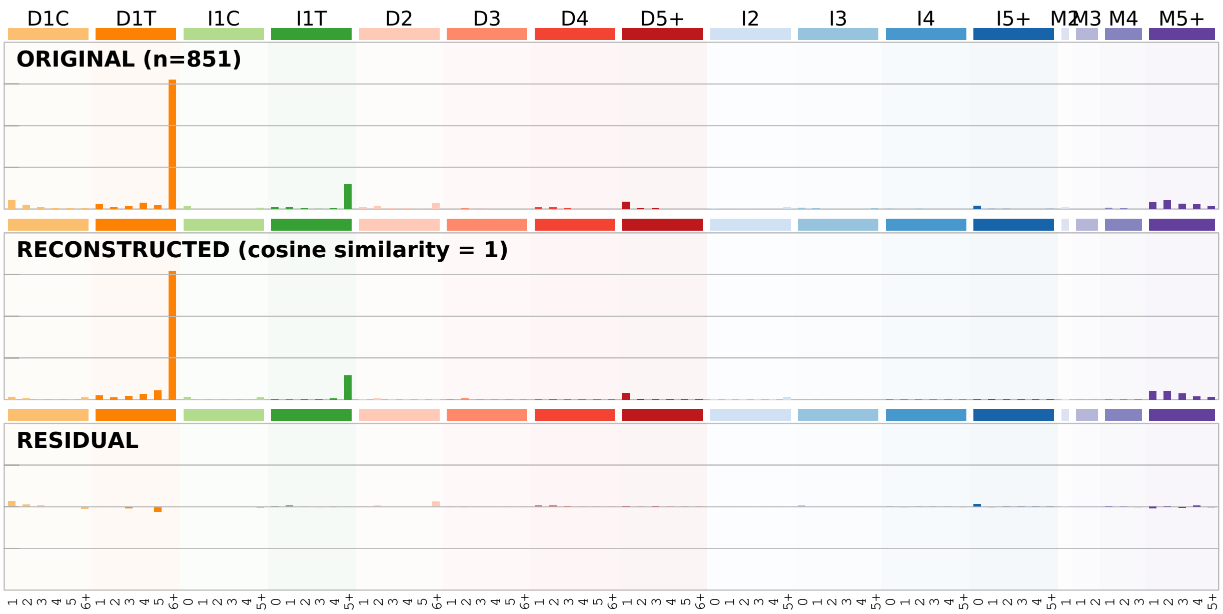

Supplementary Figure 1.

Structural Variations

Signature	Estimated count	Contribution (%)	Proposed etiology
RS3	151.26	64.0	Unknown
RS5	42.56	18.0	Unknown
RS2	37.5	15.8	Unknown

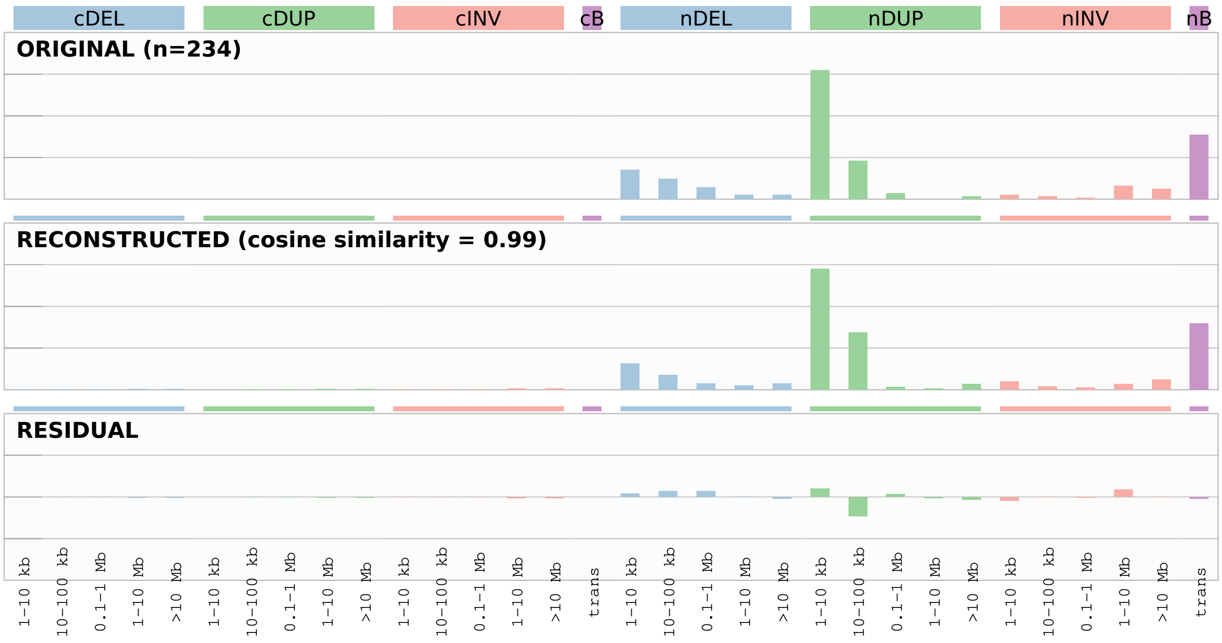

Double Base Substitutions

Signature	Estimated count	Contribution (%)	Proposed etiology
DBS7	8.52	36.4	Unknown
DBS2	8.28	35.4	Unknown
DBS4	6.57	28.1	Unknown

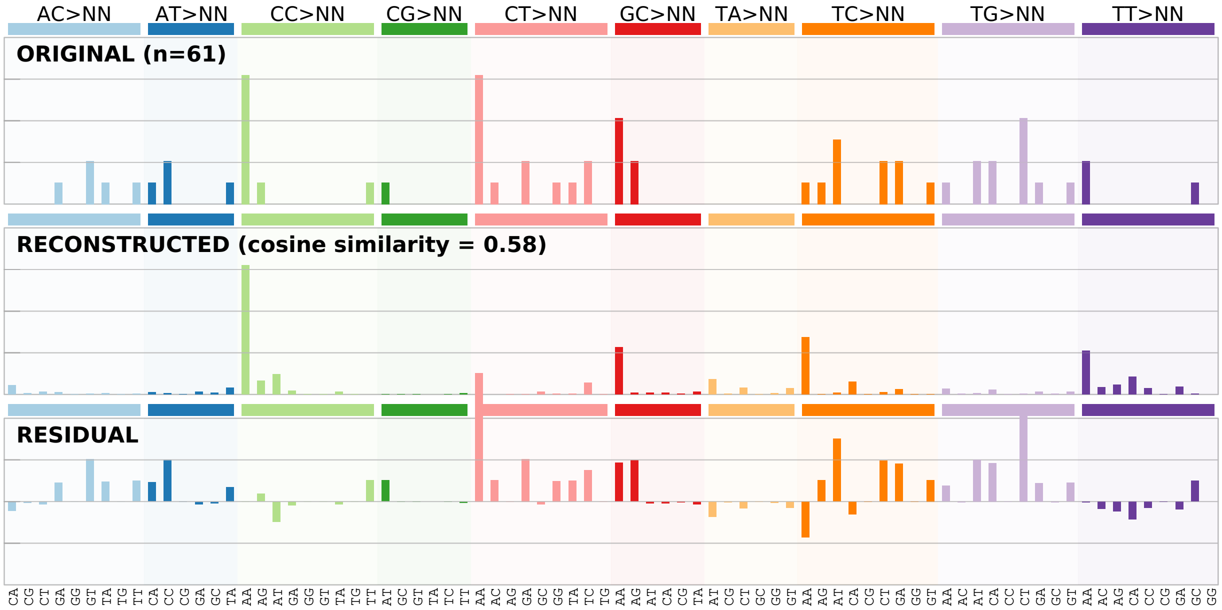

Microsatellite Instability

Stable (Score : 2.54)

Total number of site	2321
Number of somatic site	59
MSI score	2.54

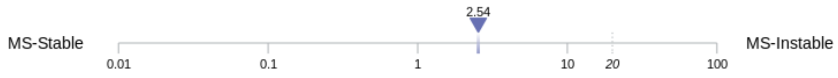

The microsatellite instability (MSI) score represents the number of somatic insertions and deletions per Mb in microsatellite regions across whole genome of the tumor. A tumor is considered microsatellite stable (MSS) if the score is < 20, and MSI-High if > 20.

Homologous Recombination Defect (HRD)

Deficient (Score : 0.93)

SBS3	2,745.0
SBS8	777.0
ID6	8.5%
RS3	151.0
RS5	43.0
LOH/TAI/LST	50

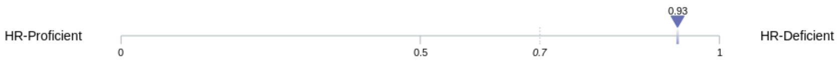

The HRD score is determined by an in-house WGS signature-based classifier comparing the signature of this sample with signatures found across samples with known BRCA1/BRCA2 inactivation. Tumors with a score greater than or equal to 0.7 are considered HR-deficient(Positive), while those with a score below this threshold are considered HR-proficient(Negative).
LOH; Loss Of Heterozygosity, TAI; Telomeric Allelic Imbalance, LST; Large-scale State Transition

Genome Portrait

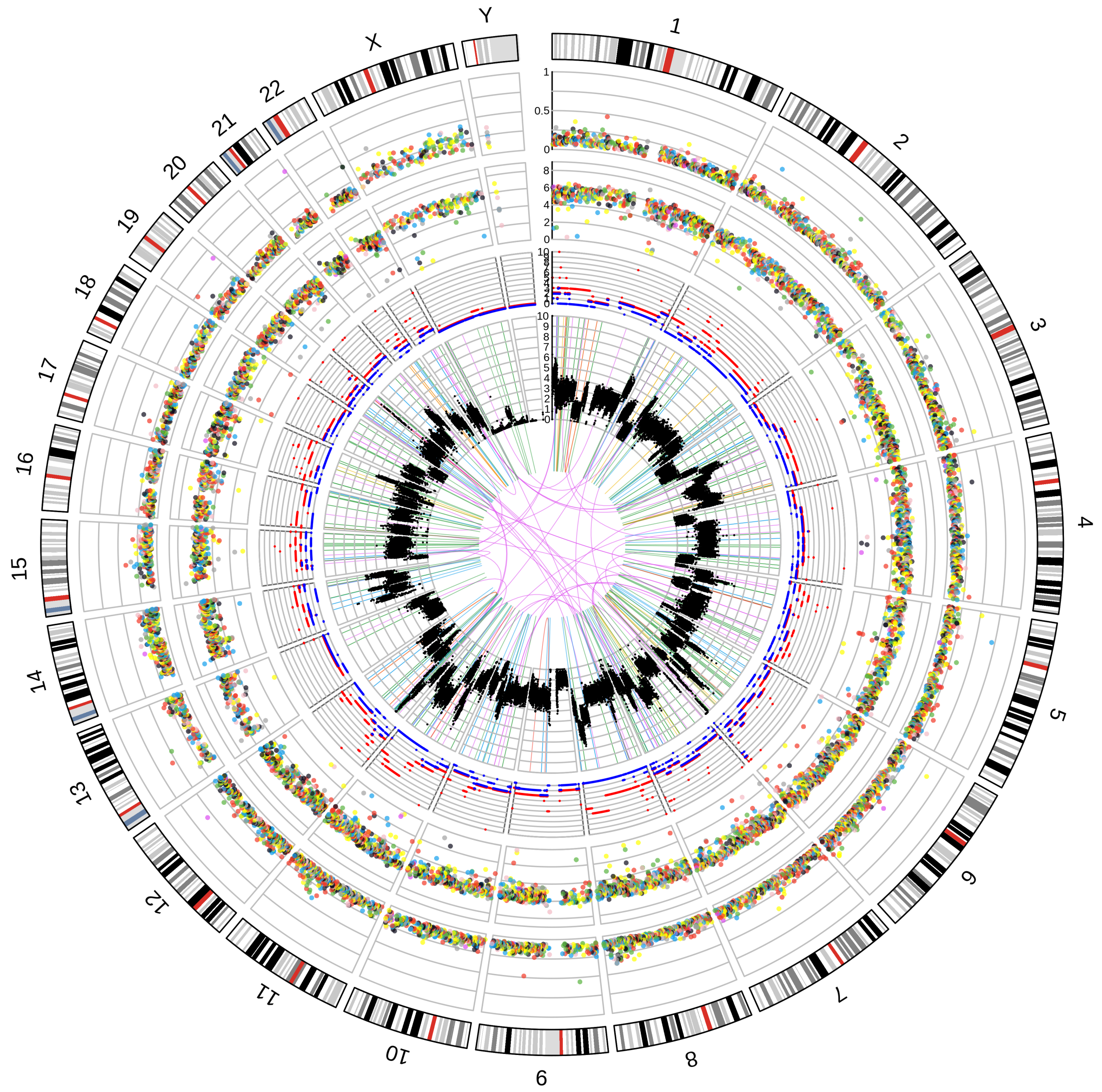

Point mutations
(n = 6,965)
- C>A (996)
- C>G (962)
- C>T (1,391)
- T>A (733)
- T>C (957)
- T>G (604)
- MNV (61)
- Indel (1,261)

Copy number variations
- Major allele
- Minor allele

Structural variations
(n = 234)
- Deletion (48)
- Duplication (120)
- 5to5 inversion (11)
- 3to3 inversion (11)
- Translocation (44)

Information in the CIRCOS plot are: cytoband, variant allele fraction of point mutations, intermutational distance of point mutations (logarithmic scale), copy number of each allele (red lines: major, blue lines: minor) total copy number (black dots), and structural variations (color lines), from the outside of the circle to the inside, in their respective orders.

Test Information

The CancerVision test is a Next Generation Sequencing tumor/normal whole genome assay that provides a list of detected single nucleotide variants (SNVs), multiple nucleotide variants (MNVs), small insertions and deletions (indels), copy number alterations (CNAs), and structural variations (SVs) in tumor tissue, along with an analysis report of mutational signature, tumor mutational burden (TMB), microsatellite instability (MSI), and homologous recombination deficiency (HRD). The genomic DNA is extracted from the patient's normal and tumor samples via the ThermoFisher KingFisher Apex and prepared using the Watchmaker Genomics enzymatic library preparation which includes end repair, purification, adapter ligation and PCR amplification. A portion of the library is hybridized to targeted probes. The libraries are sequenced using the Illumina NovaSeq X+. The sequence data are analyzed using various validated bioinformatics tools and a custom data-processing pipeline for Next Generation Sequencing platforms. GRCh38 is used for human reference genome. Each tumor's cancer-specific mutations are then queried against a proprietary gene-drug database based on peer-reviewed literature to identify potential therapeutic associations; however, this information should be considered in conjunction with other clinical and diagnostic findings.

- Whole genome sequencing: mean target coverage is 40x (unique reads) for tumor sample DNA, and 20x (unique reads) for a normal sample.
- Variant calling was performed using publicly and commercially available tools. Then, variant candidates were examined and refined by pipelines developed by Inocras. The pipelines have been established by a team of experts in genomics and engineering and powered by AI algorithms.
- For formalin-fixed paraffin-embedded (FFPE) specimens, AI-powered data correction algorithms developed in Inocras are used to mitigate data-quality issues commonly encountered in FFPE-derived samples.
- SNVs and indels whose consequences are predicted to activate oncogenes and reported as hotspot mutations by COSMIC were categorized as oncogenic mutations. Those whose consequences cause loss of function of tumor suppressor genes (TSGs) were classified as TSG-disrupting mutations.
- CNAs that amplify oncogenes more than five copies above the average ploidy were classified as oncogene amplification, whereas those that delete both copies of TSGs were categorized as biallelic deletion of TSGs.
- SVs that generate known fusion oncogenes or disrupt TSGs were classified as driver events.
- SVs that produce known fusion oncogenes by connecting two independent genes were classified as fusion oncogene-generating SVs. SVs that alter the arrangement of exons of TSGs were classified as TSG-disrupting SVs.
- Actionable findings list on-label drugs applicable for the patient's disease.
- Tumor cellularity is a proportion of tumor cells in the specimen of whole-genome sequencing. Somatic mutations may be under-detected when tumor cellularity is low.
- Variant allele frequency (VAF) is the fraction of variant-supporting reads among total sequencing reads and is dependent on tumor cellularities.
- Cancer cell fraction (CCF) of a mutation is the fraction of cancer cells harboring the mutation. CCF less than 1 indicates subclonal mutation.
- Tumor mutation burden (TMB) is the number of somatic SNVs, indels, and SVs divided by the effective genome size (~2.9 Gb).
- The mutational signature analysis is primarily based on the COSMIC mutational signatures of SNVs, double-base substitution (DSB), and indels. SV signatures were adopted from the previous studies[2].
- Microsatellite instability (MSI) score is calculated by genome-widely examining microsatellite regions.
- Homologous recombination deficiency (HRD) is evaluated through an in-house script.
- Measure of Genomic Instability, an unweighted sum of loss of heterozygosity (LOH, the total number of LOH regions across the entire genome that are larger than 15Mb but do not involve whole chromosome), telomeric allelic imbalance (TAI, the number of regions with allelic imbalance extending to the subtelomere but not crossing the centromere), and large-scale state transition (LST, the number of break points occurring between adjacent regions of at least 10 Mb). A value greater than or equal to 42 is regarded as having genomic instability.
- The OncoKB™ precision oncology knowledge base was made available under license from Memorial Sloan Kettering Cancer Center.
- The Catalogue of Somatic Mutations in Cancer (COSMIC, cancer.sanger.ac.uk) was made available under COSMIC Static Reporting Startup License from Qiagen[3].
- All analytics were analyzed with version 1.2.2 of the pipeline.

Test Limitation

Samples with a tumor content of less than 20% may have reduced sensitivity, potentially leading to false negative results. A lack of a variant call does not necessarily indicate the absence of a variant, as technical limitations may restrict data acquisition in certain genetic regions. Additionally, it is possible that the sample contains a mutation below our established limit of detection (1% allele frequency in hotspots, 5%

in other regions), or in a gene excluded by our assay. Alterations present in repetitive or high GC content region may not be detected. The inherent DNA fragmentation, damage, and background noise in FFPE samples can reduce the sensitivity and specificity of copy number alterations (amplifications/deletions) and structural variations.

References

1. Li MM, Datto M, Duncavage EJ, Kulkarni S, Lindeman NI, Roy S, Tsimberidou AM, Vnencak-Jones CL, Wolff DJ, Younes A, Nikiforova MN. Standards and Guidelines for the Interpretation and Reporting of Sequence Variants in Cancer: A Joint Consensus Recommendation of the Association for Molecular Pathology, American Society of Clinical Oncology, and College of American Pathologists. J Mol Diagn. 2017 Jan;19(1):4-23. doi: 10.1016/j.jmoldx.2016.10.002. PMID: 27993330; PMCID: PMC5707196.
2. Alexandrov LB, Kim J, Haradhvala NJ, Huang MN, Tian Ng AW, Wu Y, Boot A, Covington KR, Gordenin DA, Bergstrom EN, Islam SMA, Lopez-Bigas N, Klimczak LJ, McPherson JR, Morganella S, Sabarinathan R, Wheeler DA, Mustonen V; PCAWG Mutational Signatures Working Group; Getz G, Rozen SG, Stratton MR; PCAWG Consortium. The repertoire of mutational signatures in human cancer. Nature. 2020 Feb; 578(7793):94-101. doi: 10.1038/s41586-020-1943-3. Epub 2020 Feb 5. Erratum in: Nature. 2023 Feb;614(7948):E41. PMID: 32025018; PMCID: PMC7054213.
3. Catalogue of Somatic Mutations in Cancer (COSMIC, cancer.sanger.ac.uk), Tate, J.G, et al. (2019) COSMIC: the catalogue of somatic mutations in cancer, Nucleic Acids Research 47(D1):D941–D947 (https://doi.org/10.1093/nar/gky1015)

Disclaimer

This report is intended to provide information to the treating physician and is not intended to guarantee or promise the efficacy or usefulness of any particular drug or treatment regimen for any patient. The potential clinical benefit of any drug listed in this report may vary based on a variety of factors, including the patient's specific tumor type and other clinical considerations. In the event of a germline mutation detected under the "Germline Mutation Summary" section of this report, Inocras strongly suggests that the patient receive appropriate genetic counseling to explain the implications of this test result, its residual risks and uncertainties, and the reproductive or medical options it raises for the patient.

This test was developed, and quality-assured by Inocras, Inc. It has not been cleared or approved by the US Food and Drug Administration. The test has been validated as a Laboratory Developed Test per institutional and applicable CLIA regulation (CLIA# 05D2280195) as qualified to perform high complexity clinical laboratory testing. Data interpretations are based on our current understanding of genes and variants as of the report date. Alterations are listed alphabetically and not in order of strength of evidence or appropriateness for the patient's disease. When the report does identify variants with therapeutic implications, this does not promise or guarantee that a particular drug or treatment regimen will be effective or helpful in the treatment of disease in any patient, and the selection of any drug for patient treatment is done at the discretion of the treating physician. Genomic alterations should be considered in the context of the patient's history, risk factors and any previous genomic testing. Consideration of Variants of Unknown Significance (VUS) may associate with potential therapies in the future. Inocras does not proactively update reports or send notifications regarding the reclassification of these alterations.

Proprietary and confidential material disclaimer: This report contains confidential and proprietary information as well as intellectual property owned by Inocras. It is strictly prohibited to use, disclose, or reproduce any of the information within this document, except for the treatment of the specific patient for which it is intended.

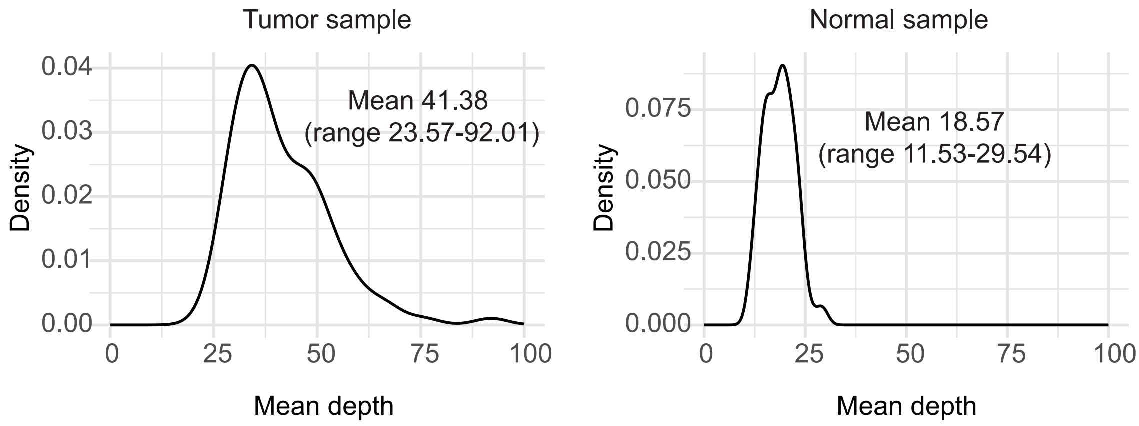

Supplementary Fig. 2. Local sequencing read-depth of tumor and normal samples.

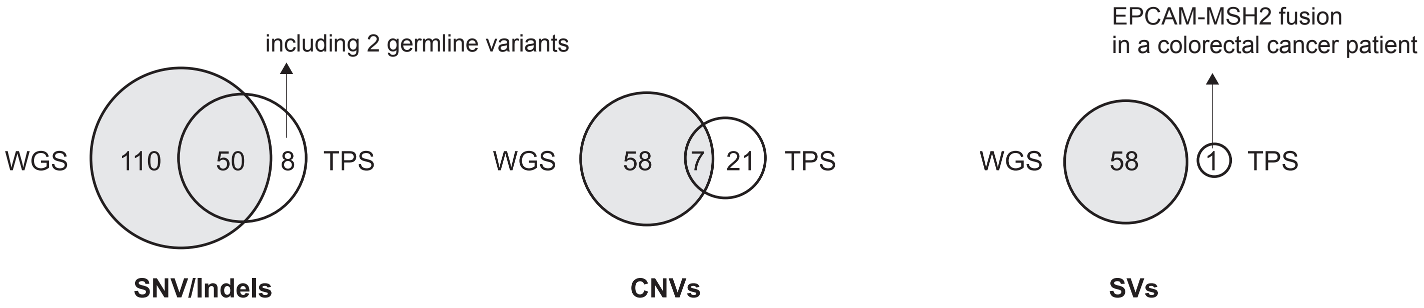

Supplementary Fig. 3. Comparison between whole genome sequencing (WGS) and targeted panel sequencing (TPS) in eight patients who underwent both tests in terms of single nucleotide variants and indels (SNV/Indels), copy number variations (CNVs), and structural variations (SVs).

Supplementary Fig. 1. A standardized patient report of CancerVisionTM.

CANCERVISION

Patient ID		Original Name			
Age at Registration		Specimen Type	Other	Tumor Collection Time	2022-09-19 00:00:00
Sex at Birth		Ordering Provider		Normal Collection Time	
Accession #		Ordering Client		Registration Date	2022-09-28 19:00:00
Diagnosis	[8140/3] Adenocarcinoma, NOS				

Report summary

Tumor mutation count 6,966 Driver copy number alterations 3 Driver structural variations 6

1. Key Biomarker Findings

**The prescribing information for the FDA-approved therapeutic option may not include the associated key biomarkers and it should be noted this information does not pertain to pediatric indications.*

Key biomarkers	Origin	FDA-approved drugs for patient's cancer	FDA-approved drugs for another cancer
TP53 E343*	Somatic	None	Acalabrutinib + Obinutuzumab, Ibrutinib, Duvelisib, etc.
BRCA1 NP_009225.1:p.Leu1780Pro (germline)	Germline	None	Olaparib + Talazoparib

2. Germline Mutation Summary

**Please refer to next pages for more detailed descriptions.*

One pathogenic and no likely pathogenic germline variant detected.

3. Genomic Instability

**Please refer to next pages for additional descriptions and reference ranges.*

	Tumor mutation burden	Microsatellite status	Homologous recombination deficiency
Result	Low	Stable	Deficient
Score	2.43 mut/Mb	2.54	0.93

4. Key signatures

Results are signatures with >10% contributions in SBS mutational signature analysis other than clock-like, unknown signatures. Please refer to next pages for more detailed descriptions.

Exogenous signature: ☐ UV light exposure ☐ Tobacco smoking signature ☐ Other

*e.g. Aflatoxin exposure

Endogenous etiology signature: ☑ HRD signature ☐ Defective DNA mismatch repair ☐ APOBEC signature ☑ Other

*e.g. polymerase eta somatic hypermutation

Supplementary Figure 1.

Actionable Findings

Genomic alteration	Treatment	Disease	Evidence level
Not found			

Level of Evidence Scoring: The level calls aligns with the AMP/ASCO/CAP consensus, ranging from A (FDA-approved biomarkers) to D (emerging diagnostic and therapeutic relevance)[1]

Somatic Driver Alterations

Single Nucleotide Variations and Small Indels

Mode	Gene symbol	Variant	Consequence	Variant allele frequency	Loss of heterozygosity
Tumor suppressor gene	TP53	E343*	Stop gained	0.138	Present
Tumor suppressor gene	MYH9	E1131Gfs*31	Frameshift variant	0.06	Absent

Copy Number Alterations

Mode	Gene symbol	Consequence	Copy number
Oncogene	QKI	Amplification	9
Oncogene	AFDN	Amplification	18
Oncogene	KRAS	Amplification	8

Structural Variations

Mode	Gene symbol	Consequence	Type	Loss of heterozygosity
Tumor suppressor gene	MAP3K13	Disruption	.	Absent
Tumor suppressor gene	FOXO3	Disruption	.	Absent
Tumor suppressor gene	POT1,NF1	Disruption	.	Absent
Tumor suppressor gene	NDRG1	Disruption	.	Absent
Tumor suppressor gene	PTEN	Disruption	.	Absent
Tumor suppressor gene	RUNX1	Disruption	.	Absent

Germline Cancer-related Variants

Pathogenicity	Mode	Gene	Variant type	Variant	Genotype	Loss of heterozygosity
Pathogenic	_	BRCA1	PM (SNV)	NP_009225.1:p.Leu1780Pro	Het	Present

Tumor Mutational Burden

(Score: 2.43 mut/Mb)

SNVs		Indels	
Count	5,705	Count	1,261
Density	1.99 mut/Mb	Density	0.44 mut/Mb

**The Tumor Mutational Burden (TMB) score represents the number of mutations per Mb across the whole genome of the tumor. A tumor is considered to have a high TMB if the score is > 10mut/mb.*

Mutational Signature

Single Base Substitutions

Signature	Estimated count	Contribution (%)	Proposed etiology
SBS3	2560.6	45.4	Defective homologous recombination DNA damage repair
SBS5	749.36	13.3	Unknown (clock-like signature)
SBS40	745.14	13.2	Unknown

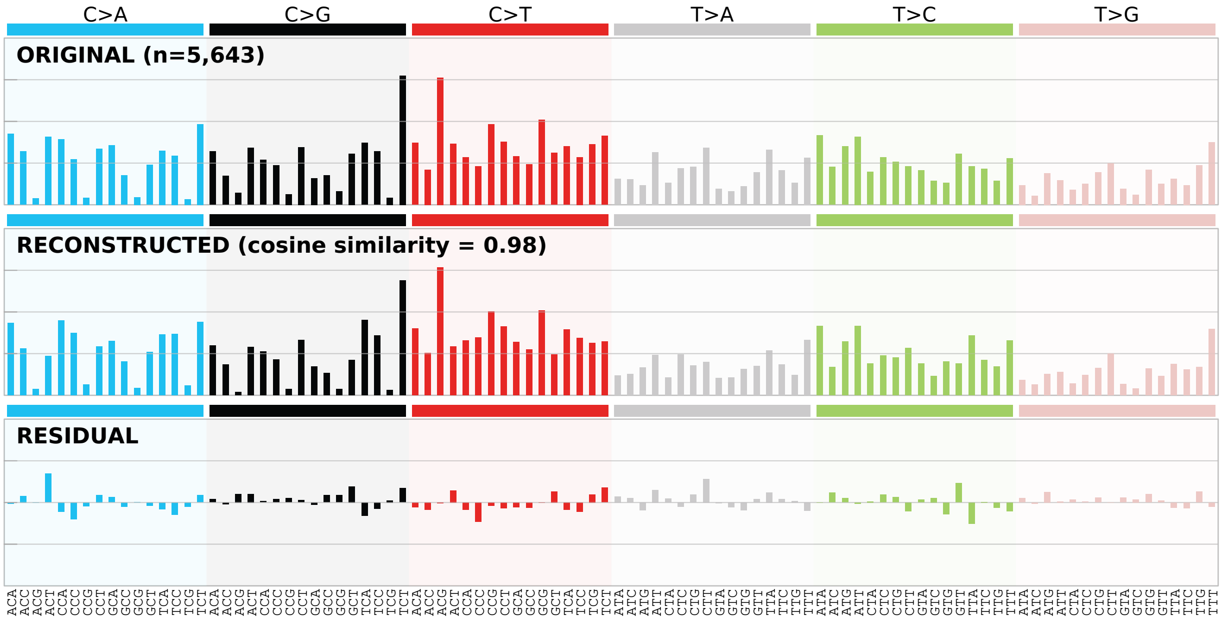

Mutational signature analysis categorizes the found somatic substitutions into 96 mutational contexts, creates a histogram, and then finds the known signature combination that best describes the relevant histogram. Combination of 6 possible types of substitutions and 4 types of nucleotides, that can be present in front of or following the mutational location, produces 96 possible cases (6x4x4).

Indels

Signature	Estimated count	Contribution (%)	Proposed etiology
ID2	496.92	61.6	Slippage during DNA replication of the replicated DNA strand

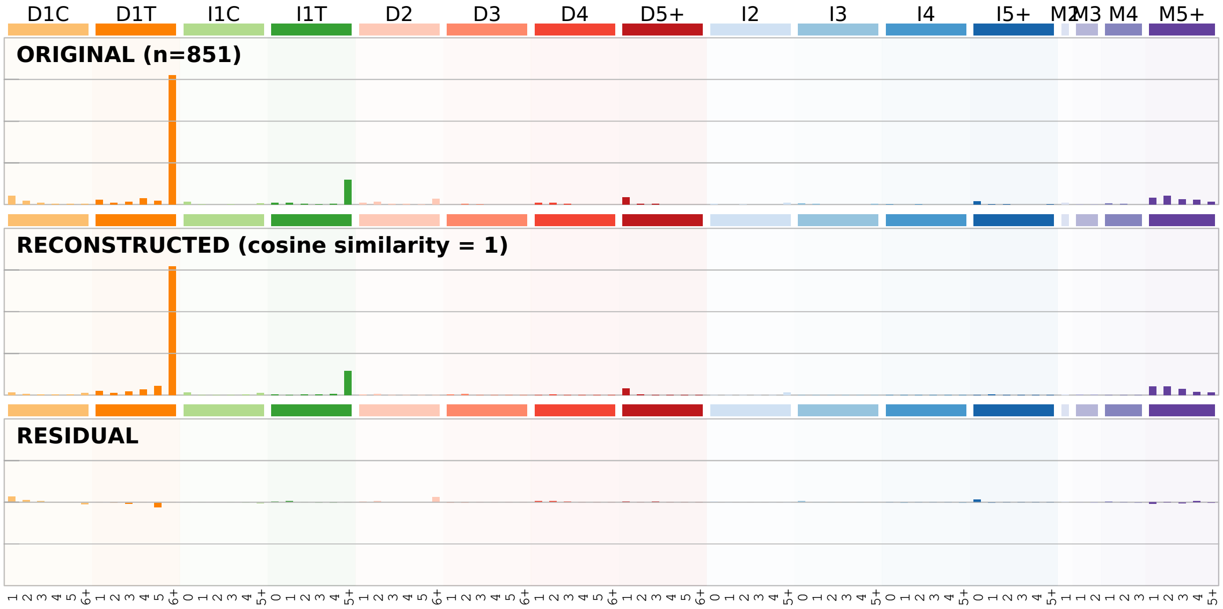

Supplementary Figure 1.

Structural Variations

Signature	Estimated count	Contribution (%)	Proposed etiology
RS3	151.26	64.0	Unknown
RS5	42.56	18.0	Unknown
RS2	37.5	15.8	Unknown

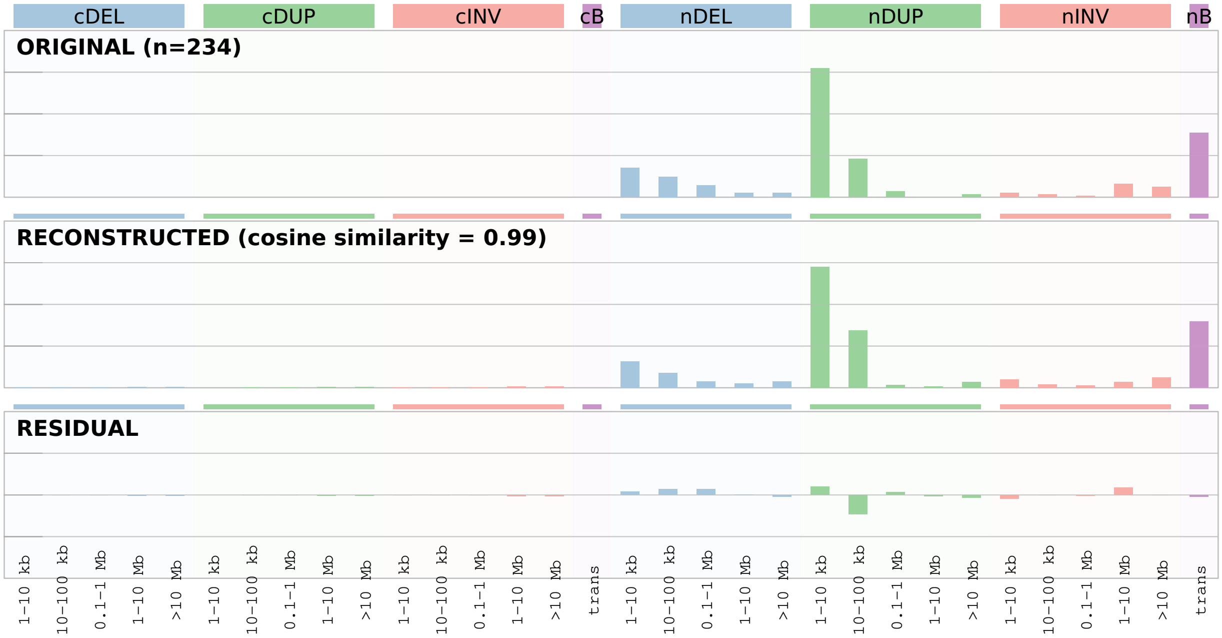

Supplementary Figure 1.

Double Base Substitutions

Signature	Estimated count	Contribution (%)	Proposed etiology
DBS7	8.52	36.4	Unknown
DBS2	8.28	35.4	Unknown
DBS4	6.57	28.1	Unknown

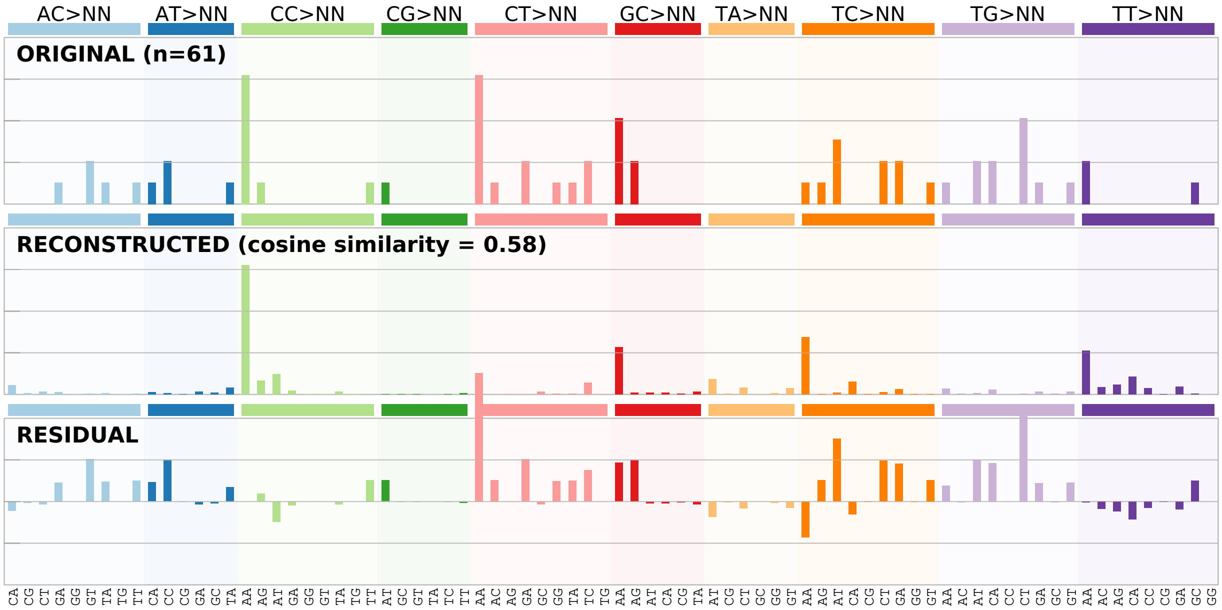

Microsatellite Instability

Stable (Score : 2.54)

Total number of site	2321
Number of somatic site	59
MSI score	2.54

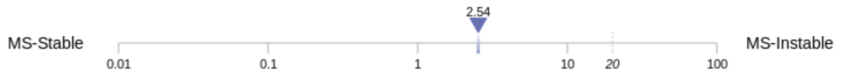

The microsatellite instability (MSI) score represents the number of somatic insertions and deletions per Mb in microsatellite regions across whole genome of the tumor. A tumor is considered microsatellite stable (MSS) if the score is < 20, and MSI-High if > 20.

Homologous Recombination Defect (HRD)

Deficient (Score : 0.93)

SBS3	2,745.0
SBS8	777.0
ID6	8.5%
RS3	151.0
RS5	43.0
LOH/TAI/LST	50

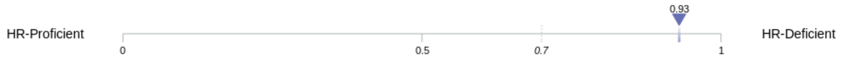

The HRD score is determined by an in-house WGS signature-based classifier comparing the signature of this sample with signatures found across samples with known BRCA1/BRCA2 inactivation. Tumors with a score greater than or equal to 0.7 are considered HR-deficient(Positive), while those with a score below this threshold are considered HR-proficient(Negative).
LOH; Loss Of Heterozygosity, TAI; Telomeric Allelic Imbalance, LST; Large-scale State Transition

Genome Portrait

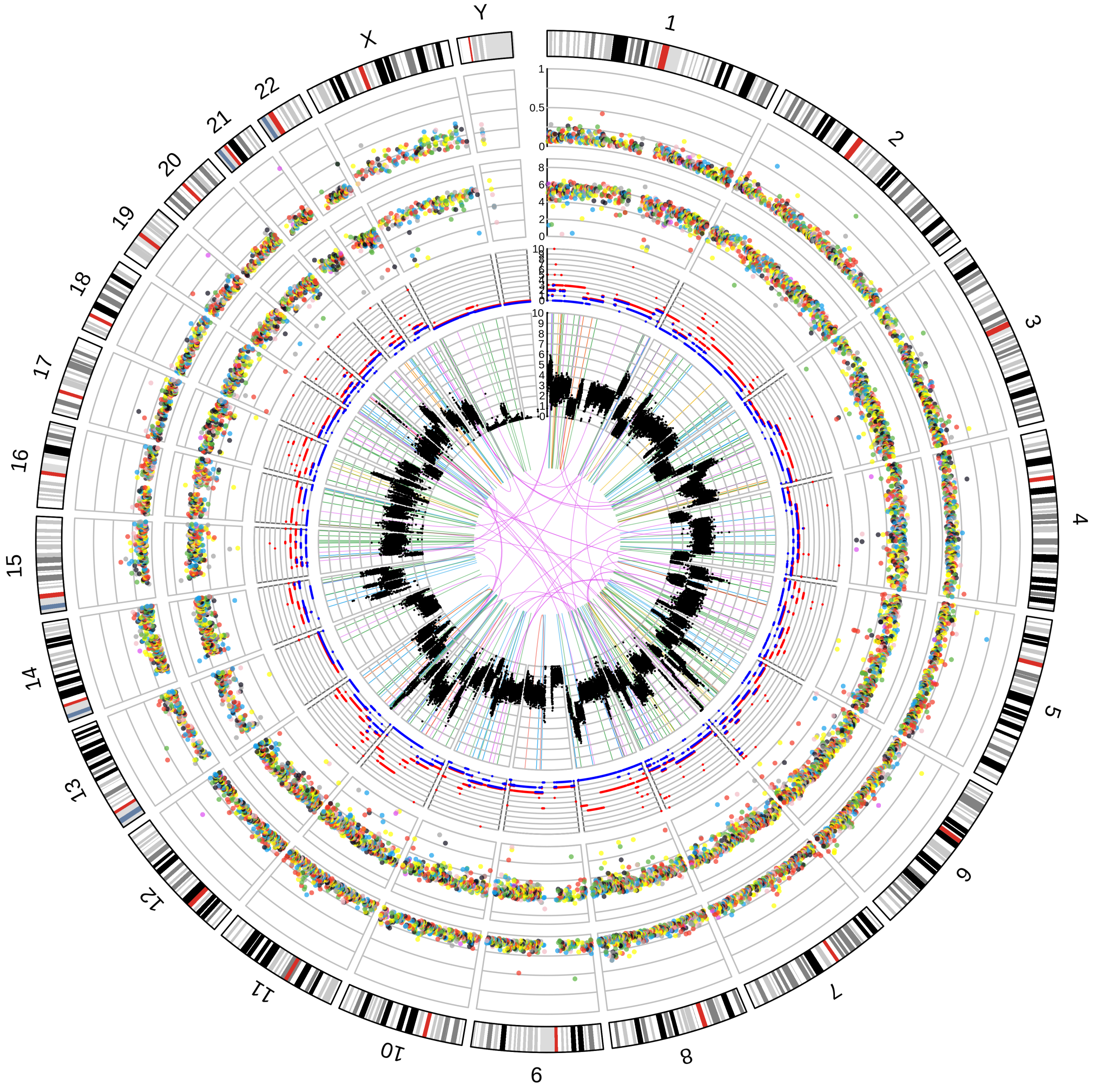

Point mutations
(n = 6,965)
- C>A (996)
- C>G (962)
- C>T (1,391)
- T>A (733)
- T>C (957)
- T>G (604)
- MNV (61)
- Indel (1,261)

Copy number variations
- Major allele
- Minor allele

Structural variations
(n = 234)
- Deletion (48)
- Duplication (120)
- 5to5 inversion (11)
- 3to3 inversion (11)
- Translocation (44)

Information in the CIRCOS plot are: cytoband, variant allele fraction of point mutations, intermutational distance of point mutations (logarithmic scale), copy number of each allele (red lines: major, blue lines: minor) total copy number (black dots), and structural variations (color lines), from the outside of the circle to the inside, in their respective orders.

Test Information

The CancerVision test is a Next Generation Sequencing tumor/normal whole genome assay that provides a list of detected single nucleotide variants (SNVs), multiple nucleotide variants (MNVs), small insertions and deletions (indels), copy number alterations (CNAs), and structural variations (SVs) in tumor tissue, along with an analysis report of mutational signature, tumor mutational burden (TMB), microsatellite instability (MSI), and homologous recombination deficiency (HRD). The genomic DNA is extracted from the patient's normal and tumor samples via the ThermoFisher KingFisher Apex and prepared using the Watchmaker Genomics enzymatic library preparation which includes end repair, purification, adapter ligation and PCR amplification. A portion of the library is hybridized to targeted probes. The libraries are sequenced using the Illumina NovaSeq X+. The sequence data are analyzed using various validated bioinformatics tools and a custom data-processing pipeline for Next Generation Sequencing platforms. GRCh38 is used for human reference genome. Each tumor's cancer-specific mutations are then queried against a proprietary gene-drug database based on peer-reviewed literature to identify potential therapeutic associations; however, this information should be considered in conjunction with other clinical and diagnostic findings.

- Whole genome sequencing: mean target coverage is 40x (unique reads) for tumor sample DNA, and 20x (unique reads) for a normal sample.
- Variant calling was performed using publicly and commercially available tools. Then, variant candidates were examined and refined by pipelines developed by Inocras. The pipelines have been established by a team of experts in genomics and engineering and powered by AI algorithms.
- For formalin-fixed paraffin-embedded (FFPE) specimens, AI-powered data correction algorithms developed in Inocras are used to mitigate data-quality issues commonly encountered in FFPE-derived samples.
- SNVs and indels whose consequences are predicted to activate oncogenes and reported as hotspot mutations by COSMIC were categorized as oncogenic mutations. Those whose consequences cause loss of function of tumor suppressor genes (TSGs) were classified as TSG-disrupting mutations.
- CNAs that amplify oncogenes more than five copies above the average ploidy were classified as oncogene amplification, whereas those that delete both copies of TSGs were categorized as biallelic deletion of TSGs.
- SVs that generate known fusion oncogenes or disrupt TSGs were classified as driver events.
- SVs that produce known fusion oncogenes by connecting two independent genes were classified as fusion oncogene-generating SVs. SVs that alter the arrangement of exons of TSGs were classified as TSG-disrupting SVs.
- Actionable findings list on-label drugs applicable for the patient’s disease.
- Tumor cellularity is a proportion of tumor cells in the specimen of whole-genome sequencing. Somatic mutations may be under-detected when tumor cellularity is low.
- Variant allele frequency (VAF) is the fraction of variant-supporting reads among total sequencing reads and is dependent on tumor cellularities.
- Cancer cell fraction (CCF) of a mutation is the fraction of cancer cells harboring the mutation. CCF less than 1 indicates subclonal mutation.
- Tumor mutation burden (TMB) is the number of somatic SNVs, indels, and SVs divided by the effective genome size (~2.9 Gb).
- The mutational signature analysis is primarily based on the COSMIC mutational signatures of SNVs, double-base substitution (DSB), and indels. SV signatures were adopted from the previous studies[2].
- Microsatellite instability (MSI) score is calculated by genome-widely examining microsatellite regions.
- Homologous recombination deficiency (HRD) is evaluated through an in-house script.
- Measure of Genomic Instability, an unweighted sum of loss of heterozygosity (LOH, the total number of LOH regions across the entire genome that are larger than 15Mb but do not involve whole chromosome), telomeric allelic imbalance (TAI, the number of regions with allelic imbalance extending to the subtelomere but not crossing the centromere), and large-scale state transition (LST, the number of break points occurring between adjacent regions of at least 10 Mb). A value greater than or equal to 42 is regarded as having genomic instability.
- The OncoKB™ precision oncology knowledge base was made available under license from Memorial Sloan Kettering Cancer Center.
- The Catalogue of Somatic Mutations in Cancer (COSMIC, cancer.sanger.ac.uk) was made available under COSMIC Static Reporting Startup License from Qiagen[3].
- All analytics were analyzed with version 1.2.2 of the pipeline.

Test Limitation

Samples with a tumor content of less than 20% may have reduced sensitivity, potentially leading to false negative results. A lack of a variant call does not necessarily indicate the absence of a variant, as technical limitations may restrict data acquisition in certain genetic regions. Additionally, it is possible that the sample contains a mutation below our established limit of detection (1% allele frequency in hotspots, 5%

in other regions), or in a gene excluded by our assay. Alterations present in repetitive or high GC content region may not be detected. The inherent DNA fragmentation, damage, and background noise in FFPE samples can reduce the sensitivity and specificity of copy number alterations (amplifications/deletions) and structural variations.

References

1. Li MM, Datto M, Duncavage EJ, Kulkarni S, Lindeman NI, Roy S, Tsimberidou AM, Vnencak-Jones CL, Wolff DJ, Younes A, Nikiforova MN. Standards and Guidelines for the Interpretation and Reporting of Sequence Variants in Cancer: A Joint Consensus Recommendation of the Association for Molecular Pathology, American Society of Clinical Oncology, and College of American Pathologists. J Mol Diagn. 2017 Jan;19(1):4-23. doi: 10.1016/j.jmoldx.2016.10.002. PMID: 27993330; PMCID: PMC5707196.
2. Alexandrov LB, Kim J, Haradhvala NJ, Huang MN, Tian Ng AW, Wu Y, Boot A, Covington KR, Gordenin DA, Bergstrom EN, Islam SMA, Lopez-Bigas N, Klimczak LJ, McPherson JR, Morganella S, Sabarinathan R, Wheeler DA, Mustonen V; PCAWG Mutational Signatures Working Group; Getz G, Rozen SG, Stratton MR; PCAWG Consortium. The repertoire of mutational signatures in human cancer. Nature. 2020 Feb; 578(7793):94-101. doi: 10.1038/s41586-020-1943-3. Epub 2020 Feb 5. Erratum in: Nature. 2023 Feb;614(7948):E41. PMID: 32025018; PMCID: PMC7054213.
3. Catalogue of Somatic Mutations in Cancer (COSMIC, cancer.sanger.ac.uk), Tate, J.G, et al. (2019) COSMIC: the catalogue of somatic mutations in cancer, Nucleic Acids Research 47(D1):D941–D947 (https://doi.org/10.1093/nar/gky1015)

Disclaimer

This report is intended to provide information to the treating physician and is not intended to guarantee or promise the efficacy or usefulness of any particular drug or treatment regimen for any patient. The potential clinical benefit of any drug listed in this report may vary based on a variety of factors, including the patient's specific tumor type and other clinical considerations. In the event of a germline mutation detected under the "Germline Mutation Summary" section of this report, Inocras strongly suggests that the patient receive appropriate genetic counseling to explain the implications of this test result, its residual risks and uncertainties, and the reproductive or medical options it raises for the patient.

This test was developed, and quality-assured by Inocras, Inc. It has not been cleared or approved by the US Food and Drug Administration. The test has been validated as a Laboratory Developed Test per institutional and applicable CLIA regulation (CLIA# 05D2280195) as qualified to perform high complexity clinical laboratory testing. Data interpretations are based on our current understanding of genes and variants as of the report date. Alterations are listed alphabetically and not in order of strength of evidence or appropriateness for the patient's disease. When the report does identify variants with therapeutic implications, this does not promise or guarantee that a particular drug or treatment regimen will be effective or helpful in the treatment of disease in any patient, and the selection of any drug for patient treatment is done at the discretion of the treating physician. Genomic alterations should be considered in the context of the patient's history, risk factors and any previous genomic testing. Consideration of Variants of Unknown Significance (VUS) may associate with potential therapies in the future. Inocras does not proactively update reports or send notifications regarding the reclassification of these alterations.

Proprietary and confidential material disclaimer: This report contains confidential and proprietary information as well as intellectual property owned by Inocras. It is strictly prohibited to use, disclose, or reproduce any of the information within this document, except for the treatment of the specific patient for which it is intended.

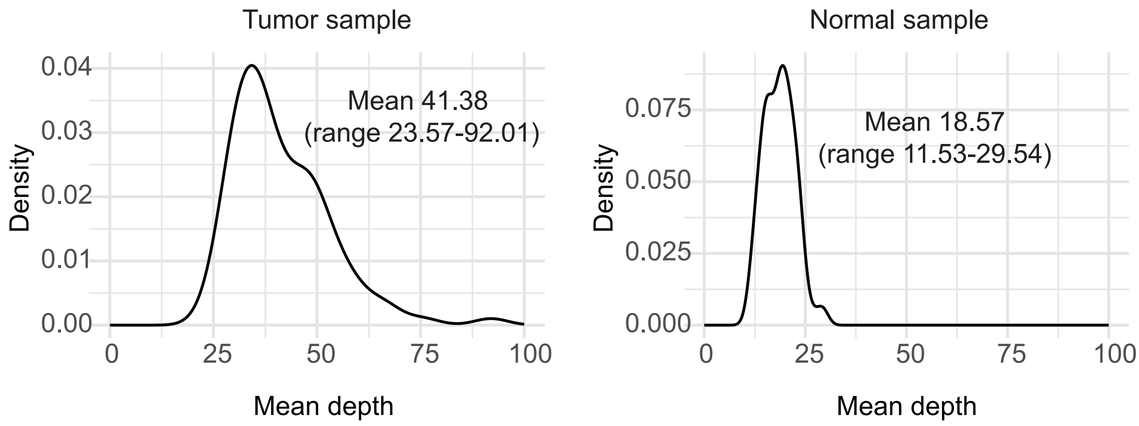

Supplementary Fig. 2. Local sequencing read-depth of tumor and normal samples.

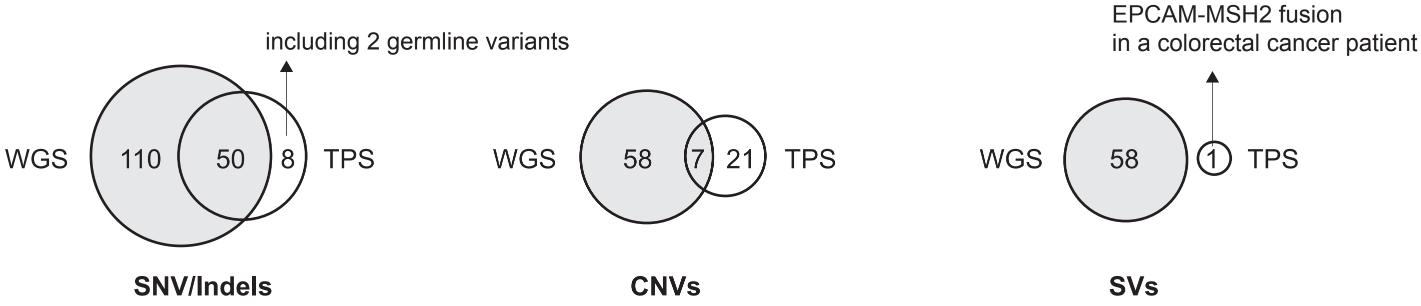

Supplementary Fig. 3. Comparison between whole genome sequencing (WGS) and targeted panel sequencing (TPS) in eight patients who underwent both tests in terms of single nucleotide variants and indels (SNV/Indels), copy number variations (CNVs), and structural variations (SVs).

Supplementary Table 1. Clinicopathological characteristics and Categories for Clinical Utility of WGS

Order	Screen ID	Case No	Sex	Age	Diagnosis	Tissue type	Staging	Title of surgery or biopsy	Organ of surgery/biopsy	FFPE / Fresh Frozen	Sample_type	Disease status (localized, metastatic)	Time-point (Tx-naive On treatment Relapsed)	Tumor Purity (calculated by WGS pipeline)	Clinical Question	Answer for clinical question or Clinically Significant Alterations	Clinical Utility Category	Related Clinical Trials	Reasons for Screen fail or Q/C fail
1	36-P-001	1	F	81	Non-smal cell lung cancer	Adenocarcinoma	cT4N3M1c	Bronchoscopy/EBUS	Bronchus RUL LN#7 LN #4R	Fresh Frozen	biopsy	metastatic	On treatment	0.2	Category II (Clarity)	EGFR mutation with EGFR amplification (CN78)	II-1 (Drug resistance/responsive mechanism)		
2	36-P-007	2	M	60	Colorectal cancer	Adenocarcinoma	cT4N2M1	Colonoscopy	Transverse colon	Fresh Frozen	biopsy	metastatic	relapsed	0.7	Category I (actionability)	PIK3CA E545K	I-2 (Screen for mutation-specific clinical trials)	NCT04753203	
3	36-P-008	3	M	54	Stomach cancer	Poorly cohesive carcinoma	cT4aN3M1	Total gastrectomy	stomach Peritoneum	Fresh Frozen	surgery	metastatic	Tx-naive	0.15	Category I (actionability)	germline BRCA1 L1790P + LOH	I-2 (Screen for mutation-specific clinical trials)	NCT04171700	
4	36-P-009	4	F	53	Breast cancer	Invasive ductal carcinoma (ER+,PR+,HER-2-)	cT4N1M1	US-guided core-needle biopsy	Breast, Rt.	Fresh Frozen	biopsy	metastatic	Tx-naive	0.9	Category II (Clarity)	Sporadic breast cancer	II-3 (Familial cancer)		
5	36-P-010	5	M	67	Colorectal cancer	Adenocarcinoma	cT4N3M1	Sigmiodoscopy	Sigmoid colon	Fresh Frozen	biopsy	metastatic	Tx-naive	0.28	Category I (actionability)		No		
6	36-P-011	6	F	54	Non-smal cell lung cancer	Adenocarcinoma	cT1bN0M0	Rt. Lower lobectomy	Lung, RLL	Fresh Frozen	surgery	localized	Tx-naive	0.27	Category I (actionability)	EGFR G719A, A776H	I-1 (Selection of targeted therapeutics)		
7	36-P-012	7	M	44	Stomach cancer	Adenocarcinoma	cT4N3M1	Total gastrectomy	Stomach	Fresh Frozen	surgery	metastatic	Tx-naive	0.99	Category I (actionability)		No		
8	36-P-013	8	M	78	Gastrointestinal stromal tumor	Gastrointestinal stromal tumor	cTxNxM1	Tumor excision, Small bowel segmental resection	Gastrojejunum	Fresh Frozen	surgery	metastatic	relapsed	0.89	Category I (actionability)	PDGFRA D842V	I-1 (Selection of targeted therapeutics)		
9	36-P-014	9	M	57	Non-smal cell lung cancer	Dedifferentiated liposarcoma	cT4N3M1	Rt. wedge resection	Lung, RLL	Fresh Frozen	surgery	metastatic	Tx-naive	0.99	Category I (actionability)	EML4-ALK	I-1 (Selection of targeted therapeutics)		
10	36-P-016	10	M	81	Stomach cancer	Adenocarcinoma	cT3N1M0	Total gastrectomy	Stomach	Fresh Frozen	surgery	localized	Tx-naive	0.97	Category I (actionability)	MSI-H	I-1 (Selection of targeted therapeutics)		
11	36-P-017	11	M	84	Colorectal cancer	Mucinous adenocarcinoma	cT4N3M1	Anterior Resection	Sigmoid colon	Fresh Frozen	surgery	metastatic	Tx-naive	0.71	Category I (actionability)	BRAF V600E	I-1 (Selection of targeted therapeutics)		
12	36-P-018	12	F	60	Colorectal cancer	Adenocarcinoma	cT3N0M0	Extended Rt. hemicolectomy	Colon, Rt.	Fresh Frozen	surgery	localized	Tx-naive	0.24	Category I (actionability)		No		
13	36-P-019	13	M	74	Non-smal cell lung cancer	Adenocarcinoma	cT1aN0M0	Lt. segmentectomy	Lung, LUL	Fresh Frozen	surgery	localized	Tx-naive	0.95	Category I (actionability)		No		
14	36-P-020	14	F	61	Colorectal cancer	Adenocarcinoma	cT4N1M1	US-guided core-needle biopsy	Liver	Fresh Frozen	biopsy	localized	Tx-naive	0.51	Category I (actionability)	BRAF V600E	I-1 (Selection of targeted therapeutics)		
15	36-P-021	15	M	79	Non-smal cell lung cancer	Adenocarcinoma	cT1N0M0	Lt. Segmentectomy	LLL	Fresh Frozen	surgery	localized	Tx-naive	0.98	Category I (actionability)	EGFR L858R	I-1 (Selection of targeted therapeutics)		
16	36-P-022	16	F	54	Breast cancer	Invasive ductal carcinoma (ER+,PR+,HER-2-)	rTxN0M1	Rt. Wedge resection	Lung, RUL	Fresh Frozen	surgery	metastatic	relapsed	0.53	Category I (actionability)	NRG1 fusion (CFAP52-NRG1 fusion)	I-2 (Screen for mutation-specific clinical trials)	NCT04100694	
17	36-P-023	17	M	54	Colorectal cancer	Adenocarcinoma	cT4N0M0	Rt. Hemicolectomy	Colon, Rt.	Fresh Frozen	surgery	localized	Tx-naive	0.46	Category I (actionability)	MSI-H	I-1 (Selection of targeted therapeutics)		
18	36-P-024	18	M	55	Colorectal cancer	Adenocarcinoma	cT4N1M1	Hartman's operation	Rectosigmoid junction	Fresh Frozen	surgery	metastatic	Tx-naive	0.59	Category I (actionability)	KRAS G12V	I-3 (Elimination of ineffective treatment options)		
19	36-P-025	19	M	68	Non-smal cell lung cancer	Adenocarcinoma	cT1N0M0	Lt segmentectomy, wedge resection	Lung, LUL	Fresh Frozen	surgery	localized	Tx-naive	0.99	Category I (actionability)	EGFR L858R	I-1 (Selection of targeted therapeutics)		
20	36-P-026	20	F	74	Non-smal cell lung cancer	Adenocarcinoma	cT2N0M1	Rt. wedge resection	Lung, RLL, RUL	Fresh Frozen	surgery	metastatic	Tx-naive	0.16	Category I (actionability)	EGFR exon 19 in-frame deletion	I-1 (Selection of targeted therapeutics)		
21	36-P-027	21	F	74	Colorectal cancer	Adenocarcinoma	cT2N0M0	Extended Rt. hemicolectomy	Colon, Rt.	Fresh Frozen	surgery	localized	Tx-naive	0.37	Category I (actionability)	NRAS 61	I-3 (Elimination of ineffective treatment options)		
22	36-P-028	22	M	52	Colorectal cancer	Adenocarcinoma	cT4aN1M0	Rt. Hemicolectomy	Colon, Rt.	Fresh Frozen	surgery	localized	Tx-naive	0.52	Category I (actionability)	BRAF V600E	I-1 (Selection of targeted therapeutics)		
23	36-P-029	23	M	72	Stomach cancer	Adenocarcinoma	cT4N2M0	EGD	Stomach	Fresh Frozen	biopsy	localized	Tx-naive	0.3	Category I (actionability)	MSI-H	I-1 (Selection of targeted therapeutics)		
24	36-P-030	24	F	81	Colorectal cancer	Adenocarcinoma	cT4bN1M1	Anterior Resection	Sigmoid colon	Fresh Frozen	surgery	metastatic	Tx-naive	0.23	Category I (actionability)		No		
25	36-P-031	25	M	35	Colorectal cancer	Adenocarcinoma	cT3N1M0	Lt. wedge resection	Lung, LUL	Fresh Frozen	surgery	localized	Tx-naive	0.68	Category II (Clarity)		II-3 (Familial cancer)		
26	36-P-033	26	F	48	Endometrial carcinoma	Endometrioid adenocarcinoma	rTxNxM1	Rt. wedge resection	Lung, RLL	Fresh Frozen	surgery	metastatic	Tx-naive	1	Category I (actionability)	PTEN mutation (p.A120GfsTer5 and p.T319Ter)	I-2 (Screen for mutation-specific clinical trials)	NCT05038839	
27	36-P-034	27	F	40	Colorectal cancer	Adenocarcinoma	cT4aN1M0	Lt. Hemicolectomy	Descending colon	Fresh Frozen	surgery	localized	Tx-naive	0.49	Category I (actionability)		No		
28	36-P-035	28	M	74	Colorectal cancer	Adenocarcinoma	cT3N1M0	Rt. Hemicolectomy	Colon, Rt.	Fresh Frozen	surgery	localized	Tx-naive	0.2	Category I (actionability)	KRAS	I-3 (Elimination of ineffective treatment options)		
29	36-P-036	29	M	72	Non-smal cell lung cancer	Large cell neuroendocrine carcinoma	cT2N0M0	Rt. Lower lobectomy	Lung, RLL	Fresh Frozen	surgery	localized	Tx-naive	0.58	Category I (actionability)	KRAS G12D	I-3 (Elimination of ineffective treatment options)	NCT06040541, NCT06179160, NCT05737706	
30	36-P-037	30	M	61	Non-smal cell lung cancer	Squamous cell carcinoma	cT4N1M0 -> relapsed	Bronchoscopy	Lung, RUL	Fresh Frozen	biopsy	metastatic	Tx-naive	0.32	Category II (Clarity)		II-1 (Drug resistance/responsive mechanism) - No		
31	36-P-038	31	M	60	Colorectal cancer	Adenocarcinoma	cT4N2M1	Low Anterior Resection	Rectum	Fresh Frozen	surgery	localized	Tx-naive	0.4	Category II (Clarity)		II-2 (Tumor orgin)		
32	36-P-039	32	F	72	Colorectal cancer	Adenocarcinoma	cT3N0M0	Rt. Hemicolectomy	Colon, Rt.	Fresh Frozen	surgery	localized	Tx-naive	0.15	Category I (actionability)		No		
33	36-P-040	33	M	85	Colorectal cancer	Adenocarcinoma	cT3N1M1c	Abdominoperineal resection	Rectum	Fresh Frozen	surgery	metastatic	Tx-naive	0.4	Category I (actionability)	ERBB2 V842I	I-2 (Screen for mutation-specific clinical trials)	NCT04380012, NCT 03457896, NCT04175397, NCT04579380, NCT0463919	
34	36-P-041	34	M	71	Non-smal cell lung cancer	Adenocarcinoma	cT1N0M1	Resection of small intestine	Jejunum	Fresh Frozen	surgery	metastatic	Tx-naive	0.45	Category II (Clarity)		II-2 (Tumor orgin)		
35	36-P-043	35	M	60	Anal cancer	Squamous cell carcinoma	cT4aN1M1	US-guided core-needle biopsy	Liver	Fresh Frozen	biopsy	metastatic	Tx-naive	0.91	Category II (Clarity)		II-2 (Tumor orgin)		
36	36-P-045	36	F	45	Non-smal cell lung cancer	Adenocarcinoma	cT2aN0M0	Rt. upper Lobectomy	Lung, RUL	Fresh Frozen	surgery	localized	Tx-naive	0.38	Category I (actionability)	KRAS G12D	I-2 (Screen for mutation-specific clinical trials)	NCT06040541, NCT06179160, NCT05737706	
37	36-P-047	37	F	55	Non-smal cell lung cancer	Adenocarcinoma	cT4N1M1	Bronchoscopy	Lung, RLL	Fresh Frozen	biopsy	metastatic	Tx-naive	0.29	Category II (Clarity)		II-1 (Drug resistance/responsive mechanism) - No		
38	36-P-048	38	F	36	Non-smal cell lung cancer	Adenocarcinoma	cT1cN0M0	Lobectomy	LLL	Fresh Frozen	surgery	localized	Tx-naive	0.54	Category I (actionability)	EML4-ALK -compex rearrangement	I-1 (Selection of targeted therapeutics)		
39	36-P-049	39	M	54	Renal cell carcinoma	clear cell carcinoma	rTxNxM1	CT-guided core-needle biopsy	Abdominal wall	Fresh Frozen	biopsy	metastatic	Tx-naive	0.41	Category II (Clarity)		II-1 (Drug resistance/responsive mechanism) - No		
40	36-P-051	40	F	46	Stomach cancer	Adenocarcinoma	cT4N2M0->sT4bN31P3	Total gastrectomy	Stomach	Fresh Frozen	surgery	metastatic	On treatment	0.29	Category II (Clarity)		II-1 (Drug resistance/responsive mechanism)		
41	36-P-052	41	F	73	Non-smal cell lung cancer	Adenocarcinoma	cT2N0M0	Lt. segmentectomy	Lung, LUL	Fresh Frozen	surgery	localized	Tx-naive	0.21	Category I (actionability)	EGFR L858R	I-1 (Selection of targeted therapeutics)		
42	36-P-053	42	M	52	Colorectal cancer	Adenocarcinoma	cT4aN2	Anterior Resection	Sigmoid colon	Fresh Frozen	surgery	localized	Tx-naive	0.5	Category I (actionability)		No		
43	36-P-054	43	F	51	Stomach cancer	Adenocarcinoma	cT4N2M0	Total gastrectomy	Stomach	Fresh Frozen	surgery	localized	Tx-naive	0.67	Category I (actionability)	MSI-H	I-1 (Selection of targeted therapeutics)		
44	36-P-056	44	M	74	Pancreatic cancer	Adenocarcinoma	borderline resectable	US-guided core-needle biopsy	Liver	Fresh Frozen	biopsy	metastatic	Tx-naive	0.35	Category I (actionability)		No		
45	36-P-058	45	M	50	Stomach cancer	Adenocarcinoma	cT4aN2M0	Distal Gastrectomy	Stomach	Fresh Frozen	surgery	localized	Tx-naive	0.98	Category I (actionability)	MSI-H	I-1 (Selection of targeted therapeutics)		

Supplementary Table 1. Clinicopathological characteristics and Categories for Clinical Utility of WGS

Order	Screen ID	Case No	Sex	Age	Diagnosis	Tissue type	Staging	Title of surgery or biopsy	Organ of surgery/biopsy	FFPE / Fresh Frozen	Sample_type	Disease status (localized, metastatic)	Time-point (Tx-naive On treatment Relapsed)	Tumor Purity (calculated by WGS pipeline)	Clinical Question	Answer for clinical question or Clinically Significant Alterations	Clinical Utility Category	Related Clinical Trials	Reasons for Screen fail or Q/C fail
46	36-P-059	**46**	M	72	Small cell lung cancer	Small cell carcinoma	cT4N3M1a	US-guided core-needle biopsy	Kidney	Fresh Frozen	biopsy	metastatic	relapsed	0.93	Category II (Clarity)		**II-2 (Tumor orgin)**		
47	36-P-060	**47**	M	52	Pancreatic cancer	adenocarcinoma	metastatic	EUS Bx	Pancreas, Head	Fresh Frozen	biopsy	metastatic	On treatment	0.2	Category I (actionability)	**germline BRCA2 + LOH**	**I-1 (Selection of targeted therapeutics)**		
48	36-P-061	**48**	F	54	Colorectal cancer	Adenocarcinoma	cT3N1M0	Anterior Resection	Sigmoid colon	Fresh Frozen	surgery	localized	Tx-naive	0.25	Category I (actionability)	**KRAS G12D**	**I-2 (Screen for mutation-specific clinical trials)**	**NCT06040541, NCT06179160, NCT05737706**	
49	36-P-065	**49**	M	51	Colorectal cancer	Adenocarcinoma	cT3N1M0	Low Anterior Resection	Rectosigmoid junction	Fresh Frozen	surgery	localized	Tx-naive	0.15	Category I (actionability)	**KRAS G12D**	**I-2 (Screen for mutation-specific clinical trials)**	**NCT06040541, NCT06179160, NCT05737706**	
50	36-P-066	**50**	F	83	Urothelial carcinoma	Papillary Urothelial carcinoma, high-grade	cT2N0M0	TURBT	Bladder	Fresh Frozen	surgery	localized	Tx-naive	0.3	Category I (actionability)	**FGFR3 G370C**	**I-1 (Selection of targeted therapeutics)**		
51	36-P-067	**51**	F	58	Pancreatic cancer	adenocarcinoma	metastatic	US-guided core-needle biopsy	Liver	Fresh Frozen	biopsy	metastatic	Tx-naive	0.72	Category I (actionability)		**No**		
52	36-P-068	**52**	M	67	Head and Neck cancer	(submandibular) salivary ductal carcinoma	pT1N2bM0	Rt. SMG WE, Rt. RND	Submandibular gland, Rt.	Fresh Frozen	surgery	Localized	Tx-naive	0.15	Category I (actionability)	**ERBB2 amplification**	**I-1 (Selection of targeted therapeutics)**		
53	36-P-069	**53**	M	58	Urothelial carcinoma	Papillary Urothelial carcinoma, high-grade	cT2N0M0	TURBT	Badder, Rt. ureteral orifice of bladder	Fresh Frozen	surgery	Localized	Tx-naive	0.32	Category I (actionability)	**HRAS Q61K**	**I-2 (Screen for mutation-specific clinical trials)**	**NCT01374789, NCT04284774**	
54	36-P-070	**54**	F	42	Breast cancer	Invasive ductal carcinoma (ER+,PR-,HER-2-)	cT4dN2M0 -> progression	US-guided core-needle biopsy	Breast, Rt..	Fresh Frozen	biopsy	metastatic	On treatment	0.95	Category I (actionability)	**PIK3CA H1047R**	**I-1 (Selection of targeted therapeutics)**		
55	36-P-071	**55**	M	62	Non-smal cell lung cancer	Adenocarcinoma	cT1bN2M1	pleural biopsy under VATS	Peritoneal nodule	Fresh Frozen	biopsy	metastatic	On treatment	0.16	Category I (actionability)	**HIP1-ALK fusion**	**I-1 (Selection of targeted therapeutics)**		
56	36-P-072	**56**	M	58	Stomach cancer	Adenocarcinoma	cT3N0M0	EGD	Stomach	FFPE	biopsy	Localized	Tx-naive	0.18	Category I (actionability)		**No**		
57	36-P-074	**57**	M	75	Urothelial carcinoma	Papillary Urothelial carcinoma, low-grade	cT4N0M0	TURBT	Bladder	FFPE	surgery	localized	Tx-naive	0.88	Category I (actionability)	**FGFR3 S249C**	**I-1 (Selection of targeted therapeutics)**		
58	36-P-075	**58**	M	71	Colorectal cancer	Adenocarcinoma	rTxNxM1	Bronchoscopy	Bronchus RUL	FFPE	biopsy	metastatic	Tx-naive	0.73	Category II (Clarity)		**II-2 (Tumor orgin)**		
59	36-P-076	**59**	F	64	Breast cancer	Invasive ductal carcinoma (ER+,PR-,HER-2-)	cT4N3M1	US-guided core-needle biopsy	Breast, Lt.	Fresh Frozen	biopsy	metastatic	On treatment	0.26	Category II (Clarity)		**II-1 (Drug resistance/responsive mechanism)**		
60	36-P-077	**60**	F	58	Head and Neck cancer	Squamous cell carcinoma	rT4bN0M0	Lt. maxillectomy, Lt. mRND, Lt. Total parotidectomy	Parotid gland, Lt.	Fresh Frozen	surgery	Localized	Tx-naive	0.26	Category I (actionability)		**No**		
61	36-P-078	**61**	M	63	Urothelial carcinoma	Papillary Urothelial carcinoma, low-grade	cT1aN0M0	TURBT	Bladder	Fresh Frozen	surgery	Localized	Tx-naive	0.96	Category I (actionability)	**FGFR3 Y373C**	**I-1 (Selection of targeted therapeutics)**		
62	36-P-083	**62**	M	76	Stomach cancer	Adenocarcinoma	cT3N0M0	Subtotal Gastrectomy	Stomach	Fresh Frozen	surgery	localized	Tx-naive	0.38	Category I (actionability)		**No**		
63	36-P-084	**63**	F	75	Head and Neck cancer	Squamous cell carcinoma	rT4aN3bM0	Lt. SND, Rt. SoND	Tongue	Fresh Frozen	surgery	Localized	Tx-naive	0.52	Category I (actionability)	**CCND1 amplification**	**I-2 (Screen for mutation-specific clinical trials)**	**NCT04439201, NCT04557449**	
64	36-P-086	**64**	F	64	Urothelial carcinoma	Infiltrating urothelial carcinoma, high-grade	cT4N1M0	Robot-assisted laparoscopic radical nephroureterectomy Lt.	Kidney, Lt.	FFPE	surgery	localized	Tx-naive	0.28	Category I (actionability)		**No**		
65	36-P-087	**65**	F	71	Urothelial carcinoma	Infiltrating urothelial carcinoma, high-grade	cT2N0M0	US-guided core-needle biopsy	Cervical lymph node, Lt.	Fresh Frozen	biopsy	localized	Tx-naive	0.55	Category I (actionability)	**HRAS G12D**	**I-2 (Screen for mutation-specific clinical trials)**	**NCT01374789, NCT04284774**	
66	36-P-088	**66**	F	53	Breast cancer	Invasive lobular carcinoma (ER+,PR+,HER-2-)	cT1N0M0	Lt. Partial mastectomy | Total gastrectomy	Breast, Lt. | Stomach	FFPE	surgery	Localized	Tx-naive	0.2	Category I (actionability)	**PIK3CA N345K**	**I-1 (Selection of targeted therapeutics)**		
67	36-P-089	**67**	M	74	Colorectal cancer	Adenocarcinoma	cT3N0M0	EMR, ESD	Rectum	FFPE	biopsy	localized	Tx-naive	0.48	Category I (actionability)	**PIK3CA M1043I**	**I-2 (Screen for mutation-specific clinical trials)**	**NCT04753203**	
68	36-P-090	**68**	M	63	Urothelial carcinoma	Papillary Urothelial carcinoma, high-grade	cT1N0M0	TURBT	Bladder	FFPE	surgery	localized	Tx-naive	0.38	Category I (actionability)	**CCND1 amplification**	**I-2 (Screen for mutation-specific clinical trials)**	**NCT04439201, NCT04557449**	
69	36-P-091	**69**	M	69	Stomach cancer	Adenocarcinoma	cT4N3M1	EGD	Stomach	FFPE	biopsy	metastatic	Tx-naive	0.3	Category I (actionability)	**MET amplification**	**I-2 (Screen for mutation-specific clinical trials)**	**NCT05620628**	
70	36-P-094	**70**	M	64	Stomach cancer	Mucinous adenocarcinoma	cT4N1	Laparoscopic biopsy	Omentum nodule	FFPE	surgery	metastatic	Tx-naive	0.2	Category I (actionability)	**CCND1 amplification**	**I-2 (Screen for mutation-specific clinical trials)**	**NCT02523014**	
71	36-P-095	**71**	F	59	Neuroendocrine tumor	neuroendocrine tumor G2	rTxNxM1	US-guided core-needle biopsy	Liver	Fresh Frozen	biopsy	metastatic	Tx-naive	0.74	Category I (actionability)		**No**		
72	36-P-097	**72**	F	62	Stomach cancer	Poorly cohesive carcinoma	cT4N2M0	EGD	Stomach	FFPE	biopsy	localized	Tx-naive	0.4	Category I (actionability)	**BRAF V600E**	**I-2 (Screen for mutation-specific clinical trials)**	**DRKS00015849**	
73	36-P-098	**73**	M	83	Urothelial carcinoma	Infiltrating urothelial carcinoma, high-grade	cT1N0M0	TURBT	Bladder	FFPE	surgery	localized	Tx-naive	0.15	Category I (actionability)		**No**		
74	36-P-099	**74**	F	70	Stomach cancer	Adenocarcinoma	cT1N0M0	Distal Gastrectomy	Stomach	Fresh Frozen	surgery	localized	Tx-naive	0.26	Category I (actionability)	**MSI-H**	**I-1 (Selection of targeted therapeutics)**		
75	36-P-100	**75**	F	64	Stomach cancer	Adenosquamous carcinoma	cT4bN2M1	Subtotal Gastrectomy Hepatectomy, segmentectomy	Stomach Liver	Fresh Frozen	surgery	metastatic	Tx-naive	0.33	Category I (actionability)		**No**		
76	36-P-101	**76**	M	48	Stomach cancer	poorly differentiated adenocarcinoma	cT4aN2M0	Distal Gastrectomy	Stomach	Fresh Frozen	surgery	localized	Tx-naive	0.3	Category I (actionability)	**germline BRCA2 + LOH**	**I-2 (Screen for mutation-specific clinical trials)**	**NCT04171700**	
77	36-P-102	**77**	M	72	Head and Neck cancer	Squamous cell carcinoma	cT4aN2M0, HPV+	Rt. Tonsil side exciseion	Tonsil, Rt.	FFPE	surgery	localized	Tx-naive	0.54	Category I (actionability)	**AKT E17K**	**I-2 (Screen for mutation-specific clinical trials)**	**NCT05172245**	
78	36-P-103	**78**	F	48	Breast cancer	Invasive ductal carcinoma (ER+,PR+,HER-2-)	cT2N1M0	US-guided core-needle biopsy	Breast, Rt..	Fresh Frozen	biopsy	localized	Tx-naive	0.77	Category I (actionability)		**No**		
79	36-P-104	**79**	M	45	Colorectal cancer	Adenocarcinoma	cT4N2M1	US-guided core-needle biopsy	Liver	Fresh Frozen	biopsy	metastatic	Tx-naive	0.25	Category I (actionability)	**KRAS Q61L**	**I-3 (Elimination of ineffective treatment options)**		
80	36-P-105	**80**	F	53	Pancreatic cancer	Adenocarcinoma	metastatic	US-guided core-needle biopsy	Abdominal wall	Fresh Frozen	biopsy	metastatic	On treatment	0.28	Category I (actionability)	**KRAS G12C**	**I-2 (Screen for mutation-specific clinical trials)**	**NCT03065387, NCT04892017**	
81	36-P-107	**81**	M	79	Stomach cancer	Adenocarcinoma	cT4aN1M0	EGD	Stomach	FFPE	biopsy	localized	Tx-naive	0.2	Category I (actionability)	**MSI-H**	**I-1 (Selection of targeted therapeutics)**		
82	36-P-109	**82**	M	41	Non-smal cell lung cancer	Adenocarcinoma	cT1bN0M0	RLLobectomy	Lung, RLL	Fresh Frozen	surgery	localized	Tx-naive	0.45	Category I (actionability)		**No**		
83	36-P-111	**83**	M	71	ACUP	Adenocarcinoma	cTxNxM1	US-guided core-needle biopsy	Peritoneum	Fresh Frozen	biopsy	metastatic	On treatment	0.71	Category II (Clarity)		**II-2 (Tumor orgin)**		

Supplementary Table 1. Clinicopathological characteristics and Categories for Clinical Utility of WGS

Order	Screen ID	Case No	Sex	Age	Diagnosis	Tissue type	Staging	Title of surgery or biopsy	Organ of surgery/biopsy	FFPE / Fresh Frozen	Sample_type	Disease status (localized, metastatic)	Time-point (Tx-naive On treatment Relapsed)	Tumor Purity (calculated by WGS pipeline)	Clinical Question	Answer for clinical question or Clinically Significant Alterations	Clinical Utility Category	Related Clinical Trials	Reasons for Screen fail or Q/C fail
84	36-P-112	**84**	F	37	Biliary cancer	combined HCC-CCC	cT4N3M1	US-guided core-needle biopsy	Liver	Fresh Frozen	biopsy	metastatic	Tx-naive	0.59	Category I (actionability)	**FGFR2 amplification**	**I-2 (Screen for mutation-specific clinical trials)**	**NCT03230318, NCT04526106**	
85	36-P-113	**85**	F	51	Breast cancer	Invasive ductal carcinoma (TNBC)	cT2N0M0	Lt. total mastectomy c SLNB	Breast, Lt.	FFPE	surgery	metastatic	relapsed	0.68	Category I (actionability)	**PIK3CA E545K**	**I-2 (Screen for mutation-specific clinical trials)**	**NCT04251533, NCT04216472**	
86	36-P-114	**86**	M	60	Stomach cancer	Adenocarcinoma	cT1N0M0	EMR, ESD	Stomach	FFPE	biopsy	localized	Tx-naive	0.27	Category I (actionability)		**No**		
87	36-P-115	**87**	M	70	Stomach cancer	Adenocarcinoma	cT2N0M0	Total gastrectomy	Stomach	FFPE	surgery	localized	Tx-naive	0.31	Category II (Clarity)		**II-2 (Tumor orgin)**		
88	36-P-117	**88**	F	49	Breast cancer	Invasive ductal carcinoma (TNBC)	cT2N1M0 -> recurred to liver	US-guided core-needle biopsy	Axillar LN, Rt.	FFPE	biopsy	metastatic	relapsed	0.52	Category I (actionability)	**BRIP1 deletion + LOH, HRD+**	**I-2 (Screen for mutation-specific clinical trials)**	**NCT02264678**	
89	36-P-119	**89**	F	58	Breast cancer	Invasive ductal carcinoma (ER+,PR+,HER-2-)	cT3N3M1	US-guided core-needle biopsy	Breast, Lt.	FFPE	biopsy	metastatic	Tx-naive	0.4	Category I (actionability)		**No**		
90	36-P-121	**90**	F	59	Brain tumor	astrocytoma grade 4	Gade 4, IDH wild, EGFR amplified	MR based neuronavigaion guided C/O, Rt. PT & GTR of tumor	Brain, Rt. frontal lobe	Fresh Frozen	surgery	localized	Tx-naive	0.37	Category I (actionability)		**No**		
91	36-P-122	**91**	M	51	Renal cell carcinoma	clear cell carcinoma	cT4N1M1, IMDC intermediate	US-guided core-needle biopsy	Kidney, Lt.	FFPE	biopsy	metastatic	Tx-naive	0.7	Category I (actionability)		**No**		
92	36-P-125	**92**	M	78	Urothelial carcinoma	Infiltrating urothelial carcinoma, high-grade	cT4N1M1	Radical nephrectomy	Renal pelvis, Rt.	FFPE	surgery	metastatic	Tx-naive	0.4	Category I (actionability)		**No**		
93	36-P-126	**93**	M	67	Prostate cancer	Adenocarcinoma, GS (5+5)	cT4N1M1	US-guided core-needle biopsy	Prostate	FFPE	biopsy	metastatic	Tx-naive	0.78	Category I (actionability)	**somatic BRCA2 G177FfsTer4 + LOH, HRD+**	**I-1 (Selection of targeted therapeutics)**		
94	36-P-127	**94**	F	47	Gastrointestinal stromal tumor	Gastrointestinal stromal tumor	rTxNxM1	Rt. hemihepatectomy	Liver, Rt. lobe	Fresh Frozen	surgery	metastatic	relapsed	0.96	Category I (actionability)	**KIT inframe deletion (p.Trp557_Thr574delinsSer)**	**I-1 (Selection of targeted therapeutics)**		
95	36-P-128	**95**	F	50	Colorectal cancer	Adenocarcinoma	cT4aN+M1	Anterior resection	Sigmoid colon	FFPE	surgery	metastatic	Tx-naive	0.62	Category II (Clarity)		**II-3 (Familial cancer)**		
96	36-P-002		M	62	Urothelial carcinoma	squamous cell carcinoma	cT4N0M0-> relapsed to pelvic wall	US-guided core-needle biopsy	Left inguinal lymph nodes	Fresh Frozen	biopsy	metastatic	On treatment	NA					Poor DNA quantity or quality
97	36-P-003		M	54	Stomach cancer	Adenocarcinoma	cT4aN3M0	EGD	Stomach	Fresh Frozen	biopsy	metastatic	On treatment	NA					Poor DNA quantity or quality
98	36-P-005		F	69	Pancreatic cancer	Adenocarcinoma	locally advanced	EUS Bx	Pancreas, Head	Fresh Frozen	biopsy	localized	Tx-naive	NA					Poor DNA quantity or quality
99	36-P-015		M	55	Stomach cancer	Adenocarcinoma	cT4aN2M1, peritoneal seeding	US-guided core-needle biopsy	Abdominal wall	Fresh Frozen	biopsy	metastatic	On treatment	NA					Poor DNA quantity or quality
100	36-P-044		M	63	Stomach cancer	Adenocarcinoma, poorly differentiated	cT4N2M1	EGD	Stomach	Fresh Frozen	biopsy	localized	Tx-naive	NA					Poor DNA quantity or quality
101	36-P-055		M	41	Stomach cancer	Poorly cohesive carcinoma	cT4N1M0 sT4aN1M1P	Diagnostic Intraabdominal Biopsy	Peritoneal nodule	Fresh Frozen	surgery	metastatic	Tx-naive	NA					Poor DNA quantity or quality
102	36-P-062		M	66	Colorectal cancer	Adenocarcinoma	cT3N1M0	Anterior Resection	Rectosigmoid junction	Fresh Frozen	surgery	localized	Tx-naive	NA					Poor DNA quantity or quality
103	36-P-073		M	61	Melanoma	Malignant melanoma	cTxNxM1	US-guided core-needle biopsy	Cervical lymph node, Rt.	Fresh Frozen	biopsy	metastatic	Tx-naive	NA					Poor DNA quantity or quality
104	36-P-093		M	53	Stomach cancer	Adenosquamous carcinoma	cT4N2M0	Total gastrectomy	Stomach	Fresh Frozen	surgery	metastatic	Tx-naive	NA					Poor DNA quantity or quality
105	36-P-108		F	57	Colorectal cancer	Poorly cohesive carcinoma	cT4N1M1	US-guided core-needle biopsy	Liver	Fresh Frozen	biopsy	metastatic	On treatment	NA					Poor DNA quantity or quality
106	36-P-124		F	65	GB cancer	Adenocarcinoma	cTxN2M1	EUS guided biopsy Rt. Excisional biopsy, Rt. tonsillectomy biopsy	Lymph node (around liver hilum) Entire right neck LN	FFPE	biopsy	metastatic	Tx-naive	NA					Poor DNA quantity or quality
107	36-P-006		M	57	Pancreatic cancer	Adenocarcinoma	metastatic	EUS Bx	Pancreas, Tail	Fresh Frozen	biopsy	metastatic	Tx-naive	0.1					Low tumor cell fraction
108	36-P-042		M	66	Pancreatic cancer	Adenocarcinoma	metastatic	EUS Bx	Pancreas, Tail	Fresh Frozen	biopsy	metastatic	Tx-naive	0					Low tumor cell fraction
109	36-P-050		F	60	Stomach cancer	Adenocarcinoma	cT4aN1M0	Total gastrectomy	Stomach	Fresh Frozen	surgery	localized	Tx-naive	0					Low tumor cell fraction
110	36-P-079		M	64	Urothelial carcinoma	Infiltrating urothelial carcinoma, high-grade	pT2N2M0	TURBT	Bladder, Lt. UVJ area	Fresh Frozen	surgery	localized	Tx-naive	0.1					Low tumor cell fraction
111	36-P-080		M	56	Head and Neck cancer	parotid gland cancer	pT4aN3bM0	Rt. Total parotidectomy, Rt. mRND	Parotid gland, Rt.	Fresh Frozen	surgery	localized	Tx-naive	0					Low tumor cell fraction
112	36-P-081		F	44	Stomach cancer	Adenocarcinoma	cT3N1M0	BSO	Ovary	Fresh Frozen	biopsy	metastatic	Tx-naive	0.1					Low tumor cell fraction
113	36-P-082		M	56	Stomach cancer	Adenocarcinoma	cT4N2aM0, sT4N2M1P1	EGD	Stomach	FFPE	biopsy	metastatic	On treatment	0.14					Low tumor cell fraction
114	36-P-085		M	55	Head and Neck cancer	Squamous cell carcinoma	cTxNxM0	Lt. SND	Cervical lymph node, Lt.	FFPE	surgery	localized	Tx-naive	0.05					Low tumor cell fraction
115	36-P-092		F	19	Soft tissue sarcoma	WD-Liposarcoma	cT4N0M0	US-guided core-needle biopsy	Posterior thigh, Rt.	FFPE	biopsy	metastatic	Tx-naive	0.05					Low tumor cell fraction
116	36-P-096		M	39	Stomach cancer	Adenocarcinoma	cT4N2M1, peritoneal meta	Total gastrectomy	Stomach, Peritoneum	Fresh Frozen	surgery	metastatic	Tx-naive	0					Low tumor cell fraction
117	36-P-106		F	54	Soft tissue sarcoma	aggressive fibromatosis	cT3N2M0, sT4N2M1P2	US-guided core-needle biopsy	Posterior neck mass, Rt.	Fresh Frozen	biopsy	metastatic	Tx-naive	0.05					Low tumor cell fraction
118	36-P-116		M	69	Colorectal cancer	Adenocarcinoma	cT4N3M1c	CT-guided core-needle biopsy	Sternum	Fresh Frozen	biopsy	metastatic	On treatment	0.04					Low tumor cell fraction
119	36-P-118		F	42	Stomach cancer	Adenocarcinoma	cT4N3M1, peritoneal meta	Total gastrectomy	Stomach	Fresh Frozen	surgery	metastatic	Tx-naive	0.11					Low tumor cell fraction
120	36-P-120		M	68	small bowel	Adenocarcinoma	cTxNxM1, peritoneal meta	Segmental resection of ileum	Ileum	Fresh Frozen	surgery	metastatic	Tx-naive	0.01					Low tumor cell fraction
S/F	36-P-004		F	63	Non-smal cell lung cancer	Adenocarcinoma	cT2N3M1a	EUS Bx	Pancreas					NA					Failed to get tissue (fail to approach to pancreas mass by EUS)
S/F	36-P-063		F	60	Stomach cancer	Poorly cohesive carcinoma	cT4aN3M0/sT4aN3M1	Open and Close						NA					Failed to get tissue (surgical procedure was changed due to peritoneal seedings)

Supplementary Table 1. Clinicopathological characteristics and Categories for Clinical Utility of WGS

Order	Screen ID	Case No	Sex	Age	Diagnosis	Tissue type	Staging	Title of surgery or biopsy	Organ of surgery/biopsy	FFPE / Fresh Frozen	Sample_type	Disease status (localized, metastatic)	Time-point (Tx-naive On treatment Relapsed)	Tumor Purity (calculated by WGS pipeline)	Clinical Question	Answer for clinical question or Clinically Significant Alterations	Clinical Utility Category	Related Clinical Trials	Reasons for Screen fail or Q/C fail
S/F	36-P-123		M	36	soft tissue sarcoma	DDLPS	cTxNxM1	Percutaneous biopsy	Pleura, Lt.					NA					Failed to get tissue (no residual tumor cells to make new slides for WGS in archival FFPE block)
S/F	36-P-110		M	57	Head and Neck cancer	(submandibular) salivary ductal carcinoma	cTxNxM1	Punch biopsy (tongue)	Tongue, Rt. lateral					NA					Failed to get tissue (too small to get tissues for WGS)
S/F	36-P-032		M	75	Lung cancer	Not available	cT4N3M1a	screening fail						NA					withdrawal to participate (trasfer to other hospital)
S/F	36-P-046		M	67	Stomach cancer	Adenocarcinoma, poorly differentiated	cT4aN1M0	screening fail						NA					withdrawal to participate (trasfer to other hospital)
S/F	36-P-057		F	74	Non-smal cell lung cancer	Squamous cell carcinoma	cT2N3M0	screening fail						NA					withdrawal to participate
S/F	36-P-064		M	40	Prostate cancer	Adenocarcinoma, GS (4+5)	cT3N1M1b	screening fail						NA					withdrawal to participate (trasfer to other hospital)

Supplementary Table 2. List of pathogenic or likely-pathogenic germline variants

Gene	Associated diseases
A2ML1	Cardiomyopathy_t2,Rasopathies_t2,Noonan_syndrome
AAGAB	Dermatology_t2
AARS1	Ataxia_t2,Epilepsy_t2,Charcot-Marie-Tooth_disease_t1,Charcot-Marie-Tooth,Charcot_Marie_Tooth_and_Related
AARS2	Cardiomyopathy_t2
ABAT	Epilepsy_t2
ABCA1	Coagulation_t2,Hereditary_Dyslipidemia_t1,Stroke,Dyslipidemia_and_Related
ABCA12	Dermatology_t1
ABCA2	Epilepsy_t2
ABCA3	Dermatology_t2
ABCA4	Hereditary_retinopathy_t2,Retinitis_pigmentosa_t1,Macular_dystrophy,Retinitis_pigmentosa,Cone-Rod_Dystrophy_and_Related,Macular_dystrophy_and_Related,Retinitis_Pigmentosa_and_Related,retina
ABCA7	Dementia_t2,Alzheimer's_disease_t1,Neurodegenerative_disease_t1
ABCB11	Cholestasis_t1,Neonatal_cholestasis
ABCB4	Cholestasis_t1,Neonatal_cholestasis
ABCB6	Dermatology_t2,Hereditary_retinopathy_t2,Anemia_t2,Anemia
ABCB7	Ataxia_t2,Anemia_t1,Anemia
ABCC2	Cholestasis_t1,Neonatal_cholestasis
ABCC6	Cardiomyopathy_t2,Dermatology_t2,Skeletal_dysplasia_t2,Connective_tissue_disorder_t2,Hereditary_retinopathy_t2,Hereditary_Stroke_t1,Stroke,retina
ABCC8	Lysosomal_storage_disease_t2,Epilepsy_t2,Polycystic_Kidney_Disease_t2,Maturity-Onset_Diabetes_of_the_Young,_MODY_t1,Monogenic_diabetes_panel
ABCC9	Cardiomyopathy_t2,Arrhythmia_t1,Dilated_cardiomyopathy,Arrhythmia_and_Related
ABCD1	Ataxia_t2,Inborn_error_of_metabolism_t2,Epilepsy_t2,Hereditary_Autism_t2,Neurodegenerative_disease_t2,Amyotrophic_lateral_sclerosis_t2,Charcot-Marie-Tooth_disease_t1,Hereditary_spastic_paraplegia_t1,Lysosomal_storage_disease_t1,Ataxia,Charcot_Marie_Tooth_and_Related,Spastic_Paraplegia_and_Related
ABCG5	Coagulation_t2,Anemia_t2,Hereditary_Dyslipidemia_t1,Anemia,Bleeding_Platelet_Disorder,Dyslipidemia_and_Related
ABCG8	Coagulation_t2,Hereditary_Dyslipidemia_t1,Anemia,Bleeding_Platelet_Disorder,Dyslipidemia_and_Related
ABHD12	Ataxia_t2,Hearing_loss_t2,Hereditary_retinopathy_t2,Neurodegenerative_disease_t2,Amyotrophic_lateral_sclerosis_t2,Retinitis_pigmentosa_t1,Charcot-Marie-Tooth,Charcot_Marie_Tooth_and_Related,Retinitis_Pigmentosa_and_Related,retina
ABHD5	Dermatology_t2
ABL1	Connective_tissue_disorder_t2,Cell-free_cancer,Hematologic_malignancy,ALL_and_others_t1,ALL_and_others_t2,AML,Lymphoma_T_NK_cell_t1,Lymphoma_T_NK_cell_t2,MDS_MPN
ABL2	ALL_and_others_t2
ABRAXAS1	Hereditary_pancancer
ACAD8	Inborn_error_of_metabolism_t2,Inborn_Error_of_Metabolism
ACAD9	Cardiomyopathy_t2,Myopathy_t2
ACADL	Myopathy_t2
ACADM	Myopathy_t2,Inborn_error_of_metabolism_t1,Inborn_Error_of_Metabolism
ACADS	Inborn_error_of_metabolism_t2,Inborn_Error_of_Metabolism
ACADSB	Inborn_error_of_metabolism_t2
ACADVL	Cardiomyopathy_t2,Myopathy_t2,Inborn_error_of_metabolism_t1,Metabolic_myopathy
ACAN	Skeletal_dysplasia_t2,Skeletal_Dysplasia_Panel
ACAT1	Hereditary_Autism_t2,Inborn_error_of_metabolism_t1
ACBD5	Hereditary_retinopathy_t2
ACD	Congenital_hematologic_disease,Dyskeratosis_congenita
ACE	Stroke

Supplementary Table 2. List of pathogenic or likely-pathogenic germline variants

Gene	Associated diseases
ACHE	Hemolytic_anemia
ACO2	Ataxia_t2,Hereditary_retinopathy_t2,Optic_neuropathy_and_Related,retina
ACOX1	Hereditary_spastic_paraplegia_t2,Lysosomal_storage_disease_t2,Hereditary_Autism_t2,Neurodegenerative_disease_t2
ACP5	Skeletal_dysplasia_t2,Skeletal_Dysplasia_Panel
ACSL4	Hereditary_Autism_t2
ACTA1	Cardiomyopathy_t2,Muscular_dystrophy_t2,Myopathy_t1,Congenital_muscular_dystrophy,Congenital_myopathy,Myofibrillar_myopathy,Myopathy_and_Related
ACTA2	Hereditary_Stroke_t2,Connective_tissue_disorder_t1,Familial_thoracic_aortic_aneurysms_and_aortic_dissections,Moyamoya_disease,Stroke,Connective_Tissue_Disorder_and_Related,Marfan_and_Related
ACTB	Skeletal_dysplasia_t2,Proportionate_short_stature_t2,Rasopathies_t2,Hearing_loss_t2,Hereditary_Autism_t2,Hemolytic_anemia,Malformation_of_cortical_development,Noonan_syndrome
ACTC1	Cardiomyopathy_t1,Dilated_cardiomyopathy,Hypertrophic_cardiomyopathy,Cardiomyopathy_and_Related
ACTG1	Skeletal_dysplasia_t2,Proportionate_short_stature_t2,Rasopathies_t2,Hearing_loss_t2,Hereditary_Autism_t2,Hereditary_hearing_loss,Malformation_of_cortical_development,Noonan_syndrome,Hearing_Loss_and_Related_t2
ACTL6B	Epilepsy_t2
ACTN1	Coagulation_t2,Bleeding_Platelet_Disorder
ACTN2	Arrhythmia_t2,Cardiomyopathy_t1,Dilated_cardiomyopathy,Hypertrophic_cardiomyopathy,Cardiomyopathy_and_Related
ACTN4	Glomerulopathy
ACVR1	Skeletal_dysplasia_t2,Anemia
ACVRL1	Primary_Pulmonary_Hypertension_and_Related_Disordisorders
ACY1	Lysosomal_storage_disease_t2,Epilepsy_t2,Hereditary_Autism_t2
ADA	Anemia_t2,Primary_immune_deficiency_t1,Inflammatory_Bowel_t1,Familial_hemophagocytic_lymphohistiocytosis,Hemolytic_anemia,Primary_immunodeficiency,Very-early-onset_inflammatory_bowel_disease,Anemia,Hemophagocytic_Lymphohistiocytosis_and_Related,Severe_Combined_Immunodeficiency_and_CID
ADA2	Cardiomyopathy_t2,Hereditary_Stroke_t1,Stroke
ADAM17	Inflammatory_Bowel_t2,Very-early-onset_inflammatory_bowel_disease,Autoimmunity_and_autoinflammatory_disorders,B_cell_and_Humoral_Immune_Deficiency
ADAM22	Epilepsy_t2
ADAM9	Hereditary_retinopathy_t2,Macular_dystrophy,Cone-Rod_Dystrophy_and_Related,Retinitis_Pigmentosa_and_Related,retina,glaucoma
ADAMTS10	Skeletal_dysplasia_t2,Proportionate_short_stature_t2,Connective_tissue_disorder_t1,Skeletal_Dysplasia_Panel,Connective_Tissue_Disorder_and_Related
ADAMTS13	Coagulation_t2,Atypical_Hemolytic_Uremic_Syndrome_t1,DNAJC21_EFL1_SBDS_SRP54,Atypical_Hemolytic_Uremic_Syndrome,Bleeding_Coagulopathy,Bleeding_Platelet_Disorder,Thrombosis
ADAMTS17	Skeletal_dysplasia_t2,Connective_tissue_disorder_t2,Skeletal_Dysplasia_Panel,corneal_keratoconus
ADAMTS18	Hereditary_retinopathy_t2,Cone-Rod_Dystrophy_and_Related,retina
ADAMTS2	Dermatology_t2,Connective_tissue_disorder_t2
ADAMTS8	corneal_keratoconus
ADAMTSL1	glaucoma
ADAMTSL2	Skeletal_dysplasia_t2,Proportionate_short_stature_t2,Lysosomal_storage_disease_t2,Skeletal_Dysplasia_Panel
ADAMTSL4	Hereditary_retinopathy_t2,Connective_tissue_disorder_t1,Connective_Tissue_Disorder_and_Related
ADAR	Dermatology_t2,Epilepsy_t2,Dystonia_t2,Parkinson's_disease_t2,Hereditary_Autism_t2
ADCY1	Hearing_loss_t2,Hereditary_hearing_loss
ADCY5	Parkinson's_disease_t2,Neurodegenerative_disease_t2,Dystonia_t1,Chorea,Dyskinesia-dystonia-paralysis,Dystonia,Paroxysmal_movement_disorder,Dystonia_and_Related
ADD2	Hemolytic_anemia
ADGRA3	Hereditary_retinopathy_t2,Retinitis_pigmentosa_t1,Retinitis_pigmentosa,Retinitis_Pigmentosa_and_Related,retina
ADGRG1	Epilepsy_t2
ADGRV1	Epilepsy_t2,Hearing_loss_t2,Hereditary_retinopathy_t1,Hereditary_hearing_loss,Hearing_Loss_and_Related_t1,Hearing_Loss_and_Related_t2,Retinitis_Pigmentosa_and_Related,retina
ADIPOR1	Retinitis_pigmentosa_t2,Hereditary_retinopathy_t2,Retinitis_pigmentosa,Retinitis_Pigmentosa_and_Related,retina
ADNP	Hereditary_Autism_t2,Autism_and_Related

Supplementary Table 2. List of pathogenic or likely-pathogenic germline variants

Gene	Associated diseases
ADPRS	Ataxia_t2,Epilepsy_t2
ADRA2B	Epilepsy_t2
ADSL	Lysosomal_storage_disease_t2,Epilepsy_t2,Hereditary_Autism_t2,Epilepsy_and_Related
AFF2	Hereditary_Autism_t2
AFF4	Skeletal_dysplasia_t2
AFG3L2	Ataxia_t2,Hereditary_spastic_paraplegia_t2,Epilepsy_t2,Hereditary_retinopathy_t2,Dystonia_t2,Parkinson's_disease_t2,Neurodegenerative_disease_t2,Ataxia_and_Related,Optic_neuropathy_and_Related
AGA	Lysosomal_storage_disease_t2,Epilepsy_t2,Skeletal_Dysplasia_Panel
AGBL1	Hereditary_retinopathy_t2,Corneal_Dystrophy_and_Related,corneal_dystrophy
AGBL5	Hereditary_retinopathy_t2,Retinitis_pigmentosa_t1,Retinitis_pigmentosa,Retinitis_Pigmentosa_and_Related,retina
AGK	Cardiomyopathy_t2,Hereditary_retinopathy_t2
AGL	Cardiomyopathy_t2,Myopathy_t2,Lysosomal_storage_disease_t1,Glycogen_storage_disease,Metabolic_myopathy,Lysosomal_Storage_Disease
AGPAT2	Dermatology_t2
AGPS	Skeletal_dysplasia_t2,Skeletal_Dysplasia_Panel,Spondyloepiphyseal_metaphyseal_dysplasia
AGRN	Congenital_myopathy
AGT	Stroke
AGTPBP1	Charcot-Marie-Tooth_disease_t2,Ataxia_t2
AGTR2	Hereditary_Autism_t2
AGXT	Ca-Pi-Mg
AHCY	Myopathy_t2,Inborn_error_of_metabolism_t2
AHDC1	Hereditary_Autism_t2
AHI1	Ataxia_t2,Epilepsy_t2,Retinitis_pigmentosa_t2,Hereditary_Autism_t2,Hereditary_retinopathy_t1,Nephronophthisis_t1,Nephronophthisis_and_Related,Retinitis_Pigmentosa_and_Related,retina
AHNAK2	Charcot_Marie_Tooth_and_Related
AHR	Retinitis_pigmentosa,retina
AICDA	Primary_immune_deficiency_t1,Inflammatory_Bowel_t1,Primary_immunodeficiency,Very-early-onset_inflammatory_bowel_disease,B_cell_and_Humoral_Immune_Deficiency
AIFM1	Skeletal_dysplasia_t2,Ataxia_t2,Epilepsy_t2,Hearing_loss_t2,Hereditary_Autism_t2,Charcot-Marie-Tooth_disease_t1,Charcot-Marie-Tooth,Hereditary_hearing_loss,Skeletal_Dysplasia_Panel,Charcot_Marie_Tooth_and_Related
AIMP1	Epilepsy_t2
AIP	Hereditary_pancancer
AIPL1	Hereditary_retinopathy_t2,Retinitis_pigmentosa_t1,Leber_congenital_amaurosis,Macular_dystrophy,Cone-Rod_Dystrophy_and_Related,Leber's_Congenital_Amaurosis_and_Related,Retinitis_Pigmentosa_and_Related,retina
AIRE	Primary_immune_deficiency_t2,Rickets_Hypoparathyroidism_panel,Autoimmunity_and_autoinflammatory_disorders,B_cell_and_Humoral_Immune_Deficiency,Hemophagocytic_Lymphohistiocytosis_and_Related,Phagocyte_Defect_and_Infectious_disease
AK1	Anemia_t1,Hemolytic_anemia,Anemia
AK2	Dermatology_t2,Primary_immune_deficiency_t2,Severe_Combined_Immunodeficiency_and_CID
AKAP9	Cardiomyopathy_t2,Arrhythmia_t1,Arrhythmia,Arrhythmia_and_Related
AKR1C2	Disorders_of_sexual_development_t2,Disorders_of_sex_development
AKR1C4	Disorders_of_sexual_development_t2,Disorders_of_sex_development
AKR1D1	Cholestasis_t1,Neonatal_cholestasis
AKT1	Skeletal_dysplasia_t2,Cell-free_cancer,Hereditary_breast_cancer,Overgrowth_Panel,ALL_and_others_t2,Breast_and_Ovarian_Cancer_Panel,Circulating_tumor_DNA_assay_55,Lymphoma_B_cell_t2,Myeloma_t1
AKT2	Cell-free_cancer,Monogenic_diabetes_panel,Lymphoma_B_cell_t2,Lymphoma_T_NK_cell_t2
AKT3	Epilepsy_t2,Hereditary_Microcephaly_t2,Focal_epilepsy,Malformation_of_cortical_development,Overgrowth_intellectual_disability,Lymphoma_B_cell_t2,Lymphoma_T_NK_cell_t2

Supplementary Table 2. List of pathogenic or likely-pathogenic germline variants

Gene	Associated diseases
ALAD	Dermatology_t2
ALAS2	Dermatology_t2,Anemia_t1,Anemia,Hemochromatosis
ALDH18A1	Dermatology_t2,Connective_tissue_disorder_t2,Hereditary_spastic_paraplegia_t2,Hereditary_Autism_t2,Neurodegenerative_disease_t2
ALDH1A3	Hereditary_retinopathy_t2
ALDH3A2	Dermatology_t2,Ataxia_t2,Epilepsy_t2
ALDH4A1	Lysosomal_storage_disease_t2,Epilepsy_t2,Hereditary_Autism_t2
ALDH5A1	Ataxia_t2,Lysosomal_storage_disease_t2,Epilepsy_t2,Hereditary_Autism_t2
ALDH7A1	Lysosomal_storage_disease_t2,Epilepsy_t1,Epilepsy_and_Related
ALDOA	Myopathy_t2,Lysosomal_storage_disease_t2,Anemia_t2,Hemolytic_anemia,Metabolic_myopathy,Anemia
ALDOB	Lysosomal_storage_disease_t1,Hemolytic_anemia
ALG1	Epilepsy_t2,Hereditary_Autism_t2
ALG11	Hereditary_Autism_t2
ALG12	Epilepsy_t2,Hereditary_Autism_t2
ALG13	Epilepsy_t2,Hereditary_Autism_t2,Congenital_muscular_dystrophy,Epilepsy_and_Related
ALG2	Epilepsy_t2,Hereditary_Autism_t2
ALG3	Epilepsy_t2,Hereditary_Autism_t2
ALG6	Epilepsy_t2,Hereditary_Autism_t2
ALG8	Epilepsy_t2,Polycystic_Kidney_Disease_t1,Polycystic_kidney_disease_and_Related
ALG9	Epilepsy_t2
ALK	Lymphoma_t1,Cell-free_cancer,Hereditary_pancancer,Circulating_tumor_DNA_assay_55,Germline_Cancer,Lymphoma_B_cell_t1,Lymphoma_B_cell_t2,Lymphoma_T_NK_cell_t1,Lymphoma_T_NK_cell_t2
ALKBH8	Epilepsy_t2
ALMS1	Cardiomyopathy_t2,Hearing_loss_t2,Hereditary_retinopathy_t2,Hereditary_Dyslipidemia_t1,Dyslipidemia_and_Related,Leber's_Congenital_Amaurosis_and_Related,Retinitis_Pigmentosa_and_Related,retina
ALOX12B	Dermatology_t1
ALOXE3	Dermatology_t1
ALPK3	Cardiomyopathy_t2
ALPL	Skeletal_dysplasia_t1,Osteogenesis_imperfecta,Skeletal_Dysplasia_Panel,Skeletal_Dysplasia_and_Related
ALS2	Ataxia_t2,Hereditary_spastic_paraplegia_t2,Neurodegenerative_disease_t2,Amyotrophic_lateral_sclerosis_t1,Amyotrophic_lateral_sclerosis
ALX3	Skeletal_dysplasia_t2
ALX4	Skeletal_dysplasia_t2,Hereditary_Autism_t2
AMACR	Charcot-Marie-Tooth_disease_t2,Epilepsy_t2,Hereditary_retinopathy_t2,Neonatal_cholestasis
AMER1	Skeletal_dysplasia_t2,Hereditary_Autism_t2,Sclerosing_Bone_Disease_Panel
AMH	Disorders_of_sexual_development_t1,Disorders_of_sex_development
AMHR2	Disorders_of_sexual_development_t1,Disorders_of_sex_development
AMMECR1	Skeletal_dysplasia_t2,Proportionate_short_stature_t2,Hearing_loss_t2
AMN	Anemia
AMPD1	Myopathy_t2
AMPD2	Hereditary_spastic_paraplegia_t2,Hereditary_Microcephaly_t2,Neurodegenerative_disease_t2
AMT	Lysosomal_storage_disease_t2,Epilepsy_t2,Hereditary_Autism_t2,Inborn_Error_of_Metabolism
ANAPC1	corneal_keratoconus
ANG	Parkinson's_disease_t1,Neurodegenerative_disease_t1,Amyotrophic_lateral_sclerosis_t1,Amyotrophic_lateral_sclerosis
ANGPT1	glaucoma

Supplementary Table 2. List of pathogenic or likely-pathogenic germline variants

Gene	Associated diseases
ANGPTL3	Hereditary_Dyslipidemia_t1,Dyslipidemia_and_Related
ANK1	Anemia_t1,Hemolytic_anemia,Anemia
ANK2	Cardiomyopathy_t2,Arrhythmia_t1,Arrhythmia,Arrhythmia_and_Related
ANK3	Epilepsy_t2,Hereditary_Autism_t2
ANKH	Skeletal_dysplasia_t2,Hearing_loss_t2,Sclerosing_Bone_Disease_Panel,Skeletal_Dysplasia_Panel
ANKLE2	Hereditary_Microcephaly_t2
ANKRD1	Arrhythmia_t2,Cardiomyopathy_t1,Dilated_cardiomyopathy,Hypertrophic_cardiomyopathy,Cardiomyopathy_and_Related
ANKRD11	Skeletal_dysplasia_t2,Epilepsy_t2,Hereditary_Autism_t2
ANKRD26	Coagulation_t2,Congenital_hematologic_disease,Hematologic_malignancy,Leukemia_predisposition,ALL_and_others_t1,ALL_and_others_t2,AML,Bleeding_Platelet_Disorder,Bone_Marrow_Failure_Germline_Predisposition,Hereditary_Myeloid_Leukemia_Panel,MDS_MPN
ANKS6	Nephronophthisis_t1,Polycystic_Kidney_Disease_t1,Nephronophthisis_and_Related,Polycystic_kidney_disease_and_Related
ANLN	Glomerulopathy
ANO10	Ataxia_t1,Ataxia,Ataxia_and_Related
ANO3	Parkinson's_disease_t2,Neurodegenerative_disease_t2,Dystonia_t1,Dyskinesia-dystonia-paralysis,Dystonia,Dystonia_and_Related
ANO5	Cardiomyopathy_t2,Skeletal_dysplasia_t2,Myopathy_t2,Muscular_dystrophy_t1,Limb_girdle_muscular_dystrophy,Osteogenesis_imperfecta,Muscular_Dystrophy_and_Related
ANO6	Coagulation_t2,Bleeding_Platelet_Disorder
ANOS1	Disorders_of_sexual_development_t1,Hypogonadotropic_hypogonadism_t1,Isolated_Hypogonadotropic_Hypogonadism
ANTXR1	Hereditary_retinopathy_t2
ANTXR2	Lysosomal_storage_disease_t2,Skeletal_Dysplasia_Panel
AP1S1	Dermatology_t2,Hereditary_Autism_t2
AP1S2	Hereditary_Autism_t2
AP2M1	Epilepsy_t2
AP2S1	Skeletal_dysplasia_t2,Atypical_Hemolytic_Uremic_Syndrome_t2,Rickets_Hypoparathyroidism_panel
AP3B1	Dermatology_t2,Lysosomal_storage_disease_t2,Coagulation_t2,Hereditary_retinopathy_t2,Hereditary_Autism_t2,Familial_hemophagocytic_lymphohistiocytosis,Albinism_and_Related,Bleeding_Platelet_Disorder,Bone_Marrow_Failure_Germline_Predisposition,Hemophagocytic_Lymphohistiocytosis_and_Related
AP3B2	Epilepsy_t2
AP3D1	Dermatology_t2
AP4B1	Ataxia_t2,Hereditary_spastic_paraplegia_t2,Epilepsy_t2,Hereditary_Autism_t2,Neurodegenerative_disease_t2
AP4E1	Ataxia_t2,Epilepsy_t2,Hereditary_Autism_t2,Neurodegenerative_disease_t2,Hereditary_spastic_paraplegia_t1,Spastic_Paraplegia_and_Related
AP4M1	Ataxia_t2,Epilepsy_t2,Hereditary_Autism_t2,Neurodegenerative_disease_t2,Hereditary_spastic_paraplegia_t1,Hereditary_Microcephaly_t1,Spastic_Paraplegia_and_Related
AP4S1	Ataxia_t2,Epilepsy_t2,Hereditary_Autism_t2,Neurodegenerative_disease_t2,Hereditary_spastic_paraplegia_t1,Spastic_Paraplegia_and_Related
AP5Z1	Ataxia_t2,Hereditary_spastic_paraplegia_t2,Neurodegenerative_disease_t2
APC	Hereditary_cancer_syndrome_Plus_t1,Cell-free_cancer,Hematologic_malignancy,Hereditary_colon_cancer,Hereditary_pancancer,Circulating_tumor_DNA_assay_55,Colorectal_Cancer_Polyposis_Panel,Germline_Cancer
APC2	Overgrowth_Panel
APCDD1	Dermatology_t2
APOA1	Cardiomyopathy_t2,Hereditary_Dyslipidemia_t1,Stroke,Dyslipidemia_and_Related
APOA2	Hereditary_Dyslipidemia_t1
APOA5	Hereditary_Dyslipidemia_t1,Dyslipidemia_and_Related
APOB	Hereditary_Stroke_t2,Hereditary_Dyslipidemia_t1,Stroke,Dyslipidemia_and_Related
APOC2	Hereditary_Dyslipidemia_t1,Dyslipidemia_and_Related
APOC3	Hereditary_Dyslipidemia_t1,Dyslipidemia_and_Related

Supplementary Table 2. List of pathogenic or likely-pathogenic germline variants

Gene	Associated diseases
APOE	Alzheimer's_disease_t2,Dementia_t2,Parkinson's_disease_t2,Hereditary_Stroke_t2,Neurodegenerative_disease_t2,Hereditary_Dyslipidemia_t1,Dyslipidemia_and_Related
APP	Dementia_t2,Parkinson's_disease_t2,Amyotrophic_lateral_sclerosis_t2,Alzheimer's_disease_t1,Hereditary_Stroke_t1,Neurodegenerative_disease_t1,Stroke,Dementia_and_Related
APPL1	Maturity-Onset_Diabetes_of_the_Young,_MODY_t1
APTX	Dystonia_t2,Parkinson's_disease_t2,Ataxia_t1,Ataxia,Chorea,Dystonia,Ataxia_and_Related
AQP1	Hemolytic_anemia,Primary_Pulmonary_Hypertension_and_Related_Disordisorders
AQP2	Acid-base-electro_gene
AQP5	Dermatology_t2
AR	Hereditary_Autism_t2,Disorders_of_sexual_development_t1,Androgenetic_alopecia,Cell-free_cancer,Disorders_of_sex_development,Circulating_tumor_DNA_assay_55
ARAF	Cell-free_cancer,Circulating_tumor_DNA_assay_55,Lymphoma_B_cell_t2
ARCN1	Skeletal_dysplasia_t2,Proportionate_short_stature_t2
ARFGEF2	Epilepsy_t2,Hereditary_Microcephaly_t2,Malformation_of_cortical_development
ARG1	Hereditary_spastic_paraplegia_t2,Lysosomal_storage_disease_t2,Inborn_error_of_metabolism_t2,Epilepsy_t2,Hereditary_Autism_t2,Neurodegenerative_disease_t2,Urea_cycle_disorders
ARHGAP31	Dermatology_t2,Skeletal_dysplasia_t2
ARHGEF10	Charcot-Marie-Tooth_disease_t2,Charcot-Marie-Tooth,Charcot_Marie_Tooth_and_Related
ARHGEF15	Epilepsy_t2
ARHGEF18	Hereditary_retinopathy_t2,Retinitis_pigmentosa_t1,Retinitis_pigmentosa,Cone-Rod_Dystrophy_and_Related,Retinitis_Pigmentosa_and_Related,retina
ARHGEF28	Neurodegenerative_disease_t2,Amyotrophic_lateral_sclerosis_t2
ARHGEF6	Hereditary_Autism_t2
ARHGEF9	Hereditary_Autism_t2,Epilepsy_t1,Early_onset_epilepsy
ARID1A	Skeletal_dysplasia_t2,Hereditary_Autism_t2,Cell-free_cancer,Lymphoma_B_cell_t1,Lymphoma_B_cell_t2,Lymphoma_T_NK_cell_t1,Lymphoma_T_NK_cell_t2,Myeloma_t1
ARID1B	Skeletal_dysplasia_t2,Epilepsy_t2,Hereditary_Autism_t2,Hematologic_malignancy,Autism_and_Related
ARID2	Hematologic_malignancy
ARL13B	Ataxia_t2,Epilepsy_t2,Hereditary_retinopathy_t2
ARL2BP	Hereditary_retinopathy_t2,Retinitis_pigmentosa_t1,Retinitis_pigmentosa,Retinitis_Pigmentosa_and_Related,retina
ARL3	Hereditary_retinopathy_t2,Retinitis_pigmentosa_t1,Retinitis_pigmentosa,Retinitis_Pigmentosa_and_Related,retina
ARL6	Ataxia_t2,Disorders_of_sexual_development_t2,Hereditary_retinopathy_t2,Hypogonadotropic_hypogonadism_t2,Retinitis_pigmentosa_t1,Retinitis_pigmentosa,Retinitis_Pigmentosa_and_Related,retina
ARL6IP1	Hereditary_spastic_paraplegia_t2,Neurodegenerative_disease_t2
ARMC9	Hereditary_retinopathy_t2
ARPC1B	Coagulation_t2,Bleeding_Platelet_Disorder
ARSA	Ataxia_t2,Inborn_error_of_metabolism_t2,Epilepsy_t2,Dystonia_t2,Parkinson's_disease_t2,Neurodegenerative_disease_t2,Charcot-Marie-Tooth_disease_t1,Hereditary_spastic_paraplegia_t1,Lysosomal_storage_disease_t1,Charcot_Marie_Tooth_and_Related,Lysosomal_Storage_Disease,Spastic_Paraplegia_and_Related
ARSB	Skeletal_dysplasia_t2,Epilepsy_t2,Lysosomal_storage_disease_t1,Mucopolysaccharidosis,Skeletal_Dysplasia_Panel,Lysosomal_Storage_Disease
ARSG	Hearing_loss_t2,Hereditary_retinopathy_t2
ARSI	Hereditary_spastic_paraplegia_t2,Neurodegenerative_disease_t2
ARSL	Dermatology_t2,Skeletal_dysplasia_t1,Skeletal_Dysplasia_Panel,Spondyloepiphyseal_metaphyseal_dysplasia,Skeletal_Dysplasia_and_Related
ARX	Disorders_of_sexual_development_t2,Hereditary_Autism_t2,Epilepsy_t1,Disorders_of_sex_development,Malformation_of_cortical_development,Epilepsy_and_Related
ASAH1	Ataxia_t2,Lysosomal_storage_disease_t2,Epilepsy_t2,Hearing_loss_t2,Hereditary_Autism_t2
ASL	Inborn_error_of_metabolism_t1,Urea_cycle_disorders
ASNS	Epilepsy_t2
ASPA	Lysosomal_storage_disease_t2,Epilepsy_t2
ASPM	Epilepsy_t2,Hereditary_Autism_t2,Hereditary_Microcephaly_t1
ASS1	Hereditary_Autism_t2,Inborn_error_of_metabolism_t1,Urea_cycle_disorders,Inborn_Error_of_Metabolism

Supplementary Table 2. List of pathogenic or likely-pathogenic germline variants

Gene	Associated diseases
ASXL1	Hereditary_Microcephaly_t2,Hereditary_Autism_t2,Hematologic_malignancy,ALL_and_others_t1,ALL_and_others_t2,AML,Hereditary_Myeloid_Leukemia_Panel,MDS_MPN
ASXL3	Epilepsy_t2,Hereditary_Microcephaly_t2,Hereditary_Autism_t2,Autism_and_Related,Lymphoma_T_NK_cell_t1,Lymphoma_T_NK_cell_t2
ATAD3A	Charcot-Marie-Tooth_disease_t2,Hereditary_spastic_paraplegia_t2,Hereditary_retinopathy_t2,Neurodegenerative_disease_t2
ATCAY	Ataxia_t2
ATF6	Hereditary_retinopathy_t2,Macular_dystrophy,Achromatopsia_and_Related,retina
ATG5	Lymphoma_T_NK_cell_t1,Lymphoma_T_NK_cell_t2
ATIC	Epilepsy_t2
ATL1	Charcot-Marie-Tooth_disease_t2,Ataxia_t2,Hereditary_Autism_t2,Neurodegenerative_disease_t2,Amyotrophic_lateral_sclerosis_t2,Hereditary_spastic_paraplegia_t1,Ataxia,Hereditary_spastic_paraplegia_panel,Charcot_Marie_Tooth_and_Related,Spastic_Paraplegia_and_Related
ATL3	Charcot-Marie-Tooth_disease_t2,Charcot_Marie_Tooth_and_Related
ATM	Dermatology_t2,Primary_immune_deficiency_t2,Dystonia_t2,Parkinson's_disease_t2,Ataxia_t1,Hereditary_cancer_syndrome_Plus_t1,Lymphoma_t1,Ataxia,Cell-free_cancer,Chorea,Congenital_hematologic_disease,Dystonia,Hematologic_malignancy,Hereditary_breast_cancer,Hereditary_colon_cancer,Hereditary_pancancer,Leukemia_predisposition,Primary_immunodeficiency,ALL_and_others_t2,Ataxia_and_Related,Bone_Marrow_Failure_Germline_Predisposition,Breast_and_Ovarian_Cancer_Panel,Hereditary_Myeloid_Leukemia_Panel,Lymphoma_B_cell_t1,Lymphoma_B_cell_t2,Lymphoma_T_NK_cell_t1,Lymphoma_T_NK_cell_t2,Myeloma_t1,Severe_Combined_Immunodeficiency_and_CID
ATN1	Chorea
ATOH7	Hereditary_retinopathy_t2,Vitreoretinopathy_and_Related
ATP10A	Hereditary_Autism_t2
ATP11C	Hemolytic_anemia,Anemia
ATP13A2	Hereditary_spastic_paraplegia_t2,Lysosomal_storage_disease_t2,Epilepsy_t2,Hereditary_retinopathy_t2,Alzheimer's_disease_t2,Dystonia_t2,Dementia_t2,Hereditary_Autism_t2,Parkinson's_disease_t1,Neurodegenerative_disease_t1,Dystonia,Neurodegeneration_with_brain_iron_accumulation,Parkinson,Parkinson_Disease_and_Related
ATP13A3	Primary_Pulmonary_Hypertension_and_Related_Disordisorders
ATP1A1	Charcot-Marie-Tooth_disease_t2,Charcot-Marie-Tooth,Charcot_Marie_Tooth_and_Related
ATP1A2	Epilepsy_t2,Hereditary_Autism_t2,Hereditary_Stroke_t1,Early_onset_epilepsy
ATP1A3	Ataxia_t2,Epilepsy_t2,Alzheimer's_disease_t2,Dementia_t2,Hereditary_Stroke_t2,Dystonia_t1,Parkinson's_disease_t1,Neurodegenerative_disease_t1,Ataxia,Dyskinesia-dystonia-paralysis,Dystonia,Parkinson,Paroxysmal_movement_disorder,Dystonia_and_Related,Parkinson_Disease_and_Related
ATP1B1	corneal_dystrophy
ATP2A1	Muscular_dystrophy_t2,Myopathy_t2
ATP2A2	Dermatology_t2,Epilepsy_t2
ATP2B2	Hearing_loss_t2,Hereditary_hearing_loss
ATP2B3	Ataxia_t2
ATP2B4	Hereditary_spastic_paraplegia_t2,Neurodegenerative_disease_t2
ATP2C1	Dermatology_t2
ATP4A	Anemia,Hemochromatosis
ATP6AP2	Epilepsy_t2,Dystonia_t2,Parkinson's_disease_t2,Hereditary_Autism_t2
ATP6V0A2	Dermatology_t2,Skeletal_dysplasia_t2,Epilepsy_t2,Connective_tissue_disorder_t1,Osteogenesis_imperfecta,Connective_Tissue_Disorder_and_Related
ATP6V0A4	Skeletal_dysplasia_t2,Atypical_Hemolytic_Uremic_Syndrome_t2,Acid-base-electro_gene,Renal_Tubular_Disorders
ATP6V1A	Epilepsy_t2
ATP6V1B1	Hearing_loss_t2,Atypical_Hemolytic_Uremic_Syndrome_t2,Acid-base-electro_gene,Renal_Tubular_Disorders
ATP6V1B2	Hearing_loss_t2
ATP6V1E1	Connective_tissue_disorder_t2
ATP7A	Dermatology_t2,Connective_tissue_disorder_t2,Charcot-Marie-Tooth_disease_t2,Ataxia_t2,Lysosomal_storage_disease_t2,Hereditary_Autism_t2,Charcot-Marie-Tooth,Charcot_Marie_Tooth_and_Related

Supplementary Table 2. List of pathogenic or likely-pathogenic germline variants

Gene	Associated diseases
ATP7B	Ataxia_t2,Lysosomal_storage_disease_t2,Dementia_t2,Parkinson's_disease_t2,Neurodegenerative_disease_t2,Dystonia_t1,Dystonia,Parkinson,Anemia,Inborn_Error_of_Metabolism
ATP8A2	Ataxia_t2
ATP8B1	Cholestasis_t1,Neonatal_cholestasis
ATPAF2	Epilepsy_t2
ATR	Skeletal_dysplasia_t2,Proportionate_short_stature_t2,Hereditary_Microcephaly_t2,Hereditary_pancancer,Myeloma_t1
ATRIP	Proportionate_short_stature_t2,Hereditary_Microcephaly_t2
ATRX	Disorders_of_sexual_development_t2,Epilepsy_t2,Hereditary_Microcephaly_t2,Hereditary_Autism_t2,Hereditary_cancer_syndrome_Plus_t2,Neuroendocrine_tumor_t1,Disorders_of_sex_development,Hematologic_malignancy,Hereditary_paraganglioma-pheochromocytoma_syndrome
ATXN2	Amyotrophic_lateral_sclerosis
ATXN7	retina
AUH	Inborn_error_of_metabolism_t2,Epilepsy_t2,Hereditary_retinopathy_t2,Hereditary_Autism_t2,Optic_neuropathy_and_Related
AUTS2	Hereditary_Autism_t2,Androgenetic_alopecia
AVPR1A	Hereditary_Autism_t1
AVPR2	Hereditary_Autism_t2,Acid-base-electro_gene
AXIN2	Hereditary_colon_cancer,Hereditary_pancancer
AXL	Hypogonadotropic_hypogonadism_t2
B2M	Primary_immune_deficiency_t2,Lymphoma_t1,Hematologic_malignancy,Hemochromatosis,Lymphoma_B_cell_t1,Lymphoma_B_cell_t2,Lymphoma_T_NK_cell_t2
B3GALNT2	Muscular_dystrophy_t2,Myopathy_t2,Congenital_muscular_dystrophy
B3GALT6	Dermatology_t2,Skeletal_dysplasia_t2,Connective_tissue_disorder_t2,Skeletal_Dysplasia_Panel
B3GAT3	Skeletal_dysplasia_t2,Proportionate_short_stature_t2,Connective_tissue_disorder_t2,Osteogenesis_imperfecta,Skeletal_Dysplasia_Panel
B3GLCT	glaucoma
B3GNT2	Congenital_muscular_dystrophy
B4GALNT1	Hereditary_spastic_paraplegia_t2,Neurodegenerative_disease_t2
B4GALT1	Epilepsy_t2
B4GALT7	Dermatology_t2,Skeletal_dysplasia_t2,Connective_tissue_disorder_t1,Osteogenesis_imperfecta,Connective_Tissue_Disorder_and_Related
B4GAT1	Muscular_dystrophy_t2,Myopathy_t2
B9D1	Ataxia_t2,Hereditary_retinopathy_t2
B9D2	Hereditary_retinopathy_t2
BAAT	Skeletal_dysplasia_t2,Cholestasis_t1,Neonatal_cholestasis
BACH2	Autoimmunity_and_autoinflammatory_disorders
BAG3	Arrhythmia_t2,Muscular_dystrophy_t2,Charcot-Marie-Tooth_disease_t2,Cardiomyopathy_t1,Myopathy_t1,Charcot-Marie-Tooth,Dilated_cardiomyopathy,Hypertrophic_cardiomyopathy,Myofibrillar_myopathy,Cardiomyopathy_and_Related,Myopathy_and_Related,Sudden_Cardiac_Arrest_and_Related
BANP	corneal_keratoconus
BAP1	Dermatology_t2,Hereditary_pancancer
BARD1	Hereditary_cancer_syndrome_Plus_t1,Hereditary_pancancer,Breast_and_Ovarian_Cancer_Panel
BBIP1	Hereditary_retinopathy_t2,Hereditary_Autism_t2,retina
BBS1	Ataxia_t2,Retinitis_pigmentosa_t2,Hereditary_retinopathy_t2,Hereditary_Autism_t2,Disorders_of_sexual_development_t1,Hypogonadotropic_hypogonadism_t1,Retinitis_pigmentosa,Retinitis_Pigmentosa_and_Related,retina
BBS10	Ataxia_t2,Retinitis_pigmentosa_t2,Hereditary_retinopathy_t2,Hereditary_Autism_t2,Disorders_of_sexual_development_t1,Hypogonadotropic_hypogonadism_t1,Retinitis_Pigmentosa_and_Related,retina
BBS12	Ataxia_t2,Disorders_of_sexual_development_t2,Retinitis_pigmentosa_t2,Hereditary_retinopathy_t2,Hereditary_Autism_t2,Hypogonadotropic_hypogonadism_t1,Retinitis_Pigmentosa_and_Related,retina
BBS2	Ataxia_t2,Disorders_of_sexual_development_t2,Hereditary_retinopathy_t2,Hereditary_Autism_t2,Retinitis_pigmentosa_t1,Hypogonadotropic_hypogonadism_t1,Retinitis_pigmentosa,Retinitis_Pigmentosa_and_Related,retina

Supplementary Table 2. List of pathogenic or likely-pathogenic germline variants

Gene	Associated diseases
BBS4	Ataxia_t2,Disorders_of_sexual_development_t2,Retinitis_pigmentosa_t2,Hereditary_retinopathy_t2,Hypogonadotropic_hypogonadism_t2,Hereditary_Autism_t2,Leber's_Congenital_Amaurosis_and_Related,Retinitis_Pigmentosa_and_Related,retina
BBS5	Ataxia_t2,Disorders_of_sexual_development_t2,Retinitis_pigmentosa_t2,Hereditary_retinopathy_t2,Hypogonadotropic_hypogonadism_t2,Retinitis_Pigmentosa_and_Related,retina
BBS7	Ataxia_t2,Disorders_of_sexual_development_t2,Retinitis_pigmentosa_t2,Hereditary_retinopathy_t2,Hypogonadotropic_hypogonadism_t2,Hereditary_Autism_t2,Retinitis_Pigmentosa_and_Related,retina
BBS9	Ataxia_t2,Disorders_of_sexual_development_t2,Retinitis_pigmentosa_t2,Hereditary_retinopathy_t2,Hereditary_Autism_t2,Hypogonadotropic_hypogonadism_t1,Retinitis_Pigmentosa_and_Related,retina
BCAP31	Dystonia_t2
BCKDHA	Inborn_error_of_metabolism_t1,Inborn_Error_of_Metabolism
BCKDHB	Inborn_error_of_metabolism_t1,Inborn_Error_of_Metabolism
BCKDK	Epilepsy_t2
BCL10	Primary_immune_deficiency_t2,Lymphoma_t2
BCL11A	Hereditary_Autism_t2
BCL2	Lymphoma_t2,Lymphoma_B_cell_t1,Lymphoma_B_cell_t2,Myeloma_t1
BCL6	Lymphoma_t1,Lymphoma_B_cell_t1,Lymphoma_B_cell_t2,Lymphoma_T_NK_cell_t2
BCOR	Hereditary_retinopathy_t2,Hereditary_Autism_t2,Hematologic_malignancy,ALL_and_others_t1,ALL_and_others_t2,AML,Lymphoma_T_NK_cell_t1,Lymphoma_T_NK_cell_t2,MDS_MPN
BCORL1	Hematologic_malignancy
BCR	Cell-free_cancer,ALL_and_others_t2
BCS1L	Dermatology_t2,Skeletal_dysplasia_t2,Proportionate_short_stature_t2,Epilepsy_t2,Hearing_loss_t2,Hereditary_Autism_t2,Renal_Tubular_Disorders
BDNF	Hereditary_Autism_t2
BDP1	Hearing_loss_t2,Hereditary_hearing_loss
BEAN1	Ataxia_t2,Ataxia
BEST1	Hereditary_retinopathy_t2,Retinitis_pigmentosa_t1,Macular_dystrophy,Retinitis_pigmentosa,Cone-Rod_Dystrophy_and_Related,Macular_dystrophy_and_Related,Retinitis_Pigmentosa_and_Related,Vitreoretinopathy_and_Related,retina
BFSP1	Hereditary_retinopathy_t2
BFSP2	Hereditary_retinopathy_t2
BGN	Dermatology_t2,Skeletal_dysplasia_t2,Connective_tissue_disorder_t2,Overgrowth_Panel,Skeletal_Dysplasia_Panel
BHLHA9	Skeletal_dysplasia_t2
BHLHE41	Erythrocytosis
BICC1	Polycystic_Kidney_Disease_t2
BICD2	Muscular_dystrophy_t2,Myopathy_t2,Charcot-Marie-Tooth_disease_t2,Hereditary_spastic_paraplegia_t2,Neurodegenerative_disease_t2,Charcot_Marie_Tooth_and_Related
BIN1	Muscular_dystrophy_t2,Hereditary_Autism_t2,Myopathy_t1,Congenital_myopathy,Myopathy_and_Related
BIRC3	Lymphoma_t1,Hematologic_malignancy,Lymphoma_B_cell_t1,Lymphoma_B_cell_t2,Myeloma_t1
BLK	Polycystic_Kidney_Disease_t2,Maturity-Onset_Diabetes_of_the_Young,_MODY_t1,Monogenic_diabetes_panel
BLM	Dermatology_t2,Primary_immune_deficiency_t2,Congenital_hematologic_disease,Hereditary_colon_cancer,Hereditary_pancancer,Leukemia_predisposition,Bone_Marrow_Failure_Germline_Predisposition,Hereditary_Myeloid_Leukemia_Panel
BLNK	Primary_immune_deficiency_t2,Primary_immunodeficiency,B_cell_and_Humoral_Immune_Deficiency
BLOC1S3	Dermatology_t2,Coagulation_t2,Hereditary_retinopathy_t2,Albinism_and_Related,Bleeding_Platelet_Disorder
BLOC1S6	Dermatology_t2,Coagulation_t2,Hereditary_retinopathy_t2,Familial_hemophagocytic_lymphohistiocytosis,Albinism_and_Related,Bleeding_Platelet_Disorder,Hemophagocytic_Lymphohistiocytosis_and_Related
BMP1	Skeletal_dysplasia_t2,Osteogenesis_imperfecta
BMP2	Skeletal_dysplasia_t2
BMP4	Hereditary_retinopathy_t2,Anemia,corneal_keratoconus,glaucoma
BMP6	Anemia,Hemochromatosis

Supplementary Table 2. List of pathogenic or likely-pathogenic germline variants

Gene	Associated diseases
BMPER	Skeletal_dysplasia_t2
BMPR1A	Hereditary_cancer_syndrome_Plus_t2,Hereditary_colon_cancer,Hereditary_pancancer,Colorectal_Cancer_Polyposis_Panel
BMPR1B	Skeletal_dysplasia_t2,Proportionate_short_stature_t2,Skeletal_Dysplasia_Panel,Primary_Pulmonary_Hypertension_and_Related_Disordisorders
BMPR2	Primary_Pulmonary_Hypertension_and_Related_Disordisorders
BOLA3	Epilepsy_t2
BPGM	Anemia_t2,Erythrocytosis
BPNT2	Skeletal_dysplasia_t2,Skeletal_Dysplasia_Panel
BRAF	Cardiomyopathy_t2,Dermatology_t2,Skeletal_dysplasia_t2,Proportionate_short_stature_t2,Epilepsy_t2,Hereditary_cancer_syndrome_Plus_t2,Rasopathies_t1,Hereditary_Autism_t1,Neuroendocrine_tumor_t1,Lymphoma_t1,Cell-free_cancer,Hematologic_malignancy,Hereditary_paraganglioma-pheochromocytoma_syndrome,Noonan_syndrome,ALL_and_others_t1,ALL_and_others_t2,AML,Circulating_tumor_DNA_assay_55,Lymphoma_B_cell_t1,Lymphoma_B_cell_t2,Lymphoma_T_NK_cell_t1,Lymphoma_T_NK_cell_t2,MDS_MPN,Myeloma_t1,Noonan_Syndrome_and_Related
BRAT1	Epilepsy_t2,Hereditary_Microcephaly_t2
BRCA1	Dermatology_t2,Coagulation_t2,Hereditary_cancer_syndrome_Plus_t1,Cell-free_cancer,Congenital_hematologic_disease,Hereditary_breast_cancer,Hereditary_pancancer,Bone_Marrow_Failure_Germline_Predisposition,Circulating_tumor_DNA_assay_55,Germline_Cancer,Lymphoma_B_cell_t2
BRCA2	Dermatology_t2,Skeletal_dysplasia_t2,Coagulation_t2,Hereditary_cancer_syndrome_Plus_t1,Cell-free_cancer,Congenital_hematologic_disease,Fanconi_anemia,Hereditary_breast_cancer,Hereditary_pancancer,Bone_Marrow_Failure_Germline_Predisposition,ALL_and_others_t2,Circulating_tumor_DNA_assay_55,Colorectal_Cancer_Polyposis_Panel,Germline_Cancer,Lymphoma_B_cell_t2
BRCC3	Moyamoya_disease,Stroke
BRD2	Epilepsy_t2
BRIP1	Dermatology_t2,Skeletal_dysplasia_t2,Coagulation_t2,Hereditary_cancer_syndrome_Plus_t1,Congenital_hematologic_disease,Fanconi_anemia,Hereditary_breast_cancer,Hereditary_pancancer,Bone_Marrow_Failure_Germline_Predisposition,Breast_and_Ovarian_Cancer_Panel,Colorectal_Cancer_Polyposis_Panel
BRSK2	Hereditary_Autism_t2
BRWD3	Hereditary_Autism_t2,Overgrowth_intellectual_disability
BSCL2	Dermatology_t2,Ataxia_t2,Neurodegenerative_disease_t2,Amyotrophic_lateral_sclerosis_t2,Charcot-Marie-Tooth_disease_t1,Hereditary_spastic_paraplegia_t1,Charcot-Marie-Tooth,Hereditary_spastic_paraplegia_panel,Charcot_Marie_Tooth_and_Related,Spastic_Paraplegia_and_Related
BSND	Hearing_loss_t2,Atypical_Hemolytic_Uremic_Syndrome_t2,Acid-base-electro_gene,Hereditary_hearing_loss
BTD	Hereditary_spastic_paraplegia_t2,Lysosomal_storage_disease_t2,Inborn_error_of_metabolism_t2,Epilepsy_t2,Hearing_loss_t2,Neurodegenerative_disease_t2
BTG2	Lymphoma_t2
BTK	Primary_immune_deficiency_t2,Inflammatory_Bowel_t1,Lymphoma_t1,Cell-free_cancer,Familial_hemophagocytic_lymphohistiocytosis,Hematologic_malignancy,Primary_immunodeficiency,Very-early-onset_inflammatory_bowel_disease,B_cell_and_Humoral_Immune_Deficiency,Hemophagocytic_Lymphohistiocytosis_and_Related,Lymphoma_B_cell_t2,Lymphoma_T_NK_cell_t2,Myeloma_t1
BTRC	Skeletal_dysplasia_t2
BUB1B	Epilepsy_t2,Hereditary_Microcephaly_t2,Hereditary_Autism_t2,Hereditary_colon_cancer
BVES	Muscular_dystrophy_t2,Myopathy_t2
C12orf4	Ataxia_t2,Hereditary_Autism_t2
C12orf57	Epilepsy_t2,retina
C19orf12	Ataxia_t2,Hereditary_spastic_paraplegia_t2,Hereditary_retinopathy_t2,Dystonia_t2,Parkinson's_disease_t1,Neurodegenerative_disease_t1,Chorea,Dystonia,Neurodegeneration_with_brain_iron_accumulation
C1orf127	Androgenetic_alopecia
C1QTNF5	Retinitis_pigmentosa_t2,Hereditary_retinopathy_t2,Macular_dystrophy_and_Related,Retinitis_Pigmentosa_and_Related,retina
C1R	Dermatology_t2,Connective_tissue_disorder_t2,Primary_immunodeficiency
C1S	Dermatology_t2,Connective_tissue_disorder_t2,Primary_immunodeficiency

Supplementary Table 2. List of pathogenic or likely-pathogenic germline variants

Gene	Associated diseases
C2	Primary_immunodeficiency
C2CD3	Hereditary_retinopathy_t2
C3	Hereditary_retinopathy_t2,Atypical_Hemolytic_Uremic_Syndrome_t1,DNAJC21_EFL1_SBDS_SRP54,Atypical_Hemolytic_Uremic_Syndrome,Bleeding_Coagulopathy,Thrombosis
C4BPA	Atypical_Hemolytic_Uremic_Syndrome,Bleeding_Coagulopathy,Thrombosis
C4BPB	Atypical_Hemolytic_Uremic_Syndrome,Bleeding_Coagulopathy,Thrombosis
C5	Primary_immunodeficiency,Atypical_Hemolytic_Uremic_Syndrome,Bleeding_Coagulopathy,Thrombosis
C6	Primary_immunodeficiency
C7	Primary_immunodeficiency
C9	Primary_immunodeficiency
C9orf72	Alzheimer's_disease_t2,Dementia_t2,Parkinson's_disease_t2,Neurodegenerative_disease_t2,Amyotrophic_lateral_sclerosis_t2,Amyotrophic_lateral_sclerosis,Chorea
CA2	Skeletal_dysplasia_t2,Hereditary_retinopathy_t2,Atypical_Hemolytic_Uremic_Syndrome_t2,Hereditary_Autism_t2,Acid-base-electro_gene,Sclerosing_Bone_Disease_Panel,Renal_Tubular_Disorders
CA4	Hereditary_retinopathy_t2,Retinitis_pigmentosa_t1,Retinitis_pigmentosa,Retinitis_Pigmentosa_and_Related,retina
CA5A	Urea_cycle_disorders
CA8	Ataxia_t2
CABP2	Hearing_loss_t2,Hereditary_hearing_loss
CABP4	Hereditary_retinopathy_t2,Cone-Rod_Dystrophy_and_Related,Congenital_Stationary_Night_Blindness_and_Related,Leber's_Congenital_Amaurosis_and_Related,Retinitis_Pigmentosa_and_Related,retina
CACNA1A	Epilepsy_t2,Dystonia_t2,Parkinson's_disease_t2,Hereditary_Autism_t2,Ataxia_t1,Hereditary_Stroke_t1,Ataxia,Paroxysmal_movement_disorder,Stroke,Ataxia_and_Related,Nystagmus_and_Related,Optic_neuropathy_and_Related
CACNA1B	Epilepsy_t2,Dystonia_t1,Dyskinesia-dystonia-paralysis
CACNA1C	Cardiomyopathy_t2,Arrhythmia_t1,Hereditary_Autism_t1,Arrhythmia,Arrhythmia_and_Related
CACNA1D	Epilepsy_t2,Hearing_loss_t2,Atypical_Hemolytic_Uremic_Syndrome_t2
CACNA1E	Epilepsy_t2
CACNA1F	Retinitis_pigmentosa_t2,Hereditary_retinopathy_t2,Macular_dystrophy,Cone-Rod_Dystrophy_and_Related,Congenital_Stationary_Night_Blindness_and_Related,Retinitis_Pigmentosa_and_Related,retina
CACNA1G	Ataxia_t2,Hereditary_spastic_paraplegia_t2,Dystonia_t2,Neurodegenerative_disease_t2
CACNA1H	Epilepsy_t2,Atypical_Hemolytic_Uremic_Syndrome_t2
CACNA1S	Muscular_dystrophy_t2,Myopathy_t2,Atypical_Hemolytic_Uremic_Syndrome_t2,Acid-base-electro_gene,Dyskinesia-dystonia-paralysis
CACNA2D1	Arrhythmia_t1,Arrhythmia_and_Related
CACNA2D2	Epilepsy_t2
CACNA2D4	Hereditary_retinopathy_t2,Macular_dystrophy,Cone-Rod_Dystrophy_and_Related,Congenital_Stationary_Night_Blindness_and_Related,Retinitis_Pigmentosa_and_Related,retina
CACNB2	Cardiomyopathy_t2,Arrhythmia_t1,Arrhythmia,Arrhythmia_and_Related
CACNB4	Epilepsy_t2,Ataxia_t1,Ataxia,Paroxysmal_movement_disorder
CACNG2	Hereditary_Autism_t2
CALM1	Arrhythmia_t1,Arrhythmia,Arrhythmia_and_Related
CALM2	Arrhythmia_t2,Arrhythmia_and_Related
CALM3	Arrhythmia_t2
CALR	Hematologic_malignancy,ALL_and_others_t1,ALL_and_others_t2,AML,Erythrocytosis,MDS_MPN,Thrombosis
CALR3	Cardiomyopathy_t2,Hypertrophic_cardiomyopathy
CAMTA1	Ataxia_t2,Hereditary_Autism_t2
CANT1	Skeletal_dysplasia_t2,Hereditary_Autism_t2,Skeletal_Dysplasia_Panel
CAPN1	Ataxia_t2,Hereditary_spastic_paraplegia_t2,Neurodegenerative_disease_t2,Ataxia
CAPN3	Cardiomyopathy_t2,Myopathy_t2,Muscular_dystrophy_t1,Limb_girdle_muscular_dystrophy,Muscular_Dystrophy_and_Related
CAPN5	Hereditary_retinopathy_t2,Vitreoretinopathy,Vitreoretinopathy_and_Related,retina

Supplementary Table 2. List of pathogenic or likely-pathogenic germline variants

Gene	Associated diseases
CARD11	Primary_immune_deficiency_t2,Lymphoma_t1,Hematologic_malignancy,B_cell_and_Humoral_Immune_Deficiency,Lymphoma_B_cell_t1,Lymphoma_B_cell_t2,Lymphoma_T_NK_cell_t2,Phagocyte_Defect_and_Infectious_disease,Severe_Combined_Immunodeficiency_and_CID
CARD14	Dermatology_t2,Autoinflammatory_disorders
CARD9	Primary_immune_deficiency_t2,Phagocyte_Defect_and_Infectious_disease
CARS2	Epilepsy_t2
CASK	Ataxia_t2,Epilepsy_t2,Hereditary_Autism_t2,Hereditary_Microcephaly_t1,Epilepsy_and_Related
CASP10	Autoimmunity_and_autoinflammatory_disorders,B_cell_and_Humoral_Immune_Deficiency,Hemophagocytic_Lymphohistiocytosis_and_Related,Hereditary_Myeloid_Leukemia_Panel,Lymphoma_T_NK_cell_t2
CASP14	Dermatology_t2
CASP8	Autoimmunity_and_autoinflammatory_disorders,B_cell_and_Humoral_Immune_Deficiency,Hemophagocytic_Lymphohistiocytosis_and_Related
CASQ2	Cardiomyopathy_t2,Arrhythmia_t1,Arrhythmia,Arrhythmia_and_Related,Sudden_Cardiac_Arrest_and_Related
CASR	Skeletal_dysplasia_t2,Epilepsy_t2,Atypical_Hemolytic_Uremic_Syndrome_t2,Acid-base-electro_gene,Hereditary_pancancer,Osteogenesis_imperfecta,Rickets_Hypoparathyroidism_panel,Skeletal_Dysplasia_Panel,Epilepsy_and_Related
CAST	corneal_keratoconus
CASZ1	Cardiomyopathy_t2
CATSPER2	Hearing_loss_t2
CAV1	Primary_Pulmonary_Hypertension_and_Related_Disordisorders
CAV3	Cardiomyopathy_t2,Myopathy_t2,Arrhythmia_t1,Muscular_dystrophy_t1,Arrhythmia,Dilated_cardiomyopathy,Hypertrophic_cardiomyopathy,Limb_girdle_muscular_dystrophy,Arrhythmia_and_Related,Muscular_Dystrophy_and_Related
CAVIN1	Limb_girdle_muscular_dystrophy
CAVIN4	Cardiomyopathy_t2
CBFB	ALL_and_others_t2
CBL	Cardiomyopathy_t2,Skeletal_dysplasia_t2,Proportionate_short_stature_t2,Epilepsy_t2,Rasopathies_t1,Cell-free_cancer,Hematologic_malignancy,Noonan_syndrome,ALL_and_others_t1,ALL_and_others_t2,AML,Bone_Marrow_Failure_Germline_Predisposition,MDS_MPN,Noonan_Syndrome_and_Related
CBLB	Hematologic_malignancy
CBLC	Hematologic_malignancy
CBS	Dermatology_t2,Hereditary_Autism_t2,Connective_tissue_disorder_t1,Inborn_error_of_metabolism_t1,Hereditary_Stroke_t1,Familial_thoracic_aortic_aneurysms_and_aortic_dissections,Stroke,Connective_Tissue_Disorder_and_Related,Inborn_Error_of_Metabolism
CBX2	Disorders_of_sexual_development_t2,Disorders_of_sex_development
CC2D1A	Epilepsy_t2,Hereditary_Autism_t2
CC2D2A	Skeletal_dysplasia_t2,Ataxia_t2,Epilepsy_t2,Retinitis_pigmentosa_t2,Hereditary_Autism_t2,Hereditary_retinopathy_t1,Nephronophthisis_t1,Nephronophthisis_and_Related,Retinitis_Pigmentosa_and_Related,retina
CCBE1	Severe_Combined_Immunodeficiency_and_CID
CCDC103	Hereditary_Primary_Ciliary_Dyskinesia_t1
CCDC22	Hereditary_Autism_t2
CCDC39	Hereditary_Primary_Ciliary_Dyskinesia_t1
CCDC40	Hereditary_Primary_Ciliary_Dyskinesia_t1
CCDC47	Skeletal_dysplasia_t2,Hereditary_Microcephaly_t2
CCDC50	Hearing_loss_t2,Hereditary_hearing_loss
CCDC65	Hereditary_Primary_Ciliary_Dyskinesia_t1
CCDC78	Muscular_dystrophy_t2,Myopathy_t2
CCDC8	Skeletal_dysplasia_t2,Proportionate_short_stature_t2

Supplementary Table 2. List of pathogenic or likely-pathogenic germline variants

Gene	Associated diseases
CCDC88A	Optic_neuropathy_and_Related
CCDC88C	Ataxia_t2,Epilepsy_t2,Hereditary_Autism_t2
CCER1	corneal_dystrophy
CCL2	Epilepsy_t2
CCM2	Cerebral_cavernous_malformations,Primary_Pulmonary_Hypertension_and_Related_Disordisorders
CCN6	Skeletal_dysplasia_t2,Skeletal_Dysplasia_Panel
CCND1	Polycystic_Kidney_Disease_t2,Cell-free_cancer,Hematologic_malignancy,Circulating_tumor_DNA_assay_55,Lymphoma_B_cell_t1,Lymphoma_B_cell_t2,Myeloma_t1
CCND2	Cell-free_cancer,Hematologic_malignancy,Circulating_tumor_DNA_assay_55
CCND3	Lymphoma_t2,ALL_and_others_t2,Circulating_tumor_DNA_assay_55
CCNE1	Cell-free_cancer
CCNK	Rasopathies_t2
CCNO	Hereditary_Primary_Ciliary_Dyskinesia_t2
CCNQ	Skeletal_dysplasia_t2
CCR4	Lymphoma_T_NK_cell_t2
CCR7	Lymphoma_T_NK_cell_t2
CCT5	Charcot-Marie-Tooth_disease_t2,Hereditary_spastic_paraplegia_t2,Neurodegenerative_disease_t2,Charcot_Marie_Tooth_and_Related
CD151	Dermatology_t2,Hearing_loss_t2,Alport_Syndrome_t1,Glomerular_basement_membrane_disorders
CD164	Hearing_loss_t2,Hereditary_hearing_loss
CD19	Primary_immune_deficiency_t1,Primary_immunodeficiency,B_cell_and_Humoral_Immune_Deficiency
CD247	Primary_immune_deficiency_t2,Severe_Combined_Immunodeficiency_and_CID
CD27	Primary_immune_deficiency_t1,Familial_hemophagocytic_lymphohistiocytosis,B_cell_and_Humoral_Immune_Deficiency,Autoimmunity_and_autoinflammatory_disorders,Hemophagocytic_Lymphohistiocytosis_and_Related
CD274	Cell-free_cancer,Lymphoma_T_NK_cell_t2
CD28	Hematologic_malignancy,Lymphoma_T_NK_cell_t1,Lymphoma_T_NK_cell_t2
CD36	Coagulation_t2,Bleeding_Platelet_Disorder
CD38	Myeloma_t1
CD3D	Primary_immune_deficiency_t2,Severe_Combined_Immunodeficiency_and_CID
CD3E	Primary_immune_deficiency_t2,Severe_Combined_Immunodeficiency_and_CID
CD3G	Primary_immune_deficiency_t2,Inflammatory_Bowel_t1,Very-early-onset_inflammatory_bowel_disease
CD40	Primary_immune_deficiency_t1,B_cell_and_Humoral_Immune_Deficiency,Lymphoma_T_NK_cell_t2,Severe_Combined_Immunodeficiency_and_CID
CD40LG	Primary_immune_deficiency_t1,Inflammatory_Bowel_t1,Very-early-onset_inflammatory_bowel_disease,B_cell_and_Humoral_Immune_Deficiency,Severe_Combined_Immunodeficiency_and_CID
CD46	Atypical_Hemolytic_Uremic_Syndrome_t1,DNAJC21_EFL1_SBDS_SRP54,Atypical_Hemolytic_Uremic_Syndrome,Bleeding_Coagulopathy,Thrombosis
CD55	Atypical_Hemolytic_Uremic_Syndrome,Bleeding_Coagulopathy,Thrombosis
CD58	Hematologic_malignancy,Lymphoma_B_cell_t1,Lymphoma_B_cell_t2
CD59	Atypical_Hemolytic_Uremic_Syndrome_t2,Anemia_t2,Hemolytic_anemia,Anemia,Atypical_Hemolytic_Uremic_Syndrome,Bleeding_Coagulopathy,Thrombosis
CD79A	Primary_immune_deficiency_t2,Lymphoma_t1,Hematologic_malignancy,Primary_immunodeficiency,B_cell_and_Humoral_Immune_Deficiency,Lymphoma_B_cell_t2,Lymphoma_T_NK_cell_t2
CD79B	Primary_immune_deficiency_t2,Lymphoma_t1,Primary_immunodeficiency,B_cell_and_Humoral_Immune_Deficiency,Lymphoma_B_cell_t1,Lymphoma_B_cell_t2,Myeloma_t1
CD81	B_cell_and_Humoral_Immune_Deficiency
CD83	Lymphoma_t2
CD8A	Primary_immune_deficiency_t2,Severe_Combined_Immunodeficiency_and_CID
CDAN1	Coagulation_t2,Anemia_t2,Congenital_hematologic_disease,Hemolytic_anemia,Anemia,Hemochromatosis
CDC14A	Hearing_loss_t2,Hereditary_hearing_loss

Supplementary Table 2. List of pathogenic or likely-pathogenic germline variants

Gene	Associated diseases
CDC25C	Hematologic_malignancy
CDC42	Skeletal_dysplasia_t2,Rasopathies_t2,Hearing_loss_t2,Hereditary_Autism_t2
CDC45	Skeletal_dysplasia_t2,Proportionate_short_stature_t2,Craniosynostosis
CDC6	Skeletal_dysplasia_t2,Proportionate_short_stature_t1
CDC73	Hereditary_pancancer,Osteogenesis_imperfecta,Skeletal_Dysplasia_Panel
CDH1	Hereditary_cancer_syndrome_Plus_t1,Cell-free_cancer,Hereditary_breast_cancer,Hereditary_colon_cancer,Hereditary_pancancer,Breast_and_Ovarian_Cancer_Panel
CDH11	Skeletal_dysplasia_t2
CDH15	Hereditary_Autism_t2
CDH2	Arrhythmia_t2,Cardiomyopathy_t2
CDH23	Retinitis_pigmentosa_t2,Hearing_loss_t1,Hereditary_retinopathy_t1,Hereditary_hearing_loss,Hearing_Loss_and_Related_t1,Hearing_Loss_and_Related_t2,Retinitis_Pigmentosa_and_Related,retina
CDH3	Dermatology_t2,Skeletal_dysplasia_t2,Hereditary_retinopathy_t2,Macular_dystrophy_and_Related,retina
CDHR1	Hereditary_retinopathy_t2,Retinitis_pigmentosa_t1,Macular_dystrophy,Cone-Rod_Dystrophy_and_Related,Retinitis_Pigmentosa_and_Related,retina
CDIN1	Hemolytic_anemia,Anemia
CDK12	Lymphoma_B_cell_t2
CDK13	Hereditary_Autism_t2
CDK16	Hereditary_Autism_t2
CDK4	Dermatology_t2,Cell-free_cancer,Hereditary_pancancer,ALL_and_others_t2,Circulating_tumor_DNA_assay_55,Lymphoma_B_cell_t1,Lymphoma_B_cell_t2,Myeloma_t1
CDK5	Hereditary_pancancer,Malformation_of_cortical_development
CDK5RAP2	Hereditary_Microcephaly_t1
CDK6	Hereditary_Microcephaly_t2,Cell-free_cancer,ALL_and_others_t2,Circulating_tumor_DNA_assay_55,Lymphoma_B_cell_t2,Myeloma_t1
CDK9	Epilepsy_t2,Hearing_loss_t2
CDKL5	Hereditary_Microcephaly_t2,Hereditary_Autism_t2,Epilepsy_t1,Early_onset_epilepsy,Autism_and_Related,Epilepsy_and_Related
CDKN1A	ALL_and_others_t2,Lymphoma_T_NK_cell_t1,Lymphoma_T_NK_cell_t2
CDKN1B	Hereditary_pancancer
CDKN1C	Skeletal_dysplasia_t2,Hearing_loss_t2,Hereditary_Autism_t2,Hereditary_pancancer
CDKN2A	Dermatology_t2,Hereditary_cancer_syndrome_Plus_t2,Lymphoma_t2,Neuroendocrine_tumor_t1,Cell-free_cancer,Hematologic_malignancy,Hereditary_pancancer,Hereditary_paraganglioma-pheochromocytoma_syndrome,ALL_and_others_t1,ALL_and_others_t2,Lymphoma_B_cell_t1,Lymphoma_B_cell_t2,Lymphoma_T_NK_cell_t1,Lymphoma_T_NK_cell_t2,Myeloma_t1
CDKN2B	ALL_and_others_t1,ALL_and_others_t2,Lymphoma_B_cell_t1,Lymphoma_B_cell_t2,Lymphoma_T_NK_cell_t1,Lymphoma_T_NK_cell_t2
CDKN2C	Lymphoma_B_cell_t2,Myeloma_t1
CDRT15	Charcot_Marie_Tooth_and_Related
CDRT4	Charcot_Marie_Tooth_and_Related
CDSN	Dermatology_t2
CDT1	Skeletal_dysplasia_t2,Proportionate_short_stature_t1
CEACAM16	Hearing_loss_t2,Hereditary_hearing_loss
CEBPA	Cell-free_cancer,Congenital_hematologic_disease,Hematologic_malignancy,Hereditary_pancancer,Leukemia_predisposition,ALL_and_others_t1,ALL_and_others_t2,AML,Bone_Marrow_Failure_Germline_Predisposition,Hereditary_Myeloid_Leukemia_Panel,MDS_MPN
CEL	Polycystic_Kidney_Disease_t2,Maturity-Onset_Diabetes_of_the_Young,_MODY_t1,Monogenic_diabetes_panel
CENPE	Hereditary_Microcephaly_t2
CENPF	Hereditary_Primary_Ciliary_Dyskinesia_t2,Hereditary_Microcephaly_t2
CENPJ	Skeletal_dysplasia_t2,Proportionate_short_stature_t2,Epilepsy_t2,Hereditary_Microcephaly_t1
CEP104	Hereditary_retinopathy_t2

Supplementary Table 2. List of pathogenic or likely-pathogenic germline variants

Gene	Associated diseases
CEP120	Skeletal_dysplasia_t2,Hereditary_retinopathy_t2,Skeletal_Dysplasia_Panel
CEP135	Hereditary_Microcephaly_t1
CEP152	Skeletal_dysplasia_t2,Proportionate_short_stature_t2,Hereditary_Microcephaly_t1
CEP164	Hereditary_retinopathy_t2,Polycystic_Kidney_Disease_t2,Nephronophthisis_t1,Nephronophthisis_and_Related,retina
CEP19	Hereditary_retinopathy_t2
CEP250	Hearing_loss_t2,Hereditary_retinopathy_t2,Cone-Rod_Dystrophy_and_Related,retina
CEP290	Skeletal_dysplasia_t2,Ataxia_t2,Epilepsy_t2,Retinitis_pigmentosa_t2,Polycystic_Kidney_Disease_t2,Hereditary_Autism_t2,Hereditary_retinopathy_t1,Nephronophthisis_t1,Leber_congenital_amaurosis,Leber's_Congenital_Amaurosis_and_Related,Nephronophthisis_and_Related,Retinitis_Pigmentosa_and_Related,retina
CEP41	Ataxia_t2,Hereditary_retinopathy_t2,Hereditary_Autism_t2,Nephronophthisis_t1,Nephronophthisis_and_Related
CEP57	Hereditary_Autism_t2
CEP63	Skeletal_dysplasia_t2,Proportionate_short_stature_t2,Hereditary_Microcephaly_t1
CEP78	Hearing_loss_t2,Hereditary_retinopathy_t2,Cone-Rod_Dystrophy_and_Related
CEP83	Polycystic_Kidney_Disease_t2,Nephronophthisis_t1,Nephronophthisis_and_Related
CERKL	Hereditary_retinopathy_t2,Retinitis_pigmentosa_t1,Macular_dystrophy,Retinitis_pigmentosa,Cone-Rod_Dystrophy_and_Related,Macular_dystrophy_and_Related,Retinitis_Pigmentosa_and_Related,retina
CERS1	Epilepsy_t2
CERS3	Dermatology_t2
CERT1	Epilepsy_t2,Hereditary_Autism_t2
CETP	Hereditary_Dyslipidemia_t1,Dyslipidemia_and_Related
CFAP298	Hereditary_Primary_Ciliary_Dyskinesia_t1
CFAP300	Hereditary_Primary_Ciliary_Dyskinesia_t2
CFAP410	Retinitis_pigmentosa_t2,Hereditary_retinopathy_t2,Skeletal_Dysplasia_Panel,Cone-Rod_Dystrophy_and_Related,Retinitis_Pigmentosa_and_Related,retina
CFAP418	Hereditary_retinopathy_t2,Retinitis_pigmentosa_t1,Macular_dystrophy,Retinitis_pigmentosa,Cone-Rod_Dystrophy_and_Related,Retinitis_Pigmentosa_and_Related,retina
CFB	Hereditary_retinopathy_t2,Atypical_Hemolytic_Uremic_Syndrome_t2,DNAJC21_EFL1_SBDS_SRP54,Atypical_Hemolytic_Uremic_Syndrome,Bleeding_Coagulopathy,Phagocyte_Defect_and_Infectious_disease,Thrombosis
CFD	Atypical_Hemolytic_Uremic_Syndrome,Bleeding_Coagulopathy,Thrombosis
CFH	Hereditary_retinopathy_t2,Atypical_Hemolytic_Uremic_Syndrome_t1,DNAJC21_EFL1_SBDS_SRP54,Atypical_Hemolytic_Uremic_Syndrome,Bleeding_Coagulopathy,Thrombosis,retina
CFHR1	Atypical_Hemolytic_Uremic_Syndrome_t2,DNAJC21_EFL1_SBDS_SRP54,Atypical_Hemolytic_Uremic_Syndrome,Bleeding_Coagulopathy,Thrombosis
CFHR2	Atypical_Hemolytic_Uremic_Syndrome_t2,DNAJC21_EFL1_SBDS_SRP54,Atypical_Hemolytic_Uremic_Syndrome,Bleeding_Coagulopathy,Thrombosis
CFHR3	Atypical_Hemolytic_Uremic_Syndrome_t2,DNAJC21_EFL1_SBDS_SRP54,Atypical_Hemolytic_Uremic_Syndrome,Bleeding_Coagulopathy,Thrombosis
CFHR4	DNAJC21_EFL1_SBDS_SRP54,Atypical_Hemolytic_Uremic_Syndrome,Bleeding_Coagulopathy,Thrombosis
CFHR5	Atypical_Hemolytic_Uremic_Syndrome_t2,DNAJC21_EFL1_SBDS_SRP54,Atypical_Hemolytic_Uremic_Syndrome,Bleeding_Coagulopathy,Thrombosis
CFI	Hereditary_retinopathy_t2,Atypical_Hemolytic_Uremic_Syndrome_t1,DNAJC21_EFL1_SBDS_SRP54,Atypical_Hemolytic_Uremic_Syndrome,Bleeding_Coagulopathy,Thrombosis
CFL1	corneal_keratoconus
CFL2	Muscular_dystrophy_t2,Myopathy_t1,Congenital_myopathy,Myofibrillar_myopathy,Myopathy_and_Related
CFP	Atypical_Hemolytic_Uremic_Syndrome,Bleeding_Coagulopathy,Thrombosis
CFTR	Hereditary_Primary_Ciliary_Dyskinesia_t2,Cholestasis_t2
CHAMP1	Hereditary_Autism_t2
CHAT	Ataxia_t2,Congenital_myopathy
CHCHD10	Charcot-Marie-Tooth_disease_t2,Amyotrophic_lateral_sclerosis_t2,Dementia_t1,Neurodegenerative_disease_t1,Amyotrophic_lateral_sclerosis,Dementia_and_Related
CHCHD2	Dementia_t2,Parkinson's_disease_t2,Neurodegenerative_disease_t2,Parkinson_Disease_and_Related
CHD2	Epilepsy_t2,Hereditary_Autism_t2,Early_onset_epilepsy,Autism_and_Related

Supplementary Table 2. List of pathogenic or likely-pathogenic germline variants

Gene	Associated diseases
CHD7	Primary_immune_deficiency_t2,Hearing_loss_t2,Hereditary_retinopathy_t2,Hereditary_Autism_t2,Disorders_of_sexual_development_t1,Hypogonadotropic_hypogonadism_t1,Hereditary_hearing_loss,Isolated_Hypogonadotropic_Hypogonadism,Hearing_Loss_and_Related_t2,Severe_Combined_Immunodeficiency_and_CID
CHD8	Hereditary_Autism_t2,Hematologic_malignancy,Autism_and_Related
CHEK1	Lymphoma_B_cell_t2
CHEK2	Hereditary_cancer_syndrome_Plus_t1,Hereditary_breast_cancer,Hereditary_colon_cancer,Hereditary_pancancer,Breast_and_Ovarian_Cancer_Panel,Circulating_tumor_DNA_assay_55
CHIT1	Lysosomal_storage_disease_t2
CHKB	Muscular_dystrophy_t2,Myopathy_t2,Congenital_muscular_dystrophy
CHM	Retinitis_pigmentosa_t2,Hereditary_retinopathy_t2,Congenital_Stationary_Night_Blindness_and_Related,Retinitis_Pigmentosa_and_Related,retina
CHMP1A	Hereditary_Microcephaly_t2
CHMP2B	Dementia_t1,Neurodegenerative_disease_t1,Amyotrophic_lateral_sclerosis_t1,Amyotrophic_lateral_sclerosis,Dementia_and_Related
CHMP4B	Hereditary_retinopathy_t2
CHN1	Nystagmus_and_Related
CHRM2	Cardiomyopathy_t2
CHRNA1	Congenital_myopathy,Nystagmus_and_Related
CHRNA2	Epilepsy_t1,Focal_epilepsy
CHRNA4	Hereditary_Autism_t2,Epilepsy_t1,Amyotrophic_lateral_sclerosis_t1
CHRNA7	Epilepsy_t2
CHRNB1	Congenital_myopathy
CHRNB2	Epilepsy_t1
CHRND	Congenital_myopathy
CHRNE	Myopathy_t1,Congenital_myopathy,Myopathy_and_Related
CHST14	Dermatology_t2,Skeletal_dysplasia_t2,Connective_tissue_disorder_t1,Skeletal_Dysplasia_Panel,Connective_Tissue_Disorder_and_Related
CHST3	Skeletal_dysplasia_t2,Skeletal_Dysplasia_Panel,Spondyloepiphyseal_metaphyseal_dysplasia
CHST6	Hereditary_retinopathy_t2,Corneal_Dystrophy_and_Related,corneal_dystrophy
CHST8	Dermatology_t2
CHSY1	Skeletal_dysplasia_t2,Hearing_loss_t2
CIB2	Hearing_loss_t2,Hereditary_retinopathy_t1,Hereditary_hearing_loss,Hearing_Loss_and_Related_t2,Retinitis_Pigmentosa_and_Related,retina
CIC	Hereditary_Autism_t2
CIITA	Primary_immune_deficiency_t2,Severe_Combined_Immunodeficiency_and_CID
CILK1	Skeletal_dysplasia_t2,Epilepsy_t2,Skeletal_Dysplasia_Panel
CISD2	Hearing_loss_t2,Hereditary_retinopathy_t2,Monogenic_diabetes_panel,Optic_neuropathy_and_Related
CIT	Hereditary_Microcephaly_t2
CIZ1	Dystonia_t1,Dyskinesia-dystonia-paralysis
CKAP2L	Skeletal_dysplasia_t2,Hereditary_Microcephaly_t2
CLCC1	Retinitis_pigmentosa,retina
CLCN1	Muscular_dystrophy_t2,Myopathy_t2,Dyskinesia-dystonia-paralysis
CLCN2	Ataxia_t2,Epilepsy_t2,Epilepsy_and_Related
CLCN4	Epilepsy_t2,Hereditary_Autism_t2
CLCN5	Skeletal_dysplasia_t2,Ca-Pi-Mg,Rickets_Hypoparathyroidism_panel,Skeletal_Dysplasia_Panel,Renal_Tubular_Disorders
CLCN7	Skeletal_dysplasia_t2,Sclerosing_Bone_Disease_Panel
CLCNKA	Atypical_Hemolytic_Uremic_Syndrome_t2,Acid-base-electro_gene
CLCNKB	Atypical_Hemolytic_Uremic_Syndrome_t2,Acid-base-electro_gene,Renal_Tubular_Disorders

Supplementary Table 2. List of pathogenic or likely-pathogenic germline variants

Gene	Associated diseases
CLDN1	Dermatology_t2,Neonatal_cholestasis
CLDN14	Hearing_loss_t2,Hereditary_hearing_loss,Hearing_Loss_and_Related_t2
CLDN16	Atypical_Hemolytic_Uremic_Syndrome_t2,Ca-Pi-Mg,Rickets_Hypoparathyroidism_panel,Renal_Tubular_Disorders
CLDN19	Atypical_Hemolytic_Uremic_Syndrome_t2,Ca-Pi-Mg
CLIC2	Hereditary_Autism_t2
CLIC5	Hearing_loss_t2,Hereditary_hearing_loss
CLN3	Lysosomal_storage_disease_t2,Epilepsy_t2,Retinitis_pigmentosa_t2,Hereditary_Autism_t2,Hereditary_retinopathy_t1,Cone-Rod_Dystrophy_and_Related,Epilepsy_and_Related,Retinitis_Pigmentosa_and_Related,retina
CLN5	Ataxia_t2,Lysosomal_storage_disease_t2,Epilepsy_t2,Hereditary_retinopathy_t2,Hereditary_Autism_t2,Epilepsy_and_Related
CLN6	Lysosomal_storage_disease_t2,Epilepsy_t2,Hereditary_retinopathy_t2,Hereditary_Autism_t2,Epilepsy_and_Related
CLN8	Lysosomal_storage_disease_t2,Epilepsy_t2,Hereditary_retinopathy_t2,Hereditary_Autism_t2,Epilepsy_and_Related
CLPB	Hereditary_retinopathy_t2,Congenital_hematologic_disease,Severe_congenital_neutropenia
CLPP	Ataxia_t2,Hearing_loss_t2,Hereditary_hearing_loss
CLRN1	Hereditary_retinopathy_t2,Retinitis_pigmentosa_t1,Hearing_loss_t1,Hereditary_hearing_loss,Retinitis_pigmentosa,Hearing_Loss_and_Related_t1,Hearing_Loss_and_Related_t2,Retinitis_Pigmentosa_and_Related,retina
CLTC	Epilepsy_t2
CLU	corneal_dystrophy
CMPK1	Hemolytic_anemia
CNGA1	Hereditary_retinopathy_t2,Retinitis_pigmentosa_t1,Retinitis_pigmentosa,Retinitis_Pigmentosa_and_Related,retina,CODA
CNGA3	Hereditary_retinopathy_t2,Macular_dystrophy,Achromatopsia_and_Related,Cone-Rod_Dystrophy_and_Related,Leber's_Congenital_Amaurosis_and_Related,Retinitis_Pigmentosa_and_Related,retina
CNGB1	Hereditary_retinopathy_t2,Retinitis_pigmentosa_t1,Retinitis_pigmentosa,Retinitis_Pigmentosa_and_Related,retina
CNGB3	Hereditary_retinopathy_t2,Macular_dystrophy,Achromatopsia_and_Related,Cone-Rod_Dystrophy_and_Related,Macular_dystrophy_and_Related,Retinitis_Pigmentosa_and_Related,retina
CNKSR2	Epilepsy_t2,Hereditary_Autism_t2
CNNM2	Atypical_Hemolytic_Uremic_Syndrome_t2,Ca-Pi-Mg
CNNM4	Hereditary_retinopathy_t2,Macular_dystrophy,Cone-Rod_Dystrophy_and_Related,Retinitis_Pigmentosa_and_Related,retina
CNOT3	Hereditary_Autism_t2
CNPY3	Epilepsy_t2
CNTN1	Muscular_dystrophy_t2,Myopathy_t2
CNTN2	Epilepsy_t2
CNTN6	Hereditary_Autism_t2
CNTNAP1	Charcot-Marie-Tooth
CNTNAP2	Epilepsy_t2,Hereditary_Autism_t1,Autism_and_Related
CNTNAP5	Hereditary_Autism_t2
COA7	Charcot-Marie-Tooth_disease_t2,Ataxia_t2,Epilepsy_t2,Charcot-Marie-Tooth
COA8	Epilepsy_t2
COASY	Ataxia_t2,Hereditary_spastic_paraplegia_t2,Dystonia_t2,Parkinson's_disease_t2,Neurodegenerative_disease_t2,Neurodegeneration_with_brain_iron_accumulation
COCH	Hearing_loss_t1,Hereditary_hearing_loss,Hearing_Loss_and_Related_t1,Hearing_Loss_and_Related_t2
COG5	Hereditary_Autism_t2
COG7	Epilepsy_t2,Hereditary_Autism_t2
COG8	Epilepsy_t2,Hereditary_Autism_t2
COL10A1	Skeletal_dysplasia_t1,Skeletal_Dysplasia_Panel,Spondyloepiphyseal_metaphyseal_dysplasia,Skeletal_Dysplasia_and_Related

Supplementary Table 2. List of pathogenic or likely-pathogenic germline variants

Gene	Associated diseases
COL11A1	Dermatology_t2,Skeletal_dysplasia_t2,Hereditary_retinopathy_t2,Connective_tissue_disorder_t1,Hearing_loss_t1,Hereditary_hearing_loss,Skeletal_Dysplasia_Panel,Spondyloepiphyseal_metaphyseal_dysplasia,Vitreoretinopathy,Connective_Tissue_Disorder_and_Related,Hearing_Loss_and_Related_t1,Hearing_Loss_and_Related_t2,Vitreoretinopathy_and_Related,retina
COL11A2	Skeletal_dysplasia_t2,Connective_tissue_disorder_t2,Lysosomal_storage_disease_t2,Hearing_loss_t2,Hereditary_retinopathy_t2,Hereditary_hearing_loss,Skeletal_Dysplasia_Panel,Vitreoretinopathy,Connective_Tissue_Disorder_and_Related,Hearing_Loss_and_Related_t2,Vitreoretinopathy_and_Related,retina
COL12A1	Dermatology_t2,Connective_tissue_disorder_t2,Muscular_dystrophy_t2,Myopathy_t2
COL17A1	Hereditary_retinopathy_t2,Dermatology_t1,corneal_dystrophy
COL18A1	Epilepsy_t2,Hereditary_retinopathy_t2,Vitreoretinopathy,Vitreoretinopathy_and_Related,retina
COL1A1	Dermatology_t2,Skeletal_dysplasia_t1,Connective_tissue_disorder_t1,Osteogenesis_imperfecta,Skeletal_Dysplasia_and_Related,corneal_keratoconus,glaucoma
COL1A2	Dermatology_t2,Skeletal_dysplasia_t1,Connective_tissue_disorder_t1,Osteogenesis_imperfecta,Connective_Tissue_Disorder_and_Related,Skeletal_Dysplasia_and_Related
COL27A1	Skeletal_dysplasia_t2,Proportionate_short_stature_t2,Skeletal_Dysplasia_Panel
COL2A1	Dermatology_t2,Lysosomal_storage_disease_t2,Hereditary_retinopathy_t2,Skeletal_dysplasia_t1,Connective_tissue_disorder_t1,Hearing_loss_t1,Hereditary_hearing_loss,Skeletal_Dysplasia_Panel,Spondyloepiphyseal_metaphyseal_dysplasia,Vitreoretinopathy,Connective_Tissue_Disorder_and_Related,Hearing_Loss_and_Related_t1,Hearing_Loss_and_Related_t2,Skeletal_Dysplasia_and_Related,Vitreoretinopathy_and_Related,retina
COL3A1	Dermatology_t2,Skeletal_dysplasia_t2,Connective_tissue_disorder_t1,Hereditary_Stroke_t1,Familial_thoracic_aortic_aneurysms_and_aortic_dissections,Stroke,Connective_Tissue_Disorder_and_Related,Marfan_and_Related
COL4A1	Muscular_dystrophy_t2,Myopathy_t2,Epilepsy_t2,Hereditary_retinopathy_t2,Polycystic_Kidney_Disease_t1,Hereditary_Stroke_t1,Hemolytic_anemia,Malformation_of_cortical_development,Stroke,Anemia,Polycystic_kidney_disease_and_Related,corneal_keratoconus,glaucoma
COL4A2	Muscular_dystrophy_t2,Myopathy_t2,Hereditary_Stroke_t1,Stroke
COL4A3	Hearing_loss_t2,Alport_Syndrome_t1,Glomerulopathy,Hereditary_hearing_loss,Glomerular_basement_membrane_disorders,corneal_dystrophy,corneal_keratoconus
COL4A4	Hearing_loss_t2,Alport_Syndrome_t1,Glomerulopathy,Hereditary_hearing_loss,Glomerular_basement_membrane_disorders,corneal_keratoconus
COL4A5	Connective_tissue_disorder_t2,Hearing_loss_t2,Alport_Syndrome_t1,Glomerulopathy,Hereditary_hearing_loss,Glomerular_basement_membrane_disorders
COL4A6	Hearing_loss_t2,Alport_Syndrome_t2,Hereditary_hearing_loss
COL5A1	Dermatology_t2,Skeletal_dysplasia_t2,Connective_tissue_disorder_t1,Ehlers-Danlos_syndrome,Familial_thoracic_aortic_aneurysms_and_aortic_dissections,Stroke,Connective_Tissue_Disorder_and_Related,Keratoconus_and_Related,corneal_keratoconus
COL5A2	Dermatology_t2,Skeletal_dysplasia_t2,Connective_tissue_disorder_t1,Ehlers-Danlos_syndrome,Familial_thoracic_aortic_aneurysms_and_aortic_dissections,Stroke,Connective_Tissue_Disorder_and_Related
COL6A1	Myopathy_t2,Muscular_dystrophy_t1,Congenital_muscular_dystrophy,Muscular_Dystrophy_and_Related,corneal_keratoconus
COL6A2	Myopathy_t2,Muscular_dystrophy_t1,Congenital_muscular_dystrophy,Muscular_Dystrophy_and_Related
COL6A3	Myopathy_t2,Ataxia_t2,Muscular_dystrophy_t1,Dystonia_t1,Congenital_muscular_dystrophy,Dyskinesia-dystonia-paralysis,Dystonia_and_Related,Muscular_Dystrophy_and_Related
COL7A1	Inflammatory_Bowel_t2,Dermatology_t1,Very-early-onset_inflammatory_bowel_disease
COL8A1	corneal_dystrophy,corneal_keratoconus
COL8A2	Hereditary_retinopathy_t2,Corneal_Dystrophy_and_Related,Keratoconus_and_Related,corneal_dystrophy,corneal_keratoconus
COL9A1	Hearing_loss_t2,Hereditary_retinopathy_t2,Skeletal_dysplasia_t1,Connective_tissue_disorder_t1,Hereditary_hearing_loss,Skeletal_Dysplasia_Panel,Spondyloepiphyseal_metaphyseal_dysplasia,Vitreoretinopathy,Connective_Tissue_Disorder_and_Related,Hearing_Loss_and_Related_t2,Vitreoretinopathy_and_Related,retina
COL9A2	Hearing_loss_t2,Hereditary_retinopathy_t2,Skeletal_dysplasia_t1,Connective_tissue_disorder_t1,Hereditary_hearing_loss,Skeletal_Dysplasia_Panel,Spondyloepiphyseal_metaphyseal_dysplasia,Vitreoretinopathy,Connective_Tissue_Disorder_and_Related,Vitreoretinopathy_and_Related,retina
COL9A3	Connective_tissue_disorder_t2,Hearing_loss_t2,Hereditary_retinopathy_t2,Skeletal_dysplasia_t1,Skeletal_Dysplasia_Panel,Spondyloepiphyseal_metaphyseal_dysplasia,Vitreoretinopathy,Skeletal_Dysplasia_and_Related,Vitreoretinopathy_and_Related,retina
COLGALT1	Connective_tissue_disorder_t2
COLQ	Congenital_myopathy
COMP	Skeletal_dysplasia_t1,Skeletal_Dysplasia_Panel,Spondyloepiphyseal_metaphyseal_dysplasia,Skeletal_Dysplasia_and_Related
COPA	Autoimmunity_and_autoinflammatory_disorders,B_cell_and_Humoral_Immune_Deficiency

Supplementary Table 2. List of pathogenic or likely-pathogenic germline variants

Gene	Associated diseases
COQ2	Myopathy_t2,Ataxia_t2,Epilepsy_t2,Ataxia,Glomerulopathy
COQ4	Epilepsy_t2
COQ6	Ataxia_t2,Glomerulopathy
COQ8A	Myopathy_t2,Epilepsy_t2,Ataxia_t1,Ataxia_and_Related
COQ8B	Glomerulopathy
COQ9	Ataxia_t2,Epilepsy_t2
CORO1A	Primary_immune_deficiency_t2,Severe_Combined_Immunodeficiency_and_CID
COX10	Charcot-Marie-Tooth_disease_t2,Epilepsy_t2,Charcot_Marie_Tooth_and_Related,Optic_neuropathy_and_Related
COX15	Cardiomyopathy_t2,Epilepsy_t2
COX20	Ataxia_t2
COX6A1	Charcot-Marie-Tooth_disease_t2,Charcot_Marie_Tooth_and_Related
COX6B1	Epilepsy_t2
COX7B	Hereditary_Microcephaly_t2
CP	Ataxia_t2,Dystonia_t2,Parkinson's_disease_t2,Hereditary_Autism_t2,Ataxia,Chorea,Neurodegeneration_with_brain_iron_accumulation,Anemia,Hemochromatosis
CPA6	Epilepsy_t2,Hereditary_Autism_t2,Epilepsy_and_Related
CPAMD8	glaucoma
CPE	Hereditary_retinopathy_t2,Hypogonadotropic_hypogonadism_t2
CPLANE1	Ataxia_t2,Hereditary_retinopathy_t1
CPOX	Dermatology_t2
CPS1	Inborn_error_of_metabolism_t2,Hereditary_Autism_t2,Urea_cycle_disorders
CPT1A	Arrhythmia_t2,Inborn_error_of_metabolism_t2,Metabolic_myopathy
CPT1C	Hereditary_spastic_paraplegia_t2,Neurodegenerative_disease_t2
CPT2	Cardiomyopathy_t2,Muscular_dystrophy_t2,Inborn_error_of_metabolism_t2,Epilepsy_t2,Myopathy_t1,Metabolic_myopathy,Myopathy_and_Related
CR1	Atypical_Hemolytic_Uremic_Syndrome_t2
CR2	Primary_immune_deficiency_t2,Atypical_Hemolytic_Uremic_Syndrome_t2
CRADD	Hereditary_Autism_t2
CRB1	Hereditary_retinopathy_t2,Retinitis_pigmentosa_t1,Leber_congenital_amaurosis,Retinitis_pigmentosa,Cone-Rod_Dystrophy_and_Related,Leber's_Congenital_Amaurosis_and_Related,Macular_dystrophy_and_Related,Retinitis_Pigmentosa_and_Related,corneal_keratoconus,retina
CRB2	Polycystic_Kidney_Disease_t2
CRBN	Hereditary_Autism_t2,Myeloma_t1
CREB3L1	Skeletal_dysplasia_t2,Osteogenesis_imperfecta
CREB3L3	Cholestasis_t2,Hereditary_Dyslipidemia_t1
CREBBP	Skeletal_dysplasia_t2,Proportionate_short_stature_t2,Hereditary_Microcephaly_t2,Hereditary_Autism_t2,Lymphoma_t1,Hematologic_malignancy,Lymphoma_B_cell_t1,Lymphoma_B_cell_t2,Myeloma_t1,glaucoma
CRH	Epilepsy_t2
CRHR1	Androgenetic_alopecia
CRIPT	Hereditary_Microcephaly_t2
CRLF1	Skeletal_dysplasia_t2
CRLF2	ALL_and_others_t2
CRPPA	Cardiomyopathy_t2,Myopathy_t2,Charcot-Marie-Tooth_disease_t2,Ataxia_t2,Muscular_dystrophy_t1,Congenital_muscular_dystrophy,Limb_girdle_muscular_dystrophy,Muscular_Dystrophy_and_Related,glaucoma
CRTAP	Dermatology_t2,Connective_tissue_disorder_t2,Skeletal_dysplasia_t1,Osteogenesis_imperfecta,Skeletal_Dysplasia_and_Related

Supplementary Table 2. List of pathogenic or likely-pathogenic germline variants

Gene	Associated diseases
CRX	Hereditary_retinopathy_t2,Retinitis_pigmentosa_t1,Leber_congenital_amaurosis,Macular_dystrophy,Retinitis_pigmentosa,Cone-Rod_Dystrophy_and_Related,Leber's_Congenital_Amaurosis_and_Related,Macular_dystrophy_and_Related,Retinitis_Pigmentosa_and_Related,corneal_keratoconus,retina
CRYAA	Hereditary_retinopathy_t2
CRYAB	Cardiomyopathy_t2,Muscular_dystrophy_t2,Hereditary_retinopathy_t2,Myopathy_t1,Dilated_cardiomyopathy,Hypertrophic_cardiomyopathy,Myofibrillar_myopathy,Myopathy_and_Related
CRYBA1	Hereditary_retinopathy_t2
CRYBA4	Hereditary_retinopathy_t2
CRYBB1	Hereditary_retinopathy_t2
CRYBB2	Hereditary_retinopathy_t2
CRYBB3	Hereditary_retinopathy_t2
CRYBG1	Lymphoma_T_NK_cell_t1,Lymphoma_T_NK_cell_t2
CRYGC	Hereditary_retinopathy_t2
CRYGD	Hereditary_retinopathy_t2
CRYGS	Hereditary_retinopathy_t2
CRYM	Hearing_loss_t2,Neurodegenerative_disease_t2,Amyotrophic_lateral_sclerosis_t2,Hereditary_hearing_loss
CSF1	ALL_and_others_t2
CSF1R	Epilepsy_t2,Alzheimer's_disease_t2,Dementia_t2,Parkinson's_disease_t2,Neurodegenerative_disease_t2,Cell-free_cancer,Sclerosing_Bone_Disease_Panel,Dementia_and_Related
CSF3R	Congenital_hematologic_disease,Hematologic_malignancy,Severe_congenital_neutropenia,ALL_and_others_t1,ALL_and_others_t2,AML,Bone_Marrow_Failure_Germline_Predisposition,MDS_MPN
CSGALNACT1	Skeletal_Dysplasia_Panel
CSNK2A1	Hereditary_Microcephaly_t2,Hereditary_Autism_t2
CSNK2B	Epilepsy_t2
CSPP1	Skeletal_dysplasia_t2,Hereditary_retinopathy_t2,retina
CSRP3	Cardiomyopathy_t2,Dilated_cardiomyopathy,Hypertrophic_cardiomyopathy
CST3	Hereditary_retinopathy_t2,Hereditary_Stroke_t1,Stroke
CSTA	Dermatology_t2
CSTB	Ataxia_t2,Epilepsy_t2
CTC1	Dermatology_t2,Epilepsy_t2,Primary_immune_deficiency_t2,Hereditary_retinopathy_t2,Hereditary_Autism_t2,Congenital_hematologic_disease,Dyskeratosis_congenita,Vitreoretinopathy,Bone_Marrow_Failure_Germline_Predisposition,Vitreoretinopathy_and_Related,retina
CTCF	Hereditary_Autism_t2
CTDP1	Charcot-Marie-Tooth_disease_t2,Ataxia_t2,Hereditary_retinopathy_t2,Charcot_Marie_Tooth_and_Related
CTF1	Cardiomyopathy_t2,Dilated_cardiomyopathy
CTLA4	Primary_immune_deficiency_t1,Inflammatory_Bowel_t1,Primary_immunodeficiency,Autoimmunity_and_autoinflammatory_disorders,B_cell_and_Humoral_Immune_Deficiency,Hemophagocytic_Lymphohistiocytosis_and_Related,Hereditary_Myeloid_Leukemia_Panel,Lymphoma_B_cell_t2
CTNNA1	Retinitis_pigmentosa_t2,Hereditary_retinopathy_t2,Macular_dystrophy,retina
CTNNA3	Arrhythmia_t2,Cardiomyopathy_t2
CTNNB1	Hereditary_spastic_paraplegia_t2,Epilepsy_t2,Hereditary_retinopathy_t2,Hereditary_Microcephaly_t2,Hereditary_Autism_t2,Neurodegenerative_disease_t2,Cell-free_cancer,Vitreoretinopathy,Circulating_tumor_DNA_assay_55,Lymphoma_T_NK_cell_t1,Lymphoma_T_NK_cell_t2,Vitreoretinopathy_and_Related,retina
CTNND2	Hereditary_Autism_t2
CTNS	Skeletal_dysplasia_t2,Lysosomal_storage_disease_t2,Nephronophthisis_t1,Ca-Pi-Mg,Corneal_Dystrophy_and_Related,Nephronophthisis_and_Related,corneal_dystrophy
CTPS1	corneal_dystrophy
CTSA	Lysosomal_storage_disease_t2,Epilepsy_t2,Hereditary_Autism_t2,Skeletal_Dysplasia_Panel
CTSC	Dermatology_t2,Lysosomal_storage_disease_t2
CTSD	Lysosomal_storage_disease_t2,Epilepsy_t2,Hereditary_retinopathy_t2,Hereditary_Autism_t2

Supplementary Table 2. List of pathogenic or likely-pathogenic germline variants

Gene	Associated diseases
CTSF	Lysosomal_storage_disease_t2,Epilepsy_t2,Hereditary_retinopathy_t2,Hereditary_Autism_t2
CTSK	Lysosomal_storage_disease_t2,Skeletal_dysplasia_t1,Sclerosing_Bone_Disease_Panel,Skeletal_Dysplasia_and_Related
CUBN	Anemia
CUL3	Acid-base-electro_gene
CUL4B	Epilepsy_t2,Hereditary_Autism_t2,Myeloma_t1
CUL7	Skeletal_dysplasia_t2,Proportionate_short_stature_t1
CUX1	Hematologic_malignancy
CWC27	Hereditary_retinopathy_t2,Retinitis_pigmentosa_t1,Leber's_Congenital_Amaurosis_and_Related,Retinitis_Pigmentosa_and_Related
CWF19L1	Ataxia_t2
CX3CR1	Hereditary_retinopathy_t2
CXCR4	Primary_immune_deficiency_t2,Lymphoma_t1,Congenital_hematologic_disease,Hematologic_malignancy,Severe_congenital_neutropenia,B_cell_and_Humoral_Immune_Deficiency,Bone_Marrow_Failure_Germline_Predisposition,Lymphoma_B_cell_t1,Lymphoma_B_cell_t2,Myeloma_t1,Phagocyte_Defect_and_Infectious_disease
CYB5R3	Hereditary_Autism_t2,Anemia_t2,Hemolytic_anemia,Anemia
CYBA	Inflammatory_Bowel_t1,Primary_immunodeficiency,Very-early-onset_inflammatory_bowel_disease,Autoimmunity_and_autoinflammatory_disorders,B_cell_and_Humoral_Immune_Deficiency,Phagocyte_Defect_and_Infectious_disease
CYBB	Primary_immune_deficiency_t2,Inflammatory_Bowel_t1,Primary_immunodeficiency,Very-early-onset_inflammatory_bowel_disease,Autoimmunity_and_autoinflammatory_disorders,B_cell_and_Humoral_Immune_Deficiency,Phagocyte_Defect_and_Infectious_disease
CYCS	Coagulation_t2,Bleeding_Platelet_Disorder
CYFIP2	Epilepsy_t2
CYP11A1	Disorders_of_sexual_development_t1,Disorders_of_sex_development
CYP11B1	Disorders_of_sex_development
CYP17A1	Disorders_of_sexual_development_t1,Disorders_of_sex_development
CYP19A1	Hypogonadotropic_hypogonadism_t2,Disorders_of_sexual_development_t1,Disorders_of_sex_development
CYP1B1	Hereditary_retinopathy_t2,glaucoma
CYP21A2	Disorders_of_sexual_development_t2,CAH_t1,Disorders_of_sex_development
CYP26B1	Craniosynostosis
CYP27A1	Inborn_error_of_metabolism_t2,Epilepsy_t2,Hereditary_retinopathy_t2,Dystonia_t2,Parkinson's_disease_t2,Hereditary_Autism_t2,Neurodegenerative_disease_t2,Charcot-Marie-Tooth_disease_t1,Ataxia_t1,Hereditary_spastic_paraplegia_t1,Hereditary_Dyslipidemia_t1,Ataxia,Neonatal_cholestasis,Ataxia_and_Related,Charcot_Marie_Tooth_and_Related,Dyslipidemia_and_Related,Spastic_Paraplegia_and_Related
CYP27B1	Skeletal_dysplasia_t2,Rickets_Hypoparathyroidism_panel,Skeletal_Dysplasia_Panel
CYP2R1	Skeletal_dysplasia_t2,Rickets_Hypoparathyroidism_panel,Skeletal_Dysplasia_Panel
CYP2U1	Ataxia_t2,Hereditary_spastic_paraplegia_t2,Neurodegenerative_disease_t2
CYP4F22	Dermatology_t1
CYP4V2	Hereditary_retinopathy_t2,Retinitis_pigmentosa_t1,Retinitis_pigmentosa,Cone-Rod_Dystrophy_and_Related,Congenital_Stationary_Night_Blindness_and_Related,Corneal_Dystrophy_and_Related,Macular_dystrophy_and_Related,Retinitis_Pigmentosa_and_Related,corneal_dystrophy,retina
CYP51A1	Hereditary_retinopathy_t2
CYP7A1	Hereditary_Dyslipidemia_t1,Neonatal_cholestasis
CYP7B1	Ataxia_t2,Neurodegenerative_disease_t2,Cholestasis_t2,Hereditary_spastic_paraplegia_t1,Hereditary_spastic_paraplegia_panel,Neonatal_cholestasis,Spastic_Paraplegia_and_Related
D2HGDH	Epilepsy_t2,Hereditary_Autism_t2
DAG1	Muscular_dystrophy_t2,Myopathy_t2,Ataxia_t2,Limb_girdle_muscular_dystrophy
DAO	Neurodegenerative_disease_t2,Amyotrophic_lateral_sclerosis_t1

Supplementary Table 2. List of pathogenic or likely-pathogenic germline variants

Gene	Associated diseases
DARS1	Hereditary_spastic_paraplegia_t2,Epilepsy_t2,Neurodegenerative_disease_t2
DARS2	Epilepsy_t2,Hereditary_Autism_t2
DBH	Arrhythmia_t2,Cardiomyopathy_t2
DBT	Hereditary_Autism_t2,Inborn_error_of_metabolism_t1,Inborn_Error_of_Metabolism
DCAF17	Hearing_loss_t2,Hypogonadotropic_hypogonadism_t2,Dystonia_t2,Parkinson's_disease_t2,Dystonia,Neurodegeneration_with_brain_iron_accumulation
DCAF8	Charcot-Marie-Tooth_disease_t2,Charcot_Marie_Tooth_and_Related
DCDC2	Hearing_loss_t2,Polycystic_Kidney_Disease_t2,Nephronophthisis_t1,Cholestasis_t1,Hereditary_hearing_loss,Nephronophthisis_and_Related
DCK	Hematologic_malignancy
DCLRE1C	Inflammatory_Bowel_t2,Primary_immune_deficiency_t1,B_cell_and_Humoral_Immune_Deficiency,Severe_Combined_Immunodeficiency_and_CID
DCN	Hereditary_retinopathy_t2,Corneal_Dystrophy_and_Related,corneal_dystrophy
DCTN1	Ataxia_t2,Alzheimer's_disease_t2,Dementia_t2,Charcot-Marie-Tooth_disease_t1,Parkinson's_disease_t1,Neurodegenerative_disease_t1,Amyotrophic_lateral_sclerosis_t1,Charcot-Marie-Tooth,Parkinson,Charcot_Marie_Tooth_and_Related,Parkinson_Disease_and_Related
DCTN2	Charcot-Marie-Tooth,Charcot_Marie_Tooth_and_Related
DCX	Epilepsy_t2,Hereditary_Autism_t2,Malformation_of_cortical_development
DDB2	Dermatology_t2
DDC	Epilepsy_t2
DDHD1	Ataxia_t2,Neurodegenerative_disease_t2,Hereditary_spastic_paraplegia_t1,Spastic_Paraplegia_and_Related
DDHD2	Ataxia_t2,Neurodegenerative_disease_t2,Hereditary_spastic_paraplegia_t1,Spastic_Paraplegia_and_Related
DDR2	Skeletal_dysplasia_t2,Cell-free_cancer,Skeletal_Dysplasia_Panel,Circulating_tumor_DNA_assay_55
DDRGK1	Skeletal_Dysplasia_Panel
DDX3X	Epilepsy_t2,Hereditary_Microcephaly_t2,Hereditary_Autism_t2,Lymphoma_t2,Lymphoma_T_NK_cell_t1,Lymphoma_T_NK_cell_t2,Myeloma_t1
DDX41	Coagulation_t2,Congenital_hematologic_disease,Hematologic_malignancy,Leukemia_predisposition,ALL_and_others_t1,ALL_and_others_t2,AML,Bone_Marrow_Failure_Germline_Predisposition,Hereditary_Myeloid_Leukemia_Panel,MDS_MPN
DDX58	Hereditary_retinopathy_t2,Osteogenesis_imperfecta,glaucoma
DECR1	Inborn_error_of_metabolism_t2
DEGS1	Epilepsy_t2
DENND5A	Epilepsy_t2
DEPDC5	Arrhythmia_t2,Epilepsy_t2,Focal_epilepsy,Epilepsy_and_Related
DES	Arrhythmia_t2,Cardiomyopathy_t2,Muscular_dystrophy_t1,Myopathy_t1,Arrhythmia,Dilated_cardiomyopathy,Limb_girdle_muscular_dystrophy,Myofibrillar_myopathy,Arrhythmia_and_Related,Muscular_Dystrophy_and_Related,Myopathy_and_Related
DGAT1	Congenital_diarrhea
DGAT2	Charcot-Marie-Tooth,Charcot_Marie_Tooth_and_Related
DGKE	Atypical_Hemolytic_Uremic_Syndrome_t1,DNAJC21_EFL1_SBDS_SRP54,Atypical_Hemolytic_Uremic_Syndrome,Bleeding_Coagulopathy,Thrombosis
DGUOK	Cholestasis_t2,Neonatal_cholestasis
DHCR24	Skeletal_dysplasia_t2,Hereditary_Autism_t2
DHCR7	Skeletal_dysplasia_t2,Proportionate_short_stature_t2,Lysosomal_storage_disease_t2,Inborn_error_of_metabolism_t2,Epilepsy_t2,Hereditary_Microcephaly_t2,Disorders_of_sexual_development_t1,Hereditary_Autism_t1,Disorders_of_sex_development,Neonatal_cholestasis,Autism_and_Related
DHDDS	Hereditary_retinopathy_t2,Retinitis_pigmentosa_t1,Retinitis_pigmentosa,Retinitis_Pigmentosa_and_Related,retina
DHFR	Epilepsy_t2,Anemia
DHH	Disorders_of_sexual_development_t2,Disorders_of_sex_development,Charcot_Marie_Tooth_and_Related
DHODH	Skeletal_dysplasia_t2
DHPS	Ataxia_t2,Epilepsy_t2

Supplementary Table 2. List of pathogenic or likely-pathogenic germline variants

Gene	Associated diseases
DHTKD1	Charcot-Marie-Tooth_disease_t2,Charcot-Marie-Tooth,Charcot_Marie_Tooth_and_Related
DHX15	Hematologic_malignancy
DHX38	Hereditary_retinopathy_t2,Retinitis_pigmentosa_t1,Retinitis_pigmentosa,Retinitis_Pigmentosa_and_Related,retina
DIABLO	Hearing_loss_t2,Hereditary_hearing_loss
DIAPH1	Epilepsy_t2,Hereditary_Microcephaly_t2,Hearing_loss_t1,Hereditary_hearing_loss,Hearing_Loss_and_Related_t1,Hearing_Loss_and_Related_t2
DIAPH3	Hearing_loss_t2,Hereditary_hearing_loss
DICER1	Hereditary_pancancer,Germline_Cancer
DIS3	Hematologic_malignancy,Myeloma_t1
DIS3L2	Hereditary_pancancer
DKC1	Dermatology_t2,Coagulation_t2,Primary_immune_deficiency_t2,Hereditary_Autism_t2,Inflammatory_Bowel_t2,Congenital_hematologic_disease,Dyskeratosis_congenita,ALL_and_others_t2,AML,Bone_Marrow_Failure_Germline_Predisposition,MDS_MPN
DLAT	Dystonia_t2,Parkinson's_disease_t2
DLD	Inborn_error_of_metabolism_t2,Epilepsy_t2
DLG3	Epilepsy_t2,Hereditary_Autism_t2
DLG4	Connective_tissue_disorder_t2
DLGAP2	Hereditary_Autism_t2
DLL3	Skeletal_dysplasia_t2
DLL4	Dermatology_t2,Skeletal_dysplasia_t2
DLX3	Skeletal_dysplasia_t2,Sclerosing_Bone_Disease_Panel
DLX5	Skeletal_dysplasia_t2,Hearing_loss_t2
DMD	Cardiomyopathy_t2,Myopathy_t2,Hereditary_Autism_t2,Muscular_dystrophy_t1,Dilated_cardiomyopathy,Limb_girdle_muscular_dystrophy,Muscular_Dystrophy_and_Related,retina
DMP1	Skeletal_dysplasia_t2,Rickets_Hypoparathyroidism_panel,Skeletal_Dysplasia_Panel
DMPK	Charcot_Marie_Tooth_and_Related,corneal_dystrophy
DMRT1	Disorders_of_sexual_development_t2,Disorders_of_sex_development
DMRT2	Disorders_of_sexual_development_t2,Disorders_of_sex_development
DMTN	Hemolytic_anemia
DMXL2	Hearing_loss_t2,Hypogonadotropic_hypogonadism_t2,Hereditary_hearing_loss
DNAAF1	Hereditary_Primary_Ciliary_Dyskinesia_t1
DNAAF11	Hereditary_Primary_Ciliary_Dyskinesia_t1
DNAAF2	Hereditary_Primary_Ciliary_Dyskinesia_t1
DNAAF3	Hereditary_Primary_Ciliary_Dyskinesia_t1
DNAAF4	Hereditary_Primary_Ciliary_Dyskinesia_t1
DNAAF5	Hereditary_Primary_Ciliary_Dyskinesia_t1
DNAAF6	Hereditary_Primary_Ciliary_Dyskinesia_t2
DNAH1	Hereditary_Primary_Ciliary_Dyskinesia_t1
DNAH11	Hereditary_Primary_Ciliary_Dyskinesia_t1
DNAH5	Hereditary_Primary_Ciliary_Dyskinesia_t1
DNAH6	Hereditary_Primary_Ciliary_Dyskinesia_t2
DNAH8	Hereditary_Primary_Ciliary_Dyskinesia_t1
DNAH9	Hereditary_Primary_Ciliary_Dyskinesia_t2
DNAI1	Hereditary_Primary_Ciliary_Dyskinesia_t1
DNAI2	Hereditary_Primary_Ciliary_Dyskinesia_t1

Supplementary Table 2. List of pathogenic or likely-pathogenic germline variants

Gene	Associated diseases
DNAJB11	Polycystic_Kidney_Disease_t1,Polycystic_kidney_disease_and_Related
DNAJB13	Hereditary_Primary_Ciliary_Dyskinesia_t2
DNAJB2	Charcot-Marie-Tooth_disease_t1,Charcot-Marie-Tooth,Charcot_Marie_Tooth_and_Related
DNAJB5	Charcot_Marie_Tooth_and_Related
DNAJB6	Muscular_dystrophy_t1,Myopathy_t1,Limb_girdle_muscular_dystrophy,Myofibrillar_myopathy,Muscular_Dystrophy_and_Related,Myopathy_and_Related
DNAJC12	Dystonia_t2
DNAJC13	Parkinson_Disease_and_Related
DNAJC19	Cardiomyopathy_t2,Ataxia_t2,Hereditary_retinopathy_t2,Optic_neuropathy_and_Related
DNAJC21	Congenital_hematologic_disease,Shwachman-Diamond_syndrome,Skeletal_Dysplasia_Panel,Bone_Marrow_Failure_Germline_Predisposition,Hereditary_Myeloid_Leukemia_Panel
DNAJC3	Charcot_Marie_Tooth_and_Related
DNAJC5	Lysosomal_storage_disease_t2,Epilepsy_t2,Hereditary_retinopathy_t2,Dystonia_t2,Parkinson's_disease_t2
DNAJC6	Dystonia_t2,Dementia_t2,Parkinson's_disease_t2,Neurodegenerative_disease_t2,Parkinson
DNAL1	Hereditary_Primary_Ciliary_Dyskinesia_t1
DNM1	Epilepsy_t2,Hereditary_Autism_t2,Epilepsy_and_Related
DNM1L	Epilepsy_t2,Hereditary_retinopathy_t2,Hereditary_Microcephaly_t2,Optic_neuropathy_and_Related
DNM2	Muscular_dystrophy_t2,Ataxia_t2,Myopathy_t1,Charcot-Marie-Tooth_disease_t1,Charcot-Marie-Tooth,Congenital_muscular_dystrophy,Congenital_myopathy,Charcot_Marie_Tooth_and_Related,Myopathy_and_Related
DNMT1	Ataxia_t2,Hearing_loss_t2,Alzheimer's_disease_t2,Dementia_t2,Parkinson's_disease_t2,Neurodegenerative_disease_t2,Charcot-Marie-Tooth_disease_t1,Charcot-Marie-Tooth,Charcot_Marie_Tooth_and_Related
DNMT3A	Hereditary_Autism_t2,Hematologic_malignancy,Overgrowth_intellectual_disability,Overgrowth_Panel,ALL_and_others_t1,ALL_and_others_t2,AML,Lymphoma_T_NK_cell_t1,Lymphoma_T_NK_cell_t2,MDS_MPN,Myeloma_t1
DNMT3B	Primary_immune_deficiency_t2
DOCK2	B_cell_and_Humoral_Immune_Deficiency,Severe_Combined_Immunodeficiency_and_CID
DOCK3	Ataxia_t2
DOCK4	Hereditary_Autism_t2
DOCK6	Dermatology_t2,Skeletal_dysplasia_t2
DOCK7	Epilepsy_t2
DOCK8	Dermatology_t2,Primary_immune_deficiency_t1,Inflammatory_Bowel_t1,Primary_immunodeficiency,Very-early-onset_inflammatory_bowel_disease,Autoimmunity_and_autoinflammatory_disorders,B_cell_and_Humoral_Immune_Deficiency,Hereditary_Myeloid_Leukemia_Panel,Phagocyte_Defect_and_Infectious_disease,Severe_Combined_Immunodeficiency_and_CID
DOCK9	corneal_keratoconus
DOK7	Muscular_dystrophy_t2,Myopathy_t2,Congenital_myopathy
DOLK	Cardiomyopathy_t2,Epilepsy_t2
DONSON	Skeletal_dysplasia_t2,Proportionate_short_stature_t2,Hereditary_Microcephaly_t2
DPAGT1	Epilepsy_t2
DPCD	Skeletal_dysplasia_t2
DPM1	Muscular_dystrophy_t2,Myopathy_t2,Epilepsy_t2,Congenital_muscular_dystrophy
DPM2	Muscular_dystrophy_t2,Myopathy_t2,Epilepsy_t2,Congenital_muscular_dystrophy
DPM3	Cardiomyopathy_t2,Muscular_dystrophy_t2,Myopathy_t2,Limb_girdle_muscular_dystrophy
DPP10	Hereditary_Autism_t1
DPP6	Hereditary_Autism_t2
DPYD	Lysosomal_storage_disease_t2,Epilepsy_t2,Hereditary_Autism_t2,Cell-free_cancer

Supplementary Table 2. List of pathogenic or likely-pathogenic germline variants

Gene	Associated diseases
DPYS	Epilepsy_t2
DRAM2	Hereditary_retinopathy_t2,Macular_dystrophy_and_Related,retina
DRC1	Hereditary_Primary_Ciliary_Dyskinesia_t1
DRD2	Dystonia_t2
DRD5	Dystonia_t2
DRP2	Charcot-Marie-Tooth,Charcot_Marie_Tooth_and_Related
DSC2	Arrhythmia_t2,Cardiomyopathy_t1,Arrhythmia,Arrhythmia_and_Related,Cardiomyopathy_and_Related
DSE	Dermatology_t2,Connective_tissue_disorder_t2,Skeletal_Dysplasia_Panel
DSG1	Dermatology_t2
DSG2	Arrhythmia_t2,Dermatology_t2,Cardiomyopathy_t1,Arrhythmia,Dilated_cardiomyopathy,Arrhythmia_and_Related,Cardiomyopathy_and_Related
DSG4	Dermatology_t2
DSP	Arrhythmia_t2,Cardiomyopathy_t1,Dermatology_t1,Arrhythmia,Dilated_cardiomyopathy,Arrhythmia_and_Related,Cardiomyopathy_and_Related
DSPP	Hearing_loss_t2,Hereditary_hearing_loss
DST	Charcot-Marie-Tooth_disease_t2,Dermatology_t1,Charcot_Marie_Tooth_and_Related
DSTYK	Hereditary_spastic_paraplegia_t2,Neurodegenerative_disease_t2
DTHD1	Hereditary_retinopathy_t2
DTNA	Cardiomyopathy_t2,Hypertrophic_cardiomyopathy
DTNBP1	Dermatology_t2,Coagulation_t2,Hereditary_retinopathy_t2,Albinism_and_Related,Bleeding_Platelet_Disorder
DUOX2	Hypothyroidism_t1,Congenital_Hypothyroidism
DUOXA2	Hypothyroidism_t1,Congenital_Hypothyroidism
DUSP22	Lymphoma_B_cell_t2
DUSP6	Hypogonadotropic_hypogonadism_t2
DUX4	Limb_girdle_muscular_dystrophy
DVL1	Skeletal_dysplasia_t2,Proportionate_short_stature_t2,Skeletal_Dysplasia_Panel
DVL3	Skeletal_dysplasia_t2,Skeletal_Dysplasia_Panel
DYM	Skeletal_dysplasia_t2,Lysosomal_storage_disease_t2,Skeletal_Dysplasia_Panel,Spondyloepiphyseal_metaphyseal_dysplasia
DYNC1H1	Muscular_dystrophy_t2,Myopathy_t2,Ataxia_t2,Epilepsy_t2,Hereditary_Microcephaly_t2,Hereditary_Autism_t2,Charcot-Marie-Tooth_disease_t1,Charcot-Marie-Tooth,Malformation_of_cortical_development,Charcot_Marie_Tooth_and_Related
DYNC1I1	Skeletal_dysplasia_t2
DYNC2H1	Skeletal_dysplasia_t2,Skeletal_Dysplasia_Panel,Spondyloepiphyseal_metaphyseal_dysplasia
DYNC2I1	Skeletal_dysplasia_t2,Skeletal_Dysplasia_Panel
DYNC2I2	Skeletal_dysplasia_t2,Skeletal_Dysplasia_Panel
DYNC2LI1	Skeletal_Dysplasia_Panel
DYNLT2B	Skeletal_dysplasia_t2,Skeletal_Dysplasia_Panel
DYRK1A	Epilepsy_t2,Hereditary_Microcephaly_t2,Hereditary_Autism_t2,Autism_and_Related
DYSF	Cardiomyopathy_t2,Myopathy_t2,Ataxia_t2,Muscular_dystrophy_t1,Limb_girdle_muscular_dystrophy,Muscular_Dystrophy_and_Related
DZIP1L	Polycystic_Kidney_Disease_t1,Polycystic_kidney_disease_and_Related
EARS2	Epilepsy_t2
EBF1	Androgenetic_alopecia,ALL_and_others_t2
EBF3	Ataxia_t2
EBP	Dermatology_t2,Hereditary_Autism_t2,Skeletal_dysplasia_t1,Skeletal_Dysplasia_Panel,Spondyloepiphyseal_metaphyseal_dysplasia,Skeletal_Dysplasia_and_Related
ECE1	Hirschsprung's_disease

Supplementary Table 2. List of pathogenic or likely-pathogenic germline variants

Gene	Associated diseases
ECHS1	Epilepsy_t2
ECM1	Dermatology_t2,Epilepsy_t2
EDA	Dermatology_t2,Ectodermal_dysplasia_Hypodontia
EDA2R	Androgenetic_alopecia
EDAR	Dermatology_t2,Ectodermal_dysplasia_Hypodontia
EDARADD	Dermatology_t2,Ectodermal_dysplasia_Hypodontia
EDN3	Dermatology_t2,Hearing_loss_t2,Hereditary_retinopathy_t2,Hereditary_hearing_loss,Hirschsprung's_disease,Albinism_and_Related
EDNRB	Dermatology_t2,Hereditary_retinopathy_t2,Hearing_loss_t1,Hereditary_hearing_loss,Hirschsprung's_disease,Albinism_and_Related,Hearing_Loss_and_Related_t1,Hearing_Loss_and_Related_t2
EED	Overgrowth_intellectual_disability
EEF1A2	Cardiomyopathy_t2,Epilepsy_t2
EEF2	Ataxia_t2
EFEMP1	Hereditary_retinopathy_t2,Macular_dystrophy_and_Related,Retinitis_Pigmentosa_and_Related,retina
EFEMP2	Dermatology_t2,Connective_tissue_disorder_t2
EFHC1	Epilepsy_t2
EFL1	Coagulation_t2,Congenital_hematologic_disease,Shwachman-Diamond_syndrome,Skeletal_Dysplasia_Panel,Hereditary_Myeloid_Leukemia_Panel
EFNB1	Skeletal_dysplasia_t2,Hereditary_Autism_t2,Craniosynostosis
EFTUD2	Skeletal_dysplasia_t2,Hereditary_Autism_t2,Hereditary_Microcephaly_t1
EGF	Atypical_Hemolytic_Uremic_Syndrome_t2,Ca-Pi-Mg
EGFR	Cell-free_cancer,Hereditary_pancancer,Circulating_tumor_DNA_assay_55,Lymphoma_B_cell_t2,Lymphoma_T_NK_cell_t2
EGLN1	Coagulation_t2,Hereditary_cancer_syndrome_Plus_t2,Neuroendocrine_tumor_t1,Hereditary_paraganglioma-pheochromocytoma_syndrome,Erythrocytosis
EGLN2	Hereditary_cancer_syndrome_Plus_t2,Neuroendocrine_tumor_t1,Hereditary_paraganglioma-pheochromocytoma_syndrome,Erythrocytosis
EGR1	Myeloma_t1
EGR2	Ataxia_t2,Charcot-Marie-Tooth_disease_t1,Lymphoma_t1,Charcot-Marie-Tooth,Charcot_Marie_Tooth_and_Related
EHMT1	Epilepsy_t2,Hereditary_Autism_t2
EIF2AK3	Skeletal_dysplasia_t2,Maturity-Onset_Diabetes_of_the_Young,_MODY_t1,Monogenic_diabetes_panel,Skeletal_Dysplasia_Panel
EIF2AK4	Primary_Pulmonary_Hypertension_and_Related_Disordisorders
EIF2B1	Ataxia_t2,Epilepsy_t2
EIF2B2	Ataxia_t2,Epilepsy_t2
EIF2B3	Ataxia_t2,Epilepsy_t2
EIF2B4	Ataxia_t2,Epilepsy_t2
EIF2B5	Ataxia_t2,Epilepsy_t2
EIF2S3	Hereditary_Autism_t2
EIF3F	Epilepsy_t2,Hearing_loss_t2
EIF4G1	Alzheimer's_disease_t2,Dementia_t2,Parkinson's_disease_t2,Neurodegenerative_disease_t2,Parkinson,Parkinson_Disease_and_Related
ELAC2	Cardiomyopathy_t2
ELANE	Coagulation_t2,Congenital_hematologic_disease,Severe_congenital_neutropenia,ALL_and_others_t2,Bone_Marrow_Failure_Germline_Predisposition,MDS_MPN
ELK1	Hereditary_Autism_t2
ELMOD3	Hearing_loss_t2,Hereditary_hearing_loss
ELN	Dermatology_t2,Connective_tissue_disorder_t1,Connective_Tissue_Disorder_and_Related
ELOVL1	retina
ELOVL4	Dermatology_t2,Ataxia_t2,Hereditary_retinopathy_t2,Hereditary_Autism_t2,Macular_dystrophy,Macular_dystrophy_and_Related,Retinitis_Pigmentosa_and_Related,retina
ELOVL5	Ataxia_t2

Supplementary Table 2. List of pathogenic or likely-pathogenic germline variants

Gene	Associated diseases
ELP1	Charcot-Marie-Tooth_disease_t2,Charcot_Marie_Tooth_and_Related
EMC1	Hereditary_retinopathy_t2,Retinitis_pigmentosa_t1,Retinitis_pigmentosa,Retinitis_Pigmentosa_and_Related,retina
EMD	Arrhythmia_t2,Cardiomyopathy_t2,Myopathy_t2,Muscular_dystrophy_t1,Dilated_cardiomyopathy,Limb_girdle_muscular_dystrophy,Muscular_Dystrophy_and_Related
EMILIN1	Charcot_Marie_Tooth_and_Related
EMP2	Glomerulopathy
EMX2	Epilepsy_t2,Malformation_of_cortical_development
EN2	Hereditary_Autism_t2
ENAM	Skeletal_dysplasia_t2
ENG	Hereditary_colon_cancer,Primary_Pulmonary_Hypertension_and_Related_Disordisorders
ENO1	Hemolytic_anemia
ENO3	Myopathy_t2,Metabolic_myopathy
ENPP1	Dermatology_t2,Skeletal_dysplasia_t2,Connective_tissue_disorder_t2,Rickets_Hypoparathyroidism_panel,Skeletal_Dysplasia_Panel
ENTPD1	Hereditary_spastic_paraplegia_t2,Neurodegenerative_disease_t2
EOGT	Dermatology_t2,Skeletal_dysplasia_t2
EP300	Skeletal_dysplasia_t2,Proportionate_short_stature_t2,Hereditary_Microcephaly_t2,Hereditary_Autism_t2,Lymphoma_t1,Hematologic_malignancy,ALL_and_others_t1,ALL_and_others_t2,Lymphoma_B_cell_t1,Lymphoma_B_cell_t2,Lymphoma_T_NK_cell_t1,Lymphoma_T_NK_cell_t2
EPAS1	Coagulation_t2,Hereditary_cancer_syndrome_Plus_t2,Neuroendocrine_tumor_t1,Hereditary_paraganglioma-pheochromocytoma_syndrome,Erythrocytosis
EPB41	Anemia_t1,Hemolytic_anemia,Anemia
EPB42	Anemia_t1,Hemolytic_anemia,Anemia
EPCAM	Dermatology_t2,Cholestasis_t2,Inflammatory_Bowel_t2,Hereditary_cancer_syndrome_Plus_t1,Congenital_diarrhea,Congenital_hematologic_disease,Hereditary_colon_cancer,Hereditary_pancancer,Leukemia_predisposition,Breast_and_Ovarian_Cancer_Panel,Colorectal_Cancer_Polyposis_Panel
EPG5	Cardiomyopathy_t2,Hereditary_retinopathy_t2
EPHA2	Hereditary_retinopathy_t2
EPHB4	Rasopathies_t2
EPHX1	Skeletal_dysplasia_t2,Cholestasis_t2
EPM2A	Epilepsy_t2,Epilepsy_and_Related
EPOR	ALL_and_others_t2,Erythrocytosis
EPRS1	Epilepsy_t2,Hereditary_Microcephaly_t2
EPS8	Hearing_loss_t2,Hereditary_hearing_loss
EPS8L2	Hearing_loss_t2,Hereditary_hearing_loss
EPYC	corneal_dystrophy
ERAL1	Hereditary_hearing_loss
ERBB2	Cell-free_cancer,Circulating_tumor_DNA_assay_55
ERBB3	Cell-free_cancer,Circulating_tumor_DNA_assay_55
ERBB4	Neurodegenerative_disease_t2,Amyotrophic_lateral_sclerosis_t2,Amyotrophic_lateral_sclerosis
ERCC1	Dermatology_t2
ERCC2	Dermatology_t2,Hearing_loss_t2,Hereditary_Autism_t2
ERCC3	Dermatology_t2,Hearing_loss_t2,Hereditary_Autism_t2
ERCC4	Dermatology_t2,Skeletal_dysplasia_t2,Coagulation_t2,Hereditary_Autism_t2,Congenital_hematologic_disease,Bone_Marrow_Failure_Germline_Predisposition
ERCC5	Dermatology_t2,Hereditary_Autism_t2
ERCC6	Dermatology_t2,Hereditary_retinopathy_t2,Hereditary_Autism_t2
ERCC6L2	Hereditary_Myeloid_Leukemia_Panel

Supplementary Table 2. List of pathogenic or likely-pathogenic germline variants

Gene	Associated diseases
ERCC8	Dermatology_t2,Hereditary_Autism_t2
ERF	Craniosynostosis
ERG	ALL_and_others_t2,Circulating_tumor_DNA_assay_55
ERLIN1	Hereditary_spastic_paraplegia_t2,Neurodegenerative_disease_t2
ERLIN2	Ataxia_t2,Neurodegenerative_disease_t2,Hereditary_spastic_paraplegia_t1,Spastic_Paraplegia_and_Related
ESCO2	Skeletal_dysplasia_t2
ESPN	Hearing_loss_t2,Hereditary_retinopathy_t2,Hereditary_hearing_loss,Hearing_Loss_and_Related_t2,retina
ESR1	Hypogonadotropic_hypogonadism_t2,Cell-free_cancer,Circulating_tumor_DNA_assay_55
ESRP1	Hereditary_hearing_loss
ESRRB	Hearing_loss_t2,Hereditary_hearing_loss,Hearing_Loss_and_Related_t2
ETFA	Cardiomyopathy_t2,Myopathy_t2,Lysosomal_storage_disease_t2,Inborn_error_of_metabolism_t2,Epilepsy_t2,Polycystic_Kidney_Disease_t1,Metabolic_myopathy,Polycystic_kidney_disease_and_Related
ETFB	Cardiomyopathy_t2,Myopathy_t2,Lysosomal_storage_disease_t2,Inborn_error_of_metabolism_t2,Epilepsy_t2,Metabolic_myopathy
ETFDH	Cardiomyopathy_t2,Myopathy_t2,Lysosomal_storage_disease_t2,Inborn_error_of_metabolism_t2,Epilepsy_t2,Metabolic_myopathy
ETHE1	Epilepsy_t2
ETNK1	Hematologic_malignancy
ETV1	Circulating_tumor_DNA_assay_55
ETV6	Coagulation_t2,Lymphoma_t2,Congenital_hematologic_disease,Hematologic_malignancy,Leukemia_predisposition,ALL_and_others_t1,ALL_and_others_t2,AML,Bleeding_Platelet_Disorder,Bone_Marrow_Failure_Germline_Predisposition,Hereditary_Myeloid_Leukemia_Panel,MDS_MPN
EVC	Dermatology_t2,Skeletal_dysplasia_t2,Skeletal_Dysplasia_Panel,Spondyloepiphyseal_metaphyseal_dysplasia
EVC2	Dermatology_t2,Skeletal_dysplasia_t2,Spondyloepiphyseal_metaphyseal_dysplasia
EXOC6B	Skeletal_Dysplasia_Panel
EXOSC2	Retinitis_Pigmentosa_and_Related
EXOSC3	Hereditary_spastic_paraplegia_t2,Hereditary_Microcephaly_t2,Neurodegenerative_disease_t2
EXPH5	Dermatology_t1
EXT1	Proportionate_short_stature_t2,Skeletal_dysplasia_t1,Skeletal_Dysplasia_and_Related
EXT2	Skeletal_dysplasia_t1,Skeletal_Dysplasia_and_Related
EXTL3	Skeletal_dysplasia_t2,Skeletal_Dysplasia_Panel
EYA1	Hereditary_retinopathy_t2,Polycystic_Kidney_Disease_t2,Hearing_loss_t1,Hereditary_hearing_loss,Hearing_Loss_and_Related_t1,Hearing_Loss_and_Related_t2
EYA4	Cardiomyopathy_t2,Hearing_loss_t2,Dilated_cardiomyopathy,Hereditary_hearing_loss,Hearing_Loss_and_Related_t2
EYS	Hereditary_retinopathy_t2,Retinitis_pigmentosa_t1,Retinitis_pigmentosa,Retinitis_Pigmentosa_and_Related,retina
EZH2	Skeletal_dysplasia_t2,Hereditary_Autism_t2,Lymphoma_t1,Hematologic_malignancy,Overgrowth_intellectual_disability,Overgrowth_Panel,ALL_and_others_t1,ALL_and_others_t2,AML,Bone_Marrow_Failure_Germline_Predisposition,Lymphoma_B_cell_t1,Lymphoma_B_cell_t2,Lymphoma_T_NK_cell_t1,Lymphoma_T_NK_cell_t2,MDS_MPN,Myeloma_t1
F10	Coagulation_t1,Atypical_Hemolytic_Uremic_Syndrome,Bleeding_Coagulopathy,Thrombosis
F11	Coagulation_t1,Atypical_Hemolytic_Uremic_Syndrome,Bleeding_Coagulopathy,Thrombosis
F12	Coagulation_t2,Atypical_Hemolytic_Uremic_Syndrome,Bleeding_Coagulopathy,Thrombosis
F13A1	Coagulation_t1,Atypical_Hemolytic_Uremic_Syndrome,Bleeding_Coagulopathy,Thrombosis
F13B	Coagulation_t1,Atypical_Hemolytic_Uremic_Syndrome,Bleeding_Coagulopathy,Thrombosis
F2	Hereditary_Stroke_t2,Coagulation_t1,Stroke,Atypical_Hemolytic_Uremic_Syndrome,Bleeding_Coagulopathy,Thrombosis
F5	Hereditary_Stroke_t2,Coagulation_t1,Stroke,Atypical_Hemolytic_Uremic_Syndrome,Bleeding_Coagulopathy,Thrombosis
F7	Coagulation_t1,Stroke,Atypical_Hemolytic_Uremic_Syndrome,Bleeding_Coagulopathy,Thrombosis
F8	Coagulation_t1,Atypical_Hemolytic_Uremic_Syndrome,Bleeding_Coagulopathy,Thrombosis
F9	Coagulation_t1,Atypical_Hemolytic_Uremic_Syndrome,Bleeding_Coagulopathy,Thrombosis

Supplementary Table 2. List of pathogenic or likely-pathogenic germline variants

Gene	Associated diseases
FA2H	Ataxia_t2,Epilepsy_t2,Dystonia_t2,Parkinson's_disease_t2,Neurodegenerative_disease_t2,Hereditary_spastic_paraplegia_t1,Chorea,Dystonia,Neurodegeneration_with_brain_iron_accumulation,Spastic_Paraplegia_and_Related
FAAH2	Hereditary_Autism_t2
FABP3	corneal_dystrophy
FADD	Familial_hemophagocytic_lymphohistiocytosis,Autoimmunity_and_autoinflammatory_disorders,B_cell_and_Humoral_Immune_Deficiency,Hemophagocytic_Lymphohistiocytosis_and_Related
FAH	Skeletal_dysplasia_t2,Cholestasis_t2,Inborn_error_of_metabolism_t1,Neonatal_cholestasis,Inborn_Error_of_Metabolism
FAM111A	Skeletal_dysplasia_t2,Atypical_Hemolytic_Uremic_Syndrome_t2,Rickets_Hypoparathyroidism_panel
FAM126A	Epilepsy_t2,Hereditary_retinopathy_t2,Hereditary_Autism_t2
FAM136A	Hearing_loss_t2
FAM161A	Hereditary_retinopathy_t2,Retinitis_pigmentosa_t1,Retinitis_pigmentosa,Retinitis_Pigmentosa_and_Related,retina
FAM20A	Skeletal_dysplasia_t2
FAM20B	Skeletal_Dysplasia_Panel
FAM20C	Skeletal_dysplasia_t2,Rickets_Hypoparathyroidism_panel
FAM83H	Skeletal_dysplasia_t2
FAN1	Nephronophthisis_t1,Nephronophthisis_and_Related
FANCA	Dermatology_t2,Skeletal_dysplasia_t2,Coagulation_t2,Anemia_t2,Congenital_hematologic_disease,Fanconi_anemia,Hereditary_pancancer,ALL_and_others_t2,Bone_Marrow_Failure_Germline_Predisposition
FANCB	Dermatology_t2,Skeletal_dysplasia_t2,Coagulation_t2,Hereditary_Autism_t2,Congenital_hematologic_disease,Fanconi_anemia,Hereditary_pancancer,Bone_Marrow_Failure_Germline_Predisposition
FANCC	Dermatology_t2,Skeletal_dysplasia_t2,Coagulation_t2,Anemia_t2,Congenital_hematologic_disease,Fanconi_anemia,Hereditary_breast_cancer,Hereditary_pancancer,ALL_and_others_t2,Bone_Marrow_Failure_Germline_Predisposition
FANCD2	Dermatology_t2,Skeletal_dysplasia_t2,Coagulation_t2,Congenital_hematologic_disease,Fanconi_anemia,Hereditary_pancancer,Bone_Marrow_Failure_Germline_Predisposition
FANCE	Dermatology_t2,Skeletal_dysplasia_t2,Coagulation_t2,Congenital_hematologic_disease,Fanconi_anemia,Hereditary_pancancer,Bone_Marrow_Failure_Germline_Predisposition
FANCF	Dermatology_t2,Skeletal_dysplasia_t2,Coagulation_t2,Congenital_hematologic_disease,Fanconi_anemia,Hereditary_pancancer,Bone_Marrow_Failure_Germline_Predisposition
FANCG	Dermatology_t2,Skeletal_dysplasia_t2,Coagulation_t2,Hereditary_Autism_t2,Anemia_t2,Congenital_hematologic_disease,Fanconi_anemia,Hereditary_pancancer,ALL_and_others_t2,Bone_Marrow_Failure_Germline_Predisposition
FANCI	Dermatology_t2,Skeletal_dysplasia_t2,Coagulation_t2,Congenital_hematologic_disease,Fanconi_anemia,Hereditary_pancancer,Bone_Marrow_Failure_Germline_Predisposition
FANCL	Dermatology_t2,Skeletal_dysplasia_t2,Coagulation_t2,Congenital_hematologic_disease,Fanconi_anemia,Hereditary_pancancer,Bone_Marrow_Failure_Germline_Predisposition
FANCM	Dermatology_t2,Skeletal_dysplasia_t2,Coagulation_t2,Congenital_hematologic_disease,Fanconi_anemia,Bone_Marrow_Failure_Germline_Predisposition
FAR1	Epilepsy_t2,Skeletal_Dysplasia_Panel
FARS2	Hereditary_spastic_paraplegia_t2,Epilepsy_t2,Neurodegenerative_disease_t2
FAS	Lymphoma_t1,Familial_hemophagocytic_lymphohistiocytosis,B_cell_and_Humoral_Immune_Deficiency,Autoimmunity_and_autoinflammatory_disorders,Hemophagocytic_Lymphohistiocytosis_and_Related,Lymphoma_T_NK_cell_t2
FASLG	Familial_hemophagocytic_lymphohistiocytosis,B_cell_and_Humoral_Immune_Deficiency,Autoimmunity_and_autoinflammatory_disorders,Hemophagocytic_Lymphohistiocytosis_and_Related,corneal_dystrophy
FASN	Epilepsy_t2
FAT4	Lymphoma_t1
FBLN1	Skeletal_dysplasia_t2
FBLN5	Dermatology_t2,Charcot-Marie-Tooth_disease_t2,Hereditary_retinopathy_t2,Connective_tissue_disorder_t1,Charcot_Marie_Tooth_and_Related,Connective_Tissue_Disorder_and_Related
FBN1	Dermatology_t2,Skeletal_dysplasia_t2,Proportionate_short_stature_t2,Hereditary_retinopathy_t2,Hereditary_Autism_t2,Connective_tissue_disorder_t1,Hereditary_Stroke_t1,Familial_thoracic_aortic_aneurysms_and_aortic_dissections,Overgrowth_Panel,Skeletal_Dysplasia_Panel,Stroke,Connective_Tissue_Disorder_and_Related,Marfan_and_Related,glaucoma
FBN2	Dermatology_t2,Skeletal_dysplasia_t2,Connective_tissue_disorder_t1,Familial_thoracic_aortic_aneurysms_and_aortic_dissections,Overgrowth_Panel,Connective_Tissue_Disorder_and_Related
FBXL4	Cardiomyopathy_t2,Ataxia_t2

Supplementary Table 2. List of pathogenic or likely-pathogenic germline variants

Gene	Associated diseases
FBXO11	Hereditary_Autism_t2,Lymphoma_t1
FBXO32	Cardiomyopathy_t2
FBXO38	Charcot_Marie_Tooth_and_Related
FBXO7	Alzheimer's_disease_t2,Dystonia_t2,Dementia_t2,Parkinson's_disease_t2,Neurodegenerative_disease_t2,Dystonia,Parkinson,Parkinson_Disease_and_Related
FBXW4	Skeletal_dysplasia_t2
FBXW7	Cell-free_cancer,Hematologic_malignancy,ALL_and_others_t2,Circulating_tumor_DNA_assay_55
FCGR3A	Phagocyte_Defect_and_Infectious_disease
FDFT1	Epilepsy_t2
FDX2	Muscular_dystrophy_t2,Myopathy_t2,Epilepsy_t2
FDXR	Ataxia_t2,Hearing_loss_t2,Hereditary_retinopathy_t2
FECH	Dermatology_t2
FEN1	corneal_dystrophy
FERMT1	Inflammatory_Bowel_t2,Dermatology_t1,Very-early-onset_inflammatory_bowel_disease
FERMT2	Coagulation_t2
FERMT3	Skeletal_dysplasia_t2,Coagulation_t2,Primary_immunodeficiency,Sclerosing_Bone_Disease_Panel
FEZF1	Hypogonadotropic_hypogonadism_t2
FGA	Coagulation_t1,Stroke,Atypical_Hemolytic_Uremic_Syndrome,Bleeding_Coagulopathy,Thrombosis
FGB	Coagulation_t1,Stroke,Atypical_Hemolytic_Uremic_Syndrome,Bleeding_Coagulopathy,Thrombosis
FGD1	Skeletal_dysplasia_t2,Proportionate_short_stature_t2,Rasopathies_t2,Epilepsy_t2,Hereditary_Autism_t2
FGD4	Ataxia_t2,Charcot-Marie-Tooth_disease_t1,Charcot-Marie-Tooth,Charcot_Marie_Tooth_and_Related
FGF10	Skeletal_dysplasia_t2
FGF12	Epilepsy_t2
FGF14	Hereditary_Autism_t2,Ataxia_t1,Ataxia_and_Related
FGF16	Skeletal_dysplasia_t2
FGF17	Hypogonadotropic_hypogonadism_t2
FGF23	Skeletal_dysplasia_t1,Rickets_Hypoparathyroidism_panel,Sclerosing_Bone_Disease_Panel,Skeletal_Dysplasia_Panel,Skeletal_Dysplasia_and_Related
FGF3	Hearing_loss_t2,Hereditary_hearing_loss,Hearing_Loss_and_Related_t2
FGF5	Androgenetic_alopecia
FGF8	Disorders_of_sexual_development_t1,Hypogonadotropic_hypogonadism_t1,Isolated_Hypogonadotropic_Hypogonadism
FGFR1	Hearing_loss_t2,Hereditary_retinopathy_t2,Hereditary_Autism_t2,Hereditary_cancer_syndrome_Plus_t2,Skeletal_dysplasia_t1,Proportionate_short_stature_t1,Disorders_of_sexual_development_t1,Hypogonadotropic_hypogonadism_t1,Neuroendocrine_tumor_t1,Cell-free_cancer,Craniosynostosis,Hereditary_paraganglioma-pheochromocytoma_syndrome,Isolated_Hypogonadotropic_Hypogonadism,Skeletal_Dysplasia_Panel,ALL_and_others_t2,Circulating_tumor_DNA_assay_55,Lymphoma_B_cell_t1,Lymphoma_B_cell_t2,Skeletal_Dysplasia_and_Related
FGFR2	Disorders_of_sexual_development_t2,Hearing_loss_t2,Hereditary_Autism_t2,Skeletal_dysplasia_t1,Cell-free_cancer,Craniosynostosis,Skeletal_Dysplasia_Panel,Circulating_tumor_DNA_assay_55,Skeletal_Dysplasia_and_Related
FGFR3	Proportionate_short_stature_t2,Epilepsy_t2,Hearing_loss_t2,Hereditary_Autism_t2,Skeletal_dysplasia_t1,Cell-free_cancer,Craniosynostosis,Skeletal_Dysplasia_Panel,Circulating_tumor_DNA_assay_55,Lymphoma_B_cell_t2,Lymphoma_T_NK_cell_t2,Myeloma_t1,Skeletal_Dysplasia_and_Related
FGFR4	Circulating_tumor_DNA_assay_55
FGG	Coagulation_t1,Stroke,Atypical_Hemolytic_Uremic_Syndrome,Bleeding_Coagulopathy,Thrombosis
FH	Lysosomal_storage_disease_t2,Epilepsy_t2,Hereditary_cancer_syndrome_Plus_t2,Neuroendocrine_tumor_t1,Hereditary_pancancer,Hereditary_paraganglioma-pheochromocytoma_syndrome,Pheochromocytoma-Paraganglioma_Panel
FHL1	Cardiomyopathy_t2,Muscular_dystrophy_t2,Myopathy_t1,Congenital_muscular_dystrophy,Limb_girdle_muscular_dystrophy,Myofibrillar_myopathy,Myopathy_and_Related

Supplementary Table 2. List of pathogenic or likely-pathogenic germline variants

Gene	Associated diseases
FHL2	Cardiomyopathy_t2,Dilated_cardiomyopathy
FHOD3	Cardiomyopathy_t2
FIG4	Ataxia_t2,Neurodegenerative_disease_t2,Charcot-Marie-Tooth_disease_t1,Amyotrophic_lateral_sclerosis_t1,Amyotrophic_lateral_sclerosis,Charcot-Marie-Tooth,Charcot_Marie_Tooth_and_Related
FITM2	Hearing_loss_t2,Dystonia_t2
FKBP10	Skeletal_dysplasia_t2,Osteogenesis_imperfecta
FKBP14	Dermatology_t2,Connective_tissue_disorder_t2,Muscular_dystrophy_t2,Myopathy_t2,Skeletal_Dysplasia_Panel
FKRP	Cardiomyopathy_t2,Myopathy_t2,Ataxia_t2,Epilepsy_t2,Hereditary_Autism_t2,Muscular_dystrophy_t1,Congenital_muscular_dystrophy,Limb_girdle_muscular_dystrophy,Muscular_Dystrophy_and_Related
FKTN	Cardiomyopathy_t2,Myopathy_t2,Ataxia_t2,Epilepsy_t2,Hereditary_Autism_t2,Muscular_dystrophy_t1,Congenital_muscular_dystrophy,Dilated_cardiomyopathy,Limb_girdle_muscular_dystrophy,Muscular_Dystrophy_and_Related
FLAD1	Myopathy_t2
FLCN	Dermatology_t2,Hereditary_colon_cancer,Hereditary_pancancer
FLG	Dermatology_t2,corneal_keratoconus
FLI1	Coagulation_t2,Bleeding_Platelet_Disorder
FLNA	Dermatology_t2,Skeletal_dysplasia_t2,Connective_tissue_disorder_t2,Coagulation_t2,Epilepsy_t2,Hereditary_Autism_t2,Familial_thoracic_aortic_aneurysms_and_aortic_dissections,Malformation_of_cortical_development,Skeletal_Dysplasia_Panel,Bleeding_Platelet_Disorder
FLNB	Skeletal_dysplasia_t1,Skeletal_Dysplasia_Panel,Skeletal_Dysplasia_and_Related
FLNC	Arrhythmia_t2,Cardiomyopathy_t2,Muscular_dystrophy_t2,Myopathy_t1,Myofibrillar_myopathy,Arrhythmia_and_Related,Myopathy_and_Related
FLRT1	Hereditary_spastic_paraplegia_t2,Neurodegenerative_disease_t2
FLRT3	Hypogonadotropic_hypogonadism_t2
FLT1	ALL_and_others_t2
FLT3	Cell-free_cancer,Hematologic_malignancy,ALL_and_others_t1,ALL_and_others_t2,AML,Circulating_tumor_DNA_assay_55,MDS_MPN
FLT4	ALL_and_others_t2
FLVCR1	Ataxia_t2,Hereditary_retinopathy_t2,Retinitis_pigmentosa_t1,Retinitis_pigmentosa,Retinitis_Pigmentosa_and_Related,retina
FMN1	Skeletal_dysplasia_t2
FMR1	Ataxia_t2,Hereditary_Autism_t2,Ataxia,Autism_and_Related
FN1	Skeletal_dysplasia_t2,Proportionate_short_stature_t2,Alport_Syndrome_t1,Skeletal_Dysplasia_Panel,Glomerular_basement_membrane_disorders
FNDC3B	corneal_keratoconus
FOLR1	Lysosomal_storage_disease_t2,Hereditary_Autism_t2,Epilepsy_t1,Epilepsy_and_Related
FOXA2	Androgenetic_alopecia
FOXC1	Hereditary_retinopathy_t2,glaucoma
FOXD4	Cardiomyopathy_t2
FOXE1	Hypothyroidism_t1,Congenital_Hypothyroidism
FOXE3	Connective_tissue_disorder_t2,Hereditary_retinopathy_t2,Familial_thoracic_aortic_aneurysms_and_aortic_dissections,Connective_Tissue_Disorder_and_Related,Marfan_and_Related,glaucoma
FOXG1	Epilepsy_t2,Hereditary_Microcephaly_t2,Hereditary_Autism_t2,Focal_epilepsy,Autism_and_Related
FOXI1	Hearing_loss_t2,Hereditary_hearing_loss
FOXL2	Disorders_of_sexual_development_t2,Disorders_of_sex_development
FOXN1	Primary_immune_deficiency_t2,Severe_Combined_Immunodeficiency_and_CID
FOXO1	Hematologic_malignancy,Lymphoma_B_cell_t1,Lymphoma_B_cell_t2,corneal_keratoconus
FOXO3	Lymphoma_T_NK_cell_t1,Lymphoma_T_NK_cell_t2
FOXP1	Hereditary_Autism_t1,Autism_and_Related
FOXP2	Hereditary_Autism_t1

Supplementary Table 2. List of pathogenic or likely-pathogenic germline variants

Gene	Associated diseases
FOXP3	Maturity-Onset_Diabetes_of_the_Young,_MODY_t1,Inflammatory_Bowel_t1,Monogenic_diabetes_panel,Primary_immunodeficiency,Very-early-onset_inflammatory_bowel_disease,Autoimmunity_and_autoinflammatory_disorders,B_cell_and_Humoral_Immune_Deficiency,Hemophagocytic_Lymphohistiocytosis_and_Related
FOXRED1	Cardiomyopathy_t2,Epilepsy_t2
FRAS1	Androgenetic_alopecia
FREM1	Hereditary_retinopathy_t2,Craniosynostosis
FRMD7	Hereditary_retinopathy_t2,Nystagmus_and_Related,Optic_neuropathy_and_Related
FRMPD4	Hereditary_Autism_t2
FRRS1L	Epilepsy_t2
FSCN2	Hereditary_retinopathy_t2,Retinitis_pigmentosa_t1,Retinitis_pigmentosa,Retinitis_Pigmentosa_and_Related,retina
FSHB	Hypogonadotropic_hypogonadism_t1
FTH1	Anemia,Hemochromatosis
FTL	Hereditary_retinopathy_t2,Dystonia_t2,Parkinson's_disease_t2,Chorea,Dystonia,Neurodegeneration_with_brain_iron_accumulation,Anemia,Hemochromatosis
FTO	Hereditary_Autism_t2
FTSJ1	Hereditary_Autism_t2
FUCA1	Lysosomal_storage_disease_t2,Epilepsy_t2,Skeletal_Dysplasia_Panel
FUS	Ataxia_t2,Parkinson's_disease_t2,Dementia_t1,Neurodegenerative_disease_t1,Amyotrophic_lateral_sclerosis_t1,Amyotrophic_lateral_sclerosis,Dementia_and_Related
FUT8	Epilepsy_t2
FUZ	Skeletal_Dysplasia_Panel
FXN	Cardiomyopathy_t2,Charcot-Marie-Tooth_disease_t2,Ataxia_t2,Hereditary_spastic_paraplegia_t2,Neurodegenerative_disease_t2,Ataxia
FXYD2	Atypical_Hemolytic_Uremic_Syndrome_t2,Ca-Pi-Mg
FYB1	Coagulation_t2,Bleeding_Platelet_Disorder
FYCO1	Hereditary_retinopathy_t2
FYN	Lymphoma_T_NK_cell_t2
FZD2	Skeletal_Dysplasia_Panel
FZD4	Hereditary_retinopathy_t2,Pediatric_retinal_vascular_disease,Vitreoretinopathy,Vitreoretinopathy_and_Related,retina
FZD5	glaucoma
G6PC1	Lysosomal_storage_disease_t1,Glycogen_storage_disease,Lysosomal_Storage_Disease
G6PC3	Hereditary_Autism_t2,Inflammatory_Bowel_t1,Congenital_hematologic_disease,Severe_congenital_neutropenia,Very-early-onset_inflammatory_bowel_disease,Bone_Marrow_Failure_Germline_Predisposition
G6PD	Anemia_t1,Hemolytic_anemia,Primary_immunodeficiency,Anemia,Phagocyte_Defect_and_Infectious_disease
GAA	Cardiomyopathy_t2,Muscular_dystrophy_t2,Myopathy_t1,Lysosomal_storage_disease_t1,Congenital_muscular_dystrophy,Limb_girdle_muscular_dystrophy,Metabolic_myopathy,Lysosomal_Storage_Disease,Myopathy_and_Related
GAB1	Hereditary_hearing_loss
GABBR2	Epilepsy_t2
GABRA1	Epilepsy_t1,Early_onset_epilepsy,Epilepsy_and_Related
GABRA2	Epilepsy_t2
GABRB2	Epilepsy_t2
GABRB3	Epilepsy_t2,Hereditary_Autism_t1,Early_onset_epilepsy,Autism_and_Related
GABRD	Epilepsy_t2
GABRG2	Hereditary_Autism_t2,Epilepsy_t1,Early_onset_epilepsy,Epilepsy_and_Related
GAD1	Ataxia_t2,Hereditary_spastic_paraplegia_t1,Spastic_Paraplegia_and_Related
GAL	Epilepsy_t2

Supplementary Table 2. List of pathogenic or likely-pathogenic germline variants

Gene	Associated diseases
GALC	Ataxia_t2,Hereditary_spastic_paraplegia_t2,Inborn_error_of_metabolism_t2,Epilepsy_t2,Neurodegenerative_disease_t2,Charcot-Marie-Tooth_disease_t1,Lysosomal_storage_disease_t1,Charcot_Marie_Tooth_and_Related,Lysosomal_Storage_Disease
GALE	Inborn_error_of_metabolism_t2,Hereditary_Autism_t2,Lysosomal_storage_disease_t1,Lysosomal_Storage_Disease,corneal_dystrophy
GALK1	Inborn_error_of_metabolism_t2,Hereditary_retinopathy_t2,Lysosomal_storage_disease_t1,Lysosomal_Storage_Disease
GALK2	Lysosomal_storage_disease_t1
GALNS	Lysosomal_storage_disease_t1,Mucopolysaccharidosis,Skeletal_Dysplasia_Panel,Lysosomal_Storage_Disease
GALNT12	Hereditary_colon_cancer,Hereditary_pancancer
GALNT3	Skeletal_dysplasia_t2,Sclerosing_Bone_Disease_Panel
GALT	Inborn_error_of_metabolism_t2,Hereditary_retinopathy_t2,Lysosomal_storage_disease_t1,Lysosomal_Storage_Disease
GAMT	Lysosomal_storage_disease_t2,Inborn_error_of_metabolism_t2,Epilepsy_t2,Hereditary_Autism_t2,Autism_and_Related
GAN	Charcot-Marie-Tooth_disease_t2,Ataxia_t2,Hereditary_Autism_t2,Charcot_Marie_Tooth_and_Related
GANAB	Polycystic_Kidney_Disease_t1,Polycystic_kidney_disease_and_Related
GAPDH	Hemolytic_anemia
GARS1	Charcot-Marie-Tooth_disease_t1,Charcot-Marie-Tooth,Charcot_Marie_Tooth_and_Related
GAS2L2	Hereditary_Primary_Ciliary_Dyskinesia_t2
GAS8	Hereditary_Primary_Ciliary_Dyskinesia_t2
GATA1	Coagulation_t2,Anemia_t2,Congenital_hematologic_disease,Diamond-Blackfan_anemia,Hematologic_malignancy,Hemolytic_anemia,ALL_and_others_t1,ALL_and_others_t2,AML,Anemia,Bleeding_Platelet_Disorder,Bone_Marrow_Failure_Germline_Predisposition,MDS_MPN
GATA2	Coagulation_t2,Primary_immune_deficiency_t2,Congenital_hematologic_disease,Hematologic_malignancy,Hereditary_pancancer,Leukemia_predisposition,ALL_and_others_t1,ALL_and_others_t2,AML,Bleeding_Platelet_Disorder,Bone_Marrow_Failure_Germline_Predisposition,Hereditary_Myeloid_Leukemia_Panel,MDS_MPN
GATA3	Hearing_loss_t2,Cell-free_cancer,Rickets_Hypoparathyroidism_panel,ALL_and_others_t2,Lymphoma_T_NK_cell_t2
GATA4	Arrhythmia_t2,Cardiomyopathy_t2,Disorders_of_sexual_development_t2,Maturity-Onset_Diabetes_of_the_Young,_MODY_t1,Disorders_of_sex_development,Monogenic_diabetes_panel
GATA5	Arrhythmia_t2,Connective_tissue_disorder_t2
GATA6	Arrhythmia_t2,Cardiomyopathy_t2,Maturity-Onset_Diabetes_of_the_Young,_MODY_t1,Monogenic_diabetes_panel
GATAD1	Cardiomyopathy_t2,Dilated_cardiomyopathy
GATAD2B	Hereditary_Autism_t2
GATC	Cardiomyopathy_t2
GATM	Inborn_error_of_metabolism_t2,Epilepsy_t2,Autism_and_Related
GBA	Coagulation_t2,Alzheimer's_disease_t2,Dystonia_t2,Parkinson's_disease_t2,Hereditary_Autism_t2,Lysosomal_storage_disease_t1,Dementia_t1,Neurodegenerative_disease_t1,Parkinson,Bleeding_Platelet_Disorder,Lysosomal_Storage_Disease,Parkinson_Disease_and_Related
GBA2	Ataxia_t2,Hereditary_spastic_paraplegia_t2,Neurodegenerative_disease_t2
GBE1	Cardiomyopathy_t2,Muscular_dystrophy_t2,Myopathy_t2,Hereditary_spastic_paraplegia_t2,Inborn_error_of_metabolism_t2,Hereditary_Autism_t2,Neurodegenerative_disease_t2,Amyotrophic_lateral_sclerosis_t2,Lysosomal_storage_disease_t1,Glycogen_storage_disease,Metabolic_myopathy,Lysosomal_Storage_Disease
GCDH	Lysosomal_storage_disease_t2,Epilepsy_t2,Dystonia_t2,Parkinson's_disease_t2,Inborn_error_of_metabolism_t1
GCH1	Hereditary_spastic_paraplegia_t2,Inborn_error_of_metabolism_t2,Epilepsy_t2,Alzheimer's_disease_t2,Dementia_t2,Dystonia_t1,Parkinson's_disease_t1,Neurodegenerative_disease_t1,Dyskinesia-dystonia-paralysis,Dystonia,Parkinson,Paroxysmal_movement_disorder,Dystonia_and_Related,Inborn_Error_of_Metabolism,Parkinson_Disease_and_Related
GCK	Polycystic_Kidney_Disease_t2,Hereditary_Autism_t2,Maturity-Onset_Diabetes_of_the_Young,_MODY_t1,Monogenic_diabetes_panel
GCKR	Hereditary_Dyslipidemia_t2
GCLC	Anemia_t1,Hemolytic_anemia,Anemia
GCM2	Osteogenesis_imperfecta,Rickets_Hypoparathyroidism_panel,Skeletal_Dysplasia_Panel
GCNT2	Hereditary_retinopathy_t2
GCSH	Lysosomal_storage_disease_t2,Epilepsy_t2

Supplementary Table 2. List of pathogenic or likely-pathogenic germline variants

Gene	Associated diseases
GDAP1	Charcot-Marie-Tooth_disease_t1,Charcot-Marie-Tooth,Charcot_Marie_Tooth_and_Related
GDF2	Primary_Pulmonary_Hypertension_and_Related_Disordisorders
GDF3	Hereditary_retinopathy_t2
GDF5	Skeletal_dysplasia_t2,Proportionate_short_stature_t2,Skeletal_Dysplasia_Panel
GDF6	Hereditary_retinopathy_t2,retina
GDI1	Hereditary_Autism_t2
GDNF	Hirschsprung's_disease
GFAP	Ataxia_t2,Epilepsy_t2,Hereditary_Autism_t2,Ataxia
GFI1	Coagulation_t2,Congenital_hematologic_disease,Severe_congenital_neutropenia,Bone_Marrow_Failure_Germline_Predisposition
GFI1B	Coagulation_t2,Bleeding_Platelet_Disorder,Erythrocytosis
GFM1	Cardiomyopathy_t2,Epilepsy_t2,Hereditary_Microcephaly_t2,Hereditary_Autism_t2
GFM2	Epilepsy_t2
GFPT1	Congenital_myopathy
GGCX	Coagulation_t2,Atypical_Hemolytic_Uremic_Syndrome,Bleeding_Coagulopathy,Thrombosis
GH1	Skeletal_dysplasia_t2,Proportionate_short_stature_t1
GHR	Skeletal_dysplasia_t2,Hereditary_Autism_t2,Proportionate_short_stature_t1
GHRHR	Skeletal_dysplasia_t2,Proportionate_short_stature_t1
GHSR	Skeletal_dysplasia_t2,Proportionate_short_stature_t2
GIPC3	Hearing_loss_t2,Hereditary_hearing_loss,Hearing_Loss_and_Related_t2
GJA1	Dermatology_t2,Skeletal_dysplasia_t2,Hearing_loss_t2,Sclerosing_Bone_Disease_Panel,glaucoma
GJA3	Hereditary_retinopathy_t2
GJA5	Arrhythmia_t2
GJA8	Hereditary_retinopathy_t2,glaucoma
GJB1	Ataxia_t2,Charcot-Marie-Tooth_disease_t1,Charcot-Marie-Tooth,Charcot_Marie_Tooth_and_Related
GJB2	Dermatology_t2,Lysosomal_storage_disease_t2,Hearing_loss_t1,Hereditary_hearing_loss,Hearing_Loss_and_Related_t1,Hearing_Loss_and_Related_t2
GJB3	Dermatology_t2,Hearing_loss_t2,Hereditary_hearing_loss,Charcot_Marie_Tooth_and_Related
GJB4	Dermatology_t2
GJB6	Dermatology_t2,Hearing_loss_t1,Hereditary_hearing_loss,Hearing_Loss_and_Related_t1,Hearing_Loss_and_Related_t2
GJC2	Ataxia_t2,Hereditary_spastic_paraplegia_t2,Epilepsy_t2,Neurodegenerative_disease_t2
GK	Hereditary_Autism_t2
GLA	Cardiomyopathy_t2,Lysosomal_storage_disease_t1,Hereditary_Stroke_t1,Hypertrophic_cardiomyopathy,Stroke,Cardiomyopathy_and_Related,Lysosomal_Storage_Disease,Sudden_Cardiac_Arrest_and_Related,corneal_dystrophy
GLB1	Cardiomyopathy_t2,Lysosomal_storage_disease_t2,Epilepsy_t2,Mucopolysaccharidosis,Skeletal_Dysplasia_Panel,Lysosomal_Storage_Disease
GLDC	Lysosomal_storage_disease_t2,Epilepsy_t2,Inborn_Error_of_Metabolism
GLE1	Amyotrophic_lateral_sclerosis
GLI2	Skeletal_dysplasia_t2,Epilepsy_t2,Proportionate_short_stature_t1
GLI3	Skeletal_dysplasia_t2,Epilepsy_t2,Hereditary_Autism_t2,Proportionate_short_stature_t1,Craniosynostosis
GLIS2	Polycystic_Kidney_Disease_t2,Nephronophthisis_t1,Nephronophthisis_and_Related
GLIS3	Maturity-Onset_Diabetes_of_the_Young,_MODY_t1,Monogenic_diabetes_panel,Congenital_Hypothyroidism
GLRA1	Epilepsy_t2,Dystonia_t2,Parkinson's_disease_t2,Hereditary_Autism_t2,Epilepsy_and_Related
GLRB	Epilepsy_t2
GLRX5	Anemia_t1,Anemia

Supplementary Table 2. List of pathogenic or likely-pathogenic germline variants

Gene	Associated diseases
GLUD1	Epilepsy_t2,Maturity-Onset_Diabetes_of_the_Young,_MODY_t2,Urea_cycle_disorders
GLUL	Hereditary_Autism_t2,Urea_cycle_disorders
GLYCTK	Hereditary_Autism_t2
GM2A	Lysosomal_storage_disease_t2,Hereditary_Autism_t2
GMPPB	Cardiomyopathy_t2,Muscular_dystrophy_t2,Myopathy_t2,Congenital_muscular_dystrophy,Limb_girdle_muscular_dystrophy
GNA11	Skeletal_dysplasia_t2,Atypical_Hemolytic_Uremic_Syndrome_t2,Cell-free_cancer,Rickets_Hypoparathyroidism_panel,Circulating_tumor_DNA_assay_55
GNA13	Hematologic_malignancy,Lymphoma_B_cell_t1,Lymphoma_B_cell_t2
GNAI3	Nystagmus_and_Related
GNAL	Parkinson's_disease_t2,Neurodegenerative_disease_t2,Dystonia_t1,Dyskinesia-dystonia-paralysis,Dystonia,Dystonia_and_Related
GNAO1	Epilepsy_t2,Hereditary_Autism_t2,Dystonia_t1,Dystonia_and_Related,Epilepsy_and_Related
GNAQ	Cell-free_cancer,Circulating_tumor_DNA_assay_55
GNAS	Dermatology_t2,Skeletal_dysplasia_t2,Proportionate_short_stature_t2,Hereditary_Autism_t2,Hypothyroidism_t1,Cell-free_cancer,Hematologic_malignancy,Circulating_tumor_DNA_assay_55,Congenital_Hypothyroidism
GNAT1	Hereditary_retinopathy_t2,Congenital_Stationary_Night_Blindness_and_Related,Retinitis_Pigmentosa_and_Related,retina
GNAT2	Hereditary_retinopathy_t2,Achromatopsia_and_Related,Cone-Rod_Dystrophy_and_Related,Retinitis_Pigmentosa_and_Related,retina
GNB1	Epilepsy_t2
GNB3	Hereditary_retinopathy_t2,Congenital_Stationary_Night_Blindness_and_Related,retina
GNB4	Charcot-Marie-Tooth_disease_t1,Charcot-Marie-Tooth,Charcot_Marie_Tooth_and_Related
GNB5	Arrhythmia_t2
GNE	Muscular_dystrophy_t2,Myopathy_t2,Charcot-Marie-Tooth_disease_t2,Lysosomal_storage_disease_t2,Epilepsy_t2,Limb_girdle_muscular_dystrophy,Myofibrillar_myopathy
GNMT	Inborn_error_of_metabolism_t2
GNPAT	Hereditary_Autism_t2,Skeletal_dysplasia_t1,Skeletal_Dysplasia_Panel,Spondyloepiphyseal_metaphyseal_dysplasia,Skeletal_Dysplasia_and_Related
GNPTAB	Lysosomal_storage_disease_t2,Hereditary_Autism_t2,Skeletal_Dysplasia_Panel,Lysosomal_Storage_Disease
GNPTG	Lysosomal_storage_disease_t2,Retinitis_pigmentosa_t2,Hereditary_retinopathy_t2,Hereditary_Autism_t2,Skeletal_Dysplasia_Panel,Retinitis_Pigmentosa_and_Related,retina
GNRH1	Disorders_of_sexual_development_t1,Hypogonadotropic_hypogonadism_t1
GNRHR	Disorders_of_sexual_development_t1,Hypogonadotropic_hypogonadism_t1,Isolated_Hypogonadotropic_Hypogonadism
GNS	Lysosomal_storage_disease_t2,Epilepsy_t2,Mucopolysaccharidosis,Skeletal_Dysplasia_Panel,Lysosomal_Storage_Disease
GOLGA2	Muscular_dystrophy_t2,Myopathy_t2,Epilepsy_t2
GORAB	Dermatology_t2,Skeletal_dysplasia_t2,Osteogenesis_imperfecta
GOSR2	Ataxia_t2,Epilepsy_t2
GP1BA	Coagulation_t2,Bleeding_Platelet_Disorder
GP1BB	Coagulation_t2,Bleeding_Platelet_Disorder
GP6	Coagulation_t2,Bleeding_Platelet_Disorder
GP9	Coagulation_t2,Bleeding_Platelet_Disorder
GPAA1	Epilepsy_t2
GPC3	Lysosomal_storage_disease_t2,Epilepsy_t2,Hereditary_Autism_t2,Hereditary_pancancer,Overgrowth_intellectual_disability
GPC6	Skeletal_dysplasia_t2,Skeletal_Dysplasia_Panel
GPD1	Hereditary_Dyslipidemia_t1,Dyslipidemia_and_Related
GPD1L	Cardiomyopathy_t2,Arrhythmia_t1,Arrhythmia,Arrhythmia_and_Related
GPHN	Epilepsy_t2
GPI	Anemia_t1,Hemolytic_anemia,Anemia
GPIHBP1	Hereditary_Dyslipidemia_t1,Dyslipidemia_and_Related

Supplementary Table 2. List of pathogenic or likely-pathogenic germline variants

Gene	Associated diseases
GPR143	Dermatology_t2,Hereditary_retinopathy_t2,Albinism_and_Related,Optic_neuropathy_and_Related
GPR179	Hereditary_retinopathy_t2,Congenital_Stationary_Night_Blindness_and_Related,Retinitis_Pigmentosa_and_Related,retina
GPSM2	Hearing_loss_t2,Hereditary_hearing_loss,Hearing_Loss_and_Related_t2
GPT2	Hereditary_spastic_paraplegia_t2,Hereditary_Microcephaly_t2,Neurodegenerative_disease_t2
GPX1	Anemia_t1,Hemolytic_anemia,Anemia
GPX4	Skeletal_Dysplasia_Panel
GRAP	Hereditary_hearing_loss
GREM1	Skeletal_dysplasia_t2,Hereditary_colon_cancer,Hereditary_pancancer,Colorectal_Cancer_Polyposis_Panel
GRHL2	Hearing_loss_t2,Hereditary_hearing_loss,corneal_dystrophy
GRHPR	Ca-Pi-Mg
GRIA3	Epilepsy_t2,Hereditary_Autism_t2
GRIA4	Epilepsy_t2
GRID2	Ataxia_t2
GRIK2	Epilepsy_t2,Hereditary_Autism_t2
GRIN1	Epilepsy_t2,Hereditary_Autism_t2
GRIN2A	Hereditary_Autism_t2,Epilepsy_t1,Epilepsy_and_Related
GRIN2B	Epilepsy_t2,Hereditary_Autism_t1,Autism_and_Related,Epilepsy_and_Related
GRIP1	Dermatology_t2,Hereditary_Autism_t2
GRK1	Hereditary_retinopathy_t2,Congenital_Stationary_Night_Blindness_and_Related,Retinitis_Pigmentosa_and_Related,retina
GRM1	Ataxia_t2,Hereditary_Autism_t2
GRM6	Hereditary_retinopathy_t2,Congenital_Stationary_Night_Blindness_and_Related,Retinitis_Pigmentosa_and_Related,retina
GRN	Lysosomal_storage_disease_t2,Epilepsy_t2,Hereditary_retinopathy_t2,Alzheimer's_disease_t2,Parkinson's_disease_t2,Hereditary_Autism_t2,Dementia_t1,Neurodegenerative_disease_t1,Amyotrophic_lateral_sclerosis_t1,Parkinson,Dementia_and_Related,Parkinson_Disease_and_Related
GRPR	Hereditary_Autism_t2
GRXCR1	Hearing_loss_t2,Hereditary_hearing_loss
GRXCR2	Hearing_loss_t2,Hereditary_hearing_loss
GSDME	Hearing_loss_t2,Hereditary_hearing_loss,Hearing_Loss_and_Related_t2
GSK3B	Cardiomyopathy_t2
GSN	Dermatology_t2,Hereditary_retinopathy_t2,Corneal_Dystrophy_and_Related,corneal_dystrophy
GSPT2	Hereditary_Autism_t2
GSR	Anemia_t1,Hemolytic_anemia,Anemia
GSS	Ataxia_t2,Hereditary_Autism_t2,Anemia_t1,Hemolytic_anemia,Anemia
GSTP1	Hereditary_pancancer
GTF2H5	Dermatology_t2
GTPBP3	Cardiomyopathy_t2,Epilepsy_t2
GUCA1A	Hereditary_retinopathy_t2,Macular_dystrophy,Cone-Rod_Dystrophy_and_Related,Retinitis_Pigmentosa_and_Related,retina
GUCA1B	Hereditary_retinopathy_t2,Retinitis_pigmentosa_t1,Retinitis_pigmentosa,Retinitis_Pigmentosa_and_Related,retina
GUCY1A1	Hereditary_Stroke_t2,Moyamoya_disease,Stroke
GUCY2C	Inflammatory_Bowel_t2
GUCY2D	Retinitis_pigmentosa_t2,Hereditary_retinopathy_t1,Leber_congenital_amaurosis,Macular_dystrophy,Cone-Rod_Dystrophy_and_Related,Leber's_Congenital_Amaurosis_and_Related,Retinitis_Pigmentosa_and_Related,retina
GUSB	Cardiomyopathy_t2,Lysosomal_storage_disease_t2,Hereditary_Autism_t2,Mucopolysaccharidosis,Skeletal_Dysplasia_Panel,Lysosomal_Storage_Disease

Supplementary Table 2. List of pathogenic or likely-pathogenic germline variants

Gene	Associated diseases
GYG1	Arrhythmia_t2,Myopathy_t2,Metabolic_myopathy
GYPA	Hemolytic_anemia
GYPB	Hemolytic_anemia
GYPC	Anemia_t2,Hemolytic_anemia,Anemia
GYS1	Muscular_dystrophy_t2,Myopathy_t2,Lysosomal_storage_disease_t2,Metabolic_myopathy
GYS2	Lysosomal_storage_disease_t2,Glycogen_storage_disease
GZF1	Skeletal_Dysplasia_Panel
H1-4	Lymphoma_t2,Overgrowth_intellectual_disability
H3-3A	Hereditary_cancer_syndrome_Plus_t2,Neuroendocrine_tumor_t1,Hereditary_paraganglioma-pheochromocytoma_syndrome
HACE1	Hereditary_spastic_paraplegia_t2,Epilepsy_t2,Neurodegenerative_disease_t2,Lymphoma_T_NK_cell_t1,Lymphoma_T_NK_cell_t2
HADH	Inborn_error_of_metabolism_t2,Maturity-Onset_Diabetes_of_the_Young,_MODY_t2
HADHA	Arrhythmia_t2,Cardiomyopathy_t2,Myopathy_t2,Inborn_error_of_metabolism_t1,Metabolic_myopathy,Charcot_Marie_Tooth_and_Related,Inborn_Error_of_Metabolism
HADHB	Myopathy_t2,Charcot-Marie-Tooth_disease_t2,Inborn_error_of_metabolism_t1,Metabolic_myopathy,Charcot_Marie_Tooth_and_Related
HAMP	Anemia,Hemochromatosis
HAND1	Cardiomyopathy_t2
HARS1	Charcot-Marie-Tooth_disease_t2,Hearing_loss_t2,Hereditary_retinopathy_t2,Charcot-Marie-Tooth,Hereditary_hearing_loss,Charcot_Marie_Tooth_and_Related,Retinitis_Pigmentosa_and_Related,retina
HARS2	Ataxia_t2,Hearing_loss_t2,Hereditary_hearing_loss
HAX1	Coagulation_t2,Hereditary_Autism_t2,Congenital_hematologic_disease,Severe_congenital_neutropenia,ALL_and_others_t2,Bone_Marrow_Failure_Germline_Predisposition,MDS_MPN
HBA1	Anemia_t2,Hemolytic_anemia,Anemia,Erythrocytosis
HBA2	Anemia_t2,Hemolytic_anemia,Anemia,Erythrocytosis
HBB	Anemia_t2,Hereditary_Stroke_t1,Hemolytic_anemia,Stroke,Anemia,Erythrocytosis
HBD	Anemia_t2,Anemia
HBG1	Anemia
HBG2	Anemia
HCCS	Hereditary_retinopathy_t2,Hereditary_Autism_t2,glaucoma
HCFC1	Hereditary_Autism_t2
HCN1	Epilepsy_t2,Hereditary_retinopathy_t2,Early_onset_epilepsy,Epilepsy_and_Related
HCN2	Epilepsy_t2
HCN4	Cardiomyopathy_t2,Connective_tissue_disorder_t2,Epilepsy_t2,Arrhythmia_t1,Arrhythmia,Arrhythmia_and_Related
HDAC4	Skeletal_dysplasia_t2,Epilepsy_t2,Hereditary_Autism_t2,Androgenetic_alopecia
HDAC8	Skeletal_dysplasia_t2,Proportionate_short_stature_t2,Hypogonadotropic_hypogonadism_t2,Hereditary_Autism_t2
HDAC9	Androgenetic_alopecia
HECW2	Epilepsy_t2,Hereditary_Autism_t2
HEPACAM	Ataxia_t2,Epilepsy_t2,Hereditary_Autism_t2
HEPH	Anemia
HES7	Skeletal_dysplasia_t2
HESX1	Skeletal_dysplasia_t2,Disorders_of_sexual_development_t2,Hereditary_retinopathy_t2,Proportionate_short_stature_t1,Hypothyroidism_t1,Hypogonadotropic_hypogonadism_t1,Congenital_Hypothyroidism,Nystagmus_and_Related,Optic_neuropathy_and_Related
HEXA	Hereditary_spastic_paraplegia_t2,Lysosomal_storage_disease_t2,Epilepsy_t2,Dystonia_t2,Neurodegenerative_disease_t2,Amyotrophic_lateral_sclerosis_t2
HEXB	Lysosomal_storage_disease_t2,Epilepsy_t2,Hereditary_Autism_t2
HFE	Cardiomyopathy_t2,Dermatology_t2,Disorders_of_sexual_development_t2,Hypogonadotropic_hypogonadism_t2,Anemia,Hemochromatosis
HGF	Hearing_loss_t2,Hereditary_hearing_loss,corneal_keratoconus

Supplementary Table 2. List of pathogenic or likely-pathogenic germline variants

Gene	Associated diseases
HGSNAT	Lysosomal_storage_disease_t2,Epilepsy_t2,Hereditary_retinopathy_t2,Retinitis_pigmentosa_t1,Mucopolysaccharidosis,Retinitis_pigmentosa,Skeletal_Dysplasia_Panel,Lysosomal_Storage_Disease,Retinitis_Pigmentosa_and_Related,retina
HIBCH	Ataxia_t2,Epilepsy_t2
HIF1A	Erythrocytosis
HIF3A	Erythrocytosis
HINT1	Charcot-Marie-Tooth_disease_t2,Charcot-Marie-Tooth,Charcot_Marie_Tooth_and_Related
HJV	Anemia,Hemochromatosis
HK1	Charcot-Marie-Tooth_disease_t2,Hereditary_retinopathy_t2,Retinitis_pigmentosa_t1,Anemia_t1,Hemolytic_anemia,Retinitis_pigmentosa,Anemia,Charcot_Marie_Tooth_and_Related,Retinitis_Pigmentosa_and_Related,retina
HLCS	Inborn_error_of_metabolism_t1
HMBS	Dermatology_t2
HMCN1	Hereditary_retinopathy_t2,retina
HMGB3	Hereditary_Microcephaly_t2
HMGCL	Inborn_error_of_metabolism_t1
HMOX1	Anemia_t2
HMX1	Hereditary_retinopathy_t2,retina
HNF1A	Maturity-Onset_Diabetes_of_the_Young,_MODY_t1,Monogenic_diabetes_panel
HNF1B	Atypical_Hemolytic_Uremic_Syndrome_t2,Maturity-Onset_Diabetes_of_the_Young,_MODY_t1,Polycystic_Kidney_Disease_t1,Ca-Pi-Mg,Monogenic_diabetes_panel,Polycystic_kidney_disease_and_Related
HNF4A	Skeletal_dysplasia_t2,Atypical_Hemolytic_Uremic_Syndrome_t2,Polycystic_Kidney_Disease_t2,Maturity-Onset_Diabetes_of_the_Young,_MODY_t1,Monogenic_diabetes_panel
HNRNPA1	Neurodegenerative_disease_t2,Amyotrophic_lateral_sclerosis_t1,Amyotrophic_lateral_sclerosis
HNRNPA2B1	Muscular_dystrophy_t2,Myopathy_t2,Dementia_t2,Neurodegenerative_disease_t2,Amyotrophic_lateral_sclerosis_t1
HNRNPDL	Muscular_dystrophy_t2,Myopathy_t2,Limb_girdle_muscular_dystrophy
HNRNPU	Epilepsy_t2,Epilepsy_and_Related
HOGA1	Ca-Pi-Mg
HOMER2	Hearing_loss_t2,Hereditary_hearing_loss
HOXA1	Hereditary_Autism_t1
HOXA11	Coagulation_t2,Bleeding_Platelet_Disorder,Skeletal_Dysplasia_and_Related
HOXA13	Skeletal_dysplasia_t2
HOXB1	Hearing_loss_t2
HOXB13	Hereditary_pancancer
HOXC13	Dermatology_t2
HOXD10	Charcot_Marie_Tooth_and_Related
HOXD13	Skeletal_dysplasia_t2
HPCA	Dystonia_t1,Dyskinesia-dystonia-paralysis,Dystonia,Dystonia_and_Related
HPD	Lysosomal_storage_disease_t2,Inborn_error_of_metabolism_t2,Epilepsy_t2,Hereditary_Autism_t2
HPGD	Skeletal_dysplasia_t2,Sclerosing_Bone_Disease_Panel
HPRT1	Hereditary_Autism_t2,Lysosomal_storage_disease_t1,Dystonia,Lysosomal_Storage_Disease
HPS1	Dermatology_t2,Coagulation_t2,Hereditary_retinopathy_t2,Inflammatory_Bowel_t2,Very-early-onset_inflammatory_bowel_disease,Albinism_and_Related,Bleeding_Platelet_Disorder
HPS3	Dermatology_t2,Coagulation_t2,Hereditary_retinopathy_t2,Albinism_and_Related,Bleeding_Platelet_Disorder
HPS4	Dermatology_t2,Coagulation_t2,Hereditary_retinopathy_t2,Inflammatory_Bowel_t2,Very-early-onset_inflammatory_bowel_disease,Albinism_and_Related,Bleeding_Platelet_Disorder

Supplementary Table 2. List of pathogenic or likely-pathogenic germline variants

Gene	Associated diseases
HPS5	Dermatology_t2,Coagulation_t2,Hereditary_retinopathy_t2,Albinism_and_Related,Bleeding_Platelet_Disorder,Optic_neuropathy_and_Related
HPS6	Dermatology_t2,Coagulation_t2,Hereditary_retinopathy_t2,Inflammatory_Bowel_t2,Very-early-onset_inflammatory_bowel_disease,Albinism_and_Related,Bleeding_Platelet_Disorder
HR	Dermatology_t2
HRAS	Cardiomyopathy_t2,Skeletal_dysplasia_t2,Proportionate_short_stature_t2,Lysosomal_storage_disease_t2,Epilepsy_t2,Hereditary_Autism_t2,Hereditary_cancer_syndrome_Plus_t2,Rasopathies_t1,Neuroendocrine_tumor_t1,Cell-free_cancer,Hematologic_malignancy,Hereditary_pancancer,Hereditary_paraganglioma-pheochromocytoma_syndrome,Noonan_syndrome,Skeletal_Dysplasia_Panel,ALL_and_others_t1,ALL_and_others_t2,Circulating_tumor_DNA_assay_55,Germline_Cancer,Lymphoma_T_NK_cell_t2,MDS_MPN,Noonan_Syndrome_and_Related
HRG	Coagulation_t2,Atypical_Hemolytic_Uremic_Syndrome,Bleeding_Coagulopathy,Thrombosis
HS3ST3B1	Charcot_Marie_Tooth_and_Related
HS6ST1	Hypogonadotropic_hypogonadism_t1
HSD11B2	Atypical_Hemolytic_Uremic_Syndrome_t2
HSD17B10	Inborn_error_of_metabolism_t2,Epilepsy_t2,Hereditary_Autism_t2
HSD17B3	Disorders_of_sexual_development_t1,Disorders_of_sex_development
HSD17B4	Hearing_loss_t2,Hereditary_hearing_loss
HSD3B2	Disorders_of_sexual_development_t1,Disorders_of_sex_development
HSD3B7	Cholestasis_t1,Neonatal_cholestasis
HSF4	Hereditary_retinopathy_t2
HSPA9	Skeletal_Dysplasia_Panel,Anemia
HSPB1	Charcot-Marie-Tooth_disease_t1,Charcot-Marie-Tooth,Charcot_Marie_Tooth_and_Related
HSPB3	Charcot-Marie-Tooth,Charcot_Marie_Tooth_and_Related
HSPB8	Charcot-Marie-Tooth_disease_t1,Charcot-Marie-Tooth,Charcot_Marie_Tooth_and_Related
HSPD1	Ataxia_t2,Hereditary_spastic_paraplegia_t2,Epilepsy_t2,Hereditary_Autism_t2,Neurodegenerative_disease_t2,Amyotrophic_lateral_sclerosis_t2,Hereditary_spastic_paraplegia_panel
HSPG2	Skeletal_dysplasia_t2,Ataxia_t2,Skeletal_Dysplasia_Panel
HTRA1	Epilepsy_t2,Hereditary_retinopathy_t2,Hereditary_Stroke_t1,Stroke,Dementia_and_Related
HTRA2	Alzheimer's_disease_t2,Dementia_t2,Parkinson's_disease_t2,Neurodegenerative_disease_t2
HTT	Epilepsy_t2
HUWE1	Hereditary_Autism_t2
HYAL1	Lysosomal_storage_disease_t2,Mucopolysaccharidosis,Lysosomal_Storage_Disease
HYDIN	Hereditary_Primary_Ciliary_Dyskinesia_t1
IARS2	Skeletal_Dysplasia_Panel
IBA57	Hereditary_spastic_paraplegia_t2,Epilepsy_t2,Neurodegenerative_disease_t2
ICOS	Primary_immune_deficiency_t1,Inflammatory_Bowel_t1,Primary_immunodeficiency,Very-early-onset_inflammatory_bowel_disease,Autoimmunity_and_autoinflammatory_disorders,B_cell_and_Humoral_Immune_Deficiency,Severe_Combined_Immunodeficiency_and_CID
ID3	Lymphoma_t2,Lymphoma_t1,Lymphoma_B_cell_t1,Lymphoma_B_cell_t2
ID4	Skeletal_Dysplasia_Panel
IDH1	Skeletal_dysplasia_t2,Cell-free_cancer,Hematologic_malignancy,ALL_and_others_t1,ALL_and_others_t2,AML,Circulating_tumor_DNA_assay_55,MDS_MPN,Myeloma_t1
IDH2	Skeletal_dysplasia_t2,Hereditary_cancer_syndrome_Plus_t2,Neuroendocrine_tumor_t1,Lymphoma_t1,Cell-free_cancer,Hematologic_malignancy,Hereditary_paraganglioma-pheochromocytoma_syndrome,ALL_and_others_t1,ALL_and_others_t2,AML,Circulating_tumor_DNA_assay_55,Lymphoma_T_NK_cell_t1,Lymphoma_T_NK_cell_t2,MDS_MPN,Myeloma_t1
IDH3A	Retinitis_pigmentosa_t2,Hereditary_retinopathy_t2,Retinitis_Pigmentosa_and_Related
IDH3B	Hereditary_retinopathy_t2,Retinitis_pigmentosa_t1,Retinitis_pigmentosa,Retinitis_Pigmentosa_and_Related,retina
IDS	Skeletal_dysplasia_t2,Epilepsy_t2,Hereditary_Autism_t2,Lysosomal_storage_disease_t1,Mucopolysaccharidosis,Skeletal_Dysplasia_Panel,Lysosomal_Storage_Disease
IDUA	Cardiomyopathy_t2,Skeletal_dysplasia_t2,Proportionate_short_stature_t2,Lysosomal_storage_disease_t1,Mucopolysaccharidosis,Skeletal_Dysplasia_Panel,Lysosomal_Storage_Disease

Supplementary Table 2. List of pathogenic or likely-pathogenic germline variants

Gene	Associated diseases
IER3IP1	Epilepsy_t2,Maturity-Onset_Diabetes_of_the_Young,_MODY_t1,Hereditary_Microcephaly_t1,Monogenic_diabetes_panel
IFIH1	Hereditary_retinopathy_t2,Osteogenesis_imperfecta,Autoimmunity_and_autoinflammatory_disorders,B_cell_and_Humoral_Immune_Deficiency,Phagocyte_Defect_and_Infectious_disease,Severe_Combined_Immunodeficiency_and_CID
IFITM5	Skeletal_dysplasia_t2,Osteogenesis_imperfecta
IFNAR2	Phagocyte_Defect_and_Infectious_disease
IFNG	Coagulation_t2
IFNGR1	Primary_immune_deficiency_t2,Primary_immunodeficiency,Phagocyte_Defect_and_Infectious_disease
IFNGR2	Primary_immune_deficiency_t2,Primary_immunodeficiency,Phagocyte_Defect_and_Infectious_disease
IFNLR1	Hereditary_hearing_loss
IFRD1	Charcot_Marie_Tooth_and_Related
IFT122	Dermatology_t2,Skeletal_dysplasia_t2,Proportionate_short_stature_t2,Craniosynostosis,Skeletal_Dysplasia_Panel
IFT140	Skeletal_dysplasia_t2,Proportionate_short_stature_t2,Hereditary_retinopathy_t2,Retinitis_pigmentosa_t1,Retinitis_pigmentosa,Skeletal_Dysplasia_Panel,Spondyloepiphyseal_metaphyseal_dysplasia,Retinitis_Pigmentosa_and_Related,retina
IFT172	Skeletal_dysplasia_t2,Hereditary_retinopathy_t2,Polycystic_Kidney_Disease_t2,Retinitis_pigmentosa_t1,Nephronophthisis_t1,Retinitis_pigmentosa,Skeletal_Dysplasia_Panel,Spondyloepiphyseal_metaphyseal_dysplasia,Nephronophthisis_and_Related,Retinitis_Pigmentosa_and_Related,retina
IFT27	Hereditary_retinopathy_t2,retina
IFT43	Skeletal_dysplasia_t2,Craniosynostosis,Skeletal_Dysplasia_Panel
IFT52	Skeletal_dysplasia_t2,Skeletal_Dysplasia_Panel
IFT80	Skeletal_dysplasia_t2,Skeletal_Dysplasia_Panel
IFT81	Hereditary_retinopathy_t2,Skeletal_Dysplasia_Panel,retina
IGBP1	Hereditary_Autism_t2
IGF1	Skeletal_dysplasia_t2,Hereditary_Autism_t2,Proportionate_short_stature_t1
IGF1R	Skeletal_dysplasia_t2,Hereditary_Autism_t2,Proportionate_short_stature_t1,Cell-free_cancer
IGFALS	Skeletal_dysplasia_t2,Proportionate_short_stature_t2
IGHMBP2	Muscular_dystrophy_t2,Myopathy_t2,Charcot-Marie-Tooth_disease_t1,Charcot-Marie-Tooth,Congenital_myopathy,Charcot_Marie_Tooth_and_Related
IGLL1	Primary_immune_deficiency_t2
IGSF1	Hypothyroidism_t2,Congenital_Hypothyroidism
IHH	Skeletal_dysplasia_t2,Proportionate_short_stature_t2,Skeletal_Dysplasia_Panel
IKBKB	Primary_immune_deficiency_t2,Lymphoma_t1,Autoimmunity_and_autoinflammatory_disorders,B_cell_and_Humoral_Immune_Deficiency,Lymphoma_T_NK_cell_t2,Phagocyte_Defect_and_Infectious_disease,Severe_Combined_Immunodeficiency_and_CID
IKBKG	Inflammatory_Bowel_t2,Primary_immunodeficiency,Sclerosing_Bone_Disease_Panel,Very-early-onset_inflammatory_bowel_disease
IKZF1	Primary_immune_deficiency_t1,Lymphoma_t1,Hematologic_malignancy,B_cell_and_Humoral_Immune_Deficiency,ALL_and_others_t1,ALL_and_others_t2,AML,Hereditary_Myeloid_Leukemia_Panel,Myeloma_t1
IL10	Inflammatory_Bowel_t2,Very-early-onset_inflammatory_bowel_disease,Autoimmunity_and_autoinflammatory_disorders,B_cell_and_Humoral_Immune_Deficiency,Phagocyte_Defect_and_Infectious_disease
IL10RA	Primary_immune_deficiency_t1,Inflammatory_Bowel_t1,Very-early-onset_inflammatory_bowel_disease,Autoimmunity_and_autoinflammatory_disorders,B_cell_and_Humoral_Immune_Deficiency,Phagocyte_Defect_and_Infectious_disease
IL10RB	Primary_immune_deficiency_t1,Inflammatory_Bowel_t1,Very-early-onset_inflammatory_bowel_disease,Autoimmunity_and_autoinflammatory_disorders,B_cell_and_Humoral_Immune_Deficiency,Phagocyte_Defect_and_Infectious_disease
IL11RA	Craniosynostosis
IL12B	Primary_immune_deficiency_t2,Primary_immunodeficiency,Phagocyte_Defect_and_Infectious_disease
IL12RB1	Primary_immune_deficiency_t2,Primary_immunodeficiency,Phagocyte_Defect_and_Infectious_disease

Supplementary Table 2. List of pathogenic or likely-pathogenic germline variants

Gene	Associated diseases
IL17F	Primary_immune_deficiency_t2,Phagocyte_Defect_and_Infectious_disease
IL17RA	Primary_immune_deficiency_t2,Phagocyte_Defect_and_Infectious_disease
IL17RC	Primary_immune_deficiency_t2,Phagocyte_Defect_and_Infectious_disease
IL17RD	Hypogonadotropic_hypogonadism_t1
IL1A	corneal_keratoconus
IL1B	corneal_keratoconus
IL1RAPL1	Hereditary_Autism_t2,Autism_and_Related
IL1RN	Autoinflammatory_disorders,Skeletal_Dysplasia_Panel,corneal_keratoconus
IL21	Primary_immune_deficiency_t2,Inflammatory_Bowel_t2,Very-early-onset_inflammatory_bowel_disease
IL21R	Primary_immune_deficiency_t2
IL2RA	Primary_immune_deficiency_t2,Inflammatory_Bowel_t1,Familial_hemophagocytic_lymphohistiocytosis,Primary_immunodeficiency,Very-early-onset_inflammatory_bowel_disease,B_cell_and_Humoral_Immune_Deficiency,Autoimmunity_and_autoinflammatory_disorders,Hemophagocytic_Lymphohistiocytosis_and_Related
IL2RG	Primary_immune_deficiency_t1,Inflammatory_Bowel_t1,Familial_hemophagocytic_lymphohistiocytosis,Primary_immunodeficiency,Very-early-onset_inflammatory_bowel_disease,Hemophagocytic_Lymphohistiocytosis_and_Related,Severe_Combined_Immunodeficiency_and_CID
IL36RN	Dermatology_t2,Autoinflammatory_disorders
IL7R	Primary_immune_deficiency_t1,Primary_immunodeficiency,ALL_and_others_t2,Lymphoma_T_NK_cell_t2,Severe_Combined_Immunodeficiency_and_CID
ILDR1	Hearing_loss_t2,Hereditary_hearing_loss,Hearing_Loss_and_Related_t2
ILK	Cardiomyopathy_t2,Dilated_cardiomyopathy
IMMP2L	Hereditary_Autism_t2,corneal_keratoconus
IMPA1	Hereditary_Autism_t2
IMPDH1	Hereditary_retinopathy_t2,Retinitis_pigmentosa_t1,Leber_congenital_amaurosis,Retinitis_pigmentosa,Leber's_Congenital_Amaurosis_and_Related,Retinitis_Pigmentosa_and_Related,retina
IMPG1	Hereditary_retinopathy_t2,Macular_dystrophy,Retinitis_pigmentosa,Macular_dystrophy_and_Related,Retinitis_Pigmentosa_and_Related,retina
IMPG2	Hereditary_retinopathy_t2,Retinitis_pigmentosa_t1,Macular_dystrophy,Retinitis_pigmentosa,Macular_dystrophy_and_Related,Retinitis_Pigmentosa_and_Related,retina
INF2	Charcot-Marie-Tooth_disease_t2,Atypical_Hemolytic_Uremic_Syndrome_t2,Charcot-Marie-Tooth,Glomerulopathy,Atypical_Hemolytic_Uremic_Syndrome,Bleeding_Coagulopathy,Charcot_Marie_Tooth_and_Related,Thrombosis
INO80	B_cell_and_Humoral_Immune_Deficiency
INPP5E	Ataxia_t2,Retinitis_pigmentosa_t2,Hereditary_retinopathy_t1,Retinitis_Pigmentosa_and_Related,retina
INPP5K	Muscular_dystrophy_t2,Myopathy_t2
INPPL1	Skeletal_dysplasia_t2,Proportionate_short_stature_t2,Skeletal_Dysplasia_Panel,Spondyloepiphyseal_metaphyseal_dysplasia
INS	Polycystic_Kidney_Disease_t2,Maturity-Onset_Diabetes_of_the_Young,_MODY_t1,Monogenic_diabetes_panel
INSL3	Disorders_of_sexual_development_t2
INSR	Skeletal_dysplasia_t2,Proportionate_short_stature_t2,Hereditary_Autism_t2,Maturity-Onset_Diabetes_of_the_Young,_MODY_t1,Monogenic_diabetes_panel
INTU	Skeletal_Dysplasia_Panel
INVS	Retinitis_pigmentosa_t2,Hereditary_retinopathy_t2,Hereditary_Primary_Ciliary_Dyskinesia_t2,Nephronophthisis_t1,Polycystic_Kidney_Disease_t1,Nephronophthisis_and_Related,Polycystic_kidney_disease_and_Related,Retinitis_Pigmentosa_and_Related,retina
IPO5	Androgenetic_alopecia,corneal_keratoconus
IQCB1	Retinitis_pigmentosa_t2,Polycystic_Kidney_Disease_t2,Hereditary_retinopathy_t1,Nephronophthisis_t1,Leber_congenital_amaurosis,Leber's_Congenital_Amaurosis_and_Related,Nephronophthisis_and_Related,Retinitis_Pigmentosa_and_Related,retina
IQSEC2	Epilepsy_t2,Hereditary_Autism_t2,Early_onset_epilepsy,Epilepsy_and_Related
IRAK1	Lymphoma_B_cell_t2,Phagocyte_Defect_and_Infectious_disease
IRAK4	Lymphoma_B_cell_t2,Phagocyte_Defect_and_Infectious_disease
IRF2BP2	Autoimmunity_and_autoinflammatory_disorders,B_cell_and_Humoral_Immune_Deficiency

Supplementary Table 2. List of pathogenic or likely-pathogenic germline variants

Gene	Associated diseases
IRF2BPL	Ataxia_t2,Hereditary_spastic_paraplegia_t2,Epilepsy_t2,Hypogonadotropic_hypogonadism_t2,Neurodegenerative_disease_t2
IRF3	Phagocyte_Defect_and_Infectious_disease
IRF4	Androgenetic_alopecia,Lymphoma_T_NK_cell_t2,Myeloma_t1
IRF7	Primary_immunodeficiency,Phagocyte_Defect_and_Infectious_disease
IRF8	Primary_immune_deficiency_t2,Phagocyte_Defect_and_Infectious_disease
IRS1	Skeletal_dysplasia_t2,Proportionate_short_stature_t2
IRX1	Hereditary_retinopathy_t2
IRX5	Hereditary_Autism_t2
ISCU	Myopathy_t2,Metabolic_myopathy
ISG15	Primary_immune_deficiency_t2,Phagocyte_Defect_and_Infectious_disease
ITCH	Hemophagocytic_Lymphohistiocytosis_and_Related
ITGA2	Coagulation_t2,Bleeding_Platelet_Disorder
ITGA2B	Coagulation_t2,Bleeding_Platelet_Disorder
ITGA3	Dermatology_t1,Bleeding_Platelet_Disorder
ITGA6	Dermatology_t1
ITGA7	Muscular_dystrophy_t2,Myopathy_t2,Hereditary_Autism_t2,Congenital_muscular_dystrophy,Limb_girdle_muscular_dystrophy
ITGB2	Primary_immune_deficiency_t2,Inflammatory_Bowel_t1,Primary_immunodeficiency,Very-early-onset_inflammatory_bowel_disease,Phagocyte_Defect_and_Infectious_disease
ITGB3	Coagulation_t2,Bleeding_Platelet_Disorder
ITGB4	Dermatology_t1
ITK	Primary_immune_deficiency_t1,Familial_hemophagocytic_lymphohistiocytosis,B_cell_and_Humoral_Immune_Deficiency,Hemophagocytic_Lymphohistiocytosis_and_Related,Hereditary_Myeloid_Leukemia_Panel,Severe_Combined_Immunodeficiency_and_CID
ITM2B	Ataxia_t2,Hereditary_retinopathy_t2,Hereditary_Stroke_t1,Stroke,retina
ITPA	Epilepsy_t2
ITPR1	Hereditary_Autism_t2,Ataxia_t1,Ataxia_and_Related
ITPR2	Androgenetic_alopecia
IVD	Inborn_error_of_metabolism_t1,Inborn_Error_of_Metabolism
IYD	Hypothyroidism_t1,Congenital_Hypothyroidism
JAG1	Hereditary_retinopathy_t2,Polycystic_Kidney_Disease_t2,Cholestasis_t1,Hereditary_hearing_loss,Neonatal_cholestasis,Hearing_Loss_and_Related_t2,retina,glaucoma
JAGN1	Congenital_hematologic_disease,Severe_congenital_neutropenia
JAK1	ALL_and_others_t2,Autoimmunity_and_autoinflammatory_disorders,B_cell_and_Humoral_Immune_Deficiency,Lymphoma_T_NK_cell_t1,Lymphoma_T_NK_cell_t2,Phagocyte_Defect_and_Infectious_disease
JAK2	Cell-free_cancer,Hematologic_malignancy,ALL_and_others_t1,ALL_and_others_t2,AML,Bone_Marrow_Failure_Germline_Predisposition,Erythrocytosis,Lymphoma_B_cell_t1,Lymphoma_B_cell_t2,Lymphoma_T_NK_cell_t1,Lymphoma_T_NK_cell_t2,MDS_MPN,Myeloma_t1,Thrombosis
JAK3	Primary_immune_deficiency_t2,Lymphoma_t1,Cell-free_cancer,Hematologic_malignancy,Primary_immunodeficiency,ALL_and_others_t2,Bone_Marrow_Failure_Germline_Predisposition,Lymphoma_T_NK_cell_t1,Lymphoma_T_NK_cell_t2,Severe_Combined_Immunodeficiency_and_CID
JMJD1C	Epilepsy_t2
JPH2	Cardiomyopathy_t2,Hypertrophic_cardiomyopathy
JUP	Arrhythmia_t2,Cardiomyopathy_t2,Dermatology_t1,Arrhythmia,Dilated_cardiomyopathy,Arrhythmia_and_Related
KANK4	corneal_dystrophy
KANSL1	Epilepsy_t2,Hereditary_Microcephaly_t2,Bone_Marrow_Failure_Germline_Predisposition

Supplementary Table 2. List of pathogenic or likely-pathogenic germline variants

Gene	Associated diseases
KARS1	Charcot-Marie-Tooth_disease_t2,Hearing_loss_t2,Hereditary_hearing_loss,Charcot_Marie_Tooth_and_Related
KAT6A	Hereditary_Microcephaly_t2,Hereditary_Autism_t2
KAT6B	Skeletal_dysplasia_t2,Rasopathies_t2,Hereditary_Autism_t2,Noonan_syndrome
KATNAL2	Hereditary_Autism_t2
KATNB1	Hereditary_Microcephaly_t2
KATNIP	Hereditary_retinopathy_t2
KBTBD13	Muscular_dystrophy_t2,Myopathy_t1,Congenital_myopathy,Myofibrillar_myopathy,Myopathy_and_Related
KCNA1	Arrhythmia_t2,Epilepsy_t2,Atypical_Hemolytic_Uremic_Syndrome_t2,Ataxia_t1,Ataxia,Ca-Pi-Mg,Metabolic_myopathy,Paroxysmal_movement_disorder,Ataxia_and_Related
KCNA2	Epilepsy_t2,Early_onset_epilepsy,Focal_epilepsy
KCNA5	Arrhythmia_t2,Primary_Pulmonary_Hypertension_and_Related_Disordisorders
KCNAB2	Epilepsy_t2
KCNB1	Epilepsy_t2,Hereditary_Autism_t2,Epilepsy_and_Related
KCNC1	Epilepsy_t2
KCNC3	Ataxia_t1,Ataxia_and_Related
KCND2	Epilepsy_t2
KCND3	Arrhythmia_t1,Ataxia_t1,Arrhythmia_and_Related,Ataxia_and_Related,corneal_keratoconus
KCNE1	Cardiomyopathy_t2,Arrhythmia_t1,Hearing_loss_t1,Arrhythmia,Hereditary_hearing_loss,Arrhythmia_and_Related
KCNE2	Cardiomyopathy_t2,Arrhythmia_t1,Arrhythmia,Arrhythmia_and_Related
KCNE3	Cardiomyopathy_t2,Arrhythmia_t1,Arrhythmia,Metabolic_myopathy,Arrhythmia_and_Related
KCNE5	Arrhythmia_t2
KCNH1	Epilepsy_t2
KCNH2	Cardiomyopathy_t2,Epilepsy_t2,Arrhythmia_t1,Arrhythmia,Arrhythmia_and_Related,Sudden_Cardiac_Arrest_and_Related
KCNH5	Epilepsy_t2
KCNJ1	Atypical_Hemolytic_Uremic_Syndrome_t2,Acid-base-electro_gene
KCNJ10	Ataxia_t2,Epilepsy_t2,Hearing_loss_t2,Atypical_Hemolytic_Uremic_Syndrome_t2,Hereditary_Autism_t2,Acid-base-electro_gene,Hereditary_hearing_loss
KCNJ11	Epilepsy_t2,Polycystic_Kidney_Disease_t2,Hereditary_Autism_t2,Maturity-Onset_Diabetes_of_the_Young,_MODY_t1,Monogenic_diabetes_panel
KCNJ13	Hereditary_retinopathy_t1,Leber_congenital_amaurosis,Vitreoretinopathy,Leber's_Congenital_Amaurosis_and_Related,Retinitis_Pigmentosa_and_Related,Vitreoretinopathy_and_Related,corneal_dystrophy,retina
KCNJ16	Acid-base-electro_gene
KCNJ2	Cardiomyopathy_t2,Muscular_dystrophy_t2,Myopathy_t2,Hereditary_Stroke_t2,Arrhythmia_t1,Arrhythmia,Dyskinesia-dystonia-paralysis,Arrhythmia_and_Related,Sudden_Cardiac_Arrest_and_Related
KCNJ5	Cardiomyopathy_t2,Atypical_Hemolytic_Uremic_Syndrome_t2,Arrhythmia_t1,Arrhythmia,Arrhythmia_and_Related
KCNJ8	Cardiomyopathy_t2,Arrhythmia_t1,Arrhythmia_and_Related
KCNK3	Arrhythmia_t2,Primary_Pulmonary_Hypertension_and_Related_Disordisorders
KCNK9	Hereditary_Autism_t2
KCNMA1	Epilepsy_t2,Dystonia_t2,Parkinson's_disease_t2,Dyskinesia-dystonia-paralysis
KCNN4	Anemia_t1,Hemolytic_anemia,Anemia
KCNQ1	Cardiomyopathy_t2,Hereditary_Stroke_t2,Arrhythmia_t1,Hearing_loss_t1,Arrhythmia,Hereditary_hearing_loss,Arrhythmia_and_Related,Sudden_Cardiac_Arrest_and_Related
KCNQ2	Arrhythmia_t2,Hereditary_Autism_t2,Epilepsy_t1,Early_onset_epilepsy,Epilepsy_and_Related
KCNQ3	Arrhythmia_t2,Epilepsy_t1,Early_onset_epilepsy,Focal_epilepsy,Epilepsy_and_Related
KCNQ4	Hearing_loss_t1,Hereditary_hearing_loss,Hearing_Loss_and_Related_t1,Hearing_Loss_and_Related_t2
KCNQ5	Epilepsy_t2
KCNT1	Arrhythmia_t2,Epilepsy_t1,Focal_epilepsy,Epilepsy_and_Related

Supplementary Table 2. List of pathogenic or likely-pathogenic germline variants

Gene	Associated diseases
KCNT2	Epilepsy_t2
KCNV2	Epilepsy_t2,Hereditary_retinopathy_t2,Cone-Rod_Dystrophy_and_Related,Retinitis_Pigmentosa_and_Related,retina
KCTD1	Dermatology_t2,Skeletal_dysplasia_t2
KCTD13	Hereditary_Autism_t2
KCTD17	Dystonia_t1,Dyskinesia-dystonia-paralysis,Dystonia,Dystonia_and_Related
KCTD3	Epilepsy_t2
KCTD7	Lysosomal_storage_disease_t2,Epilepsy_t2,Hereditary_retinopathy_t2,Hereditary_Autism_t2,Epilepsy_and_Related
KDM5B	Hereditary_Autism_t2
KDM5C	Hereditary_spastic_paraplegia_t2,Epilepsy_t2,Hereditary_Autism_t2,Neurodegenerative_disease_t2
KDM6A	Epilepsy_t2,Hereditary_Autism_t2,Proportionate_short_stature_t1,Cell-free_cancer,Hematologic_malignancy,Autoimmunity_and_autoinflammatory_disorders,Congenital_Hypothyroidism,Erythrocytosis,Severe_Combined_Immunodeficiency_and_CID
KDR	Cell-free_cancer
KDSR	Dermatology_t2
KEAP1	Cell-free_cancer
KERA	corneal_dystrophy
KIAA0586	Skeletal_dysplasia_t2,Hereditary_retinopathy_t2,Skeletal_Dysplasia_Panel
KIAA0753	Hereditary_retinopathy_t2,Skeletal_Dysplasia_Panel
KIAA1549	Hereditary_retinopathy_t2,Retinitis_pigmentosa_t1,Retinitis_pigmentosa,Retinitis_Pigmentosa_and_Related,retina
KIDINS220	Hereditary_spastic_paraplegia_t2,Neurodegenerative_disease_t2
KIF11	Hereditary_retinopathy_t2,Hereditary_Autism_t2,Hereditary_Microcephaly_t1,Pediatric_retinal_vascular_disease,Vitreoretinopathy,Vitreoretinopathy_and_Related,retina
KIF1A	Ataxia_t2,Hereditary_spastic_paraplegia_t2,Epilepsy_t2,Hereditary_Microcephaly_t2,Hereditary_Autism_t2,Neurodegenerative_disease_t2,Charcot-Marie-Tooth_disease_t1,Charcot_Marie_Tooth_and_Related,Optic_neuropathy_and_Related
KIF1B	Hereditary_cancer_syndrome_Plus_t2,Charcot-Marie-Tooth_disease_t1,Neuroendocrine_tumor_t1,Charcot-Marie-Tooth,Hereditary_paraganglioma-pheochromocytoma_syndrome,Charcot_Marie_Tooth_and_Related
KIF1C	Ataxia_t2,Hereditary_spastic_paraplegia_t2,Neurodegenerative_disease_t2
KIF21A	Hereditary_Autism_t2
KIF22	Skeletal_dysplasia_t2,Skeletal_Dysplasia_Panel
KIF23	Hemolytic_anemia,Anemia
KIF2A	Malformation_of_cortical_development
KIF5A	Charcot-Marie-Tooth_disease_t2,Ataxia_t2,Hereditary_Autism_t2,Neurodegenerative_disease_t2,Amyotrophic_lateral_sclerosis_t2,Hereditary_spastic_paraplegia_t1,Charcot-Marie-Tooth,Hereditary_spastic_paraplegia_panel,Charcot_Marie_Tooth_and_Related,Spastic_Paraplegia_and_Related
KIF5C	Malformation_of_cortical_development
KIF7	Skeletal_dysplasia_t2,Ataxia_t2,Hereditary_retinopathy_t2,Hereditary_Autism_t2,retina
KIFBP	Epilepsy_t2,Hereditary_Autism_t2,Hirschsprung's_disease
KIRREL3	Hereditary_Autism_t2
KISS1	Hypogonadotropic_hypogonadism_t1
KISS1R	Disorders_of_sexual_development_t1,Hypogonadotropic_hypogonadism_t1,Isolated_Hypogonadotropic_Hypogonadism
KIT	Dermatology_t2,Hearing_loss_t2,Cell-free_cancer,Hematologic_malignancy,Hereditary_pancancer,ALL_and_others_t1,ALL_and_others_t2,AML,Albinism_and_Related,Circulating_tumor_DNA_assay_55,Lymphoma_T_NK_cell_t1,Lymphoma_T_NK_cell_t2,MDS_MPN
KITLG	Dermatology_t2,Hearing_loss_t2,Hereditary_hearing_loss
KIZ	Hereditary_retinopathy_t2,Retinitis_pigmentosa_t1,Retinitis_pigmentosa,Retinitis_Pigmentosa_and_Related,retina

Supplementary Table 2. List of pathogenic or likely-pathogenic germline variants

Gene	Associated diseases
KL	Skeletal_dysplasia_t2,Sclerosing_Bone_Disease_Panel
KLF1	Anemia_t2,Hemolytic_anemia,Anemia
KLF11	Polycystic_Kidney_Disease_t2,Maturity-Onset_Diabetes_of_the_Young,_MODY_t1,Monogenic_diabetes_panel
KLF2	Lymphoma_t1,Lymphoma_B_cell_t1,Lymphoma_B_cell_t2
KLF8	Hereditary_Autism_t2
KLHL24	Cardiomyopathy_t2,Dermatology_t2
KLHL3	Acid-base-electro_gene
KLHL40	Muscular_dystrophy_t2,Myopathy_t1,Congenital_myopathy,Myofibrillar_myopathy,Myopathy_and_Related
KLHL41	Muscular_dystrophy_t2,Myopathy_t1,Myopathy_and_Related
KLHL6	Lymphoma_t2
KLHL7	Hereditary_retinopathy_t2,Retinitis_pigmentosa_t1,Retinitis_pigmentosa,Retinitis_Pigmentosa_and_Related,retina
KLKB1	Coagulation_t2,Atypical_Hemolytic_Uremic_Syndrome,Bleeding_Coagulopathy,Thrombosis
KMT2A	Skeletal_dysplasia_t2,Hereditary_Autism_t2,Hematologic_malignancy,ALL_and_others_t1,ALL_and_others_t2,Hereditary_Myeloid_Leukemia_Panel,Lymphoma_B_cell_t1,Lymphoma_B_cell_t2,Myeloma_t1
KMT2B	Parkinson's_disease_t2,Neurodegenerative_disease_t2,Dystonia_t1,Dystonia,Lymphoma_B_cell_t2,Dystonia_and_Related
KMT2C	Hematologic_malignancy
KMT2D	Epilepsy_t2,Hereditary_Microcephaly_t2,Hereditary_Autism_t2,Hereditary_cancer_syndrome_Plus_t2,Lymphoma_t2,Proportionate_short_stature_t1,Neuroendocrine_tumor_t1,Congenital_diarrhea,Hematologic_malignancy,Hereditary_paraganglioma-pheochromocytoma_syndrome,Severe_Combined_Immunodeficiency_and_CID,Autoimmunity_and_autoinflammatory_disorders,Congenital_Hypothyroidism,Lymphoma_B_cell_t1,Lymphoma_T_NK_cell_t1,Lymphoma_T_NK_cell_t2,Myeloma_t1
KMT2E	Epilepsy_t2,Hereditary_Autism_t2
KMT5B	Hereditary_Autism_t2
KNG1	Coagulation_t2,Atypical_Hemolytic_Uremic_Syndrome,Bleeding_Coagulopathy,Thrombosis
KNL1	Hereditary_Microcephaly_t1
KPNA7	Epilepsy_t2
KRAS	Dermatology_t2,Skeletal_dysplasia_t2,Proportionate_short_stature_t2,Epilepsy_t2,Hereditary_Autism_t2,Cardiomyopathy_t1,Rasopathies_t1,Cell-free_cancer,Hematologic_malignancy,Hereditary_pancancer,Noonan_syndrome,ALL_and_others_t1,ALL_and_others_t2,AML,Bone_Marrow_Failure_Germline_Predisposition,Cardiomyopathy_and_Related,Circulating_tumor_DNA_assay_55,Germline_Cancer,Lymphoma_B_cell_t1,Lymphoma_B_cell_t2,Lymphoma_T_NK_cell_t1,Lymphoma_T_NK_cell_t2,MDS_MPN,Myeloma_t1,Noonan_Syndrome_and_Related
KRIT1	Cerebral_cavernous_malformations,Primary_Pulmonary_Hypertension_and_Related_Disordisorders
KRT1	Dermatology_t2
KRT10	Dermatology_t2
KRT12	Hereditary_retinopathy_t2,Corneal_Dystrophy_and_Related,corneal_dystrophy
KRT14	Dermatology_t1
KRT16	Dermatology_t2
KRT17	Dermatology_t2
KRT2	Dermatology_t2
KRT3	Hereditary_retinopathy_t2,Corneal_Dystrophy_and_Related,corneal_dystrophy
KRT5	Dermatology_t1
KRT6A	Dermatology_t2
KRT6B	Dermatology_t2
KRT6C	Dermatology_t2
KRT71	Dermatology_t2

Supplementary Table 2. List of pathogenic or likely-pathogenic germline variants

Gene	Associated diseases
KRT72	corneal_keratoconus
KRT74	Dermatology_t2
KRT81	Dermatology_t2
KRT83	Dermatology_t2
KRT86	Dermatology_t2
KRT9	Dermatology_t2
KYNU	Skeletal_dysplasia_t2
L1CAM	Ataxia_t2,Hereditary_Autism_t2,Neurodegenerative_disease_t2,Hereditary_spastic_paraplegia_t1,Hereditary_spastic_paraplegia_panel,Hirschsprung's_disease,Spastic_Paraplegia_and_Related
L2HGDH	Hereditary_spastic_paraplegia_t2,Lysosomal_storage_disease_t2,Epilepsy_t2,Neurodegenerative_disease_t2
LAMA1	Ataxia_t2,Hereditary_retinopathy_t2,retina
LAMA2	Cardiomyopathy_t2,Myopathy_t2,Lysosomal_storage_disease_t2,Epilepsy_t2,Hereditary_Autism_t2,Muscular_dystrophy_t1,Congenital_muscular_dystrophy,Muscular_Dystrophy_and_Related
LAMA3	Dermatology_t1
LAMA4	Cardiomyopathy_t2,Dilated_cardiomyopathy
LAMB2	Congenital_myopathy,Glomerulopathy,Glomerular_basement_membrane_disorders,glaucoma
LAMB3	Dermatology_t1
LAMC1	corneal_dystrophy
LAMC2	Dermatology_t1
LAMC3	Hereditary_Autism_t2
LAMP2	Cardiomyopathy_t2,Muscular_dystrophy_t2,Myopathy_t2,Lysosomal_storage_disease_t2,Hereditary_Autism_t2,Dilated_cardiomyopathy,Hypertrophic_cardiomyopathy,Myofibrillar_myopathy,Retinitis_Pigmentosa_and_Related
LAMTOR2	Bone_Marrow_Failure_Germline_Predisposition
LARGE1	Cardiomyopathy_t2,Myopathy_t2,Epilepsy_t2,Hereditary_Autism_t2,Muscular_dystrophy_t1,Congenital_muscular_dystrophy,Muscular_Dystrophy_and_Related
LARP7	Skeletal_dysplasia_t2,Proportionate_short_stature_t2
LARS2	Ataxia_t2,Hearing_loss_t2,Hereditary_hearing_loss
LAS1L	Hereditary_Autism_t2
LBR	Skeletal_dysplasia_t2,Epilepsy_t2,Skeletal_Dysplasia_Panel
LBX1	Skeletal_dysplasia_t2
LCA5	Retinitis_pigmentosa_t2,Hereditary_retinopathy_t1,Leber_congenital_amaurosis,Leber's_Congenital_Amaurosis_and_Related,Retinitis_Pigmentosa_and_Related,retina
LCAT	Hereditary_Dyslipidemia_t1,Glomerulopathy,Dyslipidemia_and_Related
LCK	Primary_immune_deficiency_t2,Autoimmunity_and_autoinflammatory_disorders,Severe_Combined_Immunodeficiency_and_CID
LCT	Cholestasis_t2
LDB3	Arrhythmia_t2,Muscular_dystrophy_t2,Charcot-Marie-Tooth_disease_t2,Lysosomal_storage_disease_t2,Cardiomyopathy_t1,Myopathy_t1,Dilated_cardiomyopathy,Hypertrophic_cardiomyopathy,Myofibrillar_myopathy,Cardiomyopathy_and_Related,Myopathy_and_Related,Sudden_Cardiac_Arrest_and_Related
LDHA	Myopathy_t2,Lysosomal_storage_disease_t2,Metabolic_myopathy
LDHB	Hemolytic_anemia
LDLR	Hereditary_Stroke_t2,Hereditary_Dyslipidemia_t1,Dyslipidemia_and_Related
LDLRAP1	Hereditary_Dyslipidemia_t1,Dyslipidemia_and_Related
LEMD2	Arrhythmia_t2,Cardiomyopathy_t2,Hereditary_retinopathy_t2
LEMD3	Skeletal_dysplasia_t2,Sclerosing_Bone_Disease_Panel
LEP	Disorders_of_sexual_development_t2,Hypogonadotropic_hypogonadism_t2
LEPR	Disorders_of_sexual_development_t2,Hypogonadotropic_hypogonadism_t2

Supplementary Table 2. List of pathogenic or likely-pathogenic germline variants

Gene	Associated diseases
LFNG	Skeletal_dysplasia_t2,Proportionate_short_stature_t2
LGI1	Epilepsy_t2,Focal_epilepsy,Epilepsy_and_Related
LHB	Hypogonadotropic_hypogonadism_t1
LHCGR	Hypogonadotropic_hypogonadism_t2,Disorders_of_sexual_development_t1,Disorders_of_sex_development
LHFPL5	Hearing_loss_t2,Hereditary_hearing_loss
LHX3	Skeletal_dysplasia_t2,Disorders_of_sexual_development_t2,Hearing_loss_t2,Hereditary_Autism_t2,Proportionate_short_stature_t1,Hypothyroidism_t1,Hypogonadotropic_hypogonadism_t1,Congenital_Hypothyroidism
LHX4	Skeletal_dysplasia_t2,Proportionate_short_stature_t2
LIAS	Epilepsy_t2
LIFR	Skeletal_dysplasia_t2,Proportionate_short_stature_t2,Skeletal_Dysplasia_Panel
LIG4	Primary_immune_deficiency_t2,Hereditary_Microcephaly_t2,Hereditary_Autism_t2,Inflammatory_Bowel_t1,Hereditary_pancancer,Very-early-onset_inflammatory_bowel_disease,Bone_Marrow_Failure_Germline_Predisposition,Severe_Combined_Immunodeficiency_and_CID
LIM2	Hereditary_retinopathy_t2
LIMS2	Muscular_dystrophy_t2,Myopathy_t2,Limb_girdle_muscular_dystrophy
LIPA	Lysosomal_storage_disease_t1,Hereditary_Dyslipidemia_t1,Dyslipidemia_and_Related
LIPC	Hereditary_Dyslipidemia_t1,Dyslipidemia_and_Related
LIPG	Dyslipidemia_and_Related
LIPH	Dermatology_t2
LIPN	Dermatology_t1
LITAF	Charcot-Marie-Tooth_disease_t1,Charcot-Marie-Tooth,Charcot_Marie_Tooth_and_Related
LMAN1	Coagulation_t1,Atypical_Hemolytic_Uremic_Syndrome,Bleeding_Coagulopathy,Thrombosis
LMBR1	Skeletal_dysplasia_t2
LMBRD1	Lysosomal_storage_disease_t2,Inborn_error_of_metabolism_t2,Hereditary_Autism_t2
LMF1	Cholestasis_t2,Hereditary_Dyslipidemia_t1,Dyslipidemia_and_Related
LMNA	Arrhythmia_t2,Dermatology_t2,Skeletal_dysplasia_t2,Myopathy_t2,Cardiomyopathy_t1,Muscular_dystrophy_t1,Charcot-Marie-Tooth_disease_t1,Charcot-Marie-Tooth,Congenital_muscular_dystrophy,Dilated_cardiomyopathy,Limb_girdle_muscular_dystrophy,Arrhythmia_and_Related,Atypical_Hemolytic_Uremic_Syndrome,Bleeding_Coagulopathy,Cardiomyopathy_and_Related,Charcot_Marie_Tooth_and_Related,Muscular_Dystrophy_and_Related,Thrombosis
LMNB1	Ataxia_t2,Epilepsy_t2
LMNB2	Epilepsy_t2
LMOD2	Cardiomyopathy_t2
LMOD3	Muscular_dystrophy_t2,Myopathy_t2
LMX1A	Hearing_loss_t2,Hereditary_hearing_loss
LMX1B	Skeletal_dysplasia_t2,Glomerulopathy,Glomerular_basement_membrane_disorders,glaucoma
LONP1	Skeletal_dysplasia_t2,Skeletal_Dysplasia_Panel
LORICRIN	Dermatology_t2
LOX	Connective_tissue_disorder_t2,Familial_thoracic_aortic_aneurysms_and_aortic_dissections,Connective_Tissue_Disorder_and_Related,Marfan_and_Related,corneal_keratoconus
LOXHD1	Hearing_loss_t2,Hereditary_retinopathy_t2,Hereditary_hearing_loss,Hearing_Loss_and_Related_t2,corneal_dystrophy
LPAR6	Dermatology_t2
LPIN1	Myopathy_t2,Metabolic_myopathy
LPIN2	Anemia_t2,Skeletal_Dysplasia_Panel,Anemia
LPL	Hereditary_Dyslipidemia_t1,Stroke,Dyslipidemia_and_Related
LRAT	Retinitis_pigmentosa_t1,Hereditary_retinopathy_t1,Leber_congenital_amaurosis,Retinitis_pigmentosa,Leber's_Congenital_Amaurosis_and_Related,Retinitis_Pigmentosa_and_Related,retina

Supplementary Table 2. List of pathogenic or likely-pathogenic germline variants

Gene	Associated diseases
LRBA	Inflammatory_Bowel_t2,Primary_immune_deficiency_t1,Primary_immunodeficiency,Very-early-onset_inflammatory_bowel_disease,Autoimmunity_and_autoinflammatory_disorders,B_cell_and_Humoral_Immune_Deficiency
LRIT3	Hereditary_retinopathy_t2,Congenital_Stationary_Night_Blindness_and_Related,retina
LRMDA	Dermatology_t2,Hereditary_retinopathy_t2,Albinism_and_Related,Optic_neuropathy_and_Related
LRP2	Hearing_loss_t2,Hereditary_retinopathy_t2,Vitreoretinopathy,Vitreoretinopathy_and_Related,retina
LRP4	Skeletal_dysplasia_t2,Sclerosing_Bone_Disease_Panel
LRP5	Skeletal_dysplasia_t2,Hereditary_retinopathy_t2,Polycystic_Kidney_Disease_t1,Osteogenesis_imperfecta,Pediatric_retinal_vascular_disease,Sclerosing_Bone_Disease_Panel,Vitreoretinopathy,Polycystic_kidney_disease_and_Related,Vitreoretinopathy_and_Related,retina
LRP6	Dermatology_t2
LRPPRC	Ataxia_t2,Epilepsy_t2
LRRC10	Cardiomyopathy_t2
LRRK1	Sclerosing_Bone_Disease_Panel
LRRK2	Alzheimer's_disease_t2,Dystonia_t2,Dementia_t2,Parkinson's_disease_t1,Neurodegenerative_disease_t1,Parkinson,Parkinson_Disease_and_Related
LRSAM1	Ataxia_t2,Charcot-Marie-Tooth_disease_t1,Charcot-Marie-Tooth,Charcot_Marie_Tooth_and_Related
LRTOMT	Hearing_loss_t2,Hereditary_hearing_loss,Hearing_Loss_and_Related_t2
LSS	Hereditary_retinopathy_t2
LTBP2	Skeletal_dysplasia_t2,Proportionate_short_stature_t2,Hereditary_retinopathy_t2,Skeletal_Dysplasia_Panel,glaucoma
LTBP3	Dermatology_t2,Skeletal_dysplasia_t2,Ectodermal_dysplasia_Hypodontia,Skeletal_Dysplasia_Panel
LTBP4	Dermatology_t2,Connective_tissue_disorder_t2
LUC7L2	Hematologic_malignancy
LUM	Neurodegenerative_disease_t2,Amyotrophic_lateral_sclerosis_t2,corneal_dystrophy
LYRM7	Epilepsy_t2
LYST	Dermatology_t2,Lysosomal_storage_disease_t2,Coagulation_t2,Hereditary_retinopathy_t2,Hereditary_Autism_t2,Familial_hemophagocytic_lymphohistiocytosis,Albinism_and_Related,Bleeding_Platelet_Disorder,Bone_Marrow_Failure_Germline_Predisposition,Hemophagocytic_Lymphohistiocytosis_and_Related
LZTFL1	Hereditary_retinopathy_t2,Retinitis_Pigmentosa_and_Related,retina
LZTR1	Cardiomyopathy_t2,Dermatology_t2,Skeletal_dysplasia_t2,Proportionate_short_stature_t2,Rasopathies_t1,Noonan_syndrome,Noonan_Syndrome_and_Related
MAB21L2	Hereditary_retinopathy_t2
MACF1	Epilepsy_t2
MAD2L2	Congenital_hematologic_disease,Bone_Marrow_Failure_Germline_Predisposition
MAF	Hereditary_retinopathy_t2
MAFB	Skeletal_dysplasia_t2
MAG	Hereditary_spastic_paraplegia_t2,Neurodegenerative_disease_t2
MAGED2	Atypical_Hemolytic_Uremic_Syndrome_t2,Acid-base-electro_gene
MAGEL2	Hereditary_Autism_t2
MAGI2	Epilepsy_t2
MAGT1	Primary_immune_deficiency_t2,Atypical_Hemolytic_Uremic_Syndrome_t2,Hereditary_Autism_t2,Familial_hemophagocytic_lymphohistiocytosis,B_cell_and_Humoral_Immune_Deficiency,Hemophagocytic_Lymphohistiocytosis_and_Related,Hereditary_Myeloid_Leukemia_Panel,Severe_Combined_Immunodeficiency_and_CID
MAK	Hereditary_retinopathy_t2,Retinitis_pigmentosa_t1,Retinitis_pigmentosa,Retinitis_Pigmentosa_and_Related,retina
MALT1	Primary_immune_deficiency_t2
MAMLD1	Disorders_of_sexual_development_t2,Disorders_of_sex_development
MAN1B1	Lysosomal_storage_disease_t2,Hereditary_Autism_t2
MAN2B1	Lysosomal_storage_disease_t2,Hearing_loss_t2,Hereditary_Autism_t2,Skeletal_Dysplasia_Panel

Supplementary Table 2. List of pathogenic or likely-pathogenic germline variants

Gene	Associated diseases
MANBA	Lysosomal_storage_disease_t2,Hearing_loss_t2,Hereditary_Autism_t2,Skeletal_Dysplasia_Panel
MAOA	Hereditary_Autism_t2
MAP2K1	Dermatology_t2,Skeletal_dysplasia_t2,Proportionate_short_stature_t2,Epilepsy_t2,Hereditary_Autism_t2,Cardiomyopathy_t1,Rasopathies_t1,Cell-free_cancer,Hematologic_malignancy,Noonan_syndrome,Sclerosing_Bone_Disease_Panel,ALL_and_others_t1,ALL_and_others_t2,Cardiomyopathy_and_Related,Circulating_tumor_DNA_assay_55,Lymphoma_B_cell_t1,Lymphoma_B_cell_t2,Noonan_Syndrome_and_Related
MAP2K2	Cardiomyopathy_t2,Dermatology_t2,Skeletal_dysplasia_t2,Proportionate_short_stature_t2,Epilepsy_t2,Rasopathies_t1,Cell-free_cancer,Noonan_syndrome,ALL_and_others_t2,Circulating_tumor_DNA_assay_55,Noonan_Syndrome_and_Related
MAP3K1	Disorders_of_sexual_development_t2,Disorders_of_sex_development
MAP3K14	Primary_immune_deficiency_t2,Lymphoma_T_NK_cell_t2
MAP3K20	Muscular_dystrophy_t2,Myopathy_t2
MAP3K7	Skeletal_dysplasia_t2,Skeletal_Dysplasia_Panel
MAP3K8	Cardiomyopathy_t2,Rasopathies_t2
MAPK1	Cell-free_cancer,Lymphoma_B_cell_t2,Lymphoma_T_NK_cell_t2
MAPK10	Epilepsy_t2
MAPK3	Cell-free_cancer
MAPK8IP3	Hereditary_Autism_t2
MAPKAPK3	Macular_dystrophy,retina
MAPKBP1	Polycystic_Kidney_Disease_t2,Nephronophthisis_t1,Nephronophthisis_and_Related
MAPT	Alzheimer's_disease_t2,Parkinson's_disease_t2,Dementia_t1,Neurodegenerative_disease_t1,Amyotrophic_lateral_sclerosis_t1,Androgenetic_alopecia,Parkinson,Dementia_and_Related,Parkinson_Disease_and_Related
MARS1	Ataxia_t2,Hereditary_spastic_paraplegia_t2,Neurodegenerative_disease_t2,Charcot-Marie-Tooth_disease_t1,Charcot-Marie-Tooth,Charcot_Marie_Tooth_and_Related
MARS2	Ataxia_t2,Hereditary_spastic_paraplegia_t2,Epilepsy_t2,Dystonia_t2,Parkinson's_disease_t2,Neurodegenerative_disease_t2
MARVELD2	Hearing_loss_t2,Hereditary_hearing_loss,Hearing_Loss_and_Related_t2
MASP2	Very-early-onset_inflammatory_bowel_disease,Autoimmunity_and_autoinflammatory_disorders,B_cell_and_Humoral_Immune_Deficiency
MASTL	Coagulation_t2,Bleeding_Platelet_Disorder
MAT1A	Inborn_error_of_metabolism_t2,Hereditary_Autism_t2,Inborn_Error_of_Metabolism
MAT2A	Connective_tissue_disorder_t2,Familial_thoracic_aortic_aneurysms_and_aortic_dissections
MATN3	Skeletal_dysplasia_t1,Skeletal_Dysplasia_Panel,Spondyloepiphyseal_metaphyseal_dysplasia,Skeletal_Dysplasia_and_Related
MATR3	Muscular_dystrophy_t2,Myopathy_t2,Ataxia_t2,Neurodegenerative_disease_t2,Amyotrophic_lateral_sclerosis_t1,Amyotrophic_lateral_sclerosis,Myofibrillar_myopathy
MAX	Hereditary_cancer_syndrome_Plus_t2,Neuroendocrine_tumor_t1,Hereditary_pancancer,Hereditary_paraganglioma-pheochromocytoma_syndrome,Pheochromocytoma-Paraganglioma_Panel
MBD4	Hereditary_Myeloid_Leukemia_Panel
MBD5	Epilepsy_t2,Hereditary_Microcephaly_t2,Hereditary_Autism_t2,Autism_and_Related
MBNL1	Androgenetic_alopecia
MBOAT7	Epilepsy_t2,Hereditary_Autism_t2
MBTPS2	Dermatology_t2,Skeletal_dysplasia_t2,Hereditary_Autism_t2,Osteogenesis_imperfecta
MC1R	Dermatology_t2,Hereditary_retinopathy_t2,Albinism_and_Related
MCCC1	Inborn_error_of_metabolism_t2,Hereditary_Autism_t2,Inborn_Error_of_Metabolism
MCCC2	Inborn_error_of_metabolism_t2,Hereditary_Autism_t2,Inborn_Error_of_Metabolism
MCFD2	Coagulation_t1,Atypical_Hemolytic_Uremic_Syndrome,Bleeding_Coagulopathy,Thrombosis
MCIDAS	Hereditary_Primary_Ciliary_Dyskinesia_t2
MCM2	Hearing_loss_t2,Hereditary_hearing_loss
MCM3AP	Charcot-Marie-Tooth_disease_t2,Charcot-Marie-Tooth,Charcot_Marie_Tooth_and_Related

Supplementary Table 2. List of pathogenic or likely-pathogenic germline variants

Gene	Associated diseases
MCM4	Phagocyte_Defect_and_Infectious_disease
MCOLN1	Epilepsy_t2,Dystonia_t2,Parkinson's_disease_t2,Hereditary_Autism_t2,Lysosomal_storage_disease_t1
MCPH1	Epilepsy_t2,Hereditary_Autism_t2,Hereditary_Microcephaly_t1,Malformation_of_cortical_development
MDH2	Epilepsy_t2,Hereditary_cancer_syndrome_Plus_t2,Neuroendocrine_tumor_t1
MDM2	Cell-free_cancer,Lymphoma_B_cell_t2,Lymphoma_T_NK_cell_t2
MDM4	Lymphoma_B_cell_t2
ME2	Epilepsy_t2
MECOM	Coagulation_t2,Bleeding_Platelet_Disorder,Hereditary_Myeloid_Leukemia_Panel,Skeletal_Dysplasia_and_Related
MECP2	Hereditary_Microcephaly_t2,Hereditary_Autism_t2,Epilepsy_t1,Early_onset_epilepsy,Autism_and_Related,Epilepsy_and_Related
MECR	Ataxia_t2,Hereditary_retinopathy_t2,Dystonia_t1,Dystonia_and_Related
MED12	Cardiomyopathy_t2,Connective_tissue_disorder_t2,Epilepsy_t2,Hereditary_Autism_t2,Familial_thoracic_aortic_aneurysms_and_aortic_dissections
MED13L	Hereditary_Autism_t2
MED17	Epilepsy_t2,Hereditary_Microcephaly_t2,Hereditary_Autism_t2
MED23	Hereditary_Autism_t2
MED25	Ataxia_t2,Charcot-Marie-Tooth_disease_t1,Charcot-Marie-Tooth,Charcot_Marie_Tooth_and_Related
MEF2B	Lymphoma_t2,Hematologic_malignancy,Lymphoma_B_cell_t1,Lymphoma_B_cell_t2
MEF2C	Hereditary_Autism_t2,Epilepsy_t1,Early_onset_epilepsy,Epilepsy_and_Related
MEFV	Inflammatory_Bowel_t1,Autoinflammatory_disorders,Autoimmunity_and_autoinflammatory_disorders,B_cell_and_Humoral_Immune_Deficiency
MEGF10	Muscular_dystrophy_t2,Myopathy_t2,Myofibrillar_myopathy
MEGF8	Craniosynostosis
MEMO1	Androgenetic_alopecia
MEN1	Hereditary_cancer_syndrome_Plus_t1,Hereditary_pancancer,Pheochromocytoma-Paraganglioma_Panel
MERTK	Hereditary_retinopathy_t2,Hereditary_cancer_syndrome_Plus_t2,Retinitis_pigmentosa_t1,Neuroendocrine_tumor_t1,Hereditary_paraganglioma-pheochromocytoma_syndrome,Retinitis_pigmentosa,Cone-Rod_Dystrophy_and_Related,Leber's_Congenital_Amaurosis_and_Related,Retinitis_Pigmentosa_and_Related,retina
MESP2	Skeletal_dysplasia_t2
MET	Hearing_loss_t2,Hereditary_Autism_t2,Hereditary_cancer_syndrome_Plus_t2,Neuroendocrine_tumor_t1,Cell-free_cancer,Hereditary_hearing_loss,Hereditary_pancancer,Hereditary_paraganglioma-pheochromocytoma_syndrome,ALL_and_others_t2,Circulating_tumor_DNA_assay_55,Lymphoma_B_cell_t2
MFAP5	Connective_tissue_disorder_t2,Familial_thoracic_aortic_aneurysms_and_aortic_dissections,Connective_Tissue_Disorder_and_Related,Marfan_and_Related
MFN2	Ataxia_t2,Hereditary_retinopathy_t2,Charcot-Marie-Tooth_disease_t1,Charcot-Marie-Tooth,Charcot_Marie_Tooth_and_Related,Optic_neuropathy_and_Related,retina,CODA
MFRP	Retinitis_pigmentosa_t2,Hereditary_retinopathy_t2,Retinitis_Pigmentosa_and_Related,retina
MFSD2A	Hereditary_Microcephaly_t2
MFSD8	Lysosomal_storage_disease_t2,Epilepsy_t2,Hereditary_retinopathy_t2,Hereditary_Autism_t2,Epilepsy_and_Related,Macular_dystrophy_and_Related,retina
MGA	Lymphoma_T_NK_cell_t1,Lymphoma_T_NK_cell_t2
MGAT2	Epilepsy_t2,Hereditary_Autism_t2
MGP	Skeletal_dysplasia_t2,Hearing_loss_t2,Skeletal_Dysplasia_Panel
MIB1	Cardiomyopathy_t2
MICU1	Muscular_dystrophy_t2,Myopathy_t2
MID1	Hereditary_Autism_t2
MIP	Hereditary_retinopathy_t2
MIPEP	Cardiomyopathy_t2,Epilepsy_t2,Dystonia_t2,Hereditary_Microcephaly_t2
MIR140	Skeletal_Dysplasia_Panel
MIR17HG	Hereditary_Microcephaly_t2

Supplementary Table 2. List of pathogenic or likely-pathogenic germline variants

Gene	Associated diseases
MIR184	corneal_keratoconus
MIR96	Hearing_loss_t2,Hereditary_hearing_loss
MITF	Dermatology_t2,Hereditary_retinopathy_t2,Hearing_loss_t1,Hereditary_hearing_loss,Hereditary_pancancer,Albinism_and_Related,Hearing_Loss_and_Related_t1,Hearing_Loss_and_Related_t2
MKKS	Ataxia_t2,Disorders_of_sexual_development_t2,Retinitis_pigmentosa_t2,Hereditary_retinopathy_t2,Hereditary_Autism_t2,Hypogonadotropic_hypogonadism_t1,Retinitis_Pigmentosa_and_Related,retina
MKS1	Skeletal_dysplasia_t2,Ataxia_t2,Cholestasis_t2,Hereditary_retinopathy_t1,Retinitis_Pigmentosa_and_Related,retina
MLC1	Ataxia_t2,Epilepsy_t2
MLH1	Dermatology_t2,Hereditary_cancer_syndrome_Plus_t1,Cell-free_cancer,Congenital_hematologic_disease,Hereditary_colon_cancer,Hereditary_pancancer,Leukemia_predisposition,Breast_and_Ovarian_Cancer_Panel,Colorectal_Cancer_Polyposis_Panel,Germline_Cancer
MLH3	Hereditary_colon_cancer
MLPH	Dermatology_t2,Lysosomal_storage_disease_t2,Coagulation_t2,Hereditary_retinopathy_t2,Albinism_and_Related
MLYCD	Cardiomyopathy_t2,Inborn_error_of_metabolism_t2
MMAA	Inborn_error_of_metabolism_t1,Inborn_Error_of_Metabolism
MMAB	Inborn_error_of_metabolism_t1,Inborn_Error_of_Metabolism
MMACHC	Hereditary_spastic_paraplegia_t2,Inborn_error_of_metabolism_t2,Hereditary_retinopathy_t2,Hypogonadotropic_hypogonadism_t2,Neurodegenerative_disease_t2,Atypical_Hemolytic_Uremic_Syndrome_t1,DNAJC21_EFL1_SBDS_SRP54,Stroke,Atypical_Hemolytic_Uremic_Syndrome,Bleeding_Coagulopathy,Thrombosis
MMADHC	Inborn_error_of_metabolism_t2,Hereditary_Autism_t2,Atypical_Hemolytic_Uremic_Syndrome_t1
MME	Muscular_dystrophy_t2,Myopathy_t2,Charcot-Marie-Tooth_disease_t2,Ataxia_t2,Charcot-Marie-Tooth,Charcot_Marie_Tooth_and_Related
MMP1	Dermatology_t2
MMP13	Skeletal_dysplasia_t2,Skeletal_Dysplasia_Panel
MMP2	Skeletal_dysplasia_t2
MMP9	Skeletal_dysplasia_t2,Skeletal_Dysplasia_Panel
MMUT	Inborn_error_of_metabolism_t1,Atypical_Hemolytic_Uremic_Syndrome_t1,Stroke,Inborn_Error_of_Metabolism
MNX1	Maturity-Onset_Diabetes_of_the_Young,_MODY_t1,Monogenic_diabetes_panel
MOCS1	Lysosomal_storage_disease_t2,Epilepsy_t2
MOCS2	Lysosomal_storage_disease_t2,Epilepsy_t2,Hereditary_Autism_t2
MOGS	Epilepsy_t2,Primary_immune_deficiency_t2,B_cell_and_Humoral_Immune_Deficiency
MORC2	Charcot-Marie-Tooth_disease_t2,Charcot-Marie-Tooth,Charcot_Marie_Tooth_and_Related
MPDU1	Epilepsy_t2
MPDZ	corneal_dystrophy,corneal_keratoconus
MPL	Coagulation_t2,Cell-free_cancer,Congenital_hematologic_disease,Hematologic_malignancy,ALL_and_others_t1,ALL_and_others_t2,AML,Atypical_Hemolytic_Uremic_Syndrome,Bleeding_Coagulopathy,Bleeding_Platelet_Disorder,Bone_Marrow_Failure_Germline_Predisposition,Erythrocytosis,MDS_MPN,Thrombosis
MPLKIP	Dermatology_t2
MPO	Phagocyte_Defect_and_Infectious_disease
MPP1	Hemolytic_anemia
MPV17	Charcot-Marie-Tooth,Neonatal_cholestasis,Charcot_Marie_Tooth_and_Related
MPZ	Ataxia_t2,Charcot-Marie-Tooth_disease_t1,Charcot-Marie-Tooth,Charcot_Marie_Tooth_and_Related
MPZL2	Hearing_loss_t2,Hereditary_hearing_loss
MRAP	Hereditary_Autism_t2
MRAS	Rasopathies_t2
MRE11	Ataxia_t2,Dystonia_t2,Parkinson's_disease_t2,Hereditary_Microcephaly_t2,Hereditary_cancer_syndrome_Plus_t2,Hereditary_pancancer

Supplementary Table 2. List of pathogenic or likely-pathogenic germline variants

Gene	Associated diseases
MRPL44	Epilepsy_t2
MRPS22	Androgenetic_alopecia
MS4A1	B_cell_and_Humoral_Immune_Deficiency
MSH2	Dermatology_t2,Hereditary_cancer_syndrome_Plus_t1,Cell-free_cancer,Congenital_hematologic_disease,Hereditary_colon_cancer,Hereditary_pancancer,Leukemia_predisposition,Breast_and_Ovarian_Cancer_Panel,Colorectal_Cancer_Polyposis_Panel,Germline_Cancer
MSH3	Colorectal_Cancer_Polyposis_Panel,Germline_Cancer
MSH6	Dermatology_t2,Hereditary_cancer_syndrome_Plus_t1,Cell-free_cancer,Congenital_hematologic_disease,Hereditary_colon_cancer,Hereditary_pancancer,Leukemia_predisposition,Breast_and_Ovarian_Cancer_Panel,Colorectal_Cancer_Polyposis_Panel,Germline_Cancer
MSL3	Hereditary_Autism_t2
MSMO1	Hereditary_Microcephaly_t2
MSRB3	Hearing_loss_t2,Hereditary_hearing_loss
MSTO1	Muscular_dystrophy_t2,Myopathy_t2,Ataxia_t2
MSX1	Dermatology_t2,Ectodermal_dysplasia_Hypodontia
MSX2	Skeletal_dysplasia_t2,Craniosynostosis
MT-ATP6	Mitochondrial_DNA_t1,mitochondria
MT-ATP8	Mitochondrial_DNA_t1,mitochondria
MT-CO1	Hearing_loss_t2,Mitochondrial_DNA_t1,mitochondria
MT-CO2	Mitochondrial_DNA_t1,mitochondria
MT-CO3	Mitochondrial_DNA_t1,mitochondria
MT-CYB	Mitochondrial_DNA_t1,mitochondria
MT-ND1	Cardiomyopathy_t2,Mitochondrial_DNA_t1,mitochondria
MT-ND2	Mitochondrial_DNA_t1,mitochondria
MT-ND3	Mitochondrial_DNA_t1,mitochondria
MT-ND4	Mitochondrial_DNA_t1,mitochondria,retina
MT-ND4L	Mitochondrial_DNA_t1,mitochondria
MT-ND5	Cardiomyopathy_t2,Mitochondrial_DNA_t1,mitochondria
MT-ND6	Cardiomyopathy_t2,Mitochondrial_DNA_t1,mitochondria
MT-RNR1	Hearing_loss_t2,Mitochondrial_DNA_t1,mitochondria
MT-RNR2	Mitochondrial_DNA_t2,mitochondria
MT-TA	Mitochondrial_DNA_t2,mitochondria
MT-TC	Mitochondrial_DNA_t2,mitochondria
MT-TD	Cardiomyopathy_t2,Mitochondrial_DNA_t2,mitochondria
MT-TE	Mitochondrial_DNA_t1,mitochondria
MT-TF	Mitochondrial_DNA_t1,mitochondria
MT-TG	Cardiomyopathy_t2,Mitochondrial_DNA_t2,mitochondria
MT-TH	Cardiomyopathy_t2,Mitochondrial_DNA_t1,mitochondria
MT-TI	Cardiomyopathy_t2,Mitochondrial_DNA_t1,mitochondria
MT-TK	Cardiomyopathy_t2,Mitochondrial_DNA_t1,mitochondria
MT-TL1	Cardiomyopathy_t2,Hearing_loss_t2,Mitochondrial_DNA_t1,mitochondria,retina
MT-TL2	Cardiomyopathy_t2,Mitochondrial_DNA_t2,mitochondria

Supplementary Table 2. List of pathogenic or likely-pathogenic germline variants

Gene	Associated diseases
MT-TM	Cardiomyopathy_t2,Mitochondrial_DNA_t2,mitochondria
MT-TN	Mitochondrial_DNA_t2,mitochondria
MT-TP	Mitochondrial_DNA_t1,mitochondria
MT-TQ	Cardiomyopathy_t2,Mitochondrial_DNA_t2,mitochondria
MT-TR	Mitochondrial_DNA_t2,mitochondria
MT-TS1	Cardiomyopathy_t2,Hearing_loss_t2,Mitochondrial_DNA_t1,mitochondria
MT-TS2	Cardiomyopathy_t2,Mitochondrial_DNA_t1,mitochondria,retina
MT-TT	Mitochondrial_DNA_t1,mitochondria
MT-TV	Mitochondrial_DNA_t1,mitochondria
MT-TW	Mitochondrial_DNA_t1,mitochondria
MT-TY	Mitochondrial_DNA_t2,mitochondria
MTAP	Sclerosing_Bone_Disease_Panel
MTFMT	Ataxia_t2,Epilepsy_t2,Hereditary_Autism_t2
MTHFR	Hereditary_spastic_paraplegia_t2,Inborn_error_of_metabolism_t2,Epilepsy_t2,Hereditary_Autism_t2,Neurodegenerative_disease_t2,Stroke,Atypical_Hemolytic_Uremic_Syndrome,Bleeding_Coagulopathy,Thrombosis,corneal_dystrophy
MTM1	Muscular_dystrophy_t2,Hereditary_Autism_t2,Myopathy_t1,Congenital_myopathy,Myopathy_and_Related
MTMR2	Charcot-Marie-Tooth_disease_t1,Charcot-Marie-Tooth,Charcot_Marie_Tooth_and_Related
MTO1	Cardiomyopathy_t2
MTOR	Epilepsy_t2,Hereditary_Autism_t2,Cell-free_cancer,Focal_epilepsy,Malformation_of_cortical_development,Overgrowth_intellectual_disability,Circulating_tumor_DNA_assay_55,Lymphoma_B_cell_t2,Lymphoma_T_NK_cell_t2,Myeloma_t1
MTPAP	Ataxia_t2,Hereditary_retinopathy_t2
MTR	Inborn_error_of_metabolism_t2,Hereditary_Autism_t2
MTRFR	Charcot-Marie-Tooth_disease_t2,Ataxia_t2,Hereditary_spastic_paraplegia_t2,Hereditary_retinopathy_t2,Neurodegenerative_disease_t2,Charcot_Marie_Tooth_and_Related,Optic_neuropathy_and_Related
MTRR	Inborn_error_of_metabolism_t2
MTTP	Ataxia_t2,Hereditary_retinopathy_t2,Hereditary_Dyslipidemia_t1,Ataxia,Congenital_diarrhea,Dyslipidemia_and_Related,retina
MUC1	Polycystic_Kidney_Disease_t2
MUSK	Congenital_myopathy
MUTYH	Hereditary_cancer_syndrome_Plus_t1,Hereditary_breast_cancer,Hereditary_colon_cancer,Hereditary_pancancer,Colorectal_Cancer_Polyposis_Panel
MVK	Hereditary_retinopathy_t2,Retinitis_pigmentosa_t1,Inflammatory_Bowel_t1,Autoinflammatory_disorders,Familial_hemophagocytic_lymphohistiocytosis,Retinitis_pigmentosa,Very-early-onset_inflammatory_bowel_disease,Autoimmunity_and_autoinflammatory_disorders,B_cell_and_Humoral_Immune_Deficiency,Hemophagocytic_Lymphohistiocytosis_and_Related,Retinitis_Pigmentosa_and_Related,retina
MYBPC3	Cardiomyopathy_t1,Dilated_cardiomyopathy,Hypertrophic_cardiomyopathy,Cardiomyopathy_and_Related,Sudden_Cardiac_Arrest_and_Related
MYBPHL	Cardiomyopathy_t2
MYC	Lymphoma_t1,Cell-free_cancer,Hematologic_malignancy,Circulating_tumor_DNA_assay_55,Lymphoma_B_cell_t1,Lymphoma_B_cell_t2,Lymphoma_T_NK_cell_t1,Lymphoma_T_NK_cell_t2,Myeloma_t1
MYCN	Skeletal_dysplasia_t2,Hereditary_Microcephaly_t2,Hereditary_Autism_t2,Cell-free_cancer,Circulating_tumor_DNA_assay_55
MYD88	Lymphoma_t1,Hematologic_malignancy,ALL_and_others_t1,ALL_and_others_t2,AML,Lymphoma_B_cell_t1,Lymphoma_B_cell_t2,Lymphoma_T_NK_cell_t1,Lymphoma_T_NK_cell_t2,Myeloma_t1,Phagocyte_Defect_and_Infectious_disease
MYF6	Muscular_dystrophy_t2,Myopathy_t2
MYH11	Connective_tissue_disorder_t1,Familial_thoracic_aortic_aneurysms_and_aortic_dissections,Connective_Tissue_Disorder_and_Related,Marfan_and_Related
MYH14	Hearing_loss_t2,Hereditary_hearing_loss,Charcot_Marie_Tooth_and_Related,Hearing_Loss_and_Related_t2
MYH2	Muscular_dystrophy_t2,Myopathy_t2,Myofibrillar_myopathy

Supplementary Table 2. List of pathogenic or likely-pathogenic germline variants

Gene	Associated diseases
MYH3	Myopathy_t2,Skeletal_Dysplasia_Panel
MYH6	Arrhythmia_t2,Cardiomyopathy_t1,Dilated_cardiomyopathy,Hypertrophic_cardiomyopathy,Cardiomyopathy_and_Related
MYH7	Arrhythmia_t2,Muscular_dystrophy_t2,Cardiomyopathy_t1,Myopathy_t1,Congenital_myopathy,Dilated_cardiomyopathy,Hypertrophic_cardiomyopathy,Cardiomyopathy_and_Related,Myopathy_and_Related,Sudden_Cardiac_Arrest_and_Related
MYH9	Coagulation_t2,Hearing_loss_t2,Alport_Syndrome_t1,Glomerulopathy,Hereditary_hearing_loss,Bleeding_Platelet_Disorder,Glomerular_basement_membrane_disorders
MYL2	Cardiomyopathy_t2,Muscular_dystrophy_t2,Myopathy_t2,Hypertrophic_cardiomyopathy
MYL3	Cardiomyopathy_t1,Hypertrophic_cardiomyopathy,Cardiomyopathy_and_Related,Sudden_Cardiac_Arrest_and_Related
MYL4	Arrhythmia_t2,Cardiomyopathy_t2
MYLIP	Dyslipidemia_and_Related
MYLK	Connective_tissue_disorder_t2,Familial_thoracic_aortic_aneurysms_and_aortic_dissections,Connective_Tissue_Disorder_and_Related,Marfan_and_Related
MYLK2	Cardiomyopathy_t2,Hypertrophic_cardiomyopathy
MYO15A	Hearing_loss_t1,Hereditary_hearing_loss,Hearing_Loss_and_Related_t1,Hearing_Loss_and_Related_t2
MYO18B	Skeletal_dysplasia_t2,Muscular_dystrophy_t2,Myopathy_t2,Hereditary_Microcephaly_t2
MYO1A	Hereditary_hearing_loss
MYO1C	Hearing_loss_t2
MYO3A	Hearing_loss_t2,Hereditary_hearing_loss,Hearing_Loss_and_Related_t2
MYO5A	Dermatology_t2,Lysosomal_storage_disease_t2,Coagulation_t2,Hereditary_retinopathy_t2,Hereditary_Autism_t2,Familial_hemophagocytic_lymphohistiocytosis,Albinism_and_Related,Hemophagocytic_Lymphohistiocytosis_and_Related
MYO5B	Cholestasis_t2,Congenital_diarrhea
MYO6	Hearing_loss_t2,Hereditary_hearing_loss,Hearing_Loss_and_Related_t2
MYO7A	Hearing_loss_t1,Hereditary_retinopathy_t1,Hereditary_hearing_loss,Hearing_Loss_and_Related_t1,Hearing_Loss_and_Related_t2,Leber's_Congenital_Amaurosis_and_Related,Retinitis_Pigmentosa_and_Related,retina
MYOC	Hereditary_retinopathy_t2,glaucoma
MYOM1	Cardiomyopathy_t2
MYORG	Fahr's_disease
MYOT	Cardiomyopathy_t2,Charcot-Marie-Tooth_disease_t2,Lysosomal_storage_disease_t2,Muscular_dystrophy_t1,Myopathy_t1,Limb_girdle_muscular_dystrophy,Myofibrillar_myopathy,Muscular_Dystrophy_and_Related,Myopathy_and_Related
MYOZ2	Cardiomyopathy_t2,Hypertrophic_cardiomyopathy
MYPN	Cardiomyopathy_t2,Muscular_dystrophy_t2,Myopathy_t2,Dilated_cardiomyopathy
MYRF	Cardiomyopathy_t2
MYT1L	Hereditary_Autism_t2
NAA10	Hereditary_Autism_t2
NACC1	Epilepsy_t2
NAF1	Congenital_hematologic_disease,Dyskeratosis_congenita,Hereditary_Myeloid_Leukemia_Panel
NAGA	Lysosomal_storage_disease_t2,Hereditary_Autism_t2
NAGLU	Lysosomal_storage_disease_t2,Epilepsy_t2,Charcot-Marie-Tooth,Mucopolysaccharidosis,Skeletal_Dysplasia_Panel,Charcot_Marie_Tooth_and_Related,Lysosomal_Storage_Disease
NAGS	Urea_cycle_disorders
NALCN	Epilepsy_t2,Hereditary_Autism_t2
NANS	Skeletal_dysplasia_t2,Skeletal_Dysplasia_Panel
NARS2	Hearing_loss_t2,Hereditary_hearing_loss
NAT2	Hereditary_pancancer
NBAS	Osteogenesis_imperfecta

Supplementary Table 2. List of pathogenic or likely-pathogenic germline variants

Gene	Associated diseases
NBEA	Coagulation_t2,Epilepsy_t2,Hereditary_Autism_t2
NBEAL2	Coagulation_t2,Bleeding_Platelet_Disorder
NBN	Coagulation_t2,Primary_immune_deficiency_t2,Hereditary_Microcephaly_t2,Hereditary_Autism_t2,Hereditary_cancer_syndrome_Plus_t1,Congenital_hematologic_disease,Hereditary_breast_cancer,Hereditary_pancancer,Leukemia_predisposition,Bone_Marrow_Failure_Germline_Predisposition,Breast_and_Ovarian_Cancer_Panel,Hereditary_Myeloid_Leukemia_Panel
NCAPD3	Hereditary_Microcephaly_t2
NCF1	Inflammatory_Bowel_t2,Very-early-onset_inflammatory_bowel_disease,Autoimmunity_and_autoinflammatory_disorders,B_cell_and_Humoral_Immune_Deficiency,Phagocyte_Defect_and_Infectious_disease
NCF2	Inflammatory_Bowel_t1,Primary_immunodeficiency,Very-early-onset_inflammatory_bowel_disease,Autoimmunity_and_autoinflammatory_disorders,B_cell_and_Humoral_Immune_Deficiency,Phagocyte_Defect_and_Infectious_disease
NCF4	Primary_immunodeficiency,Very-early-onset_inflammatory_bowel_disease,Autoimmunity_and_autoinflammatory_disorders,B_cell_and_Humoral_Immune_Deficiency,Phagocyte_Defect_and_Infectious_disease
NDE1	Epilepsy_t2,Hereditary_Microcephaly_t1,Malformation_of_cortical_development
NDN	Hypogonadotropic_hypogonadism_t2
NDP	Hearing_loss_t2,Hereditary_retinopathy_t2,Hereditary_Autism_t2,Hereditary_hearing_loss,Pediatric_retinal_vascular_disease,Vitreoretinopathy,Hearing_Loss_and_Related_t2,Vitreoretinopathy_and_Related,retina,glaucoma
NDRG1	Ataxia_t2,Charcot-Marie-Tooth_disease_t1,Charcot-Marie-Tooth,Charcot_Marie_Tooth_and_Related
NDST1	Epilepsy_t2
NDUFA1	Epilepsy_t2,Hereditary_Autism_t2,Leigh_Syndrome_and_Related
NDUFA10	Leigh_Syndrome_and_Related,Optic_neuropathy_and_Related
NDUFA11	Leigh_Syndrome_and_Related,Optic_neuropathy_and_Related
NDUFA12	Leigh_Syndrome_and_Related,Optic_neuropathy_and_Related
NDUFA13	Optic_neuropathy_and_Related
NDUFA2	Epilepsy_t2,Leigh_Syndrome_and_Related,Optic_neuropathy_and_Related
NDUFA4	Leigh_Syndrome_and_Related
NDUFA9	Leigh_Syndrome_and_Related,Optic_neuropathy_and_Related
NDUFAF2	Cardiomyopathy_t2,Leigh_Syndrome_and_Related,Optic_neuropathy_and_Related
NDUFAF3	Epilepsy_t2
NDUFAF5	Epilepsy_t2,Hereditary_Autism_t2,Leigh_Syndrome_and_Related,Optic_neuropathy_and_Related
NDUFAF6	Ataxia_t2,Epilepsy_t2,Leigh_Syndrome_and_Related,Optic_neuropathy_and_Related
NDUFB11	Cardiomyopathy_t2
NDUFS1	Ataxia_t2,Epilepsy_t2,Hereditary_retinopathy_t2,Hereditary_Autism_t2,Leigh_Syndrome_and_Related,Optic_neuropathy_and_Related
NDUFS2	Ataxia_t2,Epilepsy_t2,Leigh_Syndrome_and_Related,Optic_neuropathy_and_Related
NDUFS3	Epilepsy_t2,Leigh_Syndrome_and_Related,Optic_neuropathy_and_Related
NDUFS4	Ataxia_t2,Epilepsy_t2,Leigh_Syndrome_and_Related,Optic_neuropathy_and_Related
NDUFS6	Epilepsy_t2
NDUFS7	Ataxia_t2,Epilepsy_t2,Leigh_Syndrome_and_Related,Optic_neuropathy_and_Related
NDUFS8	Ataxia_t2,Epilepsy_t2,Leigh_Syndrome_and_Related,Optic_neuropathy_and_Related
NDUFV1	Ataxia_t2,Epilepsy_t2,Leigh_Syndrome_and_Related,Optic_neuropathy_and_Related
NEB	Muscular_dystrophy_t2,Myopathy_t1,Congenital_myopathy,Myofibrillar_myopathy,Myopathy_and_Related
NEBL	Cardiomyopathy_t2,Dilated_cardiomyopathy
NECAP1	Epilepsy_t2
NECTIN4	Dermatology_t2

Supplementary Table 2. List of pathogenic or likely-pathogenic germline variants

Gene	Associated diseases
NEDD4L	Epilepsy_t2
NEFH	Charcot-Marie-Tooth_disease_t2,Neurodegenerative_disease_t2,Amyotrophic_lateral_sclerosis_t2,Amyotrophic_lateral_sclerosis,Charcot-Marie-Tooth,Charcot_Marie_Tooth_and_Related
NEFL	Ataxia_t2,Charcot-Marie-Tooth_disease_t1,Charcot-Marie-Tooth,Charcot_Marie_Tooth_and_Related
NEGR1	Hereditary_Autism_t2
NEIL1	corneal_dystrophy
NEK1	Skeletal_dysplasia_t2,Neurodegenerative_disease_t2,Amyotrophic_lateral_sclerosis_t1,Skeletal_Dysplasia_Panel
NEK2	Hereditary_retinopathy_t2,Retinitis_pigmentosa_t1,Retinitis_pigmentosa,Retinitis_Pigmentosa_and_Related,retina
NEK8	Polycystic_Kidney_Disease_t2,Nephronophthisis_t1,Nephronophthisis_and_Related
NEU1	Lysosomal_storage_disease_t2,Epilepsy_t2,Skeletal_Dysplasia_Panel
NEUROD1	Hereditary_retinopathy_t2,Polycystic_Kidney_Disease_t2,Retinitis_pigmentosa_t1,Maturity-Onset_Diabetes_of_the_Young,_MODY_t1,Monogenic_diabetes_panel,Retinitis_pigmentosa,Retinitis_Pigmentosa_and_Related,retina
NEUROD2	Epilepsy_t2
NEUROG3	Cholestasis_t2,Maturity-Onset_Diabetes_of_the_Young,_MODY_t1,Congenital_diarrhea,Monogenic_diabetes_panel
NEXMIF	Epilepsy_t2,Hereditary_Autism_t2
NEXN	Cardiomyopathy_t2,Dilated_cardiomyopathy,Hypertrophic_cardiomyopathy
NF1	Cardiomyopathy_t2,Dermatology_t2,Skeletal_dysplasia_t2,Epilepsy_t2,Hereditary_Autism_t2,Rasopathies_t1,Hereditary_cancer_syndrome_Plus_t1,Neuroendocrine_tumor_t1,Cell-free_cancer,Congenital_hematologic_disease,Hematologic_malignancy,Hereditary_breast_cancer,Hereditary_pancancer,Hereditary_paraganglioma-pheochromocytoma_syndrome,Leukemia_predisposition,ALL_and_others_t1,ALL_and_others_t2,AML,Bone_Marrow_Failure_Germline_Predisposition,Breast_and_Ovarian_Cancer_Panel,Germline_Cancer,Hereditary_Myeloid_Leukemia_Panel,Lymphoma_B_cell_t2,MDS_MPN,Pheochromocytoma-Paraganglioma_Panel,glaucoma
NF2	Dermatology_t2,Rasopathies_t2,Hearing_loss_t2,Hereditary_cancer_syndrome_Plus_t2,Cell-free_cancer,Hereditary_pancancer,Germline_Cancer
NFAT5	Autoimmunity_and_autoinflammatory_disorders,B_cell_and_Humoral_Immune_Deficiency,Phagocyte_Defect_and_Infectious_disease
NFE2L2	Cell-free_cancer
NFIB	Hereditary_Autism_t2
NFIX	Skeletal_dysplasia_t2,Overgrowth_intellectual_disability,Overgrowth_Panel
NFKB1	Lymphoma_t2,Primary_immunodeficiency,Autoimmunity_and_autoinflammatory_disorders,B_cell_and_Humoral_Immune_Deficiency
NFKB2	Primary_immune_deficiency_t1,Primary_immunodeficiency,B_cell_and_Humoral_Immune_Deficiency
NFKBIA	Dermatology_t2,Primary_immune_deficiency_t2,Ectodermal_dysplasia_Hypodontia,Severe_Combined_Immunodeficiency_and_CID
NFKBIE	Lymphoma_t1
NFU1	Epilepsy_t2
NGF	Hereditary_Autism_t2,Charcot-Marie-Tooth_disease_t1,Charcot_Marie_Tooth_and_Related
NGLY1	Epilepsy_t2,Hereditary_Autism_t2
NHEJ1	Primary_immune_deficiency_t2,Hereditary_Autism_t2,Hereditary_Microcephaly_t1,Severe_Combined_Immunodeficiency_and_CID
NHLRC1	Epilepsy_t2,Epilepsy_and_Related
NHP2	Dermatology_t2,Coagulation_t2,Primary_immune_deficiency_t2,Hereditary_Autism_t2,Congenital_hematologic_disease,Dyskeratosis_congenita,Bone_Marrow_Failure_Germline_Predisposition
NHS	Hereditary_retinopathy_t2,Hereditary_Autism_t2,glaucoma
NIN	Hereditary_Microcephaly_t2
NIPA1	Ataxia_t2,Neurodegenerative_disease_t2,Hereditary_spastic_paraplegia_t1,Hereditary_spastic_paraplegia_panel,Spastic_Paraplegia_and_Related
NIPAL4	Dermatology_t1
NIPBL	Skeletal_dysplasia_t2,Epilepsy_t2,Hereditary_Microcephaly_t2,Hereditary_Autism_t2,Proportionate_short_stature_t1
NKX2-1	Dystonia_t2,Parkinson's_disease_t2,Hypothyroidism_t1,Chorea,Congenital_Hypothyroidism
NKX2-2	Maturity-Onset_Diabetes_of_the_Young,_MODY_t1,Monogenic_diabetes_panel
NKX2-5	Arrhythmia_t2,Cardiomyopathy_t2,Hypothyroidism_t1,Congenital_Hypothyroidism

Supplementary Table 2. List of pathogenic or likely-pathogenic germline variants

Gene	Associated diseases
NKX3-2	Skeletal_dysplasia_t2,Sclerosing_Bone_Disease_Panel,Skeletal_Dysplasia_Panel
NKX6-2	Ataxia_t2,Hereditary_spastic_paraplegia_t2,Epilepsy_t2,Neurodegenerative_disease_t2
NLGN3	Hereditary_Autism_t2
NLGN4X	Hereditary_Autism_t1
NLRC4	Autoimmunity_and_autoinflammatory_disorders,B_cell_and_Humoral_Immune_Deficiency
NLRP1	Autoinflammatory_disorders,Autoimmunity_and_autoinflammatory_disorders,B_cell_and_Humoral_Immune_Deficiency
NLRP12	Autoinflammatory_disorders,Autoimmunity_and_autoinflammatory_disorders,B_cell_and_Humoral_Immune_Deficiency
NLRP3	Hearing_loss_t2,Autoinflammatory_disorders,Hereditary_hearing_loss,Skeletal_Dysplasia_Panel,Autoimmunity_and_autoinflammatory_disorders,B_cell_and_Humoral_Immune_Deficiency
NLRP7	Autoinflammatory_disorders
NME5	Hereditary_Primary_Ciliary_Dyskinesia_t2
NME8	Hereditary_Primary_Ciliary_Dyskinesia_t1
NMNAT1	Retinitis_pigmentosa_t2,Hereditary_retinopathy_t1,Leber_congenital_amaurosis,Leber's_Congenital_Amaurosis_and_Related,Retinitis_Pigmentosa_and_Related,retina
NOD2	Autoinflammatory_disorders,Autoimmunity_and_autoinflammatory_disorders,B_cell_and_Humoral_Immune_Deficiency
NOG	Skeletal_dysplasia_t2
NOL3	Ataxia_t2,Epilepsy_t2
NONO	Cardiomyopathy_t2,Hereditary_Autism_t2
NOP10	Dermatology_t2,Coagulation_t2,Primary_immune_deficiency_t2,Congenital_hematologic_disease,Dyskeratosis_congenita,Bone_Marrow_Failure_Germline_Predisposition
NOS1AP	Arrhythmia_t2
NOTCH1	Dermatology_t2,Skeletal_dysplasia_t2,Connective_tissue_disorder_t2,Lymphoma_t1,Cell-free_cancer,Familial_thoracic_aortic_aneurysms_and_aortic_dissections,Hematologic_malignancy,ALL_and_others_t2,Lymphoma_B_cell_t1,Lymphoma_B_cell_t2,Lymphoma_T_NK_cell_t1,Lymphoma_T_NK_cell_t2
NOTCH2	Skeletal_dysplasia_t2,Proportionate_short_stature_t2,Polycystic_Kidney_Disease_t1,Cholestasis_t1,Lymphoma_t1,Hematologic_malignancy,Neonatal_cholestasis,Lymphoma_B_cell_t1,Lymphoma_B_cell_t2,Polycystic_kidney_disease_and_Related,glaucoma
NOTCH3	Ataxia_t2,Epilepsy_t2,Alzheimer's_disease_t2,Dementia_t2,Parkinson's_disease_t2,Neurodegenerative_disease_t2,Hereditary_Stroke_t1,Stroke,Dementia_and_Related
NPC1	Epilepsy_t2,Dystonia_t2,Parkinson's_disease_t2,Hereditary_Autism_t2,Inflammatory_Bowel_t2,Lysosomal_storage_disease_t1,Cholestasis_t1,Ataxia,Neonatal_cholestasis,Parkinson_Disease_and_Related
NPC2	Epilepsy_t2,Dystonia_t2,Parkinson's_disease_t2,Hereditary_Autism_t2,Lysosomal_storage_disease_t1,Cholestasis_t1,Neonatal_cholestasis,Parkinson_Disease_and_Related
NPHP1	Ataxia_t2,Epilepsy_t2,Retinitis_pigmentosa_t2,Polycystic_Kidney_Disease_t2,Cholestasis_t2,Hereditary_retinopathy_t1,Nephronophthisis_t1,Nephronophthisis_and_Related,Retinitis_Pigmentosa_and_Related,retina
NPHP3	Retinitis_pigmentosa_t2,Hereditary_retinopathy_t2,Hereditary_Autism_t2,Cholestasis_t2,Nephronophthisis_t1,Polycystic_Kidney_Disease_t1,Nephronophthisis_and_Related,Polycystic_kidney_disease_and_Related,Retinitis_Pigmentosa_and_Related,retina
NPHP4	Retinitis_pigmentosa_t2,Hereditary_retinopathy_t2,Polycystic_Kidney_Disease_t2,Cholestasis_t2,Nephronophthisis_t1,Nephronophthisis_and_Related,Retinitis_Pigmentosa_and_Related,retina
NPHS1	Glomerulopathy,Nephronophthisis_and_Related,glaucoma
NPHS2	Glomerulopathy
NPM1	Cell-free_cancer,Hematologic_malignancy,ALL_and_others_t1,ALL_and_others_t2,AML,MDS_MPN
NPPA	Arrhythmia_t2,Cardiomyopathy_t2
NPR2	Skeletal_dysplasia_t2,Proportionate_short_stature_t2,Lysosomal_storage_disease_t2,Overgrowth_Panel,Skeletal_Dysplasia_Panel
NPR3	Overgrowth_Panel
NPRL2	Focal_epilepsy
NPRL3	Epilepsy_t2,Focal_epilepsy
NR0B1	Disorders_of_sexual_development_t1,Hypogonadotropic_hypogonadism_t1,Disorders_of_sex_development
NR1H4	Cholestasis_t1,Neonatal_cholestasis
NR2E1	Hereditary_Microcephaly_t2

Supplementary Table 2. List of pathogenic or likely-pathogenic germline variants

Gene	Associated diseases
NR2E3	Hereditary_retinopathy_t2,Retinitis_pigmentosa_t1,Retinitis_pigmentosa,Vitreoretinopathy,Retinitis_Pigmentosa_and_Related,Vitreoretinopathy_and_Related,retina
NR2F1	Epilepsy_t2,Hereditary_retinopathy_t2,Hereditary_Autism_t2,Optic_neuropathy_and_Related
NR3C2	Acid-base-electro_gene
NR5A1	Hypogonadotropic_hypogonadism_t2,Disorders_of_sexual_development_t1,Disorders_of_sex_development
NRAP	Cardiomyopathy_t2
NRAS	Cardiomyopathy_t2,Skeletal_dysplasia_t2,Proportionate_short_stature_t2,Rasopathies_t1,Cell-free_cancer,Hematologic_malignancy,Noonan_syndrome,Skeletal_Dysplasia_Panel,ALL_and_others_t1,ALL_and_others_t2,AML,Bone_Marrow_Failure_Germline_Predisposition,Circulating_tumor_DNA_assay_55,Germline_Cancer,Lymphoma_B_cell_t1,Lymphoma_B_cell_t2,Lymphoma_T_NK_cell_t1,Lymphoma_T_NK_cell_t2,MDS_MPN,Myeloma_t1,Noonan_Syndrome_and_Related
NRG1	Hirschsprung's_disease
NRG2	Charcot_Marie_Tooth_and_Related
NRL	Hereditary_retinopathy_t2,Retinitis_pigmentosa_t1,Retinitis_pigmentosa,Retinitis_Pigmentosa_and_Related,retina
NRTN	Hirschsprung's_disease
NRXN1	Epilepsy_t2,Hereditary_Autism_t2
NSD1	Skeletal_dysplasia_t2,Epilepsy_t2,Hereditary_Autism_t1,Overgrowth_intellectual_disability,Overgrowth_Panel
NSD2	ALL_and_others_t2
NSDHL	Dermatology_t2,Skeletal_dysplasia_t2,Hereditary_Autism_t2,Skeletal_Dysplasia_Panel
NSF	Androgenetic_alopecia
NSMF	Hypogonadotropic_hypogonadism_t1
NSUN2	Rasopathies_t2,Hereditary_Autism_t2
NT5C2	Hereditary_spastic_paraplegia_t2,Epilepsy_t2,Neurodegenerative_disease_t2
NT5C3A	Anemia_t1,Hemolytic_anemia,Anemia
NTF4	Hereditary_retinopathy_t2
NTHL1	Colorectal_Cancer_Polyposis_Panel
NTNG1	Epilepsy_t2,Hereditary_Autism_t2
NTRK1	Charcot-Marie-Tooth_disease_t2,Cell-free_cancer,ALL_and_others_t2,Charcot_Marie_Tooth_and_Related,Circulating_tumor_DNA_assay_55
NTRK2	Cell-free_cancer
NTRK3	Cell-free_cancer,ALL_and_others_t2,Circulating_tumor_DNA_assay_55,Lymphoma_B_cell_t2
NUBPL	Ataxia_t2,Epilepsy_t2
NUP107	Glomerulopathy
NUP155	Arrhythmia_t2
NUS1	Epilepsy_t2
NXF5	Hereditary_Autism_t2
NXN	Skeletal_Dysplasia_Panel
NYX	Hereditary_retinopathy_t2,Congenital_Stationary_Night_Blindness_and_Related,Retinitis_Pigmentosa_and_Related,retina
OAT	Retinitis_pigmentosa_t2,Hereditary_retinopathy_t2,Urea_cycle_disorders,Retinitis_Pigmentosa_and_Related,retina
OBSCN	Hypertrophic_cardiomyopathy,Sudden_Cardiac_Arrest_and_Related
OBSL1	Skeletal_dysplasia_t2,Proportionate_short_stature_t1
OCA2	Dermatology_t2,Hereditary_retinopathy_t2,Albinism_and_Related,Optic_neuropathy_and_Related
OCRL	Skeletal_dysplasia_t2,Hereditary_retinopathy_t2,Hereditary_Autism_t2,Ca-Pi-Mg,Renal_Tubular_Disorders
ODAD1	Hereditary_Primary_Ciliary_Dyskinesia_t1
ODAD2	Hereditary_Primary_Ciliary_Dyskinesia_t1
ODAD3	Hereditary_Primary_Ciliary_Dyskinesia_t1

Supplementary Table 2. List of pathogenic or likely-pathogenic germline variants

Gene	Associated diseases
ODAD4	Hereditary_Primary_Ciliary_Dyskinesia_t2
OFD1	Ataxia_t2,Epilepsy_t2,Hereditary_retinopathy_t2,Hereditary_Primary_Ciliary_Dyskinesia_t2,Hereditary_Autism_t2,Retinitis_pigmentosa_t1,Nephronophthisis_t1,Polycystic_Kidney_Disease_t1,Retinitis_pigmentosa,Nephronophthisis_and_Related,Polycystic_kidney_disease_and_Related,Retinitis_Pigmentosa_and_Related,retina
OGT	Hereditary_Autism_t2
OPA1	Myopathy_t2,Ataxia_t2,Hearing_loss_t2,Hereditary_retinopathy_t2,Dominant_optic_atrophy,Charcot_Marie_Tooth_and_Related,Optic_neuropathy_and_Related,Retinitis_Pigmentosa_and_Related,retina
OPA3	Myopathy_t2,Ataxia_t2,Hereditary_spastic_paraplegia_t2,Inborn_error_of_metabolism_t2,Hereditary_retinopathy_t2,Neurodegenerative_disease_t2,Dominant_optic_atrophy,Optic_neuropathy_and_Related,Retinitis_Pigmentosa_and_Related,retina
OPHN1	Ataxia_t2,Epilepsy_t2,Hereditary_Microcephaly_t2,Hereditary_Autism_t2
OPN1LW	Hereditary_retinopathy_t2,retina
OPN1MW	Hereditary_retinopathy_t2,retina
OPTN	Hereditary_retinopathy_t2,Neurodegenerative_disease_t2,Amyotrophic_lateral_sclerosis_t1,Amyotrophic_lateral_sclerosis
ORAI1	Primary_immune_deficiency_t2,Myofibrillar_myopathy,Autoimmunity_and_autoinflammatory_disorders,Severe_Combined_Immunodeficiency_and_CID
ORC1	Skeletal_dysplasia_t2,Hereditary_Autism_t2,Proportionate_short_stature_t1
ORC4	Skeletal_dysplasia_t2,Proportionate_short_stature_t1
ORC6	Skeletal_dysplasia_t2,Proportionate_short_stature_t1
OSBPL2	Hearing_loss_t2,Hereditary_hearing_loss
OSGEP	Skeletal_dysplasia_t2,Proportionate_short_stature_t2
OSMR	Dermatology_t2
OSTM1	Skeletal_dysplasia_t2,Sclerosing_Bone_Disease_Panel
OTC	Inborn_error_of_metabolism_t2,Hereditary_Stroke_t2,Hereditary_Autism_t2,Urea_cycle_disorders,Inborn_Error_of_Metabolism
OTOA	Hearing_loss_t2,Hereditary_hearing_loss,Hearing_Loss_and_Related_t2
OTOF	Hearing_loss_t1,Hereditary_hearing_loss,Hearing_Loss_and_Related_t1,Hearing_Loss_and_Related_t2
OTOG	Hearing_loss_t2,Hereditary_hearing_loss
OTOGL	Hearing_loss_t2,Hereditary_hearing_loss,Hearing_Loss_and_Related_t2
OTUD4	Hypogonadotropic_hypogonadism_t2
OTULIN	Inflammatory_Bowel_t2
OTX2	Skeletal_dysplasia_t2,Proportionate_short_stature_t2,Hereditary_retinopathy_t2,Leber's_Congenital_Amaurosis_and_Related,Retinitis_Pigmentosa_and_Related,retina
OVOL2	Hereditary_retinopathy_t2,corneal_dystrophy
P2RX1	Bleeding_Platelet_Disorder
P2RX2	Hearing_loss_t2,Hereditary_hearing_loss
P2RY12	Coagulation_t2,Bleeding_Platelet_Disorder
P3H1	Dermatology_t2,Connective_tissue_disorder_t2,Skeletal_dysplasia_t1,Osteogenesis_imperfecta,Skeletal_Dysplasia_and_Related
P3H2	Hereditary_retinopathy_t2,Vitreoretinopathy,Vitreoretinopathy_and_Related,retina
P4HB	Skeletal_dysplasia_t2,Osteogenesis_imperfecta
P4HTM	Epilepsy_t2
PABPN1	Muscular_dystrophy_t2,Myopathy_t2,Myofibrillar_myopathy
PACS1	Epilepsy_t2,Hereditary_Autism_t2
PAFAH1B1	Epilepsy_t2,Hereditary_Autism_t2,Hereditary_Microcephaly_t1,Malformation_of_cortical_development
PAH	Hereditary_spastic_paraplegia_t2,Hereditary_Autism_t2,Neurodegenerative_disease_t2,Inborn_error_of_metabolism_t1,Inborn_Error_of_Metabolism
PAK3	Epilepsy_t2,Hereditary_Autism_t2
PALB2	Dermatology_t2,Skeletal_dysplasia_t2,Coagulation_t2,Hereditary_cancer_syndrome_Plus_t1,Congenital_hematologic_disease,Fanconi_anemia,Hereditary_breast_cancer,Hereditary_pancancer,Bone_Marrow_Failure_Germline_Predisposition,Breast_and_Ovarian_Cancer_Panel,Germline_Cancer

Supplementary Table 2. List of pathogenic or likely-pathogenic germline variants

Gene	Associated diseases
PALLD	Hereditary_pancancer
PAM16	Skeletal_Dysplasia_Panel
PANK2	Ataxia_t2,Epilepsy_t2,Hereditary_retinopathy_t2,Dystonia_t2,Parkinson's_disease_t1,Neurodegenerative_disease_t1,Chorea,Dystonia,Neurodegeneration_with_brain_iron_accumulation,Parkinson_Disease_and_Related,retina
PAPSS2	Skeletal_dysplasia_t2,Skeletal_Dysplasia_Panel,Spondyloepiphyseal_metaphyseal_dysplasia
PARK7	Alzheimer's_disease_t2,Dystonia_t2,Dementia_t2,Parkinson's_disease_t1,Neurodegenerative_disease_t1,Parkinson,Parkinson_Disease_and_Related
PARN	Dermatology_t2,Congenital_hematologic_disease,Dyskeratosis_congenita
PARS2	Epilepsy_t2
PAX1	Androgenetic_alopecia
PAX2	Hereditary_retinopathy_t2,Polycystic_Kidney_Disease_t1,Glomerulopathy,Polycystic_kidney_disease_and_Related,retina
PAX3	Dermatology_t2,Hereditary_retinopathy_t2,Hearing_loss_t1,Hereditary_hearing_loss,Albinism_and_Related,Hearing_Loss_and_Related_t1,Hearing_Loss_and_Related_t2
PAX4	Polycystic_Kidney_Disease_t2,Maturity-Onset_Diabetes_of_the_Young,_MODY_t1,Monogenic_diabetes_panel
PAX5	Congenital_hematologic_disease,Leukemia_predisposition,ALL_and_others_t2,Hereditary_Myeloid_Leukemia_Panel,Lymphoma_B_cell_t2,Lymphoma_T_NK_cell_t2
PAX6	Ataxia_t2,Hereditary_retinopathy_t2,Hereditary_Autism_t2,Maturity-Onset_Diabetes_of_the_Young,_MODY_t1,Nystagmus_and_Related,Optic_neuropathy_and_Related,glaucoma
PAX8	Hypothyroidism_t1,Congenital_Hypothyroidism
PAX9	Dermatology_t2,Ectodermal_dysplasia_Hypodontia
PC	Epilepsy_t2
PCARE	Hereditary_retinopathy_t2,Retinitis_pigmentosa_t1,Retinitis_pigmentosa,Retinitis_Pigmentosa_and_Related,retina
PCBD1	Inborn_error_of_metabolism_t2,Atypical_Hemolytic_Uremic_Syndrome_t2,Inborn_Error_of_Metabolism
PCCA	Cardiomyopathy_t2,Inborn_error_of_metabolism_t1,Inborn_Error_of_Metabolism
PCCB	Cardiomyopathy_t2,Inborn_error_of_metabolism_t1,Inborn_Error_of_Metabolism
PCDH12	Hereditary_Microcephaly_t2
PCDH15	Retinitis_pigmentosa_t2,Hearing_loss_t2,Hereditary_retinopathy_t1,Hereditary_hearing_loss,Hearing_Loss_and_Related_t1,Hearing_Loss_and_Related_t2,Retinitis_Pigmentosa_and_Related,retina
PCDH19	Arrhythmia_t2,Epilepsy_t1,Hereditary_Autism_t1,Early_onset_epilepsy,Epilepsy_and_Related
PCDH9	Hereditary_Autism_t2
PCGF2	Hereditary_Autism_t2
PCLO	Hereditary_Microcephaly_t2
PCNT	Skeletal_dysplasia_t2,Epilepsy_t2,Hereditary_Autism_t2,Proportionate_short_stature_t1,Hereditary_Microcephaly_t1
PCSK1	Disorders_of_sexual_development_t2,Hypogonadotropic_hypogonadism_t2,Congenital_diarrhea
PCSK9	Hereditary_Stroke_t2,Hereditary_Dyslipidemia_t1,Dyslipidemia_and_Related
PCYT1A	Skeletal_dysplasia_t2,Hereditary_retinopathy_t2,Skeletal_Dysplasia_Panel,Spondyloepiphyseal_metaphyseal_dysplasia,Cone-Rod_Dystrophy_and_Related,retina
PDCD10	Cerebral_cavernous_malformations,Primary_Pulmonary_Hypertension_and_Related_Disordisorders
PDCD1LG2	Cell-free_cancer,Lymphoma_T_NK_cell_t2
PDE10A	Dystonia_t2,Hereditary_Autism_t2,Chorea
PDE1C	Hearing_loss_t2,Hereditary_hearing_loss
PDE3A	Skeletal_dysplasia_t2
PDE4D	Skeletal_dysplasia_t2,Hereditary_Autism_t2,Skeletal_Dysplasia_Panel
PDE6A	Hereditary_retinopathy_t2,Retinitis_pigmentosa_t1,Retinitis_pigmentosa,Retinitis_Pigmentosa_and_Related,retina
PDE6B	Hereditary_retinopathy_t2,Retinitis_pigmentosa_t1,Retinitis_pigmentosa,Congenital_Stationary_Night_Blindness_and_Related,Retinitis_Pigmentosa_and_Related,retina
PDE6C	Hereditary_retinopathy_t2,Achromatopsia_and_Related,Cone-Rod_Dystrophy_and_Related,Retinitis_Pigmentosa_and_Related,retina
PDE6D	Hereditary_retinopathy_t2
PDE6G	Hereditary_retinopathy_t2,Retinitis_pigmentosa_t1,Retinitis_pigmentosa,Retinitis_Pigmentosa_and_Related,retina

Supplementary Table 2. List of pathogenic or likely-pathogenic germline variants

Gene	Associated diseases
PDE6H	Hereditary_retinopathy_t2,Achromatopsia_and_Related,Cone-Rod_Dystrophy_and_Related,Retinitis_Pigmentosa_and_Related,retina
PDGFB	Dystonia_t2,Parkinson's_disease_t2,Chorea,Fahr's_disease
PDGFRA	Cell-free_cancer,Hematologic_malignancy,Hereditary_pancancer,ALL_and_others_t2,Circulating_tumor_DNA_assay_55
PDGFRB	Dystonia_t2,Parkinson's_disease_t2,Cell-free_cancer,Chorea,Fahr's_disease,ALL_and_others_t2
PDHA1	Epilepsy_t2,Hereditary_Autism_t2,Anemia,Leigh_Syndrome_and_Related,Optic_neuropathy_and_Related
PDHB	Leigh_Syndrome_and_Related
PDHX	Hereditary_Autism_t2,Anemia,Leigh_Syndrome_and_Related,Optic_neuropathy_and_Related
PDK3	Charcot-Marie-Tooth_disease_t1,Charcot-Marie-Tooth,Charcot_Marie_Tooth_and_Related
PDLIM3	Arrhythmia_t2,Cardiomyopathy_t2,Hypertrophic_cardiomyopathy
PDSS1	Ataxia_t2,Hereditary_Autism_t2
PDSS2	Ataxia_t2,Epilepsy_t2,Leigh_Syndrome_and_Related
PDX1	Polycystic_Kidney_Disease_t2,Maturity-Onset_Diabetes_of_the_Young,_MODY_t1,Monogenic_diabetes_panel
PDYN	Ataxia_t2
PDZD7	Hearing_loss_t2,Hereditary_retinopathy_t2,Hereditary_hearing_loss,Hearing_Loss_and_Related_t2,retina
PET100	Leigh_Syndrome_and_Related,Optic_neuropathy_and_Related
PEX1	Epilepsy_t2,Retinitis_pigmentosa_t2,Hearing_loss_t2,Hereditary_retinopathy_t2,Lysosomal_storage_disease_t1,Cholestasis_t1,Charcot_Marie_Tooth_and_Related,Retinitis_Pigmentosa_and_Related,retina,glaucoma
PEX10	Ataxia_t2,Hereditary_retinopathy_t2,Lysosomal_storage_disease_t1,Cholestasis_t1
PEX11B	Hereditary_retinopathy_t2
PEX12	Lysosomal_storage_disease_t2,Epilepsy_t2,Hereditary_retinopathy_t2,Cholestasis_t1
PEX13	Lysosomal_storage_disease_t2,Hereditary_retinopathy_t2
PEX14	Skeletal_dysplasia_t2,Lysosomal_storage_disease_t2,Epilepsy_t2,Hereditary_retinopathy_t2
PEX16	Lysosomal_storage_disease_t2,Hereditary_retinopathy_t2
PEX19	Skeletal_dysplasia_t2,Lysosomal_storage_disease_t2,Hereditary_retinopathy_t2
PEX2	Lysosomal_storage_disease_t2,Epilepsy_t2,Retinitis_pigmentosa_t2,Hereditary_retinopathy_t2,Cholestasis_t1,Retinitis_Pigmentosa_and_Related,retina
PEX26	Lysosomal_storage_disease_t2,Epilepsy_t2,Retinitis_pigmentosa_t2,Hearing_loss_t2,Hereditary_retinopathy_t2,Cholestasis_t1,Retinitis_Pigmentosa_and_Related
PEX3	Lysosomal_storage_disease_t2,Epilepsy_t2,Hereditary_retinopathy_t2
PEX5	Lysosomal_storage_disease_t2,Epilepsy_t2,Hereditary_retinopathy_t2,Cholestasis_t1,Skeletal_Dysplasia_Panel
PEX6	Lysosomal_storage_disease_t2,Epilepsy_t2,Hearing_loss_t2,Hereditary_retinopathy_t2,Cholestasis_t1
PEX7	Dermatology_t2,Epilepsy_t2,Retinitis_pigmentosa_t2,Hereditary_retinopathy_t2,Hereditary_Autism_t2,Skeletal_dysplasia_t1,Ataxia_t1,Ataxia,Skeletal_Dysplasia_Panel,Spondyloepiphyseal_metaphyseal_dysplasia,Ataxia_and_Related,Charcot_Marie_Tooth_and_Related,Retinitis_Pigmentosa_and_Related,Skeletal_Dysplasia_and_Related,retina
PFKM	Lysosomal_storage_disease_t2,Anemia_t2,Myopathy_t1,Hemolytic_anemia,Metabolic_myopathy,Anemia,Myopathy_and_Related
PFN1	Neurodegenerative_disease_t2,Amyotrophic_lateral_sclerosis_t2,Amyotrophic_lateral_sclerosis
PGAM2	Myopathy_t2,Metabolic_myopathy
PGAP1	Hereditary_spastic_paraplegia_t2,Neurodegenerative_disease_t2
PGD	Hemolytic_anemia
PGK1	Myopathy_t2,Lysosomal_storage_disease_t2,Epilepsy_t2,Hereditary_retinopathy_t2,Hereditary_Autism_t2,Anemia_t1,Hemolytic_anemia,Metabolic_myopathy,Anemia,retina
PGM1	Myopathy_t2,Metabolic_myopathy
PGM3	Skeletal_dysplasia_t2,Autoimmunity_and_autoinflammatory_disorders,Severe_Combined_Immunodeficiency_and_CID
PHACTR1	Epilepsy_t2
PHC1	Hereditary_Microcephaly_t2
PHEX	Skeletal_dysplasia_t1,Rickets_Hypoparathyroidism_panel,Skeletal_Dysplasia_Panel,Renal_Tubular_Disorders,Skeletal_Dysplasia_and_Related

Supplementary Table 2. List of pathogenic or likely-pathogenic germline variants

Gene	Associated diseases
PHF6	Skeletal_dysplasia_t2,Epilepsy_t2,Hereditary_Autism_t2,Hematologic_malignancy,ALL_and_others_t1,ALL_and_others_t2,AML,MDS_MPN
PHF8	Hereditary_Autism_t2
PHGDH	Hereditary_Microcephaly_t2
PHKA1	Myopathy_t2,Metabolic_myopathy
PHKA2	Hereditary_Autism_t2,Lysosomal_storage_disease_t1,Glycogen_storage_disease,Lysosomal_Storage_Disease
PHKB	Lysosomal_storage_disease_t2,Glycogen_storage_disease,Lysosomal_Storage_Disease
PHKG1	Metabolic_myopathy
PHKG2	Lysosomal_storage_disease_t2,Hereditary_Autism_t2,Glycogen_storage_disease,Lysosomal_Storage_Disease
PHOX2B	Hereditary_pancancer,Hirschsprung's_disease
PHYH	Dermatology_t2,Ataxia_t2,Lysosomal_storage_disease_t2,Retinitis_pigmentosa_t2,Hereditary_retinopathy_t2,Charcot-Marie-Tooth_disease_t1,Ataxia,Charcot_Marie_Tooth_and_Related,Retinitis_Pigmentosa_and_Related,retina
PIEZO1	Anemia_t1,Hemolytic_anemia,Anemia
PIGA	Epilepsy_t2,Atypical_Hemolytic_Uremic_Syndrome_t2,Hereditary_Autism_t2,Hemolytic_anemia,AML,MDS_MPN
PIGB	Epilepsy_t2
PIGC	Epilepsy_t2
PIGG	Epilepsy_t2
PIGL	Hereditary_retinopathy_t2,Hereditary_Autism_t2
PIGN	Epilepsy_t2
PIGO	Epilepsy_t2,Hereditary_Autism_t2
PIGP	Epilepsy_t2
PIGQ	Epilepsy_t2
PIGS	Epilepsy_t2
PIGT	Epilepsy_t2
PIGV	Skeletal_dysplasia_t2,Epilepsy_t2,Hereditary_Autism_t2
PIGW	Epilepsy_t2
PIK3AP1	Epilepsy_t2
PIK3CA	Skeletal_dysplasia_t2,Cell-free_cancer,Focal_epilepsy,Malformation_of_cortical_development,Overgrowth_intellectual_disability,Overgrowth_Panel,ALL_and_others_t2,Circulating_tumor_DNA_assay_55,Lymphoma_T_NK_cell_t1,Lymphoma_T_NK_cell_t2,Myeloma_t1
PIK3CD	Primary_immune_deficiency_t1,Inflammatory_Bowel_t1,Primary_immunodeficiency,Phagocyte_Defect_and_Infectious_disease,Autoimmunity_and_autoinflammatory_disorders,B_cell_and_Humoral_Immune_Deficiency,Hereditary_Myeloid_Leukemia_Panel,Lymphoma_T_NK_cell_t2
PIK3CG	Lymphoma_T_NK_cell_t2
PIK3R1	Inflammatory_Bowel_t2,Primary_immune_deficiency_t1,Cell-free_cancer,Primary_immunodeficiency,Very-early-onset_inflammatory_bowel_disease,Phagocyte_Defect_and_Infectious_disease,B_cell_and_Humoral_Immune_Deficiency,Lymphoma_T_NK_cell_t1,Lymphoma_T_NK_cell_t2,Myeloma_t1,glaucoma
PIK3R2	Lymphoma_T_NK_cell_t2
PIKFYVE	Hereditary_retinopathy_t2,Corneal_Dystrophy_and_Related,corneal_dystrophy
PIM1	Lymphoma_t2,Lymphoma_T_NK_cell_t2
PINK1	Alzheimer's_disease_t2,Dystonia_t2,Dementia_t2,Parkinson's_disease_t1,Neurodegenerative_disease_t1,Parkinson,Parkinson_Disease_and_Related
PIP5K1B	Hereditary_Autism_t2
PISD	Skeletal_dysplasia_t2,Proportionate_short_stature_t2,Hearing_loss_t2,Hereditary_retinopathy_t2,Skeletal_Dysplasia_Panel
PITPNM3	Retinitis_pigmentosa_t2,Hereditary_retinopathy_t2,Cone-Rod_Dystrophy_and_Related,Retinitis_Pigmentosa_and_Related,retina
PITRM1	Epilepsy_t2

Supplementary Table 2. List of pathogenic or likely-pathogenic germline variants

Gene	Associated diseases
PITX1	Skeletal_dysplasia_t2
PITX2	Skeletal_dysplasia_t2,Proportionate_short_stature_t2,Hereditary_retinopathy_t2,corneal_dystrophy,glaucoma
PITX3	Hereditary_retinopathy_t2,glaucoma
PJVK	Hearing_loss_t2,Hereditary_hearing_loss,Hearing_Loss_and_Related_t2
PKD1	Polycystic_Kidney_Disease_t1,Polycystic_kidney_disease_and_Related
PKD2	Polycystic_Kidney_Disease_t1,Polycystic_kidney_disease_and_Related
PKHD1	Nephronophthisis_t1,Polycystic_Kidney_Disease_t1,Neonatal_cholestasis,Nephronophthisis_and_Related,Polycystic_kidney_disease_and_Related
PKLR	Anemia_t1,Hemolytic_anemia,Anemia,Erythrocytosis
PKP1	Dermatology_t1
PKP2	Arrhythmia_t1,Cardiomyopathy_t1,Arrhythmia,Arrhythmia_and_Related,Cardiomyopathy_and_Related,Sudden_Cardiac_Arrest_and_Related
PLA2G4A	Coagulation_t2
PLA2G5	Retinitis_pigmentosa_t2,Hereditary_retinopathy_t2,Macular_dystrophy_and_Related,Retinitis_Pigmentosa_and_Related,retina
PLA2G6	Epilepsy_t2,Alzheimer's_disease_t2,Dystonia_t2,Dementia_t2,Hereditary_Autism_t2,Parkinson's_disease_t1,Neurodegenerative_disease_t1,Ataxia,Chorea,Dystonia,Neurodegeneration_with_brain_iron_accumulation,Parkinson,Charcot_Marie_Tooth_and_Related,Parkinson_Disease_and_Related
PLAA	Epilepsy_t2
PLAT	Coagulation_t2,Atypical_Hemolytic_Uremic_Syndrome,Bleeding_Coagulopathy,Thrombosis
PLAU	Coagulation_t2,Bleeding_Platelet_Disorder
PLAUR	Atypical_Hemolytic_Uremic_Syndrome,Bleeding_Coagulopathy,Thrombosis
PLB1	Dilated_cardiomyopathy
PLCB1	Epilepsy_t2
PLCB3	Skeletal_Dysplasia_Panel
PLCE1	Glomerulopathy
PLCG1	Lymphoma_t1,Lymphoma_T_NK_cell_t1,Lymphoma_T_NK_cell_t2
PLCG2	Primary_immune_deficiency_t2,Inflammatory_Bowel_t2,Lymphoma_t1,Very-early-onset_inflammatory_bowel_disease,Autoimmunity_and_autoinflammatory_disorders,B_cell_and_Humoral_Immune_Deficiency
PLEC	Cardiomyopathy_t2,Myopathy_t2,Dermatology_t1,Muscular_dystrophy_t1,Limb_girdle_muscular_dystrophy,Myofibrillar_myopathy,Muscular_Dystrophy_and_Related
PLEKHG1	Stroke
PLEKHG2	Hereditary_Microcephaly_t2
PLEKHG5	Charcot-Marie-Tooth_disease_t2,Charcot-Marie-Tooth,Charcot_Marie_Tooth_and_Related
PLEKHM1	Sclerosing_Bone_Disease_Panel
PLEKHM2	Cardiomyopathy_t2
PLG	Coagulation_t2,Atypical_Hemolytic_Uremic_Syndrome_t1,DNAJC21_EFL1_SBDS_SRP54,Atypical_Hemolytic_Uremic_Syndrome,Bleeding_Coagulopathy,Thrombosis
PLK4	Hereditary_retinopathy_t2,Hereditary_Microcephaly_t2,retina
PLN	Arrhythmia_t2,Cardiomyopathy_t2,Dilated_cardiomyopathy,Hypertrophic_cardiomyopathy,Arrhythmia_and_Related
PLOD1	Dermatology_t2,Connective_tissue_disorder_t1,Skeletal_Dysplasia_Panel,Connective_Tissue_Disorder_and_Related,glaucoma
PLOD2	Skeletal_dysplasia_t2,Osteogenesis_imperfecta
PLP1	Ataxia_t2,Epilepsy_t2,Hereditary_Autism_t2,Neurodegenerative_disease_t2,Charcot-Marie-Tooth_disease_t1,Hereditary_spastic_paraplegia_t1,Hereditary_spastic_paraplegia_panel,Charcot_Marie_Tooth_and_Related,Optic_neuropathy_and_Related,Spastic_Paraplegia_and_Related
PLPBP	Epilepsy_t2
PLS3	Skeletal_dysplasia_t2,Osteogenesis_imperfecta
PMM2	Ataxia_t2,Epilepsy_t2,Hereditary_retinopathy_t2,Hereditary_Autism_t2,Polycystic_Kidney_Disease_t1,Charcot_Marie_Tooth_and_Related,Polycystic_kidney_disease_and_Related
PMP2	Charcot-Marie-Tooth,Charcot_Marie_Tooth_and_Related

Supplementary Table 2. List of pathogenic or likely-pathogenic germline variants

Gene	Associated diseases
PMP22	Ataxia_t2,Hearing_loss_t2,Charcot-Marie-Tooth_disease_t1,Charcot-Marie-Tooth,Charcot_Marie_Tooth_and_Related
PMS1	Germline_Cancer
PMS2	Dermatology_t2,Primary_immune_deficiency_t2,Hereditary_cancer_syndrome_Plus_t1,Cell-free_cancer,Congenital_hematologic_disease,Hereditary_colon_cancer,Hereditary_pancancer,Leukemia_predisposition,Breast_and_Ovarian_Cancer_Panel,Colorectal_Cancer_Polyposis_Panel,Germline_Cancer
PNKD	Ataxia_t2,Epilepsy_t2,Parkinson's_disease_t2,Neurodegenerative_disease_t2,Dystonia_t1,Dyskinesia-dystonia-paralysis,Dystonia,Paroxysmal_movement_disorder,Dystonia_and_Related
PNKP	Epilepsy_t2,Dystonia_t2,Parkinson's_disease_t2,Hereditary_Autism_t2,Ataxia_t1,Hereditary_Microcephaly_t1,Ataxia,Chorea,Ataxia_and_Related,Charcot_Marie_Tooth_and_Related
PNP	Primary_immune_deficiency_t2,Familial_hemophagocytic_lymphohistiocytosis,Hemophagocytic_Lymphohistiocytosis_and_Related,Severe_Combined_Immunodeficiency_and_CID
PNPLA1	Dermatology_t1
PNPLA2	Cardiomyopathy_t2,Muscular_dystrophy_t2,Myopathy_t2
PNPLA4	Bleeding_Platelet_Disorder
PNPLA6	Hereditary_spastic_paraplegia_t2,Hereditary_retinopathy_t2,Hypogonadotropic_hypogonadism_t2,Neurodegenerative_disease_t2,Ataxia_t1,Ataxia,Ataxia_and_Related,retina
PNPO	Epilepsy_t1,Early_onset_epilepsy,Epilepsy_and_Related
PNPT1	Hearing_loss_t2,Hereditary_hearing_loss
POC1A	Skeletal_dysplasia_t2,Proportionate_short_stature_t2
POC1B	Hereditary_retinopathy_t2,Macular_dystrophy,Cone-Rod_Dystrophy_and_Related,retina
POC5	retina
POFUT1	Dermatology_t2
POGLUT1	Dermatology_t2,Muscular_dystrophy_t2,Myopathy_t2
POGZ	Hereditary_Autism_t2,Autism_and_Related
POLA1	Skeletal_dysplasia_t2
POLD1	Hereditary_cancer_syndrome_Plus_t1,Hereditary_colon_cancer,Hereditary_hearing_loss,Hereditary_pancancer,Colorectal_Cancer_Polyposis_Panel
POLE	Hereditary_cancer_syndrome_Plus_t1,Hereditary_colon_cancer,Hereditary_pancancer,Colorectal_Cancer_Polyposis_Panel
POLE2	Primary_immune_deficiency_t2,Autoimmunity_and_autoinflammatory_disorders,Colorectal_Cancer_Polyposis_Panel,Severe_Combined_Immunodeficiency_and_CID
POLG	Muscular_dystrophy_t2,Myopathy_t2,Charcot-Marie-Tooth_disease_t2,Hereditary_retinopathy_t2,Alzheimer's_disease_t2,Dystonia_t2,Dementia_t2,Parkinson's_disease_t2,Hereditary_Stroke_t2,Neurodegenerative_disease_t2,Ataxia_t1,Epilepsy_t1,Ataxia,Focal_epilepsy,Neonatal_cholestasis,Ataxia_and_Related,Charcot_Marie_Tooth_and_Related,Epilepsy_and_Related,Optic_neuropathy_and_Related,Parkinson_Disease_and_Related
POLG2	Myopathy_t2,Ataxia_t2
POLH	Dermatology_t2
POLL	Skeletal_dysplasia_t2
POLR1C	Skeletal_dysplasia_t2,Hearing_loss_t2,Hereditary_hearing_loss
POLR1D	Skeletal_dysplasia_t2,Hearing_loss_t2,Hereditary_hearing_loss
POLR3A	Ataxia_t2,Epilepsy_t2,Hypogonadotropic_hypogonadism_t2,Osteogenesis_imperfecta
POLR3B	Ataxia_t2,Epilepsy_t2,Hypogonadotropic_hypogonadism_t2,Dystonia_t2,Parkinson's_disease_t2,Sclerosing_Bone_Disease_Panel
POMGNT1	Myopathy_t2,Ataxia_t2,Epilepsy_t2,Hereditary_retinopathy_t2,Hereditary_Autism_t2,Muscular_dystrophy_t1,Retinitis_pigmentosa_t1,Congenital_muscular_dystrophy,Limb_girdle_muscular_dystrophy,Retinitis_pigmentosa,Muscular_Dystrophy_and_Related,Retinitis_Pigmentosa_and_Related,retina
POMGNT2	Muscular_dystrophy_t2,Myopathy_t2,Congenital_muscular_dystrophy
POMK	Muscular_dystrophy_t2,Myopathy_t2,Congenital_muscular_dystrophy
POMP	Dermatology_t2
POMT1	Myopathy_t2,Ataxia_t2,Epilepsy_t2,Hereditary_Autism_t2,Muscular_dystrophy_t1,Hereditary_Microcephaly_t1,Congenital_muscular_dystrophy,Limb_girdle_muscular_dystrophy,Muscular_Dystrophy_and_Related,glaucoma
POMT2	Myopathy_t2,Ataxia_t2,Epilepsy_t2,Hereditary_Autism_t2,Muscular_dystrophy_t1,Congenital_muscular_dystrophy,Limb_girdle_muscular_dystrophy,Muscular_Dystrophy_and_Related

Supplementary Table 2. List of pathogenic or likely-pathogenic germline variants

Gene	Associated diseases
PON1	Stroke
PON3	Hereditary_Autism_t2
POP1	Skeletal_dysplasia_t2,Proportionate_short_stature_t2,Skeletal_Dysplasia_Panel
POR	Skeletal_dysplasia_t2,Disorders_of_sexual_development_t1,Craniosynostosis,Disorders_of_sex_development
PORCN	Dermatology_t2,Hereditary_Autism_t2
POT1	Dermatology_t2,Lymphoma_t1,Congenital_hematologic_disease,Dyskeratosis_congenita,Hereditary_pancancer
POU1F1	Skeletal_dysplasia_t2,Hereditary_Autism_t2,Proportionate_short_stature_t1,Hypothyroidism_t1,Congenital_Hypothyroidism
POU3F4	Hearing_loss_t1,Hereditary_hearing_loss,Hearing_Loss_and_Related_t1,Hearing_Loss_and_Related_t2
POU4F3	Hearing_loss_t2,Hereditary_hearing_loss,Hearing_Loss_and_Related_t2
PPA2	Arrhythmia_t2,Cardiomyopathy_t2
PPARG	Maturity-Onset_Diabetes_of_the_Young,_MODY_t2
PPCS	Cardiomyopathy_t2
PPIB	Skeletal_dysplasia_t2,Osteogenesis_imperfecta
PPIP5K2	Hereditary_hearing_loss
PPM1D	Hematologic_malignancy,ALL_and_others_t1,ALL_and_others_t2,MDS_MPN
PPOX	Dermatology_t2,Hereditary_Autism_t2
PPP1CB	Cardiomyopathy_t2,Hereditary_Autism_t2,Rasopathies_t1
PPP1R15B	Hereditary_Microcephaly_t2
PPP2CA	Epilepsy_t2
PPP2R1A	Hereditary_Autism_t2,Cell-free_cancer
PPP2R5D	Epilepsy_t2,Hereditary_Autism_t2,Overgrowth_intellectual_disability
PPP3CA	Skeletal_dysplasia_t2,Proportionate_short_stature_t2,Epilepsy_t2,Lymphoma_B_cell_t2,Lymphoma_T_NK_cell_t2,corneal_keratoconus
PPP3CB	Lymphoma_B_cell_t2,Lymphoma_T_NK_cell_t2
PPP3CC	Lymphoma_B_cell_t2,Lymphoma_T_NK_cell_t2
PPP3R1	Lymphoma_B_cell_t2,Lymphoma_T_NK_cell_t2
PPP3R2	Lymphoma_B_cell_t2,Lymphoma_T_NK_cell_t2
PPT1	Lysosomal_storage_disease_t2,Epilepsy_t2,Hereditary_Autism_t2,Hereditary_retinopathy_t1,Epilepsy_and_Related
PQBP1	Epilepsy_t2,Hereditary_Microcephaly_t2,Hereditary_Autism_t1
PRCD	Hereditary_retinopathy_t2,Retinitis_pigmentosa_t1,Retinitis_pigmentosa,Retinitis_Pigmentosa_and_Related,retina
PRDM1	Lymphoma_t1,Lymphoma_B_cell_t1,Lymphoma_B_cell_t2,Lymphoma_T_NK_cell_t1,Lymphoma_T_NK_cell_t2
PRDM12	Charcot-Marie-Tooth_disease_t2,Charcot_Marie_Tooth_and_Related
PRDM13	Hereditary_retinopathy_t2,Macular_dystrophy_and_Related
PRDM16	Cardiomyopathy_t2
PRDM5	Dermatology_t2,Connective_tissue_disorder_t2,Hereditary_retinopathy_t2,Keratoconus_and_Related,corneal_keratoconus
PRDM8	Epilepsy_t2
PRF1	Coagulation_t2,Neurodegenerative_disease_t2,Amyotrophic_lateral_sclerosis_t2,Primary_immune_deficiency_t1,Familial_hemophagocytic_lymphohistiocytosis,Hemophagocytic_Lymphohistiocytosis_and_Related
PRICKLE1	Epilepsy_t2
PRICKLE2	Epilepsy_t2
PRIMA1	Epilepsy_t2
PRKACG	Coagulation_t2,Bleeding_Platelet_Disorder
PRKAG2	Arrhythmia_t2,Cardiomyopathy_t2,Hypertrophic_cardiomyopathy

Supplementary Table 2. List of pathogenic or likely-pathogenic germline variants

Gene	Associated diseases
PRKAR1A	Dermatology_t2,Skeletal_dysplasia_t2,Proportionate_short_stature_t2,Hereditary_Autism_t2,Hereditary_pancancer,Skeletal_Dysplasia_Panel
PRKCB	Lymphoma_T_NK_cell_t1,Lymphoma_T_NK_cell_t2
PRKCD	Autoimmunity_and_autoinflammatory_disorders,B_cell_and_Humoral_Immune_Deficiency,Hemophagocytic_Lymphohistiocytosis_and_Related
PRKCG	Retinitis_pigmentosa_t2,Hereditary_retinopathy_t2,Ataxia_t1,Ataxia_and_Related,Retinitis_Pigmentosa_and_Related
PRKCSH	Polycystic_Kidney_Disease_t1,Neonatal_cholestasis,Polycystic_kidney_disease_and_Related
PRKD1	Dermatology_t2
PRKDC	Primary_immune_deficiency_t2,Severe_Combined_Immunodeficiency_and_CID
PRKG1	Connective_tissue_disorder_t2,Familial_thoracic_aortic_aneurysms_and_aortic_dissections,Connective_Tissue_Disorder_and_Related,Marfan_and_Related
PRKN	Alzheimer's_disease_t2,Dystonia_t2,Dementia_t2,Parkinson's_disease_t1,Neurodegenerative_disease_t1,Dyskinesia-dystonia-paralysis,Parkinson,Paroxysmal_movement_disorder,Parkinson_Disease_and_Related
PRKRA	Alzheimer's_disease_t2,Dementia_t2,Parkinson's_disease_t2,Neurodegenerative_disease_t2,Dystonia_t1,Dyskinesia-dystonia-paralysis,Dystonia,Dystonia_and_Related
PRMT7	Skeletal_dysplasia_t2,Proportionate_short_stature_t2,Hereditary_Autism_t2
PRNP	Alzheimer's_disease_t2,Parkinson's_disease_t2,Amyotrophic_lateral_sclerosis_t2,Dementia_t1,Neurodegenerative_disease_t1,Dementia_and_Related
PROC	Coagulation_t2,Hereditary_Stroke_t2,Stroke,Atypical_Hemolytic_Uremic_Syndrome,Bleeding_Coagulopathy,Thrombosis
PROCR	Atypical_Hemolytic_Uremic_Syndrome,Bleeding_Coagulopathy,Thrombosis
PRODH	Lysosomal_storage_disease_t2,Epilepsy_t2
PROK2	Disorders_of_sexual_development_t1,Hypogonadotropic_hypogonadism_t1,Isolated_Hypogonadotropic_Hypogonadism
PROKR2	Hereditary_retinopathy_t2,Disorders_of_sexual_development_t1,Hypogonadotropic_hypogonadism_t1,Isolated_Hypogonadotropic_Hypogonadism
PROM1	Hereditary_retinopathy_t2,Retinitis_pigmentosa_t1,Macular_dystrophy,Retinitis_pigmentosa,Cone-Rod_Dystrophy_and_Related,Macular_dystrophy_and_Related,Retinitis_Pigmentosa_and_Related,retina
PROP1	Skeletal_dysplasia_t2,Proportionate_short_stature_t1,Disorders_of_sexual_development_t1,Hypothyroidism_t1,Hypogonadotropic_hypogonadism_t1,Congenital_Hypothyroidism
PROS1	Coagulation_t2,Hereditary_Stroke_t2,Stroke,Atypical_Hemolytic_Uremic_Syndrome,Bleeding_Coagulopathy,Thrombosis
PROZ	Atypical_Hemolytic_Uremic_Syndrome,Bleeding_Coagulopathy,Thrombosis
PRPF3	Hereditary_retinopathy_t2,Retinitis_pigmentosa_t1,Retinitis_pigmentosa,Retinitis_Pigmentosa_and_Related,retina
PRPF31	Hereditary_retinopathy_t2,Retinitis_pigmentosa_t1,Retinitis_pigmentosa,Retinitis_Pigmentosa_and_Related,retina
PRPF4	Hereditary_retinopathy_t2,Retinitis_pigmentosa_t1,Retinitis_pigmentosa,Retinitis_Pigmentosa_and_Related,retina
PRPF6	Hereditary_retinopathy_t2,Retinitis_pigmentosa_t1,Retinitis_pigmentosa,Retinitis_Pigmentosa_and_Related,retina
PRPF8	Hereditary_retinopathy_t2,Retinitis_pigmentosa_t1,Retinitis_pigmentosa,ALL_and_others_t1,ALL_and_others_t2,AML,MDS_MPN,Retinitis_Pigmentosa_and_Related,retina
PRPH	Neurodegenerative_disease_t2,Amyotrophic_lateral_sclerosis_t2
PRPH2	Hereditary_retinopathy_t2,Retinitis_pigmentosa_t1,Macular_dystrophy,Retinitis_pigmentosa,Cone-Rod_Dystrophy_and_Related,Macular_dystrophy_and_Related,Retinitis_Pigmentosa_and_Related,retina
PRPS1	Ataxia_t2,Hearing_loss_t2,Hereditary_retinopathy_t2,Hereditary_Autism_t2,Charcot-Marie-Tooth_disease_t1,Charcot-Marie-Tooth,Hereditary_hearing_loss,Charcot_Marie_Tooth_and_Related,retina
PRRT2	Arrhythmia_t2,Ataxia_t2,Parkinson's_disease_t2,Neurodegenerative_disease_t2,Epilepsy_t1,Dystonia_t1,Dyskinesia-dystonia-paralysis,Dystonia,Early_onset_epilepsy,Focal_epilepsy,Paroxysmal_movement_disorder,Dystonia_and_Related,Epilepsy_and_Related
PRSS1	Hereditary_cancer_syndrome_Plus_t2,Hereditary_pancancer
PRSS12	Hereditary_Autism_t2
PRSS56	Hereditary_retinopathy_t2
PRUNE1	Optic_neuropathy_and_Related
PRX	Charcot-Marie-Tooth_disease_t2,Ataxia_t2,Charcot-Marie-Tooth,Charcot_Marie_Tooth_and_Related
PSAP	Lysosomal_storage_disease_t2,Epilepsy_t2
PSEN1	Dementia_t2,Parkinson's_disease_t2,Amyotrophic_lateral_sclerosis_t2,Alzheimer's_disease_t1,Neurodegenerative_disease_t1,Dilated_cardiomyopathy,Dementia_and_Related
PSEN2	Dementia_t2,Parkinson's_disease_t2,Amyotrophic_lateral_sclerosis_t2,Alzheimer's_disease_t1,Neurodegenerative_disease_t1,Dilated_cardiomyopathy,Dementia_and_Related

Supplementary Table 2. List of pathogenic or likely-pathogenic germline variants

Gene	Associated diseases
PSMB5	Myeloma_t1
PSMB8	Autoimmunity_and_autoinflammatory_disorders
PSMC3IP	Disorders_of_sex_development
PSTPIP1	Autoinflammatory_disorders
PTCH1	Dermatology_t2,Epilepsy_t2,Hereditary_pancancer,Germline_Cancer
PTCH2	Dermatology_t2
PTCHD1	Hereditary_Autism_t1
PTDSS1	Skeletal_dysplasia_t2,Sclerosing_Bone_Disease_Panel
PTEN	Dermatology_t2,Epilepsy_t2,Inflammatory_Bowel_t2,Hereditary_Autism_t1,Hereditary_cancer_syndrome_Plus_t1,Cell-free_cancer,Hematologic_malignancy,Hereditary_breast_cancer,Hereditary_colon_cancer,Hereditary_pancancer,Overgrowth_intellectual_disability,Primary_immunodeficiency,ALL_and_others_t2,Autoimmunity_and_autoinflammatory_disorders,B_cell_and_Humoral_Immune_Deficiency,Breast_and_Ovarian_Cancer_Panel,Circulating_tumor_DNA_assay_55,Colorectal_Cancer_Polyposis_Panel,Germline_Cancer,Lymphoma_B_cell_t2,Lymphoma_T_NK_cell_t1,Lymphoma_T_NK_cell_t2,Myeloma_t1
PTF1A	Maturity-Onset_Diabetes_of_the_Young,_MODY_t1,Monogenic_diabetes_panel
PTH	Rickets_Hypoparathyroidism_panel
PTH1R	Skeletal_dysplasia_t2,Skeletal_Dysplasia_Panel
PTHLH	Skeletal_dysplasia_t2
PTPN1	Lymphoma_B_cell_t1,Lymphoma_B_cell_t2
PTPN11	Dermatology_t2,Skeletal_dysplasia_t2,Epilepsy_t2,Cardiomyopathy_t1,Proportionate_short_stature_t1,Rasopathies_t1,Hereditary_Autism_t1,Cell-free_cancer,Congenital_hematologic_disease,Hematologic_malignancy,Leukemia_predisposition,Noonan_syndrome,ALL_and_others_t1,ALL_and_others_t2,AML,Bone_Marrow_Failure_Germline_Predisposition,Cardiomyopathy_and_Related,Germline_Cancer,Hereditary_Myeloid_Leukemia_Panel,Lymphoma_T_NK_cell_t2,MDS_MPN,Noonan_Syndrome_and_Related
PTPN23	Epilepsy_t2
PTPRC	Primary_immune_deficiency_t2,Severe_Combined_Immunodeficiency_and_CID
PTPRD	Maturity-Onset_Diabetes_of_the_Young,_MODY_t1
PTPRG	corneal_dystrophy
PTPRK	Lymphoma_T_NK_cell_t1,Lymphoma_T_NK_cell_t2
PTPRQ	Hearing_loss_t2,Hereditary_hearing_loss
PTRH2	Charcot-Marie-Tooth
PTS	Hereditary_spastic_paraplegia_t2,Inborn_error_of_metabolism_t2,Epilepsy_t2,Neurodegenerative_disease_t2,Inborn_Error_of_Metabolism
PUF60	Skeletal_dysplasia_t2,Proportionate_short_stature_t2
PUM1	Ataxia_t2,Epilepsy_t2
PURA	Epilepsy_t2,Hereditary_Autism_t2
PUS1	Anemia_t1,Anemia
PXDN	Hereditary_retinopathy_t2,Alport_Syndrome_t1,Glomerular_basement_membrane_disorders,glaucoma
PYCR1	Dermatology_t2,Skeletal_dysplasia_t2,Hereditary_Autism_t2,Connective_tissue_disorder_t1,Osteogenesis_imperfecta,Connective_Tissue_Disorder_and_Related
PYCR2	Epilepsy_t2
PYGL	Hereditary_Autism_t2,Lysosomal_storage_disease_t1,Glycogen_storage_disease,Lysosomal_Storage_Disease
PYGM	Myopathy_t1,Lysosomal_storage_disease_t1,Metabolic_myopathy,Lysosomal_Storage_Disease,Myopathy_and_Related
PYROXD1	Muscular_dystrophy_t2,Myopathy_t2
QDPR	Lysosomal_storage_disease_t2,Inborn_error_of_metabolism_t2,Epilepsy_t2,Dystonia,Parkinson,Inborn_Error_of_Metabolism
QRSL1	Cardiomyopathy_t2
RAB18	Hypogonadotropic_hypogonadism_t2,Hereditary_Microcephaly_t2
RAB23	Craniosynostosis

Supplementary Table 2. List of pathogenic or likely-pathogenic germline variants

Gene	Associated diseases
RAB27A	Dermatology_t2,Lysosomal_storage_disease_t2,Coagulation_t2,Hereditary_retinopathy_t2,Familial_hemophagocytic_lymphohistiocytosis,Albinism_and_Related,Bone_Marrow_Failure_Germline_Predisposition,Hemophagocytic_Lymphohistiocytosis_and_Related
RAB28	Hereditary_retinopathy_t2,Cone-Rod_Dystrophy_and_Related,Retinitis_Pigmentosa_and_Related,retina
RAB33B	Skeletal_dysplasia_t2,Skeletal_Dysplasia_Panel,Spondyloepiphyseal_metaphyseal_dysplasia
RAB39B	Epilepsy_t2,Parkinson's_disease_t2,Hereditary_Autism_t2,Chorea,Dystonia
RAB3GAP1	Epilepsy_t2,Hereditary_retinopathy_t2,Hypogonadotropic_hypogonadism_t2,Hereditary_Microcephaly_t2,Keratoconus_and_Related,corneal_keratoconus
RAB3GAP2	Hereditary_spastic_paraplegia_t2,Hypogonadotropic_hypogonadism_t2,Hereditary_Microcephaly_t2,Neurodegenerative_disease_t2
RAB7A	Charcot-Marie-Tooth_disease_t1,Charcot-Marie-Tooth,Charcot_Marie_Tooth_and_Related
RAC2	Primary_immune_deficiency_t2,Primary_immunodeficiency,Bone_Marrow_Failure_Germline_Predisposition,Phagocyte_Defect_and_Infectious_disease
RAD21	Skeletal_dysplasia_t2,Proportionate_short_stature_t2,Hereditary_Autism_t2,Hematologic_malignancy
RAD50	Hereditary_cancer_syndrome_Plus_t1,Hereditary_breast_cancer,Hereditary_pancancer,Breast_and_Ovarian_Cancer_Panel
RAD51	Congenital_hematologic_disease,Bone_Marrow_Failure_Germline_Predisposition,Breast_and_Ovarian_Cancer_Panel,corneal_dystrophy
RAD51B	Breast_and_Ovarian_Cancer_Panel
RAD51C	Dermatology_t2,Skeletal_dysplasia_t2,Coagulation_t2,Hereditary_cancer_syndrome_Plus_t1,Congenital_hematologic_disease,Fanconi_anemia,Hereditary_breast_cancer,Hereditary_pancancer,Bone_Marrow_Failure_Germline_Predisposition,Breast_and_Ovarian_Cancer_Panel
RAD51D	Hereditary_cancer_syndrome_Plus_t1,Hereditary_pancancer,Breast_and_Ovarian_Cancer_Panel
RAF1	Dermatology_t2,Skeletal_dysplasia_t2,Proportionate_short_stature_t2,Cardiomyopathy_t1,Rasopathies_t1,Cell-free_cancer,Noonan_syndrome,ALL_and_others_t2,Bone_Marrow_Failure_Germline_Predisposition,Cardiomyopathy_and_Related,Circulating_tumor_DNA_assay_55,Lymphoma_B_cell_t2,Noonan_Syndrome_and_Related
RAG1	Inflammatory_Bowel_t2,Primary_immune_deficiency_t1,Hemolytic_anemia,Primary_immunodeficiency,Severe_Combined_Immunodeficiency_and_CID
RAG2	Inflammatory_Bowel_t2,Primary_immune_deficiency_t1,Hemolytic_anemia,Primary_immunodeficiency,Very-early-onset_inflammatory_bowel_disease,Severe_Combined_Immunodeficiency_and_CID
RAI1	Lysosomal_storage_disease_t2,Epilepsy_t2,Hereditary_Autism_t1,Autism_and_Related
RALA	Skeletal_dysplasia_t2,Proportionate_short_stature_t2,Epilepsy_t2
RANBP2	Epilepsy_t2
RANGRF	Cardiomyopathy_t2,Arrhythmia_t1,Arrhythmia_and_Related
RAPSN	Hereditary_Autism_t2,Myopathy_t1,Congenital_myopathy,Myopathy_and_Related
RARA	ALL_and_others_t2
RARB	Hereditary_retinopathy_t2
RARS1	Hereditary_spastic_paraplegia_t2,Epilepsy_t2,Neurodegenerative_disease_t2
RARS2	Epilepsy_t2,Hereditary_Microcephaly_t2
RASA1	Cardiomyopathy_t2,Rasopathies_t2,Primary_Pulmonary_Hypertension_and_Related_Disordisorders
RASA2	Cardiomyopathy_t2,Skeletal_dysplasia_t2,Proportionate_short_stature_t2,Rasopathies_t2
RASGRP2	Coagulation_t2,Bleeding_Platelet_Disorder
RAX	Hereditary_retinopathy_t2
RAX2	Hereditary_retinopathy_t2,Cone-Rod_Dystrophy_and_Related,Macular_dystrophy_and_Related,Retinitis_Pigmentosa_and_Related,retina
RB1	Dermatology_t2,Hereditary_retinopathy_t2,Hereditary_cancer_syndrome_Plus_t2,Cell-free_cancer,Hematologic_malignancy,Hereditary_pancancer,ALL_and_others_t1,ALL_and_others_t2,AML,Germline_Cancer,Lymphoma_B_cell_t1,Lymphoma_B_cell_t2,MDS_MPN,Myeloma_t1,retina
RBBP6	Hematologic_malignancy
RBBP8	Skeletal_dysplasia_t2,Proportionate_short_stature_t2,Hereditary_Microcephaly_t2,Hereditary_Autism_t2
RBCK1	Cardiomyopathy_t2,Muscular_dystrophy_t2,Myopathy_t2,Metabolic_myopathy,Severe_Combined_Immunodeficiency_and_CID
RBFOX1	Epilepsy_t2,Hereditary_Autism_t2,Focal_epilepsy
RBFOX3	Epilepsy_t2

Supplementary Table 2. List of pathogenic or likely-pathogenic germline variants

Gene	Associated diseases
RBM10	Hereditary_Autism_t2
RBM20	Arrhythmia_t2,Cardiomyopathy_t1,Dilated_cardiomyopathy,Cardiomyopathy_and_Related
RBM28	Hypogonadotropic_hypogonadism_t2
RBM8A	Skeletal_dysplasia_t2,Coagulation_t2,Bleeding_Platelet_Disorder
RBP3	Hereditary_retinopathy_t2,Retinitis_pigmentosa_t1,Retinitis_pigmentosa,Retinitis_Pigmentosa_and_Related,retina
RBP4	Retinitis_pigmentosa_t2,Hereditary_retinopathy_t2,Retinitis_Pigmentosa_and_Related,retina
RBPJ	Dermatology_t2,Skeletal_dysplasia_t2
RCBTB1	Retinitis_pigmentosa_t2,Hereditary_retinopathy_t2
RD3	Retinitis_pigmentosa_t2,Hereditary_retinopathy_t1,Leber_congenital_amaurosis,Leber's_Congenital_Amaurosis_and_Related,Retinitis_Pigmentosa_and_Related,retina
RDH11	Retinitis_pigmentosa_t2,Hereditary_retinopathy_t2,Retinitis_Pigmentosa_and_Related,retina
RDH12	Hereditary_retinopathy_t2,Retinitis_pigmentosa_t1,Leber_congenital_amaurosis,Retinitis_pigmentosa,Leber's_Congenital_Amaurosis_and_Related,Macular_dystrophy_and_Related,Retinitis_Pigmentosa_and_Related,retina
RDH5	Retinitis_pigmentosa_t2,Hereditary_retinopathy_t2,Macular_dystrophy,Cone-Rod_Dystrophy_and_Related,Congenital_Stationary_Night_Blindness_and_Related,Leber's_Congenital_Amaurosis_and_Related,Macular_dystrophy_and_Related,Retinitis_Pigmentosa_and_Related,retina
RDX	Hearing_loss_t2,Hereditary_hearing_loss
RECQL4	Dermatology_t2,Skeletal_dysplasia_t2,Hereditary_retinopathy_t2,Craniosynostosis,Familial_hemophagocytic_lymphohistiocytosis,Hereditary_pancancer,Hemophagocytic_Lymphohistiocytosis_and_Related
REEP1	Charcot-Marie-Tooth_disease_t2,Ataxia_t2,Neurodegenerative_disease_t2,Amyotrophic_lateral_sclerosis_t2,Hereditary_spastic_paraplegia_t1,Hereditary_spastic_paraplegia_panel,Charcot_Marie_Tooth_and_Related,Spastic_Paraplegia_and_Related
REEP2	Hereditary_spastic_paraplegia_t2,Neurodegenerative_disease_t2
REEP6	Hereditary_retinopathy_t2,Retinitis_pigmentosa_t1,Retinitis_pigmentosa,Retinitis_Pigmentosa_and_Related,retina
REL	Lymphoma_T_NK_cell_t2
RELN	Epilepsy_t2,Hereditary_Microcephaly_t2,Dystonia_t1,Hereditary_Autism_t1,Focal_epilepsy,Malformation_of_cortical_development,Dystonia_and_Related
REN	Anemia
REST	Hereditary_hearing_loss
RET	Hereditary_cancer_syndrome_Plus_t1,Neuroendocrine_tumor_t1,Cell-free_cancer,Hereditary_pancancer,Hereditary_paraganglioma-pheochromocytoma_syndrome,Hirschsprung's_disease,ALL_and_others_t2,Circulating_tumor_DNA_assay_55,Germline_Cancer,Lymphoma_B_cell_t2,Pheochromocytoma-Paraganglioma_Panel
RETREG1	Ataxia_t2,Charcot-Marie-Tooth_disease_t1,Charcot_Marie_Tooth_and_Related
RFC1	Ataxia
RFT1	Epilepsy_t2
RFWD3	Congenital_hematologic_disease,Bone_Marrow_Failure_Germline_Predisposition
RFX5	Primary_immune_deficiency_t2,Severe_Combined_Immunodeficiency_and_CID
RFX6	Maturity-Onset_Diabetes_of_the_Young,_MODY_t1,Congenital_diarrhea,Monogenic_diabetes_panel
RFXANK	Primary_immune_deficiency_t2,Severe_Combined_Immunodeficiency_and_CID
RFXAP	Primary_immune_deficiency_t2,Severe_Combined_Immunodeficiency_and_CID
RGR	Hereditary_retinopathy_t2,Retinitis_pigmentosa_t1,Retinitis_pigmentosa,Retinitis_Pigmentosa_and_Related,retina
RGS9	Hereditary_retinopathy_t2,Achromatopsia_and_Related,Cone-Rod_Dystrophy_and_Related,Retinitis_Pigmentosa_and_Related,retina
RGS9BP	Hereditary_retinopathy_t2,Achromatopsia_and_Related,Cone-Rod_Dystrophy_and_Related,retina
RHAG	Anemia_t1,Anemia
RHEB	Cell-free_cancer
RHO	Hereditary_retinopathy_t2,Retinitis_pigmentosa_t1,Retinitis_pigmentosa,Congenital_Stationary_Night_Blindness_and_Related,Macular_dystrophy_and_Related,Retinitis_Pigmentosa_and_Related,retina

Supplementary Table 2. List of pathogenic or likely-pathogenic germline variants

Gene	Associated diseases
RHOA	Lymphoma_t1,Cell-free_cancer,Hematologic_malignancy,Lymphoma_B_cell_t1,Lymphoma_B_cell_t2,Lymphoma_T_NK_cell_t1,Lymphoma_T_NK_cell_t2
RHOH	Primary_immune_deficiency_t2
RICTOR	Lymphoma_B_cell_t2,Lymphoma_T_NK_cell_t2
RIMS1	Retinitis_pigmentosa_t2,Hereditary_retinopathy_t2,Macular_dystrophy,Cone-Rod_Dystrophy_and_Related,Retinitis_Pigmentosa_and_Related,retina
RIN2	Connective_tissue_disorder_t1,Connective_Tissue_Disorder_and_Related
RIPOR2	Hearing_loss_t2,Hereditary_hearing_loss
RIT1	Cardiomyopathy_t2,Skeletal_dysplasia_t2,Proportionate_short_stature_t2,Hereditary_Autism_t2,Rasopathies_t1,Cell-free_cancer,Noonan_syndrome,Noonan_Syndrome_and_Related
RLBP1	Hereditary_retinopathy_t2,Retinitis_pigmentosa_t1,Retinitis_pigmentosa,Congenital_Stationary_Night_Blindness_and_Related,Macular_dystrophy_and_Related,Retinitis_Pigmentosa_and_Related,retina
RLIM	Hereditary_Autism_t2
RMND1	Cardiomyopathy_t2,Epilepsy_t2,Hearing_loss_t2
RMRP	Dermatology_t2,Skeletal_dysplasia_t2,Primary_immune_deficiency_t2,Skeletal_Dysplasia_Panel,Spondyloepiphyseal_metaphyseal_dysplasia,Severe_Combined_Immunodeficiency_and_CID
RNASEH2A	Epilepsy_t2
RNASEH2B	Epilepsy_t2
RNASEH2C	Epilepsy_t2,Hereditary_Microcephaly_t2
RNASET2	Epilepsy_t2
RNF213	Hereditary_Stroke_t1,Moyamoya_disease,Stroke,Primary_Pulmonary_Hypertension_and_Related_Disordisorders
RNF216	Ataxia_t2,Epilepsy_t2,Hypogonadotropic_hypogonadism_t2,Dementia_t2,Neurodegenerative_disease_t2
RNF31	Severe_Combined_Immunodeficiency_and_CID
RNF43	Cell-free_cancer,Colorectal_Cancer_Polyposis_Panel
RNU4ATAC	Skeletal_dysplasia_t2,Proportionate_short_stature_t2,Skeletal_Dysplasia_Panel
ROGDI	Epilepsy_t2
ROM1	Retinitis_pigmentosa_t2,Hereditary_retinopathy_t2,Retinitis_pigmentosa,Retinitis_Pigmentosa_and_Related,retina
ROR1	Hearing_loss_t2,Hereditary_hearing_loss
ROR2	Skeletal_dysplasia_t2,Proportionate_short_stature_t2,Skeletal_Dysplasia_Panel
RORA	Ataxia_t2,Hereditary_Autism_t2
RORB	Epilepsy_t2
RORC	Primary_immune_deficiency_t2,Phagocyte_Defect_and_Infectious_disease
ROS1	Cell-free_cancer,Circulating_tumor_DNA_assay_55
RP1	Hereditary_retinopathy_t2,Retinitis_pigmentosa_t1,Retinitis_pigmentosa,Retinitis_Pigmentosa_and_Related,retina
RP1L1	Retinitis_pigmentosa_t2,Hereditary_retinopathy_t2,Macular_dystrophy,Retinitis_pigmentosa,Cone-Rod_Dystrophy_and_Related,Macular_dystrophy_and_Related,Retinitis_Pigmentosa_and_Related,retina
RP2	Hereditary_retinopathy_t2,Retinitis_pigmentosa_t1,Retinitis_pigmentosa,Retinitis_Pigmentosa_and_Related,retina
RP9	Hereditary_retinopathy_t2,Retinitis_pigmentosa_t1,Retinitis_pigmentosa,Retinitis_Pigmentosa_and_Related,retina
RPE65	Hereditary_retinopathy_t2,Retinitis_pigmentosa_t1,Leber_congenital_amaurosis,Retinitis_pigmentosa,Congenital_Stationary_Night_Blindness_and_Related,Leber's_Congenital_Amaurosis_and_Related,Retinitis_Pigmentosa_and_Related,retina
RPGR	Hereditary_retinopathy_t2,Hereditary_Primary_Ciliary_Dyskinesia_t2,Retinitis_pigmentosa_t1,Macular_dystrophy,Retinitis_pigmentosa,Cone-Rod_Dystrophy_and_Related,Macular_dystrophy_and_Related,Retinitis_Pigmentosa_and_Related,retina
RPGRIP1	Retinitis_pigmentosa_t2,Hereditary_retinopathy_t1,Leber_congenital_amaurosis,Macular_dystrophy,Cone-Rod_Dystrophy_and_Related,Leber's_Congenital_Amaurosis_and_Related,Retinitis_Pigmentosa_and_Related,retina
RPGRIP1L	Skeletal_dysplasia_t2,Ataxia_t2,Epilepsy_t2,Retinitis_pigmentosa_t2,Polycystic_Kidney_Disease_t2,Hereditary_Autism_t2,Hereditary_retinopathy_t1,Nephronophthisis_t1,Nephronophthisis_and_Related,Retinitis_Pigmentosa_and_Related,retina
RPL10	Hereditary_Autism_t2

Supplementary Table 2. List of pathogenic or likely-pathogenic germline variants

Gene	Associated diseases
RPL11	Coagulation_t2,Congenital_hematologic_disease,Diamond-Blackfan_anemia,Bone_Marrow_Failure_Germline_Predisposition
RPL15	Congenital_hematologic_disease,Diamond-Blackfan_anemia,Bone_Marrow_Failure_Germline_Predisposition
RPL18	Congenital_hematologic_disease,Diamond-Blackfan_anemia
RPL21	Dermatology_t2
RPL26	Anemia_t2,Congenital_hematologic_disease,Diamond-Blackfan_anemia,Bone_Marrow_Failure_Germline_Predisposition
RPL27	Congenital_hematologic_disease,Diamond-Blackfan_anemia
RPL31	Anemia_t2,Congenital_hematologic_disease,Diamond-Blackfan_anemia
RPL35	Congenital_hematologic_disease,Diamond-Blackfan_anemia
RPL35A	Coagulation_t2,Anemia_t2,Congenital_hematologic_disease,Diamond-Blackfan_anemia,Bone_Marrow_Failure_Germline_Predisposition
RPL36	Congenital_hematologic_disease,Diamond-Blackfan_anemia
RPL5	Coagulation_t2,Congenital_hematologic_disease,Diamond-Blackfan_anemia,Bone_Marrow_Failure_Germline_Predisposition
RPS10	Coagulation_t2,Anemia_t2,Congenital_hematologic_disease,Diamond-Blackfan_anemia,Bone_Marrow_Failure_Germline_Predisposition
RPS14	Hematologic_malignancy
RPS15	Lymphoma_t1
RPS15A	Congenital_hematologic_disease,Diamond-Blackfan_anemia
RPS17	Anemia_t2,Congenital_hematologic_disease,Diamond-Blackfan_anemia,Bone_Marrow_Failure_Germline_Predisposition
RPS19	Coagulation_t2,Anemia_t2,Congenital_hematologic_disease,Diamond-Blackfan_anemia,ALL_and_others_t2,Bone_Marrow_Failure_Germline_Predisposition
RPS24	Coagulation_t2,Anemia_t2,Congenital_hematologic_disease,Diamond-Blackfan_anemia,Bone_Marrow_Failure_Germline_Predisposition
RPS26	Coagulation_t2,Anemia_t2,Congenital_hematologic_disease,Diamond-Blackfan_anemia,Bone_Marrow_Failure_Germline_Predisposition
RPS27	Congenital_hematologic_disease,Diamond-Blackfan_anemia
RPS28	Congenital_hematologic_disease,Diamond-Blackfan_anemia
RPS29	Congenital_hematologic_disease,Diamond-Blackfan_anemia,Bone_Marrow_Failure_Germline_Predisposition
RPS6KA3	Hearing_loss_t2,Hereditary_Autism_t2,Proportionate_short_stature_t1
RPS7	Coagulation_t2,Anemia_t2,Congenital_hematologic_disease,Diamond-Blackfan_anemia,Bone_Marrow_Failure_Germline_Predisposition
RRAGC	Lymphoma_t1
RRAS	Cardiomyopathy_t2,Skeletal_dysplasia_t2,Proportionate_short_stature_t2,Rasopathies_t2
RRM2B	Myopathy_t2,Ataxia_t2
RS1	Retinitis_pigmentosa_t2,Hereditary_retinopathy_t2,Macular_dystrophy,Macular_dystrophy_and_Related,Retinitis_Pigmentosa_and_Related,Vitreoretinopathy_and_Related,retina
RSPH1	Hereditary_Primary_Ciliary_Dyskinesia_t1
RSPH3	Hereditary_Primary_Ciliary_Dyskinesia_t1
RSPH4A	Hereditary_Primary_Ciliary_Dyskinesia_t1
RSPH9	Hereditary_Primary_Ciliary_Dyskinesia_t1
RSPO1	Disorders_of_sexual_development_t2,Disorders_of_sex_development
RSPRY1	Skeletal_dysplasia_t2,Skeletal_Dysplasia_Panel
RTEL1	Dermatology_t2,Inflammatory_Bowel_t2,Congenital_hematologic_disease,Dyskeratosis_congenita,Bone_Marrow_Failure_Germline_Predisposition,Hereditary_Myeloid_Leukemia_Panel
RTN2	Ataxia_t2,Neurodegenerative_disease_t2,Hereditary_spastic_paraplegia_t1,Spastic_Paraplegia_and_Related
RTN4IP1	Hereditary_retinopathy_t2,Optic_neuropathy_and_Related
RTTN	Skeletal_dysplasia_t2,Proportionate_short_stature_t2,Hereditary_Microcephaly_t2
RUBCN	Ataxia_t2
RUNX1	Coagulation_t2,Cell-free_cancer,Congenital_hematologic_disease,Hematologic_malignancy,Hereditary_pancancer,Leukemia_predisposition,ALL_and_others_t1,ALL_and_others_t2,AML,Bleeding_Platelet_Disorder,Bone_Marrow_Failure_Germline_Predisposition,Hereditary_Myeloid_Leukemia_Panel,MDS_MPN

Supplementary Table 2. List of pathogenic or likely-pathogenic germline variants

Gene	Associated diseases
RUNX2	Skeletal_dysplasia_t1,Skeletal_Dysplasia_Panel,Skeletal_Dysplasia_and_Related
RXRA	corneal_keratoconus
RXYLT1	Muscular_dystrophy_t2,Myopathy_t2,Congenital_muscular_dystrophy
RYR1	Muscular_dystrophy_t2,Myopathy_t1,Congenital_myopathy,Myopathy_and_Related
RYR2	Cardiomyopathy_t2,Arrhythmia_t1,Arrhythmia,Hypertrophic_cardiomyopathy,Arrhythmia_and_Related,Sudden_Cardiac_Arrest_and_Related
RYR3	Epilepsy_t2
S1PR2	Hearing_loss_t2,Hereditary_hearing_loss
SACS	Charcot-Marie-Tooth_disease_t2,Hereditary_Autism_t2,Neurodegenerative_disease_t2,Ataxia_t1,Hereditary_spastic_paraplegia_t1,Ataxia,Ataxia_and_Related,Nystagmus_and_Related,Optic_neuropathy_and_Related,Spastic_Paraplegia_and_Related
SAG	Hereditary_retinopathy_t2,Retinitis_pigmentosa_t1,Retinitis_pigmentosa,Congenital_Stationary_Night_Blindness_and_Related,Retinitis_Pigmentosa_and_Related,retina
SALL1	Skeletal_dysplasia_t2,Hearing_loss_t2
SALL4	Arrhythmia_t2,Skeletal_dysplasia_t2,Hearing_loss_t2
SAMD11	Retinitis_pigmentosa_t2,Hereditary_retinopathy_t2,Retinitis_pigmentosa,Retinitis_Pigmentosa_and_Related,retina
SAMD9	Inflammatory_Bowel_t2,Congenital_hematologic_disease,Leukemia_predisposition,Hereditary_Myeloid_Leukemia_Panel
SAMD9L	Congenital_hematologic_disease,Leukemia_predisposition,Hereditary_Myeloid_Leukemia_Panel
SAMHD1	Epilepsy_t2,Hereditary_Autism_t2,Autoinflammatory_disorders,Autoimmunity_and_autoinflammatory_disorders
SAR1B	Hereditary_Dyslipidemia_t1,Congenital_diarrhea,Dyslipidemia_and_Related
SARS2	Atypical_Hemolytic_Uremic_Syndrome_t2
SASH1	Rasopathies_t2
SASS6	Hereditary_Microcephaly_t2
SATB2	Epilepsy_t2,Hereditary_Autism_t2,Autism_and_Related
SBDS	Skeletal_dysplasia_t2,Coagulation_t2,Congenital_hematologic_disease,Severe_congenital_neutropenia,Shwachman-Diamond_syndrome,Skeletal_Dysplasia_Panel,Spondyloepiphyseal_metaphyseal_dysplasia,ALL_and_others_t2,AML,Bone_Marrow_Failure_Germline_Predisposition,Hereditary_Myeloid_Leukemia_Panel,MDS_MPN
SBF1	Charcot-Marie-Tooth_disease_t2,Charcot-Marie-Tooth,Charcot_Marie_Tooth_and_Related
SBF2	Charcot-Marie-Tooth_disease_t1,Charcot-Marie-Tooth,Charcot_Marie_Tooth_and_Related,glaucoma
SCAPER	Retinitis_pigmentosa_t2,Hereditary_retinopathy_t2,Retinitis_Pigmentosa_and_Related
SCARB1	Hereditary_Dyslipidemia_t1,Dyslipidemia_and_Related
SCARB2	Epilepsy_t2,Epilepsy_and_Related
SCLT1	Retinitis_pigmentosa_t2,Hereditary_retinopathy_t2
SCN10A	Arrhythmia_t2,Epilepsy_t2,Charcot_Marie_Tooth_and_Related,Sudden_Cardiac_Arrest_and_Related
SCN11A	Charcot-Marie-Tooth_disease_t2,Charcot_Marie_Tooth_and_Related
SCN1A	Arrhythmia_t2,Epilepsy_t1,Hereditary_Stroke_t1,Hereditary_Autism_t1,Early_onset_epilepsy,Focal_epilepsy,Epilepsy_and_Related
SCN1B	Cardiomyopathy_t2,Arrhythmia_t1,Epilepsy_t1,Arrhythmia,Arrhythmia_and_Related
SCN2A	Epilepsy_t1,Hereditary_Autism_t1,Early_onset_epilepsy,Paroxysmal_movement_disorder,Autism_and_Related,Epilepsy_and_Related
SCN2B	Arrhythmia_t2
SCN3A	Epilepsy_t2,Focal_epilepsy
SCN3B	Cardiomyopathy_t2,Arrhythmia_t1,Arrhythmia,Arrhythmia_and_Related
SCN4A	Muscular_dystrophy_t2,Myopathy_t2,Ataxia_t2,Epilepsy_t2,Atypical_Hemolytic_Uremic_Syndrome_t2,Acid-base-electro_gene,Dyskinesia-dystonia-paralysis
SCN4B	Cardiomyopathy_t2,Arrhythmia_t1,Arrhythmia,Arrhythmia_and_Related
SCN5A	Epilepsy_t2,Hereditary_Stroke_t2,Arrhythmia_t1,Cardiomyopathy_t1,Arrhythmia,Dilated_cardiomyopathy,Arrhythmia_and_Related,Cardiomyopathy_and_Related,Sudden_Cardiac_Arrest_and_Related

Supplementary Table 2. List of pathogenic or likely-pathogenic germline variants

Gene	Associated diseases
SCN8A	Arrhythmia_t2,Hereditary_Autism_t2,Epilepsy_t1,Dyskinesia-dystonia-paralysis,Early_onset_epilepsy,Focal_epilepsy,Epilepsy_and_Related
SCN9A	Arrhythmia_t2,Ataxia_t2,Epilepsy_t2,Charcot-Marie-Tooth_disease_t1,Charcot_Marie_Tooth_and_Related
SCNN1A	Acid-base-electro_gene
SCNN1B	Cardiomyopathy_t2,Atypical_Hemolytic_Uremic_Syndrome_t2,Acid-base-electro_gene
SCNN1G	Cardiomyopathy_t2,Atypical_Hemolytic_Uremic_Syndrome_t2,Acid-base-electro_gene
SCO1	Cardiomyopathy_t2,Epilepsy_t2
SCO2	Cardiomyopathy_t2,Epilepsy_t2,Charcot-Marie-Tooth,Leigh_Syndrome_and_Related
SCP2	Dystonia_t2,Parkinson's_disease_t2,corneal_dystrophy
SCYL1	Charcot-Marie-Tooth_disease_t2,Ataxia_t2,Cholestasis_t2,Charcot_Marie_Tooth_and_Related
SDCCAG8	Hereditary_retinopathy_t2,Polycystic_Kidney_Disease_t2,Hereditary_Autism_t2,Nephronophthisis_t1,Nephronophthisis_and_Related,Retinitis_Pigmentosa_and_Related,retina
SDHA	Cardiomyopathy_t2,Epilepsy_t2,Hereditary_cancer_syndrome_Plus_t2,Neuroendocrine_tumor_t1,Dilated_cardiomyopathy,Hereditary_pancancer,Hereditary_paraganglioma-pheochromocytoma_syndrome,Germline_Cancer,Leigh_Syndrome_and_Related,Optic_neuropathy_and_Related,Pheochromocytoma-Paraganglioma_Panel
SDHAF1	Epilepsy_t2,Leigh_Syndrome_and_Related,Optic_neuropathy_and_Related
SDHAF2	Hereditary_cancer_syndrome_Plus_t2,Neuroendocrine_tumor_t1,Hereditary_pancancer,Hereditary_paraganglioma-pheochromocytoma_syndrome,Pheochromocytoma-Paraganglioma_Panel
SDHB	Hereditary_cancer_syndrome_Plus_t2,Neuroendocrine_tumor_t1,Hereditary_breast_cancer,Hereditary_pancancer,Hereditary_paraganglioma-pheochromocytoma_syndrome,Breast_and_Ovarian_Cancer_Panel,Germline_Cancer,Pheochromocytoma-Paraganglioma_Panel
SDHC	Hereditary_cancer_syndrome_Plus_t2,Neuroendocrine_tumor_t1,Hereditary_pancancer,Hereditary_paraganglioma-pheochromocytoma_syndrome,Pheochromocytoma-Paraganglioma_Panel
SDHD	Hereditary_cancer_syndrome_Plus_t2,Neuroendocrine_tumor_t1,Hereditary_breast_cancer,Hereditary_pancancer,Hereditary_paraganglioma-pheochromocytoma_syndrome,Pheochromocytoma-Paraganglioma_Panel
SDR9C7	Dermatology_t2
SEC23B	Coagulation_t2,Anemia_t2,Hemolytic_anemia,Anemia,Hemochromatosis
SEC24D	Skeletal_dysplasia_t2,Osteogenesis_imperfecta
SEC61A1	Polycystic_Kidney_Disease_t1,Polycystic_kidney_disease_and_Related
SEC63	Polycystic_Kidney_Disease_t1,Polycystic_kidney_disease_and_Related
SECISBP2	Hypothyroidism_t2,Congenital_Hypothyroidism
SELENON	Cardiomyopathy_t2,Muscular_dystrophy_t1,Myopathy_t1,Congenital_muscular_dystrophy,Congenital_myopathy,Myofibrillar_myopathy,Muscular_Dystrophy_and_Related,Myopathy_and_Related
SEMA3A	Hypogonadotropic_hypogonadism_t1
SEMA3E	Primary_immune_deficiency_t2,Hearing_loss_t2,Hypogonadotropic_hypogonadism_t2,Hereditary_hearing_loss
SEMA4A	Hereditary_retinopathy_t2,Retinitis_pigmentosa_t1,Macular_dystrophy,Retinitis_pigmentosa,Cone-Rod_Dystrophy_and_Related,Retinitis_Pigmentosa_and_Related,retina
SEMA7A	Hypogonadotropic_hypogonadism_t2
SEPSECS	Hereditary_Microcephaly_t2
SEPTIN9	Muscular_dystrophy_t2,Myopathy_t2,Charcot-Marie-Tooth_disease_t2,Charcot_Marie_Tooth_and_Related
SERAC1	Ataxia_t2,Epilepsy_t2
SERPINA1	Cholestasis_t1,Neonatal_cholestasis
SERPINA10	Atypical_Hemolytic_Uremic_Syndrome,Bleeding_Coagulopathy,Thrombosis
SERPINB6	Hearing_loss_t2,Hereditary_hearing_loss
SERPINB7	Dermatology_t2
SERPINC1	Coagulation_t2,Hereditary_Stroke_t2,Atypical_Hemolytic_Uremic_Syndrome,Bleeding_Coagulopathy,Thrombosis
SERPIND1	Coagulation_t2,Atypical_Hemolytic_Uremic_Syndrome,Bleeding_Coagulopathy,Thrombosis
SERPINE1	Coagulation_t1,Stroke,Atypical_Hemolytic_Uremic_Syndrome,Bleeding_Coagulopathy,Thrombosis
SERPINF1	Skeletal_dysplasia_t2,Osteogenesis_imperfecta
SERPINF2	Coagulation_t1,Atypical_Hemolytic_Uremic_Syndrome,Bleeding_Coagulopathy,Thrombosis

Supplementary Table 2. List of pathogenic or likely-pathogenic germline variants

Gene	Associated diseases
SERPINH1	Skeletal_dysplasia_t2,Osteogenesis_imperfecta
SERPINI1	Epilepsy_t2
SETBP1	Skeletal_dysplasia_t2,Epilepsy_t2,Hereditary_Autism_t2,Androgenetic_alopecia,Hematologic_malignancy,ALL_and_others_t1,ALL_and_others_t2,AML,MDS_MPN
SETD2	Epilepsy_t2,Hereditary_Autism_t2,Cell-free_cancer,Overgrowth_Panel,Lymphoma_T_NK_cell_t1,Lymphoma_T_NK_cell_t2
SETD5	Hereditary_Autism_t2
SETX	Charcot-Marie-Tooth_disease_t2,Hereditary_spastic_paraplegia_t2,Neurodegenerative_disease_t2,Ataxia_t1,Amyotrophic_lateral_sclerosis_t1,Amyotrophic_lateral_sclerosis,Ataxia,Charcot-Marie-Tooth,Chorea,Dystonia,Ataxia_and_Related,Charcot_Marie_Tooth_and_Related
SF3B1	Lymphoma_t1,Hematologic_malignancy,ALL_and_others_t1,ALL_and_others_t2,AML,Circulating_tumor_DNA_assay_55,Lymphoma_B_cell_t1,Lymphoma_B_cell_t2,MDS_MPN,Myeloma_t1
SF3B4	Skeletal_dysplasia_t2
SFRP4	Sclerosing_Bone_Disease_Panel
SFTPB	Dermatology_t2
SFTPC	Dermatology_t2
SGCA	Cardiomyopathy_t2,Myopathy_t2,Muscular_dystrophy_t1,Limb_girdle_muscular_dystrophy,Muscular_Dystrophy_and_Related
SGCB	Cardiomyopathy_t2,Myopathy_t2,Muscular_dystrophy_t1,Limb_girdle_muscular_dystrophy,Muscular_Dystrophy_and_Related
SGCD	Myopathy_t2,Cardiomyopathy_t1,Muscular_dystrophy_t1,Dilated_cardiomyopathy,Limb_girdle_muscular_dystrophy,Cardiomyopathy_and_Related,Muscular_Dystrophy_and_Related
SGCE	Muscular_dystrophy_t2,Myopathy_t2,Ataxia_t2,Epilepsy_t2,Parkinson's_disease_t2,Neurodegenerative_disease_t2,Dystonia_t1,Dyskinesia-dystonia-paralysis,Dystonia,Dystonia_and_Related
SGCG	Cardiomyopathy_t2,Myopathy_t2,Muscular_dystrophy_t1,Limb_girdle_muscular_dystrophy,Muscular_Dystrophy_and_Related
SGK1	Lymphoma_t2,Lymphoma_B_cell_t1,Lymphoma_B_cell_t2,Lymphoma_T_NK_cell_t2
SGMS2	Skeletal_dysplasia_t2,Proportionate_short_stature_t2,Osteogenesis_imperfecta
SGPL1	Charcot-Marie-Tooth
SGSH	Lysosomal_storage_disease_t2,Epilepsy_t2,Hereditary_Autism_t2,Mucopolysaccharidosis,Skeletal_Dysplasia_Panel,Lysosomal_Storage_Disease
SH2B3	Congenital_hematologic_disease,Hematologic_malignancy,Leukemia_predisposition,ALL_and_others_t1,ALL_and_others_t2,AML,Bone_Marrow_Failure_Germline_Predisposition,Erythrocytosis,Hereditary_Myeloid_Leukemia_Panel,MDS_MPN
SH2D1A	Primary_immune_deficiency_t1,Inflammatory_Bowel_t1,Familial_hemophagocytic_lymphohistiocytosis,Very-early-onset_inflammatory_bowel_disease,B_cell_and_Humoral_Immune_Deficiency,Hemophagocytic_Lymphohistiocytosis_and_Related
SH3BP2	Skeletal_dysplasia_t2
SH3PXD2B	Skeletal_dysplasia_t2,Skeletal_Dysplasia_Panel,glaucoma
SH3TC2	Charcot-Marie-Tooth_disease_t1,Charcot-Marie-Tooth,Charcot_Marie_Tooth_and_Related
SHANK2	Hereditary_Autism_t1
SHANK3	Hereditary_Autism_t2,Autism_and_Related
SHH	Skeletal_dysplasia_t2,Epilepsy_t2,Hereditary_retinopathy_t2
SHOC2	Cardiomyopathy_t2,Dermatology_t2,Skeletal_dysplasia_t2,Proportionate_short_stature_t2,Epilepsy_t2,Hereditary_Autism_t2,Rasopathies_t1,Noonan_syndrome,Noonan_Syndrome_and_Related
SHOX	Skeletal_dysplasia_t2,Proportionate_short_stature_t2,Skeletal_Dysplasia_Panel
SHROOM4	Hereditary_Autism_t2
SI	Congenital_diarrhea
SIGMAR1	Dementia_t1,Neurodegenerative_disease_t1,Amyotrophic_lateral_sclerosis_t1,Amyotrophic_lateral_sclerosis,Charcot-Marie-Tooth,Charcot_Marie_Tooth_and_Related
SIK1	Epilepsy_t2
SIL1	Hereditary_retinopathy_t2,Hereditary_Autism_t2,Ataxia_t1,Ataxia,Myofibrillar_myopathy,Ataxia_and_Related
SIPA1L3	Hereditary_retinopathy_t2
SIX1	Hearing_loss_t2,Hereditary_hearing_loss
SIX3	Epilepsy_t2
SIX5	Polycystic_Kidney_Disease_t2,Hearing_loss_t1,Hereditary_hearing_loss,Hearing_Loss_and_Related_t1,Hearing_Loss_and_Related_t2

Supplementary Table 2. List of pathogenic or likely-pathogenic germline variants

Gene	Associated diseases
SIX6	Hereditary_retinopathy_t2
SKI	Skeletal_dysplasia_t2,Hereditary_Autism_t2,Connective_tissue_disorder_t1,Craniosynostosis,Familial_thoracic_aortic_aneurysms_and_aortic_dissections,Connective_Tissue_Disorder_and_Related,Marfan_and_Related
SKIV2L	Inflammatory_Bowel_t2,Congenital_diarrhea
SLC10A1	Neonatal_cholestasis
SLC10A2	Congenital_diarrhea,Neonatal_cholestasis
SLC10A7	Skeletal_Dysplasia_Panel
SLC11A2	Anemia
SLC12A1	Atypical_Hemolytic_Uremic_Syndrome_t2,Acid-base-electro_gene
SLC12A3	Atypical_Hemolytic_Uremic_Syndrome_t2,Acid-base-electro_gene,Renal_Tubular_Disorders
SLC12A5	Epilepsy_t2
SLC12A6	Charcot-Marie-Tooth_disease_t2,Ataxia_t2,Charcot_Marie_Tooth_and_Related
SLC13A5	Epilepsy_t2
SLC14A2	Androgenetic_alopecia
SLC16A1	Maturity-Onset_Diabetes_of_the_Young,_MODY_t2
SLC16A12	Hereditary_retinopathy_t2
SLC16A2	Ataxia_t2,Dystonia_t2,Parkinson's_disease_t2,Hereditary_Autism_t2,Neurodegenerative_disease_t2,Hereditary_spastic_paraplegia_t1,Hypothyroidism_t1,Hereditary_spastic_paraplegia_panel,Congenital_Hypothyroidism,Spastic_Paraplegia_and_Related
SLC17A5	Lysosomal_storage_disease_t2,Epilepsy_t2,Skeletal_Dysplasia_Panel
SLC17A8	Hearing_loss_t2,Hereditary_hearing_loss
SLC19A2	Hearing_loss_t2,Maturity-Onset_Diabetes_of_the_Young,_MODY_t1,Anemia_t1,Anemia,Retinitis_Pigmentosa_and_Related
SLC19A3	Hereditary_spastic_paraplegia_t2,Inborn_error_of_metabolism_t2,Epilepsy_t2,Neurodegenerative_disease_t2,Dystonia_t1,Dystonia_and_Related,Leigh_Syndrome_and_Related
SLC1A3	Ataxia_t2,Epilepsy_t2,Ataxia,Paroxysmal_movement_disorder
SLC1A4	Hereditary_spastic_paraplegia_t2,Epilepsy_t2,Hereditary_Microcephaly_t2,Neurodegenerative_disease_t2
SLC20A2	Ataxia_t2,Dystonia_t2,Parkinson's_disease_t2,Hereditary_Autism_t2,Chorea,Dystonia,Fahr's_disease,Parkinson
SLC22A12	Ca-Pi-Mg
SLC22A4	Hearing_loss_t2,Hereditary_hearing_loss
SLC22A5	Cardiomyopathy_t2,Myopathy_t2,Lysosomal_storage_disease_t2,Inborn_error_of_metabolism_t1,Metabolic_myopathy,Inborn_Error_of_Metabolism
SLC24A1	Hereditary_retinopathy_t2,Congenital_Stationary_Night_Blindness_and_Related,Retinitis_Pigmentosa_and_Related,retina
SLC24A5	Dermatology_t2,Hereditary_retinopathy_t2,Albinism_and_Related,Optic_neuropathy_and_Related
SLC25A1	Epilepsy_t2
SLC25A12	Epilepsy_t2,Hereditary_Autism_t2
SLC25A13	Inborn_error_of_metabolism_t2,Hereditary_Autism_t2,Cholestasis_t1,Neonatal_cholestasis,Urea_cycle_disorders,Inborn_Error_of_Metabolism
SLC25A15	Hereditary_spastic_paraplegia_t2,Lysosomal_storage_disease_t2,Epilepsy_t2,Hereditary_Autism_t2,Neurodegenerative_disease_t2,Urea_cycle_disorders
SLC25A19	Epilepsy_t2,Hereditary_Microcephaly_t1
SLC25A20	Arrhythmia_t2,Cardiomyopathy_t2,Myopathy_t2,Inborn_error_of_metabolism_t2
SLC25A22	Epilepsy_t1,Early_onset_epilepsy
SLC25A38	Anemia_t1,Anemia,Hemochromatosis
SLC25A4	Cardiomyopathy_t2,Hypertrophic_cardiomyopathy
SLC25A42	Epilepsy_t2
SLC25A46	Charcot-Marie-Tooth_disease_t2,Ataxia_t2,Hereditary_retinopathy_t2,Charcot_Marie_Tooth_and_Related,Optic_neuropathy_and_Related,retina
SLC26A2	Skeletal_dysplasia_t1,Skeletal_Dysplasia_Panel,Spondyloepiphyseal_metaphyseal_dysplasia,Skeletal_Dysplasia_and_Related

Supplementary Table 2. List of pathogenic or likely-pathogenic germline variants

Gene	Associated diseases
SLC26A3	Atypical_Hemolytic_Uremic_Syndrome_t2,Cholestasis_t2,Acid-base-electro_gene,Congenital_diarrhea
SLC26A4	Hypothyroidism_t1,Hearing_loss_t1,Hereditary_hearing_loss,Congenital_Hypothyroidism,Hearing_Loss_and_Related_t1,Hearing_Loss_and_Related_t2
SLC26A5	Hearing_loss_t2,Hereditary_hearing_loss
SLC27A4	Dermatology_t2
SLC29A1	Hematologic_malignancy
SLC29A3	Skeletal_dysplasia_t2,Hearing_loss_t2,Sclerosing_Bone_Disease_Panel
SLC2A1	Arrhythmia_t2,Ataxia_t2,Parkinson's_disease_t2,Hereditary_Stroke_t2,Hereditary_Autism_t2,Neurodegenerative_disease_t2,Anemia_t2,Epilepsy_t1,Dystonia_t1,Dyskinesia-dystonia-paralysis,Dystonia,Early_onset_epilepsy,Paroxysmal_movement_disorder,Anemia,Dystonia_and_Related,Epilepsy_and_Related
SLC2A10	Dermatology_t2,Connective_tissue_disorder_t2,Hereditary_Stroke_t1,Familial_thoracic_aortic_aneurysms_and_aortic_dissections,Connective_Tissue_Disorder_and_Related,Marfan_and_Related
SLC2A2	Lysosomal_storage_disease_t2,Hereditary_Autism_t2,Maturity-Onset_Diabetes_of_the_Young,_MODY_t1,Glycogen_storage_disease,Monogenic_diabetes_panel
SLC2A9	Ca-Pi-Mg
SLC30A10	Parkinson's_disease_t2,Neurodegenerative_disease_t2,Dystonia_t1,Dystonia,Parkinson,Dystonia_and_Related
SLC30A2	Dermatology_t2
SLC33A1	Ataxia_t2,Hereditary_spastic_paraplegia_t2,Hearing_loss_t2,Neurodegenerative_disease_t2
SLC34A1	Skeletal_dysplasia_t2,Atypical_Hemolytic_Uremic_Syndrome_t2,Ca-Pi-Mg
SLC34A3	Skeletal_dysplasia_t1,Rickets_Hypoparathyroidism_panel,Skeletal_Dysplasia_Panel,Skeletal_Dysplasia_and_Related
SLC35A1	Epilepsy_t2
SLC35A2	Epilepsy_t2,Hereditary_Autism_t2
SLC35A3	Epilepsy_t2
SLC35C1	Epilepsy_t2,Hereditary_Autism_t2,Primary_immunodeficiency
SLC35D1	Skeletal_dysplasia_t2,Proportionate_short_stature_t2,Skeletal_Dysplasia_Panel
SLC37A4	Lysosomal_storage_disease_t1,Inflammatory_Bowel_t1,Congenital_hematologic_disease,Glycogen_storage_disease,Severe_congenital_neutropenia,Very-early-onset_inflammatory_bowel_disease,Bone_Marrow_Failure_Germline_Predisposition,Lysosomal_Storage_Disease
SLC38A8	Dermatology_t2,Albinism_and_Related
SLC39A13	Dermatology_t2,Skeletal_dysplasia_t2,Connective_tissue_disorder_t2,Skeletal_Dysplasia_Panel
SLC39A14	Dystonia_t2,Parkinson's_disease_t2
SLC39A4	Dermatology_t2,Ataxia_t2,Congenital_diarrhea
SLC39A8	Epilepsy_t2
SLC3A1	Ca-Pi-Mg
SLC40A1	Anemia,Hemochromatosis
SLC45A2	Dermatology_t2,Hereditary_retinopathy_t2,Albinism_and_Related
SLC46A1	Lysosomal_storage_disease_t2,Epilepsy_t2,Hereditary_Autism_t2
SLC4A1	Skeletal_dysplasia_t2,Atypical_Hemolytic_Uremic_Syndrome_t2,Anemia_t1,Acid-base-electro_gene,Hemolytic_anemia,Anemia,Renal_Tubular_Disorders
SLC4A10	Epilepsy_t2
SLC4A11	Hearing_loss_t2,Hereditary_retinopathy_t2,Corneal_Dystrophy_and_Related,corneal_dystrophy,corneal_keratoconus
SLC4A3	Retinitis_Pigmentosa_and_Related
SLC4A4	Hereditary_Autism_t2,Acid-base-electro_gene,Renal_Tubular_Disorders,glaucoma
SLC4A7	Hereditary_retinopathy_t2
SLC52A2	Hearing_loss_t2,Hereditary_retinopathy_t2,Neurodegenerative_disease_t2,Amyotrophic_lateral_sclerosis_t2,Ataxia_t1,Ataxia,Ataxia_and_Related
SLC52A3	Ataxia_t2,Hearing_loss_t2,Neurodegenerative_disease_t2,Amyotrophic_lateral_sclerosis_t2
SLC5A1	Congenital_diarrhea
SLC5A2	Ca-Pi-Mg

Supplementary Table 2. List of pathogenic or likely-pathogenic germline variants

Gene	Associated diseases
SLC5A5	Hereditary_Autism_t2,Hypothyroidism_t1,Congenital_Hypothyroidism
SLC5A7	Congenital_myopathy,Charcot_Marie_Tooth_and_Related
SLC6A1	Epilepsy_t2,Hereditary_Autism_t2,Early_onset_epilepsy
SLC6A3	Alzheimer's_disease_t2,Dystonia_t2,Dementia_t2,Parkinson's_disease_t1,Neurodegenerative_disease_t1,Dyskinesia-dystonia-paralysis,Dystonia,Parkinson
SLC6A4	Hereditary_Autism_t1
SLC6A8	Inborn_error_of_metabolism_t2,Epilepsy_t2,Hereditary_Autism_t2
SLC7A14	Hereditary_retinopathy_t2,Retinitis_pigmentosa_t1,Retinitis_pigmentosa,Retinitis_Pigmentosa_and_Related,retina
SLC7A7	Hereditary_Autism_t2,Familial_hemophagocytic_lymphohistiocytosis,Urea_cycle_disorders,Hemophagocytic_Lymphohistiocytosis_and_Related
SLC7A9	Ca-Pi-Mg
SLC9A1	Ataxia_t2
SLC9A3	Congenital_diarrhea
SLC9A3R1	Skeletal_dysplasia_t2
SLC9A6	Ataxia_t2,Epilepsy_t2,Hereditary_Microcephaly_t2,Hereditary_Autism_t2,Autism_and_Related
SLC9A9	Hereditary_Autism_t1
SLCO1B1	Hereditary_Dyslipidemia_t2,Anemia_t2,Hemolytic_anemia,Neonatal_cholestasis,Anemia
SLCO1B3	Anemia_t2,Hemolytic_anemia,Neonatal_cholestasis,Anemia
SLCO2A1	Skeletal_dysplasia_t2,Sclerosing_Bone_Disease_Panel
SLFN14	Coagulation_t2,Bleeding_Platelet_Disorder
SLITRK6	Hearing_loss_t2
SLMAP	Arrhythmia_t2,Arrhythmia
SLURP1	Dermatology_t2
SLX4	Dermatology_t2,Skeletal_dysplasia_t2,Coagulation_t2,Hereditary_Autism_t2,Congenital_hematologic_disease,Fanconi_anemia,Hereditary_pancancer,Bone_Marrow_Failure_Germline_Predisposition
SMAD1	Primary_Pulmonary_Hypertension_and_Related_Disordisorders
SMAD2	Connective_tissue_disorder_t2,Overgrowth_Panel
SMAD3	Dermatology_t2,Skeletal_dysplasia_t2,Charcot-Marie-Tooth_disease_t2,Connective_tissue_disorder_t1,Familial_thoracic_aortic_aneurysms_and_aortic_dissections,Overgrowth_Panel,Connective_Tissue_Disorder_and_Related,Marfan_and_Related
SMAD4	Skeletal_dysplasia_t2,Proportionate_short_stature_t2,Connective_tissue_disorder_t2,Hearing_loss_t2,Hereditary_cancer_syndrome_Plus_t1,Cell-free_cancer,Familial_thoracic_aortic_aneurysms_and_aortic_dissections,Hereditary_colon_cancer,Hereditary_pancancer,Skeletal_Dysplasia_Panel,Anemia,Circulating_tumor_DNA_assay_55,Colorectal_Cancer_Polyposis_Panel,Lymphoma_B_cell_t2,Primary_Pulmonary_Hypertension_and_Related_Disordisorders
SMAD6	Connective_tissue_disorder_t2
SMAD9	Primary_Pulmonary_Hypertension_and_Related_Disordisorders
SMARCA2	Skeletal_dysplasia_t2,Proportionate_short_stature_t2,Epilepsy_t2,Hereditary_Microcephaly_t2,Hereditary_Autism_t2
SMARCA4	Skeletal_dysplasia_t2,Hereditary_Autism_t2,Hereditary_pancancer,Germline_Cancer,Lymphoma_B_cell_t1,Lymphoma_B_cell_t2
SMARCAD1	Dermatology_t2
SMARCAL1	Skeletal_dysplasia_t2,Primary_immune_deficiency_t2,Proportionate_short_stature_t1,Glomerulopathy,Skeletal_Dysplasia_Panel,Severe_Combined_Immunodeficiency_and_CID
SMARCB1	Dermatology_t2,Skeletal_dysplasia_t2,Rasopathies_t2,Hereditary_Autism_t2,Hereditary_pancancer,Germline_Cancer
SMARCE1	Skeletal_dysplasia_t2,Proportionate_short_stature_t2,Hereditary_Microcephaly_t2,Hereditary_pancancer
SMC1A	Skeletal_dysplasia_t2,Epilepsy_t2,Hereditary_Autism_t2,Proportionate_short_stature_t1,Hematologic_malignancy
SMC3	Skeletal_dysplasia_t2,Epilepsy_t2,Hereditary_Autism_t2,Proportionate_short_stature_t1,Hematologic_malignancy
SMCHD1	Cardiomyopathy_t2,Muscular_dystrophy_t2,Myopathy_t2,Limb_girdle_muscular_dystrophy
SMG6	Hereditary_Autism_t2
SMN1	Congenital_myopathy

Supplementary Table 2. List of pathogenic or likely-pathogenic germline variants

Gene	Associated diseases
SMO	Cell-free_cancer,Circulating_tumor_DNA_assay_55
SMOC1	Hereditary_retinopathy_t2
SMPD1	Dystonia_t2,Parkinson's_disease_t2,Lysosomal_storage_disease_t1,Cholestasis_t1,Bleeding_Platelet_Disorder
SMPX	Hearing_loss_t2,Hereditary_hearing_loss,Hearing_Loss_and_Related_t2
SMS	Epilepsy_t2,Hereditary_Autism_t2
SNAI2	Dermatology_t2,Hearing_loss_t1,Hereditary_hearing_loss,Albinism_and_Related,Hearing_Loss_and_Related_t1,Hearing_Loss_and_Related_t2
SNAP25	Epilepsy_t2,Hereditary_Autism_t2
SNAP29	Dermatology_t2,Hereditary_Microcephaly_t2
SNCA	Alzheimer's_disease_t2,Dystonia_t2,Dementia_t2,Amyotrophic_lateral_sclerosis_t2,Parkinson's_disease_t1,Neurodegenerative_disease_t1,Parkinson,Parkinson_Disease_and_Related
SNCB	Alzheimer's_disease_t2,Dementia_t2,Parkinson's_disease_t2,Neurodegenerative_disease_t2,Dementia_and_Related
SNIP1	Hereditary_Autism_t2
SNORD118	Epilepsy_t2
SNRNP200	Hereditary_retinopathy_t2,Retinitis_pigmentosa_t1,Retinitis_pigmentosa,Retinitis_Pigmentosa_and_Related,retina
SNRPE	Dermatology_t2
SNRPN	Hereditary_Autism_t2
SNTA1	Cardiomyopathy_t2,Arrhythmia_t1,Arrhythmia,Arrhythmia_and_Related
SNX10	Skeletal_dysplasia_t2,Hereditary_retinopathy_t2,Sclerosing_Bone_Disease_Panel
SNX14	Ataxia_t1,Ataxia,Ataxia_and_Related
SNX27	Epilepsy_t2
SOBP	Hereditary_Autism_t2
SOCS1	Lymphoma_t1,Lymphoma_B_cell_t1,Lymphoma_B_cell_t2,Lymphoma_T_NK_cell_t2
SOD1	Ataxia_t2,Neurodegenerative_disease_t2,Hereditary_spastic_paraplegia_t1,Amyotrophic_lateral_sclerosis_t1,Amyotrophic_lateral_sclerosis,Spastic_Paraplegia_and_Related,corneal_keratoconus
SORL1	Dementia_t2,Alzheimer's_disease_t1,Neurodegenerative_disease_t1,Dementia_and_Related
SOS1	Dermatology_t2,Skeletal_dysplasia_t2,Hereditary_Autism_t2,Cardiomyopathy_t1,Proportionate_short_stature_t1,Rasopathies_t1,Noonan_syndrome,Bone_Marrow_Failure_Germline_Predisposition,Cardiomyopathy_and_Related,Noonan_Syndrome_and_Related
SOS2	Cardiomyopathy_t2,Hereditary_Autism_t2,Rasopathies_t1,Noonan_syndrome
SOST	Skeletal_dysplasia_t2,Sclerosing_Bone_Disease_Panel
SOX10	Dermatology_t2,Epilepsy_t2,Hereditary_retinopathy_t2,Hypogonadotropic_hypogonadism_t2,Hereditary_Autism_t2,Hearing_loss_t1,Hereditary_hearing_loss,Hirschsprung's_disease,Albinism_and_Related,Charcot_Marie_Tooth_and_Related,Hearing_Loss_and_Related_t1,Hearing_Loss_and_Related_t2
SOX11	Skeletal_dysplasia_t2,Proportionate_short_stature_t2,Hereditary_Microcephaly_t2
SOX17	Primary_Pulmonary_Hypertension_and_Related_Disordisorders
SOX2	Skeletal_dysplasia_t2,Proportionate_short_stature_t2,Hearing_loss_t2,Hereditary_retinopathy_t2,Hypogonadotropic_hypogonadism_t2,Hereditary_Autism_t2
SOX3	Skeletal_dysplasia_t2,Disorders_of_sexual_development_t2,Hereditary_retinopathy_t2,Hypogonadotropic_hypogonadism_t2,Hereditary_Autism_t2,Proportionate_short_stature_t1,Disorders_of_sex_development
SOX5	Hereditary_Autism_t2,Optic_neuropathy_and_Related
SOX9	Skeletal_dysplasia_t2,Proportionate_short_stature_t2,Disorders_of_sexual_development_t2,Disorders_of_sex_development,Skeletal_Dysplasia_Panel
SP110	Primary_immune_deficiency_t2
SP7	Skeletal_dysplasia_t2,Osteogenesis_imperfecta
SPAG1	Hereditary_Primary_Ciliary_Dyskinesia_t1
SPARC	Skeletal_dysplasia_t2,Osteogenesis_imperfecta,corneal_keratoconus
SPART	Ataxia_t2,Neurodegenerative_disease_t2,Amyotrophic_lateral_sclerosis_t2,Hereditary_spastic_paraplegia_t1,Amyotrophic_lateral_sclerosis,Hereditary_spastic_paraplegia_panel,Spastic_Paraplegia_and_Related

Supplementary Table 2. List of pathogenic or likely-pathogenic germline variants

Gene	Associated diseases
SPAST	Ataxia_t2,Hereditary_Autism_t2,Neurodegenerative_disease_t2,Amyotrophic_lateral_sclerosis_t2,Hereditary_spastic_paraplegia_t1,Ataxia,Hereditary_spastic_paraplegia_panel,Spastic_Paraplegia_and_Related
SPATA5	Epilepsy_t2,Hearing_loss_t2,Hereditary_Microcephaly_t2,Hereditary_Autism_t2
SPATA7	Hereditary_retinopathy_t2,Retinitis_pigmentosa_t1,Leber_congenital_amaurosis,Retinitis_pigmentosa,Leber's_Congenital_Amaurosis_and_Related,Retinitis_Pigmentosa_and_Related,retina
SPEG	Cardiomyopathy_t2,Muscular_dystrophy_t2,Myopathy_t2
SPEN	Lymphoma_t2
SPG11	Ataxia_t2,Neurodegenerative_disease_t2,Charcot-Marie-Tooth_disease_t1,Hereditary_spastic_paraplegia_t1,Amyotrophic_lateral_sclerosis_t1,Amyotrophic_lateral_sclerosis,Ataxia,Charcot-Marie-Tooth,Hereditary_spastic_paraplegia_panel,Parkinson,Charcot_Marie_Tooth_and_Related,Spastic_Paraplegia_and_Related,CODA
SPG21	Ataxia_t2,Hereditary_spastic_paraplegia_t2,Neurodegenerative_disease_t2,Hereditary_spastic_paraplegia_panel
SPG7	Hereditary_retinopathy_t2,Neurodegenerative_disease_t2,Ataxia_t1,Hereditary_spastic_paraplegia_t1,Ataxia,Hereditary_spastic_paraplegia_panel,Ataxia_and_Related,Spastic_Paraplegia_and_Related
SPINK1	Hereditary_cancer_syndrome_Plus_t2,Hereditary_pancancer
SPINK5	Dermatology_t2,Primary_immune_deficiency_t2,Congenital_diarrhea,Severe_Combined_Immunodeficiency_and_CID
SPINT2	Cholestasis_t2,Congenital_diarrhea
SPNS2	Hereditary_hearing_loss
SPP2	Hereditary_retinopathy_t2,Retinitis_pigmentosa_t1,Retinitis_pigmentosa,Retinitis_Pigmentosa_and_Related,retina
SPR	Ataxia_t2,Hereditary_spastic_paraplegia_t2,Inborn_error_of_metabolism_t2,Dystonia_t2,Dementia_t2,Parkinson's_disease_t2,Hereditary_Autism_t2,Neurodegenerative_disease_t2,Dyskinesia-dystonia-paralysis,Dystonia
SPRED1	Cardiomyopathy_t2,Dermatology_t2,Epilepsy_t2,Hereditary_Autism_t2,Rasopathies_t1,Noonan_syndrome,Noonan_Syndrome_and_Related
SPRY4	Hypogonadotropic_hypogonadism_t1
SPTA1	Anemia_t1,Hemolytic_anemia,Anemia
SPTAN1	Hereditary_Autism_t2,Epilepsy_t1,Early_onset_epilepsy
SPTB	Anemia_t1,Hemolytic_anemia,Anemia
SPTBN2	Ataxia_t1,Ataxia_and_Related
SPTBN4	Muscular_dystrophy_t2,Myopathy_t2,Charcot-Marie-Tooth_disease_t2,Epilepsy_t2
SPTLC1	Charcot-Marie-Tooth_disease_t1,Charcot-Marie-Tooth,Charcot_Marie_Tooth_and_Related
SPTLC2	Charcot-Marie-Tooth_disease_t1,Charcot_Marie_Tooth_and_Related
SPTLC3	Charcot_Marie_Tooth_and_Related
SQSTM1	Skeletal_dysplasia_t2,Muscular_dystrophy_t2,Myopathy_t2,Dementia_t1,Neurodegenerative_disease_t1,Amyotrophic_lateral_sclerosis_t1,Amyotrophic_lateral_sclerosis,Dementia_and_Related
SRA1	Hypogonadotropic_hypogonadism_t2
SRC	Coagulation_t2,Bleeding_Platelet_Disorder
SRCAP	Skeletal_dysplasia_t2,Proportionate_short_stature_t2,Polycystic_Kidney_Disease_t2
SRD5A1	Androgenetic_alopecia
SRD5A2	Disorders_of_sexual_development_t1,Androgenetic_alopecia,Disorders_of_sex_development
SRD5A3	Hereditary_retinopathy_t2,Hereditary_Autism_t2
SRGAP2	Epilepsy_t2
SRP54	Coagulation_t2,Congenital_hematologic_disease,Severe_congenital_neutropenia,Shwachman-Diamond_syndrome,Skeletal_Dysplasia_Panel
SRP72	Coagulation_t2,Bone_Marrow_Failure_Germline_Predisposition,Hereditary_Myeloid_Leukemia_Panel
SRPX2	Epilepsy_t2,Hereditary_Autism_t2
SRSF2	Hematologic_malignancy,ALL_and_others_t1,ALL_and_others_t2,AML,MDS_MPN
SRY	Disorders_of_sexual_development_t1,Disorders_of_sex_development
SSPN	Androgenetic_alopecia
ST14	Dermatology_t2

Supplementary Table 2. List of pathogenic or likely-pathogenic germline variants

Gene	Associated diseases
ST3GAL3	Epilepsy_t2,Hereditary_Autism_t2
ST3GAL5	Epilepsy_t2
ST7	Hereditary_Autism_t2
STAC3	Muscular_dystrophy_t2,Myopathy_t2
STAG2	Skeletal_dysplasia_t2,Hearing_loss_t2,Hereditary_Microcephaly_t2,Cell-free_cancer,Hematologic_malignancy,ALL_and_others_t1,ALL_and_others_t2,AML,MDS_MPN
STAMBP	Skeletal_dysplasia_t2,Rasopathies_t2,Hereditary_Microcephaly_t2
STAP1	Hereditary_Dyslipidemia_t1,Dyslipidemia_and_Related
STAR	Disorders_of_sexual_development_t1,Disorders_of_sex_development
STAT1	Primary_immune_deficiency_t2,Inflammatory_Bowel_t1,Very-early-onset_inflammatory_bowel_disease,Phagocyte_Defect_and_Infectious_disease
STAT2	Primary_immune_deficiency_t2,Phagocyte_Defect_and_Infectious_disease
STAT3	Dermatology_t2,Primary_immune_deficiency_t1,Inflammatory_Bowel_t1,Lymphoma_t1,Hematologic_malignancy,Primary_immunodeficiency,Phagocyte_Defect_and_Infectious_disease,Severe_Combined_Immunodeficiency_and_CID,ALL_and_others_t1,ALL_and_others_t2,Autoimmunity_and_autoinflammatory_disorders,B_cell_and_Humoral_Immune_Deficiency,Hemophagocytic_Lymphohistiocytosis_and_Related,Lymphoma_T_NK_cell_t1,Lymphoma_T_NK_cell_t2,MDS_MPN,Myeloma_t1
STAT5B	Skeletal_dysplasia_t2,Proportionate_short_stature_t2,Primary_immune_deficiency_t2,Hereditary_Autism_t2,Lymphoma_t1,Hematologic_malignancy,Autoimmunity_and_autoinflammatory_disorders,Lymphoma_T_NK_cell_t1,Lymphoma_T_NK_cell_t2,Severe_Combined_Immunodeficiency_and_CID
STAT6	Lymphoma_B_cell_t1,Lymphoma_B_cell_t2,Lymphoma_T_NK_cell_t2
STEAP3	Anemia,Hemochromatosis
STIL	Epilepsy_t2,Hereditary_Microcephaly_t1
STIM1	Muscular_dystrophy_t2,Myopathy_t2,Coagulation_t2,Primary_immune_deficiency_t2,Myofibrillar_myopathy,Autoimmunity_and_autoinflammatory_disorders,Severe_Combined_Immunodeficiency_and_CID
STING1	Autoinflammatory_disorders
STK11	Hereditary_cancer_syndrome_Plus_t1,Cell-free_cancer,Hereditary_breast_cancer,Hereditary_colon_cancer,Hereditary_pancancer,ALL_and_others_t2,Breast_and_Ovarian_Cancer_Panel,Colorectal_Cancer_Polyposis_Panel
STK24	corneal_keratoconus
STK3	Hereditary_Autism_t2
STK36	Hereditary_Primary_Ciliary_Dyskinesia_t2
STK4	Primary_immune_deficiency_t2,Congenital_hematologic_disease,Severe_congenital_neutropenia,Severe_Combined_Immunodeficiency_and_CID
STN1	Congenital_hematologic_disease,Dyskeratosis_congenita
STOM	Hemolytic_anemia
STRA6	Hereditary_retinopathy_t2,Hereditary_Autism_t2
STRADA	Epilepsy_t2
STRC	Hearing_loss_t2,Hereditary_hearing_loss
STS	Dermatology_t2,corneal_dystrophy
STUB1	Ataxia_t2,Hypogonadotropic_hypogonadism_t2
STX11	Hereditary_Autism_t2,Primary_immune_deficiency_t1,Familial_hemophagocytic_lymphohistiocytosis,Hemophagocytic_Lymphohistiocytosis_and_Related
STX1B	Epilepsy_t2
STX3	Congenital_diarrhea
STXBP1	Hereditary_Autism_t2,Epilepsy_t1,Early_onset_epilepsy,Epilepsy_and_Related
STXBP2	Primary_immune_deficiency_t1,Inflammatory_Bowel_t1,Familial_hemophagocytic_lymphohistiocytosis,Very-early-onset_inflammatory_bowel_disease,Hemophagocytic_Lymphohistiocytosis_and_Related
SUCLA2	Myopathy_t2,Ataxia_t2,Hearing_loss_t2,Leigh_Syndrome_and_Related
SUCLG1	Myopathy_t2,Hearing_loss_t2,Hereditary_Autism_t2,Leigh_Syndrome_and_Related

Supplementary Table 2. List of pathogenic or likely-pathogenic germline variants

Gene	Associated diseases
SUCNR1	Androgenetic_alopecia
SUFU	Dermatology_t2,Hereditary_retinopathy_t2,Hereditary_pancancer,Germline_Cancer
SULF1	Skeletal_dysplasia_t2
SUMF1	Dermatology_t2,Lysosomal_storage_disease_t2,Epilepsy_t2,Skeletal_Dysplasia_Panel
SUN1	Muscular_dystrophy_t2,Myopathy_t2
SUN2	Muscular_dystrophy_t2,Myopathy_t2
SUOX	Lysosomal_storage_disease_t2,Epilepsy_t2
SURF1	Charcot-Marie-Tooth_disease_t2,Epilepsy_t2,Charcot_Marie_Tooth_and_Related,Leigh_Syndrome_and_Related,Optic_neuropathy_and_Related
SYK	Lymphoma_B_cell_t2,Lymphoma_T_NK_cell_t2
SYN1	Epilepsy_t2,Hereditary_Autism_t2
SYNE1	Muscular_dystrophy_t2,Myopathy_t2,Ataxia_t1,Ataxia,Limb_girdle_muscular_dystrophy,Ataxia_and_Related
SYNE2	Myopathy_t2,Muscular_dystrophy_t1,Limb_girdle_muscular_dystrophy,Muscular_Dystrophy_and_Related
SYNE4	Hearing_loss_t2,Hereditary_hearing_loss
SYNGAP1	Rasopathies_t2,Epilepsy_t2,Hereditary_Autism_t2,Early_onset_epilepsy,Epilepsy_and_Related
SYNJ1	Epilepsy_t2,Dystonia_t2,Parkinson's_disease_t2,Parkinson
SYP	Epilepsy_t2,Hereditary_Autism_t2
SYT14	Ataxia_t2,Hereditary_Autism_t2
SYT2	Hearing_loss_t2
SYT9	Maturity-Onset_Diabetes_of_the_Young,_MODY_t1
SZT2	Epilepsy_t2,Epilepsy_and_Related
TAB2	Cardiomyopathy_t2,Connective_tissue_disorder_t2,Skeletal_Dysplasia_Panel
TAC3	Disorders_of_sexual_development_t1,Hypogonadotropic_hypogonadism_t1,Isolated_Hypogonadotropic_Hypogonadism
TACO1	Epilepsy_t2,Leigh_Syndrome_and_Related,Optic_neuropathy_and_Related
TACR3	Disorders_of_sexual_development_t1,Hypogonadotropic_hypogonadism_t1,Isolated_Hypogonadotropic_Hypogonadism
TACSTD2	Hereditary_retinopathy_t2,Corneal_Dystrophy_and_Related,corneal_dystrophy
TAF1	Epilepsy_t2,Alzheimer's_disease_t2,Dementia_t2,Parkinson's_disease_t2,Hereditary_Autism_t2,Neurodegenerative_disease_t2,Dystonia_t1,Dyskinesia-dystonia-paralysis,Dystonia,Parkinson,Dystonia_and_Related
TAF15	Neurodegenerative_disease_t2,Amyotrophic_lateral_sclerosis_t1,Amyotrophic_lateral_sclerosis
TAF6	Hereditary_Autism_t2
TAFAZZIN	Cardiomyopathy_t2,Inborn_error_of_metabolism_t2,Congenital_hematologic_disease,Dilated_cardiomyopathy,Severe_congenital_neutropenia,Bone_Marrow_Failure_Germline_Predisposition
TALDO1	Skeletal_dysplasia_t2,Proportionate_short_stature_t2,Anemia
TANGO2	Myopathy_t2
TAP1	Primary_immune_deficiency_t2,Severe_Combined_Immunodeficiency_and_CID
TAP2	Primary_immune_deficiency_t2,Severe_Combined_Immunodeficiency_and_CID
TAPBP	Primary_immune_deficiency_t2,Severe_Combined_Immunodeficiency_and_CID
TAPT1	Skeletal_dysplasia_t2
TARDBP	Dementia_t1,Neurodegenerative_disease_t1,Amyotrophic_lateral_sclerosis_t1,Amyotrophic_lateral_sclerosis,Androgenetic_alopecia,Dementia_and_Related
TAT	Inborn_error_of_metabolism_t2
TBC1D20	Epilepsy_t2,Hypogonadotropic_hypogonadism_t2,Hereditary_Microcephaly_t2
TBC1D23	Hereditary_Microcephaly_t2
TBC1D24	Epilepsy_t2,Hearing_loss_t2,Hereditary_Autism_t2,Focal_epilepsy,Hereditary_hearing_loss,Epilepsy_and_Related,Hearing_Loss_and_Related_t2
TBCD	Epilepsy_t2,Optic_neuropathy_and_Related

Supplementary Table 2. List of pathogenic or likely-pathogenic germline variants

Gene	Associated diseases
TBCE	Skeletal_dysplasia_t2,Epilepsy_t2,Hereditary_Autism_t2,Rickets_Hypoparathyroidism_panel
TBCK	Epilepsy_t2
TBK1	Hereditary_retinopathy_t2,Dementia_t1,Neurodegenerative_disease_t1,Amyotrophic_lateral_sclerosis_t1,Amyotrophic_lateral_sclerosis,Dementia_and_Related,Phagocyte_Defect_and_Infectious_disease
TBL1X	Hypothyroidism_t2,Hearing_loss_t2
TBL1XR1	Epilepsy_t2,Hereditary_Autism_t2,Lymphoma_t1,Lymphoma_B_cell_t2,Lymphoma_T_NK_cell_t2
TBP	Chorea
TBR1	Hereditary_Autism_t2
TBX1	Epilepsy_t2,Primary_immune_deficiency_t2,Hearing_loss_t2,Hereditary_Autism_t2,Severe_Combined_Immunodeficiency_and_CID
TBX15	Skeletal_dysplasia_t2
TBX19	Skeletal_dysplasia_t2,Proportionate_short_stature_t2
TBX2	Skeletal_dysplasia_t2,Proportionate_short_stature_t2
TBX20	Cardiomyopathy_t2
TBX3	Skeletal_dysplasia_t2,Proportionate_short_stature_t2
TBX4	Skeletal_dysplasia_t2,Primary_Pulmonary_Hypertension_and_Related_Disordisorders
TBX5	Arrhythmia_t2,Cardiomyopathy_t2,Skeletal_dysplasia_t2
TBX6	Skeletal_dysplasia_t2
TBXA2R	Coagulation_t2,Bleeding_Platelet_Disorder
TBXAS1	Skeletal_dysplasia_t2,Coagulation_t2,Sclerosing_Bone_Disease_Panel,Bleeding_Platelet_Disorder
TCAP	Myopathy_t2,Cardiomyopathy_t1,Muscular_dystrophy_t1,Congenital_muscular_dystrophy,Dilated_cardiomyopathy,Hypertrophic_cardiomyopathy,Limb_girdle_muscular_dystrophy,Myofibrillar_myopathy,Cardiomyopathy_and_Related,Muscular_Dystrophy_and_Related,Sudden_Cardiac_Arrest_and_Related
TCF12	Skeletal_dysplasia_t2,Craniosynostosis
TCF20	Hereditary_Autism_t2
TCF3	Lymphoma_t1,B_cell_and_Humoral_Immune_Deficiency,Lymphoma_B_cell_t1,Lymphoma_B_cell_t2
TCF4	Lysosomal_storage_disease_t2,Epilepsy_t2,Hereditary_retinopathy_t2,Hereditary_Microcephaly_t2,Hereditary_Autism_t1,Hirschsprung's_disease,Corneal_Dystrophy_and_Related,corneal_dystrophy
TCF7L2	Cell-free_cancer
TCIRG1	Skeletal_dysplasia_t2,Congenital_hematologic_disease,Sclerosing_Bone_Disease_Panel,Severe_congenital_neutropenia
TCN2	Inborn_error_of_metabolism_t2,Primary_immune_deficiency_t2,Anemia
TCOF1	Skeletal_dysplasia_t2,Hearing_loss_t2,Hereditary_hearing_loss
TCTN1	Ataxia_t2,Hereditary_retinopathy_t2,Nephronophthisis_t1,Nephronophthisis_and_Related
TCTN2	Ataxia_t2,Hereditary_retinopathy_t1
TCTN3	Skeletal_dysplasia_t2,Ataxia_t2,Hereditary_retinopathy_t2,Skeletal_Dysplasia_Panel
TDP1	Ataxia_t2,Charcot_Marie_Tooth_and_Related
TDRD7	Hereditary_retinopathy_t2
TEAD1	Hereditary_retinopathy_t2,retina
TECPR2	Ataxia_t2,Neurodegenerative_disease_t2,Hereditary_spastic_paraplegia_t1,Charcot_Marie_Tooth_and_Related,Spastic_Paraplegia_and_Related
TECR	Hereditary_Autism_t2
TECRL	Arrhythmia_t2
TECTA	Hearing_loss_t1,Hereditary_hearing_loss,Hearing_Loss_and_Related_t1,Hearing_Loss_and_Related_t2
TEK	Hereditary_retinopathy_t2,glaucoma
TEKT3	Charcot_Marie_Tooth_and_Related
TENM3	Hereditary_retinopathy_t2
TENT5A	Skeletal_dysplasia_t2,Osteogenesis_imperfecta

Supplementary Table 2. List of pathogenic or likely-pathogenic germline variants

Gene	Associated diseases
TENT5C	Myeloma_t1
TERC	Dermatology_t2,Coagulation_t2,Primary_immune_deficiency_t2,Congenital_hematologic_disease,Dyskeratosis_congenita,Hereditary_pancancer,Bone_Marrow_Failure_Germline_Predisposition
TERT	Dermatology_t2,Coagulation_t2,Primary_immune_deficiency_t2,Cell-free_cancer,Congenital_diarrhea,Congenital_hematologic_disease,Dyskeratosis_congenita,Hereditary_pancancer,AML,Bone_Marrow_Failure_Germline_Predisposition,Lymphoma_B_cell_t2,Lymphoma_T_NK_cell_t2,MDS_MPN
TET2	Lymphoma_t1,Hematologic_malignancy,ALL_and_others_t1,ALL_and_others_t2,AML,Erythrocytosis,Lymphoma_T_NK_cell_t1,Lymphoma_T_NK_cell_t2,MDS_MPN,Myeloma_t1
TET3	Hereditary_Autism_t2,Erythrocytosis
TF	Anemia,Hemochromatosis
TFAP2A	Hearing_loss_t2,glaucoma
TFG	Charcot-Marie-Tooth_disease_t2,Hereditary_spastic_paraplegia_t2,Neurodegenerative_disease_t2,Amyotrophic_lateral_sclerosis_t2,Charcot_Marie_Tooth_and_Related
TFPI	Atypical_Hemolytic_Uremic_Syndrome,Bleeding_Coagulopathy,Thrombosis
TFR2	Anemia,Hemochromatosis
TFRC	Primary_immune_deficiency_t2
TG	Hypothyroidism_t1,Congenital_Hypothyroidism
TGFB1	Skeletal_dysplasia_t1,Sclerosing_Bone_Disease_Panel,Stroke,Skeletal_Dysplasia_and_Related,corneal_keratoconus
TGFB2	Dermatology_t2,Skeletal_dysplasia_t2,Connective_tissue_disorder_t1,Familial_thoracic_aortic_aneurysms_and_aortic_dissections,Overgrowth_Panel,Stroke,Connective_Tissue_Disorder_and_Related,Marfan_and_Related
TGFB3	Arrhythmia_t2,Cardiomyopathy_t2,Skeletal_dysplasia_t2,Connective_tissue_disorder_t1,Arrhythmia,Familial_thoracic_aortic_aneurysms_and_aortic_dissections,Overgrowth_Panel,Stroke,Connective_Tissue_Disorder_and_Related,Marfan_and_Related
TGFBI	Hereditary_retinopathy_t2,Corneal_Dystrophy_and_Related,corneal_dystrophy,corneal_keratoconus
TGFBR1	Dermatology_t2,Skeletal_dysplasia_t2,Inflammatory_Bowel_t2,Connective_tissue_disorder_t1,Craniosynostosis,Familial_thoracic_aortic_aneurysms_and_aortic_dissections,Overgrowth_Panel,Stroke,Connective_Tissue_Disorder_and_Related,Marfan_and_Related
TGFBR2	Dermatology_t2,Skeletal_dysplasia_t2,Inflammatory_Bowel_t2,Connective_tissue_disorder_t1,Craniosynostosis,Familial_thoracic_aortic_aneurysms_and_aortic_dissections,Overgrowth_Panel,Stroke,Connective_Tissue_Disorder_and_Related,Marfan_and_Related
TGIF1	Hereditary_Autism_t2
TGM1	Dermatology_t1
TGM5	Dermatology_t1
TGM6	Ataxia_t2
TH	Hereditary_spastic_paraplegia_t2,Inborn_error_of_metabolism_t2,Alzheimer's_disease_t2,Dementia_t2,Hereditary_Autism_t2,Dystonia_t1,Parkinson's_disease_t1,Neurodegenerative_disease_t1,Dyskinesia-dystonia-paralysis,Dystonia,Dystonia_and_Related
THAP1	Parkinson's_disease_t2,Neurodegenerative_disease_t2,Dystonia_t1,Dyskinesia-dystonia-paralysis,Dystonia,Dystonia_and_Related
THBD	Coagulation_t2,Atypical_Hemolytic_Uremic_Syndrome_t1,DNAJC21_EFL1_SBDS_SRP54,Atypical_Hemolytic_Uremic_Syndrome,Bleeding_Coagulopathy,Thrombosis
THOC2	Hereditary_Autism_t2
THOC6	Hereditary_Microcephaly_t2
THPO	Skeletal_dysplasia_t2,Coagulation_t2,Bleeding_Platelet_Disorder
THRA	Hypothyroidism_t1,Congenital_Hypothyroidism
THRB	Hereditary_Autism_t2,Hypothyroidism_t1,Congenital_Hypothyroidism
TIA1	Muscular_dystrophy_t2,Myopathy_t2,Neurodegenerative_disease_t2,Amyotrophic_lateral_sclerosis_t2,Myofibrillar_myopathy
TICAM1	Primary_immune_deficiency_t2,Phagocyte_Defect_and_Infectious_disease
TIMM8A	Hearing_loss_t2,Hereditary_retinopathy_t2,Dystonia_t2,Parkinson's_disease_t2,Hereditary_Autism_t2,Dementia_t1,Neurodegenerative_disease_t1,Dementia_and_Related,Optic_neuropathy_and_Related
TIMP1	corneal_keratoconus
TIMP3	Hereditary_retinopathy_t2,Macular_dystrophy_and_Related,Retinitis_Pigmentosa_and_Related,corneal_keratoconus,retina

Supplementary Table 2. List of pathogenic or likely-pathogenic germline variants

Gene	Associated diseases
TINF2	Dermatology_t2,Coagulation_t2,Primary_immune_deficiency_t2,Hereditary_Autism_t2,Congenital_hematologic_disease,Dyskeratosis_congenita,Bone_Marrow_Failure_Germline_Predisposition
TIRAP	Phagocyte_Defect_and_Infectious_disease
TJP2	Skeletal_dysplasia_t2,Hearing_loss_t2,Cholestasis_t1,Hereditary_hearing_loss,Neonatal_cholestasis
TK2	Myopathy_t2,Ataxia_t2,Ataxia
TLR3	Primary_immune_deficiency_t2,Phagocyte_Defect_and_Infectious_disease
TLR4	Hereditary_retinopathy_t2
TMC1	Hearing_loss_t1,Hereditary_hearing_loss,Hearing_Loss_and_Related_t1,Hearing_Loss_and_Related_t2
TMC6	Primary_immune_deficiency_t2
TMC8	Primary_immune_deficiency_t2
TMCO1	Hereditary_Autism_t2
TMEM107	Hereditary_retinopathy_t2
TMEM126A	Hereditary_retinopathy_t2,Dominant_optic_atrophy,Optic_neuropathy_and_Related
TMEM126B	Muscular_dystrophy_t2,Myopathy_t2
TMEM127	Hereditary_cancer_syndrome_Plus_t2,Neuroendocrine_tumor_t1,Hereditary_pancancer,Hereditary_paraganglioma-pheochromocytoma_syndrome,Pheochromocytoma-Paraganglioma_Panel
TMEM132E	Hearing_loss_t2,Hereditary_hearing_loss
TMEM138	Ataxia_t2,Hereditary_retinopathy_t2,Nephronophthisis_t1,Nephronophthisis_and_Related
TMEM165	Hereditary_Autism_t2,Skeletal_Dysplasia_Panel
TMEM216	Skeletal_dysplasia_t2,Ataxia_t2,Hereditary_Autism_t2,Cholestasis_t2,Hereditary_retinopathy_t1,Nephronophthisis_t1,Nephronophthisis_and_Related,retina
TMEM230	Parkinson's_disease_t2
TMEM231	Ataxia_t2,Hereditary_retinopathy_t2,Hereditary_Autism_t2
TMEM237	Ataxia_t2,Hereditary_retinopathy_t2,Nephronophthisis_t1,Nephronophthisis_and_Related,retina
TMEM240	Ataxia_t1,Ataxia_and_Related
TMEM38B	Skeletal_dysplasia_t2,Osteogenesis_imperfecta
TMEM43	Arrhythmia_t2,Cardiomyopathy_t2,Muscular_dystrophy_t2,Myopathy_t2,Arrhythmia,Hereditary_hearing_loss,Limb_girdle_muscular_dystrophy,Arrhythmia_and_Related
TMEM67	Skeletal_dysplasia_t2,Ataxia_t2,Epilepsy_t2,Polycystic_Kidney_Disease_t2,Hereditary_Autism_t2,Hereditary_retinopathy_t1,Nephronophthisis_t1,Nephronophthisis_and_Related
TMEM70	Cardiomyopathy_t2,Epilepsy_t2,Hereditary_Autism_t2
TMEM98	Hereditary_retinopathy_t2
TMIE	Hearing_loss_t1,Hereditary_hearing_loss,Hearing_Loss_and_Related_t1,Hearing_Loss_and_Related_t2
TMOD1	Hemolytic_anemia
TMPO	Cardiomyopathy_t1,Dilated_cardiomyopathy
TMPRSS3	Hearing_loss_t1,Hereditary_hearing_loss,Hearing_Loss_and_Related_t1,Hearing_Loss_and_Related_t2
TMPRSS5	Hearing_loss_t2
TMPRSS6	Anemia_t1,Anemia
TMTC3	Hereditary_Microcephaly_t2
TNC	Hearing_loss_t2,Hereditary_hearing_loss
TNFAIP3	Lymphoma_t1,Autoinflammatory_disorders,Hematologic_malignancy,Autoimmunity_and_autoinflammatory_disorders,B_cell_and_Humoral_Immune_Deficiency
TNFRSF11A	Skeletal_dysplasia_t2,Sclerosing_Bone_Disease_Panel
TNFRSF11B	Skeletal_dysplasia_t2,Sclerosing_Bone_Disease_Panel
TNFRSF13B	Primary_immune_deficiency_t2,B_cell_and_Humoral_Immune_Deficiency,Autoimmunity_and_autoinflammatory_disorders
TNFRSF13C	Primary_immune_deficiency_t2,B_cell_and_Humoral_Immune_Deficiency,Autoimmunity_and_autoinflammatory_disorders
TNFRSF14	Lymphoma_t1,Hematologic_malignancy,Lymphoma_B_cell_t1,Lymphoma_B_cell_t2
TNFRSF1A	Autoinflammatory_disorders,Autoimmunity_and_autoinflammatory_disorders

Supplementary Table 2. List of pathogenic or likely-pathogenic germline variants

Gene	Associated diseases
TNFRSF1B	Lymphoma_T_NK_cell_t1,Lymphoma_T_NK_cell_t2
TNFRSF4	Primary_immune_deficiency_t2
TNFSF11	Skeletal_dysplasia_t2,Sclerosing_Bone_Disease_Panel
TNFSF12	B_cell_and_Humoral_Immune_Deficiency
TNK2	Epilepsy_t2
TNNC1	Cardiomyopathy_t1,Dilated_cardiomyopathy,Hypertrophic_cardiomyopathy,Cardiomyopathy_and_Related
TNNC2	Dilated_cardiomyopathy,Hypertrophic_cardiomyopathy
TNNI3	Arrhythmia_t2,Cardiomyopathy_t1,Dilated_cardiomyopathy,Hypertrophic_cardiomyopathy,Cardiomyopathy_and_Related,Sudden_Cardiac_Arrest_and_Related
TNNI3K	Arrhythmia_t2,Cardiomyopathy_t2
TNNT1	Muscular_dystrophy_t2,Myopathy_t1,Congenital_myopathy,Myopathy_and_Related
TNNT2	Arrhythmia_t2,Cardiomyopathy_t1,Dilated_cardiomyopathy,Hypertrophic_cardiomyopathy,Cardiomyopathy_and_Related,Sudden_Cardiac_Arrest_and_Related
TNPO3	Muscular_dystrophy_t2,Myopathy_t2,Limb_girdle_muscular_dystrophy
TNXB	Dermatology_t2,Connective_tissue_disorder_t2
TOE1	Hereditary_Microcephaly_t2
TONSL	Skeletal_dysplasia_t2,Skeletal_Dysplasia_Panel
TOP2A	Cell-free_cancer
TOP3A	Skeletal_dysplasia_t2,Proportionate_short_stature_t2,Hereditary_Microcephaly_t2
TOPORS	Hereditary_retinopathy_t2,Retinitis_pigmentosa_t1,Retinitis_pigmentosa,Retinitis_Pigmentosa_and_Related,retina
TOR1A	Parkinson's_disease_t2,Neurodegenerative_disease_t2,Dystonia_t1,Dyskinesia-dystonia-paralysis,Dystonia,Dystonia_and_Related
TOR1AIP1	Cardiomyopathy_t2,Muscular_dystrophy_t2,Myopathy_t2,Dystonia_t2,Parkinson's_disease_t2,Limb_girdle_muscular_dystrophy
TP53	Dermatology_t2,Hereditary_cancer_syndrome_Plus_t1,Neuroendocrine_tumor_t1,Lymphoma_t1,Cell-free_cancer,Congenital_hematologic_disease,Hematologic_malignancy,Hereditary_breast_cancer,Hereditary_colon_cancer,Hereditary_pancancer,Hereditary_paraganglioma-pheochromocytoma_syndrome,Leukemia_predisposition,ALL_and_others_t1,ALL_and_others_t2,AML,Breast_and_Ovarian_Cancer_Panel,Circulating_tumor_DNA_assay_55,Colorectal_Cancer_Polyposis_Panel,Germline_Cancer,Hereditary_Myeloid_Leukemia_Panel,Lymphoma_B_cell_t1,Lymphoma_B_cell_t2,Lymphoma_T_NK_cell_t1,Lymphoma_T_NK_cell_t2,MDS_MPN,Myeloma_t1
TP63	Dermatology_t2,Skeletal_dysplasia_t2,Lymphoma_t1,Lymphoma_B_cell_t2
TPH2	Hereditary_Autism_t2
TPI1	Anemia_t1,Hemolytic_anemia,Anemia
TPK1	Epilepsy_t2,Dystonia_t2,Parkinson's_disease_t2,Hereditary_Autism_t2
TPM1	Cardiomyopathy_t1,Dilated_cardiomyopathy,Hypertrophic_cardiomyopathy,Cardiomyopathy_and_Related
TPM2	Muscular_dystrophy_t2,Ataxia_t2,Myopathy_t1,Congenital_myopathy,Myopathy_and_Related
TPM3	Muscular_dystrophy_t2,Ataxia_t2,Myopathy_t1,Congenital_myopathy,Hemolytic_anemia,Myopathy_and_Related
TPO	Hypothyroidism_t1,Congenital_Hypothyroidism
TPP1	Ataxia_t2,Lysosomal_storage_disease_t2,Epilepsy_t2,Hereditary_Autism_t2,Hereditary_retinopathy_t1,Epilepsy_and_Related
TPP2	Hemophagocytic_Lymphohistiocytosis_and_Related
TPRN	Hearing_loss_t2,Hereditary_hearing_loss,Hearing_Loss_and_Related_t2
TRAF3	Primary_immune_deficiency_t2,Lymphoma_t1,Hematologic_malignancy,Myeloma_t1,Phagocyte_Defect_and_Infectious_disease
TRAF3IP1	Hereditary_retinopathy_t2,Nephronophthisis_t1,Skeletal_Dysplasia_Panel,Nephronophthisis_and_Related
TRAF3IP2	Primary_immune_deficiency_t2
TRAK1	Epilepsy_t2
TRAPPC11	Muscular_dystrophy_t2,Myopathy_t2,Dystonia_t2,Parkinson's_disease_t2,Congenital_muscular_dystrophy,Limb_girdle_muscular_dystrophy
TRAPPC2	Skeletal_dysplasia_t2,Skeletal_Dysplasia_Panel,Spondyloepiphyseal_metaphyseal_dysplasia
TRAPPC9	Hereditary_Microcephaly_t2,Hereditary_Autism_t2

Supplementary Table 2. List of pathogenic or likely-pathogenic germline variants

Gene	Associated diseases
TRDN	Cardiomyopathy_t2,Arrhythmia_t1,Arrhythmia,Arrhythmia_and_Related
TREM2	Skeletal_dysplasia_t2,Alzheimer's_disease_t2,Parkinson's_disease_t2,Dementia_t1,Neurodegenerative_disease_t1,Amyotrophic_lateral_sclerosis_t1,Dementia_and_Related
TREX1	Epilepsy_t2,Hereditary_retinopathy_t2,Hereditary_Stroke_t1,Autoinflammatory_disorders,Stroke,retina
TRH	Hypothyroidism_t1,Congenital_Hypothyroidism
TRHR	Hereditary_Autism_t2,Hypothyroidism_t1,Congenital_Hypothyroidism
TRIM2	Charcot-Marie-Tooth_disease_t2,Charcot-Marie-Tooth,Charcot_Marie_Tooth_and_Related
TRIM32	Cardiomyopathy_t2,Muscular_dystrophy_t2,Myopathy_t2,Ataxia_t2,Disorders_of_sexual_development_t2,Retinitis_pigmentosa_t2,Hereditary_retinopathy_t2,Hypogonadotropic_hypogonadism_t2,Limb_girdle_muscular_dystrophy,Myofibrillar_myopathy,Retinitis_Pigmentosa_and_Related,retina
TRIM37	Skeletal_dysplasia_t2,Proportionate_short_stature_t2
TRIM44	glaucoma
TRIM63	Cardiomyopathy_t2
TRIM8	Epilepsy_t2
TRIO	Hereditary_Autism_t2
TRIOBP	Hearing_loss_t2,Hereditary_hearing_loss,Hearing_Loss_and_Related_t2
TRIP11	Skeletal_dysplasia_t2,Proportionate_short_stature_t2,Skeletal_Dysplasia_Panel
TRIP12	Hereditary_Autism_t2
TRIP4	Muscular_dystrophy_t2,Myopathy_t2
TRMT10A	Skeletal_dysplasia_t2,Proportionate_short_stature_t2,Hereditary_Microcephaly_t2
TRMU	Hearing_loss_t2,Cholestasis_t2,Neonatal_cholestasis
TRNT1	Hereditary_retinopathy_t2,Retinitis_pigmentosa_t1,Retinitis_pigmentosa,Retinitis_Pigmentosa_and_Related,retina
TRPC6	Glomerulopathy
TRPM1	Hereditary_retinopathy_t2,Congenital_Stationary_Night_Blindness_and_Related,Retinitis_Pigmentosa_and_Related,retina
TRPM4	Arrhythmia_t1,Arrhythmia_and_Related
TRPM6	Atypical_Hemolytic_Uremic_Syndrome_t2,Ca-Pi-Mg,Rickets_Hypoparathyroidism_panel
TRPM7	Neurodegenerative_disease_t2,Amyotrophic_lateral_sclerosis_t2
TRPS1	Proportionate_short_stature_t2,Skeletal_dysplasia_t1,Skeletal_Dysplasia_Panel,Skeletal_Dysplasia_and_Related
TRPV3	Dermatology_t2
TRPV4	Skeletal_dysplasia_t2,Muscular_dystrophy_t2,Myopathy_t2,Charcot-Marie-Tooth_disease_t1,Charcot-Marie-Tooth,Skeletal_Dysplasia_Panel,Spondyloepiphyseal_metaphyseal_dysplasia,Charcot_Marie_Tooth_and_Related
TRPV6	Osteogenesis_imperfecta,Skeletal_Dysplasia_Panel
TSC1	Dermatology_t2,Polycystic_Kidney_Disease_t2,Hereditary_cancer_syndrome_Plus_t2,Epilepsy_t1,Hereditary_Autism_t1,Cell-free_cancer,Focal_epilepsy,Hereditary_pancancer,Autism_and_Related,Epilepsy_and_Related,Lymphoma_B_cell_t2,Lymphoma_T_NK_cell_t2
TSC2	Dermatology_t2,Polycystic_Kidney_Disease_t2,Hereditary_cancer_syndrome_Plus_t2,Epilepsy_t1,Hereditary_Autism_t1,Cell-free_cancer,Focal_epilepsy,Hereditary_pancancer,Autism_and_Related,Epilepsy_and_Related,Lymphoma_B_cell_t2,Lymphoma_T_NK_cell_t2
TSEN15	Hereditary_Microcephaly_t2
TSEN2	Epilepsy_t2,Hereditary_Microcephaly_t2
TSEN34	Epilepsy_t2,Hereditary_Microcephaly_t2
TSEN54	Epilepsy_t2,Hereditary_Microcephaly_t2
TSFM	Cardiomyopathy_t2,Hereditary_retinopathy_t2
TSHB	Hypothyroidism_t1,Congenital_Hypothyroidism
TSHR	Hereditary_Autism_t2,Hypothyroidism_t1,Congenital_Hypothyroidism
TSHZ1	Hearing_loss_t2

Supplementary Table 2. List of pathogenic or likely-pathogenic germline variants

Gene	Associated diseases
TSPAN12	Hereditary_retinopathy_t2,Pediatric_retinal_vascular_disease,Vitreoretinopathy,Vitreoretinopathy_and_Related,retina
TSPAN7	Hereditary_Autism_t2
TSPEAR	Hearing_loss_t2,Hereditary_hearing_loss
TSR2	Congenital_hematologic_disease,Diamond-Blackfan_anemia
TTBK2	Ataxia_t1,Ataxia_and_Related
TTC19	Ataxia_t2,Epilepsy_t2,Leigh_Syndrome_and_Related
TTC21B	Skeletal_dysplasia_t2,Hereditary_retinopathy_t2,Polycystic_Kidney_Disease_t2,Nephronophthisis_t1,Skeletal_Dysplasia_Panel,Spondyloepiphyseal_metaphyseal_dysplasia,Nephronophthisis_and_Related
TTC37	Hereditary_Autism_t2,Cholestasis_t2,Inflammatory_Bowel_t1,Congenital_diarrhea,B_cell_and_Humoral_Immune_Deficiency
TTC7A	Primary_immune_deficiency_t2,Inflammatory_Bowel_t2,Very-early-onset_inflammatory_bowel_disease,Severe_Combined_Immunodeficiency_and_CID
TTC8	Ataxia_t2,Disorders_of_sexual_development_t2,Hereditary_retinopathy_t2,Hypogonadotropic_hypogonadism_t2,Hereditary_Autism_t2,Retinitis_pigmentosa_t1,Retinitis_pigmentosa,Retinitis_Pigmentosa_and_Related,retina
TTLL5	Hereditary_retinopathy_t2,Cone-Rod_Dystrophy_and_Related,Retinitis_Pigmentosa_and_Related,retina
TTN	Arrhythmia_t2,Cardiomyopathy_t1,Muscular_dystrophy_t1,Myopathy_t1,Congenital_myopathy,Dilated_cardiomyopathy,Hypertrophic_cardiomyopathy,Limb_girdle_muscular_dystrophy,Myofibrillar_myopathy,Cardiomyopathy_and_Related,Muscular_Dystrophy_and_Related,Myopathy_and_Related
TTPA	Retinitis_pigmentosa_t2,Hereditary_retinopathy_t2,Ataxia_t1,Ataxia,Ataxia_and_Related,Retinitis_Pigmentosa_and_Related,retina
TTR	Cardiomyopathy_t2,Dermatology_t2,Charcot-Marie-Tooth_disease_t2,Ataxia_t2,Hereditary_spastic_paraplegia_t2,Neurodegenerative_disease_t2,Dilated_cardiomyopathy,Hypertrophic_cardiomyopathy,Charcot_Marie_Tooth_and_Related,Sudden_Cardiac_Arrest_and_Related
TUB	Retinitis_pigmentosa_t2,Hereditary_retinopathy_t2,Retinitis_Pigmentosa_and_Related,retina
TUBA1A	Epilepsy_t2,Hereditary_Microcephaly_t2,Hereditary_Autism_t2,Malformation_of_cortical_development
TUBA4A	Dementia_t2,Amyotrophic_lateral_sclerosis_t2,Alzheimer's_disease_t1,Neurodegenerative_disease_t1,Dementia_and_Related
TUBA8	Epilepsy_t2,Hereditary_Microcephaly_t2,Hereditary_Autism_t2,Malformation_of_cortical_development
TUBB	Hereditary_Microcephaly_t2,Malformation_of_cortical_development
TUBB1	Coagulation_t2,Bleeding_Platelet_Disorder
TUBB2A	Epilepsy_t2,Malformation_of_cortical_development
TUBB2B	Epilepsy_t2,Hereditary_Microcephaly_t2,Hereditary_Autism_t2,Malformation_of_cortical_development
TUBB3	Hypogonadotropic_hypogonadism_t2,Hereditary_Microcephaly_t2,Hereditary_Autism_t2,Malformation_of_cortical_development,Charcot_Marie_Tooth_and_Related
TUBB4A	Ataxia_t2,Epilepsy_t2,Parkinson's_disease_t2,Neurodegenerative_disease_t2,Dystonia_t1,Dyskinesia-dystonia-paralysis,Dystonia_and_Related
TUBB4B	Hearing_loss_t2,Hereditary_retinopathy_t2,retina
TUBG1	Malformation_of_cortical_development
TUBGCP4	Hereditary_retinopathy_t2,Hereditary_Microcephaly_t2,retina
TUBGCP6	Hereditary_retinopathy_t2,Hereditary_Microcephaly_t1,retina
TULP1	Hereditary_retinopathy_t2,Retinitis_pigmentosa_t1,Leber_congenital_amaurosis,Retinitis_pigmentosa,Leber's_Congenital_Amaurosis_and_Related,Nystagmus_and_Related,Optic_neuropathy_and_Related,Retinitis_Pigmentosa_and_Related,retina
TUSC3	Hereditary_Autism_t2
TWIST1	Skeletal_dysplasia_t2,Hereditary_Autism_t2,Androgenetic_alopecia,Craniosynostosis
TWIST2	Androgenetic_alopecia
TWNK	Myopathy_t2,Epilepsy_t2,Hearing_loss_t2,Dystonia_t2,Parkinson's_disease_t2,Ataxia_t1,Ataxia,Hereditary_hearing_loss,Ataxia_and_Related,Charcot_Marie_Tooth_and_Related
TXNRD2	Cardiomyopathy_t2
TYK2	Dermatology_t2,Primary_immune_deficiency_t2,Primary_immunodeficiency,Phagocyte_Defect_and_Infectious_disease,Severe_Combined_Immunodeficiency_and_CID
TYMP	Myopathy_t2,Charcot-Marie-Tooth_disease_t2,Ataxia_t2,Charcot_Marie_Tooth_and_Related

Supplementary Table 2. List of pathogenic or likely-pathogenic germline variants

Gene	Associated diseases
TYR	Dermatology_t2,Hearing_loss_t2,Hereditary_retinopathy_t2,Albinism_and_Related,Optic_neuropathy_and_Related
TYROBP	Skeletal_dysplasia_t2,Alzheimer's_disease_t2,Dementia_t2,Parkinson's_disease_t2,Neurodegenerative_disease_t2
TYRP1	Dermatology_t2,Hereditary_retinopathy_t2,Albinism_and_Related,Optic_neuropathy_and_Related
U2AF1	Cell-free_cancer,Hematologic_malignancy,ALL_and_others_t1,ALL_and_others_t2,AML,MDS_MPN
U2AF2	Hematologic_malignancy
UBA1	Muscular_dystrophy_t2,Myopathy_t2
UBA5	Ataxia_t2,Epilepsy_t2
UBAP1	Hereditary_spastic_paraplegia_t2,Neurodegenerative_disease_t2
UBE2A	Epilepsy_t2,Hereditary_Autism_t2
UBE2T	Dermatology_t2,Coagulation_t2,Congenital_hematologic_disease,Bone_Marrow_Failure_Germline_Predisposition
UBE3A	Epilepsy_t2,Dementia_t2,Hereditary_Microcephaly_t2,Neurodegenerative_disease_t2,Hereditary_Autism_t1,Autism_and_Related
UBE3B	Hereditary_Microcephaly_t2
UBIAD1	Hereditary_retinopathy_t2,Corneal_Dystrophy_and_Related,corneal_dystrophy
UBQLN2	Dementia_t2,Neurodegenerative_disease_t2,Amyotrophic_lateral_sclerosis_t1,Amyotrophic_lateral_sclerosis
UBR1	Dermatology_t2,Skeletal_dysplasia_t2,Congenital_Hypothyroidism
UBR5	Lymphoma_t1
UBTF	Ataxia_t2,Dystonia_t2
UBXN4	corneal_keratoconus
UCHL1	Ataxia_t2,Hereditary_retinopathy_t2,Alzheimer's_disease_t2,Dementia_t2,Parkinson's_disease_t2,Neurodegenerative_disease_t2,Parkinson
UCP2	Maturity-Onset_Diabetes_of_the_Young,_MODY_t2
UFSP2	Skeletal_Dysplasia_Panel
UGT1A1	Cholestasis_t1,Anemia_t1,Cell-free_cancer,Hemolytic_anemia,Anemia
UGT1A6	Hemolytic_anemia
UGT1A7	Hemolytic_anemia
UMOD	Polycystic_Kidney_Disease_t1,Polycystic_kidney_disease_and_Related
UNC119	Primary_immune_deficiency_t2,Hereditary_retinopathy_t2,Cone-Rod_Dystrophy_and_Related,Retinitis_Pigmentosa_and_Related,Severe_Combined_Immunodeficiency_and_CID,retina
UNC13A	Neurodegenerative_disease_t2,Amyotrophic_lateral_sclerosis_t2
UNC13D	Primary_immune_deficiency_t1,Familial_hemophagocytic_lymphohistiocytosis,Hemophagocytic_Lymphohistiocytosis_and_Related
UNC80	Epilepsy_t2,Hereditary_Autism_t2
UNC93B1	Primary_immune_deficiency_t2,Phagocyte_Defect_and_Infectious_disease
UNG	Primary_immune_deficiency_t1,Primary_immunodeficiency,B_cell_and_Humoral_Immune_Deficiency
UPB1	Hereditary_Autism_t2
UPF3B	Connective_tissue_disorder_t2,Hereditary_Autism_t2
UQCRQ	Leigh_Syndrome_and_Related
UROC1	Hereditary_Autism_t2
UROD	Dermatology_t2
UROS	Dermatology_t2
USB1	Dermatology_t2,Bone_Marrow_Failure_Germline_Predisposition
USH1C	Retinitis_pigmentosa_t2,Hearing_loss_t1,Hereditary_retinopathy_t1,Hereditary_hearing_loss,Hearing_Loss_and_Related_t1,Hearing_Loss_and_Related_t2,Retinitis_Pigmentosa_and_Related,retina
USH1G	Hearing_loss_t2,Hereditary_retinopathy_t1,Hereditary_hearing_loss,Hearing_Loss_and_Related_t2,Retinitis_Pigmentosa_and_Related,retina
USH2A	Hereditary_retinopathy_t2,Retinitis_pigmentosa_t1,Hearing_loss_t1,Hereditary_hearing_loss,Retinitis_pigmentosa,Hearing_Loss_and_Related_t1,Hearing_Loss_and_Related_t2,Retinitis_Pigmentosa_and_Related,retina

Supplementary Table 2. List of pathogenic or likely-pathogenic germline variants

Gene	Associated diseases
USP45	retina
USP8	Hereditary_spastic_paraplegia_t2,Neurodegenerative_disease_t2
USP9X	Hereditary_Autism_t2
VAC14	Dystonia_t1,Dystonia_and_Related
VAMP1	Ataxia_t2,Hereditary_spastic_paraplegia_t2,Neurodegenerative_disease_t2
VAMP2	Epilepsy_t2,Hereditary_Autism_t2
VAPB	Neurodegenerative_disease_t2,Amyotrophic_lateral_sclerosis_t1,Amyotrophic_lateral_sclerosis,Charcot_Marie_Tooth_and_Related
VARS1	Epilepsy_t2,Hereditary_Microcephaly_t2
VAV1	Lymphoma_T_NK_cell_t1,Lymphoma_T_NK_cell_t2
VAX1	Hereditary_retinopathy_t2
VCAN	Connective_tissue_disorder_t2,Hearing_loss_t2,Hereditary_retinopathy_t2,Vitreoretinopathy,Vitreoretinopathy_and_Related,retina
VCL	Cardiomyopathy_t1,Dilated_cardiomyopathy,Hypertrophic_cardiomyopathy,Cardiomyopathy_and_Related
VCP	Cardiomyopathy_t2,Muscular_dystrophy_t2,Myopathy_t2,Ataxia_t2,Charcot-Marie-Tooth_disease_t1,Dementia_t1,Neurodegenerative_disease_t1,Amyotrophic_lateral_sclerosis_t1,Amyotrophic_lateral_sclerosis,Charcot-Marie-Tooth,Limb_girdle_muscular_dystrophy,Myofibrillar_myopathy,Charcot_Marie_Tooth_and_Related,Dementia_and_Related
VDR	Skeletal_dysplasia_t2,Ca-Pi-Mg,Rickets_Hypoparathyroidism_panel,Skeletal_Dysplasia_Panel
VEGFA	Neurodegenerative_disease_t2,Amyotrophic_lateral_sclerosis_t2
VHL	Polycystic_Kidney_Disease_t2,Hereditary_cancer_syndrome_Plus_t2,Neuroendocrine_tumor_t1,Cell-free_cancer,Hereditary_pancancer,Hereditary_paraganglioma-pheochromocytoma_syndrome,Erythrocytosis,Germline_Cancer,Pheochromocytoma-Paraganglioma_Panel
VIM	Hereditary_retinopathy_t2
VIPAS39	Skeletal_dysplasia_t2,Coagulation_t2,Cholestasis_t1,Neonatal_cholestasis,Renal_Tubular_Disorders
VKORC1	Coagulation_t2,Atypical_Hemolytic_Uremic_Syndrome,Bleeding_Coagulopathy,Thrombosis
VLDLR	Ataxia_t2,Hereditary_Microcephaly_t2,Hereditary_Autism_t2,Malformation_of_cortical_development
VMA21	Muscular_dystrophy_t2,Myopathy_t2,Myofibrillar_myopathy
VPS13A	Cardiomyopathy_t2,Muscular_dystrophy_t2,Myopathy_t2,Ataxia_t2,Epilepsy_t2,Parkinson's_disease_t2,Neurodegenerative_disease_t2,Dystonia_t1,Chorea,Dystonia_and_Related,Parkinson_Disease_and_Related
VPS13B	Epilepsy_t2,Retinitis_pigmentosa_t2,Hereditary_retinopathy_t2,Hereditary_Microcephaly_t2,Hereditary_Autism_t2,Congenital_hematologic_disease,Severe_congenital_neutropenia,Bone_Marrow_Failure_Germline_Predisposition,Macular_dystrophy_and_Related,Retinitis_Pigmentosa_and_Related
VPS13C	Parkinson's_disease_t2,Parkinson
VPS33A	Skeletal_Dysplasia_Panel
VPS33B	Dermatology_t2,Coagulation_t2,Cholestasis_t1,Neonatal_cholestasis,Renal_Tubular_Disorders
VPS35	Alzheimer's_disease_t2,Dystonia_t2,Dementia_t2,Parkinson's_disease_t2,Neurodegenerative_disease_t2,Parkinson,Parkinson_Disease_and_Related
VPS37A	Ataxia_t2,Hereditary_spastic_paraplegia_t2,Neurodegenerative_disease_t2
VPS45	Congenital_hematologic_disease,Severe_congenital_neutropenia,Bone_Marrow_Failure_Germline_Predisposition
VPS53	Hereditary_Microcephaly_t2
VRK1	Muscular_dystrophy_t2,Myopathy_t2,Ataxia_t2,Hereditary_Microcephaly_t2,Charcot_Marie_Tooth_and_Related
VSX1	Hereditary_retinopathy_t2,Corneal_Dystrophy_and_Related,Keratoconus_and_Related,corneal_dystrophy,corneal_keratoconus
VSX2	Hereditary_retinopathy_t2
VTN	Atypical_Hemolytic_Uremic_Syndrome,Bleeding_Coagulopathy,Thrombosis
VWF	Coagulation_t1,Stroke,Atypical_Hemolytic_Uremic_Syndrome,Bleeding_Coagulopathy,Bleeding_Platelet_Disorder,Thrombosis
WAC	Hereditary_Autism_t2
WARS1	Charcot-Marie-Tooth,Charcot_Marie_Tooth_and_Related

Supplementary Table 2. List of pathogenic or likely-pathogenic germline variants

Gene	Associated diseases
WARS2	Epilepsy_t2
WAS	Dermatology_t2,Coagulation_t2,Primary_immune_deficiency_t2,Inflammatory_Bowel_t1,Congenital_hematologic_disease,Familial_hemophagocytic_lymphohistiocytosis,Primary_immunodeficiency,Severe_congenital_neutropenia,Very-early-onset_inflammatory_bowel_disease,Autoimmunity_and_autoinflammatory_disorders,B_cell_and_Humoral_Immune_Deficiency,Bleeding_Platelet_Disorder,Bone_Marrow_Failure_Germline_Predisposition,Hemophagocytic_Lymphohistiocytosis_and_Related,Hereditary_Myeloid_Leukemia_Panel,Phagocyte_Defect_and_Infectious_disease,Severe_Combined_Immunodeficiency_and_CID
WASF1	Epilepsy_t2,Hereditary_Autism_t2
WASHC5	Ataxia_t2,Hereditary_spastic_paraplegia_t2,Neurodegenerative_disease_t2,Amyotrophic_lateral_sclerosis_t2,Hereditary_spastic_paraplegia_panel
WBP2	Hearing_loss_t2,Hereditary_hearing_loss
WDPCP	Ataxia_t2,Hereditary_retinopathy_t2,Retinitis_Pigmentosa_and_Related,retina
WDR11	Hypogonadotropic_hypogonadism_t1
WDR13	Hereditary_Autism_t2
WDR19	Skeletal_dysplasia_t2,Proportionate_short_stature_t2,Retinitis_pigmentosa_t2,Hereditary_retinopathy_t2,Polycystic_Kidney_Disease_t2,Nephronophthisis_t1,Craniosynostosis,Skeletal_Dysplasia_Panel,Spondyloepiphyseal_metaphyseal_dysplasia,Nephronophthisis_and_Related,Retinitis_Pigmentosa_and_Related,retina
WDR26	Epilepsy_t2
WDR35	Dermatology_t2,Skeletal_dysplasia_t2,Proportionate_short_stature_t2,Nephronophthisis_t1,Craniosynostosis,Skeletal_Dysplasia_Panel,Nephronophthisis_and_Related
WDR36	Hereditary_retinopathy_t2
WDR45	Epilepsy_t2,Dystonia_t2,Parkinson's_disease_t2,Hereditary_Autism_t2,Chorea,Dystonia,Neurodegeneration_with_brain_iron_accumulation,Autism_and_Related,Parkinson_Disease_and_Related
WDR48	Hereditary_spastic_paraplegia_t2,Neurodegenerative_disease_t2
WDR62	Hereditary_Autism_t2,Hereditary_Microcephaly_t1,Malformation_of_cortical_development
WDR73	Ataxia_t2,Hereditary_Microcephaly_t2,Hereditary_Autism_t2,Glomerulopathy
WDR81	Ataxia_t2,Hereditary_Autism_t2
WFS1	Retinitis_pigmentosa_t2,Hereditary_retinopathy_t2,Ataxia_t1,Hearing_loss_t1,Maturity-Onset_Diabetes_of_the_Young,_MODY_t1,Ataxia,Hereditary_hearing_loss,Monogenic_diabetes_panel,Ataxia_and_Related,Hearing_Loss_and_Related_t1,Hearing_Loss_and_Related_t2,Optic_neuropathy_and_Related,Retinitis_Pigmentosa_and_Related,retina
WHRN	Retinitis_pigmentosa_t2,Hearing_loss_t2,Hereditary_retinopathy_t1,Hereditary_hearing_loss,Hearing_Loss_and_Related_t2,Retinitis_Pigmentosa_and_Related,retina
WIPF1	Coagulation_t2,Primary_immune_deficiency_t2,Bleeding_Platelet_Disorder,Bone_Marrow_Failure_Germline_Predisposition
WNK1	Ataxia_t2,Charcot-Marie-Tooth_disease_t1,Acid-base-electro_gene,Charcot_Marie_Tooth_and_Related
WNK4	Acid-base-electro_gene
WNT1	Skeletal_dysplasia_t2,Osteogenesis_imperfecta
WNT10A	Dermatology_t2,Androgenetic_alopecia,Ectodermal_dysplasia_Hypodontia,corneal_keratoconus
WNT10B	Skeletal_dysplasia_t2
WNT3	Skeletal_dysplasia_t2
WNT4	Disorders_of_sexual_development_t2,Disorders_of_sex_development
WNT5A	Skeletal_dysplasia_t2,Proportionate_short_stature_t2,Skeletal_Dysplasia_Panel
WNT7A	Skeletal_dysplasia_t2
WRAP53	Dermatology_t2,Congenital_hematologic_disease,Dyskeratosis_congenita,Bone_Marrow_Failure_Germline_Predisposition
WRN	Dermatology_t2,Hereditary_pancancer
WT1	Disorders_of_sexual_development_t2,Hereditary_cancer_syndrome_Plus_t2,Disorders_of_sex_development,Glomerulopathy,Hematologic_malignancy,Hereditary_pancancer,ALL_and_others_t1,ALL_and_others_t2,AML,Germline_Cancer,Lymphoma_T_NK_cell_t2,MDS_MPN
WWOX	Ataxia_t2,Disorders_of_sexual_development_t2,Epilepsy_t2,Disorders_of_sex_development
XBP1	Myeloma_t1

Supplementary Table 2. List of pathogenic or likely-pathogenic germline variants

Gene	Associated diseases
XIAP	Primary_immune_deficiency_t1,Inflammatory_Bowel_t1,Familial_hemophagocytic_lymphohistiocytosis,Very-early-onset_inflammatory_bowel_disease,Severe_Combined_Immunodeficiency_and_CID,Autoimmunity_and_autoinflammatory_disorders,B_cell_and_Humoral_Immune_Deficiency,Hemophagocytic_Lymphohistiocytosis_and_Related,Phagocyte_Defect_and_Infectious_disease
XK	Cardiomyopathy_t2,Anemia_t2,Chorea,Anemia,Parkinson_Disease_and_Related
XPA	Dermatology_t2
XPC	Dermatology_t2
XPNPEP3	Hereditary_Autism_t2,Nephronophthisis_t1,Nephronophthisis_and_Related
XPO1	Lymphoma_t1,Hematologic_malignancy,Lymphoma_B_cell_t2
XPR1	Dystonia_t2,Parkinson's_disease_t2,Chorea,Fahr's_disease
XRCC1	corneal_dystrophy
XRCC2	Dermatology_t2,Skeletal_dysplasia_t2,Coagulation_t2,Congenital_hematologic_disease,Fanconi_anemia,Hereditary_breast_cancer,Hereditary_pancancer,Bone_Marrow_Failure_Germline_Predisposition,Breast_and_Ovarian_Cancer_Panel
XRCC4	Skeletal_dysplasia_t2,Proportionate_short_stature_t2,Hereditary_Microcephaly_t2
XYLT1	Skeletal_dysplasia_t2,Skeletal_Dysplasia_Panel
XYLT2	Skeletal_dysplasia_t2,Hearing_loss_t2,Osteogenesis_imperfecta
YARS1	Charcot-Marie-Tooth_disease_t1,Charcot-Marie-Tooth,Charcot_Marie_Tooth_and_Related
YARS2	Anemia_t1,Anemia
YME1L1	Hereditary_retinopathy_t2,Optic_neuropathy_and_Related
YWHAE	Cardiomyopathy_t2
YY1	Epilepsy_t2
ZAP70	Primary_immune_deficiency_t2,Inflammatory_Bowel_t2,Hematologic_malignancy,Primary_immunodeficiency,Very-early-onset_inflammatory_bowel_disease,Autoimmunity_and_autoinflammatory_disorders,B_cell_and_Humoral_Immune_Deficiency,Severe_Combined_Immunodeficiency_and_CID
ZBTB16	Hereditary_Autism_t2
ZBTB24	Primary_immune_deficiency_t2,Hereditary_Autism_t2
ZC4H2	Hereditary_Autism_t2
ZCCHC12	Hereditary_Autism_t2
ZDHHC9	Connective_tissue_disorder_t2,Epilepsy_t2,Hereditary_Autism_t2
ZEB1	Hereditary_retinopathy_t2,Corneal_Dystrophy_and_Related,corneal_dystrophy,corneal_keratoconus
ZEB2	Epilepsy_t2,Hereditary_Microcephaly_t2,Hereditary_Autism_t1,Hirschsprung's_disease,Epilepsy_and_Related
ZFHX4	Myeloma_t1
ZFP57	Maturity-Onset_Diabetes_of_the_Young,_MODY_t2,Hereditary_Autism_t2,Monogenic_diabetes_panel
ZFR	Hereditary_spastic_paraplegia_t2,Neurodegenerative_disease_t2
ZFYVE26	Charcot-Marie-Tooth_disease_t2,Ataxia_t2,Epilepsy_t2,Hereditary_Autism_t2,Neurodegenerative_disease_t2,Hereditary_spastic_paraplegia_t1,Ataxia,Hereditary_spastic_paraplegia_panel,Parkinson,Spastic_Paraplegia_and_Related
ZFYVE27	Ataxia_t2,Neurodegenerative_disease_t2,Hereditary_spastic_paraplegia_t1,Hereditary_spastic_paraplegia_panel,Spastic_Paraplegia_and_Related
ZIC1	Craniosynostosis
ZIC2	Epilepsy_t2,Hereditary_Autism_t2
ZMPSTE24	Dermatology_t2,Skeletal_dysplasia_t2
ZMYM3	Hereditary_Autism_t2
ZMYND10	Hereditary_Primary_Ciliary_Dyskinesia_t1
ZNF148	Hereditary_Microcephaly_t2

Supplementary Table 2. List of pathogenic or likely-pathogenic germline variants

Gene	Associated diseases
ZNF335	Hereditary_Microcephaly_t2
ZNF408	Hereditary_retinopathy_t2,Retinitis_pigmentosa_t1,Pediatric_retinal_vascular_disease,Retinitis_pigmentosa,Vitreoretinopathy,Retinitis_Pigmentosa_and_Related,Vitreoretinopathy_and_Related,retina
ZNF41	Hereditary_Autism_t2
ZNF423	Ataxia_t2,Hereditary_retinopathy_t2,Polycystic_Kidney_Disease_t2,Nephronophthisis_t1,Nephronophthisis_and_Related,retina
ZNF469	Dermatology_t2,Connective_tissue_disorder_t2,Hereditary_retinopathy_t2,Keratoconus_and_Related,corneal_keratoconus
ZNF507	Hereditary_Autism_t2
ZNF513	Hereditary_retinopathy_t2,Retinitis_pigmentosa_t1,Retinitis_pigmentosa,Retinitis_Pigmentosa_and_Related,retina
ZNF592	Ataxia_t2
ZNF674	Hereditary_Autism_t2
ZNF711	Hereditary_Autism_t2
ZNF804A	Hereditary_Autism_t2
ZNF81	Hereditary_Autism_t2
ZNHIT3	Hereditary_retinopathy_t2,Optic_neuropathy_and_Related
ZNHIT6	Hereditary_Autism_t2
ZRSR2	Hematologic_malignancy,ALL_and_others_t1,ALL_and_others_t2,AML,MDS_MPN
ZSWIM6	Hereditary_Autism_t2

**Case 1**	F/81	**Lung adenocarcinoma**	**Utility: Category II-1**
**Comments for Clinical Utility**	EGFR mutation and amplification is actionable alteration for lung adenocarcinoma. From the WGS data, co-occuring amplification and mutation of EGFR was identified which suggest possible resistance mechanism of erlortinib and osimertinib **[Category II-1 Drug resistance/responsive mechanisms].**		

Clinical Presentation> This case involves a patient diagnosed with Non-Small Cell Lung Cancer (NSCLC), which has metastasized to her liver and multiple bones, and is accompanied by pleural effusion.

The patient's cancer exhibits an EGFR mutation L858R, is negative for ALK, and is PD-L1 negative as determined by immunohistochemistry (clone 22C3 and SP263). The patient started erlotinib therapy on September 21, 2021, and achieved a partial response for the first response evaulation. However, New lesions developed throughout the lungs, confirming disease progression 7 months after erlotinib. Subsequent blood tests indicated circulating free DNA (cfDNA) positive for the EGFR T790M mutation. The regimen was change to 3rd generation TKI, lazertinib, from May 26, 2022, but she experienced severe side effects, including Grade 3 fatigue and Grade 3 anorexia. The patient did not experience any adverse events after switching to Osimertinib and was taking the medication well. However, the first response evaluation after three months of treatment showed an increase in the size of multiple lung masses, resulting in the evaluation of tumor response as disease progression. To identify the resistance mechanism, a biopsy was performed, and the patient was enrolled in the WGS study. On September 7, 2022, the patient's treatment was changed to palliative pemetrexed + carboplatin. She was transferred to another hospital at her request.

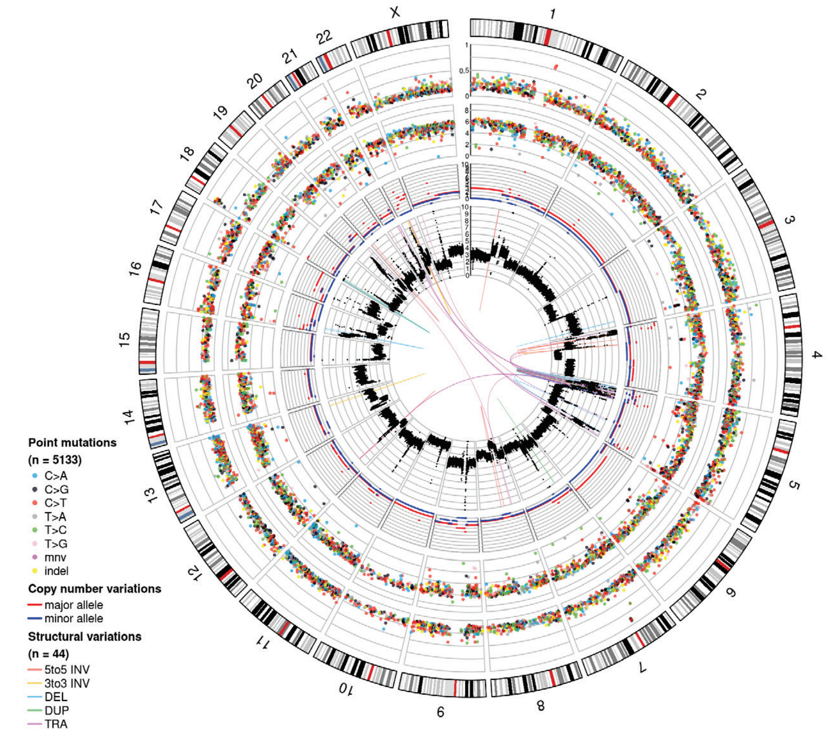

**Genome interpretation>**	Depth: Tissue: 30.4X, Normal: 16.7X	Total mutation counts: SNV: 4,671 Indel: 463 SV: 44
	Tumor fraction: 0.20, Mean ploidy: 2.7	Germline: not specific findings

Genome has EGFR p.L858R mutation and EGFR amplification. Considering variant allele frequency (0.035) and copy number status (78) of EFGR, EGFR amplification occurred prior to EGFR p.L858R mutation.

This is thought to explain the limited therapeutic response to erlotinib.

Case 2	M/60	**Colorectal cancer**	**Utility: Category I-2**
**Comments for Clinical Utility**	The PIK3CA mutation is implicated in metastatic breast cancer that is hormone-positive. Additionally, this mutation is recognised as a significant driver mutation in gastrointestinal cancers. The patient, who suffered from refractory colon cancer, was confirmed to have a PIK3CA (E545K) hotspot mutation and was given the chance to participate in a clinical trial. **[Category I-2 clinical trial, NCT04753203]**		

Clinical Presentation> This case concerns a 60-year-old male with metastatic colorectal cancer haboring a PIK3CA hotspot mutation.
He presented to the emergency department with dizziness and was found to be anaemic with a haemoglobin level of 4 mg/dl. A 7cm section of colon cancer was discovered in the hepatic flexure, along with multiple liver metastases. The diagnosis was confirmed as ascending colon cancer with unresectable liver metastases, resulting in a clinical stage of cT4N2M1. Due to symptomatic bowel obstruction, a loop ileostomy was performed, the presence of KRAS and NRAS mutation were confirmed and palliative bevacizumab + mFOLFOX6 was administered. The patient showed a stable response after 19 cycles of bevacizumab + mFOLFOX6 but progressed to new lesion lung metastases. He was treated with 10 cycles of aflibercept + FOLFIRI and achieved a response of stable disease, but progressed to metastases in both liver and lung. He received bevacizumab + trifluridine plus tipiracil as third-line chemotherapy, but exhibited rapid progression after one cycle. A colonoscopic biopsy of the hepatic flexure region was then conducted to perform cancer WGS. He was subsequently enrolled in the alpelisib + capecitabine clinical trial (NCT04753203) after confirmation of the PIK3CA mutation. This resulted in a significant reduction in the size of the lung metastases. He obtained a PFS of 3.1 months before experiencing progression.

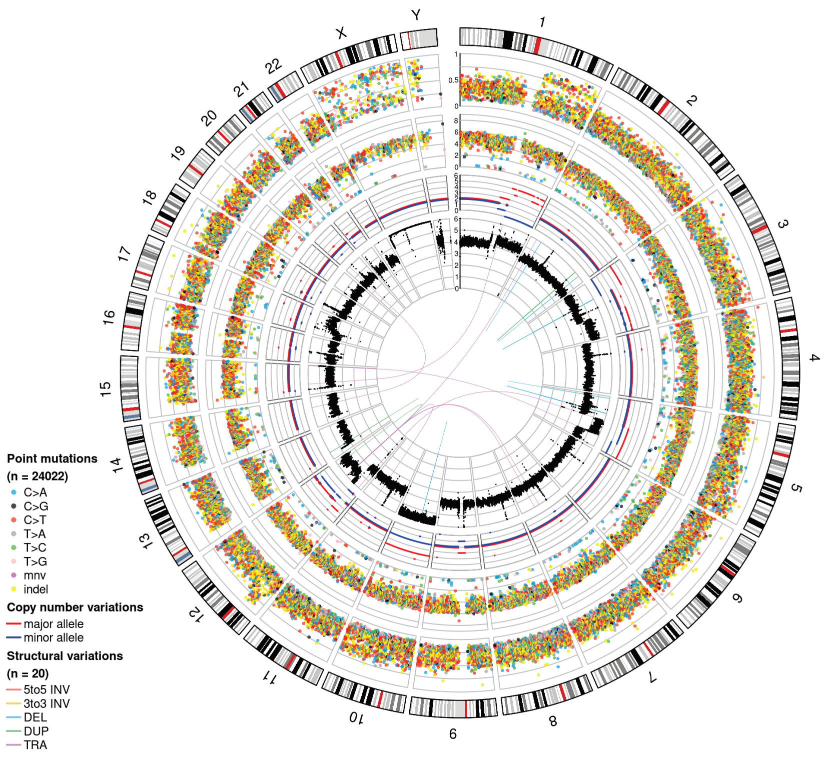

**Genome interpretation>**	Depth: Tissue: 28.5X, Normal: 15.7X	Total mutation counts: SNV: 17,690 Indel: 6,336 SV: 20
	Tumor fraction: 0.7, Mean ploidy: 4.1	Germline: not specific findings

KRAS p.G12S, ERBB3 p.G284R, and PIK3CA p.E545K mutations were identified. Mutaiotnal signature analysis revealed some proportion of damage by reactive oxygen species.

Case 3	M/54	Stomach cancer	Utility: Category I-2
**Comments for Clinical Utility**	The germline BRCA1 (gBRCA1) mutation is licensed for Olaparib in ovarian, breast, pancreatic, and prostate cancers; however, no PARP inhibitor is labelled for use in gastric cancer. There are clinical trials of PARP inhibitors in gastric cancer patients with BRCA1 mutations **[Category I-2 Clinical trial, NCT04171700]**. Whole genome sequencing of cancer revealed that the patient's gastric cancer was associated with a hereditary cancer syndrome caused by the gBRCA1 mutation [Category 2-1 hereditary] . In addition, the brother of the patient was tested for gBRCA1 carrier status and was advised to undergo extended cancer screening to prevent cancer.		

Clinical Presentation> The presented study involves a patient who has been diagnosed with advanced gastric cancer and has a familial history of cancer associated with germline BRCA1. A 54-year-old man presented with continuous abdominal pain one month prior to his visit. During the endoscopic examination, a 10cm ulcerofungating mass was identified on the greater curvature of the body. Poorly cohesive carcinoma was confirmed through an endoscopic biopsy. Laparoscopic examination found colon adhesions and peritoneal metastases, which were removable. The patient underwent total gastrectomy with segmental resection of the transverse colon. The patient was referred to the oncology department for palliative chemotherapy. He has received nivolumab and mFOLFOX6, exhibiting a partial response along with a durable and continuous response for 13 months. The patient has a gBRCA1 pathogenic mutation, his father died of gastric cancer at the age of 40, his sister developed breast cancer at the same age, and his niece was diagnosed with breast cancer at the age of 30. The patients questioned whether their younger brother had a gBRCA1 mutation, and subsequent testing revealed that he had the same mutation as the patient.

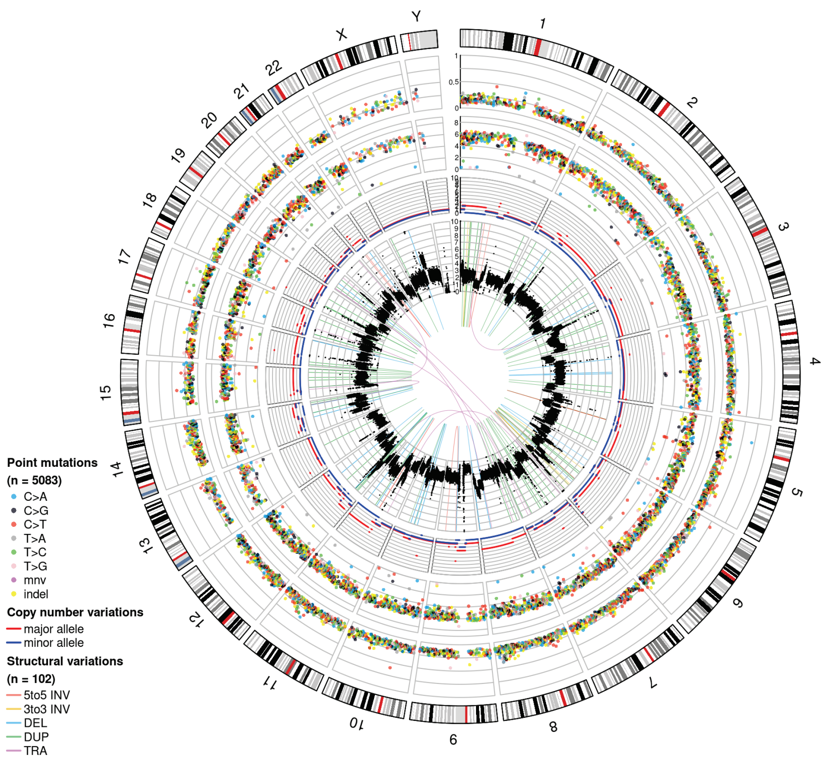

**Genome interpretation>**	Depth: Tissue: 37.5X, Normal: 17.65X	Total mutation counts: SNV: 4,330 Indel: 754 SV: 102	
	Tumor fraction: 0.15, Mean ploidy: 1.8	Germline:	***gBRCA1 p.Leu1780Pro***

This sample has deficient homologous recombination pathway evidenced by mutational signature SBS3, ID6, and HRD score of 0.71. We found germline pathogenic mutation in BRCA1 gene (p.Leu1780Pro) with loss of heterozygosity.

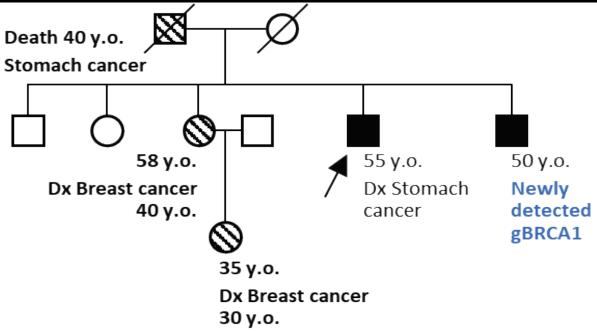

**Case 4**	F/53	**Breast cancer**	**Utility: Category II-3**
**Comments for Clinical Utility**	A patient with bilateral breast cancer who has a family history of her older sister dying of ovarian cancer at age 45 should be strongly suspected of having BRCA-associated hereditary breast cancer syndrome. The patient was also afraid of a family history of cancer and was very concerned about passing on the risk of cancer to her two daughters. However, the patient in this case did not have a homologous repair defect identified by WGS, did not have pathologic mutations in her germline data, and her overall genomic portrait resembled a typical luminal A breast cancer. On this basis, we concluded that the patient's hormone-positive breast cancer was sporadic **[Category II-3 Familial cancer].**		
**Clinical Presentation> The present study involves a patient with sporadic hormone-positive metastatic breast cancer with a rich family history of cancer, who would have been suspected of having a hereditary cancer syndrome.** A 53-year-old female presented with a palpable mass in the left breast. ER+, PR+, HER-2- invasive ductal carcinoma was identified in the left breast and papillary carcinoma in the right breast. Multiple pulmonary metastases were identified and pleural seeding was confirmed, leading to the diagnosis of bilateral cT4N1M1 breast cancer. The patient had a family history of gastric cancer in her father and gallbladder cancer in her mother, and her oldest sister died of ovarian cancer at the age of 45. She presented with dyspnea due to pleural effusion and achieved a partial response after starting palliative paclitaxel + carboplatin.			
**Genome interpretation>**	Depth: Tissue: 33.3X, Normal: 15.6X	Total mutation counts: SNV: 2,287 Indel: 329 SV: 50	
	Tumor fraction: 0.9, Mean ploidy: 1.9	Germline: not specific findings	
Most of the mutations were attributable to clock-like signature (SBS5, ID1). Chromothripsis and kataegis were noted in chr20.			

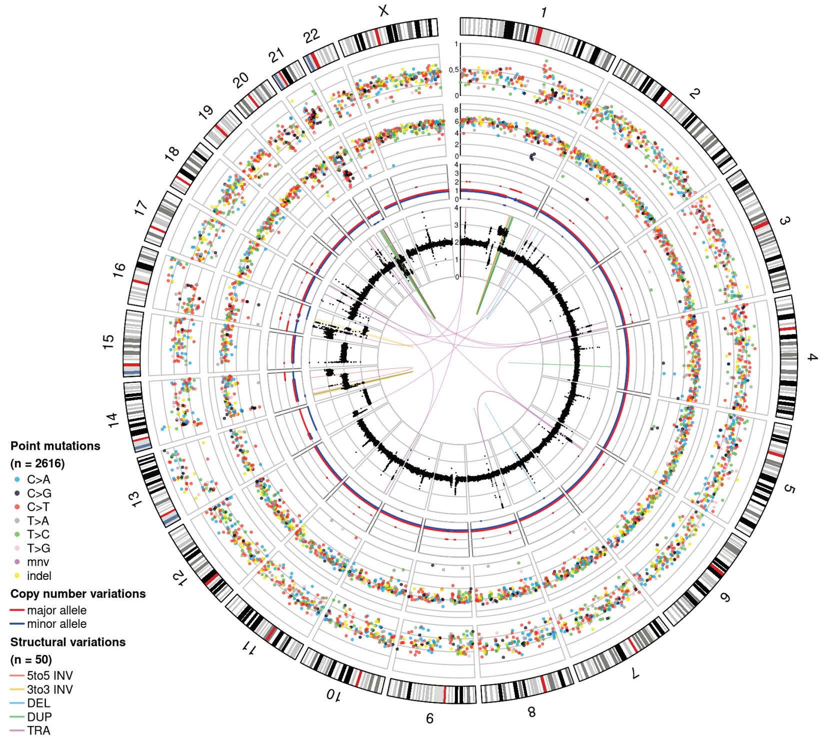

Case 5	M/67	Colorectal cancer	Utility: No (Category I-1)
**Comments for Clinical Utility**	No actionable driver was reported.		

Clinical Presentation> A 67-year-old man presents to a local clinic with a one-month weight loss of 5 kg and right upper quadrant pain since 2 weeks ago, and is referred to a tertiary center with a confirmed liver mass on ultrasound. On work up, sigmoid colon cancer was confirmed and metastases were found in both lobes of the liver. He was treated with bevacizumab plus mFOLFOX6 for unresectable sigmoid colon cancer with clinical stage cT4N3M1, and the best of response was a partial response.

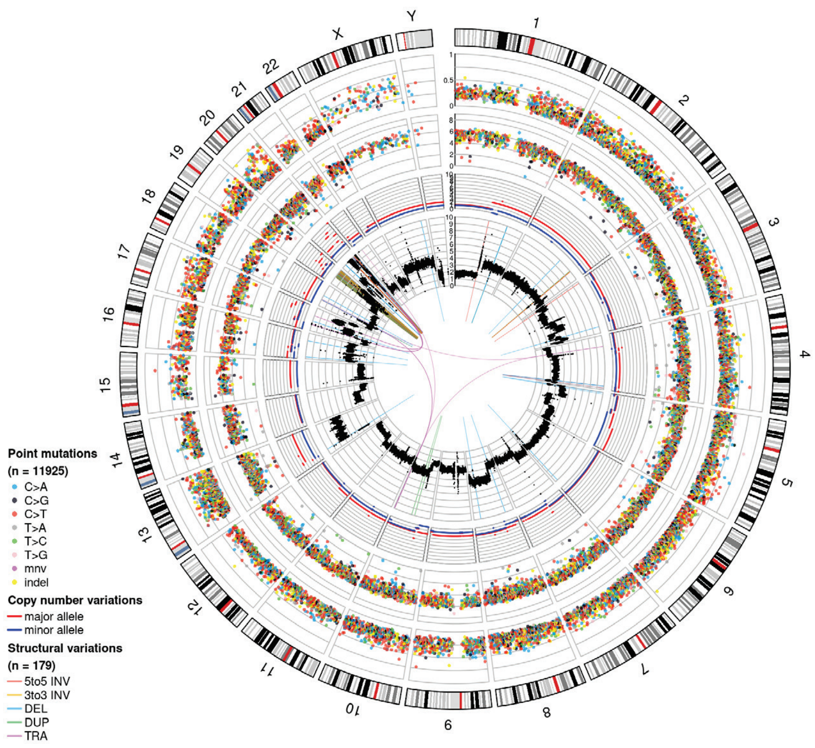

**Genome interpretation>**	Depth: Tissue: 29.8X, Normal: 19.1X	Total mutation counts: SNV: 11,263 Indel: 2,074 SV: 178
	Tumor fraction: 0.28, Mean ploidy: 4.5	Germline: not specific findings

Chr 19 and 20 were damaged by chromothripsis. There was a chromoplexy involving chr4, 10, and 18. Whole genome duplication had occured resulting in mean ploidy of 4.5.

**Case 6**	F/54	**Non-smal cell lung cancer**	**Utility: Category I-1**
**Comments for Clinical Utility**	Cancer WGS detected two umcommon EGFR mutations (G719A and R776H). These uncommon EGFR mutations are recommnended to use second or third generation EGFR TKI (afatinib or osimertinib) **[Category I-1 FDA approved drug]**.		

Clinical Presentation> The patient is a 54-year-old female with non-small cell lung cancer (NSCLC) who has two EGFR mutations (G719A, R776H) in WGS but one in PNA clamp PCR (G719A).

A 54-year-old female patient was diagnosed with NSCLC after a right lower lobe mass was discovered during an outpatient evaluation for flank pain. The patient reported a 10 year history of working with CO2 welding in a shipyard. She was never a smoker. A 1.9 cm consolidation was found in the right lower lobe, leading to a right lower lobectomy with clinical staging of cT1bN0M0. Histological examination of the surgical specimen verified an invasive adenocarcinoma that was poorly differentiated, with 50% acinar, 30% papillary, and 20% solid components. pT1c (2.1 cm) staging was reported and there was no involvement of reginal lymph node and the pleura. EGFR G719A was identified using the PNA clamp EGFR PCR test (PANAMutyper™) on the surgical tissue. No further adjuvant chemotherapy was given, and the patient is free from disease at 1-year follow-up.

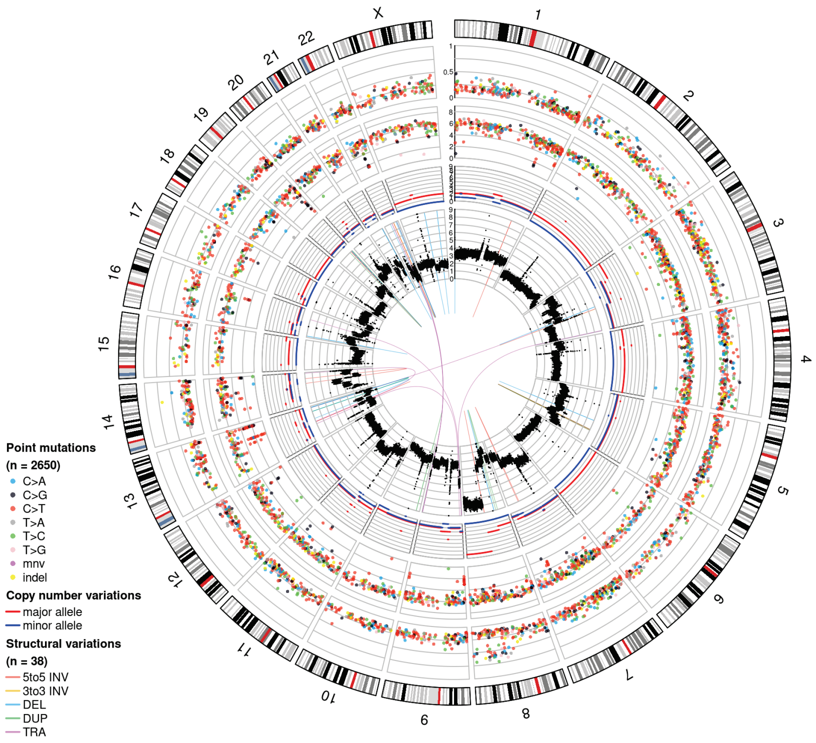

**Genome interpretation>**	Depth: Tissue: 28.0X, Normal: 16.3X	Total mutation counts: SNV: 2,486 Indel: 166 SV: 38
	Tumor fraction: 0.27, Mean ploidy: 2.6	Germline: not specific findings

EGFR p.G719A and EGFR p.R776H mutations were identified. The q arm of chr8 seems to be amplified early in the tumorigenesis.

Supplementary Table 3

Case 7	M/44	**Stomach cancer**	**Utility: No (Category I-1)**
**Comments for Clinical Utility**	No actionable driver was reported.		

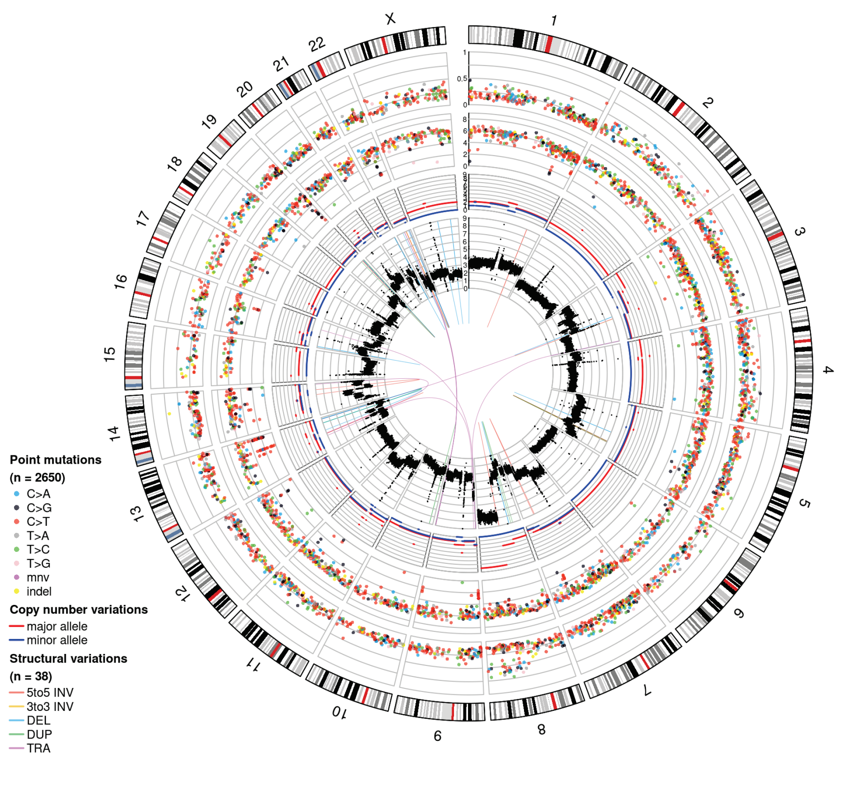

Clinical Presentation> The case involves a 44-year-old man with advanced gastric cancer.
The patient presented with persistent dyspepsia for the past three months and experienced a weight loss of 5 kg in the same period. A diagnosis of gastric cancer was made following an esophagoduodenal scopic examination, which revealed a 2.5cm metastatic mass in the liver, diagnosed as gastric adenocarcinoma with clinical staging cT4N3M1. The multidisciplinary tumour board recommended surgical resection followed by palliative chemotherapy for advanced gastric cancer with oligometastasis. Total gastrectomy, distal pancreatectomy and splenectomy were performed along with segmentectomy for removal of the mass in liver S8. One month after surgery, the patient started palliative chemotherapy with Nivolumab + FOLFOX and achieved a partial response. Subsequently, after 14 cycles, the patient was diagnosed with multiple liver metastases, and was thus started on second line chemotherapy with paclitaxel + ramucirumab.

**Genome interpretation>**	Depth: Tissue: 28.1X, Normal: 16.0X	Total mutation counts: SNV: 4,700 Indel: 5,060 SV: 45
	Tumor fraction: 0.98, Mean ploidy: 2	Germline: not specific findings

Higher number of indel mutations was noted. The q-arm of chr3 was damaged by chromothripsis.

Case 8	M/78	Gastrointestinal stromal tumor	Utility: Category I-1
**Comments for Clinical Utility**	PDGFR D842V is the major TKI resistance mutation in GIST, with avapritinib recommended as first-line therapy and dasatinib suggested as an alternative (NCCN guideline, JAMA Oncol. 2018;4(6):814-820) **[Category I-1 FDA approved drug]**.		

Clinical Presentation> This patient is a case of PDGFR D842V mutated GIST with primary resistance to conventional tyrosine kinase inhibitors.

The patient had failed multiple TKIs, including imatinib and sunitinib, and had undergone multiple surgical resections of the malignant GIST. WGS identified PDGFR D842V, which is known to be resistant to all major TKIs. Avapritinib is known to be an effective agent, but was not available, so dasatinib was administered as an alternative. After only 1 cycle, the patient, who was in poor general condition with a huge abdominal mass, died due to pneumonia.

small bowel GIST
s/p omentectomy (2013.08.23) 10.5 x 8.5 x 6cm 30/10 HPF
s/p adjuvant imatinib 1yr 6month
- recurred
 s/p small bowel segmental resection (2017.06.21)
 s/p palliative imatinib 300mg (2017.07.20-12.10)
- progression of remnant seeding nodule (2017.09.20 2017.12.05)
 s/p palliative sunitinib (2017.12.11-2018.10.08) best of response: PR
- progression of remnant seeding nodule and new lesion
 s/p palliative regorafenib 400mg (2019.01.22-04.10) best of response: PD
 s/p palliative imatinib high dose 600mg (2019.04.17- 400mg (2019.06.11-07.10) PD
 s/p small bowel segmental resection (2019.08.07)
 s/p multiple GIST excision (2020.04.27)
 s/p multiple GIST excision (2021.10.06)
 s/p multiple GIST excision SB segmental resection (2022.10.06)
 s/p palliative dasatinib (2022.11.04~) best of response: SD

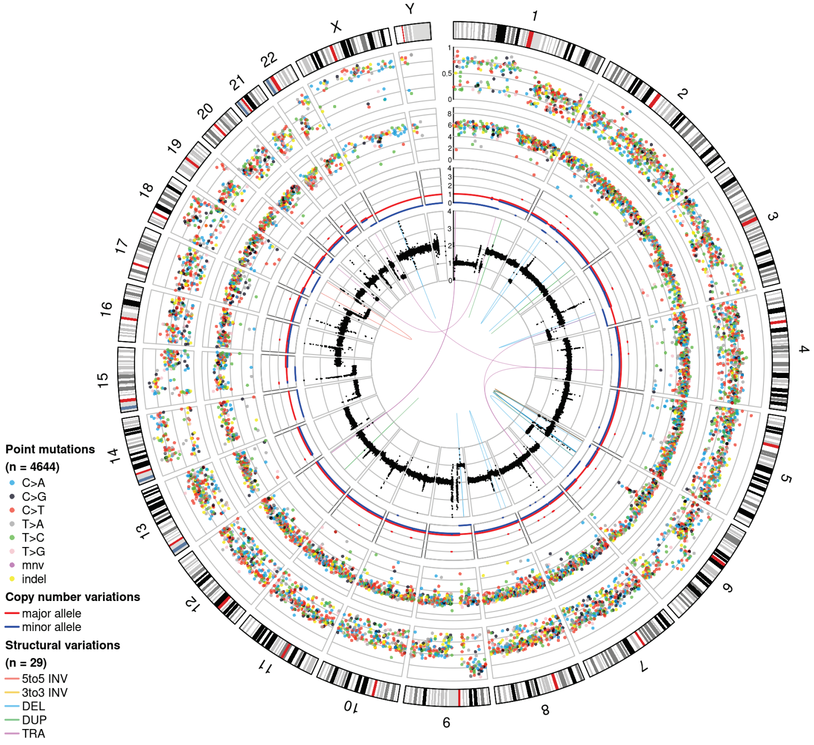

Genome interpretation>	Depth: Tissue: 37.6X, Normal: 14.6X	Total mutation counts: SNV: 4,251 Indel: 393 SV: 29
	Tumor fraction: 0.89, Mean ploidy: 1.7	Germline: not specific findings

PDGFRA p.D842V mutation was identified. Both allele of CDKN2A gene were deleted.

Supplementary Table 3

**Case 9**	M/57	**Non-smal cell lung cancer**	**Utility: Category I-1**
**Comments for Clinical Utility**	EML-ALK is indicated to use ALK TKI (brigatinib or alectinib) for the metastatic ALK rearranged NSCLC **[Category I-1 FDA approved drug]**.		

Clinical Presentation> The patient is a 57-year-old man diagnosed with double primary cancer of dedifferentiated liposarcoma (ddLPS) and ALK-rearranged non-small cell lung cancer.

The patient presented with a palpable mass in the right lower quadrant for one month and was referred after an abdominal ultrasound revealed a substantial growth. There was a retroperitoneal mass measuring 9.3 x 21.5 x 27.3 cm, which received a diagnosis of dedifferentiated liposarcoma (ddLPS) at stage cT4N3M1. During the imaging analysis, a 15 x 7mm peribronchiolar nodule in the right lower lobe was detected, indicating the possibility of primary lung cancer. During the imaging investigation, a 15 x 7mm peribronchiolar nodule was detected in the right lower lobe, indicating possible primary lung cancer. Thoracoscopic wedge resection was carried out for diagnostic and therapeutic purposes, and pathological analysis confirmed poorly differentiated adenocarcinoma that was ALK-IHC positive (pT1bN0M0). Instead of carrying out additional lobectomy for early-stage ALK-rearranged NSCLC in the presence of remaining ddLPS, the multidisciplinary tumor board recommended initiating palliative chemotherapy for ddLPS. Therefore, treatment with doxorubicin and cyclophosphamide was started.

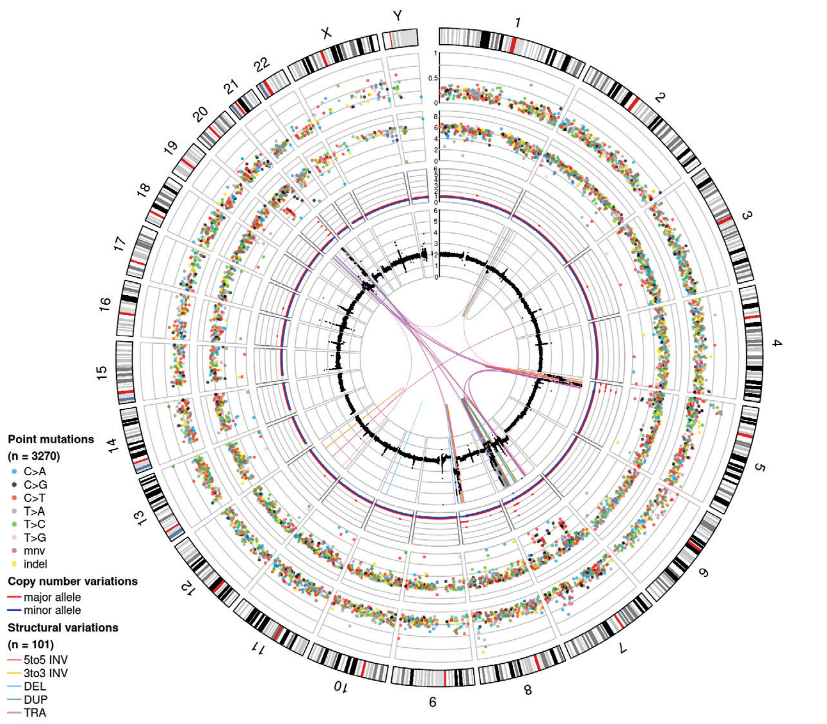

**Genome interpretation>**	Depth: Tissue: 33.2X, Normal: 18.1X	Total mutation counts: SNV: 3,446 Indel: 5,136 SV: 56
	Tumor fraction: 0.98, Mean ploidy: 2	Germline: not specific findings
EML4::ALK fusion has been identified. Interchromosomal chromthripsis was observed between chr5, 7, 8, and 20.		

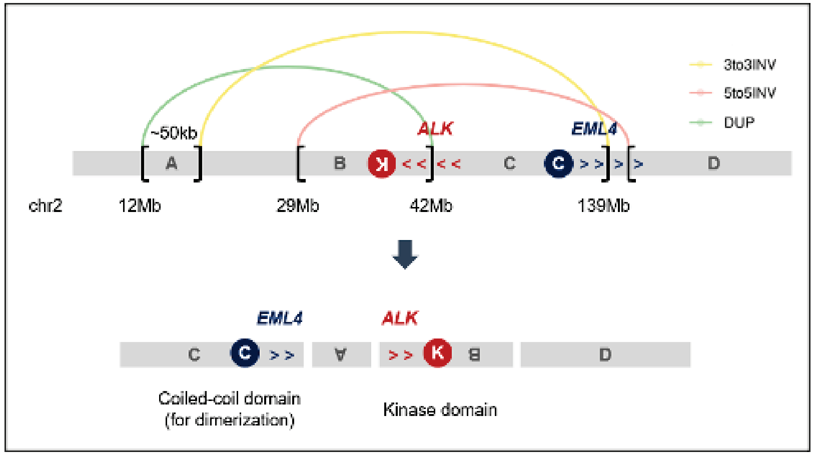

**Case 10**	M/81	**Stomach cancer**	**Utility: Category I-1**
**Comments for Clinical Utility**	Pembrolizumab is indicated for the metastatic solid cancer with microsatellite high (MSI-H), including gastric cancer **[Category I-1 FDA approved drug]**		

Clinical Presentation> The patient is a 81-year-old woman diagnosed with gastric cancer with MSI-H

An 81-year-old female patient with pre-existing medical conditions of bronchial asthma, hypertension, Parkinson's disease, and aortic stenosis has presented to the hospital with a complaint of melena. An esophagoduodenoscopic exam revealed an elevated lesion near the posterior wall body and accompanying bleeding. Histological examination confirmed moderately differentiated adenocarcinoma, and the patient underwent total gastrectomy with a diagnosis of clinical stage cT3N1M0. The pathological diagnosis of the surgical specimen confirmed moderately differentiated tubular adenocarcinoma. The pathological staging was pT2N0M0. The patient was treated for pneumonia in the intensive care unit during post-operative recovery, but unfortunately died after repeated improvement and worsening of the pneumonia over a period of six months.

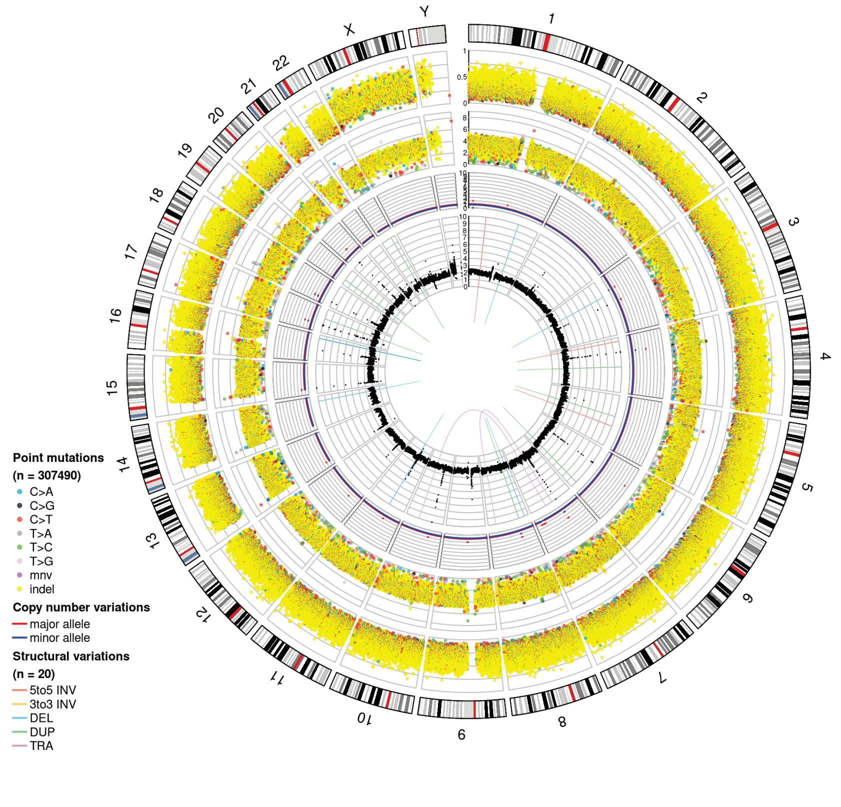

**Genome interpretation>**	Depth: Tissue: 36.6X, Normal: 21.5X	Total mutation counts: SNV: 93,337 Indel: 340,150 SV: 20
	Tumor fraction: 0.9, Mean ploidy: 4	Germline: not specific findings

Very high number of indels. Mutational signature analysis revealed mutational process associated with mismatch deficiency (e.g. SBS44, SBS20, SBS30)

**Case 11**	M/84	**Colorectal cancer**	**Utility: Category I-1**
**Comments for Clinical Utility**	BRAF V600E is indicated for encorafenib in colorectal cancer **[Category I-1 FDA approved drug]**.		

Clinical Presentation> An 84-year-old man was referred to a tertiary center with a 5 kg weight loss over 2 months and a palpable mass in the left lower abdomen for 2 weeks. Further evaluation confirmed stage S colon cancer with bilateral pulmonary metastases and peritoneal metastases. His clinical staging was cT4N3M1 with unresectable sigmoid colon cancer, but CT findings showed microperforation and focal abscess in the primary cancer site, so low anterior resection was performed first. Palliative mFOLFOX6 treatment was administered for the first time at 4 weeks postoperatively.

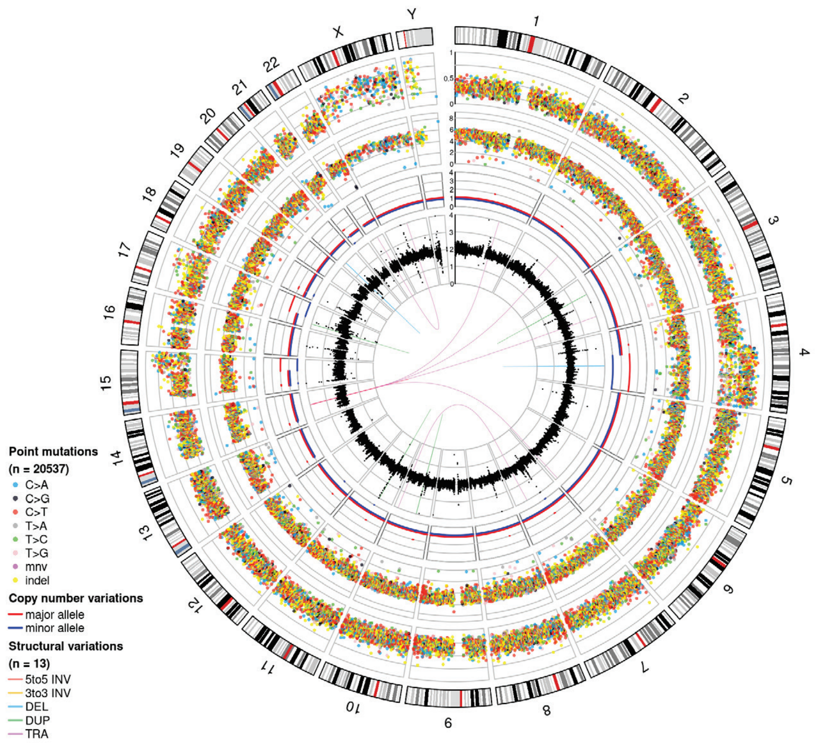

**Genome interpretation>**	Depth: Tissue: 25.5X, Normal: 18.2X	Total mutation counts: SNV: 15,022 Indel: 5,516 SV: 13
	Tumor fraction: 0.7, Mean ploidy: 1.9	Germline: not specific findings

PIK3CA p.E545K and BRAF p.V600E mutations were identified.

<table>
<tr><td>Case 12</td><td>F/60</td><td>Colorectal cancer</td><td>Utility: No (Category I-1)</td><td rowspan="3">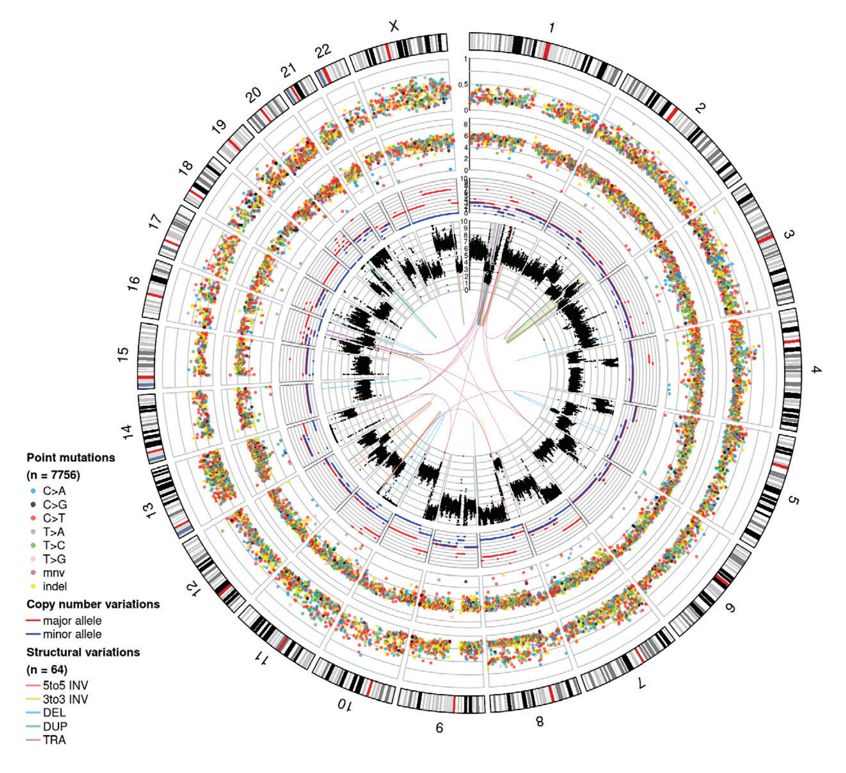
</td></tr>
<tr><td colspan="2">Comments for Clinical Utility</td><td colspan="2">No actionable driver was reported.</td></tr>
<tr><td colspan="4">Clinical Presentation> A 60-year-old woman with a history of HER-2-positive and hormone-positive early breast cancer, stage IA non-small cell lung cancer, and adenocarcinoma presents with a suspicious colon cancer in the hepatic flexure during routine follow-up. The clinical staging was cT3N0M0 and right hemicolectomy was performed. Postoperative pathology results reported adenocarcinoma, moderately differentiated, pT3N1, and identified lymphovascular invasion and perineural invasion. He received 12 cycles of mFOLFOX6 as adjuvant chemotherapy.</td></tr>
<tr><td colspan="2" rowspan="2">Genome interpretation></td><td>Depth: Tissue: 28.9X, Normal: 13.6X</td><td colspan="2">Total mutation counts: SNV: 6,403 Indel: 1,354 SV: 64</td></tr>
<tr><td>Tumor fraction: 0.2, Mean ploidy: 5.4</td><td colspan="2">Germline: not specific findings</td></tr>
<tr><td colspan="5">Canonical driver mutations of colorectal cancer were identified: APC and TP53 mutation. A significant level of copy number variation and whole genome duplication were observed.</td></tr>
</table>

**Case 13**	M/74	**Non-smal cell lung cancer**	**Utility: No (Category I-1)**	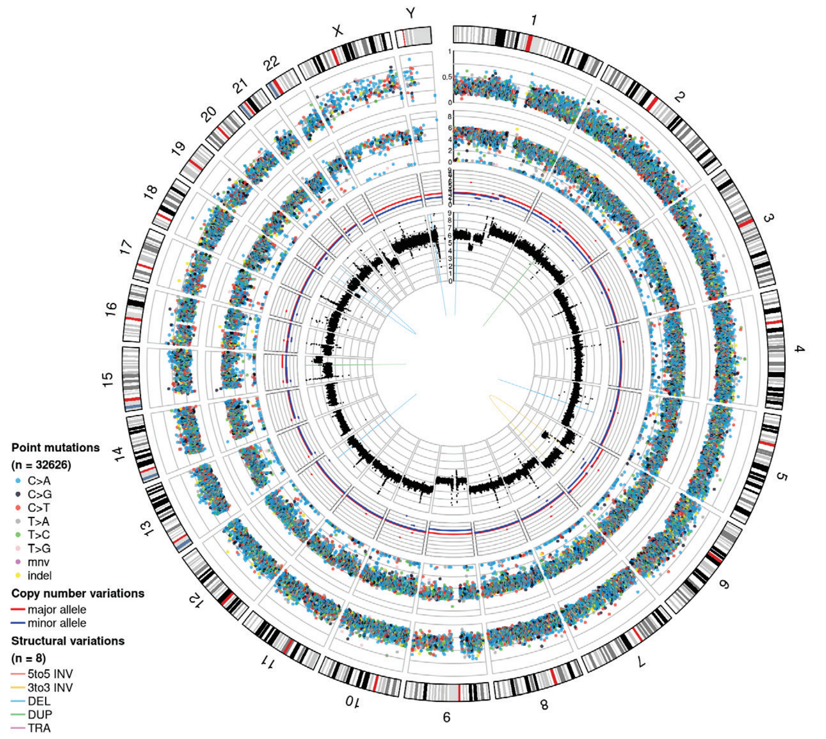
**Comments for Clinical Utility**	No actionable driver was reported.			
Clinical Presentation> A 74-year-old man with a 45 pack-year smoking history presented with a case of early stage Ia non-small cell lung cancer. The patient was asymptomatic, bronchial asthma was diagnosed, and a chest CT performed revealed a lung nodule. The clinical staging was cT1aN0M0 and a left upper lobectomy was performed. Postoperative pathology reported adenocarcinoma, moderately differentiated, pT1aN0 without pleural involvement and vascular, perineural, lymphatic invasion.				
**Genome interpretation>**	Depth: Tissue: 20.4X, Normal: 12.9X	Total mutation counts: SNV: 31,183 Indel: 1,444 SV: 8		
	Tumor fraction: 0.9, Mean ploidy: 5.8	Germline: not specific findings		
High proportion of mutational signatures attributable to tobacco smoking were observed: SBS4 and ID3.				

**Case 14**	F/61	**Colorectal cancer**	**Utility: Category I-1**
**Comments for Clinical Utility**	Pembrolizumab is indicated for the metastatic solid cancer with microsatellite high (MSI-H), including colorectal cancer **[Category I-1 FDA approved drug]**.		
Clinical Presentation> A patient presented with worsening abdominal pain for 1 month and was confirmed to have ascending colon cancer with multiple metastases to the liver and lung. Colonoscopy confirmed poorly differentiated adenocarcinoma in the ascending colon. After diagnosis, the patient progressed rapidly, presented with lactic acidosis and unstable vital signs, was admitted to the intensive care unit, and died unresponsive to 2 cycles of FOLFOX chemotherapy.			
**Genome interpretation>**	Depth: Tissue: 44.1X, Normal: 14.6X	Total mutation counts: SNV: 78,333 Indel: 272,623 SV: 25	
	Tumor fraction: 0.5, Mean ploidy: 1.9	Germline: not specific findings	
This sample has deficient DNA mismatch repair pathway evidenced by high proportion of SBS44, high number of indel and MSI-score of 11.58 (cutoff 2.0).			

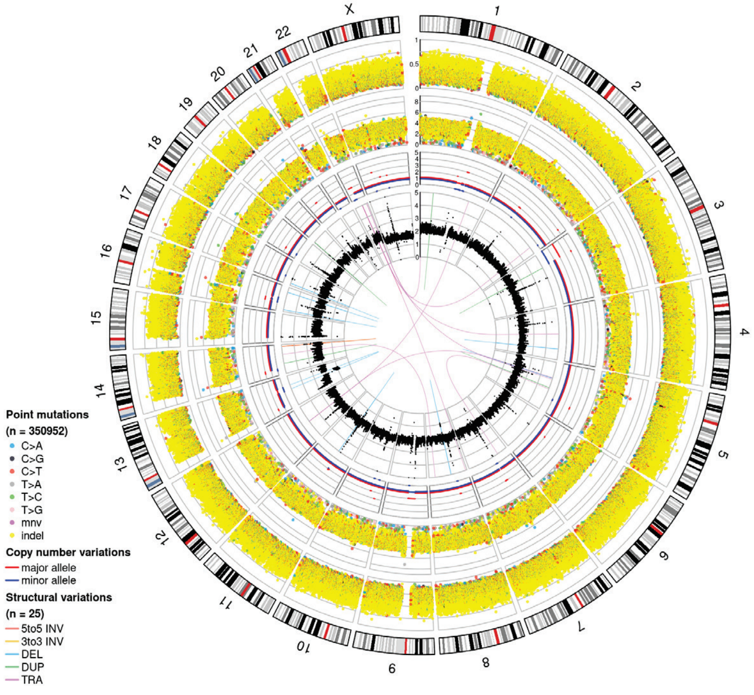

**Case 15**	M/79	**Non-smal cell lung cancer**	**Utility: Category I-1**
**Comments for Clinical Utility**	EGFR TKIs such as erlotinib or osimertinib are approved and available for lung adenocarcinoma with EGFR exon 19 deletion **[Category I-1 FDA approved drug]**.		

Clinical Presentation> A 79-year-old man with no smoking history has early stage lung cancer with a history of hypertension and surgery for thyroid cancer.
The patient was asymptomatic and was referred because of a lung nodule identified on chest CT during a physical examination. The clinical staging was cT1N0M0 and an anterobasal segmentectomy was performed. Postoperative pathology was reported as pT1bN0 with adenocarcinoma, moderately differentiated, papillary pattern predominant pleural involvement and no vascular, perineural or lymphatic involvement.

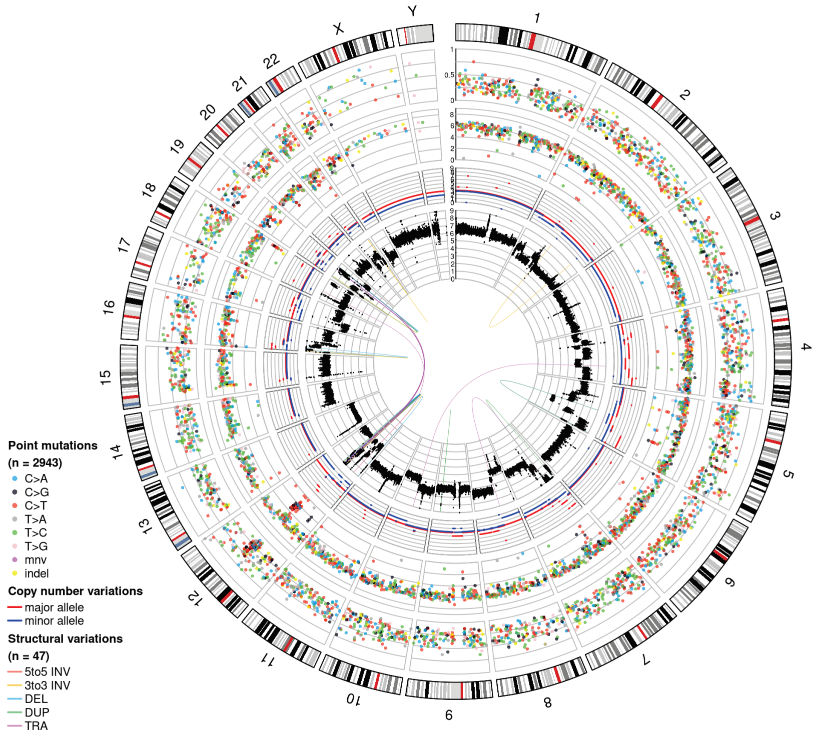

**Genome interpretation>**	Depth: Tissue: 23.5X, Normal: 13.0X	Total mutation counts: SNV: 2,774 Indel: 171 SV: 47
	Tumor fraction: 0.98, Mean ploidy: 5.7	Germline: not specific findings

EGFR p.L878R mutation was identified. Mean ploidy was 5.7 implying whole genome duplication.

**Case 16**	F/54	**Breast cancer**	**Utility: Category I-1**
**Comments for Clinical Utility**	NRG1 fusions are known to be rare, occurring in 0.2% of all breast cancers, and CFAP52 is a lesser-known fusion partner. For NRG1 fusions, afatinib is known to be potentially effective. Based on promising clinical trial results for genocutuzumab, the FDA granted it Breakthrough Therapy designation for pancreatic cancer, and there are expectations that the indication will be expanded in other cancers **[Category I-2 Clinical trial, NCT04100694].**		

Clinical Presentation> A 54-year-old woman with no history of smoking and a history of surgery for hormone-positive early breast cancer 9 years ago was referred with a confirmed lung nodule from breast cancer.

The patient underwent a total mastectomy 9 years ago (invasive ductal carcinoma, ER/PR/HER-2 8+/8+/0) and received 6 cycles of CMF followed by tamoxifen for 5 years as adjuvant anticancer therapy. During follow-up for breast cancer, a lung nodule is identified on chest CT and referred. Metastatic carcinoma was suspected and biopsy was performed by wedge resection. The postoperative pathology was reported as metastatic ductal carcinoma of the breast (GATA-3 and ER positive and HER-2 negative in IHC test). For metastatic breast cancer, palbociclib + letrozole was administered as palliative systemic therapy, and she has been progression-free for 14 months.

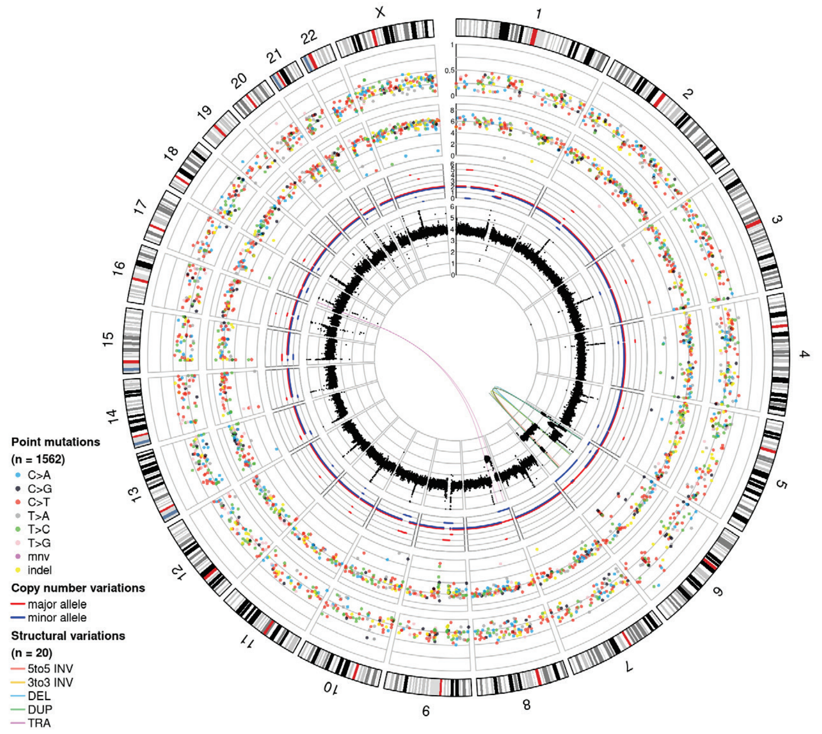

**Genome interpretation>**	Depth: Tissue: 47.2X, Normal: 12.6X	Total mutation counts: SNV: 1,386 Indel: 176 SV: 20
	Tumor fraction: 0.53, Mean ploidy: 3.5	Germline: not specific findings

CFAP52-NRG1 fusion was identified.

**Case 17**	M/55	**Colorectal cancer**	**Utility: Category I-1**
**Comments for Clinical Utility**	Pembrolizumab is indicated for the metastatic solid cancer with microsatellite high (MSI-H), including colorectal cancer **[Category I-1 FDA approved drug]**.		

Clinical Presentation> Asymptomatic 55-year-old male patient with cecal cancer identified on physical examination.

The patient had cecal cancer confirmed by colonoscopy performed at a medical examination and underwent right hemicolectomy with clinical staging of cT4N0M0. Postoperative pathology was reported as pT4N0 with adenocarcinoma, poorly differentiated, ulcerofungating type and identified no lymphovascular invasion and perineural invasion.

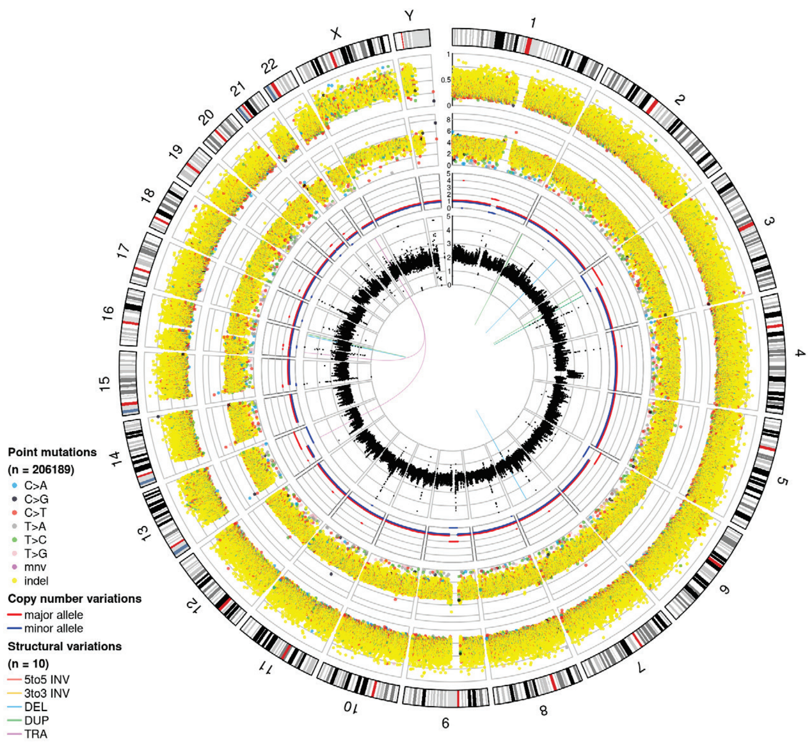

**Genome interpretation>**	Depth: Tissue:18.5X, Normal: 14.3X	Total mutation counts: SNV: 43,600 Indel: 162,589 SV: 10
	Tumor fraction: 0.4, Mean ploidy: 1.9	Germline: not specific findings

This sample has deficient DNA mismatch repair pathway evidenced by high proportion of SBS44, high number of indel and MSI-score of 4.03 (cutoff 2.0). We identified a somatic splice donor variant in inton 9 of MLH1 gene. The mutation was associated with loss of heterozygosity.

Case 18	M/55	Colorectal cancer	**Utility: Category I-3**
**Comments for Clinical Utility**	Mutated NRAS in colon cancer renders it resistant to EGFR-targeting antibodies such as cetuximab **[Category I-3, Elimination of ineffective treatment options]**.		

Clinical Presentation> A patient presented with worsening abdominal discomfort for 1 month and was found to have rectosigmoid colon cancer with huge and extensive liver metastases. On colonoscopy, the lumen was 100% obstructed by a fungating mass lesion from 10 cm above the superior anal verge of the rectum, and it was difficult to pass the endoscope, so colon stenting was performed. However, after the stent insertion, the stent pierced the posterior wall of the tumor and there was a perforation, so Hartmann's procedure was performed by emergency surgery. After surgery, the patient underwent two cycles of palliative mFOLFOX6; however, because of the continued deterioration of liver function caused by extensive liver metastasis, no additional chemotherapy was administered and the patient passed away.

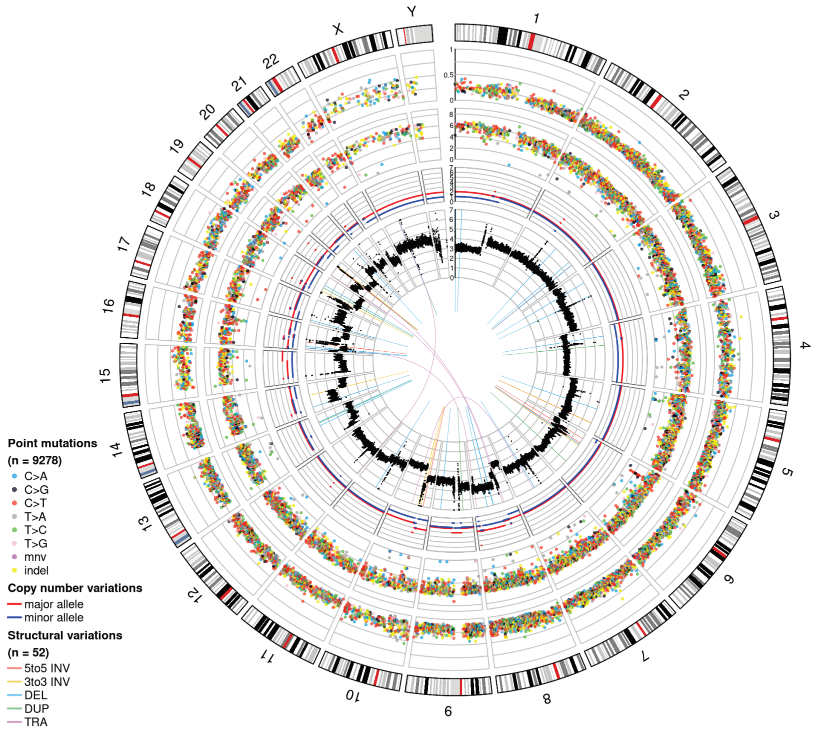

**Genome interpretation>**	Depth: Tissue: 36.13X, Normal: 15.4X	Total mutation counts: SNV: 7,837 Indel: 1,442 SV: 19
	Tumor fraction: 0.59, Mean ploidy: 3.6	Germline: not specific findings

Canonical driver mutations of colorectal cancer were identified: APC p.Y935*, KRAS p.G12V.

Case 19	M/68	Non-smal cell lung cancer	**Utility: Category I-1**
**Comments for Clinical Utility**	EGFR TKIs such as erlotinib and osimertinib are approved and available for lung adenocarcinoma with EGFR L858R mutation **[Category I-1 FDA approved drug]**.		

Clinical Presentation> A 68-year-old man with a history of confirmed cutaneous melanoma in the right pubic area underwent surgical resection one year ago. He was referred to thoracic surgery due to a nodule in the left upper lung that was suspicious for invasive adenocarcinoma, as demonstrated by chest CT. Clinical staging imaging showed a 1-cm subsolid nodule with no other metastases (cT1N0M0). The upper division of the left upper lung was removed by video-assisted thoracic surgery through segmentectomy. Histopathologic examination confirmed moderately differentiated invasive adenocarcinoma without pleural involvement (pT1a, PL0). No further treatment was administered, and the patient is being monitored for recurrence one year postoperatively.

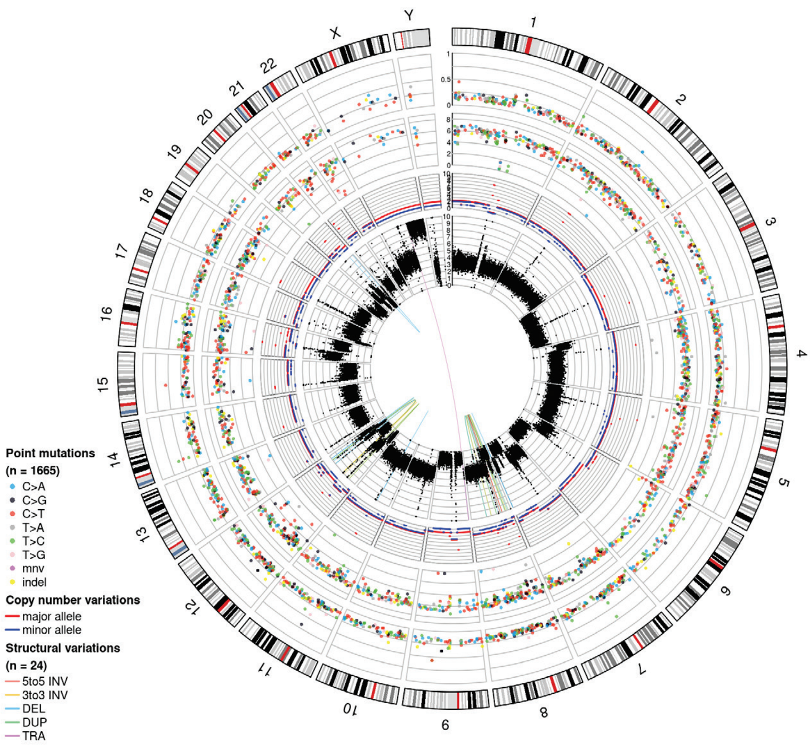

**Genome interpretation>**	Depth: Tissue: 44.8X, Normal: 14.2X	Total mutation counts: SNV: 1,708 Indel: 3,026 SV: 14
	Tumor fraction: 0.99, Mean ploidy: 2.1	Germline: not specific findings

EGFR p.L858R mutation was identified. There was a 5to5 inversion between ZMAT intron 4 and NRG1 intron yeidling ZMAT4-NRG1 fusion.

Case 20	F/74	Non-smal cell lung cancer	**Utility: Category I-1**
**Comments for Clinical Utility**	EGFR TKIs such as erlotinib or osimertinib are approved and available for lung adenocarcinoma with EGFR exon 19 deletion **[Category I-1 FDA approved drug]**.		

Clinical Presentation> A 74-year-old man with no significant medical history was referred for evaluation of a 3-cm mass in the right lower lobe. Imaging confirmed a clinical staging of cT2N0M0. Video-assisted thoracic surgery was planned, and surgical findings revealed a seeding nodule in the visceral and parietal pleura. Frozen biopsy confirmed metastatic carcinoma. To obtain a pathologic cancer diagnosis of the primary mass, only wedge resection was performed and the surgery was terminated. Erlotinib was prescribed as palliative systemic therapy. The patient responded well to erlotinib and demonstrated no evidence of disease in resonse evaluation at 8 months post-treatment, and has been on erlotinib for 14 months without progression.

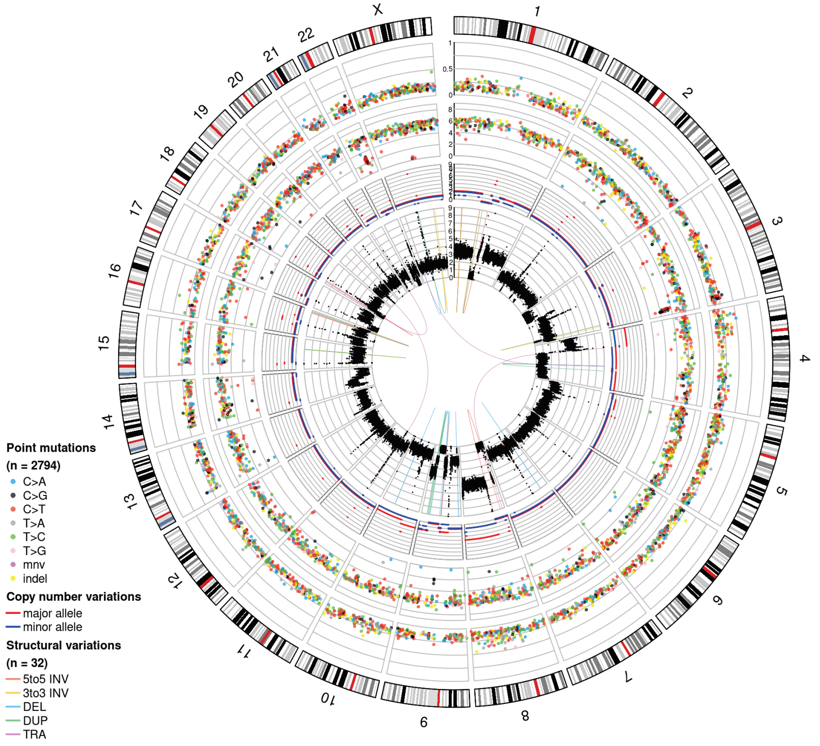

**Genome interpretation>**	Depth: Tissue: 39.0X, Normal: 21.1X	Total mutation counts: SNV: 2,521 Indel: 276 SV: 32
	Tumor fraction: 0.16, Mean ploidy: 1.7	Germline: not specific findings

EGFR exon19 deletion was identified.

Case 21	F/74	Colorectal cancer	**Utility: Category I-3**
**Comments for Clinical Utility**	Mutated NRAS in colon cancer renders it resistant to EGFR-targeting antibodies such as cetuximab **[Category I-3, Elimination of ineffective treatment options]**.		

Clinical Presentation> A 74-year-old woman with hypertension and parkinsonism was referred to a tertiary center because of the presence of colon cancer in the hepatic flexure on health checkup. Right hemicolectomy was performed under the diagnosis of ascending colon cancer, adenocarcinoma well differentiated, clinical stage cT2N0M0. Postoperative pathology reported adenocarcinoma, moderately differentiated with pathologic staging pT3N0, without lymphatic, venous, or perineural invasion. No additional adjuvant anticancer treatment was administered, and she has remained recurrence free for 1 year.

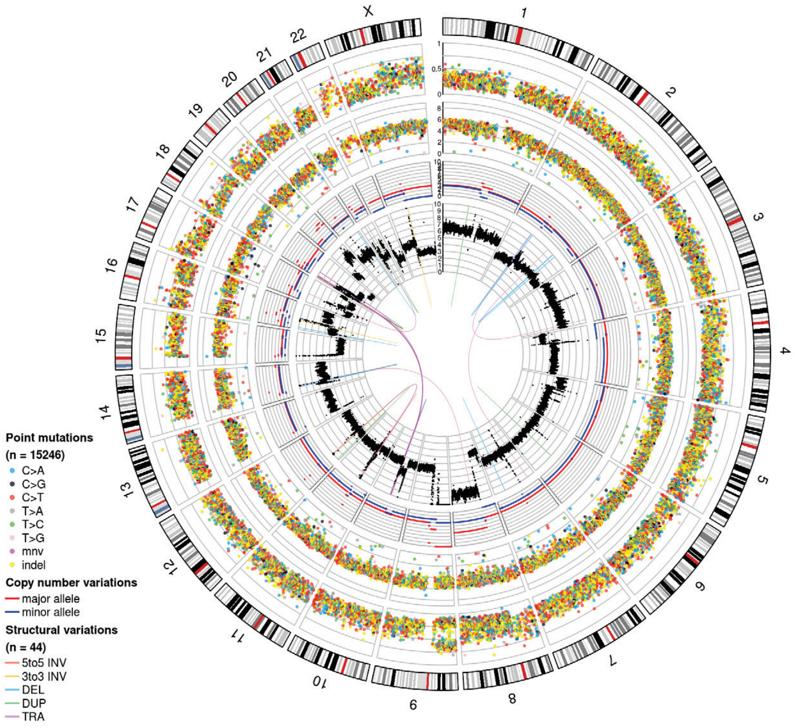

**Genome interpretation>**	Depth: Tissue: 33.1X, Normal: 16.2X	Total mutation counts: SNV: 10,880 Indel: 4,368 SV: 44
	Tumor fraction: 0.37, Mean ploidy: 5.1	Germline: not specific findings

NRAS p.Q61K and double hit mutation in APC exon 16 (p.R805* and p.S1411Rfs*4).

Case 22	M/52	Colorectal cancer	**Utility: Category I-1**
**Comments for Clinical Utility**	BRAF V600E is indicated for encorafenib in colorectal cancer **[Category I-1 FDA approved drug]**.		
**Clinical Presentation> A 52-year-old man with hypertension was referred to a tertiary center after a health checkup revealed an ascending colon cancer.** The clinical stage was cT4aN1M0. A right hemicolectomy was performed and postoperative pathology findings were reported as moderately differentiated adenocarcinoma with pathologic stage pT3N1a without lymphatic, venous, or perineural invasion. She received 12 cycles of mFOLFOX6 as additional adjuvant chemotherapy and has remained recurrence-free for 6 months.			
**Genome interpretation>**	Depth: Tissue: 31.2X, Normal: 13.5X	Total mutation counts: SNV: 25,700 Indel: 6,656 SV: 128	
	Tumor fraction: 0.52, Mean ploidy:3.1	Germline: not specific findings	
BRAF p.V600E mutation was identified. Non-clustered deletions (I-10kbp) are spread throughout the entire genome.			

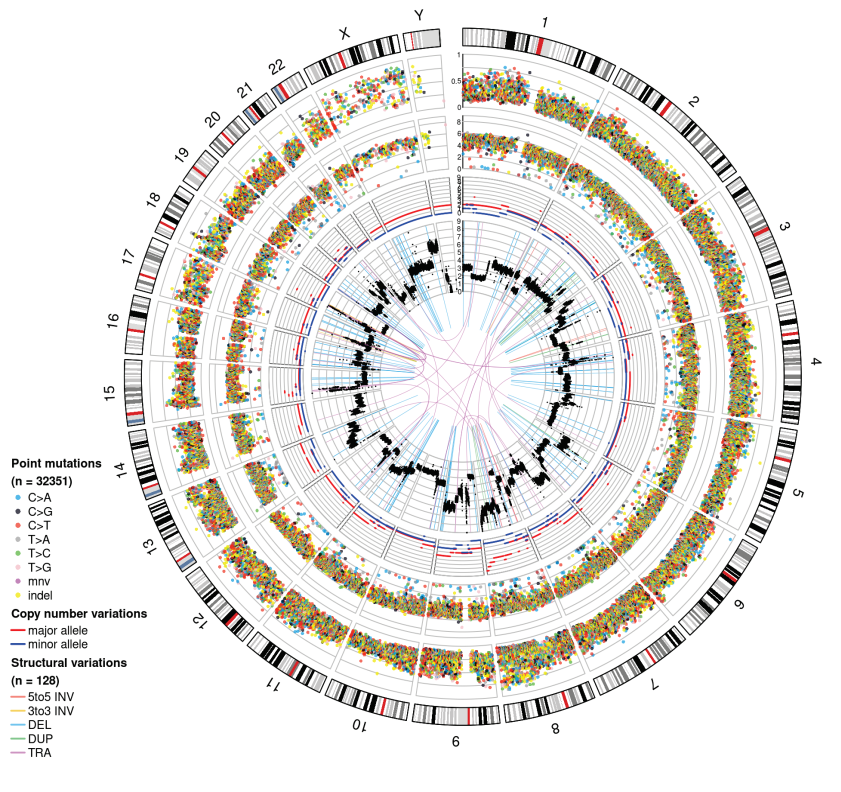

**Case 23**	M/72	**Stomach cancer**	**Utility: Category I-1**
**Comments for Clinical Utility**	Pembrolizumab is indicated for the metastatic solid cancer with microsatellite high (MSI-H), including gastric cancer **[Category I-1 FDA approved drug]**		
Clinical Presentation> A 72-year-old man with no significant medical history presented with a diagnosis of gastric adenocarcinoma in the anturum region during an upper GI endoscopy performed for epigastric discomfort. The clinical staging on imaging was cT4N2M0. A distal gastrectomy with Billroth II was performed, and the final pathological diagnosis was poorly differentiated tubular adenocarcinoma, pT3N1M0. The patient received adjuvant anti-cancer therapy with Tegafur/gimeracil/oteracil for one year and is currently under follow-up without any signs of recurrence.			
**Genome interpretation>**	Depth: Tissue: 28.9X, Normal: 20.7X	Total mutation counts: SNV: 45,137 Indel: 173,042 SV: 22	
	Tumor fraction: 0.3, Mean ploidy: 2	Germline: not specific findings	
MSI score is below threshold (0.36%). But, based on the number of indels and molecular signature (SBS44, ID1 and ID2), this sample is highly likely to be MSI-H.			

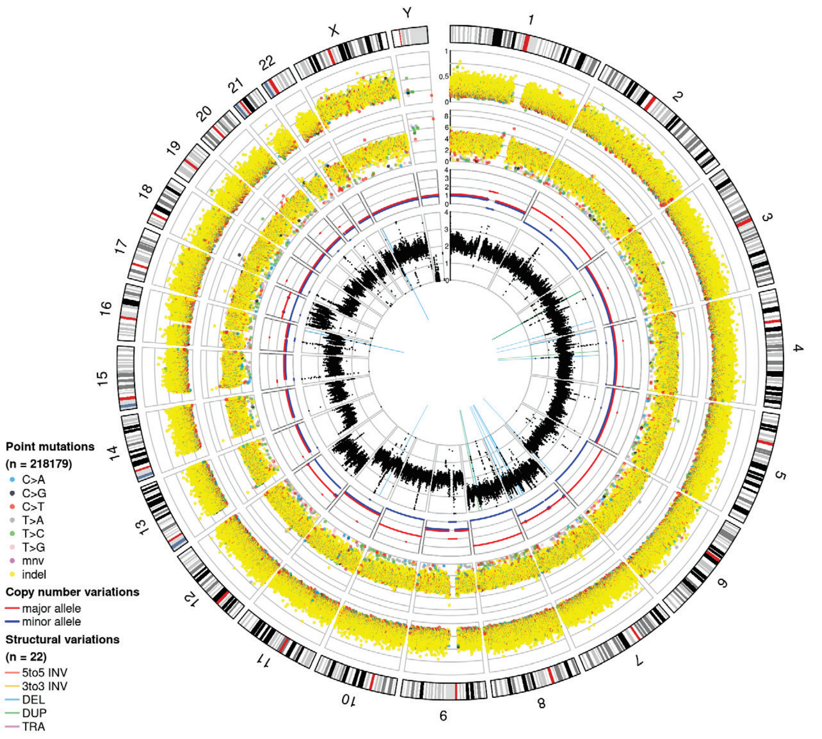

Case 24	F/81	Colorectal cancer	**Utility: No**
**Comments for Clinical Utility**	No actionable driver was reported.		

Clinical Presentation> An 81-year-old woman with hypertension, diabetes mellitus, and hyperlipidemia presented with abdominal pain and diarrhea, and workup revealed masses in the sigmoid colon and gallbladder, paraaortic LN enlargement, and adrenal metastases. Because of colonic obstruction and abdominal pain, anterior resection and cholecystectomy were performed as palliative surgery. Postoperative pathology findings were reported as sigmoid colon adenocarcinoma, poorly differentiated, pT3N2b with lymphatic, venous, perineural invasion positive, and papillary neoplasm in situ with high grade intraepithelial neoplasia in the gallbladder. The patient required palliative chemotherapy, but due to the patient's medical condition and advanced age, both the patient and guardian refused further chemotherapy. She has received only supportive care and is alive more than one year after surgery.

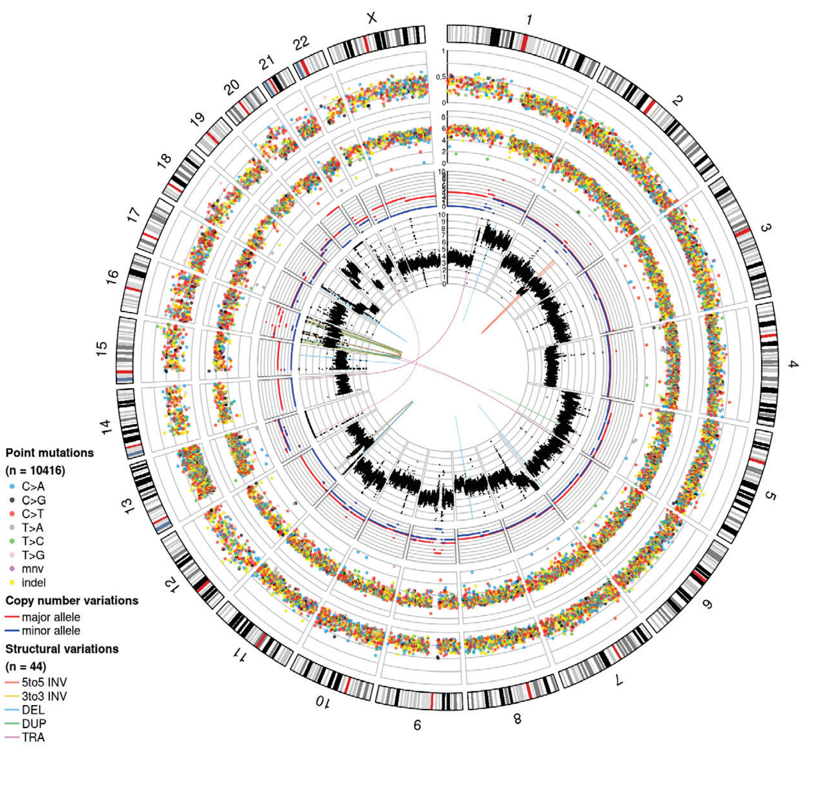

**Genome interpretation>**	Depth: Tissue: 18.4X, Normal: 12.1X	Total mutation counts: SNV: 4,028 Indel: 829 SV: 24
	Tumor fraction: 0.23, Mean ploidy: 5.4	Germline: not specific findings

Canonical drivers of colorectal cancer (TP53 p.R213*, TP53 p.E171G and APC p.E853*) were found.

**Case 25**	M/35	**Colorectal cancer**	**Utility: Category II-3**	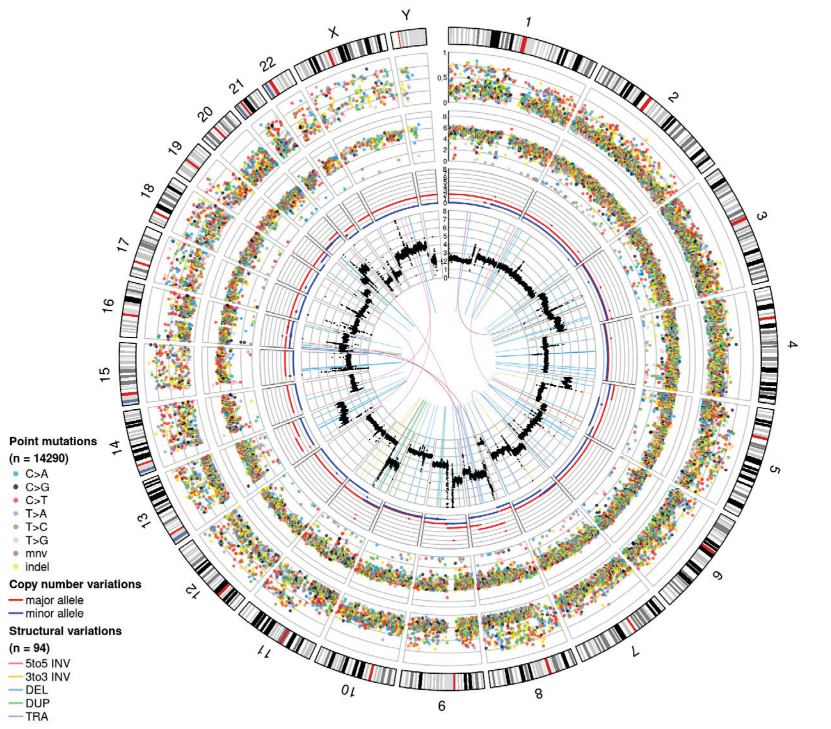
**Comments for Clinical Utility**		Mutated NRAS in colon cancer renders it resistant to EGFR-targeting antibodies such as cetuximab [Category 1-4, Drug resistance]. A germline APC frame-shift mutation was identified in a patient with a history of FAP, and the tumor DNA contained a LOH in APC, suggesting that the patient's rectal cancer could be explained by FAP in the APC gene **[Category II-3 Familial cancer]**.		
Clinical Presentation> A patient with familial adenomatous polyposis with a family history of colorectal cancer diagnosed in his father and brother.<br>A moderately differentiated adenocarcinoma of the rectum, located 6 cm from the anal verge, was identified at screening and the clinical staging was determined to be cT3N1M0. Preoperative concurrent chemoradiation was performed, followed by total protocolectomy, confirming moderately differentiated adenocarcinoma in the rectum and sigmoid colon. After surgery, mFOLFOX6 was administered as adjuvant chemotherapy, and after 8 cycles, chest CT showed a nodule with suspected metastasis in the left upper lobe. A wedge resection was performed by video-assisted thoracic surgery and metastatic adenocarcinoma from the rectum (CDX+, CK20+, CK-, TTF-1-) was confirmed and whole genome sequencing was performed. Subsequently, mFOLFOX6 was switched to palliative chemotherapy and bevacizumab was added. She has been progression free for 14 months since starting bevacizumab + mFOLFOX6.				
**Genome interpretation>**		Depth: Tissue: 49.3X, Normal: 15.0X	Total mutation counts: SNV: 12,071 Indel: 2,220 SV: 94	
		Tumor fraction: 0.68, Mean ploidy: 2.8	Germline: not specific findings	
NRAS p.G13R and TP53 p.R213Dfs* with loss of heterozygosity were identified. This patient have a frameshift germline mutation of APC gene (p.Leu540PhefsTer9) and tumor tissue had loss of zygosity at the APC gene locus.				

**Case 26**	F/48	**Endometrial carcinoma**	**Utility: Category I-2**
**Comments for Clinical Utility**	A clinical trial (cabozantinib + pamiparib, NCT05038839) is identified for participation in solid tumors with PTEN mutations **[Category I-2 Clinical trial]**.		

Clinical Presentation> A 48-year-old woman who underwent total hysterectomy bilateral salpingo-oophorectomy for endometrial cancer 2 years ago was referred to the Department of Thoracic Surgery because a chest CT revealed a cavitary nodule in the right lower lung during follow-up. Lung wedge resection was performed with findings consistent with metastasis of endometrial cancer. Histopathologic examination confirmed metastatic endometrioid adenocarcinoma from uterus (immunohistochemical stain ER+, PR+, P53+, TTF-1 -). She was treated with paclitaxel + carboplatin 6 cycle as palliative chemotherapy and is under active surveillance with no evidence of disease.

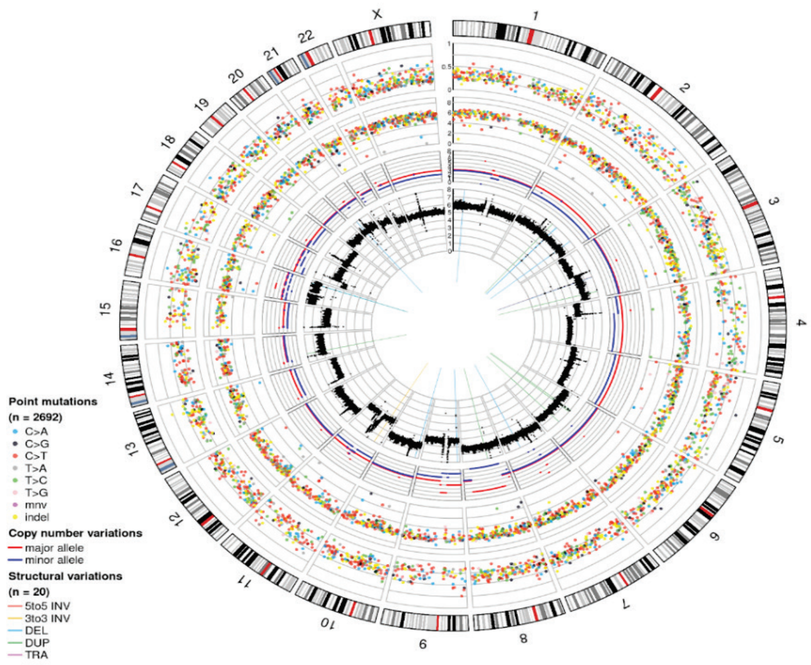

**Genome interpretation>**	Depth: Tissue: 28.1X, Normal: 13.7X	Total mutation counts: SNV: 2,073 Indel: 508 SV: 20
	Tumor fraction: 1, Mean ploidy: 5.5	Germline: not specific findings

PTEN double hit mutations (p.Ala120GlyfsTer5 and p.Thr319Ter, FGFR2 p.N550K mutation (reported 49 times in COSMIC database) and CTNNB1 hotspot mutation (p.S37F) were identified.

**Case 27**	F/40	**Colorectal cancer**	**Utility: No (Category I-1)**
**Comments for Clinical Utility**	No actionable driver was reported.		

Clinical Presentation> A 40-year-old woman with no past medical history was referred to a tertiary hospital after a colonoscopy performed for persistent constipation revealed descending colon cancer. The lumen was narrow to the point of difficulty in passage of the scope, but there was no dilatation of the proximal colon on CT.

She was diagnosed with clinical stage cT4aN1M0 colon cancer. Left hemicolectomy was performed and postoperative pathology was reported as moderately differentiated adenocarcinoma with lymphatic and perineural invasion and no venous invasion, pathological stage pT3N1a. She received 12 cycles of mFOLFOX6 as adjuvant chemotherapy and has remained relapse free for 6 years.

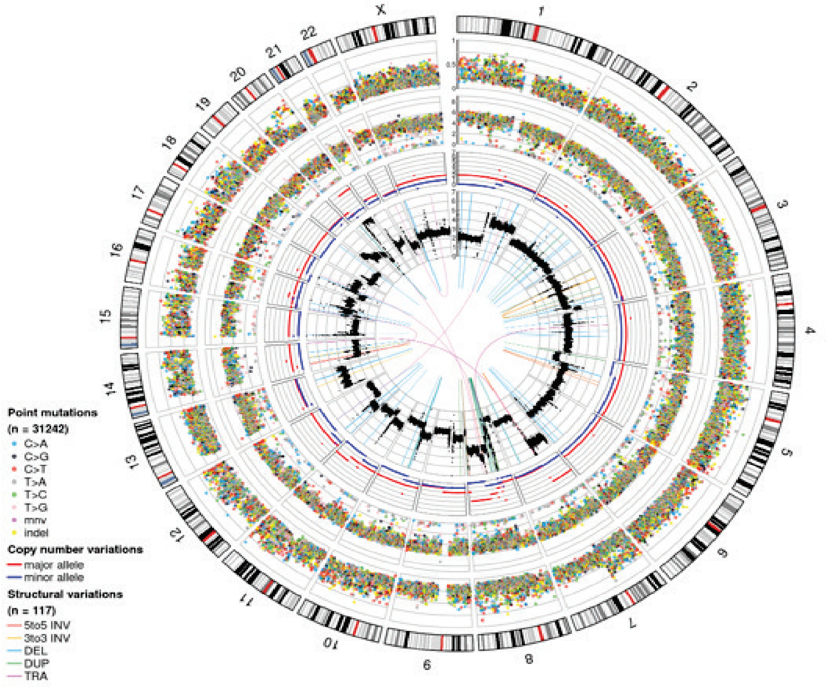

**Genome interpretation>**	Depth: Tissue: 37.0X, Normal: 15.4X	Total mutation counts: SNV: 27,401 Indel: 3,843 SV: 117
	Tumor fraction: 0.49, Mean ploidy: 2.8	Germline: not specific findings

TP53 p.Q167* and double hit APC mutations (splice donor variant and p.D1394Vfs*21) were identified.

**Case 28**	M/74	**Colorectal cancer**	**Utility: Category I-3**
**Comments for Clinical Utility**	Mutated KRAS in colon cancer renders it resistant to EGFR-targeting antibodies such as cetuximab **[Category I-3, Elimination of ineffective treatment options]**.		
Clinical Presentation> A 74-year-old man with no unusual medical history was referred to a tertiary hospital for IC valve cancer detected during a routine health check-up. Biopsy by colonoscopy confirmed adenocarcinoma moderately differentiated and was diagnosed with clinical stage cT3N1M0. A right hemicolectomy was performed, and postoperative pathology finding was reported as adenocarcinoma moderately differentiated, no lymphatic, perineural, or venous invasion, and clinical stage pT3N0. He did not receive adjuvant chemotherapy and has remained recurrence-free for 1 year.			
**Genome interpretation>**	Depth: Tissue: 22.7X, Normal: 12.8X	Total mutation counts: SNV: 2,778 Indel: 593 SV: 0	
	Tumor fraction: 0.2, Mean ploidy: 2	Germline: not specific findings	
KRAS p.G12V mutation and PIK3CA p.R115P mutation were identified.			

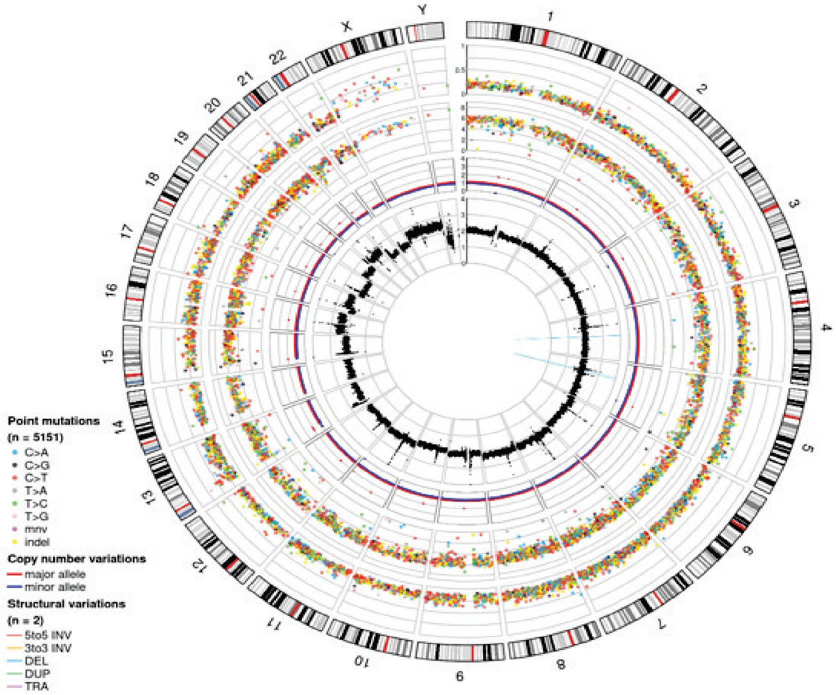

**Case 29**	M/72	**Non-smal cell lung cancer**	**Utility: Category I-2**
**Comments for Clinical Utility**	Solid cancer Patients with KRAS G12D mutation may be eligible for investigational treatments. **[Category I-2 Clinical trial, NCT06040541, NCT06179160, NCT05737706]**		

Clinical Presentation> A 72-year-old man with no significant medical history was referred for evaluation after a low-dose chest CT revealed a 2.5 cm mass in the right upper lobe. Clinical staging was confirmed as cT2N0M0. A right upper lobectomy was performed via video-assisted thoracoscopic surgery. Final pathology was reported as invasive adenocarcinoma, moderately differentiated palpillary (60%) + acinar (40%) with invasion beyond the elastic layer (PL1), pT2 N0. No adjuvant anti-cancer treatment was administered, and the patient has been followed up for one year after surgery without any signs of recurrence.

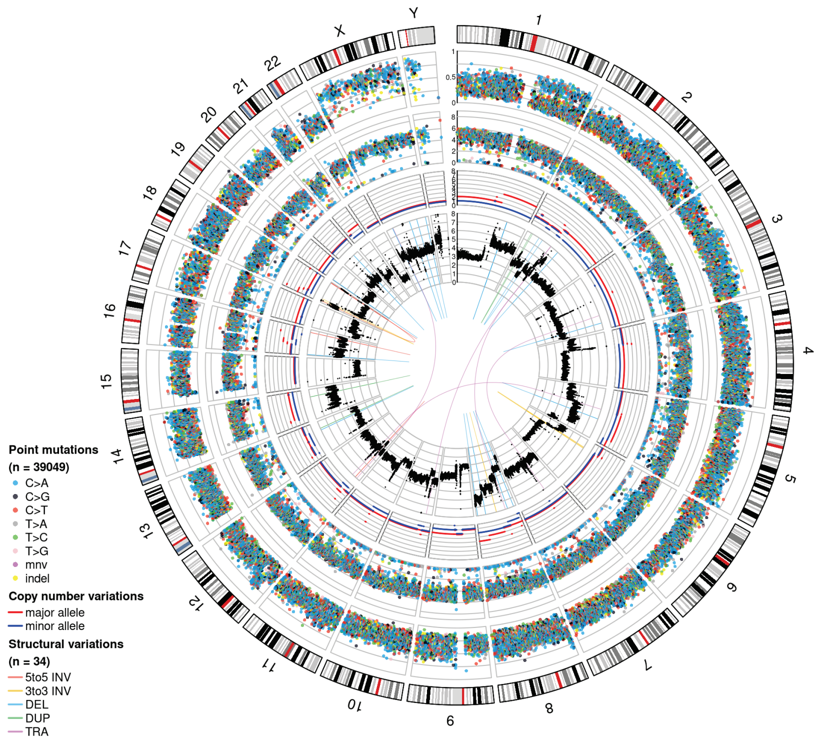

**Genome interpretation>**	Depth: Tissue: 35.0X, Normal: 16.4X	Total mutation counts: SNV: 37,139 Indel: 1,915 SV: 34
	Tumor fraction: 0.58, Mean ploidy: 3.5	Germline: not specific findings

High mutational burden and high proportion of SBS4, ID3, indicating an exposure to tobacco smoking. KRAS p.G12D and double hit mutation in STK11 gene (25.7kb-sized deletion and p.K48*) were identified.

**Case 30**	M/61	**Non-smal cell lung cancer**	**Utility: No (II-1)**
**Comments for Clinical Utility**	The patient was in a situation of consecutive failure of radiotherapy and chemotherapy, and WGS was performed with tissue obtained by EBUS biopsy after the end of the 3rd line. The results identified a p53 SNV and a CNV with relatively frequent and high copy number gain. The patient's TMB was found to be relatively low (compared to the PCAWG cohort), with 16,861 sbsititutions identified across the entire genome, resulting in a mutation rate of 5.44 mutations/MB. The pembrolizumab administered in subsequent treatments did not provide clinical benefit and was discontinued after 1 cycle. We did not get a clear answer from WGS showing resistance to chemotherapy refractoy. **[Category II-1 Drug resistance/responsive mechanisms]**		

Clinical Presentation> A 61-year-old man with an 80 pack-year smoking history presented with chest pain and sputum that had been present for a week. A chest x-ray revealed a 6.8 cm mass located in the right upper lung zone, which was confirmed by chest CT. The mass showed involvement of the right 7th rib. A CT-guided percutaneous lung biopsy was performed and confirmed moderately differentiated squamous cell carcinoma. Clinical staging was confirmed as cT4N1M0. Definitive concurrent chemoradiation was selected and paclitaxel + cisplatin was administered. Post-treatment response evaluation revealed an increase in the size of the primary lesion, confirming disease progression. Palliative chemotherapy was administered using gemcitabine and carboplatin, and after three cycles, response evaluation confirmed disease progression. Subsequently, the patient received sequential treatment with irinotecan + cisplatin, pembrolizumab, and docetaxel. However, disease progression was confirmed at the first response evaluation of each regimen. The patient passed away 16 months after diagnosis.

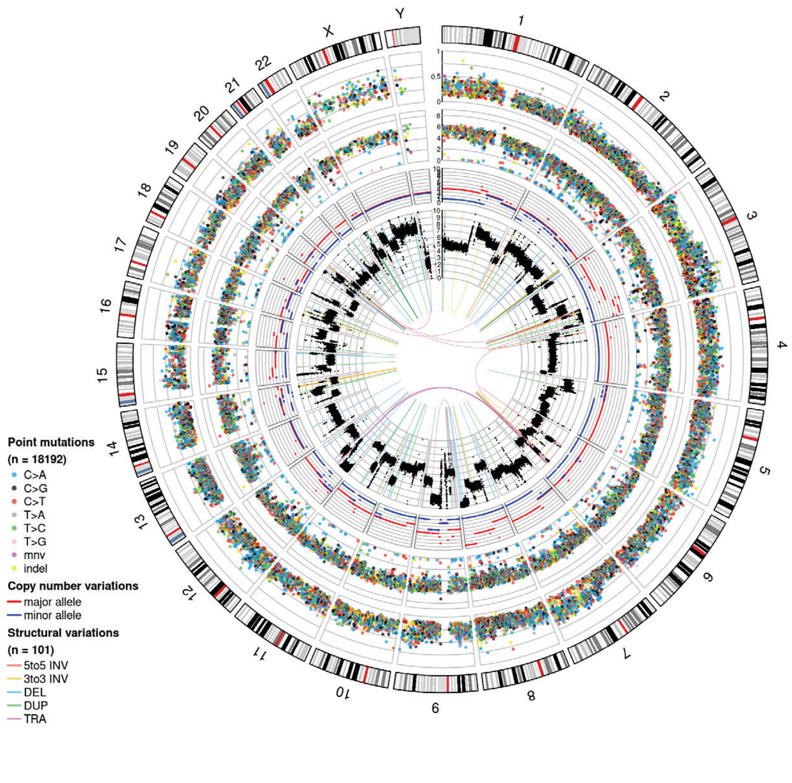

**Genome interpretation>**	Depth: Tissue: 32.9X, Normal: 14.5X	Total mutation counts: SNV: 16,861 Indel: 1,335 SV: 101
	Tumor fraction: 0.32, Mean ploidy: 5.5	Germline: not specific findings

High proportion of smoking signature (SBS4, ID3).

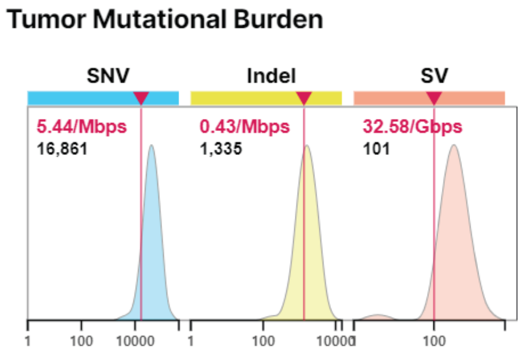

The background distributions are derived from **Squamous cell carcinoma of lung (n=47)** published by **ICGC/PCAWG**.

Case 31	M/60	Colorectal cancer	Utility: Category II-2

Comments for Clinical Utility

This case presented two clinical questions regarding the diagnosis and origin of the cancer. 1) Whether the identified stomach cancer and sigmoid colon cancer are double primary as separate cancers, or whether they are a form of stomach metastasis of sigmoid colon cancer or sigmoid colon metastasis of stomach cancer. 2) If the stomach cancer and sigmoid colon cancer are determined to be double primary, the patient has been diagnosed with three primary cancers, including prostate cancer. Is there a germline mutation that would explain the significantly higher heritability of the cancer?
WGS was performed on both gastric cancer and sigmoid colon cancer, and the two cancer genomes have completely different mutation profiles and CNV patterns, so they are considered double primary cancers. No known pathological germline mutations were identified. [Category II-2 Tumor orgin] Solid cancer Patients with KRAS G12D mutation may be eligible for investigational treatments. **[Category I-2 Clinical trial, NCT06040541, NCT06179160, NCT05737706]**

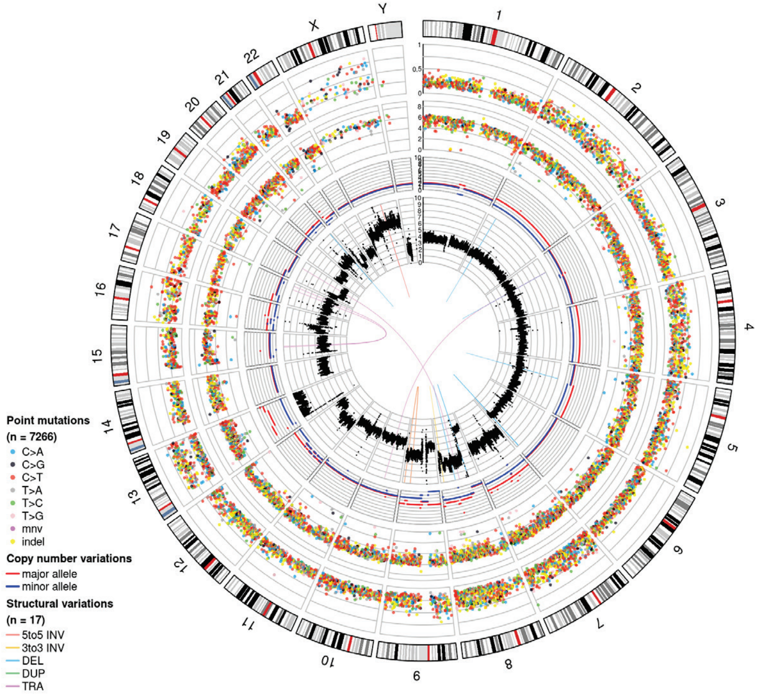

Clinical Presentation> A 60-year-old man with a history of cerebral infarction 10 years ago and prostate cancer diagnosed and operated on 1 year ago is referred for sigmoid colon cancer detected during a routine health check-up. Colonoscopic biopsy confirmed moderately differentiated adenocarcinoma and imaging confirmed lung metastases, resulting in a clinical stage of cT4N2M1. In addition, HER-2 negative localized gastric cancer (adenocarcinoma, well differentiated, cT3N0) was identified. At the tumor board for treatment planning, it was decided to perform surgery of the stomach and sigmoid colon first, followed by palliative chemotherapy. A laparoscopic low anterior resection with loop ileostomy was performed for the sigmoid colon cancer, followed by a laparoscopic distal gastrectomy with Billroth II for the stomach cancer on the same day. Postoperative pathology confirmed adenocarcinoma, moderately differentiated with focal neuroendocrine differentiation, pT3N2b, lymphovascular invasion in the sigmoid colon and stomach, body, tubular adenocarcinoma, poorly differentiated, Bormann type 3, pT3N1, with lymphovascular and perineural invasion. He received palliative bevacizumab + mFOLFOX6 for metastatic lung cancer from sigmoid colon cancer at 4 weeks postoperatively and the best response was stable disease. After 5.2 months, progression occurred with new liver lesions and second-line chemotherapy was changed to afilbercept + FOLFIRI. Two weeks after the change in chemotherapy, he was admitted to the emergency room with pneumonia and septic shock and received ventilator support, and after recovery from the pneumonia, he is no longer receiving chemotherapy, but only follow-up. He has been alive for one year since diagnosis.

**Genome interpretation>**	Depth: Tissue: 30.4X, Normal: 16.7X	Total mutation counts: SNV: 4,671 Indel: 463 SV: 44
	Tumor fraction: 0.20, Mean ploidy: 2.7	Germline: not specific findings

FGFR1 focal amplification (copy number of ~10) were identified. There were canonical driver mutations of colorectal cancer: KRAS p.G12D, APC p.E1322* and TP53 p.175H). Relatively early amplification of chr13, on which FLT3, FOXO1, and CYSTLR2 exist. KRAS G12D mutation was identified.

Supplementary Table 3

Sigmoid colon	Stomach
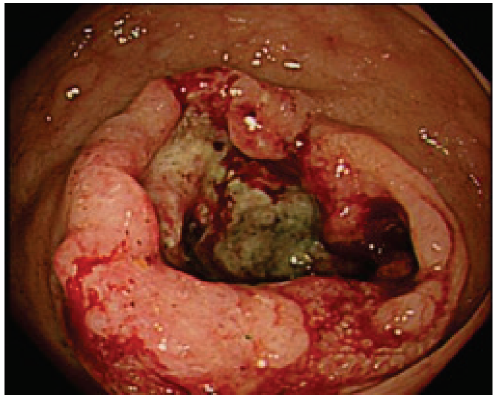 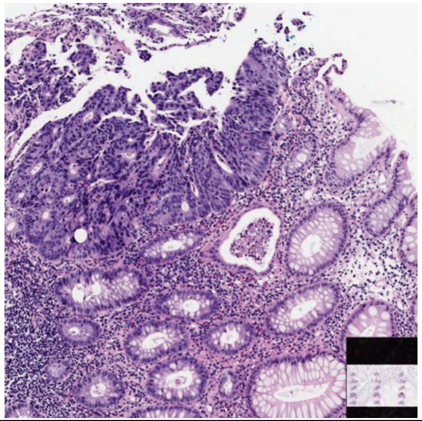	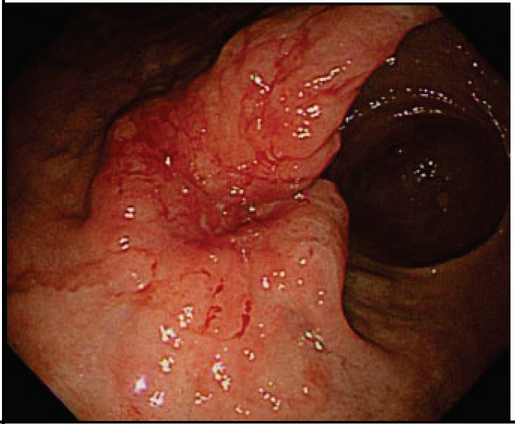 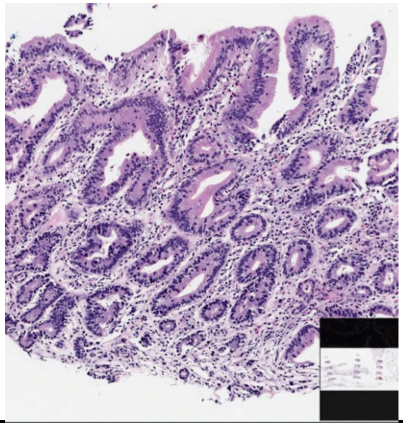
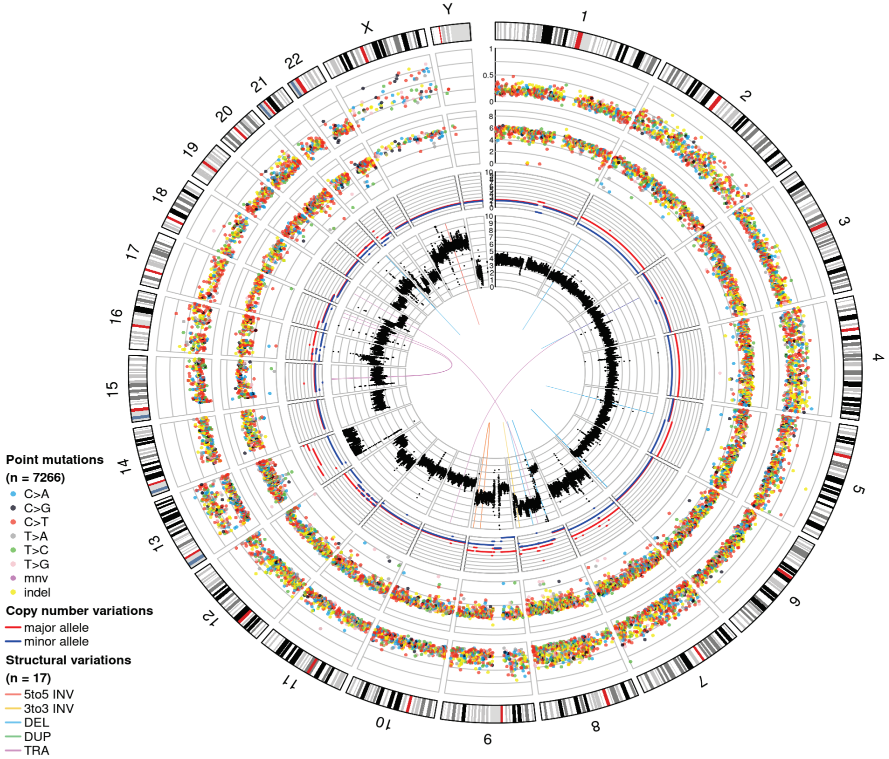	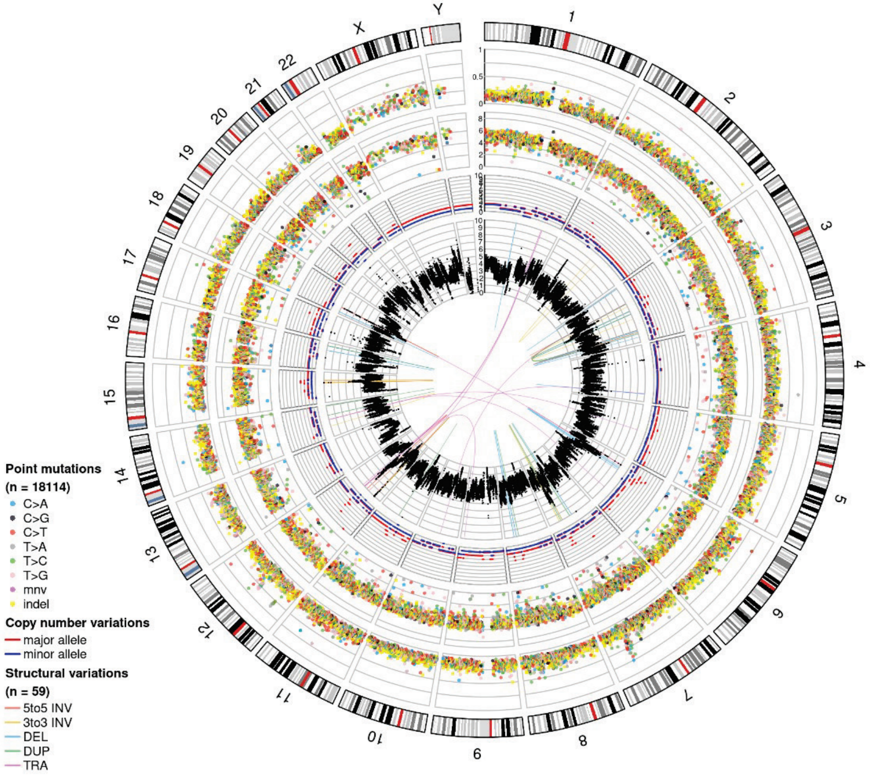

**Case 32**	F/72	**Colorectal cancer**	**Utility: No**
**Comments for Clinical Utility**	No actionable driver was reported.		

Clinical Presentation> A 72-year-old woman with no special medical history was referred to a tertiary hospital after a routine physical examination revealed an ascending crystalline carcinoma. Colonoscopic biopsy confirmed moderately differentiated adenocarcinoma and was diagnosed as clinical stage cT3N0M0. Laparoscopic right hemicolectomy was performed, and postoperative pathology findings were reported as moderately differentiated adenocarcinoma, no lymph node, perineural or venous involvement, clinical stage pT2N0. He did not receive adjuvant chemotherapy and has remained recurrence-free for 1 year.

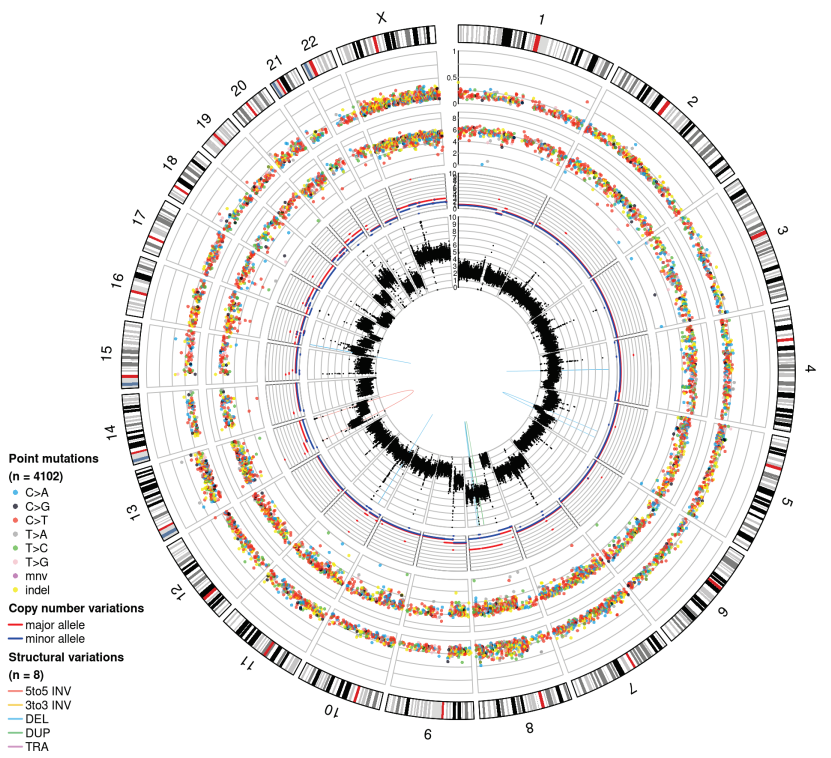

**Genome interpretation>**	Depth: Tissue: 30.3X, Normal: 18.7X	Total mutation counts: SNV: 3,457 Indel: 646 SV: 8
	Tumor fraction: 0.15, Mean ploidy: 2	Germline: not specific findings

Most of the point mutations were attributable to clock-like mutational process (SBS1, SBS5, ID1 and ID2). A focal amplification of FLT3 gene (copy number of ~14) has been identified.

<table>
<tr><td>Case 33</td><td>M/85</td><td>Colorectal cancer</td><td>Utility: Category I-2</td><td rowspan="3">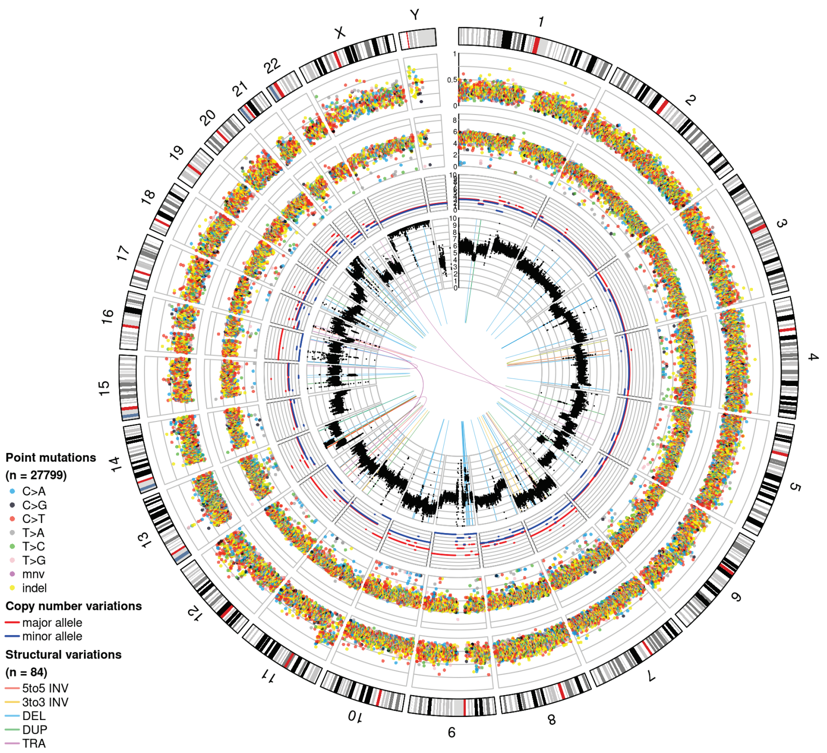
</td></tr>
<tr><td colspan="2">Comments for Clinical Utility</td><td colspan="2">NCCN guidelines recommend that the following regimens are preferred for ERBB2 amplified (HER-2 amplified) colorectal cancer: trastuzumab+pertuzumab, trastuzumab+lapatinib, trastuzumab+tucatinib, trastuzumab deruxetecan. However, there are no active clinical trials for ERBB2 mutated colorectal cancer.
[Category I-2 Clinical trial, NCT04380012, NCT 03457896, NCT04175397, NCT04579380, NCT0463919]</td></tr>
<tr><td colspan="4">Clinical Presentation> An 85-year-old man who had undergone right lower lobectomy for lung cancer 9 years ago and total gastrectomy for gastric cancer 4 years ago had suspicious findings of rectal cancer on abdominal CT performed during follow-up after gastric cancer surgery. Rectal cancer was identified at the 8 cm anal verge and histologic examination confirmed adenocarcinoma moderately differentiated. Clinical staging was cT3N1 and laparoscopic anterior resection was performed. Operative findings confirmed peritoneal seeding, but palliative surgery was performed to relieve symptoms. Postoperative pathology confirmed moderately differentiated adenocarcinoma, lymphatic, perineural and venous involvement and pathological stage was pT4N1b. The patient required palliative chemotherapy after surgery, but both the patient and guardian refused active treatment considering the patient's age and performance status, and the patient was lost to follow-up 2 months after surgery.</td></tr>
<tr><td colspan="2" rowspan="2">Genome interpretation></td><td>Depth: Tissue: 32.9X, Normal: 17.2X</td><td colspan="2">Total mutation counts: SNV: 20,160 Indel: 7,641 SV: 84</td></tr>
<tr><td>Tumor fraction: 0.4, Mean ploidy: 6</td><td colspan="2">Germline: not specific findings</td></tr>
<tr><td colspan="5">ERBB2 p.V842I mutation (report 76 times in COSMIC database) was identified. This sample has mean ploidy of 6 implying whole genome duplication.</td></tr>
</table>

Case 34	M/71	Non-smal cell lung cancer	Utility: Category II-2
**Comments for Clinical Utility**		The information from the mutational signature identified by WGS significantly helped us to asses the cancer primary. **[Category II-2 Tumor orgin]**	

Clinical Presentation> A 71-year-old man with no past medical history other than diabetes mellitus presents with a 2-month weight loss of 8 kg and is referred to a tertiary center for a 4.3 cm mass in the right adrenal gland identified on abdominal CT during workup. During workup, findings of non-functioning adrenal adenoma r/o adrenal cortical carcinoma were confirmed. The patient's unexplained anemia continued to worsen and was checked with Hb 7.6 g/dl, melena was identified, and abdominal angio-CT revealed bleeding from the jejunal mass and surgical resection was performed under the impression of lymphoma. The histopathological examination performed gave a histological diagnosis of sarcomatoid carcinoma, and although many immunohistochemical staining tests were performed, (CK(AE1/AE3): diffuse weak positive in tumor cells / CK7: focal weak positive in tumor cells / CK20: negative in tumor cells / Vimentin: diffuse strong positive in tumor cells / SATB-2: weak positive in the majority of tumor cells / CDX-2: negative in tumor cells / Calretinin, Inhibin: negative in tumor cells / PAX-8, P63: negative in tumor cells / GATA-3: focal weak positive in tumor cells / MART-1: negative in tumor cells / LCA, CD3, CD20: negative in tumor cells / TTF-1, Napsin A: negative in tumor cells). To confirm the cancer origin, chest and abdomen enhanced CT and PET-CT were performed. The imaging findings showed that the adrenal gland had increased in size to 6.8 cm, invaded the inferior vena cava, and multiple enlarged lymph nodes suggestive of lymph node metastasis in the retrocaval, retorocaval, and aortocaval spaces, and new evidence of peritoneal carcinomatosis was also confirmed. PET-CT confirmed a hypermetabolic mass in the right adrenal gland with SUVmax 14.2, multiple lymph nodes confirmed on CT, and a hypermetabolic lesion in the peritoneum. There was faint FDG uptake in multiple small nodules in both lungs, suggesting that pulmonary metastases could not be excluded, and the findings on FDG-PET-CT were highly suggestive of primary adrenal cancer. The clinical, pathologic, and radiologic findings all suggested no reasonable primary site, the possibility of metastases from renal cell carcinoma, and the possibility of a rare type of adrenal cancer. Although unlikely, adrenal metastases from primary lung cancer were also suggested as a possibility. The cancer whole genome results showed a mutational signature dominated by a smoking signature, suggesting it was more consistent with lung cancer than RCC. Finally, a diagnosis of sarcomatoid carcinoma type NSCLC was made and nivolumab + ipilimumab + paclitaxel + carboplatin was administered. The patient had a good response (partial response) with disappearance of SUV in most lesions. He has been on maintenance nivolumab + ipilimumab for 14 months without progression.

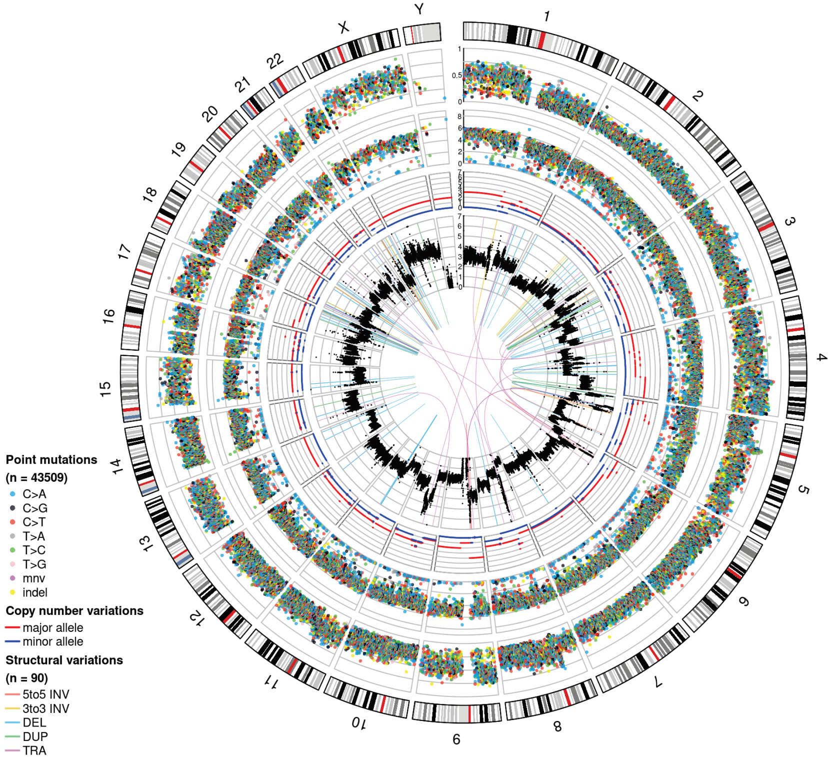

**Genome interpretation>**	Depth: Tissue: 30.4X, Normal: 14.0X	Total mutation counts: SNV: 33,876 Indel: 2,555 SV: 170
	Tumor fraction: 34, Mean ploidy: 3.8	Germline: not specific findings

Focal FGFR1 amplification was detected. High tumor mutational burden and high proportion of SBS4, ID3 signature, indicating exposure to tobacco smoking.

Case 35	M/60	Anal cancer	Utility: Category II-2
**Comments for Clinical Utility**	WGS showed a dominant APOBEC signature, consistent with HPV16 positivity, and a genomic profile more consistent with anal squamous cell carcinoma than urothelial carcinoma. **[Category II-2 Tumor orgin]**		

Clinical Presentation> A 60-year-old man with no significant past medical history was referred to a tertiary center with findings of r/o rectosigmoid colon cancer on colonoscopy performed in response to recurrent abdominal pain and constipation for one month. Although liver metastases were identified on CT, no cancer cells were identified on histology, a lumen-obstructing mass was observed on endoscopy, and palliative anterior resection was performed. Pathological examination of the surgical tissue confirmed metastatic carcinoma with squamous cell differentiation and was reported as pT4N2a. (CK7+, P63+, GATA-3+, CK20-, CDX2-, CD56-). Immunohistochemistry confirmed P16 positive and PCR confirmed HPV 16 positive. The pathology report recommended considering the possibility of metastatic squamous cell carcinoma or urothelial carcinoma. The multidisciplinary tumor board ruled out urothelial carcinoma, penile cancer, and other urinary tract tumors as unlikely, and also ruled out head and neck squamous cell carcinoma as there were no other findings to support it. It was concluded that it was anal squamous cell carcinoma, and palliative chemotherapy was decided. Palliative 5-FU + cisplatin regimen showed stable disease but liver progression after 4 cycles, nivolumab showed disease progression after only 2 cycles, and mitomycin C + 5-FU also showed disease progression at first evaluation after 3 cycles. Subsequently, paclitaxel + carboplatin, docetaxel + cisplatin + 5-FU were rechallenged, but liver progression persisted. The patient received sequential chemotherapy for 11 months after diagnosis, but showed primary resistance and the treatment plan was changed to best supportive care only.

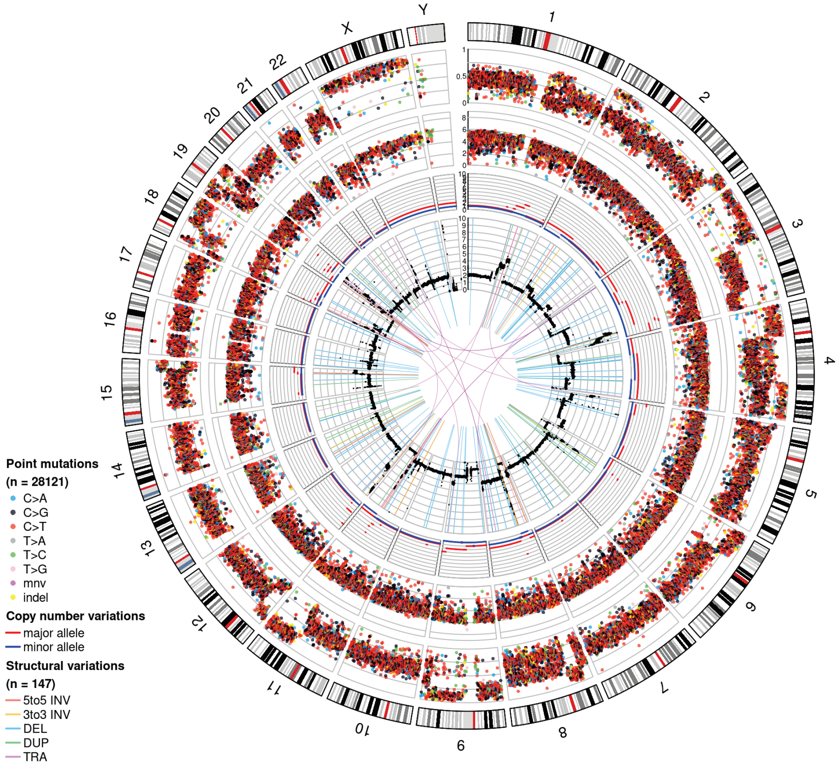

Genome interpretation>	Depth: Tissue: 45.9X, Normal: 16.3X	Total mutation counts: SNV: 26,984 Indel: 1,137 SV: 147
	Tumor fraction: 0.9, Mean ploidy: 2.3	Germline: not specific findings

Considering the high proportion of APOBEC signature (SBS2, SBS13) and paucity of canonical drivers in colorectal adenocarcinoma, this sample is more likley to be metastasis of HPV-associated anal cancer.

Supplementary Table 3

Case 36	F/45	Non-smal cell lung cancer	Utility: Category I-2
**Comments for Clinical Utility**	Solid cancer Patients with KRAS G12D mutation may be eligible for investigational treatments. **[Category I-2 Clinical trial, NCT06040541, NCT06179160, NCT05737706]**		

Clinical Presentation> A 2.5 cm lobulating nodule was identified in the left lung lobe on a chest CT performed as part of a medical examination and referred to a tertiary center. The clinical staging work up confirmed cT2aN0M0 and surgery was decided. Left upper lobe lobectomy and systemic mediastinal LN dissection were performed by video assisted thoracic surgery method. Pathologic diagnosis of the surgical tissue was reported as invasive adenocarcinoma, moderately differentiated and reported as pT2aN0, PL1. No additional adjuvant therapy was performed. The patient was followed up for 1 year after surgery and is under observation without recurrence.

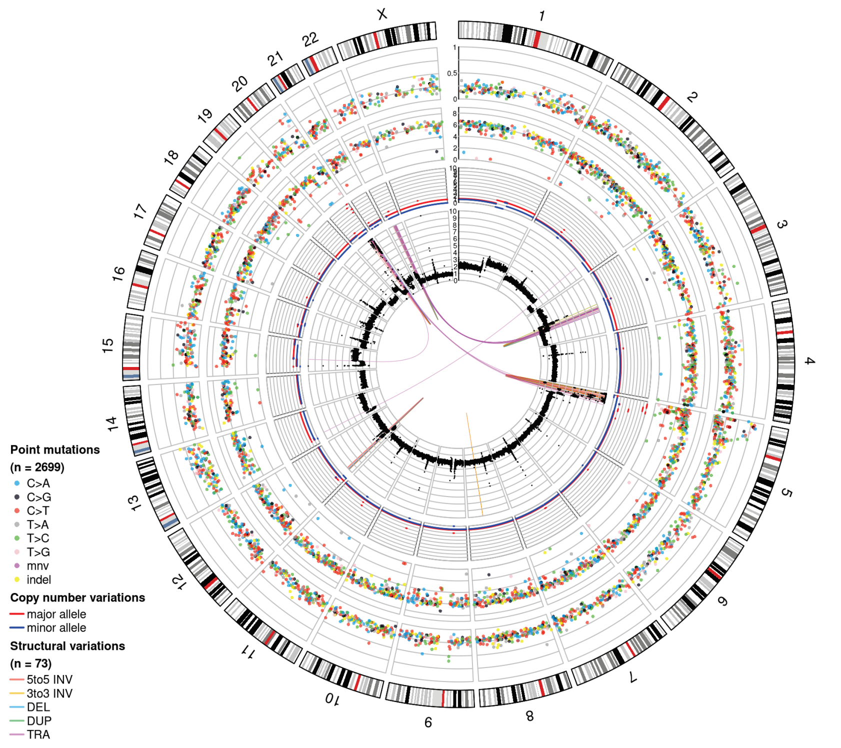

**Genome interpretation>**	Depth: Tissue: 30.6X, Normal: 16.7X	Total mutation counts: SNV: 2,290 Indel: 213 SV: 65
	Tumor fraction: 0.3, Mean ploidy: 2	Germline: not specific findings

KRAS p.G12D and STK11 p.E130Gfs* mutation with loss of heterozygosity was identified. We found TERT amplification. Considering VAF distrubution of somatic mutations in chr5 p-arm, the TERT amplification may have occured quite a long time ago.

Case 37	F/55	**Non-smal cell lung cancer**	**Utility: No (II-1)**
**Comments for Clinical Utility**		Re-biopsy was performed to identify the resistance mechanism in a patient taking afatinib, a 2nd generation TKI in EGFR mutated NSCLC. EGFR T790M mutation was confirmed by EGFR PCR by conventional method, but T790M was not confirmed by WGS. As shown in the figure, there were little cancer cells (only small areas in one out of six biopsy cores identified on the pathology slide), so the pathology report was described as "atypical cells, suggestive of adenocarcinoma" rather than "adenocarcinoma". In the WGS report, the QC criteria were met and passed, but only 306 SNVs were reported in total, and the vaf of the EGFR exon 19 deletion was low at 0.14. In a small tissue with a low purity cancer cell fraction, such as in this case, 40x WGS alone does not answer the question of resistance, and methods such as targeted enrichment or microdissection need to be supplemented. **[Category II-1 Drug resistance/responsive mechanisms]**	

Clinical Presentation> A chest CT with persistent cough and bloody sputum 3 months prior to presentation revealed a 2.6 cm central lung mass in the right lower lobe surrounding the pulmonary artery. A brain MRI revealed a focal nodular lesion in the left middle frontal lobe, a lesion that should be considered for metastasis. Clinical staging workup confirmed cT4N1M1 and EGFR exon 19 deletion, so he was started on afatinib and the confirmed brain lesion was treated with brain streotactic radiosurgery. After 62 months of afatinib without progression and a total of 4 brain streotactic radiosurgeries, the size of the main RLL increased and multiple pulmonary nodules developed. To determine the resistance mechanism, bronchoscopic biopsy was performed and tissue was obtained from the right intermedius bronchus. Along with fresh snap frozen tissue for WGS, FFPE samples were obtained for routine pathology. Pathology report was confirmed as atypical cells suggestive of adenocarcinoma and performed PANAMutyper mediated real-time EGFR PCR confirmed exon 19 deletion and T790M mutation. The patient was started on osimertinib and all small multiple pulmonary nodules disappeared and a partial response was noted. She has maintained good control of cranial and extracranial small brain metastases and no progression for 13 months.

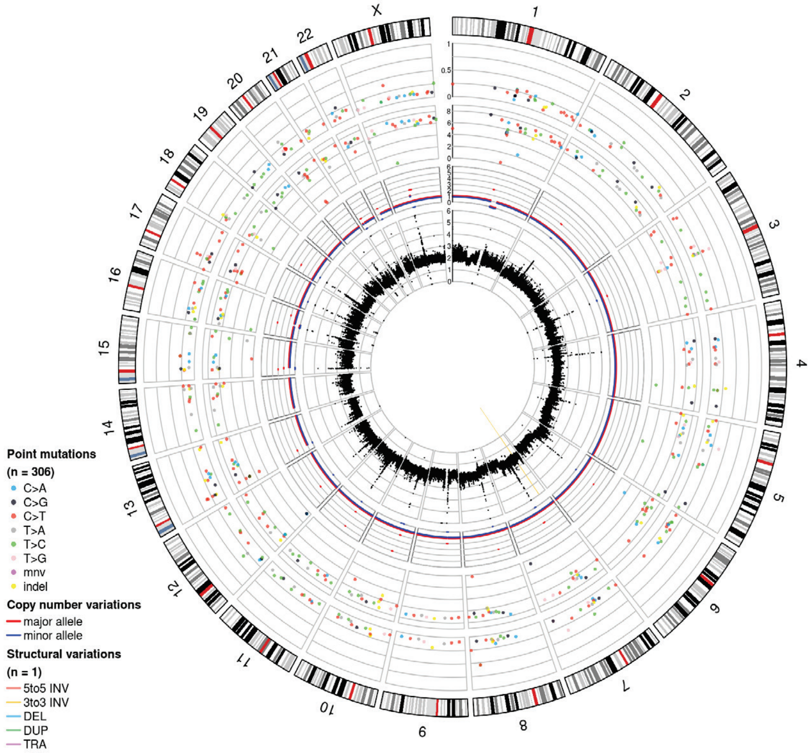

**Genome interpretation>**	Depth: Tissue: 39.4X, Normal: 19.6X	Total mutation counts: SNV: 280 Indel: 27 SV: 1
	Tumor fraction: 0.2, Mean ploidy: 1.8	Germline: not specific findings

EGFR exon 19 deletion was identified (VAF ~ 0.14).

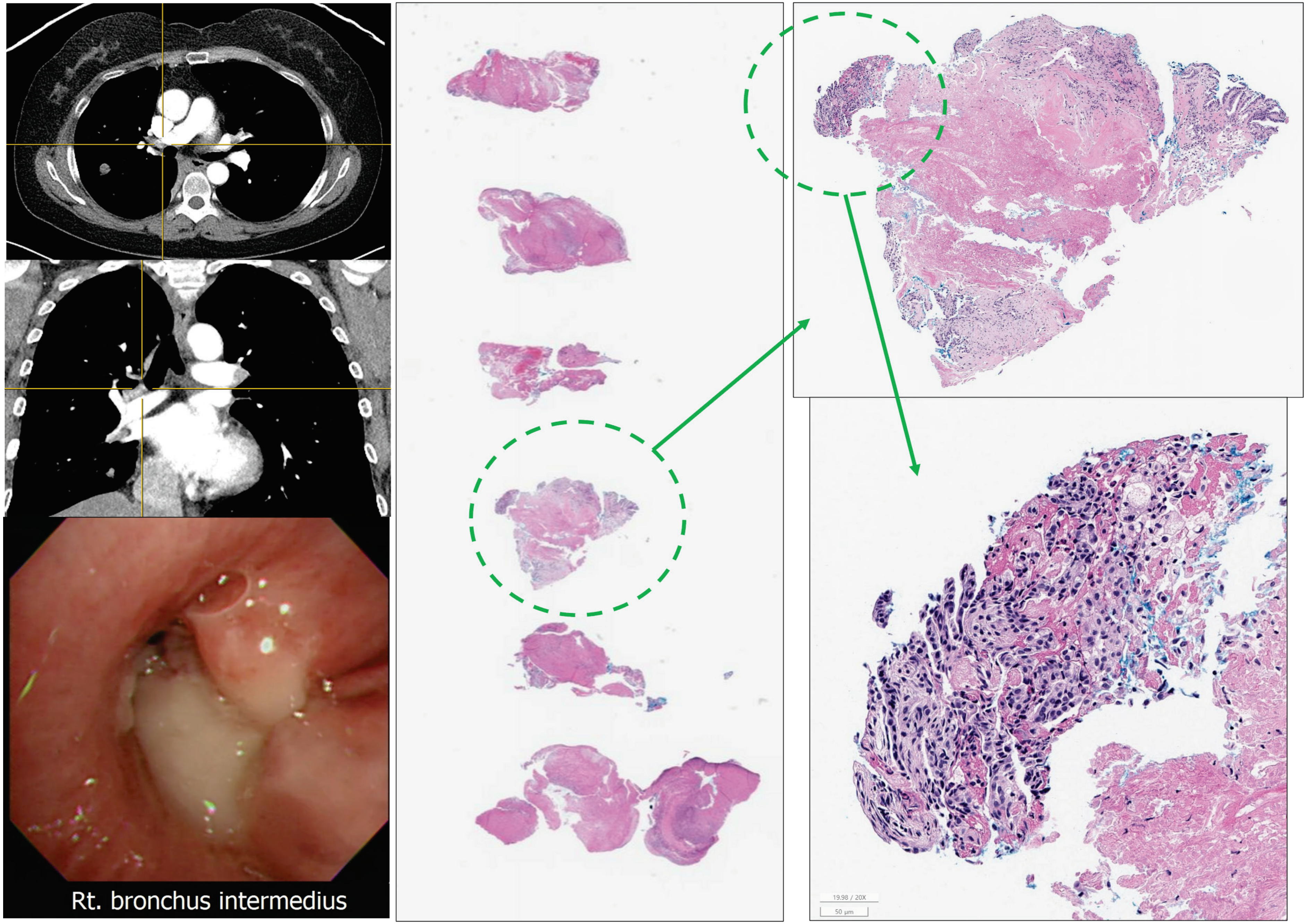
Rt. bronchus intermedius
19.98 / 20X
50 μm

**Case 38**	F/36	**Non-smal cell lung cancer**	**Utility: Category I-1**
**Comments for Clinical Utility**	ALK TKIs (alectinib, brigatinib, lorlatinib) are FDA approved in ALK rearranged NSCLC. **[Category I-1 FDA approved drug]**		

Clinical Presentation> A 2.1 cm mass with pseudocavity in the left lower lobe was identified on a chest CT performed for screening and referred to a tertiary center. The clinical staging was confirmed as cT1cN0M0 and surgery was decided. Left lower lobe lobectomy and systemic mediastinal LN dissection were performed by video-assisted thoracic surgery. Pathological diagnosis of surgical tissue was reported as invasive adenocarcinoma, moderately differentiated and reported as pT1bN0, PL0. ALK(D5F3) CDx immunohistochemical staining was reported as positive. No additional adjuvant therapy was administered. 1-year postoperative follow-up was performed and the patient is under observation without recurrence.

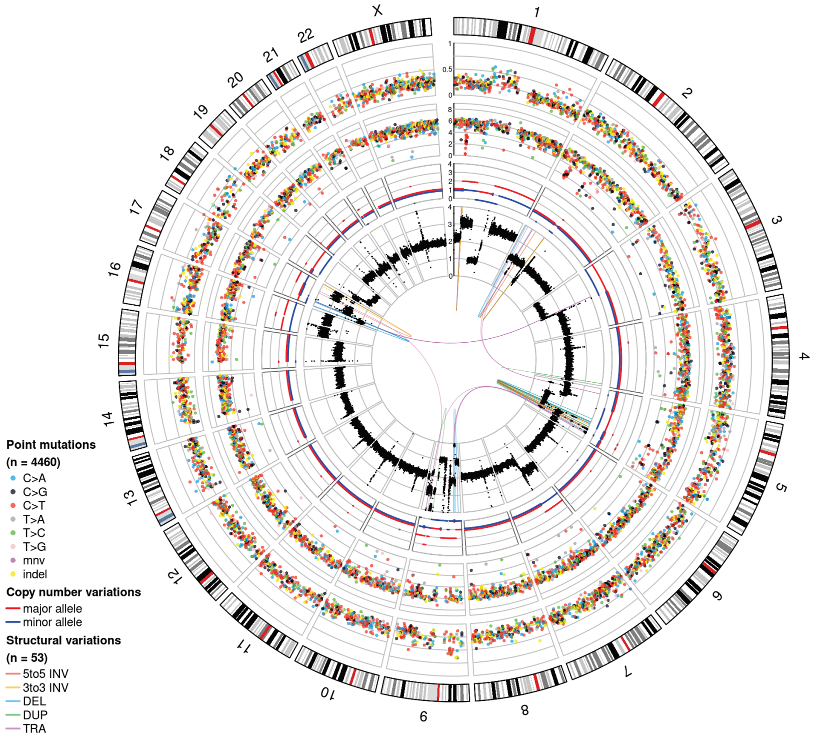

**Genome interpretation>**	Depth: Tissue: 32.5X, Normal: 21.9X	Total mutation counts: SNV: 3,717 Indel: 610 SV: 51
	Tumor fraction: 0.54, Mean ploidy: 1.9	Germline: not specific findings

EML4-ALK fusion was identified. There were double hit mutations in APC gene: APC p.S987_E988delins* and APC p.S1032*.
Mutational signature analysis revealed a proportion of APOBEC-related mutational process.

Case 39	M/54	**Renal cell carcinoma**	**Utility: No (II-1)**
**Comments for Clinical Utility**		No genomic features have been identified to explain treatment resistance or refractoriness. [Category II-1 Drug resistance/responsive mechanisms]	

Clinical Presentation> A patient who was diagnosed with clear cell renal cell carcinoma 19 years ago and underwent right radical nephrectomy, relapsed with lung metastasis 4 years ago and underwent left upper lobectomy and was lost to follow-up. 1 year ago, the left 9th rib, multiple subcutaneous metastatic nodules were identified throughout the body, and progression was confirmed in the mediastinal and cervical lymph nodes, both lungs. Two months after receiving axitinib, he developed pulmonary lymphangitic metastases and presented to the emergency department with worsening dyspnea. A core needle biopsy of a subcutaneous nodule on the abdominal wall was performed to determine the mechanism of resistance. After switching to cabozantinib, there was a partial response and improvement in pulmonary lymphangitic metastases with a reduction in overall nodule size, and the patient showed improvement in general condition and dyspnea. Eleven months later, he progressed with an increase in the size of nodules or masses throughout the body, including the lungs, and was switched to everolimus. No response was seen and he died 14 months after starting systemic therapy.

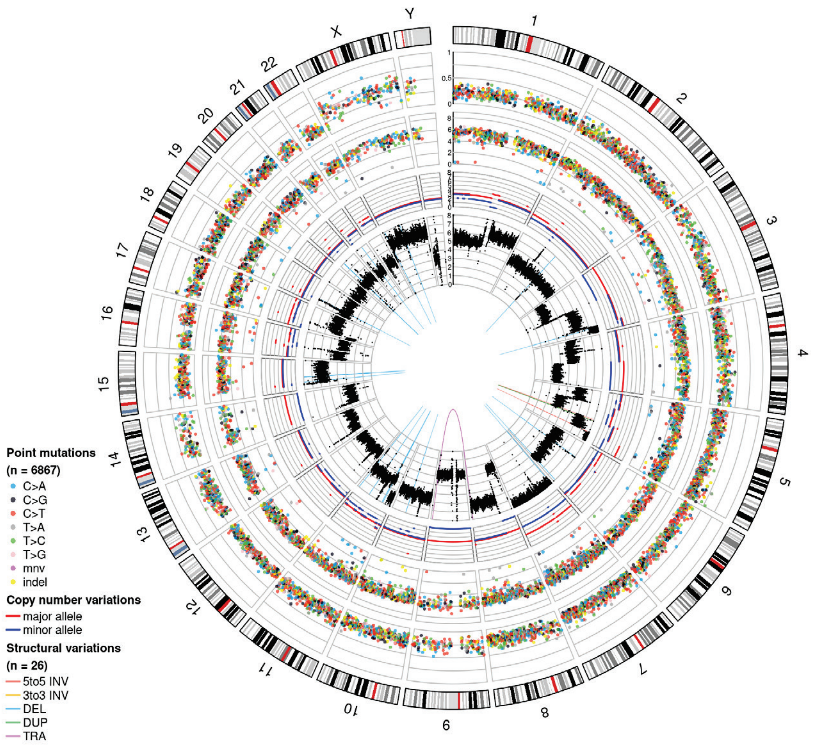

**Genome interpretation>**	Depth: Tissue: 36.3X, Normal: 22.1X	Total mutation counts: SNV: 6,274 Indel: 593 SV: 26
	Tumor fraction: 0.4, Mean ploidy: 1.8	Germline: not specific findings

TERT promotor mutation and VHL p.W88Rfs* mutation were identified.

**Case 40**	F/46	**Stomach cancer**	**Utility: Category II-1**
**Comments for Clinical Utility**	Gastric cancer with the histotype of poorly cohesive carcinoma is known to present with peritoneal metastases and have a poor prognosis. Contrary to these clinicopathologic factors, this patient had an early response to chemoimmunotherapy, confirmed a deep response, and has a very good outcome with no evidence of disease. We wanted to determine if the factors determining this good responder could be identified in the whole cancer genome. The mutational signature showed an APOBEC-related mutational process and a high mutational burden. These features are known to be associated with durable complete responses to immune checkpoint inhibitors. (J Natl Compr Canc Netw. 2020;18(5):517-521./ OncoImmunology 8, 1550341 (2019).) **[Category II-1 Drug Resistance/Response Mechanisms].**		

Clinical Presentation> A 46-year-old woman with a 1-month weight loss of 5 kg and epigastric pain was referred to a tertiary center because of findings of gastric cancer on upper GI endoscopy. Generalized hypertrophic mucosa from the fundus to the lower body, gastric curvature, anterior wall, and lesser curvature were noted, and histology was reported as adenocarcinoma, poorly differentiated. Clinical staging was cT4N2M0 and total gastrectomy with laparoscopy was planned. Operative findings revealed multiple peritoneal seeding in the diaphragm, pelvis, and multiple omentum, and surgery was terminated after biopsy only. Surgical histopathology performed on the omental nodule showed metastatic poorly cohesive carcinoma and reported a CPS of 5 on PD-L1 (28-8) PharmDx IHC. She was started on a palliative regimen of nivolumab + capecitabine + oxaliplatin. On day 4 of the first cycle, the patient presented to the emergency department with severe left upper quadrant abdominal pain occurring after a meal, and a perforation was noted near the upper body of the greater curvature. The patient underwent emergency surgery with laparoscopic simple closure. The perforation was thought to be caused by regression of the tumor involved gastric wall due to the rapid and dramatic response. After recovery, the patient was further treated with 3 cycles of chemotherapy and a partial response was achieved. The patient developed colitis, which was considered a grade 4 immune-related adverse event, and recovered with systemic corticosteroids and infliximab. After 6 months of CapeOx therapy skipping nivolumab, PET-CT confirmed a metabolic PR with residual uptake in the left gastric lymph node. The multidisciplinary tumor board decided on conversion surgery and total gastrectomy was performed. Surgical pathology result showed no redisual tumor, confirming ypT0 and only one station #3 lymph node (lesser curvature). (ypN1, 1/33) Postoperatively, 6 cycles of CapeOx therapy were performed, followed by maintenance with capecitabine monotherapy, and she has maintained no evidence of disease.

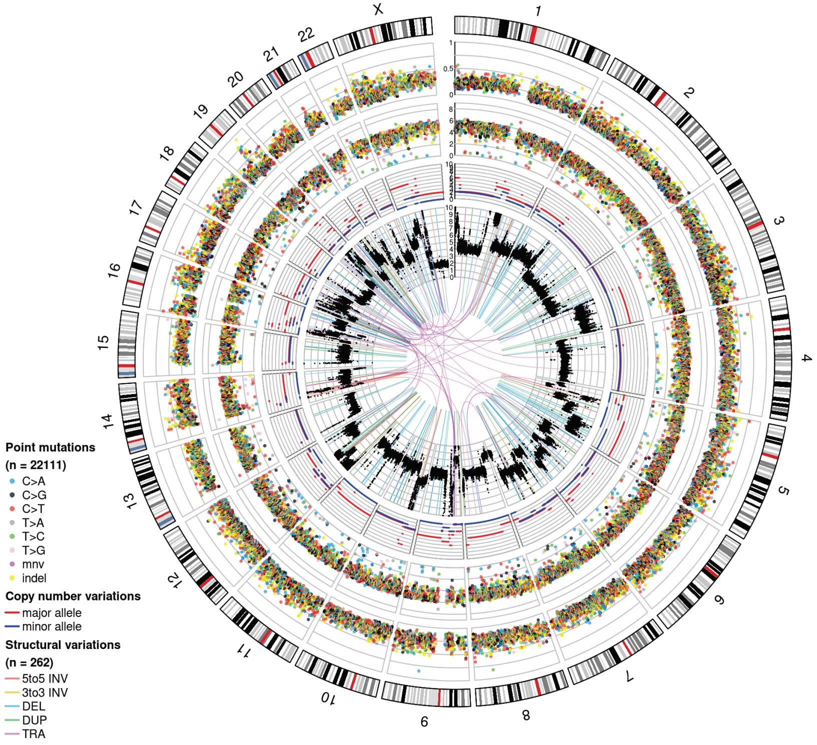

**Genome interpretation>**	Depth: Tissue: 35.6X, Normal: 22.7X	Total mutation counts: SNV: 18,620 Indel: 3,496 SV: 262
	Tumor fraction: 0.2, Mean ploidy: 4.9	Germline: not specific findings

High number of structural variations throughout the whole genome. Whole genome duplicated had occured leading to amplification of a set of oncogenes including KRAS, ERBB3, CDK4, MDM2, CCNE1.
A biallelic deletion of CDKN2A gene and TP53 p.R213* mutation with loss of heterozygosity were identified.
Mutational signature analysis revealed a proportion of APOBEC-related mutational process.

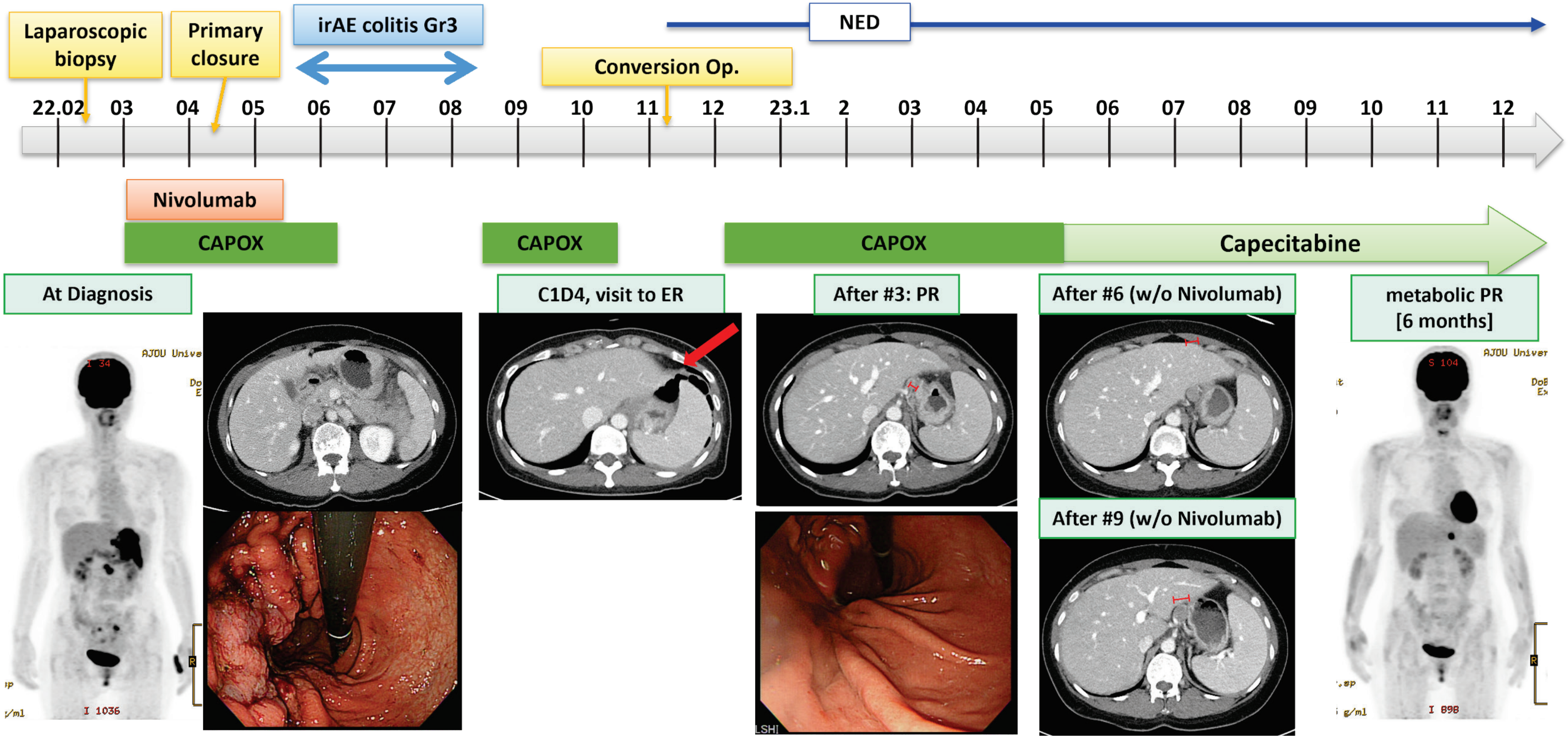

<table>
<tr><td>Case 41</td><td>F/73</td><td>Non-smal cell lung cancer</td><td>Utility: Category I-1</td><td rowspan="3">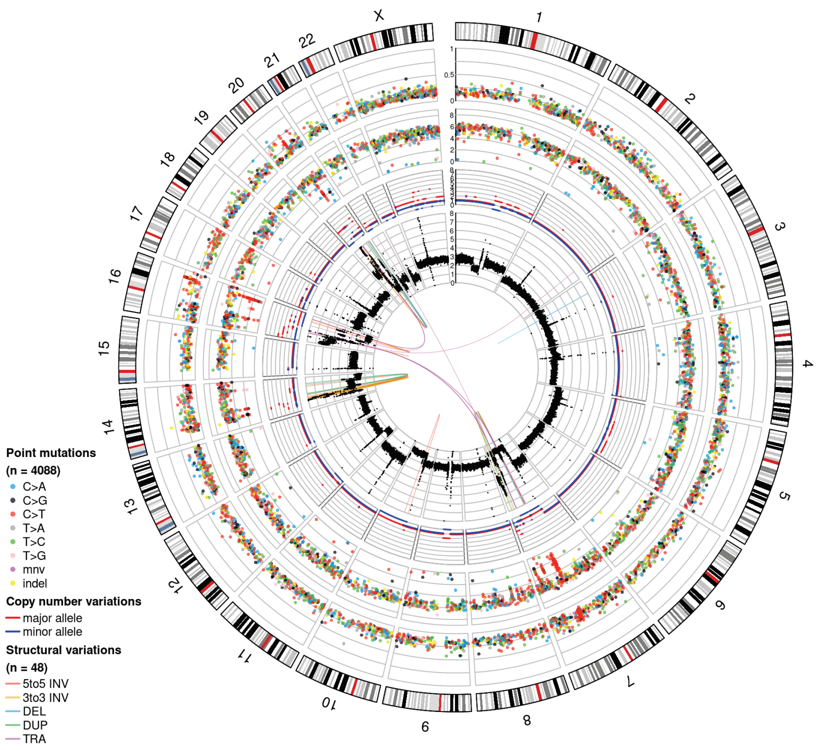
</td></tr>
<tr><td colspan="2">Comments for Clinical Utility</td><td colspan="2">EGFR L858R mutation is confirmed and EGFR TKIs (erlotinib, osimertinib) can be administered as palliative systemic therapy in NSCLC. [Category I-1 FDA approved drug]</td></tr>
<tr><td colspan="4">Clinical Presentation> After presenting to the emergency department with dizziness and testing positive for COVID-19, a chest CT revealed a 2.8 cm subpleural solid nodule at the apex of the left lung and he was referred to cardiothoracic surgery. Clinical staging confirmed cT2N0M0 and surgery was decided. Upper segmentectomy and systemic mediastinal LN dissection were performed by video-assisted thoracic surgery method. Pathological examination of surgical tissue was reported as invasive adenocarcinoma, moderately differentiated and reported as pT2N0, PL1. The patient was followed up for 1 year after surgery and is under observation without recurrence.</td></tr>
<tr><td colspan="2" rowspan="2">Genome interpretation></td><td>Depth: Tissue: 32.4X, Normal: 16.3X</td><td colspan="2">Total mutation counts: SNV: 3,517 Indel: 246 SV: 44</td></tr>
<tr><td>Tumor fraction: 0.2, Mean ploidy: 4.5</td><td colspan="2">Germline: not specific findings</td></tr>
<tr><td colspan="5">EGFR p.L858R mutation was identified.</td></tr>
</table>

**Case 42**	M/52	**Colorectal cancer**	**Utility: No (Category I-1)**
**Comments for Clinical Utility**	No actionable driver was reported.		

Clinical Presentation> A 52-year-old man with a history of hypertension presented with constipation and stool caliber changes. Colonoscopy revealed sigmoid colon cancer at 20 cm from the anal verge, and histology confirmed adenocarcinoma, moderately differentiated. The patient's father and brother had a family history of rectal cancer. The patient was diagnosed with cT4aN2 at clinical staging and underwent laparoscopic anterior resection. Postoperative pathology confirmed moderately differentiated adenocarcinoma with lymphovascular invasion and was reported as pT4N1b. The patient received 12 cycles of mFOLFOX6 as adjuvant chemotherapy for high-risk stage II sigmoid colon cancer with obstruction. He is disease free at 1 year post-operative follow-up.

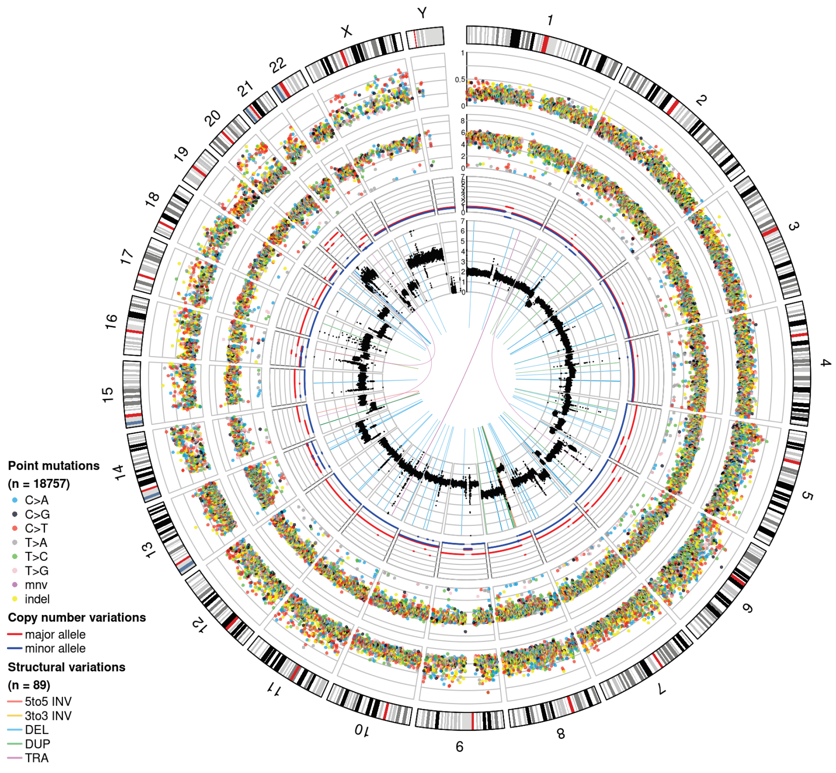

**Genome interpretation>**	Depth: Tissue: 33.7X, Normal: 15.3X	Total mutation counts: SNV: 15,291 Indel: 3,468 SV: 89
	Tumor fraction: 0.5, Mean ploidy: 2.1	Germline: not specific findings

Canonical mutations of colorectal cancer were identified: APC splice donor variant (NM_000051.4:c.7522G>T) and TP53 p.W146* with loss of heterozygosity.

**Case 43**	F/51	**Stomach cancer**	**Utility: Category I-1**
**Comments for Clinical Utility**	Pembrolizumab is indicated for the metastatic solid cancer with microsatellite high (MSI-H), including gastric cancer **[Category I-1 FDA approved drug]**		

Clinical Presentation> A patient with dyspepsia and epigastric pain for 1 month was referred to a tertiary center due to findings of gastric cancer on upper gastrointestinal endoscopy. Histologic examination of mid-body, lesser curvature was reported as poorly differentiated carcinoma. The clinical staging was cT4N2M0, and total gastrectomy with Roux-en Y was performed by laparoscopy. Pathology of the surgical specimen confirmed poorly differentiated tubular adenocarcinoma and was reported as pT3N0. No further adjuvant anticancer treatment was administered, and he is being followed for up to 10 months post-operatively for recurrence-free follow-up.

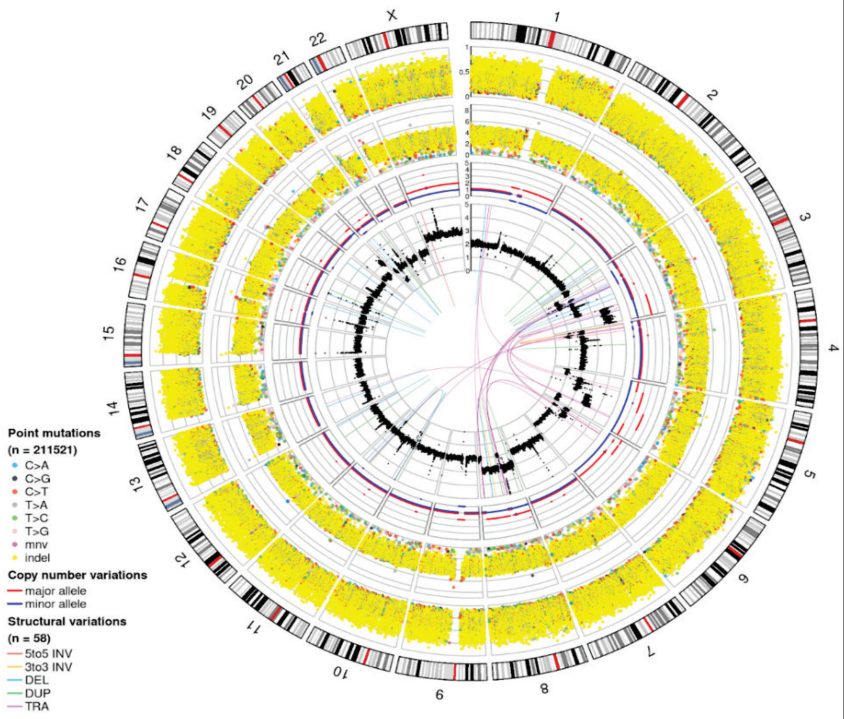

**Genome interpretation>**	Depth: Tissue: 52.6X, Normal: 23.8X	Total mutation counts: SNV: 43,929 Indel: 167,593 SV: 58
	Tumor fraction: 0.6, Mean ploidy: 2	Germline: not specific findings

This sample has deficient DNA mismatch repair pathway evidenced by high proportion of SBS44, high number of indel and MSI-score of 7.02 (cutoff 2.0). We identified a structural variant (translocation) disrupting MLH1 gene. The mutation was associated with loss of heterozygosity.

Case 44	M/74	Pancreatic cancer	Utility: No (Category I-1)
**Comments for Clinical Utility**	No actionable driver was reported.		

Clinical Presentation> A 74-year-old man presents with epigastric pain and a pancreatic mass identified on abdominal ultrasound. Abdominal CT showed a 2.4 cm mass in the neck of the pancreas, portal vein invasion and SMA abutting. EUS-guided biopsy was performed from the neck part of the pancreas and reported as adenocarcinoma. Neoadjuvant FOLFIRINOX was performed for borderline resectable pancreatic cancer. #12 cycle and the best response was confirmed as stable disease. After a multidisciplinary discussion, it was decided to give concurrent chemoradiation as an additional treatment due to the presence of SMV invasion and residual SMA abutting, making complete resection unlikely. He received 4500 cGy to the pancreas and capecitabine. A follow-up CT performed 3 months after radiotherapy revealed liver metastases and an enlarged pericardial lymph node. She was started on gemcitabine + nab-paclitaxel in the palliative setting and was hospitalized with pneumonia and septic shock after 1 cycle. The patient has since refused chemotherapy and is only being followed and has been alive for 2 years since diagnosis.

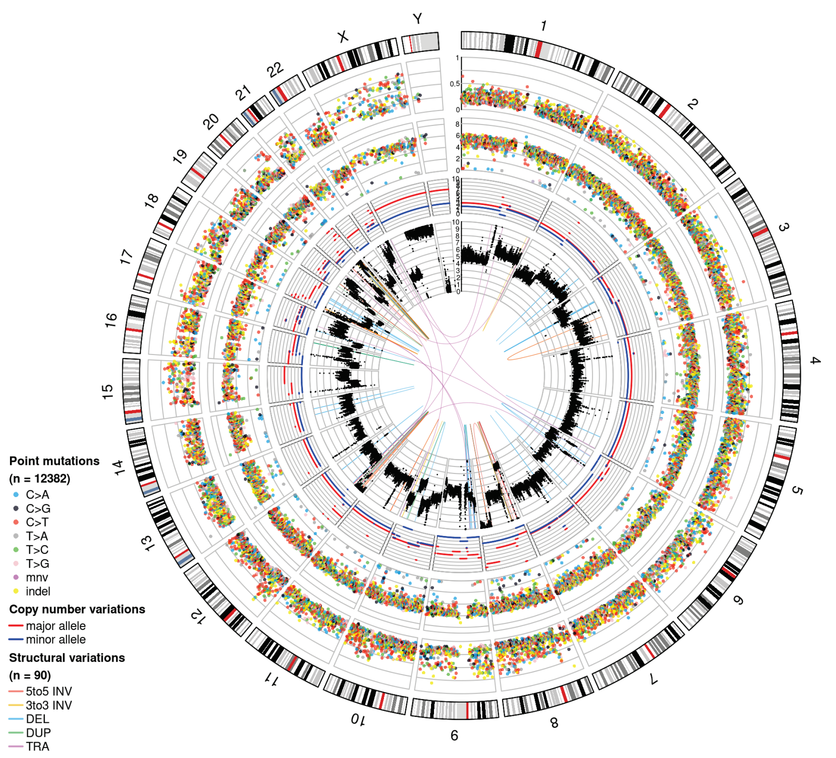

**Genome interpretation>**	Depth: Tissue: 41.9X, Normal: 19.3X	Total mutation counts: SNV: 10,135 Indel: 2,247 SV: 90
	Tumor fraction: 0.35, Mean ploidy: 5.3	Germline: not specific findings

Whole genome duplicaton with mean ploidy of 5.3. KRAS p.G12V and KRAS focal amplification (copy number of ~23) were identified.

**Case 45**	M/50	**Stomach cancer**	**Utility: Category I-1**
**Comments for Clinical Utility**	Pembrolizumab is indicated for the metastatic solid cancer with microsatellite high (MSI-H), including gastric cancer **[Category I-1 FDA approved drug]**		

Clinical Presentation> A patient with dyspepsia and melena for 1 month was referred to a tertiary center for gastric cancer on upper gastrointestinal endoscopy. Histological examination of the antrum was reported as adenocarcinoma, moderately differentiated. The clinical staging was cT4aN2M0, and distal gastrectomy with Billroth II Braun via laparoscopy was performed. Pathology of the surgical specimen confirmed tubular adenocarcinoma, poorly differentiated, and was reported as pT4aN3. Capecitabine + oxaliplatin was administered as additional adjuvant chemotherapy. After 4 cycles, grade 3 hand-foot syndrome developed and adjuvant chemotherapy was discontinued. She is being followed up for recurrence free up to 12 months after surgery.

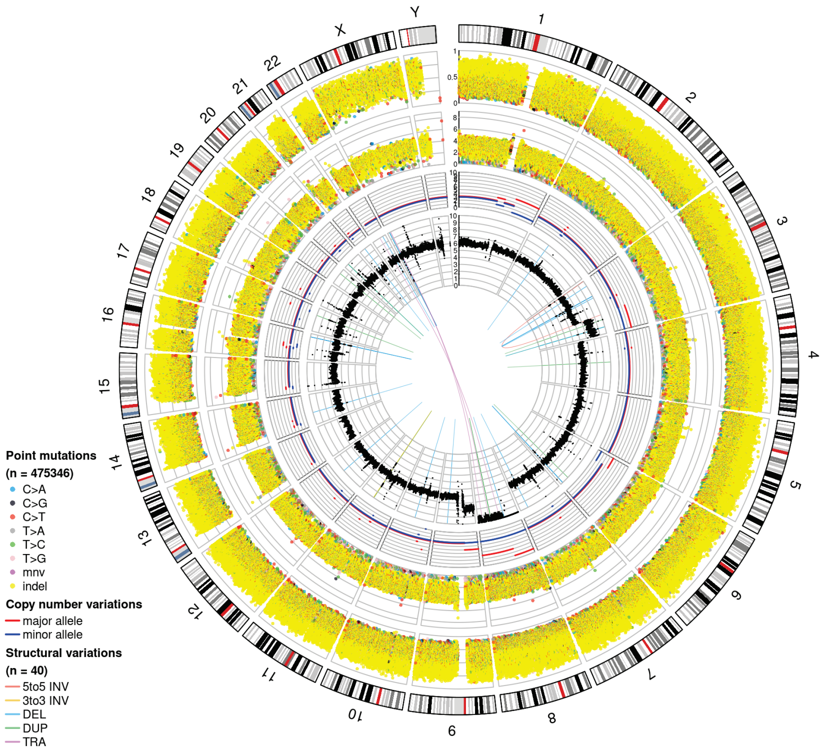

**Genome interpretation>**	Depth: Tissue: 36.7X, Normal: 17.6X	Total mutation counts: SNV: 108,278 Indel: 367,069 SV: 40
	Tumor fraction: 0.98, Mean ploidy: 6.1	Germline: not specific findings

This sample has deficient DNA mismatch repair pathway evidenced by high proportion of SBS44, high number of indel and MSI-score of 24.35 (cutoff 2.0).
FGFR1 focal amplification with copy number of ~12 was identified.

Supplementary Table 3

**Case 46**	M/72	**Small cell lung cancer**	**Utility: Category II-2**
**Comments for Clinical Utility**	A single mass was identified in the kidney during systemic chemotherapy for small cell lung cancer. As kidney metastases are known to be rare (Journal of Medical Cases. 2019;10(8):253-6.), a biopsy was performed to differentiate from the primary cancer such as renal cell carcinoma. We tried to obtain information about the cancer orgin through genomic features. In the mutational signature profile of cancer WGS, smoking-related carcinogenesis was evident, and TP53, RB1, etc., which are frequently identified alterations in small cell lung cancer, were identified. The cancer genome also showed a profile consistent with small cell lung cancer **[Category II-2 Tumor orgin].**		

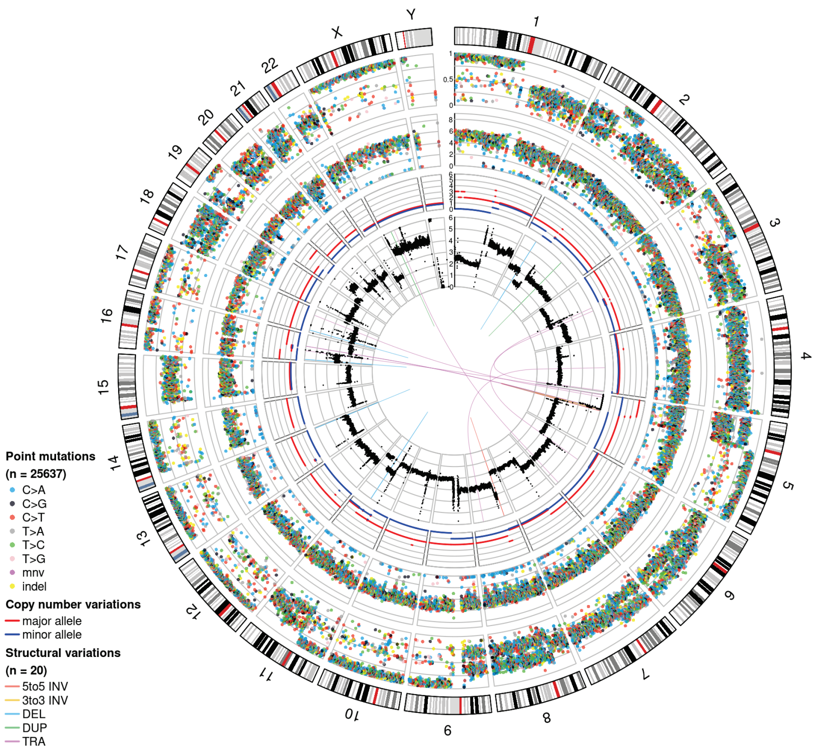

Clinical Presentation> A 71-year-old man with a 50 pack-year smoking history presented with facial edema and dyspnea. A chest CT was performed and showed a 7.9x7.5cm mass in the right upper lobe with invasion of the superior vena cava and right pulmonary artery. Small cell carcinoma was diagnosed by bronchoscopic biopsy. It was accompanied by ipilateral pleural effusion, TNM staging was cT4N3M1a, and was diagnosed as small cell lung cancer with extended disease. He was treated with a palliative regimen of atezolizumab + etoposide + carboplatin and achieved a partial response. After 6 months of systemic therapy, brain and left kidney metastases were confirmed, and after whole brain radiotherapy, the regimen was changed to irinotecan + cisplatin. The kidney biopsy confirmed small cell lung cancer and WGS was performed on this specimen. In the second cycle, he was hospitalized for proctocolitis, developed sepsis, and died.

**Genome interpretation>**	Depth: Tissue:55.2X, Normal: 16.7X	Total mutation counts: SNV: 24,207 Indel: 1,431 SV: 20
	Tumor fraction: 0.9, Mean ploidy: 2.7	Germline: not specific findings

A larger deletion in TP53 and splice donor variant of RB1 gene with loss of heterozygosity were found. High proportion of smoking signatures (SBS4, ID3) were noted.

**Case 47**	M/52	**Pancreatic cancer**	**Utility: Category I-1**
**Comments for Clinical Utility**	Olaparib is approved for pancreatic cancer with germline BRCA1/2 mutations **[Category I-1 FDA approved drug].** The patient had loss of heterozygosity on WGS and a family history of breast cancer in his aunt (sister of his father). At the time of consideration for olaparib, he had progressed and could not be maintained on a PARP inhibitor and was receiving second-line chemotherapy.		

Clinical Presentation> A 52-year-old man with a history of hospitalization for acute pancreatitis 10 months ago and an enlarging mass in the body of the pancreas during follow-up presented to a tertiary center. Imaging showed a 3.8 cm mass and regional lymph nodes from the body of the pancreas to the tail, and multiple metastases in the liver.EUS-guided biopsy was performed from a body part of the pancreas and reported as adenocarcinoma, moderately to poorly differentiated.The patient's aunt had a history of breast cancer. Palliative modified FOLFIRINOX was planned for metastatic pancreatic cancer and a partial response was achieved. A CT scan performed after 10 cycles showed an increase in the size of the main pancreatic mass, and subsequent therapy was gemcitabine + nab-paclitaxel, but disease progression occurred after only 2 cycles. Liposomal irinotecan + 5-FU + leucovorin therapy was performed, but symptomatic brain metastases were confirmed after 1 cycle and whole brain radiotherapy was performed. Palliative radiotherapy to the pancreatic bed was then performed. Palliative gemcitabine + cisplatin was administered without response and new cardiac metastases were identified. The patient was placed on best supportive care and died 13 months after diagnosis.

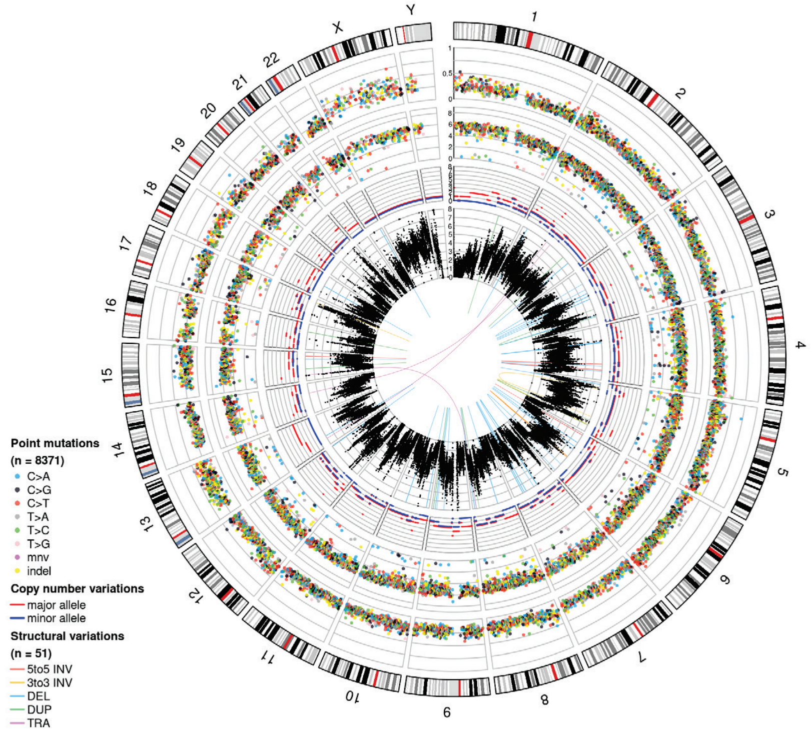

**Genome interpretation>**	Depth: Tissue: 35.5X, Normal: 20.2X	Total mutation counts: SNV: 7,192 Indel: 1,180 SV: 51
	Tumor fraction: 0.2, Mean ploidy: 2.3	Germline: not specific findings

PIK3CA p.H1047R, KRAS p.G12V and TP53 p.P250L were identified.
Mutationa signature analysis showed high proportion of SBS3, ID6 signature indicating homologous recombination deficiency. The HRD score was 0.87 (cutoff 0.7).
This patient had a germline BRCA2 mutation (p.Asn863LysfsTer18) with loss of heterozygosity.

**Case 48**	F/54	**Colorectal cancer**	**Utility: Category I-2**
**Comments for Clinical Utility**	Solid cancer Patients with KRAS G12D mutation may be eligible for investigational treatments. **[Category I-2 Clinical trial, NCT06040541, NCT06179160, NCT05737706]**		

Clinical Presentation> A 54-year-old woman with type 2 diabetes mellitus and alcoholic liver disease presented with hematochezia, and a colonoscopy performed for hematochezia revealed a 20 cm area of squamous cell carcinoma at the anal verge, and histologic examination confirmed well to moderately differentiated adenocarcinoma. At clinical staging, it was diagnosed as cT3N1 and laparoscopic anterior resection was performed. Postoperative pathology confirmed moderately differentiated adenocarcinoma, perineural invasion, and was reported as pT4N0. The patient received 12 cycles of mFOLFOX6 as adjuvant chemotherapy for stage III sigmoid colon cancer. He is disease free at 1 year postoperative follow up.

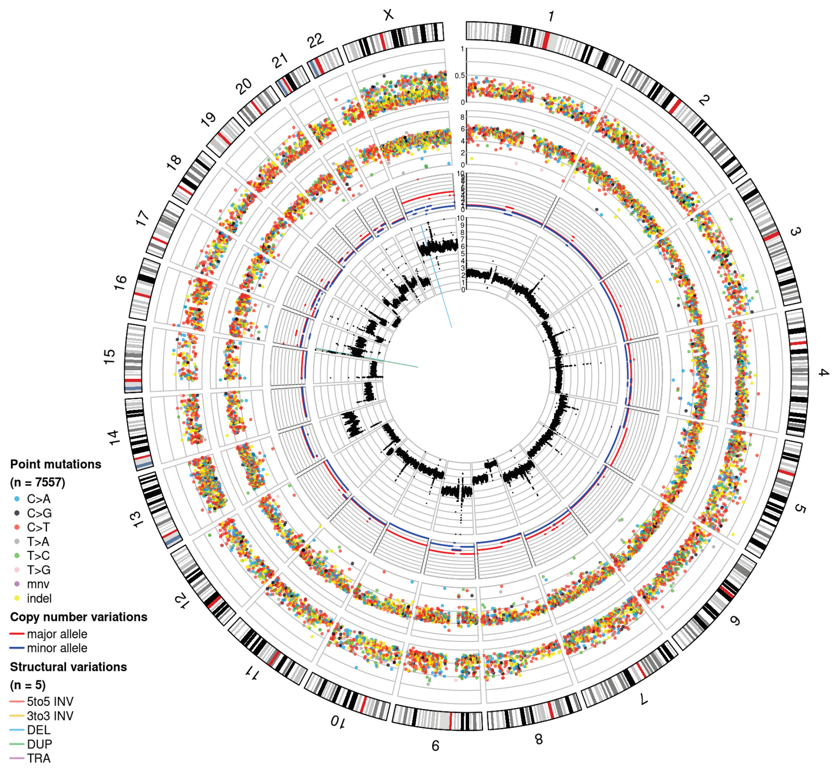

**Genome interpretation>**	Depth: Tissue: 33.6X, Normal: 16.8X	Total mutation counts: SNV: 5,736 Indel: 1,375 SV: 5
	Tumor fraction: 0.25, Mean ploidy: 4.2	Germline: not specific findings

Canonical drivers in CRC: KRAS p.G12D, TP53 splice donor variant, APC p.E1464Vfs* mutations.

**Case 49**	M/51	**Colorectal cancer**	**Utility: Category I-2**
**Comments for Clinical Utility**	Solid cancer Patients with KRAS G12D mutation may be eligible for investigational treatments. **[Category I-2 Clinical trial, NCT06040541, NCT06179160, NCT05737706]**		

Clinical Presentation> A 51-year-old man, an HBV carrier, presented with hematochezia and stook's caliber change and underwent colonoscopic stent insertion for adenocarcinoma at 18 cm of the anal verge due to obstruction. Histologic examination confirmed adenocarcinoma moderately differentiated. At clinical staging, it was diagnosed as cT3N1 and laparoscopic anterior resection was performed. Postoperative pathology confirmed moderately differentiated adenocarcinoma, venous invasion, and was reported as pT3N0. The patient received 12 cycles of mFOLFOX6 as adjuvant chemotherapy for high risk stage II sigmoid colon cancer with osbstruction. He is disease free at 1 year postoperative follow up.

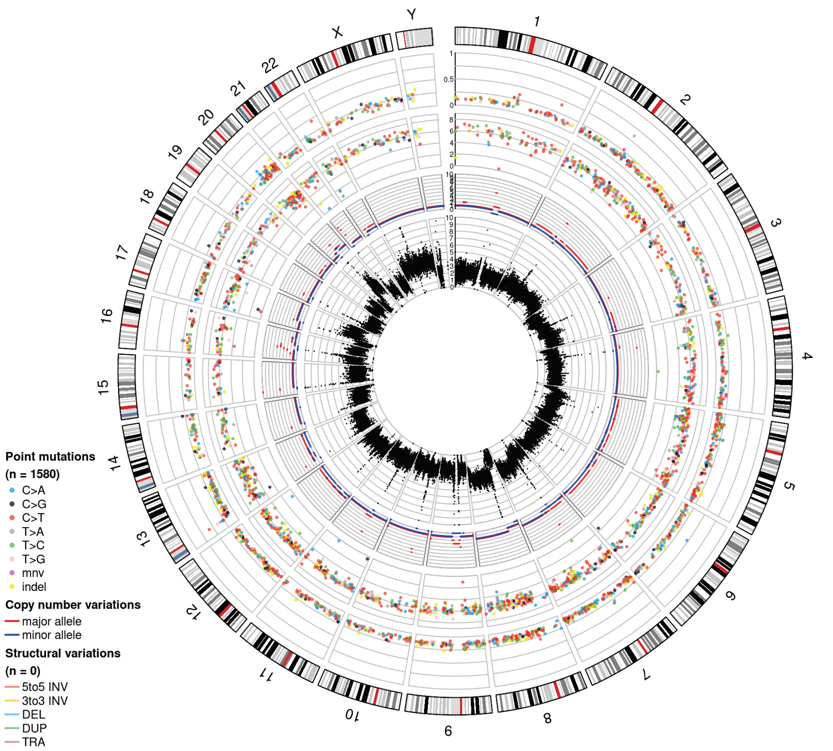

**Genome interpretation>**	Depth: Tissue: 35.8X, Normal: 20.0X	Total mutation counts: SNV: 1,429 Indel: 151 SV: 0
	Tumor fraction: 0.1, Mean ploidy: 2	Germline: not specific findings

KRAS p.G12D and APC p.S1315Qfs* mutation were identified.

**Case 50**	F/83	**Urothelial carcinoma**	**Utility: Category I-1**
**Comments for Clinical Utility**	Erdafitinib is approved for bladder cancer with FGFR3 mutation **[Category I-1 FDA approved drug]**.		

Clinical Presentation> An abdominal CT performed in an 83-year-old woman presenting with painless hematuria identified on physical examination confirms the findings of bladder cancer. The clinical staging was cT2N0M0. A transurethral resection was performed, which revealed a papillary and nodular mass and confirmed high-grade papillary urothelial carcinoma (pT1) with invasion from the bladder base to the lamina propria. The patient was followed up with urine cytology and abdominal CT in an outpatient setting, and a cystoscopic examination was performed 1 year later, and there was no evidence of recurrence in all examinations up to 1 year later.

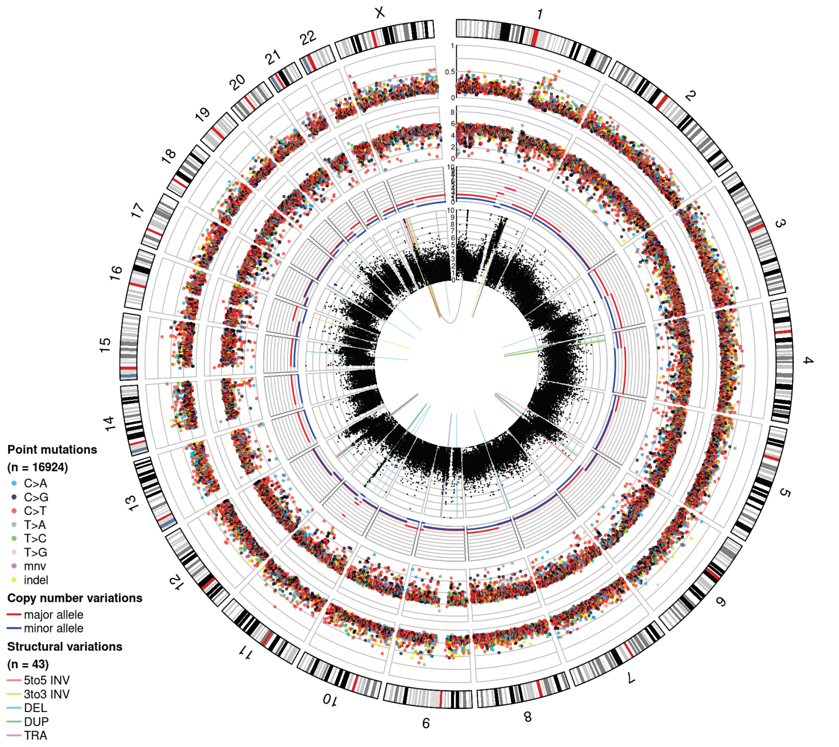

**Genome interpretation>**	Depth: Tissue: 6.0X, Normal: 30.4X	Total mutation counts: SNV: 16,169 Indel: 757 SV: 43
	Tumor fraction: 0.3, Mean ploidy: 2.9	Germline: not specific findings

FGFR3 p.G370C mutation was identified. The MYCL gene was focally amplified with copy number of ~9.
Mutational signature analysis revealed a high proportion of APOBEC-related mutational process.

Case 51	F/58	Pancreatic cancer	Utility: No (Category I-1)
**Comments for Clinical Utility**	No actionable driver was reported.		

Clinical Presentation> A 58-year-old woman presents with left upper quadrant abdominal pain and imaging studies show evidence of pancreatic cancer. Workup revealed a 3.6 cm mass in the tail of the pancreas and 4.1 cm metastases in the S5 liver. Ultrasound-guided core-needle liver biopsy confirmed adenocarcinoma. First response evaluation after cycle 4c confirmed progression in liver metastases and was switched to gemcitabine + nab-paclitaxel regimen; response evaluation after cycle 2 confirmed increase in mass size in liver metastases but was in stable disease category; imaging after cycle 4 confirmed progression. Liposomal irinotecal + 5-FU + leucovorin regimen was started and after 2 cycles disease progression was confirmed with increased size of liver metastases and peritoneal seeding. She underwent liver biopsy to identify actionable alterations on additional therapeutic agents and was enrolled in a WGS study. Subsequently, the patient's phrenic pain worsened with a metastatic mass in the right flank, and palliative radiotherapy was performed. A patient with chemotherapy-refractory pancreatic cancer with primary resistance to three lines of standard pancreatic cancer chemotherapy died 7 months after diagnosis.

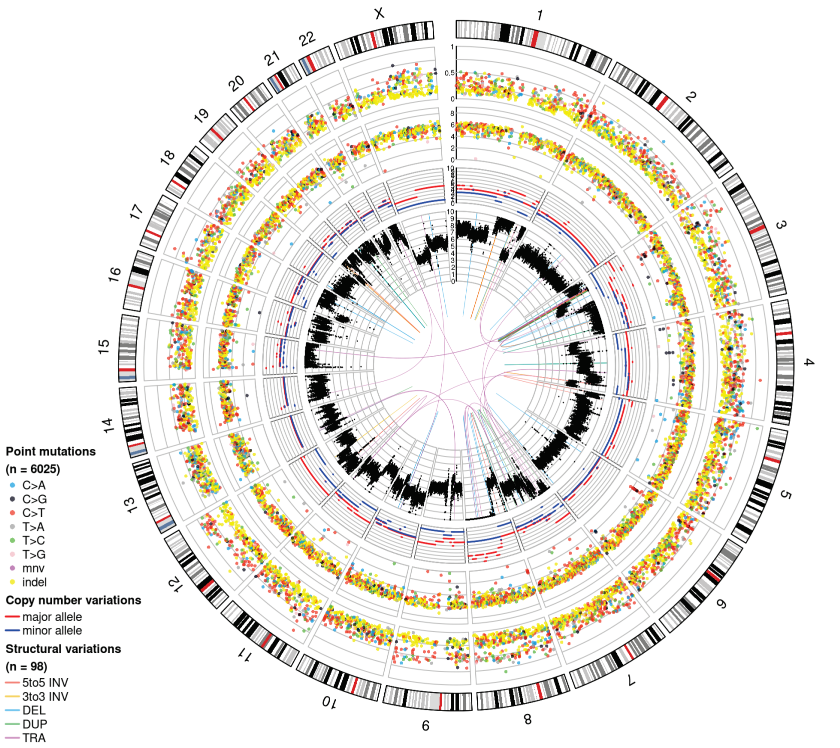

**Genome interpretation>**	Depth: Tissue: 41.9X, Normal: 15.7X	Total mutation counts: SNV: 3,567 Indel: 2,460 SV: 98
	Tumor fraction: 0.72, Mean ploidy: 7.4	Germline: not specific findings

Whole genome duplication with mean ploidy of 7.4 was noted. Considering the variant allele frequency distribution, most of the chromosomal gain appeared to had occured early in the tumorigenesis.

Case 52	M/67	Head and Neck cancer	Utility: Category I-1
**Comments for Clinical Utility**	In HER-2-positive salivary gland cancer, HER-2-targeted therapies (trastuzumab, trastuzumab emtansine, pertuzumab, tastuzumab deruxtecan) have a category 2A recommendation in the NCCN guideline and are considered standard of care.		

Clinical Presentation> A 67-year-old man presents with a palpable mass in the right neck. Examination revealed a 3 to 4 cm mobile, firm mass at the right level II. Core needle biopsy confirmed metastatic adenocarcinoma compatible with salivary duct carcinoma. A wide excision and right modified radical neck dissection was performed under the impression of a submandibular gland tumor. Pathological examination of the surgical tissue confirmed salivary duct carcinoma, pT1N2bM0 and C erb B2 IHC positive. Postoperative concurrent chemoradiation (60Gy/30fx) to the right neck was performed. She is followed up with recurrence free status at 1 year postoperatively.

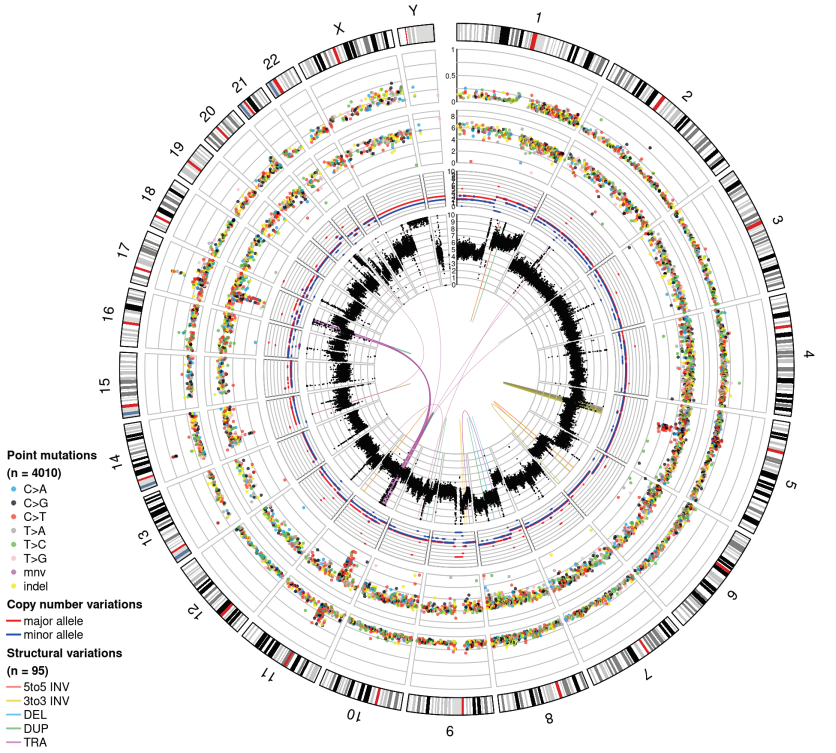

**Genome interpretation>**	Depth: Tissue: 47.4X, Normal: 19.0X	Total mutation counts: SNV: 43,471 Indel: 542 SV: 95
	Tumor fraction: 0.15, Mean ploidy: 5	Germline: not specific findings

ERBB2 p.D769H mutation and ERBB2 focal amplification with a copy number of ~31 were identified.

Case 53	M/58	Urothelial carcinoma	Utility: Category I-1
**Comments for Clinical Utility**	There are eligible trials for solid tumors with HRAS mutations **[Category I-2 Clinical trial, NCT01374789, NCT04284774].**		

Clinical Presentation> A 58-year-old man presents with a 1-month history of painless gross hematuria and a bladder mass on ultrasound. An abdominal CT confirmed the findings of bladder cancer. The clinical staging was cT2N0M0. Transurethral resection was performed to remove a 3 cm bladder mass near the right ureteral orifice. Pathology confirmed high-grade papillary urothelial carcinoma with lamina propria invasion (pT1). He was followed up with urine cytology and abdominal CT, and cystoscopy was performed every 6 months, but there was no evidence of recurrence for 13 months after surgery.

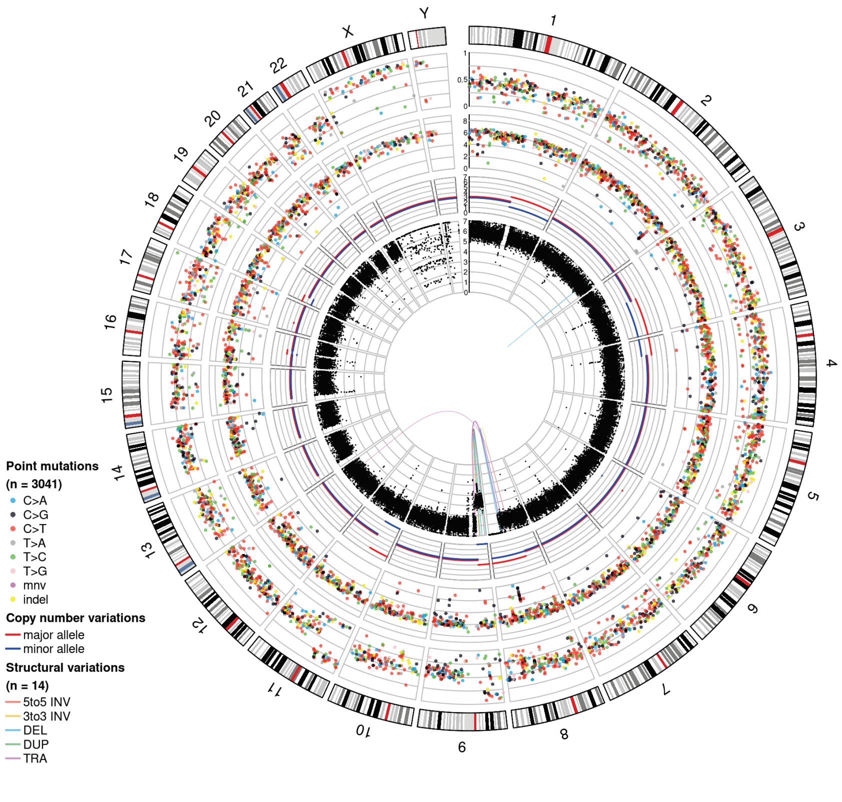

**Genome interpretation>**	Depth: Tissue: 4.57 X, Normal: 21.32X	Total mutation counts: SNV: 2,782 Indel: 260 SV: 14
	Tumor fraction: 0.32, Mean ploidy: 2.3	Germline: not specific findings

TERT promoter mutation and HRAS p.Q61K mutation were identified. Mutational signature analysis revealed a high proportion of APOBEC-relatated mutational process.

Case 54	F/42	**Breast cancer**	**Utility: Category I-1**
**Comments for Clinical Utility**	Tastuzumab is approved for erbb2 amplification in metastatic breast cancer. PIK3CA H1047R is approved for alpelisib in HER-2 negative and hormone positive breast cancer.		

Clinical Presentation> A 42-year-old woman presented with a growing lump in the right breast since 6 months, and a 9 cm irregularly shaped huge mass with skin invasion was identified, and a metastatic lymph node was identified at the right axillary levels 1 and 2. Histological examination revealed invasive ductal carcinoma, ER/PR/Her-2 8+/6+/0 ki-67 >90%, and clinical staging was confirmed as cT4dN2M0. He received adrimycin + cyclophosphamide (AC) followed by docetaxel as neoadjuvant chemotherapy. A partial response was achieved after AC therapy, but preoperative evaluation revealed bone metastases at C3, T1, T3, and T4 of the spine. Due to neck pain, palliative radiotherapy was initially administered. Re-biopsy was performed for clinical trial enrollment and confirmed subtyping to be HER-2+HR+ with ER/PR/Her-2: 7/2/2+ SISH positive. At this point, she underwent breast biopsy to identify actionable alterations for additional therapeutic agents and was enrolled in the WGS study. Due to the change in HER-2 type, it was difficult to enroll in the trial and palliative trastuzumab + pertuzumab + docetaxel was administered as standard of care. After 3 cycles, the disease progressed at the first response evaluation, and subsequently, trastuzumab emtansin was changed to trastuzumab deruxetecan due to disease progression in 2 cycles. She had a partial response, but after 4 cycles she had a mixed response with some lesions re-increasing. Re-biopsy of the enlarged lesion was performed and it was again changed to HER-2 negative and hormone positive type (ER/PR/Her-2: 6/4/1+). The lapatinib + capecitabine regimen also showed progression at first response assessment after 2 cycles. She was switched to nab-paclitaxel for 3 cycles and achieved a partial response.

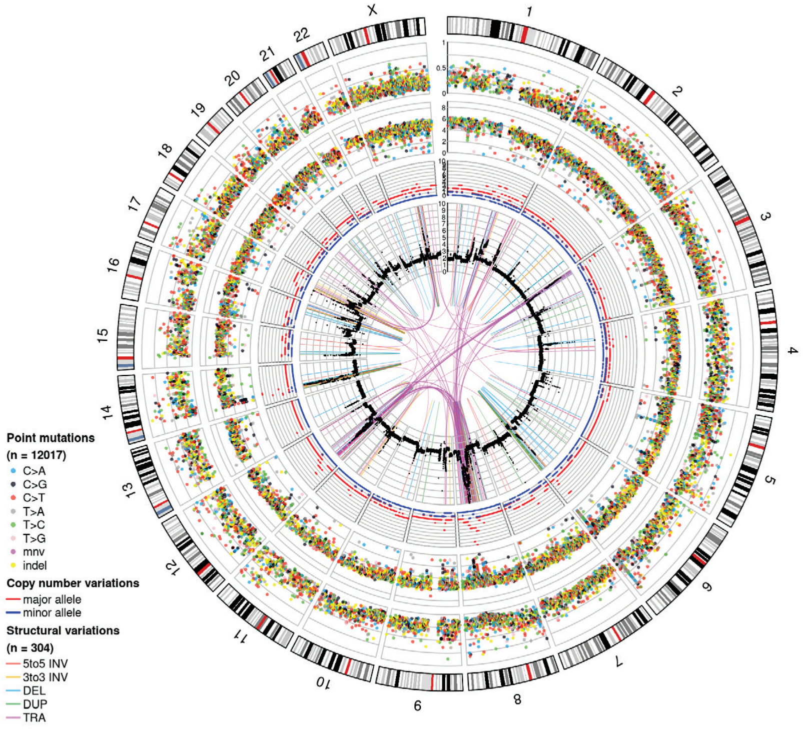

**Genome interpretation>**	Depth: Tissue: 34.5X, Normal: 1.97X	Total mutation counts: SNV: 10,059 Indel: 1,961 SV: 304
	Tumor fraction: 0.95, Mean ploidy: 2.4	Germline: not specific findings

ERBB2 p.L755S and focal amplification with a copy number of ~10 were identified. PIK3CA p.H1047R mutation was identified.

**Case 55**	M/62	**Non-smal cell lung cancer**	**Utility: Category I-1**
**Comments for Clinical Utility**	For ALK-rearranged NSCLC, brigatinib, alectinib, ceretinib and lorlatinib are approved **[Category I-1 FDA approved drug].**		

Clinical Presentation> A 62-year-old male, current smoker of 20 pack-years, presented with a 16 mm right lower lobe mass identified on chest CT during physical examination. A metastatic lymph node was identified in the right interlobar, subcarinal, upper paratracheal space and multiple perifissural and juxtapleural nodules along the right major and minor costal fissure and subpleural space. Histology was performed by video-assisted thoracic surgery on the pleural nodule and confirmed as adenocarcinoma, poorly differentiated, ALK (D5F3) CDx immunohistochemical staining positive. Barin MRI was performed and a single brain metastasis of 1.1 cm in the parietal lobe was identified. Clinical staging was confirmed as cT1bN2M1. Brain stereotactic radiosurgery was performed for ALK-positive metastatic non-small cell lung cancer, adenocarcinoma, and brigatinib was started. He had a partial response and has been treated for 13 months without progression of intracranial and extracranial disease.

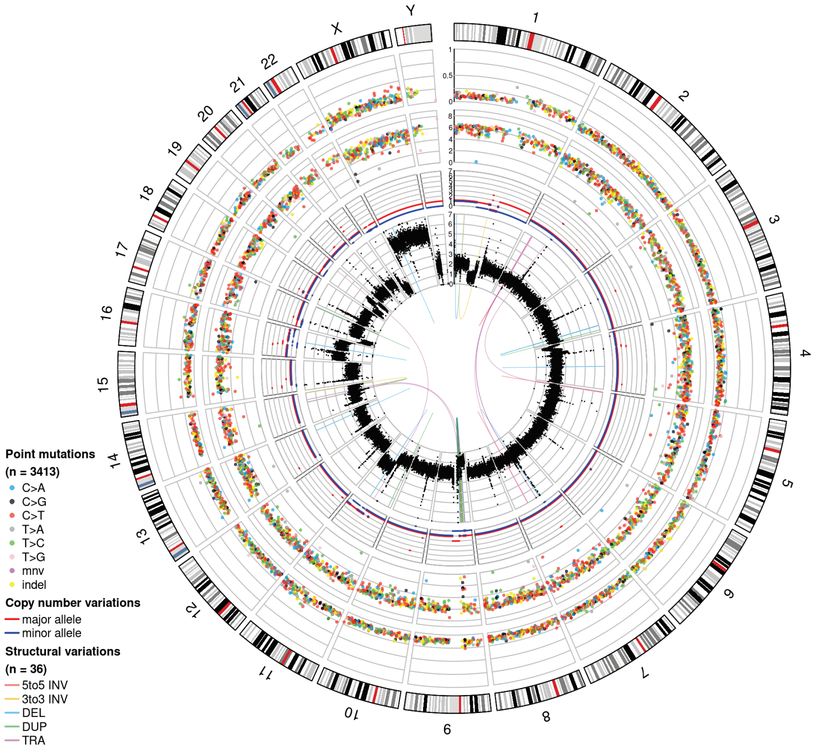

**Genome interpretation>**	Depth: Tissue: 68.1X, Normal: 25.3X	Total mutation counts: SNV: 2,974 Indel: 443 SV: 36
	Tumor fraction: 0.16, Mean ploidy: 1.7	Germline: not specific findings

A HIP1-ALK fusion was identified.

**Case 56**	M/58	**Stomach cancer**	**Utility: No (Category I-1)**
**Comments for Clinical Utility**	No actionable driver was reported.		

Clinical Presentation> A 58-year-old man was referred to a tertiary center for gastric cancer on upper GI endoscopy due to epigastric pain since 1 week. A diffuse large ulcerative lesion in the lower body was identified and the histology was reported as poorly cohesive carcinoma. The clinical staging was cT3N0M0 and distal Roux-en-Y gastrectomy was performed via laparoscopy. Pathology of the surgical specimen confirmed poorly cohesive carcinoma and was reported as pT4aN3b. Capecitabine + oxaliplatin was administered for 8 cycles as additional adjuvant anticancer treatment. He is being followed up recurrence free up to 12 months after surgery.

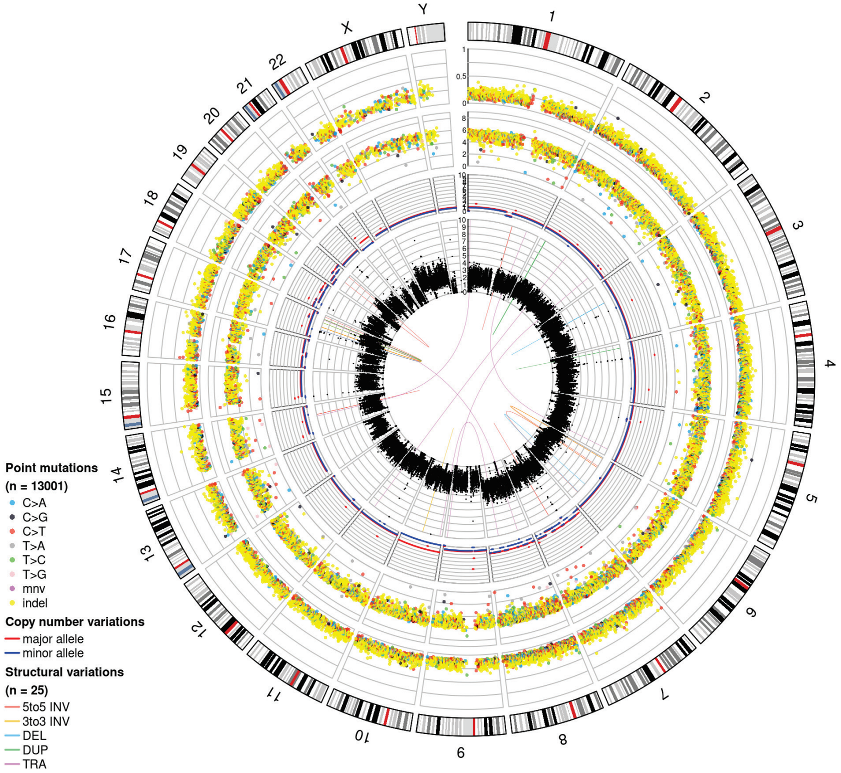

**Genome interpretation>**	Depth: Tissue: 49.9X, Normal: 18.9X	Total mutation counts: SNV: 5,037 Indel: 7,968 SV: 25
	Tumor fraction: 0.1, Mean ploidy: 1.5	Germline: not specific findings

RHOA p.G17E mutation that could be oncogenic driver or tumor suppressor gene in gastric cancer was identified.

<table>
<tr><td>Case 57</td><td>M/75</td><td>Urothelial carcinoma</td><td>Utility: Category I-1</td><td rowspan="3">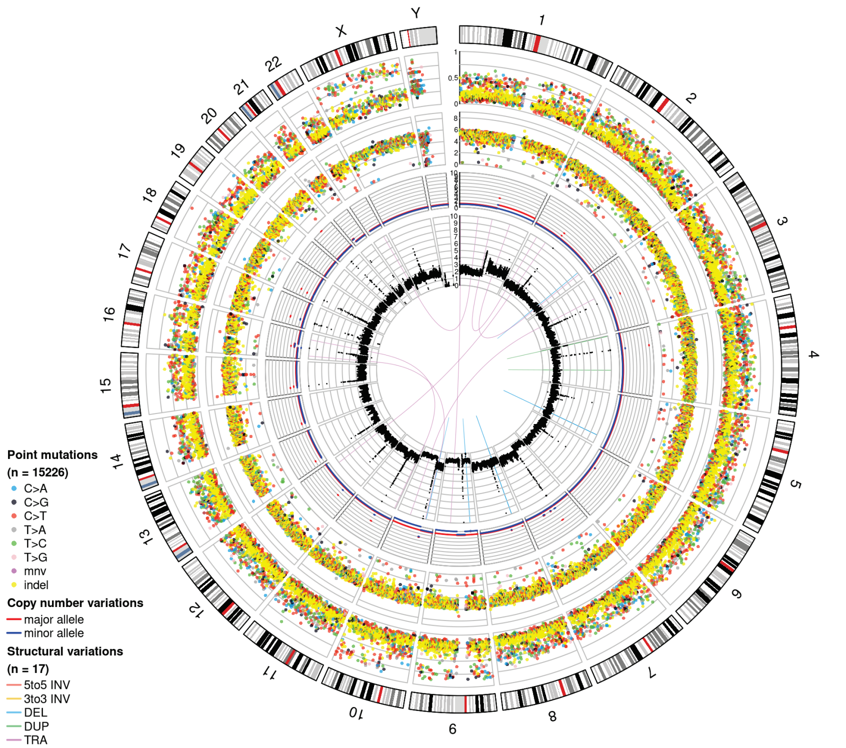
</td></tr>
<tr><td colspan="2">Comments for Clinical Utility</td><td colspan="2">Erdafitinib is approved for bladder cancer with FGFR3 mutation [Category I-1 FDA approved drug].</td></tr>
<tr><td colspan="4">Clinical Presentation> Transrectal ultrasound and cystoscopy performed on a 75-year-old man with voiding dysfunction and a 2-month history of gross hematuria revealed a large bladder mass. An abdominal CT confirmed the findings of bladder cancer and suspected prostate cancer. The clinical staging was cT4N0M0. Transurethral resection was performed, which revealed multiple papillary masses in the posterior and lateral walls of the bladder base, and the prostate mass was also resected by transurethral resection, confirming high-grade papillary urothelial carcinoma (pT1) with invasion from the bladder base area to the lamina propria, and adenocarcinoma of the prostate with Gleason score 3+4. He was followed up on an outpatient basis with urine cytology and abdominal CT, and cystoscopic examination was performed 1 year and 3 months later, and a papillary mass was identified in the bladder neck area, and a second transurethral resection was performed, and he was enrolled in a cancer whole genome study. Pathology confirmed high-grade noninvasive urothelial carcinoma. Six months later, a second transurethral resection was performed to remove the bladder-ureter-vesical junction mass, and pathology confirmed low-grade noninvasive papillary urothelial carcinoma.</td></tr>
<tr><td colspan="2" rowspan="2">Genome interpretation></td><td>Depth: Tissue: 41.3X, Normal: 22.5X</td><td colspan="2">Total mutation counts: SNV: 7,564 Indel: 7,664 SV: 17</td></tr>
<tr><td>Tumor fraction: 0.88, Mean ploidy: 2</td><td colspan="2">Germline: not specific findings</td></tr>
<tr><td colspan="5">PIK3CA p.E545K, FGFR3 p.S249C, TERT promoter mutation were identified.</td></tr>
</table>

Case 58	M/71	Colorectal cancer	Utility: Category II-2
**Comments for Clinical Utility**	This patient has an unusual clinical history: colorectal cancer treated 12 years ago has recurred with lung metastases, is poorly responsive to chemotherapy, and has a very aggressive biology with brain metastases. Given these unusual features, there was a clinical question as to whether it was metastatic colorectal cancer or another primary cancer. **[Category II-2 Tumor orgin]** In addition to carrying SNVs of APC and TP53, which are canonical drivers in common colorectal cancers, the whole genome shows overall duplication (mean ploidy 6) and copy number gain in various oncogenes. These patterns in the cancer genome are distinct from the average colorectal cancer and may be associated with aggressive cancer behavior or clinical course.		

Clinical Presentation> A 71-year-old man with a history of hypertension, diabetes mellitus, and 6 months of postoperative adjuvant chemotherapy for colon cancer 12 years ago presented with a mass of 8 cm in the right upper lung and was referred to the medical oncology department because histology confirmed metastatic adenocarcinoma from the colon (IHC result: TTF-1(-), NapsinA(-), CDX2(+), CK20(+), CK7(-)).
The best response of partial response was obtained with bevacizumab + mFOLFOX6 as palliative chemotherapy, but the response evaluation CT performed after 8 cycles confirmed progression of lung mass. Subsequently, afilbercept + FOLFIRI regimen was administered as second-line chemotherapy and tumor response evaluation of stable disease was obtained. After 6 cycles, tumor response evaluation showed progression of mediastinal lymph nodes and lung mass. The patient was financially unable to afford expensive chemotherapy and received capecitabine as third-line chemotherapy. After 2 cycles, he presented to the emergency department with right-sided motor weakness and brain MRI confirmed muptifocal bilateral brain metastases with peritumoral edema. He was treated with high-dose dexamethasone and whole-brain radiotherapy. 10.1 months after starting palliative chemotherapy, he was transferred to a hospice care center with best supportive care.

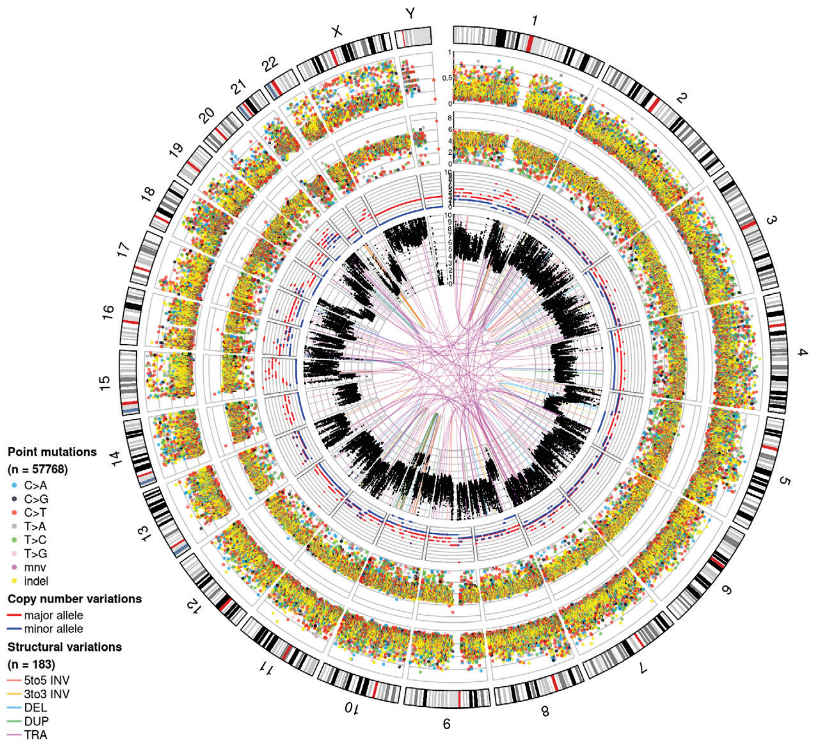

**Genome interpretation>**	Depth: Tissue: 24.3X, Normal: 21.2X	Total mutation counts: SNV: 39,095 Indel: 18,677 SV: 183
	Tumor fraction: 0.7, Mean ploidy: 6	Germline: not specific findings

Whole genome duplication with mean ploidy of 6 was noted.

Case 59	F/64	Breast cancer	Utility: Category II-1
**Comments for Clinical Utility**	Histologic examination of a hormone-positive metastatic breast cancer resistant to hormone therapy showed subtyping switching to HER-2+HR+, and a PIK3CA H1047R mutation was identified **[Category II-1 Drug resistance/responsive mechanisms]**.		

Clinical Presentation> A 64-year-old woman presented with a palpable, firm mass in the left breast of 5 months' duration. There were findings of inflammatory breast cancer in the left breast, lymph node metastasis, distant liver and sternum metastasis in the left axillary, supraclavicular and mediastinal spaces. The patient's father had a history of liver cancer and her mother had a history of colon cancer. Histology confirmed invasive ductal carcinoma and ER/PR/Her-2: 5+/0/- ki-67 65%. Palliative paclitaxel + carboplatin regimen was performed and a partial response was achieved, and letrozole regimen was maintained after 7 cycles. The patient had a mixed response, with masses in different locations decreasing and increasing in size, respectively. Re-biopsy was performed on the increasing site and subtyping was confirmed as ER/PR/Her-2: 3+/0/+ (IHC 2+ SISH positive). Treatment for HER-2+ HR+ metastatic breast cancer was trastuzumab + pertuzumab + docetaxel, and a primary refractory response was demonstrated as progression after 3 cycles and progression after 3 cycles of tastuzumab emtansine. Palliative adriamycin + cyclophosphamide was administered, and a partial response was shown and maintained for 5 cycles, but a progressive pattern was confirmed in some parts, and re-biopsy was confirmed as ER/PR/Her-2: 5+/0/+ (IHC 3+). After lapatinib + capecitabine therapy, she showed stable disease and is in the 5th cycle.

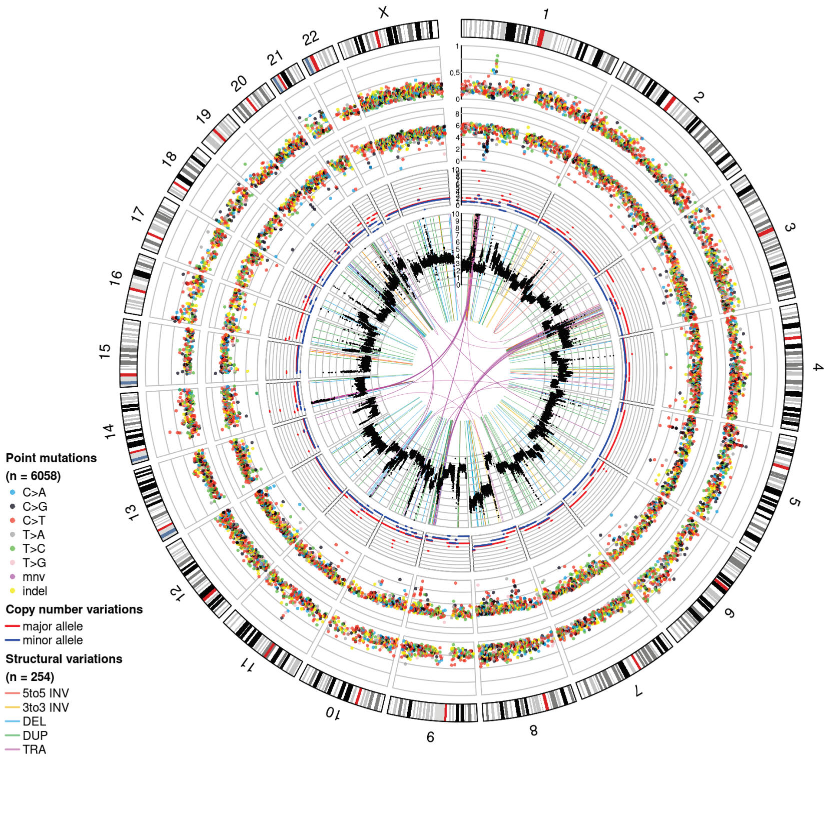

**Genome interpretation>**	Depth: Tissue: 37.9X, Normal: 19.7X	Total mutation counts: SNV: 5,248 Indel: 814 SV: 254
	Tumor fraction: 0.26, Mean ploidy: 2.6	Germline: not specific findings

PIK3CA p.H1047R mutation was identified. Mutation signature analysis revealed a high proportion of APOBEC-related mutational process.
Although BRCAness probability is below the threshold, the distribution of DUP/DEL resembles that of samples with BRCAness. It may indicate an early process of HRD-induced genomic scars, so close follow-up is required.

**Case 60**	F/58	**Head and Neck cancer**	**Utility: No (Category I-1)**
**Comments for Clinical Utility**	The tumor mutation burden of panel NGS performed in clinical practice was reported to be 14.8 mutations/megabase (Trusight Oncology 500). On WGS analysis, APOBEC mutational signatures were identified, the tumor mutation burden on WGS was relatively high at 24,588 mutations/whole genome. No actionable driver was reported.		

Clinical Presentation> A 58-year-old woman underwent wide excision surgery for maxillary sinus cancer, squamous cell carcinoma (pT4N0M0), postoperative 5-FU + cisplatin for 2 cycles and was referred for second opinion due to recurrence in left orbit, zygomatic bone, left masticatory space. Multidisciplinary tumor board discussed and planned revision maxillectomy, radical neck dissection, total parotidectomy, free flap reconstruction. A PD-L1 pharmDx 22C3 (IHC) CPS of 50 points was identified in the surgical tissue. Postoperative radiotherapy was done (60Gy/25Fx) and along with that pembrolizumab + 5-FU + cisplatin regimen was done. After 5 cycles of regimen she is on pembrolizumab maintenance only for 4 more cycles. best of response is partial response and she is progression free for 9 months after chemotherapy.

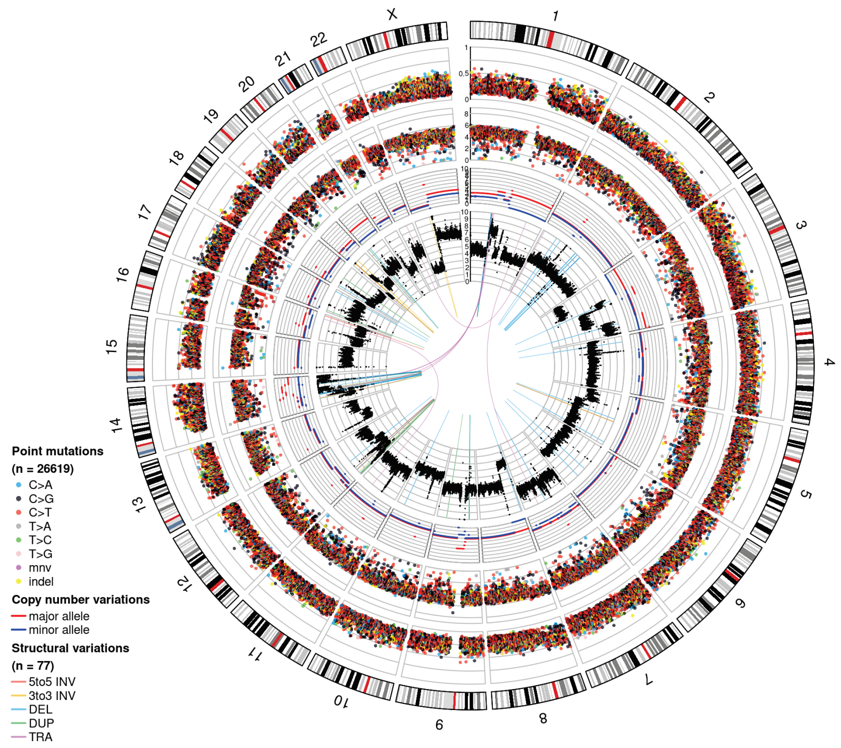

**Genome interpretation>**	Depth: Tissue: 38.1X, Normal: 23.8X	Total mutation counts: SNV: 24,588 Indel: 2,034 SV: 77
	Tumor fraction: 0.26, Mean ploidy: 5.1	Germline: not specific findings

There was a high proportion of APOBEC signature. Association with the virus (HPV or EBV) needs to be reviewed.
TERT promoter mutation and TGFBR2 stop gain mutation were identified

**Case 61**	M/63	**Urothelial carcinoma**	**Utility: Category I-1**
**Comments for Clinical Utility**	Erdafitinib is approved for bladder cancer with FGFR3 mutation **[Category I-1 FDA approved drug]**.		

Clinical Presentation> A 64-year-old man presented with painless gross hematuria of 3 days' duration and underwent cystoscopy, which revealed a 2-cm mass in the left posterior bladder wall. An abdominal CT confirmed the findings of bladder cancer. The clinical staging was cT1aN0M0. A transurethral resection was performed to remove the bladder mass. Pathology confirmed high-grade noninvasive urothelial carcinoma. Six months later, a second transurethral resection was performed and noninvasive papillary urothelial carcinoma, low grade, was confirmed in the right posterior wall and neck region of the bladder. A third transurethral resection was performed and noninvasive papillary urothelial carcinoma, low grade, was confirmed in the anterior bladder wall area.cancer whole genome study was enrolled. Pathology confirmed low-grade noninvasive urothelial carcinoma. Cystoscopy was performed at 6-month intervals, and the bladder mass was resected twice by transurethral resection, and all pathology results confirmed noninvasive urothelial carcinoma, low grade.

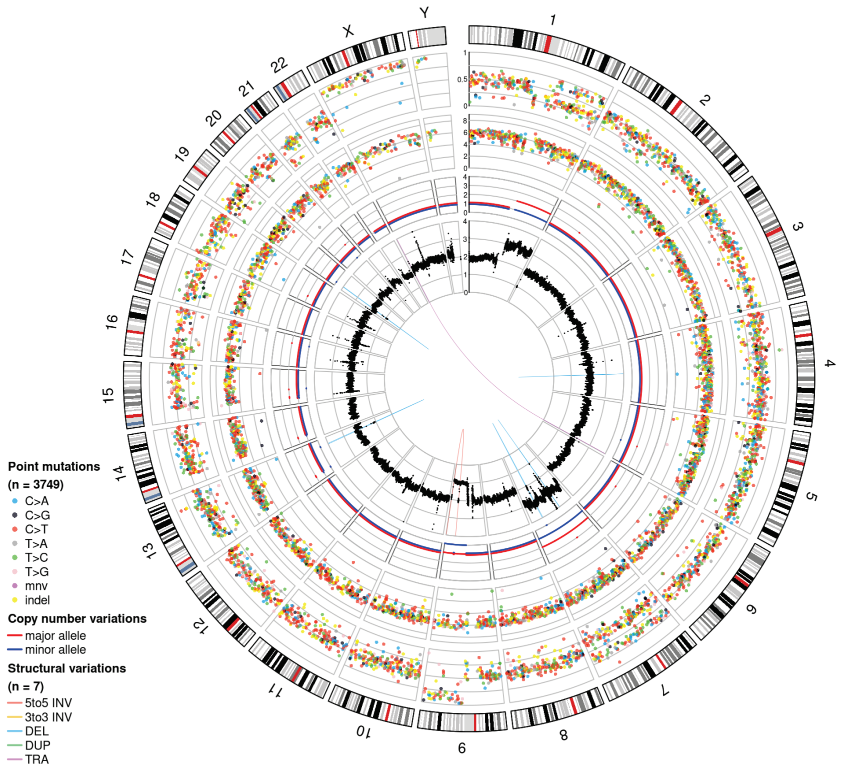

**Genome interpretation>**	Depth: Tissue: 48.6X, Normal: 19.8X	Total mutation counts:	SNV: 3,169 Indel: 584 SV: 7
	Tumor fraction: 0.96, Mean ploidy: 2	Germline:	not specific findings

FGFR3 p.Y373C mutation was identified.

**Case 2**	M/76	**Stomach cancer**	**Utility: No (Cagegory I-1)**
**Comments for Clinical Utility**	No actionable driver was reported.		

Clinical Presentation> A 76-year-old man with a history of bronchiectasis presented with a 4-5 cm ulcerofungating mass in the midbody, posterior wall on upper gastrointestinal endoscopy due to decreased appetite and weight loss of 8 kg in 5 months. The histological examination performed was reported as adenocarcinoma, poorly differentiated. Clinical staging was cT3N0M0 and distal Roux-en-Y gastrectomy was performed via laparoscopy. Pathology of the surgical specimen confirmed tubular adenocarcinoma, poorly differentiated, and was reported as pT1bN0. No further adjuvant therapy was administered and he is being followed up at 1 year postoperative f/u without recurrence.

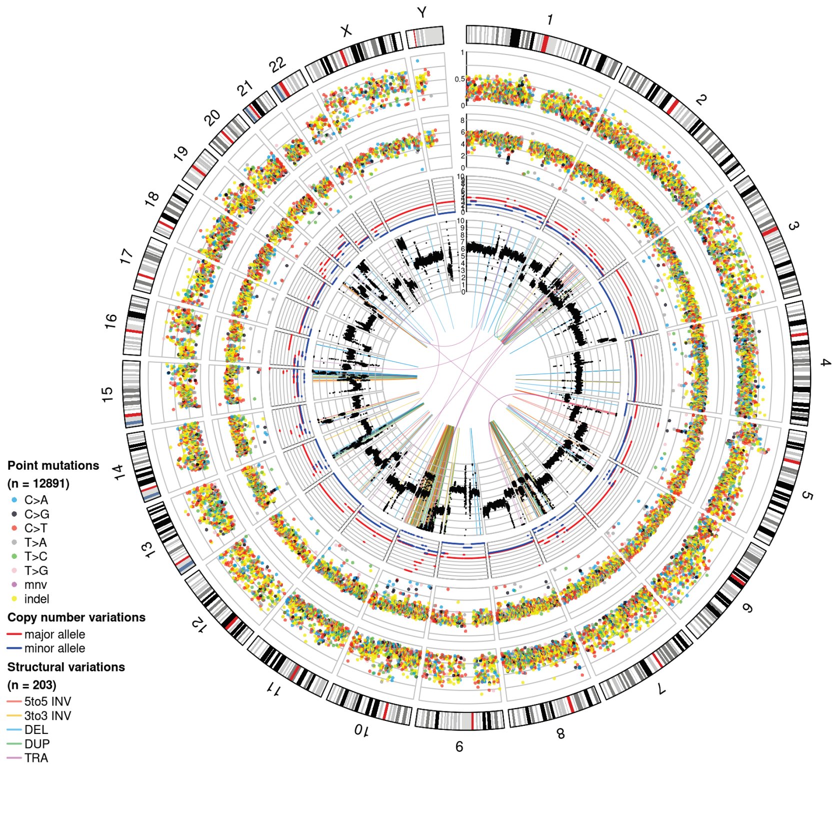

**Genome interpretation>**	Depth: Tissue: 39.3X, Normal: 24.0X	Total mutation counts: SNV: 8,856 Indel: 4,041 SV: 203
	Tumor fraction: 38, Mean ploidy: 5.2	Germline: not specific findings

Whole duplication with a mean ploidy of 5.2 was noted. We observed structural variations associated with copy number oscillation in chr2, chr7, chr10 and chr15, indicating chromothripsis.

**Case 63**	F/75	**Head and Neck cancer**	**Utility: Category I-2**
**Comments for Clinical Utility**	There are eligible trials for solid tumors with CCND1 amplification **[Category I-2 Clinical trial, NCT04439201, NCT04557449]**.		

Clinical Presentation> A 75-year-old woman who underwent partial glossectomy for tongue cancer, squamous cell carcinoma, 8 months ago presented with recurrence at the base of the tongue and axillary lymph nodes. The patient had received definitive RTx to both necks for Hodgkin lymphoma, stage IIA 5 years ago. removal of tongue tumor with modified radical both neck lymph node dissection, axillary lymph node dissection was performed for recurrent oral cavity cancer. Squamous cell carcinoma was confirmed in tongue and neck lymph node and only reactive hyperplasia was confirmed in axillary lymph node (rpT4aN3bM0). Postoperative concurrent chemoradiotherapy combined with 5-FU + cisplatin 2 cycle was performed. Further palliative therapy with pembrolizumab + 5-FU + cisplatin was considered, but the patient developed pneumonia due to COVID-19 infection and right femur fracture. Orthopedic surgery could not be performed due to poor general condition and bed-ridden status, and follow-up was lost, which could be followed up to 9 months after surgery.

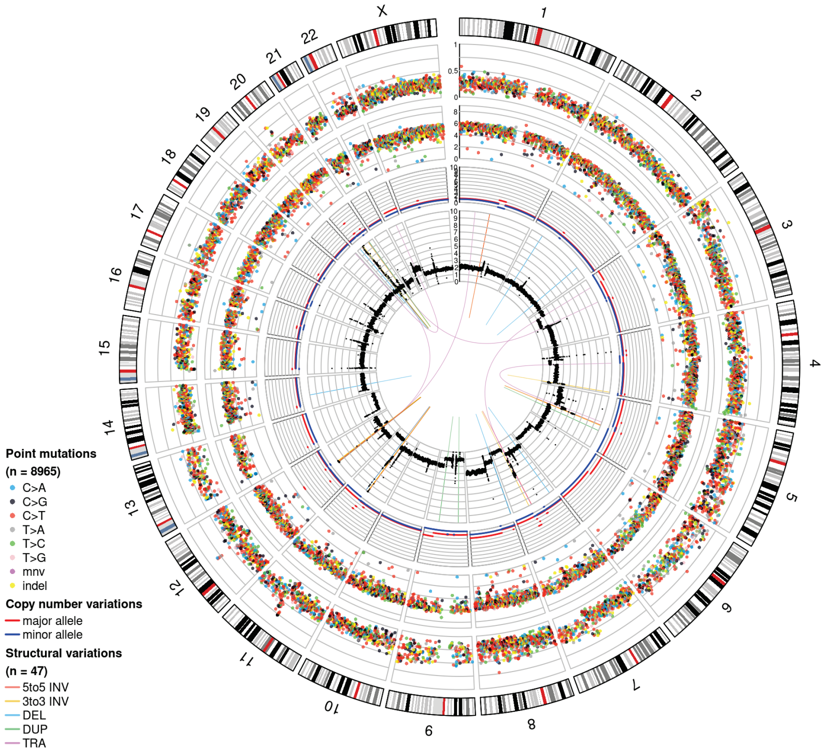

**Genome interpretation>**	Depth: Tissue: 38.5X, Normal: 20X	Total mutation counts: SNV: 7,839 Indel: 1,126 SV: 47
	Tumor fraction: 0.52, Mean ploidy: 2	Germline: not specific findings

CDKN2A deletion, double hit mutation in TP53 gene (p.T253A and p.S241P) and TERT promoter mutation were identified. We identified CCND1 focal amplification with a copy number of ~10 and MDM2 focal amplification with a copy number of ~34.
Some proportion of APOBEC-related mutational signature was noted.

Case 64	F/64	Urothelial carcinoma	Utility: No (Category I-1)
**Comments for Clinical Utility**	No actionable driver was reported.		

Clinical Presentation> A 64-year-old woman with an 11-year history of untreated painless gross hematuria presented with flank pain and dyuria. An abdominal CT was performed and showed a heterogeneous enhancing mass measuring more than 3 cm from the lower anterior portion of the left renal pelvis. Retrograde intrarenal surgery confirmed a large round mass in the left renal pelvis, and histologic examination was reported as fibroepithelial poly with squamous metaplasia. Histologic examination did not confirm malignancy, but considering the multiple clinical features, laparoscopic radical nephroureterectomy was performed under the impression of r/o upper ureteral cancer (clinical staging was cT4N1M0). Pathology confirmed infiltrating urothelial carcinoma, high grade with squamous and sarcomatous differentiation and tumor invasion into adjacent organs or through the kidney into the perinephric fat (pT4), and a metastatic mass in the perirenal fat was confirmed (pT4N2M0). Three weeks after surgery, the patient presented to the emergency room with severe back pain, and a CT and spinal MRI scan revealed multiple bone metastases at C5, T3-4, T7-8, T11-L5, sacrum, right posterior ilium, 4th, 5th and 7th ribs, multiple liver metastases, and exacerbated multiple conglomerate necrotic LNs along the left para-aortic chain. Palliative gemcitabine carboplatin 3 cycles were administered for rapidly progressing tumor, but progression was noted at first tumor response evaluation and subsequent pembrolizumab and paclitaxel regimens showed primary resistance. The patient died 7 months after diagnosis.

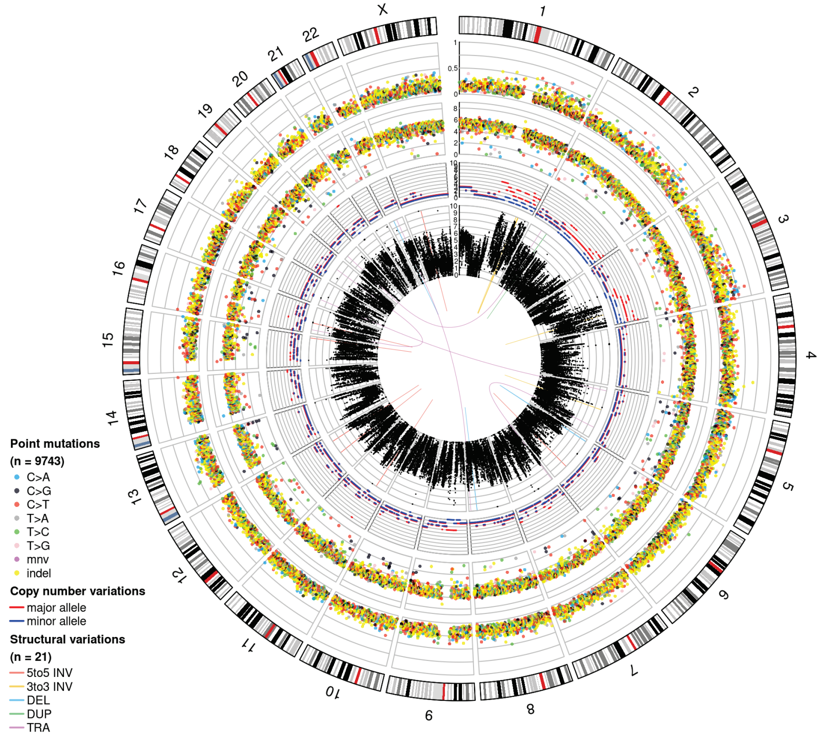

**Genome interpretation>**	Depth: Tissue: 48.5X, Normal: 18.4X	Total mutation counts: SNV: 6,063 Indel: 3,681 SV: 21
	Tumor fraction: 28, Mean ploidy: 3.1	Germline: not specific findings

NFE2L2 p.G81D, PIK3CA p.H1047R oncogenic mutations and TERT promoter mutation were identified.

Case 65	F/71	Urothelial carcinoma	Utility: Category I-2
**Comments for Clinical Utility**	There are eligible trials for solid tumors with HRAS mutations **[Category I-2 Clinical trial, NCT01374789, NCT04284774].**		

Clinical Presentation> An abdominal CT performed on a 71-year-old woman who presented with a painless gross hematuria of 1 month's duration revealed a large bladder neck mass. A 3 cm mass was excised from the posterior bladder neck via transurethral resection under r/o bladder cancer impression (clinical staging was cT2N0M0). pathology confirmed high grade poorly differentiated carcinoma. radical cystectomy with intracorporeal urinary diversion was performed. The pathology of the surgical specimen was confirmed as high grade infiltrating urothelial carcinoma, pT3. The patient received 3 cycles of gemcitabine + cisplatin as adjuvant chemotherapy, followed by 3 cycles of palliative pembrolizumab as metastases were identified in multiple lung and left neck lymph nodes. The patient showed a partial response and a reduction in the size of the metastases in the neck node, but as an immune-related adverse event, he developed Gr3 uveitis and was treated with high dose corticosteroids. After 3 months of pembrolizumab discontinuation, progression was noted and palliative paclitaxel was administered and a partial response was achieved. After a total of 6 cycles, he is currently on active surveillance.

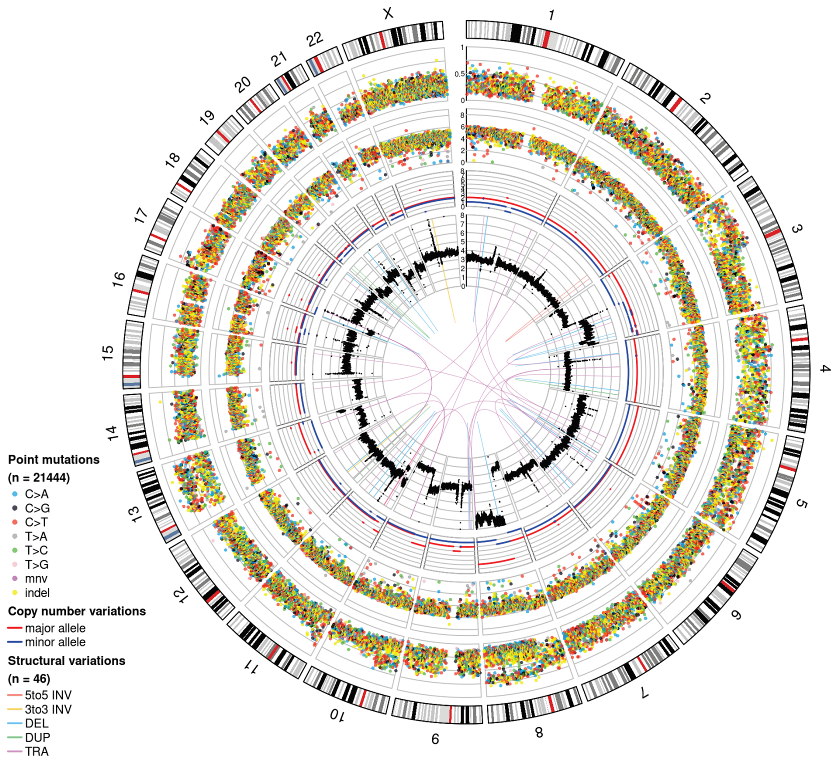

**Genome interpretation>**	Depth: Tissue: 38.8X, Normal: 16.9X	Total mutation counts: SNV: 15,504 Indel: 5,943 SV: 46
	Tumor fraction: 0.55, Mean ploidy: 3.4	Germline: not specific findings

HRAS p.G13D mutation and TP53 p.A69Lfs* mutation were identified.

Case 66	F/53	**Breast cancer**	**Utility: Category I-1**
**Comments for Clinical Utility**	Alpelisib is approved for hormone-sensitive metastatic breast cancer with a PIK3CA mutation [Category I-1 FDA approved drug]. In this patient's case, cancer WGS played a key role in the differential diagnosis of a double primary cancer, which helped to clarify the origin of the cancer, change the patient's diagnosis and modify the treatment strategy **[Category II-2 Tumor orgin].**		

Clinical Presentation> A 53-year-old woman diagnosed with metastatic gastric cancer with peritoneal and bone metastases and receiving palliative Capecitabine + Oxaliplatin (CapeOx) presented with a palpable mass in her left breast. She had received 9 cycles of CapeOx with a partial response. At 9 hours, a 1.0 cm irregularly shaped mass was noted in the left breast. Histology confirmed invasive lobular carcinoma and reported ER/PR/Her-2 7+/4+/0, ki-67 5%. The clinical staging was cT1N0M0. The patient's mother had a history of gastric and breast cancer in her 60s. As the treatment of metastatic gastric cancer is more important for the patient's survival, and capecitabine may have anticancer effect on breast cancer, the treatment of breast cancer was decided to be active surveillance. Five months later, the gastric cancer was in metabolic complete remission with no SUV uptake on PET-CT, and the breast mass increased in size to 1.8 cm, so we decided to surgically resect the breast cancer. A partial mastectomy was performed, and pathological examination of the surgical tissue confirmed the histological type as invasive lobular carcinoma, and the size of the largest invasive cancer was 30x28 mm and the extent was 50x45 mm. The resection margin was confirmed as positive and reported as ypT2N2M0. After surgery, a multidisciplinary tumor board was performed, and radiation therapy was omitted because she could not take a long break from systemic therapy for gastric cancer, and it was decided to start letrozole therapy as adjuvant therapy for hormone-positive breast cancer and resume palliative chemotherapy for gastric cancer. In addition, given the patient's family history, it was suggested that she be evaluated for hereditary diffuse gastric cancer. Cancer WGS was performed and WGS was performed for gastric cancer and breast cancer, respectively. On the molecular tumor board, it was concluded that the stomach and breast shared a high number of mutational profiles and that a pathology review of the primary cancer was necessary. As no differential diagnosis for breast cancer was performed at the time of the initial diagnosis of gastric cancer, additional immunohistochemical staining was performed and GATA3, BRST-2, and ER were all reported as positive in the gastric surgical specimen. Ultimately, the diagnosis was revised and it was concluded that the gastric cancer was not the primary cancer, but rather an invasive lobular breast carcinoma with gastric and peritoneal metastases. CapeOx therapy was discontinued and letrozole administered with adjuvant intent was changed to palliative intent. She remains on letrozole for one year after breast cancer surgery without progression.

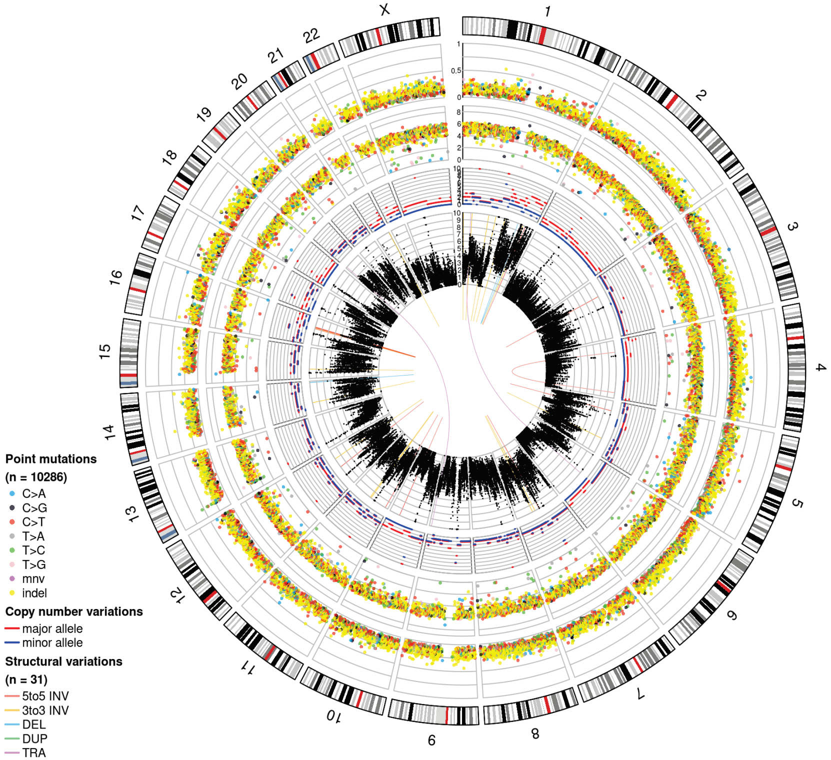

**Genome interpretation>**	Depth: Tissue: 63.1X, Normal: 22.1X	Total mutation counts: SNV: 3,820 Indel: 5,028 SV: 55
	Tumor fraction: 0.2, Mean ploidy: 2.6	Germline: not specific findings

CDH1 p.V55L mutation with loss of heterozygosity and PIK3CA p.N345K mutation were identified.

**Case 67**	M/74	**Colorectal cancer**	**Utility: Category I-2**
**Comments for Clinical Utility**	PIK3CA mutations have an indication for alpelisib in hormone positive metastatic breast cancer and are being investigated in clinical trials for efficacy in gastrointestinal cancers such as colorectal cancer. **[Category I-2 Screening for mutation-specific clinical trials NCT02264678]**		

Clinical Presentation> A 74-year-old man with Parkinson's disease and moderate cognitive dysfunction was referred to a tertiary center because of a positive fecal occult blood test at routine health screening and a rectal cancer finding at colonoscopy.
A 3.2 cm ulcerofungating mass was identified in the rectum, 9 cm from the anal verge. Histology reported adenocarcinoma, moderately differentiated, with clinical staging of cT3N0, circumferential resection margin negative. Preoperative concurrent chemoradiotherapy was planned and radiotherapy of 5000 cGy with capecitabine was performed. Preoperative response assessment showed a moderate response with decreased mass size on MRI, corresponding to MR tumor regression grade 3, and PET-CT showed a complete metabolic response in the main rectal mass. However, a small non-calcific nodule of 9 mm was identified in the right lower lung on chest CT, and the possibility of metastasis could not be excluded. After being informed of the possibility of metastasis and postoperative recurrence, the patient and her guardian refused surgery and are currently undergoing regular follow-up.

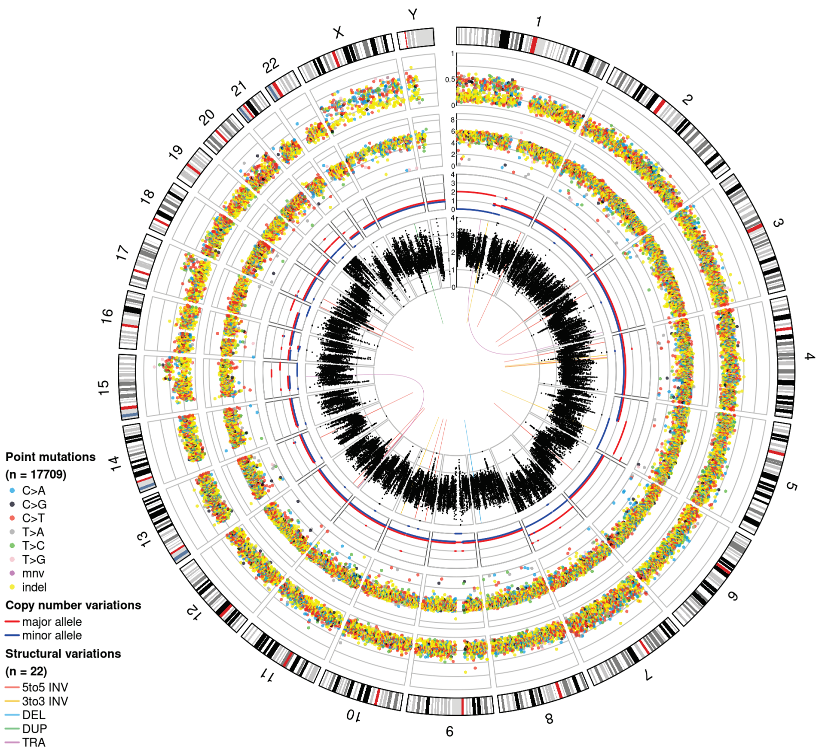

**Genome interpretation>**	Depth: Tissue: 49.8X, Normal: 21.2X	Total mutation counts: SNV: 10,879 Indel: 6,831 SV: 23
	Tumor fraction: 48, Mean ploidy: 2	Germline: not specific findings

PIK3CA p.M1043I mutation was identified.

Case 68	M/63	Urothelial carcinoma	Utility: Category I-1
**Comments for Clinical Utility**		There are eligible trials for solid tumors with CCND1 amplification **[Category I-2 Clinical trial, NCT04439201, NCT04557449]**.	

Clinical Presentation> A 63-year-old man presented with a 2-month history of painless gross hematuria and underwent abdominal CT, which revealed a bladder neck mass. A 1.5 cm mass was removed from the left lateral wall of the bladder by transurethral resection under r/o bladder cancer impression (clinical staging was cT1N0M0), and pathology confirmed high-grade papillary urothelial carcinoma invading the lamina propria. During a tansurethral resection performed 6 months later for non-muscle invasive bladder cancer, 2 masses were removed from the anterior bladder wall, both of which were confirmed to be high-grade papillary urothelial carcinoma invading the lamina propria, and he participated in the cancer WGS study at this point. He is being followed up with urine cytology, abdominal CT.

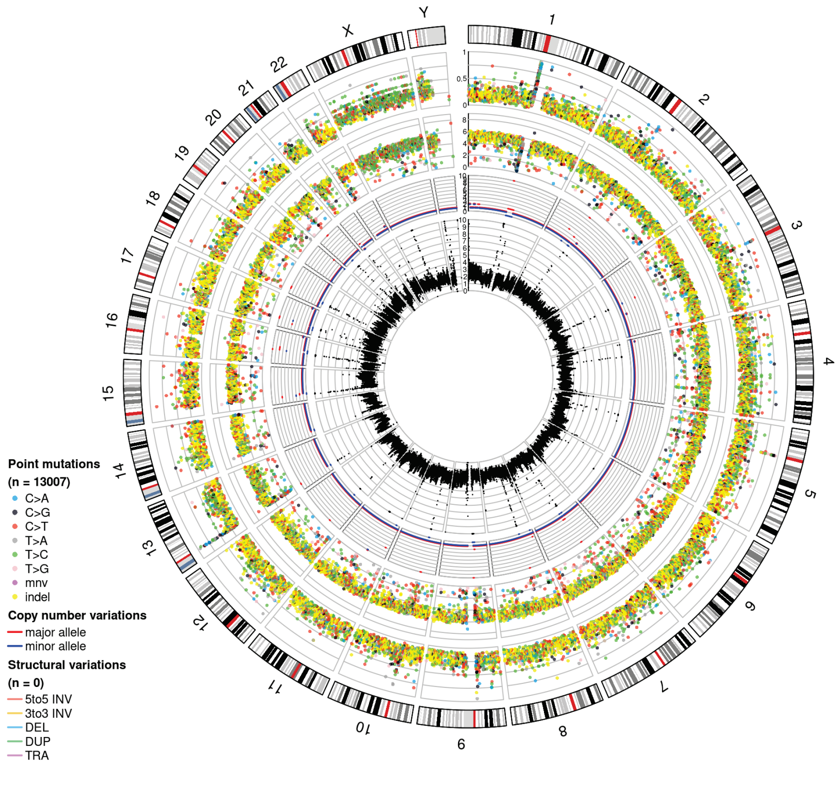

**Genome interpretation>**	Depth: Tissue: 50.2X, Normal: 27.2X	Total mutation counts: SNV: 33,467 Indel: 8,611 SV: 37
	Tumor fraction: 0.38, Mean ploidy: 2.9	Germline: not specific findings

CCND1 focal amplification with a very high copy number of ~131 was identified.

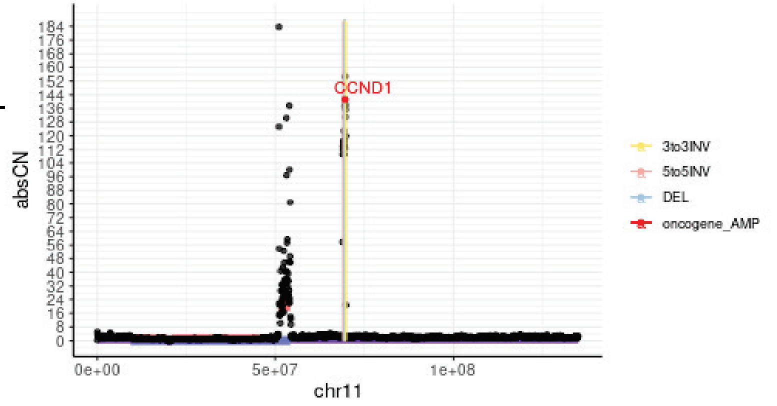

**Case 69**	M/69	**Stomach cancer**	**Utility: Category I-2**
**Comments for Clinical Utility**	A Clinical Trial on MET Amplification in Gastric Cancer is Available for Participation. **[Category I-2 Clinical trial, NCT05620628]**		

Clinical Presentation> A 69-year-old man with no significant medical history presented to the emergency department with dyspnea on exertion and a weight loss of 20 kg in 6 months. Upper gastrointestinal endoscopy performed revealed an ulcer-infiltrative mass measuring 12 cm in size in the distal antrum. The histologic examination performed was reported as adenocarcinoma, poorly differentiated. Retroperitoneal lymph node metastases were identified, and palliative capecitabine + oxaliplatin therapy was instituted. He died due to pneumonia that developed after 2 cycles.

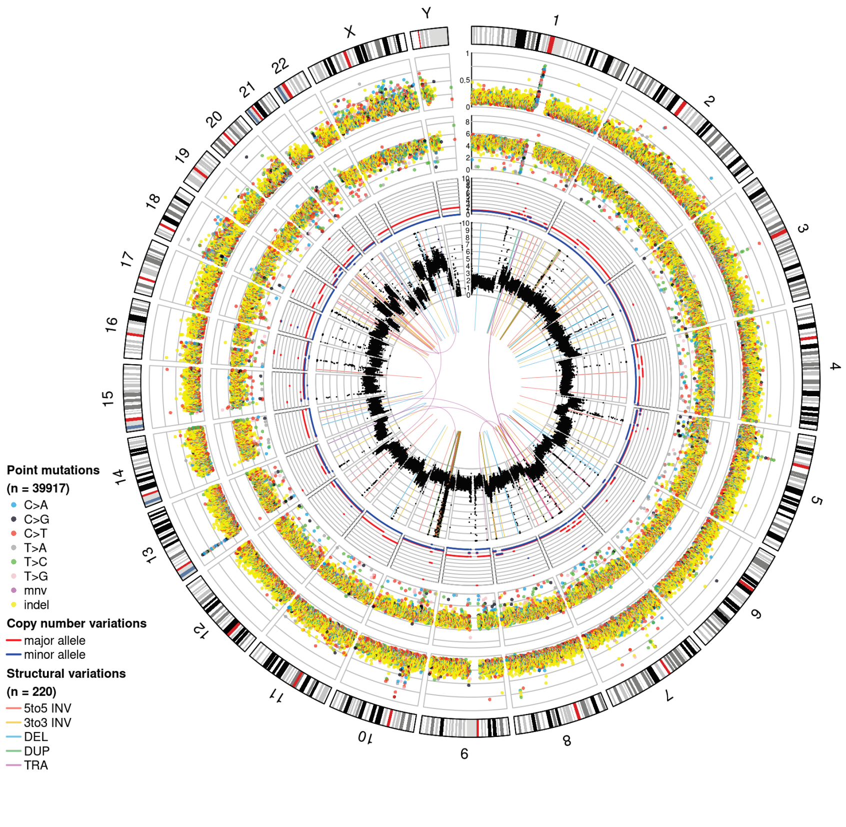

**Genome interpretation>**	Depth: Tissue: 44.9X, Normal: 19.9X	Total mutation counts: SNV: 21,459 Indel: 18,460 SV: 220
	Tumor fraction: 0.3, Mean ploidy: 2.4	Germline: not specific findings

Focal amplification involving EGFR (copy number ~73), CDK6 (copy number ~37), MET (copy number ~28) and MYC (copy number ~34) oncogene were identified.

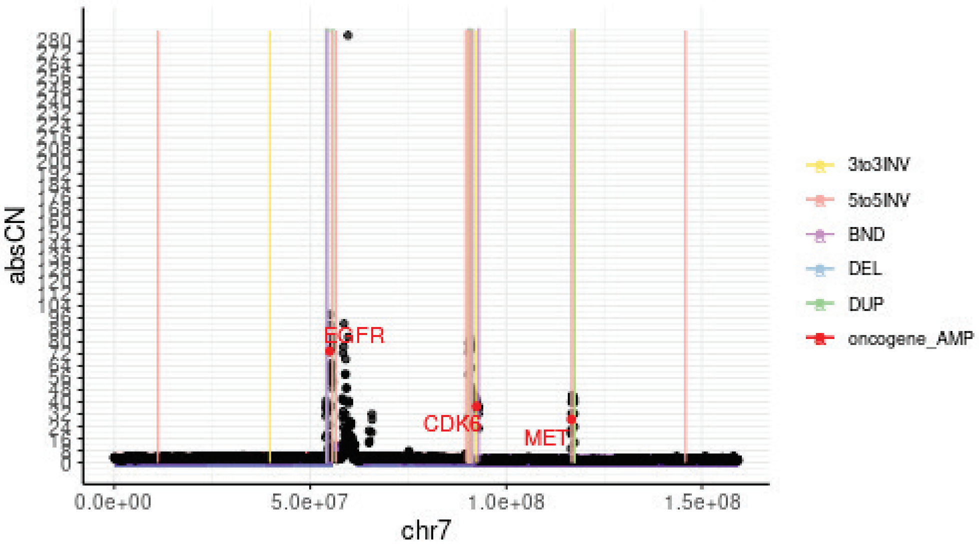
absCN
EGFR
CDK6
MET
0.0e+00
5.0e+07
1.0e+08
1.5e+08
chr7
3to3INV
5to5INV
BND
DEL
DUP
oncogene_AMP

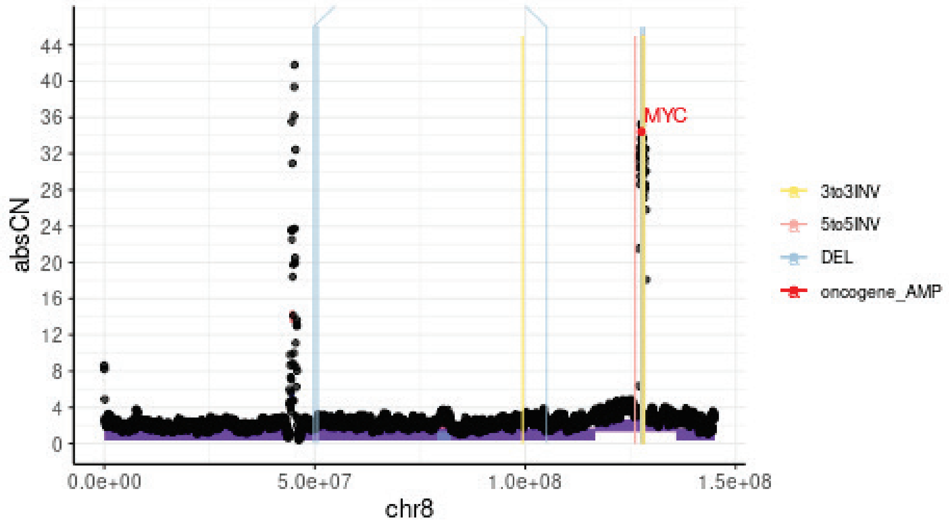
absCN
44
40
36
32
28
24
20
16
12
8
4
0
MYC
0.0e+00
5.0e+07
1.0e+08
1.5e+08
chr8
3to3INV
5to5INV
DEL
oncogene_AMP

**Case 70**	M/64	**Stomach cancer**	**Utility: Category I-2**
**Comments for Clinical Utility**	A Clinical Trial on CCND1 Amplification in Gastric Cancer is Available for Participation. **[Category I-2 Clinical trial, NCT02523014]**		

Clinical Presentation> A 64-year-old man with no unusual medical history presented with heartburn and a 3-month weight loss of 8 kg. Upper gastrointestinal endoscopy revealed an ulcerated infiltrative mass measuring 12 cm in the proximal antrum. Histology was reported as mucinous adenocarcinoma. Clinical staging was reported as cT4N1 and total gastrectomy was planned, but operative findings confirmed peritoneal seeding and surgery was not performed. After cycle #9, peritoneal seeding progression was confirmed, so regimen change to pacliataxel + ramucirumab was performed, and after 3 cycles, ascites decreased, stable disease response was achieved, and ongoing treatment is ongoing. palliative nivolumab + mFOLFOX6 was performed, and stable disease was confirmed with best response.

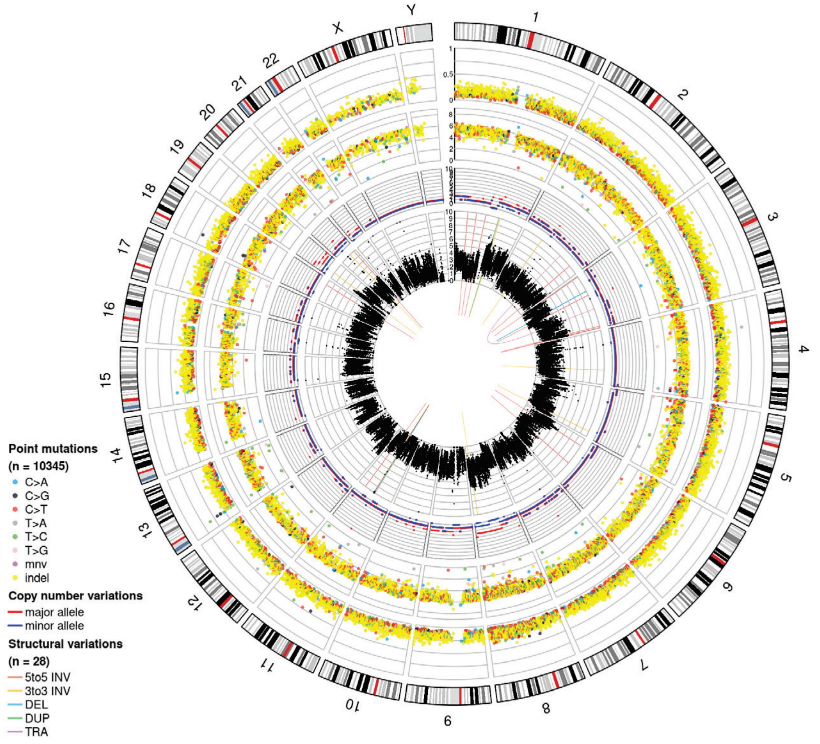

**Genome interpretation>**	Depth: Tissue: 52.4X, Normal: 21.6X	Total mutation counts: SNV: 3,694 Indel: 6,651 SV: 28
	Tumor fraction: 0.2, Mean ploidy: 2	Germline: not specific findings

CCND1 focal amplification was identified.

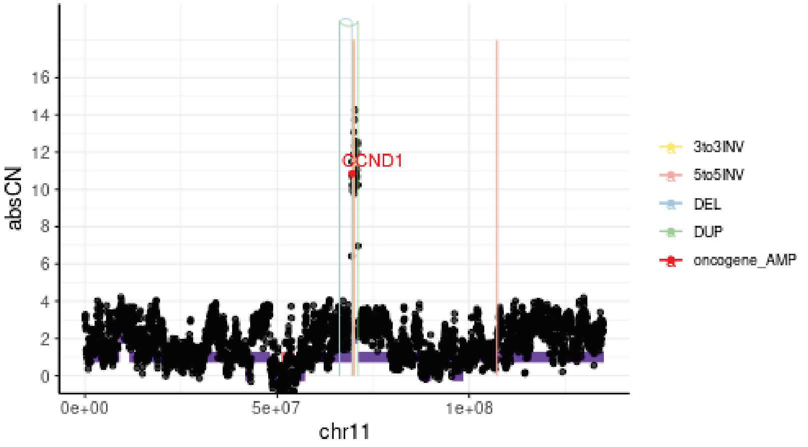

**Case 71**	F/59	**Neuroendocrine tumor**	**Utility: No (Category I-1)**
**Comments for Clinical Utility**	No actionable driver was reported.		

Clinical Presentation> A patient who underwent pylorus-preserving pancreaticoduodenectomy for grade 2 pancreatic NET 8 years ago presented with facial, hand, and foot edema for 1 month, and abdominal CT showed an enhancing mass of 9.7 cm in the central liver. Liver core needle biopsy was performed and a grade 2 neuroendocrine tumor was confirmed. Subsequently, lanreotide LAR was administered and the patient progressed 7 months later and remains on everlorimus therapy.

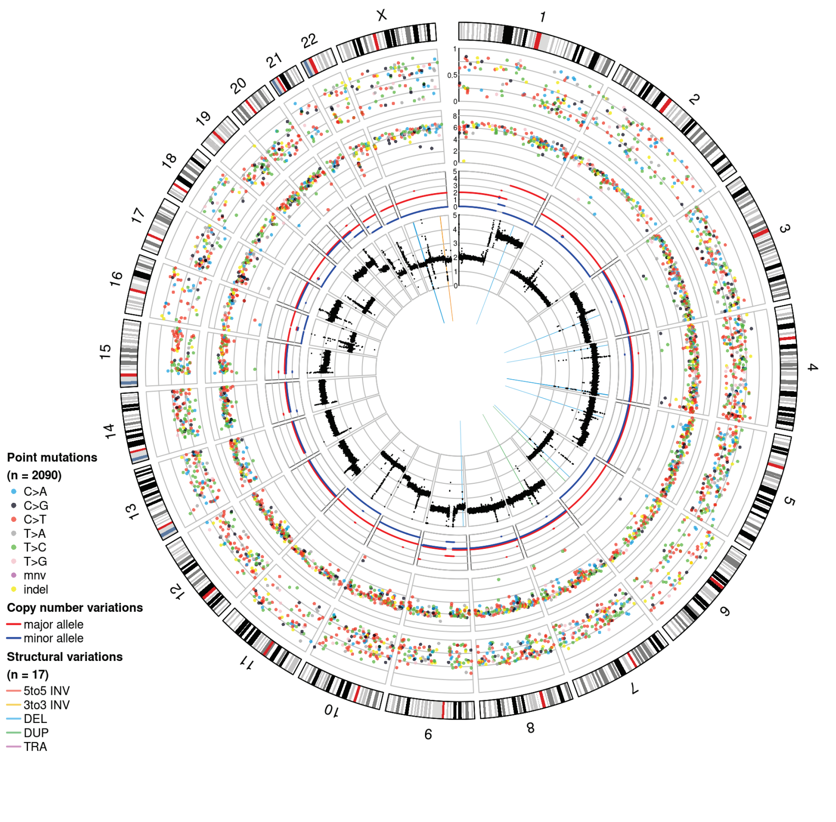

**Genome interpretation>**	Depth: Tissue: 39.6X, Normal: 23.4X	Total mutation counts: SNV: 1,917 Indel: 173 SV: 17
	Tumor fraction: 0.74, Mean ploidy: 3.1	Germline: not specific findings

MEN1 frameshift mutation (p.H139Pfs*) was identified.

Case 72	F/62	Stomach cancer	Utility: Category I-2
**Comments for Clinical Utility**	The BRAF V600E mutation in gastric cancer has no approved drugs and there are no clinical trials available. **[Category I-2 Clinical trial, DRKS00015849)]**		

Clinical Presentation> A 62-year-old woman with no significant past medical history presented with an 8 cm ulcerated infiltrative mass in the lower body to the cardia on upper GI endoscopy performed for epigastric pain. The histological examination performed was reported as poorly cohesive carcinoma. It was reported as cT4N2 at clinical staging and total gastrectomy with Roux-en Y was performed. Pathologic examination of surgical tissue was reported as poorly cohesive carcinoma, pT4aN3b. Adjuvant capecitabine + oxaliplatin 8 cycle was performed, and he is followed up without any recurrence in the follow-up examination up to 1 year after surgery.

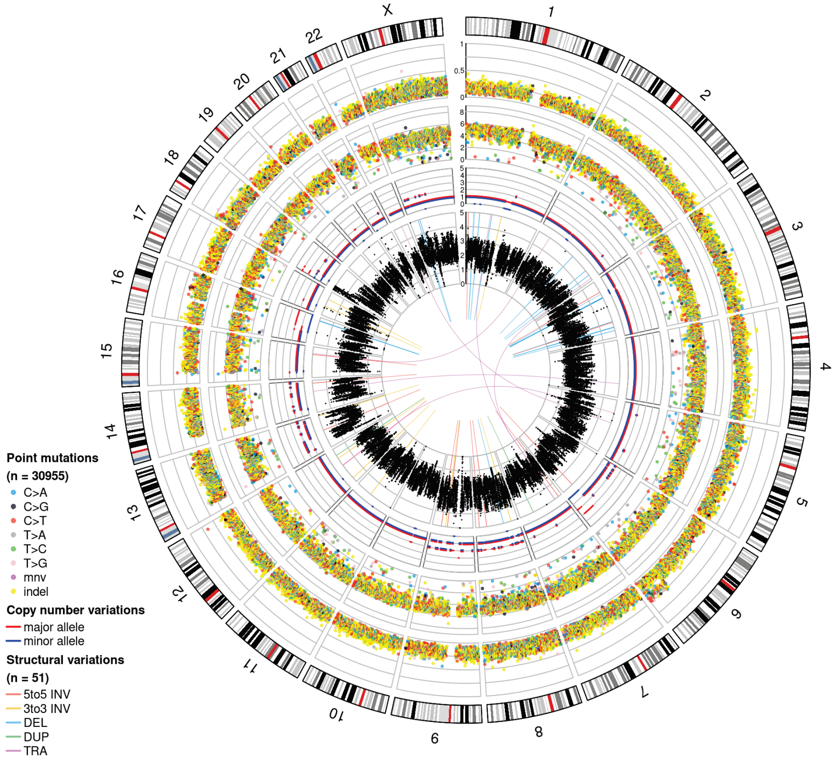

Genome interpretation>	Depth: Tissue: 77.3X, Normal: 20.7X	Total mutation counts: SNV: 19,193 Indel: 1,1764 SV: 52
	Tumor fraction: 0.4, Mean ploidy: 2	Germline: not specific findings

TGFBR2 p.D446N mutation and BRAF p.V600E mutation were identified.

Case 73	M/83	Urothelial carcinoma	Utility: No (Category I-1)
**Comments for Clinical Utility**	No actionable driver was reported.		

Clinical Presentation> An 83-year-old man presented with painless gross hematuria for 1 month, an abdominal CT was performed and a bladder neck mass was identified. Cystoscopy revealed a multiple bladder mass extending over the entire bladder. Random biopsies were taken from the anterior, posterior and both lateral sides of the bladder by transurethral resection under r/o bladder cancer impression (clinical staging was cT1N0M0) and pathology confirmed high grade non-muscle invasive urothelial carcinoma. He is being followed up after 6 months with no abnormal findings on outpatient urine cytology.

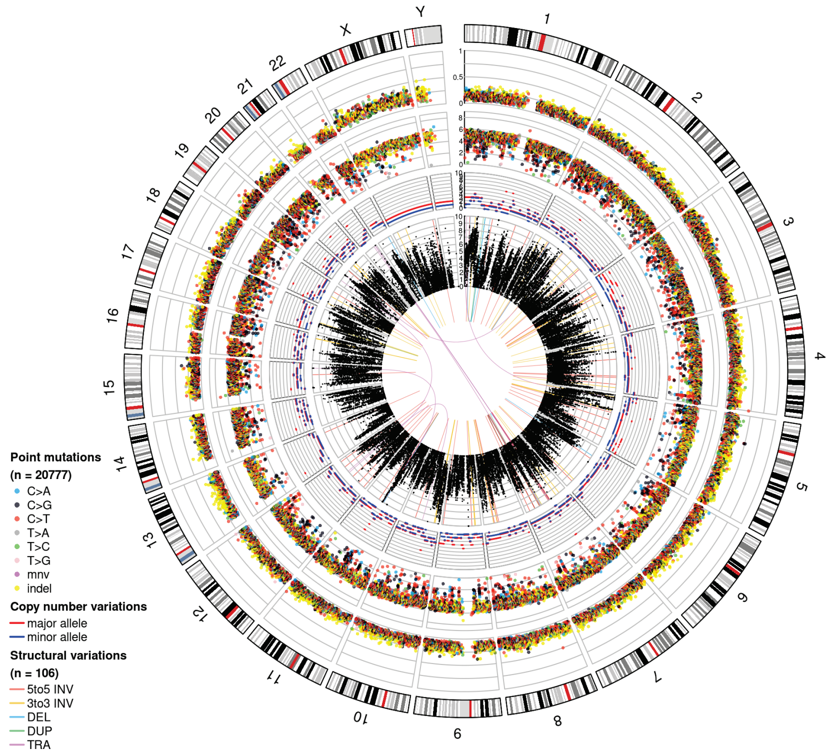

Genome interpretation>	Depth: Tissue: 60.7X, Normal: 29.9X	Total mutation counts: SNV: 17,150 Indel: 3629 SV: 106
	Tumor fraction: 0.13, Mean ploidy: 2.5	Germline: not specific findings

Mutational signature analysis revealed a high proportion of APOBEC-related mutational process.

**Case 74**	F/70	**Stomach cancer**	**Utility: Category I-1**
**Comments for Clinical Utility**	Pembrolizumab is indicated for the metastatic solid cancer with microsatellite high (MSI-H), including gastric cancer **[Category I-1 FDA approved drug]**		

Clinical Presentation> A 70-year-old woman who underwent left upper lobectomy for non-small cell lung cancer, adenocarcinoma, pT2bN0M0, 3 years ago presented to the emergency department for melena. An upper gastrointestinal endoscopy was performed and revealed a 2 cm ulcerated fungating mass in the gastric cardia, presumed to be the focus of bleeding. Histology revealed adenocarcinoma, moderately differentiated. Clinical staging was cT1N0M0 and distal Billroth II Braun gastrectomy was performed via laparoscopy. Pathology of the surgical specimen confirmed tubular adenocarcinoma, poorly differentiated, and was reported as pT3N0. No additional adjuvant chemotherapy was administered. He is being followed up for recurrence free up to 12 months after surgery.

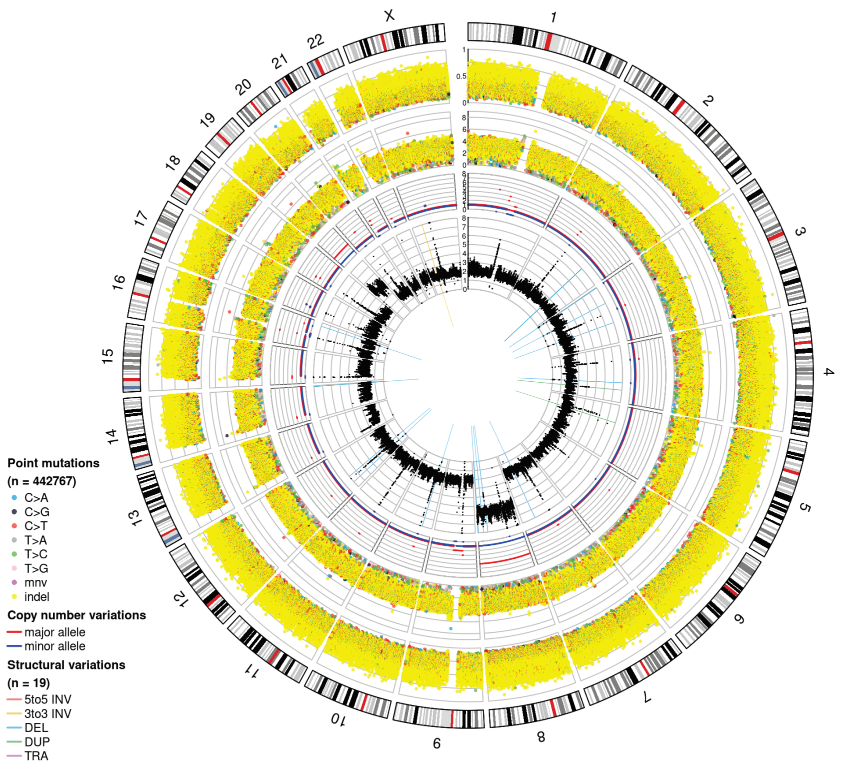

**Genome interpretation>**	Depth: Tissue: 42.7X, Normal: 21.4X	Total mutation counts: SNV: 82,184 Indel: 36,087 SV: 19
	Tumor fraction: 0.26, Mean ploidy: 2.1	Germline: not specific findings

This sample has deficient DNA mismatch repair pathway evidenced by high proportion of SBS44, high number of indel and MSI-score of 22.0 (cutoff 2.0). PIK3CA p.E545G mutation was identified.

Case 75	F/64	Stomach cancer	Utility: No (Category I-1)
**Comments for Clinical Utility**	No actionable alteration was identified.		

Clinical Presentation> A 64-year-old woman with no significant past medical history underwent upper gastrointestinal endoscopy for a 1-month history of heartburn, which revealed an ulcerated infiltrative mass measuring more than 6 cm from mid-antrum to midbody. The histologic examination performed was reported as poorly differentiated adenocarcinoma. Clinical staging was reported as cT4bN2 and total gastrectomy with Roux-en Y was performed. Pathology of surgical tissue was reported as adenosquamous carcinoma, pT4bN3b. She completed 8 cycles of capecitabine + oxaliplatin as adjuvant chemotherapy. She is followed up and remains recurrence free until 1 year post surgery.

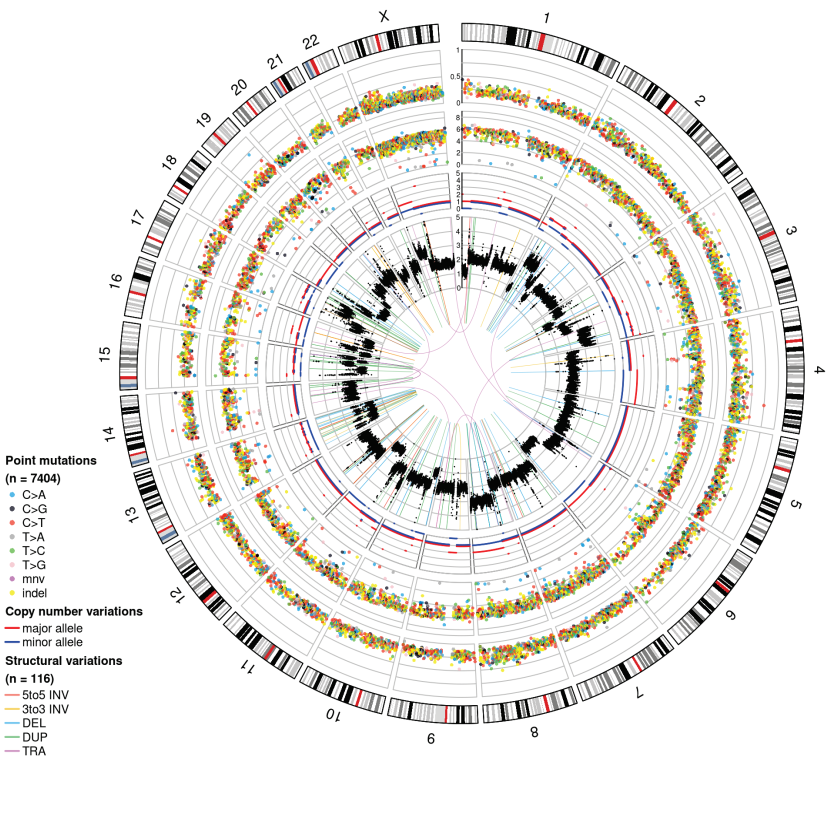

**Genome interpretation>**	Depth: Tissue: 36.8X, Normal: 18.6X	Total mutation counts: SNV: 5,650 Indel: 1,759 SV: 116
	Tumor fraction: 0.33, Mean ploidy: 1.8	Germline: not specific findings

Widespread duplications over whole genome was noted. CDH1 p.D254Y mutation with loss of heterozygosity and a splice donor variant of STK11 gene with loss of heterozygosity were identified.

**Case 76**	M/48	**Stomach cancer**	**Utility: Category I-2**
**Comments for Clinical Utility**	A patient with gastric cancer with BRCA2 germline mutation and HRD may be considered for a clinical trial of a PARP inhibitor. **[Category I-2 Clinical trial, NCT04171700]**		

Clinical Presentation> A 48-year-old man with no significant past medical history presented with upper gastrointestinal endoscopy for epigastric pain of 3 months' duration, which revealed an ulcerated infiltrative mass of more than 7 cm in the posterior wall of the lower body. The histologic examination performed was reported as poorly differentiated adenocarcinoma. The patient's mother had a history of ovarian cancer. Clinical staging was reported as cT4aN2 and total gastrectomy with Billroth II Braun was performed. Pathologic examination of surgical tissue revealed adenocarcinoma, poorly differentiated pT4bN3a, and station #8 lymph node encasing the common hepatic artery resulting in incomplete resection (R2). Palliative nivolumab+capecitabine+oxaliplatin regimen was administered. A hypermetabolic lesion was confirmed in the pancreatic head prior to surgery and shrinkage was observed after chemotherapy. The patient remains on nivolumab + capecitabine with progression free at 11 months.

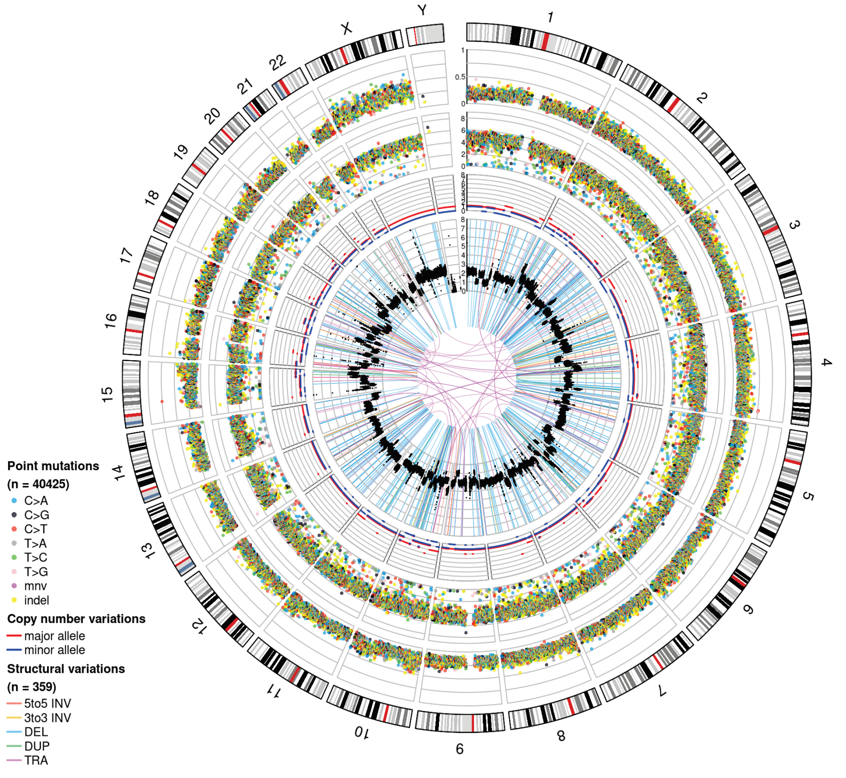

**Genome interpretation>**	Depth: Tissue: 44.5X, Normal: 17.7X	Total mutation counts: SNV: 30,967 Indel: 7,886 SV: 360
	Tumor fraction: 0.3, Mean ploidy: 1.8	Germline: not specific findings

This sample was deficient for homologous recombination pathway yielding HRD score of 0.84 (cutoff 0.7). We identified germline BRCA2 mutation (p.Cys717Ter) and somatic BRCA2 stopgain mutation (p.S511*) mutation.

**Case 77**	M/72	**Head and Neck**	**Utility: Category I-2**
**Comments for Clinical Utility**	There is an eligible trial for solid tumors with AKT1 mutations **[Category I-2 Clinical trial, NCT05172245]**.		

Clinical Presentation> A 72-year-old man was referred to our tertiary center for left neck swelling after an implant procedure at his dental clinic two weeks ago, with findings of tonsillar carcinoma after neck ultrasound and CT. He had an ulcerative and infiltrative tumor measuring 3.7 cm in the right palatine tonsil, with regional lymph node involvement confirmed, and clinical staging was cT4aN2M0, HPV+. right wide excision with left lateral neck dissection and right modified radical neck dissection were performed. Surgical histopathology confirmed squamous cell carcinoma, HPV-associated. Post operative concurrent chemoradiation therapy with cisplatin was performed. She is being followed up without recurrence up to 12 months postoperatively.

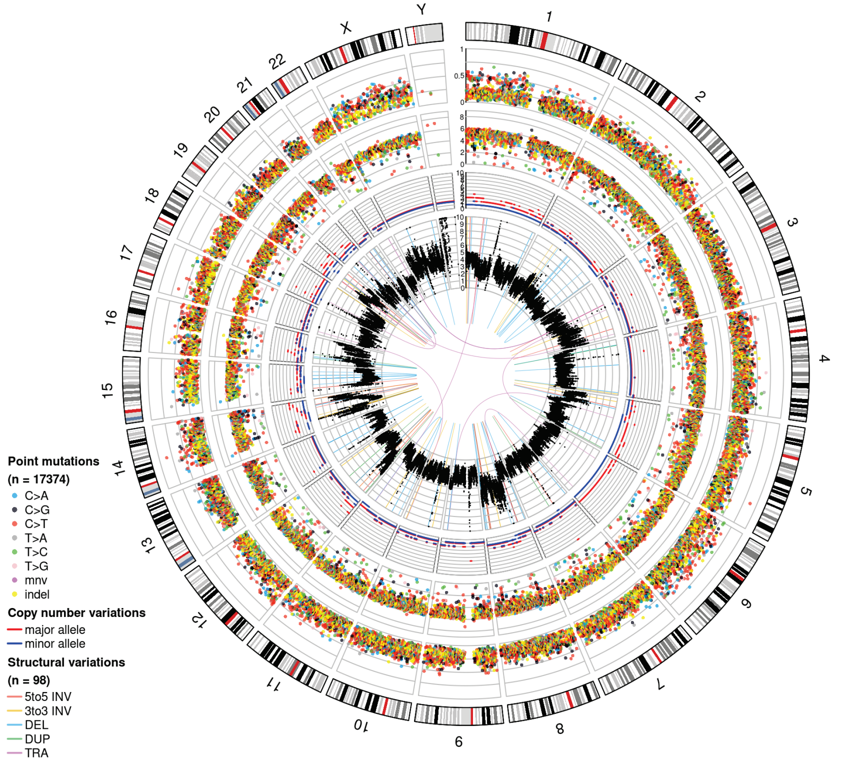

**Genome interpretation>**	Depth: Tissue: 46.8X, Normal: 18.0X	Total mutation counts: SNV: 13,510 Indel: 4062 SV: 85
	Tumor fraction: 0.54, Mean ploidy: 2.8	Germline: not specific findings

AKT1 p.E17K mutation has been identified.

**Case 78**	F/48	**Breast cancer**	**Utility: No (I-1)**
**Comments for Clinical Utility**	No actionable driver was reported.		

Clinical Presentation> A 48-year-old woman presents with a right breast mass identified on physical examination. A 2.5 cm mass and a right axillary level 1 metastatic lymph node were identified in the right breast at 9 hour. Histology confirmed invasive ductal carcinoma and ER/PR/Her-2: 8+/8+/- ki-67 50%. Her sister was diagnosed with breast cancer 5 years ago. Neoadjuvant adriamyin + cyclophosphamide followed by docetaxel regimen was administered with clinical stage cT2N1M0. Response Evaluation confirmed partial response and partial mastectomy with sentinel lymph node dissection was performed. Pathologic examination of surgical tissue confirmed invasive ductal carcinoma with focal neuroendocrine differentiation ypT1N1. Adjuvant radiotherapy was performed and she has remained recurrence free for 1 year after surgery with adjuvant anastrozole therapy.

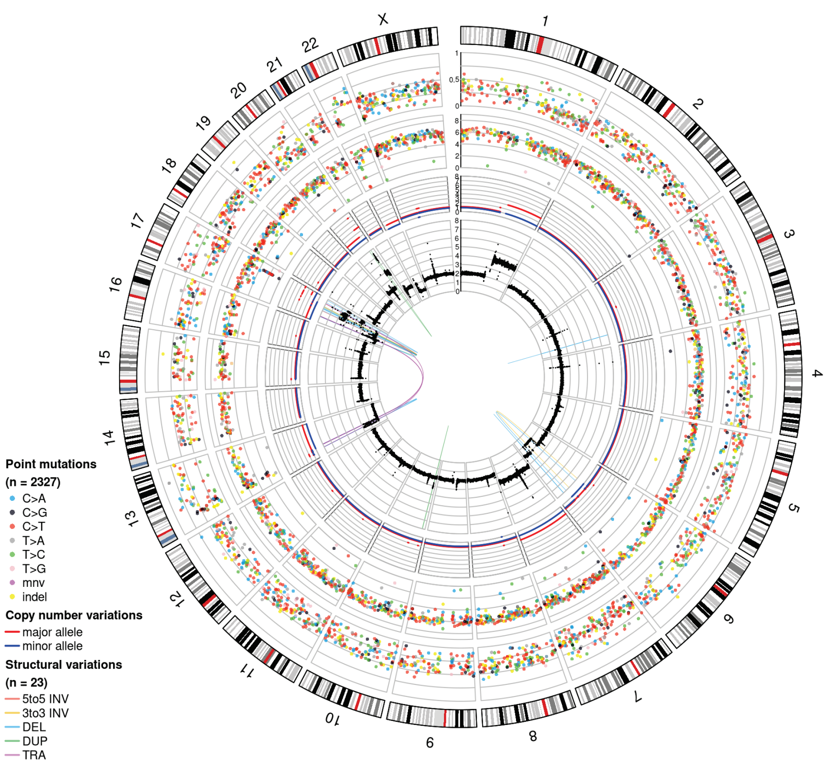

**Genome interpretation>**	Depth: Tissue: 44.5X, Normal: 20.9X	Total mutation counts: SNV: 2,067 Indel: 261 SV: 23
	Tumor fraction: 0.77, Mean ploidy: 2.1	Germline: not specific findings

Chromothripsis at chr17 p-arm.

**Case 79**	M/45	**Colorectal cancer**	**Utility: Category I-3**
**Comments for Clinical Utility**	Mutated KRAS in colorectal cancer renders it resistant to EGFR-targeting antibodies such as cetuximab **[Category I-3, Dropping ineffective options]**.		

Clinical Presentation> A 45-year-old man with no unusual medical history presented with anal bleeding, tenesmus, and stool caliber change, and colonoscopy AV 6 cm revealed an ulcerofungating mass enclosing a lumen. Histology confirmed adenocarcinoma, moderately differentiated, with metastases to liver, lung and T12 spine, clinical staging was cT4N2M1. He was treated with bevacizumab + mFOLFOX6 as palliative chemotherapy and achieved a partial response. After a total of 14 cycles, liver progression was confirmed and treatment was changed to afilbercept + FOLFIRI. Bevacizumab + trifluridine/tipiracil regimen was initiated and progression was confirmed at assessment after 3 cycles and subsequent treatment with 5-FU + cisplatin and capecitabine was initiated, all with initial progression. The patient died 19 months after diagnosis.

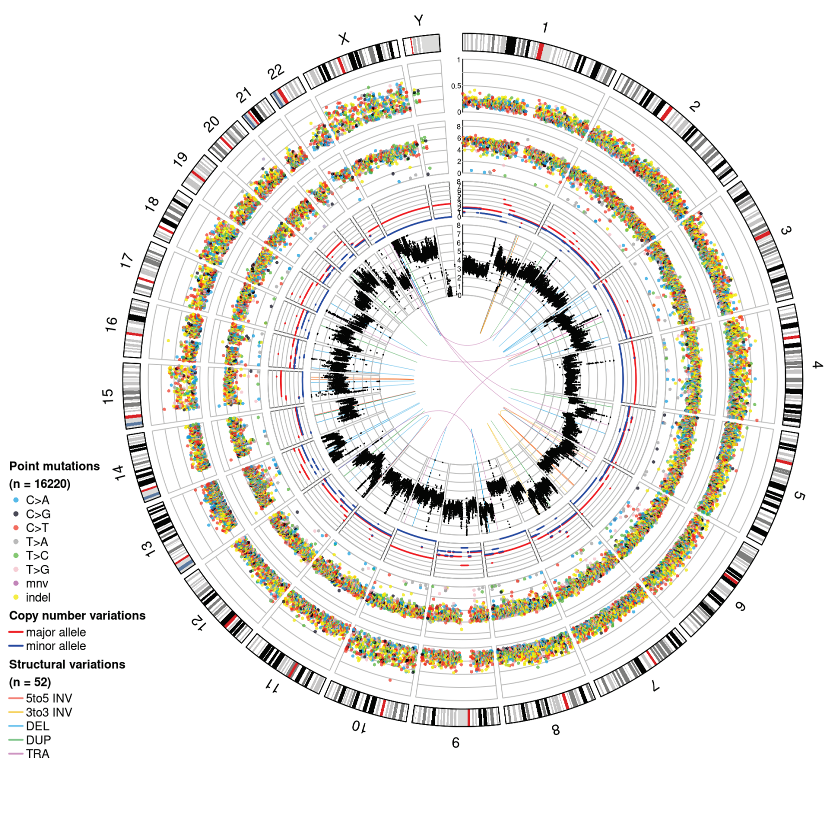

**Genome interpretation>**	Depth: Tissue: 48.2X, Normal: 24.8X	Total mutation counts: SNV: 12,549 Indel: 3673 SV: 52
	Tumor fraction: 0.25, Mean ploidy: 4.1	Germline: not specific findings

KRAS p.Q61L mutation and double mutations in APC gene (p.E1550ffs* and transposable element insertion in exon15) were identified.

Case 80	F/53	Pancreatic cancer	Utility: Category I-2
**Comments for Clinical Utility**	For KRAS G12C mutation, available drugs are in development and several clinical trials are underway in pancreatic cancer **[Category I-2 Clinical trial, NCT03065387, NCT04892017].** .		

Clinical Presentation> A 53-year-old woman was receiving systemic chemotherapy for metastatic pancreatic cancer and presented for a second opinion about surgical treatment due to increasing ovarian metastases. The patient underwent a pancreaticoduodenectomy for caudal pancreatic cancer 4 years ago and completed 12 cycles of mFOLFIRINOX as adjuvant chemotherapy. However, the tumor progressed 8 months after completion and she received palliative gemcitabine + nab-paclitaxel for 26 months, followed by erlotinib, liposomal irinotecan + 5-FU, and tegafur/gimeracil/oteracil. After 30 months of systemic therapy, no additional anti-cancer agents were available for the massive right ovarian mass of 12 cm, and a multidisciplinary tumor board was conducted to evaluate opinions regarding surgery for palliation. The gynecologic surgeon commented that surgery would not benefit the patient, and the medical oncologist suggested that the genomic alterations be evaluated to determine the possibility of a clinical trial for a novel drug or an early access program. A US-guided core needle biopsy was performed on the abdominal wall, panel NGS was performed, and the patient was enrolled in the WGS study. While awaiting the panel NGS report, she received 2 cycles of gemcitabine + cisplatin as salvage chemotherapy, but the disease progressed. KRAS G12C was confirmed by both panel and WGS, and she was transferred to a hospital with an available KRAS inhibitor clinical trial. She remained progression free for 4 months after enrollment.

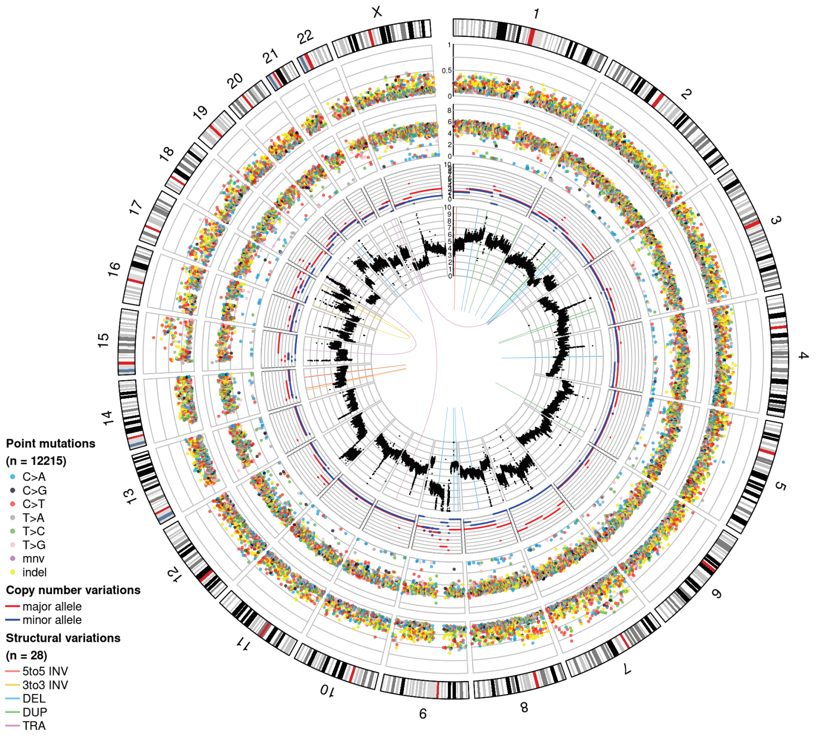

**Genome interpretation>**	Depth: Tissue: 50.9X, Normal: 24.6X	Total mutation counts: SNV:10,017 Indel: 2199 SV: 28
	Tumor fraction: 0.28, Mean ploidy: 4.4	Germline: not specific findings

KRAS p.G12C mutation, TP53 p.R213* mutation with loss of heterozygosity, SMAD2 p.R427Q, CDKN2A biallelic deletion were identified.

Case 81	M/79	Stomach cancer	Utility: Category I-1
**Comments for Clinical Utility**	Pembrolizumab is indicated for the metastatic solid cancer with microsatellite high (MSI-H), including gastric cancer **[Category I-1 FDA approved drug]**		

Clinical Presentation> A 79-year-old man presented with a huge size ulcerofungating mass in the mid body lesser curvature, posterior wall on upper gastrointestinal endoscopy performed for heartburn. Histology reported adenocarcinoma, moderately differentiated. Clinical staging was cT4aN1M0 and distal gastrectomy with Billroth II Braun via laparoscopy was performed. Invasion from station #12a lymph node to the pancreas was confirmed and R1 resection was performed. Pathology of the surgical specimen confirmed tubular adenocarcinoma, poorly differentiated, and was reported as pT4aN3b. Final pathology confirmed metastases in the LN of #12 and additional post-operative radiotherapy was performed based on the findings of the R1 resection. After radiotherapy, capecitabine + oxaliplatin was administered in the palliative setting. After 5 cycles, the patient was admitted to the emergency department with neutropenic fever and left lower abdominal pain, and died in the hospital within 24 hours due to septic shock with pneumonia and enterocolitis.

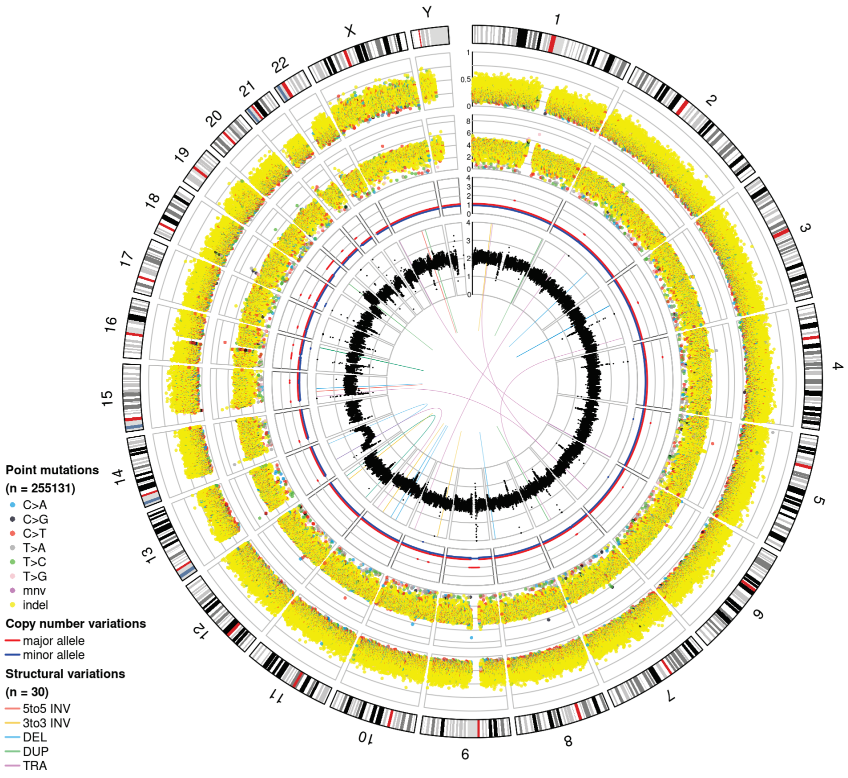

**Genome interpretation>**	Depth: Tissue: 40.3X, Normal: 15.0X	Total mutation counts: SNV: 46,381 Indel: 151,030 SV: 26
	Tumor fraction: 0.20, Mean ploidy: 2	Germline: not specific findings

This sample has deficient DNA mismatch repair pathway evidenced by high number of indel and high proportion of SBS44.
Oncogenic mutations in BRAF gene (p.N581S) and ERBB2 gene (p.R678Q) were identified.

**Case 82**	M/41	**Non-smal cell lung cancer**	**Utility: No (I-1)**
**Comments for Clinical Utility**	Less is known about the clinical implications of the BRAF G509V mutation and more research is needed.		

Clinical Presentation> A chest CT performed for persistent cough 2 months before the visit showed peribronchiolar ground-glass opacities with fuzzy borders and dense consolidations in the right upper lobe, suggestive of pulmonary tuberculosis; sputum AFB stain, culture, and TB PCR were all negative, and the lesions showed some improvement after antibiotic treatment for pneumonia. Follow-up chest CT was performed for persistent ground-glass opacities, and at 2 years, the lesion increased in size, and percutaneous lung biopsy was performed and reported atypical mucous epithelium suggestive of invasive mucinous adenocarcinoma. Imaging studies revealed two additional separate lesions in the right lower lobe. Under the impression of non-small cell lung cancer, adenocarcinoma, clinical staging cT1bN0M0, the patient was referred to thoracic surgery and underwent right lower lobectomy via video-assisted thoracic surgery. Surgical tissue pathology confirmed invasive mucinous adenocarcinoma, pT3N0 PL1. Four cycles of pemetrexed + cisplatin were administered as adjuvant chemotherapy. During post-operative follow-up, an ill-defined ground glass lesion was identified in the left lower lung, but it is not a clear sign of cancer, so he is on active surveillance and has completed 10 months of post-operative follow-up.

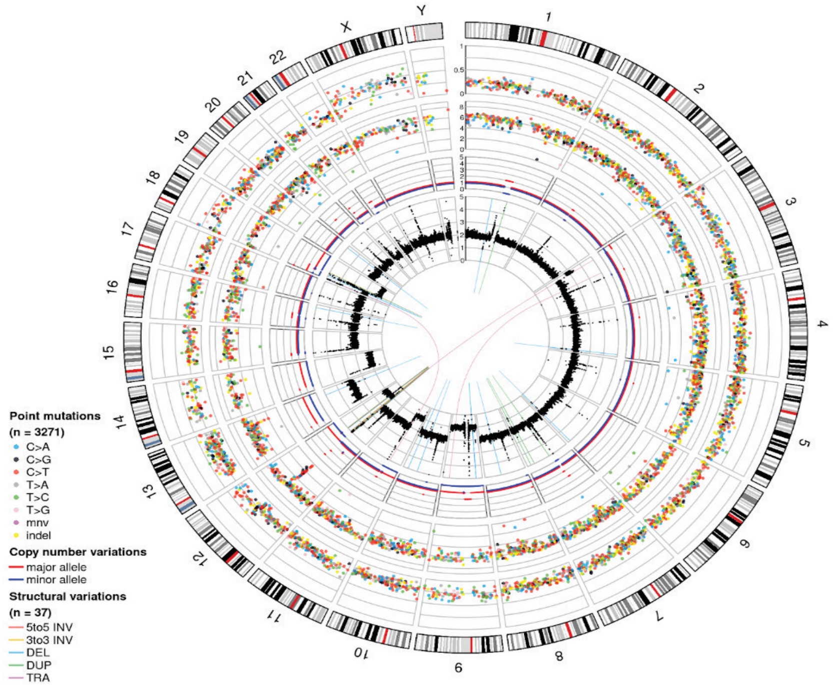

**Genome interpretation>**	Depth: Tissue: 53.6X, Normal: 21.6X	Total mutation counts: SNV: 2,807 Indel: 467 SV: 37
	Tumor fraction: 0.45, Mean ploidy: 1.8	Germline: not specific findings

BRAF p.G509V mutation and CDKN2A biallelic deletion were identified.

Case 83	M/71	Adenocarcinoma of Unkown Primary	Utility: Category II-2
**Comments for Clinical Utility**	A patient diagnosed with carcinoma of unknown primary (CUP) and suspected to have pancreatic cancer showed an alteration profile commonly identified in pancreatic cancer, such as HRD and BRCA2 SNVs. PARP inhibitors may be considered for BRCA2 SNV, but there is no approved indication for CUP. **[Category II-2 Tumor orgin]**		

Clinical Presentation> A 71-year-old man presented with persistent abdominal pain for 1 month, and laparoscopic excisional biopsy of a 2.7 cm mass in the omentum confirmed metastatic adenocarcinoma (immunohistochemical staining: CK7 and CDX2 positive). Abdominal CT and PET-CT, esophagogastroduodenoscopy and colonoscopy showed no findings to identify the primary cancer, only multiple peritoneal masses. Tumor markers such as CEA, PSA, CA19-9 were all normal. The pathology findings considered the possibility of upper GI or pancreatobiliary origin, but there were no imaging and endoscopic findings consistent with primary cancer, so adenocarcinoma of unknown origin was diagnosed. Palliative paclitaxel + carboplatin regimen was performed and response was assessed as stable disease and 6 cycles were performed. Peritoneal nodal progression was noted and palliative gemcitabine + cisplatin regimen was administered and best of response was noted as stable disease and progression was confirmed after 4 cycles. The patient was treated with nab-paclitaxel + 5-FU + leucovorin regimen and showed best of response with stable disease at first response evaluation, but progression in liver and peritoneal seeding was noted after 6 cycles. mFOLFOX6 was administered for 11 cycles and a partial response was noted. Subsequently, nivolumab was administered for 2 cycles, but the patient developed liver progression and died 18 months after diagnosis.

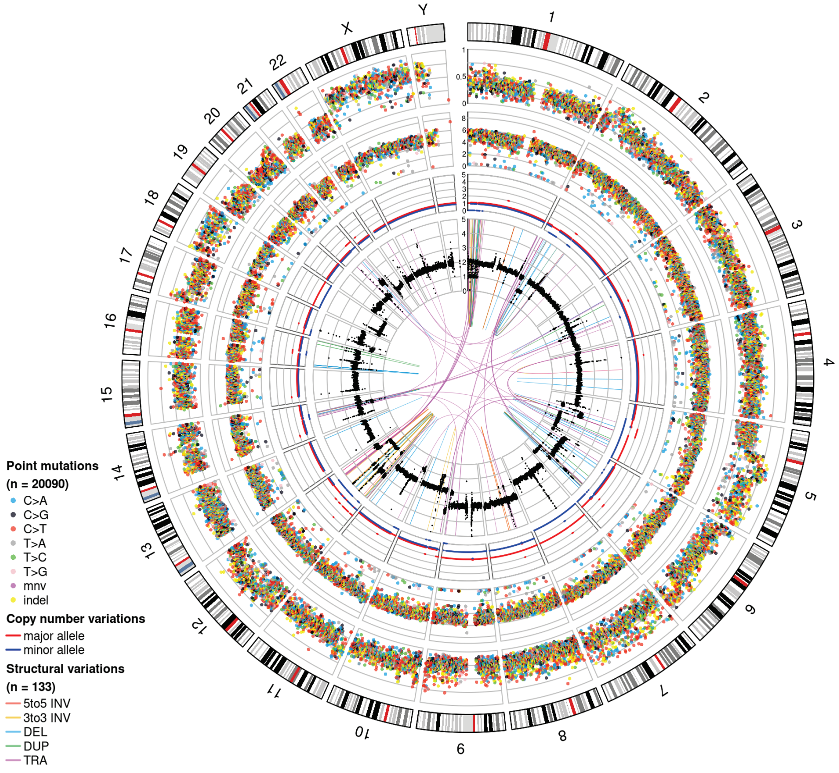

**Genome interpretation>**	Depth: Tissue: 36.6X, Normal: 26.6X	Total mutation counts: SNV: 16,722 Indel: 3,369 SV: 133
	Tumor fraction: 71, Mean ploidy: 1.9	Germline: not specific findings

Mutational analysis revealed a proportion of homologous recombination deficiency-related mutational process (SBS3, SBS8).
BRCA2 frameshift mutation (p.Thr3033AsnfsTer11) without loss of heterozygosity and a structural variation (deletion) disrupting RAD51B gene with loss of heterozygosity were identified.

<table>
<tr><td>Case 84</td><td>F/37</td><td>Biliary cancer</td><td>Utility: Category I-2</td><td rowspan="3">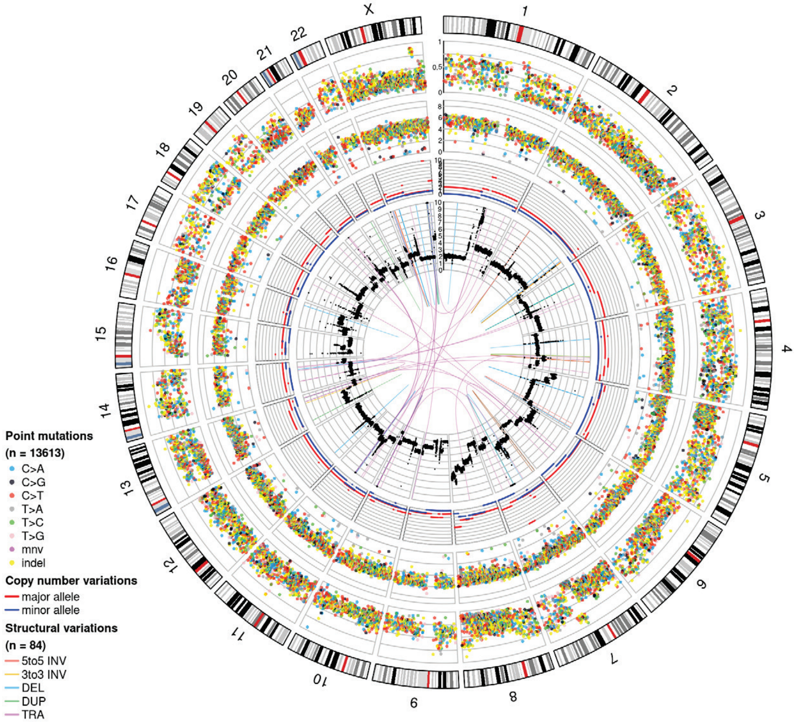
</td></tr>
<tr><td colspan="2">Comments for Clinical Utility</td><td colspan="2">Pemigatinib is approved for biliary cancer with FGFR2 rearrangement and fusion.This patient received pemigatinib because targeted panel sequencing (TPS) reported EML4-FGFR2 fusion. No FGFR2 rearrangement was identified by WGS, suggesting that heterogeneity due to differences in tumor tissue is a possible explanation. The patient also has a mixed response, with some lesions showing response and others showing progression after pemigatinib. [Category I-2 Clinical trial, NCT03230318]</td></tr>
<tr><td colspan="4">Clinical Presentation> A 37-year-old woman with no unusual medical history presented with persistent postprandial nausea and vomiting for 1 week and was referred to a tertiary center because endoscopy revealed a submucosal tumor in the cardia region of the stomach. Abdominal CT revealed a 6 cm exophytic soft tissue mass in the cardia region and multiple liver metastases, endoscopic ultrasound-guided biopsy revealed a poorly differentiated carcinoma, and liver histology revealed a combined hepatocellular carcinoma-cholangiocarcinoma (ccHCC-CCC). The patient was diagnosed with ccHCC-CCC with direct invasion from the liver to the gastrointestinal cardia and paraaortic, both common iliac, left external and internal iliac LN metastases. He was confirmed with AFP 2.2 ng/ml, PIVKAII 35mAU/ml, CA19-9 >10,000 U/ml, and was given nab-paclitaxel + gemcitabine + cisplatin regimen as palliative chemotherapy for biliary cancer. best response confirmed partial response and showed continuous response up to 8 cycles. She complained of neck pain, and bone metastases were confirmed in the cervical spine, so palliative radiotherapy was performed. One month after radiotherapy, relapse was confirmed in the liver and new ovarian metastases were confirmed. She was treated with pemigatinib for FGFR2 amplification and fusion and showed a partial response, and her general condition is improving. She has been on pemigatinib for 2.5 months and showed a mixed response. The masses around the liver and stomach decreased significantly, but the right ovarian mass increased in size to more than 14 cm, and palliative oophorectomy was performed. She is taking pemigatinib and awaiting her next tumor response evaluation.</td></tr>
<tr><td colspan="2" rowspan="2">Genome interpretation></td><td>Depth: Tissue: 50.4X, Normal: 22.2X</td><td colspan="2">Total mutation counts: SNV: 10,157 Indel: 3,456 SV: 84</td></tr>
<tr><td>Tumor fraction: 59, Mean ploidy: 3.8</td><td colspan="2">Germline: not specific findings</td></tr>
<tr><td colspan="5">FGFR2 (copy number 184.65), CCND1 (23.87), CDK6 (18.2) amplification were identified.</td></tr>
</table>

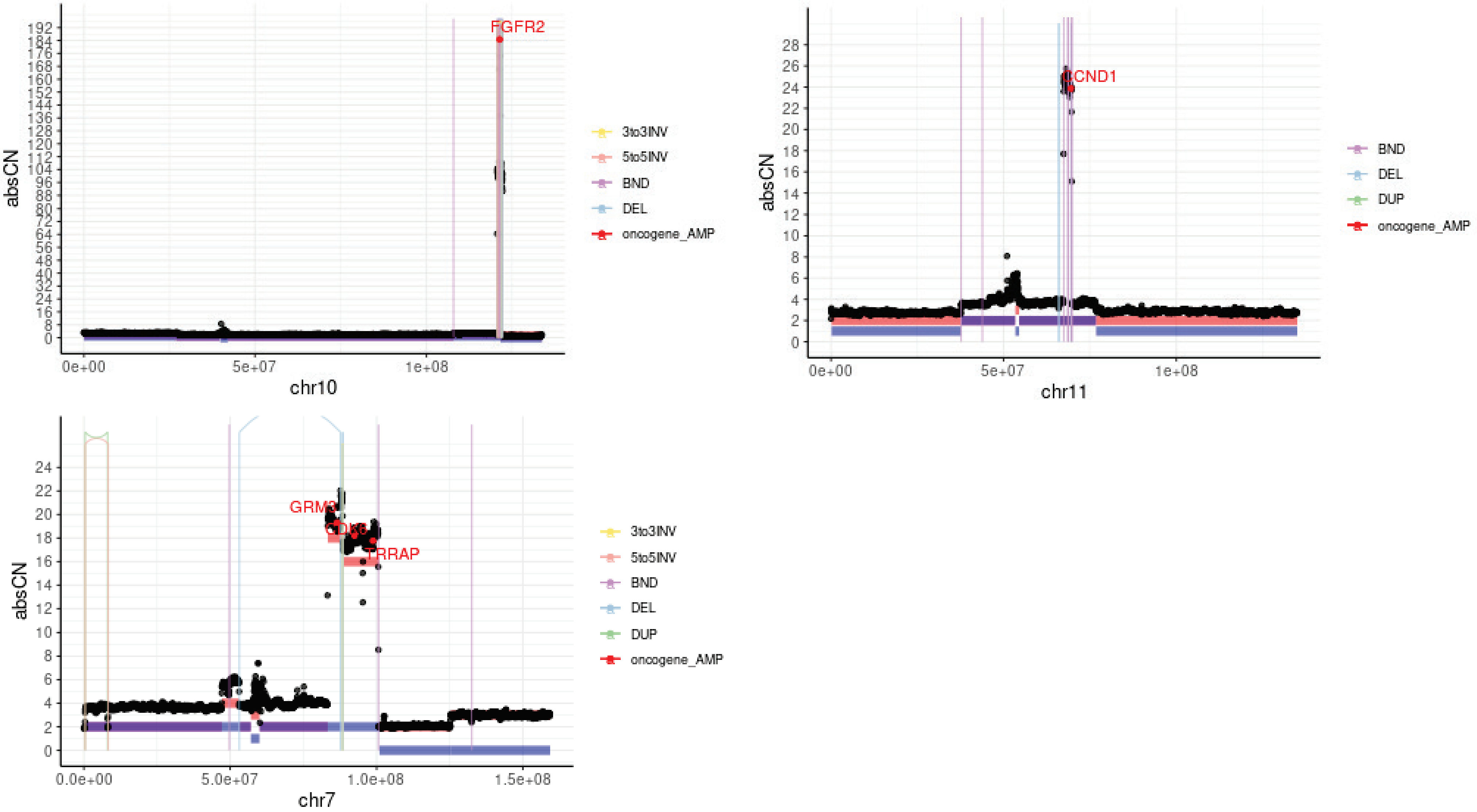
FGFR2
absCN
chr10
0e+00
5e+07
1e+08
3to3INV
5to5INV
BND
DEL
oncogene_AMP
CCND1
absCN
chr11
BND
DEL
DUP
oncogene_AMP
GRM3
CDK6
TRRAP
absCN
chr7
0.0e+00
5.0e+07
1.0e+08
1.5e+08
3to3INV
5to5INV
BND
DEL
DUP
oncogene_AMP

**Case 85**	F/51	**Breast cancer**	**Utility: Category I-2**
**Comments for Clinical Utility**	PIK3CA E545K is a hotspot mutation that allows alpelisib to be administered in hormone positive metastatic breast cancer. This patient has TNBC, which is not a direct actionable alteration and constitutes off-label use. There are clinical trials available for enrollment in TNBC with PIK3CA mutations. **[Category I-2 Clinical trial, NCT04251533, NCT04216472]**		

Clinical Presentation> A 51-year-old woman presented with a palpable left breast mass of 3 weeks' duration. Metaplastic carcinoma was confirmed in a 2.5 cm mass in the left lower outer breast, and a partial mastectomy with sentinel lymph node dissection was performed. On postoperative pathological examination, the final diagnosis was malignant adenomyoepithelioma with metaplastic carcinoma features and staging was reported as pT2. estrogen receptor, progresterone receptor, her-2 receptor negative findings were reported. adjuvant docetaxel + cyclophosphamide 4 cycles, adjuvant radiotherapy was performed. The patient was found to have a local recurrence in the left breast on follow-up imaging 13 months after radiotherapy, and a total mastectomy was performed. A multidisciplinary tumor board was performed and additional chemotherapy was decided as a patient with high risk of recurrence and poor prognosis. She was reported as PD-L1 CPS 30 (22C3 pharmDx) and received pembrolizumab + gemcitabine + carboplatin as palliative systemic therapy for TNBC. She has remained progression -free for 7 months after 10 cycles and is on active surveillance with a rest from chemotherapy.

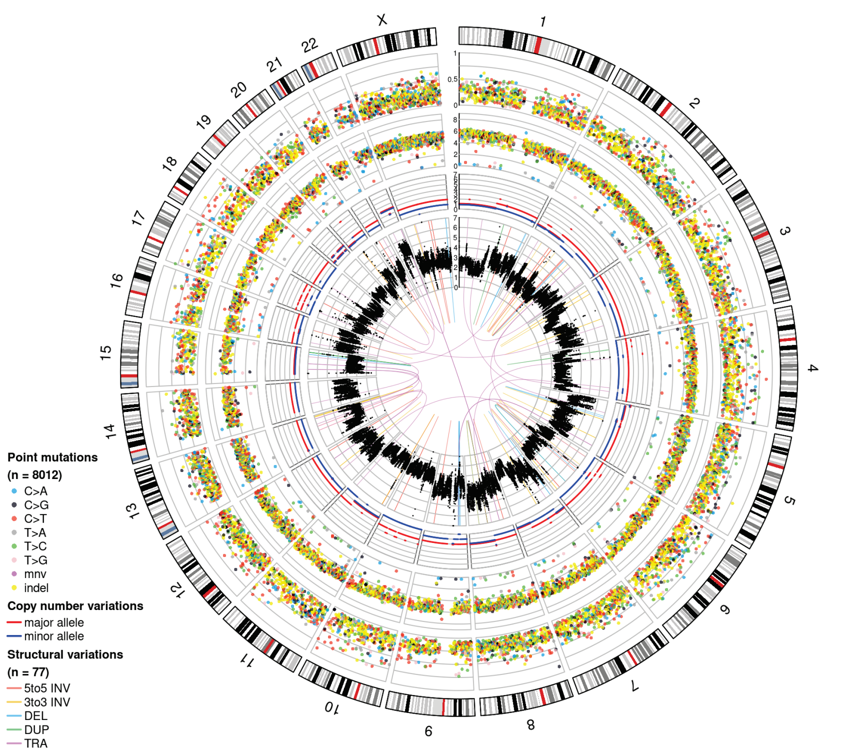

**Genome interpretation>**	Depth: Tissue: 53.4X, Normal: 22.8X	Total mutation counts: SNV: 5,312 Indel: 2,701 SV: 77
	Tumor fraction: 68, Mean ploidy: 2.7	Germline: not specific findings

PIK3CA (p.E545K), HRAS (p.G12C) oncogene mutations, and TERT promoter mutation have been identified.

**Case 86**	M/60	**Stomach cancer**	**Utility: No (Category 1-1)**
**Comments for Clinical Utility**	This patient has gastric cancer with CCNE1 amplification, which is known to be associated with poor prognosis. (Annals of Oncology. 2015;26(2):438-9) [Category III] In addition, the BRCA2 SNV was identified (p.Ser2554Gly) and there was no loss of heterozygosity (LOH).		

Clinical Presentation> A 60-year-old man with severe aortic regurgitation and atrial fibrillation was found to have cancer in the gastric esophagus on gastroscopy performed during a routine check-up. Endoscopic biopsy confirmed a well-differentiated adenocarcinoma and endoscopic submucosal dissection was performed for early gastric cancer. Pathologic evaluation confirmed submucosal invasion (pT1b, sm invasion 0.22cm), venous invasion and carcinoma involvement in the deep resection margin. As the patient required additional surgery, a laparoscopic duodenal gastrectomy was performed and a comprehensive pathologic examination with serial sections was performed, which confirmed the results of no residual carcinoma with no regional lymph node metastases (out of 24 lymph nodes). The final pathologic staging was confirmed as pT1bN0M0. There was a complication of stump leakage at postoperative week 3, but he fully recovered and is being followed up with no recurrence at 6 months postoperative F/U.

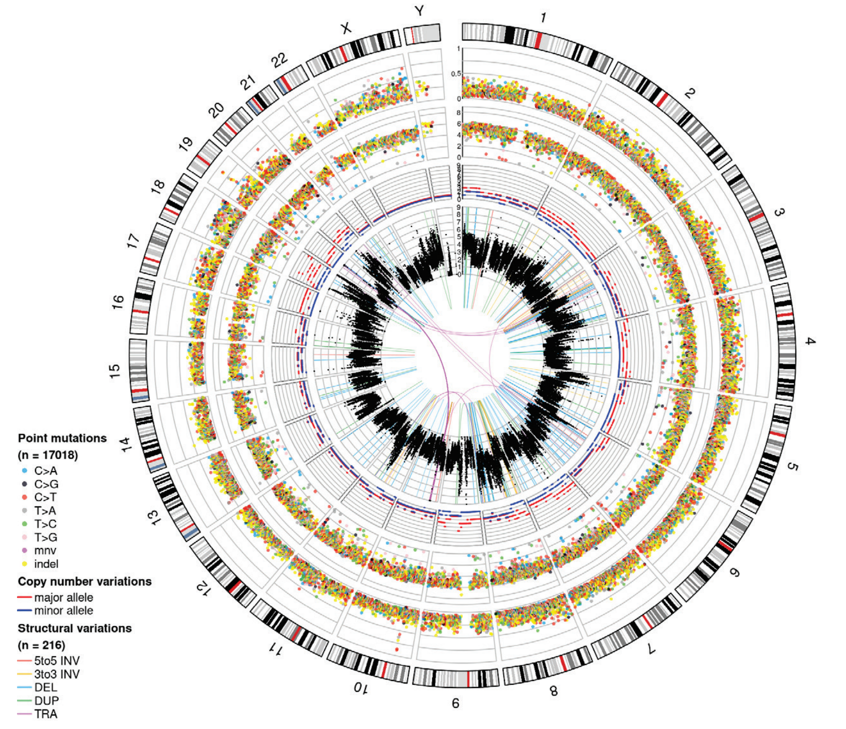

**Genome interpretation>**	Depth: Tissue: 64.9X, Normal: 21.9X	Total mutation counts: SNV: 13,440 Indel: 3,585 SV: 216
	Tumor fraction: 27, Mean ploidy: 2.6	Germline: not specific findings

CCNE1 focal amplification (copy number of 50.68).

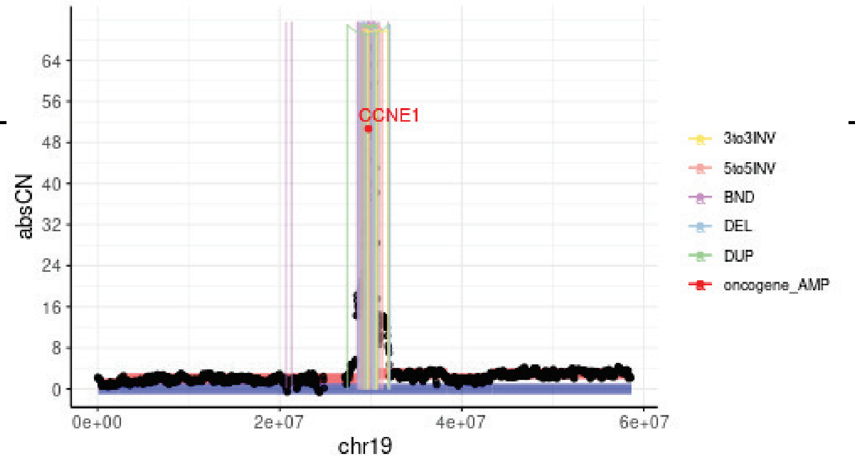

Case 87	M/70	Stomach cancer	Utility: Category II-2
**Comments for Clinical Utility**		The patient had adenocarcinoma confirmed in the GB during surgery for gastric cancer. The clinical context suggested that the stomach and GB were likely separate adenocarcinomas. In practice, the distinction between a double primary and a cancer that has metastasized elsewhere is an important clinical assessment, but it is very difficult to give a definitive answer. Pathologic differentiation between adenocarcinomas of the two digestive organs is often limited, with immunohistochemical stains often indistinguishable. Currently, it is common practice to rely on the opinion of the pathologist based on microscopic and histopathologic similarities. Panel NGS can be used to try to differentiate between the two, but since only a small set of genes is used for comparison, there is not enough confidence that it is 100% accurate. In this patient, we performed WGS on both the GB adenocarcinoma and the stomach adenocarcinoma and found that the driver mutation profiles did not overlap at all. This allowed us to conclude that the two cancers were different primary cancers.**[Category II-2 Tumor orgin]**	

Clinical Presentation> A 70-year-old man with a history of hypertension presented with symptoms of heartburn and gastroscopy revealed a cancer in the upper part of the stomach. Endoscopic biopsy confirmed a well-differentiated adenocarcinoma and clinical stage was confirmed as cT2N0M0, so total gastrectomy was performed. Postoperative pathology confirmed mixed tubular adenocarcinoma, well differentiated, with lymphatic and perineural invasion and carcinoma involvement at the proximal resection margin. Surgical staging was reported as pT4aN1. In addition, preoperative CT showed chronic cholecystitis with gallbladder stone, so cholecystectomy was performed and reported as mucinous adenocarcinoma, pT2. There was a problem that R1 resection was performed for gastric cancer and complete resection including lymphadenectomy could not be performed for gallbladder cancer due to incidental discovery during gastric cancer surgery. The multidisciplinary tumor board discussed the treatment plan and decided to administer Capecitabine + Oxaliplatin (CapOx) as palliative systemic therapy for gastric cancer. After 8 cycles of CapOx, he is on active surveillance and has been followed for 4 months without recurrence.

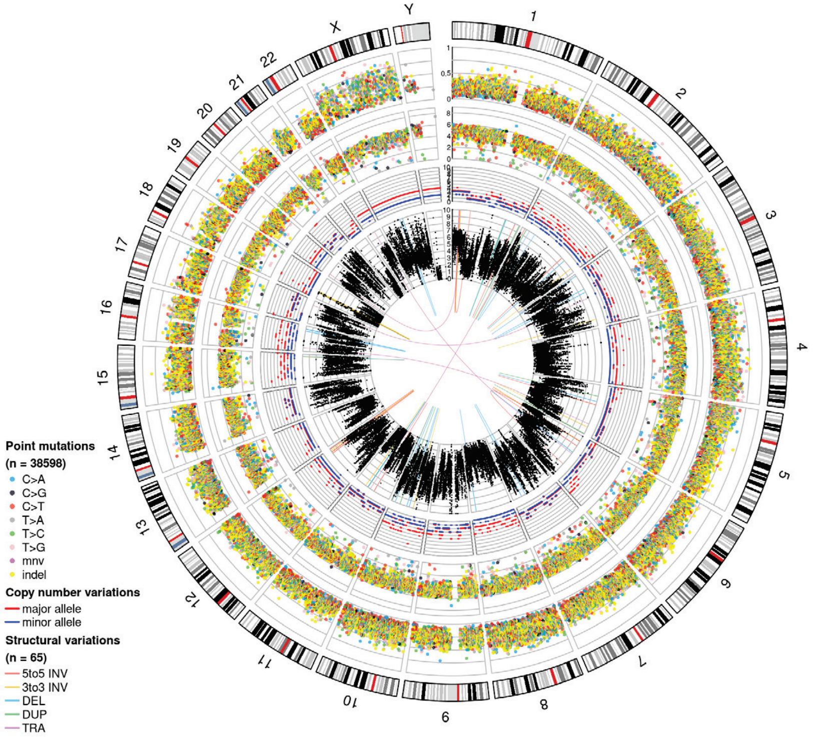

**Genome interpretation>**			
	Depth: Tissue: 54.2X, Normal: 16.2X	Total mutation counts:	SNV: 25,160 Indel: 10,060 SV: 65
	Tumor fraction: 31.0, Mean ploidy: 3.7	Germline:	not specific findings

A high proportion of SBS17b mutational signature (unknown etiology) and SBS18 (damage by reactive oxygen species) was observed.

Supplementary Table 3

<table>
<tr><th>Stomach</th><th>Gallbladder</th></tr>
<tr><td>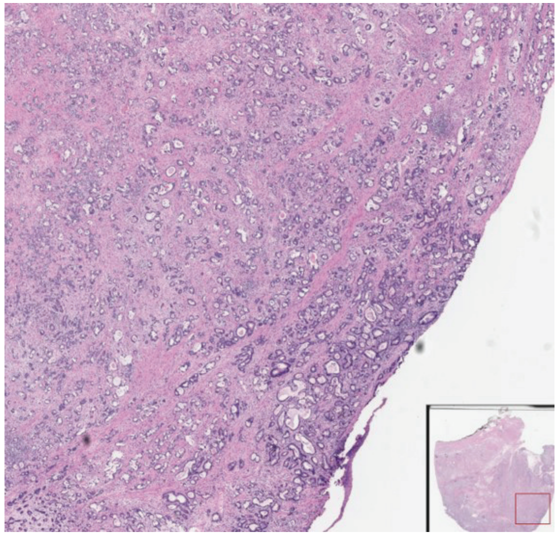</td><td>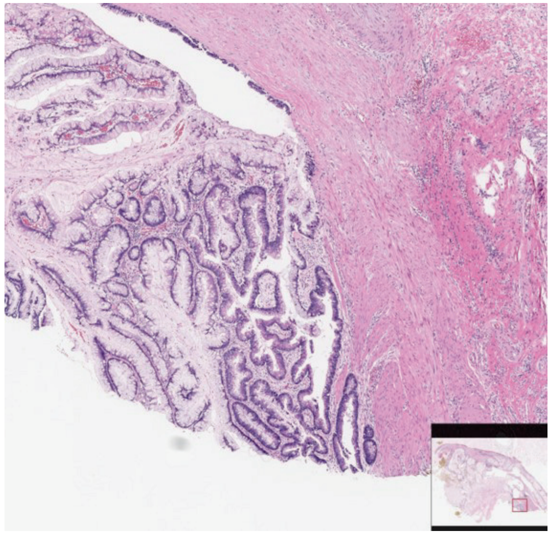</td></tr>
<tr><td>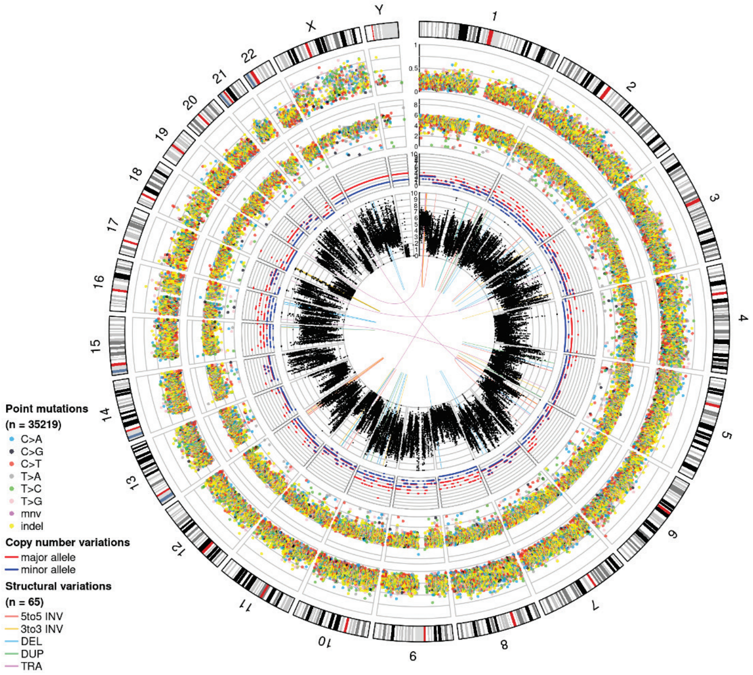
</td><td>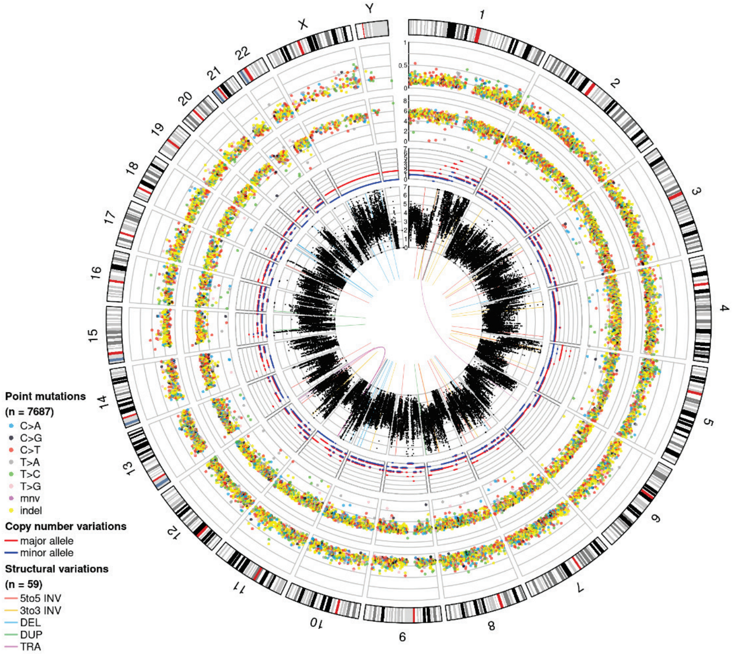
</td></tr>
</table>

**Case 88**	F/49	**Breast cancer**	**Utility: Category I-2**
**Comments for Clinical Utility**	The patient is germline BRCA1/2 wild type but has a confirmed BRIP gene deletion with loss of heterogeneity, and a homologous repair defect was identified in the cancer whole genome. This patient is an HRD case of BRCA wild type, sensitive to platinum based chemotherapy, and may be a candidate for PARP inhibitor administration. **[Category I-2 Screening for mutation-specific clinical trials NCT02264678]**		

Clinical Presentation> A 49-year-old woman with no significant past medical history presented with a palpable mass in the right breast since 1 month ago, and the histological examination confirmed triple-negative breast cancer. The clinical stage was confirmed as cT2N1M0. Neoadjuvant chemotherapy with doxorubicin + cyclophosphamide followed by docetaxel therapy and total mastectomy was performed. Postoperative staging confirmed ypT1cN1a and adjuvant capecitabine therapy was performed. 3 months after completion of adjuvant therapy, imaging revealed local replase of the right axillary lymph node and axillary lymph node excision was performed. An oligo-metastatic lesion in the pubic bone was confirmed and palliative radiotherapy was performed. However, liver metastases were identified on follow-up imaging 2 months later and palliative gemcitabine + carboplatin therapy was administered. He achieved a partial response on tumor response assessment and was enrolled in a PARP inhibitor clinical trial after 6 cycles.

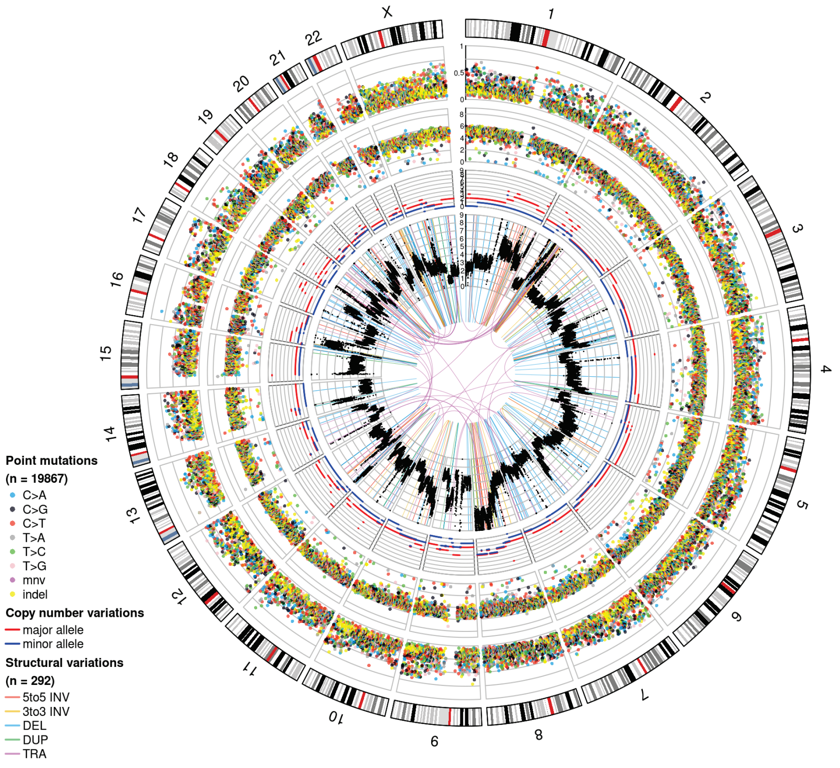

**Genome interpretation>**	Depth: Tissue: 66.5X, Normal: 23.2X	Total mutation counts: SNV: 15,954 Indel: 3,914 SV: 292
	Tumor fraction: 52, Mean ploidy: 3.3	Germline: not specific findings

A high proportion of SBS3 (homologous recombination deficiency) and non-clustered large deletion and insertions thoughout the entire genome were observed, implying deficient homologous recombination pathway.
An oncogenic ERBB3 mutation (p.Glu928Gly) was identified.

**Case 89**	F/58	**Breast cancer**	**Utility: No (Category 1-1)**
**Comments for Clinical Utility**	This patient has high mutation burden hormone positive metastatic breast cancer by APOBEC mutagenesis. It has been reported that hormone-positive breast cancer with APOBEC mutagenesis has a poor outcome to CDK4/6 inhibitor and endocrine therapy (JCO Precision Oncology 2022:6), which may explain the short PFS duration of abemaciclib + letrozole therapy in this patient. APOBEC mutagenesis has been reported to be associated with high tumor mutational burden and good response to immune checkpoint inhibitors. (Annals of Oncology. 2020;31(3):387-94.) This patient has TMB 11.03 mutations/megabase on a whole-genome basis, and pembrolizumab is approved for TMB 10 mutations/megabase. No actionable driver was reported.		
Clinical Presentation> A 58-year-old woman with a history of hypertension and diabetes mellitus presented with a palpable left breast mass for 3 months, and histology confirmed estrogen receptor and progesterone positive and her-2 negative invasive ductal carcinoma. The clinical stage was confirmed as cT3N3M1 with mediastinal nodal involvement. She was started on palliative abemaciclib + letrozole regimen and 6 months later an increase in the size of the main breast mass and generalized lymph node progression including left axillary and supraclavicular was noted. She was started on fulvestrant as second-line systemic therapy, but progression of the primary breast mass was confirmed 2 months later. The patient underwent clinical panel sequencing (TruSight Oncology 500) and a tumor mutation burden (TMB) of 24.2 mutations per megabase was reported. A clinical trial for TMB-high at data cut-off is being considered.			
**Genome interpretation>**	Depth: Tissue: 45.0X, Normal: 22.5X	Total mutation counts: SNV: 34,198 Indel: 5,872 SV: 262	
	Tumor fraction: 40, Mean ploidy: 3.5	Germline: not specific findings	
A high proportion of APOBEC signature with germline APOBEC3A/3B homozygous deletion was observed.			

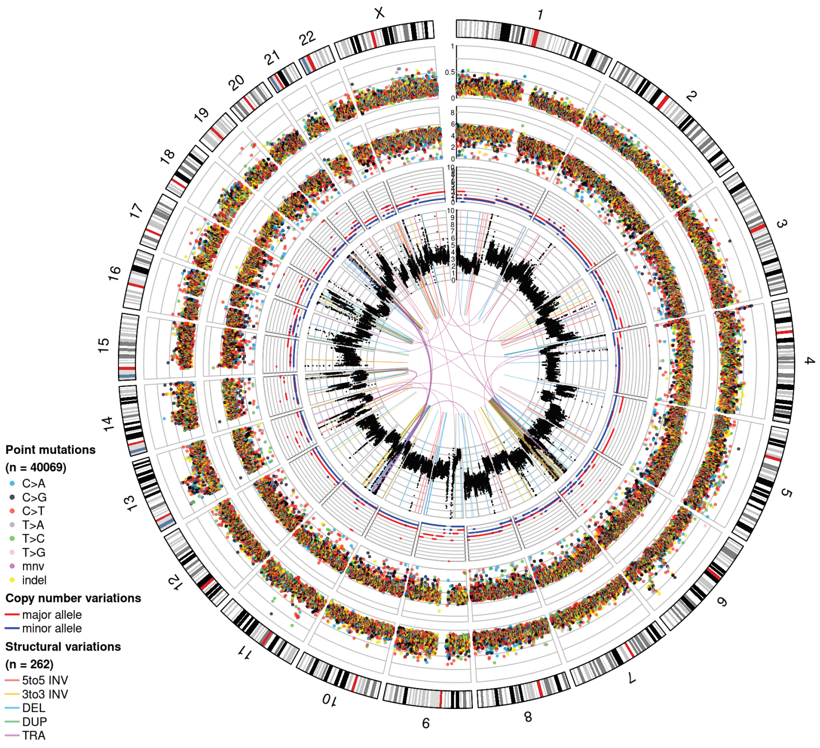

Case 90	F/59	Brain tumor	Utility: No (Category 1-1)
**Comments for Clinical Utility**	NF1 Pathogenic in-frame deletion confirmed by driver mutation. EGFR CNV (copy number 5) was reported on panel NGS (TruSight oncology 500), but no CNV in EGFR was identified on WGS. For NF1, a SNV of the same profile was identified with a variant allele frequency of 76.9% and reported as tier III, which was included with other germline variants, making it less likely to implicate a driver mutation. No actionable driver was reported.		

Clinical Presentation> A 59-year-old woman with no unusual findings other than hypertension presents to the emergency department with dysarthria for one week and right leg weakness for two days. A brain MRI showed a 5.6 cm necrotic rim-enhancing mass in the right frontal lobe and multifocal infiltrative masses in the left insular, frontal, and thalamic lobes. Streotactic surgery was performed to remove the tumor, and histology confirmed a high-grade glioma, WHO grade 4. The guardian refused further radiotherapy and surgery and continued rehabilitation. Six months after surgery, he was hospitalized for pneumonia, his disease worsened, and he died seven months after diagnosis.

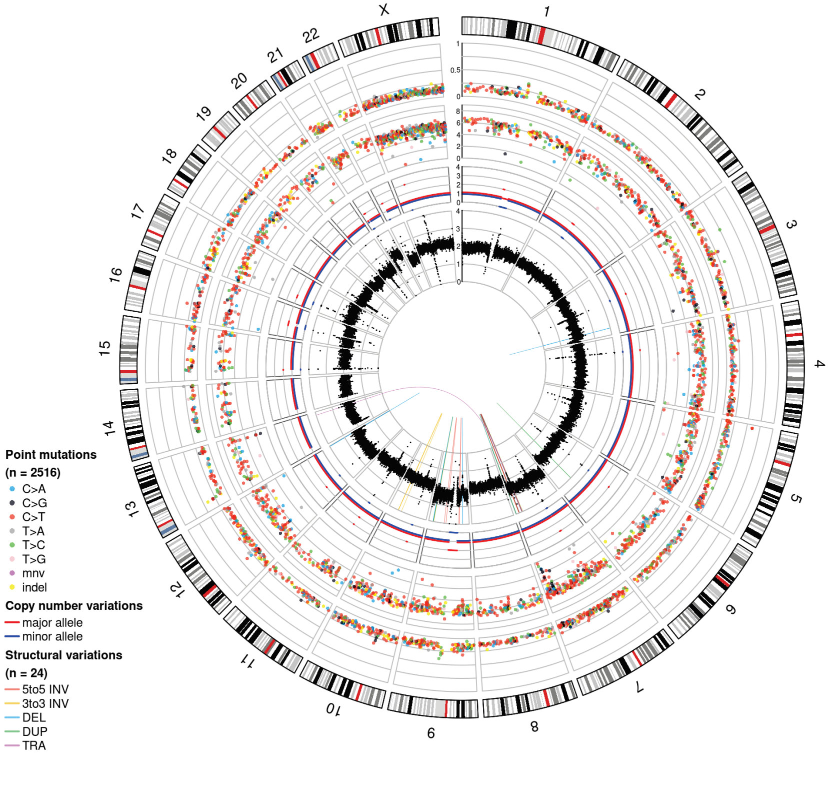

**Genome interpretation>**	Depth: Tissue: 40.3X, Normal: 22.5X	Total mutation counts: SNV: 2,348 Indel: 169 SV: 24
	Tumor fraction: 37, Mean ploidy: 1.9	Germline: not specific findings

Pathogenic inframe deletion of NF1 has been identified.

**Case 91**	M/51	**Renal cell carcinoma**	**Utility: No (Category 1-1)**
**Comments for Clinical Utility**		Multiple myeloma and metastatic renal cell carcinoma were identified. No actionable driver mutations were identified, and an aristolochic acid-related mutational process (SBS 22) was identified in the mutational signature. The patient's occupational and drug exposures did not identify any exposure to aristolochic acid. No actionable driver was reported.	

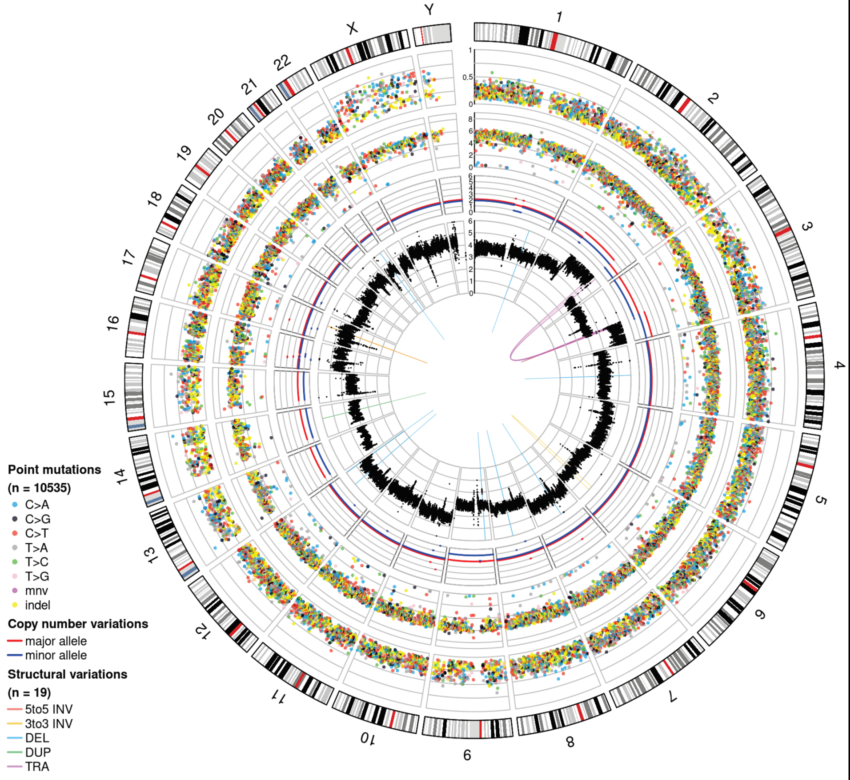

Clinical Presentation> A 51-year-old man with no significant medical history presented to the emergency department with vomiting and was diagnosed and treated for acute kidney injury with BUN/creatinine 49.3/7.38 (mg/dl). The diagnosis was symptomatic multiple myeloma, light chain disease, kappa type. A left kidney mass was identified and histology was performed, and renal cell carcinoma (RCC) of clear cell type was diagnosed, with liver, lung, and multiple bone metastases identified. The bone lesion was confirmed as renal cell carcinoma by histological examination. There was no family history of cancer, and the patient works as a forklift operator in a warehouse.
The metastatic RCC was classified as IMDC intermediate risk and nivolumab + ipilimumab was started. After 8.5 months of treatment, progression of lung metastases was confirmed and axitinib was started as second-line therapy, which remained progression-free after 2 months of treatment. For myeloma, he received 6 cycles of bortezomib + dexamethasone and achieved a very good partial response on response assessment. He is on active surveillance for myeloma and has been stable for 3 months.

**Genome interpretation>**	Depth: Tissue: 41.4X, Normal: 18.5X	Total mutation counts: SNV: 8,274 Indel: 2,101 SV: 18
	Tumor fraction: 70, Mean ploidy: 5.3	Germline: not specific findings

VHL (p.Asn131Tyr) mutation was identified. Mutational signature analysis revealed a proportion of aristolochic acid-related mutational process.

Case 92	M/78	**Urothelial carcinoma**	**Utility: No (Category 1-1)**
**Comments for Clinical Utility**	No actionable driver was reported.		

Clinical Presentation> A 78-year-old man with a history of prostate cancer diagnosed 1 year ago and treated with high-intensity focused ultrasound was diagnosed with urothelial carcinoma originating from the upper ureter with sternal bone metastases. He received palliative chemotherapy with gemcitabine plus cisplatin with a partial response as best response, but progressed to multiple bone metastases after 4 cycles. He received pembrolizumab as second-line therapy, but was switched to paclitaxel as third-line therapy due to disease progression at first response assessment after 3 cycles. The patient elected best supportive care due to worsening systemic condition and pneumonia after 1 cycle, and died of complicated urinary tract infection and pneumonia 13.1 months after the initiation of systemic chemotherapy.

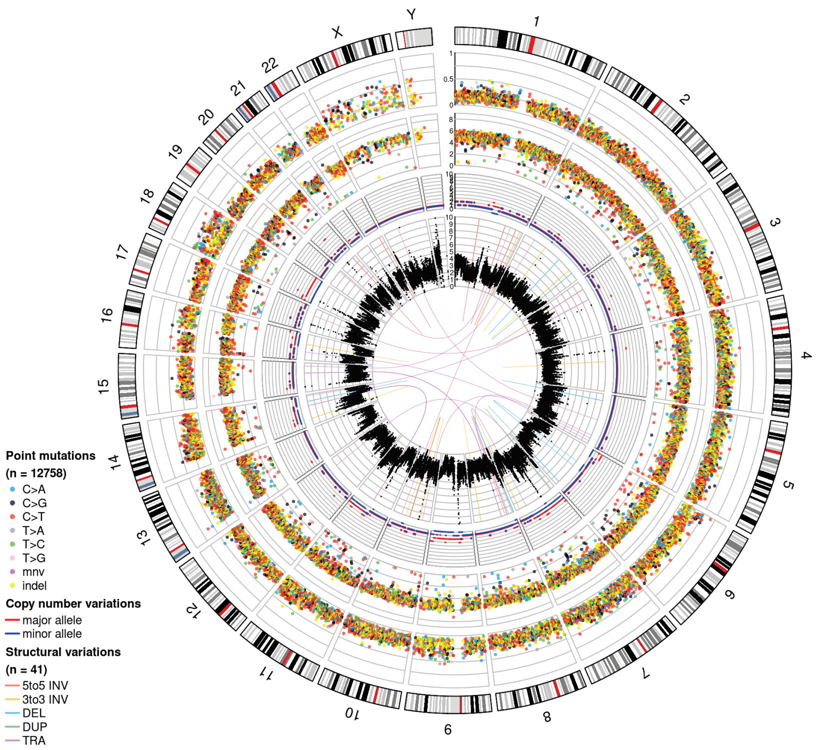

**Genome interpretation>**	Depth: Tissue: 58.7X, Normal: 17.2X	Total mutation counts: SNV: 9,417 Indel: 3,343 SV: 41
	Tumor fraction: 40, Mean ploidy: 2.4	Germline: not specific findings

TERT promotor mutation has been identied. A high proportion of APOBEC signature was noted.

Case 93	M/67	Prostate cancer	Utility: Category I-1
**Comments for Clinical Utility**	BRCA2 has a frame shift variant and loss of heterozygosity is confirmed. In prostate cancer, BRCA2 mutation is an actionable vatiant that allows the use of PARP inhibitors such as olaparib and niraparib. **[Category I-1 Selection of targeted therapeutics]**		

Clinical Presentation> A patient with underlying medical conditions of hypertension, diabetes mellitus, alcoholic liver cirrhosis, who presented with worsening systemic condition, was diagnosed with prostate cancer with bone metastases during evaluation and management of liver cirrhosis.(Serum PSA level 34.1 ng/ml) Anti-deandrogen therapy and upfront docetaxel therapy were performed for high-volume, de novo metastatic hormone-sensitive prostate cancer with Gleason score 5+5. After 4 cycles of chemotherapy, PSA decreased to 0.299 ng/mL and systemic symptoms improved. However, the patient was intolerant to cytotoxic chemotherapy due to underlying hepatic decompensation and was switched to apalutamide therapy, which he continues to receive.

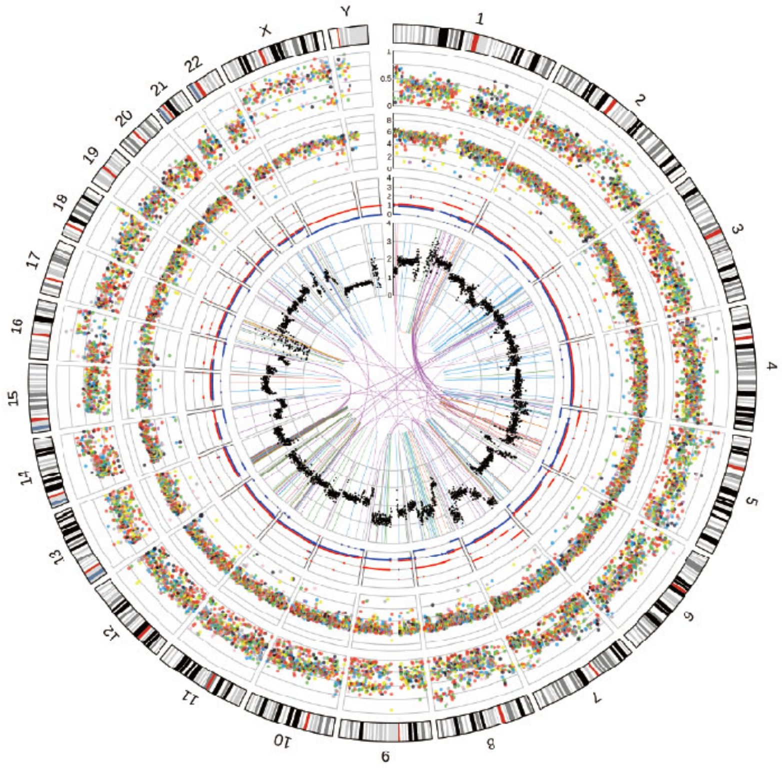

**Genome interpretation>**	Depth: Tissue: 45.9X, Normal: 20.3X	Total mutation counts: SNV: 1,0119 Indel: 1,0544 SV: 228
	Tumor fraction: 78, Mean ploidy: 2.1	Germline: not specific findings

ERG-TMPRSS2 fusion gene has been identified. This sample had homologous recombination deficiency (HRD score 0.99) and somatic BRCA1 p.G12fsTer4 mutation associated with loss of heterozygosity.

**Case 94**	F/47	**Gastrointestinal stromal tumor**	**Utility: Category I-1**
**Comments for Clinical Utility**	A KIT in-frame (p.Trp557_Thr574delinsSer) deletion was identified and the exon 11 deletion is known to be an imatinib sensitive variant. **[Category I-1 Selection of targeted therapeutics]**		

Clinical Presentation> In a patient who underwent tumor resection for a duodenal gastrointestinal tumor 20 years ago, CT imaging revealed a 9 cm mass in liver segments 7 and 8. Right hemihepatectomy was performed. multiple nodules of 5mm or less in the lung were further identified, which were considered as metastases by serial CT imaging. Palliative imatinib therapy was initiated and she has maintained a response without progression for 7 months.

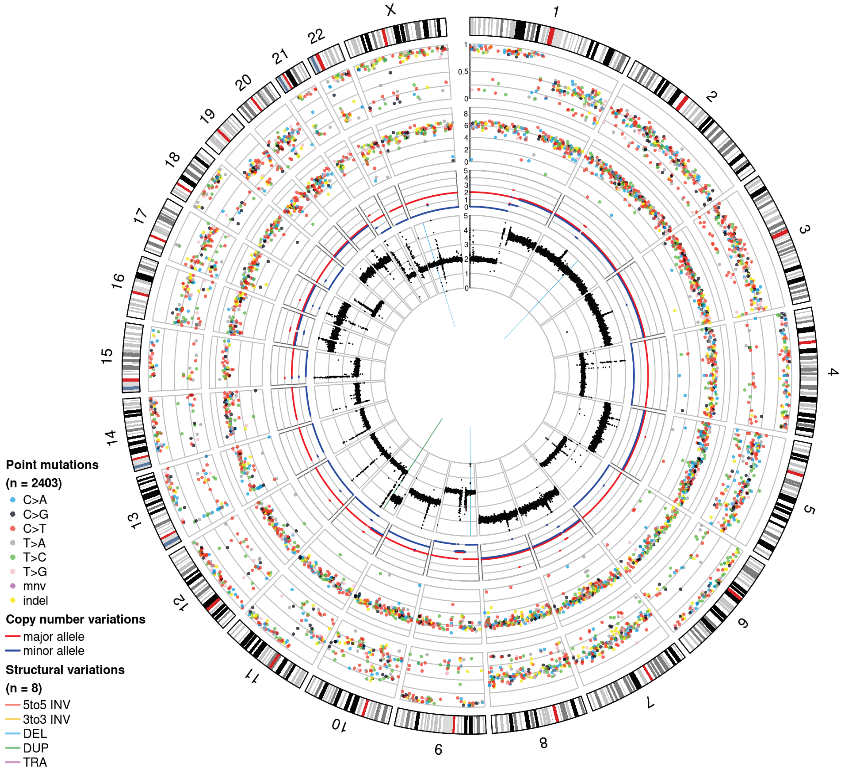

**Genome interpretation>**	Depth: Tissue: 36.9X, Normal: 18.0X	Total mutation counts: SNV: 2,181 Indel: 223 SV: 8
	Tumor fraction: 96, Mean ploidy: 3	Germline: not specific findings

Somatic KIT inframe deletion (p.Trp557_Thr574delinsSer) has been identified.

**Case 95**	F/50	**Colorectal cancer**	**Utility: Category II-2**
**Comments for Clinical Utility**		The alterations identified were not distinct enough to suggest that the patient's colon cancer was familial. **[Category II-2 Tumor orgin]**	

Clinical Presentation> A 50-year-old woman with no significant past medical history was referred to a tertiary center for sigmoid colon cancer findings on colonoscopy performed for hematochezia.
A fungating mass obstructing the lumen was identified in the sigmoid colon, 28 cm from the anal verge, and a moderately differentiated adenocarcinoma was reported. CT imaging showed distant lymph node metastases in the left paraaortic space and clinical staging confirmed cT4aN+M1. The patient's mother also had a history of surgery for colorectal cancer. Laparoscopic anterior resection was planned, and surgical findings confirmed adjacent left ovary, so en bloc resection of the left ovary was also performed. Postoperative pathology confirmed moderately differentiated adenocarcinoma with lymphatic, venous, and perineural invasion, with fibrous adhesions in the left adnexa but no tumor invasion, and pathologic staging was pT4N2bM1. Postoperative CT imaging revealed a new liver mass measuring 1.6 cm, and FDG-PET-CT showed findings consistent with metastasis. At 5 weeks post-surgery, palliative bevacizumab + mFOLFOX6 was administered, best response was stable disease, and after 12 cycles, there was evidence of sigmoid colon surgical site progression, so bevacizumab + FOLFIRI is administered as second-line chemotherapy. It has been 9 months since surgery and he is currently on follow-up chemotherapy.

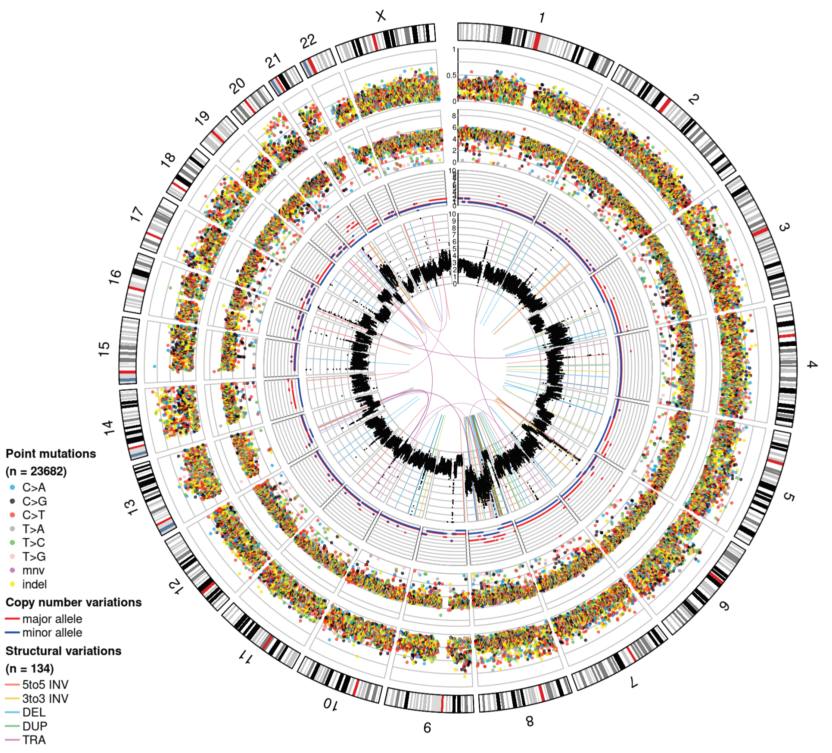

**Genome interpretation>**	Depth: Tissue: 48.5X, Normal: 18.2X	Total mutation counts: SNV: 18,602 Indel: 5,082 SV: 134
	Tumor fraction: 62, Mean ploidy: 2.4	Germline: not specific findings

High proportion of APOBEC signature was observed.
